# Genome-wide analysis reveals signatures of selection for important traits in domestic sheep from different ecoregions

**DOI:** 10.1186/s12864-016-3212-2

**Published:** 2016-11-03

**Authors:** Zhaohua Liu, Zhibin Ji, Guizhi Wang, Tianle Chao, Lei Hou, Jianmin Wang

**Affiliations:** Shandong Provincial Key Laboratory of Animal Biotechnology and Disease Control and Prevention, College of Animal Science and Technology, Shandong Agricultural University, Taian, Shandong 271018 China

**Keywords:** Sheep, Microevolution, Whole-genome resequencing, Selection signal, Reproduction

## Abstract

**Background:**

Throughout a long period of adaptation and selection, sheep have thrived in a diverse range of ecological environments. Mongolian sheep is the common ancestor of the Chinese short fat-tailed sheep. Migration to different ecoregions leads to changes in selection pressures and results in microevolution. Mongolian sheep and its subspecies differ in a number of important traits, especially reproductive traits. Genome-wide intraspecific variation is required to dissect the genetic basis of these traits.

**Results:**

This research resequenced 3 short fat-tailed sheep breeds with a 43.2-fold coverage of the sheep genome. We report more than 17 million single nucleotide polymorphisms and 2.9 million indels and identify 143 genomic regions with reduced pooled heterozygosity or increased genetic distance to each other breed that represent likely targets for selection during the migration. These regions harbor genes related to developmental processes, cellular processes, multicellular organismal processes, biological regulation, metabolic processes, reproduction, localization, growth and various components of the stress responses. Furthermore, we examined the haplotype diversity of 3 genomic regions involved in reproduction and found significant differences in TSHR and PRL gene regions among 8 sheep breeds.

**Conclusions:**

Our results provide useful genomic information for identifying genes or causal mutations associated with important economic traits in sheep and for understanding the genetic basis of adaptation to different ecological environments.

**Electronic supplementary material:**

The online version of this article (doi:10.1186/s12864-016-3212-2) contains supplementary material, which is available to authorized users.

## Background

**Fig. 1:**
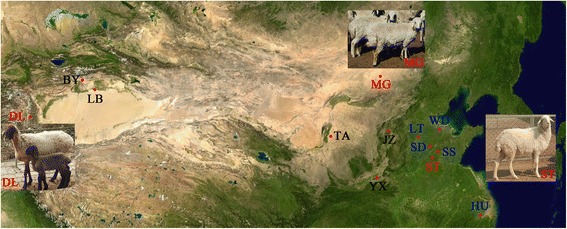
Geographic distribution of Chinese short fat-tailed sheep. MG, DL, LB, BY, JZ, TA, YX, ST, LT, WD, SD, SS and HU are abbreviations for Mongolian sheep, Duolang sheep, Luobu sheep, Buyinbuluk sheep, Jinzhong sheep, Tan sheep, Yuxi fat-tailed sheep, Small-tailed Han sheep, Large-tailed Han sheep, Wadi sheep, Luzhong Shandi sheep, Sishui Fur sheep and Hu sheep, respectively. The 3 breeds used for whole-genome resequencing are labeled in red, and the additional 5 breeds used for genotyping are labeled in *blue*. This figure has been modified from China 100.78713E 35.63718 N.jpg (https://commons.wikimedia.org/wiki/File:China_100.78713E_35.63718N.jpg). This image is in the public domain because it is a screenshot from NASA’s globe software World Wind using a public domain layer, such as Blue Marble, MODIS, Landsat, SRTM, USGS or GLOBE

Sheep were initially reared mainly for meat and were subsequently specialized for other products approximately 4000–5000 years ago. With the development of animal husbandry and the application of directed mating technology, phenotypic radiation under selection has resulted in the spectrum of modern sheep breeds, adapted to a diverse range of environments and specialized for the production of meat, milk, and wool [1, 2]. China has a long history of sheep domestication and rich resources of sheep breeds [3, 4]. Based on tail type, the Chinese domesticated sheep can be divided into five types: short fat-tailed sheep, long fat-tailed sheep, short thin-tailed sheep, long thin-tailed sheep and buttock-tailed sheep. According to archaeological and genetic research, Mongolian sheep is the common ancestor of Chinese short fat-tailed sheep breeds. Mongolian sheep evolved from the wild Argali sheep in the mountain regions of Central Asia. More than 2000 years ago, with the development of free trade, inter-ethnic war and the southward migration of steppe tribes, a large number of populations had moved south of the Great Wall. As a result, Mongolian sheep also migrated to North China villages and were introduced into Gansu, Xinjiang, Qinghai, Shandong and other provinces. Thus, most modern Chinese sheep breeds have a relationship to Mongolian sheep. Small-tailed Han sheep, Duolang sheep, Hu sheep, Tan sheep and others are all Mongolian sheep subspecies [5] (Fig. 1). However, from the Mongolian plateau to various ecoregions around almost the entire country, Mongolian sheep have experienced changes in climate, environment and feeding conditions (from pastoral areas to rural areas) and been subjected to artificial selection in different directions [6]. All of these factors have the potential to drive changes in selection and thereby cause microevolution [7]. The subspecies of Mongolian sheep show significant differences in a number of traits, especially related to reproduction, but how species differ genetically in relation to these traits is not well understood [5]. Chinese short fat-tailed sheep are an excellent model for genetic studies on phenotypic evolution and adaptations to various ecoregions.

In recent years, many studies have analyzed the genetic changes between wild and domestic animals, between species adapted to extreme environments and common domestic species and among different local breeds with phenotypic diversity. These studies have identified genes with key roles in domestication [8–12], adaptation to extreme environments [13–16] or prominent economic traits [17–24]. Studies on the domestication of livestock have demonstrated that genes affecting brain and neuronal development have often been targeted [8]. In addition, a striking selective sweep at the locus for thyroid-stimulating hormone receptor (TSHR), which likely affects seasonal reproduction, was identified in domestic chicken [9]. A coat color locus, *MC1R*, was identified in wild and domestic pigs [10]. A set of genes with key roles in starch digestion separating wolves from dogs were identified [11]. Research on domestic horse breeds has provided evidence of the importance of *MSTN* in racing breeds and selection for gait and size [12]. In the Tibetan Plateau, many species have become popular research topics, such as the Tibetan wild boar [13], Tibetan Mastiffs [14, 15] and ground tit [16]. A number of genes associated with adaptation to high altitudes and hypoxia have been revealed. To understand the molecular basis underlying phenotypic variation in economically important traits in livestock, many studies have also focused on genome-wide genetic variations among different local breeds, such as chicken [17], pig [18, 19] and cattle [20–24]. However, in Chinese short fat-tailed sheep, there have been few similar studies.

In this study we performed pooled whole-genome resequencing of 3 sheep breeds (Mongolian sheep, Small-tailed Han sheep and Duolang sheep) distributed over a wide range of geographical distance to examine the genetic variation among them. This effort identified a large number of single-nucleotide polymorphisms (SNPs) and short sequence insertions and deletions (indels) in sheep. Comparison of these variations defined potential genomic regions and metabolic pathways associated with important biological functions. This study revealed different signals of selection in the 3 sheep breeds and focused on the genetic relationships between Mongolian sheep and the two subspecies. Furthermore, to validate the presence of selection, we examined the haplotype diversity of 3 regions showed evidence of selective pressure related to reproduction among 8 sheep breeds. The substantial genomic resources provided here are useful for identifying genetic variations for phenotypic diversity and for revealing different signatures of selection associated with adaptation to various ecoregions.

## Results

### Data production

**Fig. 2:**
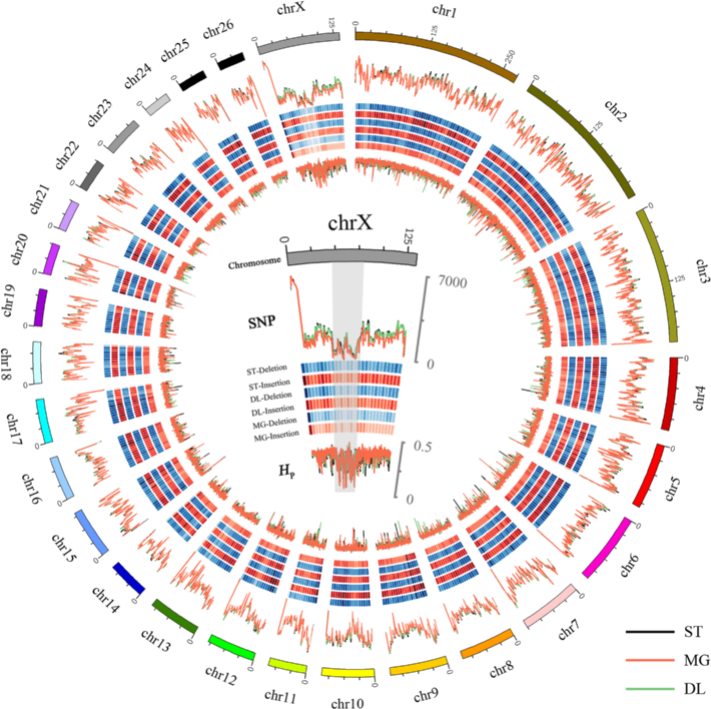
Summary of genome-wide genetic variation in the 3 Chinese short fat-tailed sheep breeds. Chromosomes are shown in different colors in the outermost circle, and the innermost circles show the distribution of SNPs (counted in 1-Mb windows), indels (counted in 5-Mb windows) and pooled heterozygosity (*H*_P_) in 200-kb windows of the Small-tailed Han sheep (ST), Mongolian sheep (MG) and Duolang sheep (DL) genomes relative to the sheep reference genome. For SNPs and *H*_P_ scores, *black* represents Small-tailed Han sheep, red represents Mongolian sheep, and green represents Duolang sheep. At the center of the circular map, chr. X is shown separately. A remarkably homozygous region (in *gray shadow*) was observed from 43 to 78 Mb in Chinese short fat-tailed sheep. This figure was created using the Circos program [60]

In total, more than 480 million 2 X 100-bp paired-end reads were generated and aligned to the sheep reference sequence (USUC oar_ref_Oar_v3.1) with a genome size of 2,587,507,083 bp. Thus, we achieved a 43.2-fold coverage of the reference genome, with a 14.4 X average sequencing depth for each breed. We identified 17,420,695 putative SNPs and 2,912,131 indels for the three breeds (Fig. 2, Additional file 1: Table S1; Additional file 2: Table S2; Additional file 3: Figure S1). We were able to experimentally validate 100 out of 102 tested SNPs (Additional file 4: Table S3).

### SNP annotations

The numbers of SNPs and indels in the coding sequences (CDS) of the 3 breeds are shown in Additional file 5: Table S4. Small-tailed Han sheep and Duolang sheep both exhibit year-round estrous and prolificacy in breeding, whereas Mongolian sheep exhibits seasonal estrous and singleton breeding. Thus, we identified 3105 genes that contained missense SNPs or stop gained/loss variants in Mongolian sheep but not in Small-tailed Han sheep or Duolang sheep. And we also identified 803 genes that contained missense SNPs or stop gained/loss variants in both Small-tailed Han sheep and Duolang sheep, but not in Mongolian sheep. We speculated that these genes might contribute to the phenotypic differentiation. We searched for significantly overrepresented gene ontology (GO) terms in the above two groups of genes respectively and identified several categories (Additional file 6: Table S5; Additional file 7: Table S6; Additional file 8: Figure S2). In the former group one conspicuous cluster related to reproduction; 59 genes belonged to this category (13 genes were related to single fertilization and 24 genes were related to spermatogenesis). Another conspicuous cluster with 284 genes was related to response to stimulus (Table 1 and Additional file 6: Table S5); one of these genes *TRPM8* has been shown to be a major determinant of cold perception in the mouse [25] and was reported to be related to adaptation to cold climate on sheep [26]; 12 of the 284 genes were related to response to light stimulus and we suggested these genes might had potential relationship with seasonal estrous. In the latter group one conspicuous cluster related to reproduction as well; 15 genes belonged to this category (12 genes were related to spermatogenesis). Ten Kyoto Encyclopedia of Genes and Genomes (KEGG) pathways were also significantly enriched in the former group genes; four ones were enriched in the latter group genes among which “Progesterone-mediated oocyte maturation” contained 7 genes (*RPS6KA3*, *MAD2L1*, *CCNB2*, *GNAI2*, *ADCY5*, *PIK3R5*, *CDC25B*) that may be related to the reproductive traits of short fat-tailed sheep [27] (Additional file 9: Table S7; Additional file 10: Table S8; Additional file 8: Figure S2). We also identified 3916 genes that contained SNPs in promoter region (1 kb up- or downstream of genes) in Mongolian sheep but not in Small-tailed Han sheep or Duolang sheep and 136 genes that contained SNPs in promoter region (1 kb up- or downstream of genes) in both Small-tailed Han sheep and Duolang sheep, but not in Mongolian sheep. The GO term enrichment/KEGG analysis indicated that in the former 3916 genes, one conspicuous cluster containing 53 genes were related to reproduction (10 genes were related to fertilization) and another cluster containing 12 genes were related to rhythmic process (9 genes were related to circadian rhythm) (Additional file 11: Table S9; Additional file 12: Table S10; Additional file 13: Table S11; Additional file 14: Figure S3). These genes might be associated with the differences in reproductive performance of short fat-tailed sheep as well. In addition, the functional effects of missense SNPs were further investigated with SIFT and Provean. The result indicated 565, 490 and 320 SNPs were detected in protein coding genes from Mongolian sheep, Small-tailed Han sheep and Duolang sheep, respectively (Additional file 15: Table S12).

**Table 1:** Enriched gene ontology terms related to response to stimulus among genes containing unique missense SNPs or stop gained/loss variants in Mongolian sheep

Gene ontology term	Gene count	*P* value
Response to stress;	140#1222	3.06488E-11
Response to external stimulus;	85#633	4.65E-11
Response to wounding;	61#423	1.46E-09
Response to chemical stimulus;	73#589	1.39E-07
Response to hypoxia;	13#48	0.000115
Response to stimulus # regulation of response to stimulus;	9#35	0.003204
Response to biotic stimulus;	33#289	0.007919
Response to radiation;	15#101	0.010492
Response to abiotic stimulus;	21#165	0.014159
Response to drug;	11#63	0.017014
Response to lipopolysaccharide;	3#5	0.018798
Response to bacterium # response to molecule of bacterial origin;	4#10	0.019823
Response to fungus # response to molecule of fungal origin;	2#2	0.027385
Response to protein stimulus;	10#59	0.027385
Response to unfolded protein;	10#59	0.027385
Response to light stimulus;	12#81	0.028201

### Selective sweep analysis

**Fig. 3:**
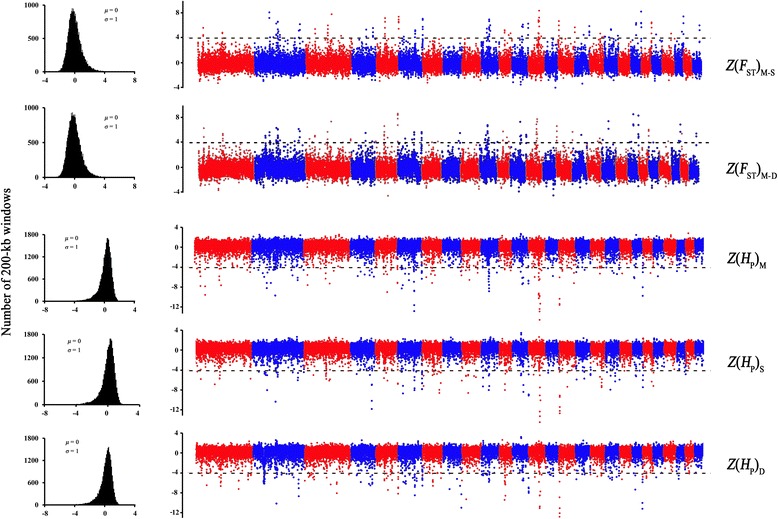
Selection analyses identified selection signals in 3 Chinese short fat-tailed sheep breeds. The distributions of Z-transformed average pooled heterozygosity (*H*_P_) as well as the average fixation index between any two breeds for autosomal 200-kb windows (σ, standard deviation; μ, average) are shown on the left. The Z-value distribution plotted along sheep autosomes 1–26 (chromosomes are separated by *red* and *blue* coloring) is shown on the right. The dashed horizontal line indicates the cut-off (Z > 4 or Z < -4) used for extracting outliers

To search genomic regions under selection during the migration of the short fat-tailed sheep, we measured the pooled heterozygosity (*H*_P_) in 200-kb windows, with half-step sliding along the genomes of the three breeds, and the fixation index (*F*_ST_) between any two breeds also in 200-kb windows with half-step sliding. The distributions of *H*_P_ and *F*_ST_ values, as well as the Z transformations of these values, Z(*H*_P_) and Z(*F*_ST_), for the 3 sheep breeds are plotted in Fig. 3, Additional file 16: Figure S4 and Additional file 17: Figure S5 (the Z transformation of *H*_P_ in Mongolian sheep, Small-tailed Han sheep and Duolang sheep are Z(*H*_P_)_M_, Z(*H*_P_)_S_ and Z(*H*_P_)_D_ for short, respectively; the Z transformation of Z(*F*_ST_) between Mongolian sheep and Small-tailed Han sheep, between Mongolian sheep and Duolang sheep as well as between Small-tailed Han sheep and Duolang sheep are Z(*F*_ST_)_M-S_, Z(*F*_ST_)_M-D_ and Z(*F*_ST_)_S-D_ for short, respectively). The skewed distributions of the *H*_P_ and *F*_ST_ scores indicated the existence of selection on these sheep breeds. The windows with Z(*H*_P_) < -4 or (and) Z(*F*_ST_) > 4 were considered the target windows because these windows were at the extreme ends of the distribution (Fig. 3). Figure 3 shows intuitive evidence of different selection signals in each autosome among the 3 breeds. For example, on chromosome (chr.) 10, there were several windows with a low Z(*H*_P_)_M_, and high Z(*F*_ST_)_M-S_ and Z(*F*_ST_)_M-D_, suggesting that a high selection signal was located on this chromosome. This finding is consistent with a previous study in which the genome-wide distribution of global *F*_ST_ for 49,034 SNPs from 75 sheep breeds all over the world revealed the highest selection signal detected on chr. 10 [2]. The highest ranked SNP was located at Mb position 29.54 near Relaxin/insulin-like family peptide receptor 2 (*RXFP2*), which is linked to the absence of horns (poll) in sheep [2]. In our research, *RXFP2* was also found in the selection region of Small-tailed Han sheep (*F*_ST_ = 0.514, Z(*F*_ST_)_M-S_ = 4.08).

We identified regions of selection in the genomes of the two subspecies Small-tailed Han sheep and Duolang sheep. As a result, in the autosomes of Small-tailed Han sheep, 108 regions with extremely high levels of homozygosity and 65 regions with increased genetic distance to Mongolian sheep were identified. In total, 134 regions containing 774 protein-coding genes were identified (Additional file 18: Table S13). Interestingly, the highest *H*_P_ and the lowest *F*_ST_ were located in the same window: 53.3–53.5 Mb on chr. 13 (*F*_ST_ = 0.692, Z(*F*_ST_) = 8.158; *H*_P_ = 0.0597, Z(*H*_P_) = -14.091). This window harbors 10 protein-coding genes, *ZBTB46*, *ZGPAT*, *ARFRP1*, *RTEL1*, *STMN3*, *GMEB2*, *C20orf195*, *SRMS*, *PTK6* and *EEF1A2*, of which *RTEL1* and *EEF1A2* are related to the inhibition of apoptosis and programmed cell death. On chr. X, 9 regions containing 24 protein-coding genes were identified by applying a threshold of Z(*H*_P_) < -3 or (and) Z(*F*_ST_) > 3 (Additional file 18: Table S13). One of these genes, *AR*, which encodes the androgen receptor, is essential for prostate gland development, urogenital system development and reproductive development. In the whole genome, 143 regions with a total length of 45.7 Mb accounted for 1.77 % of the entire genome. Similarly, in the genome of Duolang sheep, 143 regions (134 autosomal regions and 9 regions on chr. X) with a total length of 51.7 Mb accounted for 2.0 % of the entire genome (Additional file 19: Table S14). There were 74 regions that overlapped each other between the 2 breeds, accounting for 25.1 Mb.

**Fig. 4:**
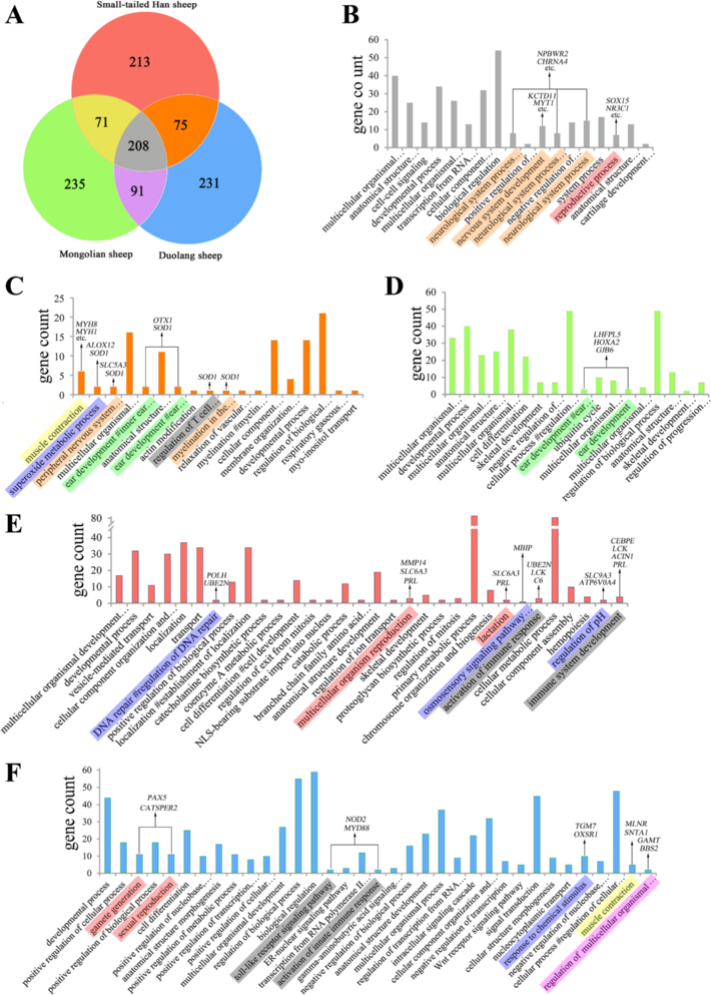
Biological processes enrichment of genes located in regions with a Z(*H*_P_) < -4. **a** All of the genes in regions with a Z(*H*_P_) < -4 were divided into 3 groups: I, Z(*H*_P_)_M_ < -4 (605 genes); II, Z(*H*_P_)_S_ < -4 (567 genes); and III, Z(*H*_P_)_D_ < -4 (605 genes). **b**, **c**, **d**, **e** and **f** indicate the biological process enrichment of genes belonging to all 3 groups; belonging to both group II and III; belonging to only group I; belonging to only group II and belonging to only group III. Important enriched terms are color coded to reflect the relatedness of biological processes. *Orange*, nervous system development; *red*, reproductive process; *yellow*, muscle contraction; *blue*, various processes related to stress responses; *green*, ear development; *grey*, immune system development and regulation; *pink*, regulation of multicellular organism growth

We divided all of the protein-coding genes residing in the genomic regions with a Z(*H*_P_) < -4 into 3 groups: I, Z(*H*_P_)_M_ < -4 (605 genes); II, Z(*H*_P_)_S_ < -4 (567 genes); and III, Z(*H*_P_)_D_ < -4 (605 genes) (Fig. 4a), and then analyzed significantly enriched GO terms among these groups. In the 208 genes belonging to all the 3 groups, nervous system development, reproductive processes and other biological functions were enriched (Fig. 4b). These findings were in agreement with the notion that genes affecting brain and neuronal development have often been targeted during animal domestication [8]. In addition, 5 genes involved in ear development were pinpointed in the 3 sheep breeds: *OTX1* and *SOD1*, in both group II and III (Fig. 4c), and *LHFPL5*, *HOXA2* and *GJB6*, in group I (Fig. 4d). This result indicated that Chinese indigenous sheep breeds might have been subjected to different selection for ear development than Texel sheep, which were sequenced for the sheep reference genome. In relation to adaptation to different ecoregions, the three Chinese short fat-tailed sheep breeds show different selection signals. Among the subspecies, Small-tailed Han sheep and Duolang sheep both show signals of selection for immune system development and regulation, reproduction, muscle contraction and stress responses (Fig. 4c). The stress responses could be divided to two kinds: first, superoxide metabolic processes including genes *ALOX12* and *SOD1* in both group II and III (Fig. 4c); second, DNA repair including genes *POLH* and *UBE2N* only in group II (Fig. 4e) and the response to chemical stimuli including genes *TGM7* and *OXSR1* only in group III (Fig. 4f).

The protein-coding genes in the regions of selection with a Z(*F*_ST_)_M-S_ > 4 or (and) Z(*F*_ST_)_M-D_ > 4 were also compared and used to detect significantly enriched GO terms. A total of 361 genes located in regions with a Z(*F*_ST_)_M-S_ > 4 and a Z(*F*_ST_)_M-D_ > 4 were related to nervous system development, ear development, fatty acid biosynthetic and metabolic processes, regulation of growth, sexual reproduction and other biological processes (Additional file 20: Table S15). Additionally, 91 genes located in regions with a Z(*F*_ST_)_M-S_ > 4 were related to vesicle-mediated transport, protein digestion, regulation of protein polymerization, chemical homeostasis, reproductive processes and other biological processes (Additional file 21: Table S16), while 43 genes located in regions with a Z(*F*_ST_)_M-D_ > 4 were related to spermatogenesis, single fertilization, post-translational protein modification, regulation of protein kinase activity, regulation of fatty acid beta-oxidation, blastoderm segmentation, cell proliferation and other biological processes (Additional file 22: Table S17).

Interestingly, gene *HOXA10* met both Z(*F*_ST_)_M-S_ > 4 and Z(*H*_P_)_M_ < -4 (Z(*F*_ST_)_M-S_ = 4.168, Z(*H*_P_)_M_ = -5.39), and also contained missense SNPs in both Small-tailed Han sheep and Duolang sheep, but not in Mongolian sheep. In previous reports *HOXA10* is a well-known transcriptional factor gene, shown to be one of the most promising candidate genes that play major roles in endometrial differentiation and development, establishing the conditions required for implantation and normal pregnancy maintenance [28] and have been widely studied in human, mouse and other species [29–33]. In this research, *HOXA10* showed strong selection signatures in Chinese short fat-tailed sheep breeds. We suggested this gene is an important factor causing the differences in reproductive performance among Chinese short fat-tailed sheep breeds.

On chr. X, we found that the average *H*_P_ score was lower (*H*_P_-chrX = 0.371 < *H*_P_-chrA = 0.386) and the average *F*_ST_ score was higher (*F*_ST_-chrX = 0.427 > *F*_ST_-chrA = 0.337) than the autosomes (Additional file 16: Figure S4 and Additional file 17: Figure S5). Furthermore, we noted that the distributions of the standard deviations of *H*_P_ (σX = 0.049 > σA = 0.023) and *F*_ST_ (σX = 0.089 > σA = 0.043) were larger on chr. X. As shown in Additional file 17: Figure S5, a larger proportion of the windows resided in the tails of the distributions on chr. X compared to the autosomes.

### Three putative sweeps related to reproductive traits

**Fig. 5:**
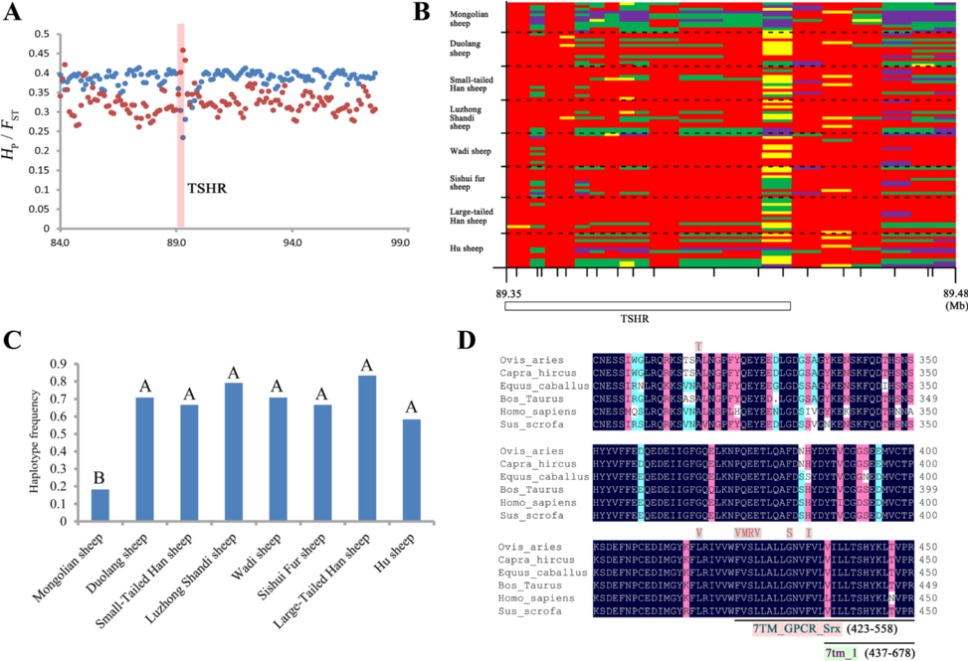
The haplotype diversity and candidate mutations of *TSHR*. **a** Pooled heterozygosity, *H*_P_ (in *blue*), and average fixation index between Mongolian sheep and Small-tailed Han sheep, *F*_ST_ (in *red*), plotted for 200-kb windows spanning the region harboring *TSHR* (in *pink shadow*) on chr. 7. **b** Genetic variation in the region 89.35–89.48 Mb on chr. 7 across partial *TSHR*. Individual sheep (95 from 8 breeds) were genotyped using WaferGen genotyping. *Dashed horizontal lines* separate the 8 breeds. At the *bottom* of the figure, short tick marks represent individual SNPs. *Long tick marks* indicate the position in Mb. *Red color*: homozygous A-allele; *green color*: heterozygous; *purple color*: homozygous a-allele; *yellow color*: missing genotype call. **c** The haplotype frequencies of the 8 sheep breeds. The haplotype frequency of Mongolian sheep was significantly less than those of the other 7 breeds (*p* < 0.01). **d** A total of 8 missense variants in *TSHR* were identified by an amino acid sequence comparison (300–450) from 6 species: *Ovis aries*, *Capra hircus*, *Equus caballus*, *Bos Taurus*, *Homo sapiens* and *Sus scrofa. Asterisks*, *double dots*, and *single dots* denote fully, strongly, and weakly conserved residues, respectively. Conserved Protein Domain: 7TM_GPCR_Srx and 7tm_1 are both *underlined*

Reproductive traits showed the most significant phenotypic differences among the Chinese short fat-tailed sheep breeds. Therefore, we chose 3 genomic regions related to reproduction to validate the different signals of selection among 8 sheep breeds. *TSHR* showed evidence of selective pressure in both Small-tailed Han sheep and Duolang sheep (Z(*H*_P_)_S_ = -6.55; Z(*H*_P_)_D_ = -4.9; Z(*H*_P_)_M_ = 0.6) and has been reported to have pivotal roles in metabolic regulation and photoperiod control of reproduction in vertebrates [9, 34] (Fig. 5a; Additional file 23: Figure S6). We studied haplotype diversity across the partial gene region (89.35–89.48 Mb on chr. 7) to validate its selection and genotyped 19 randomly selected SNPs in 95 individuals from 8 sheep breeds. These 95 tested individuals carried 123 copies of a 119-kb haplotype (Fig. 5b). The haplotype frequency of Mongolian sheep was significantly lower than those of the other 7 breeds (*p* < 0.01; Fig. 5c). This remarkable genetic diversity indicated that *TSHR* showed strong evidence of selective pressure. In selection signal analysis of sheep breeds from all over the world, positive selection was also detected surrounding *TSHR* [2, 26].

In addition to haplotype diversity, we identified 8 missense variants in the *TSHR* genic region, 7 of which were unique to Mongolian sheep (Duolang sheep also has p. 315Ala > Thr). As shown in Fig. 5d, p.423Phe > Val, p.424Val > Met, p.425Ser > Arg, p.426Leu > Val, p.431Gly > Ser and p.434Phe > Ile were located in the Conserved Protein Domain 7TM_GPCR_Srx (Serpentine type seven-transmembrane G-protein-coupled receptor class chemoreceptor Srx) and close to 7tm_1 (7-transmembrane receptor (rhodopsin family)). The seven-transmembrane structure has a decisive effect on the biological function of TSHR. Thus, these 6 missense variants were considered candidate causal mutations for the *TSHR* sweep. It is possible that selection for these variants could be associated with photoperiod control of reproduction in Chinese short fat-tailed sheep.

*PRL* (Z(*H*_P_)_S_ = -4.16; Z(*H*_P_)_D_ = -0.91; Z(*H*_P_)_M_ = -0.93) and *HMGCR* (Z(*H*_P_)_S_ = -5.14; Z(*H*_P_)_D_ = -2.42; Z(*H*_P_)_M_ = -1.57) showed evidence of selective pressure in Small-tailed Han sheep only (Additional file 24: Figure S7A and S7B). *PRL* encodes prolactin and is essential for female pregnancy and lactation. In sheep, the luteinizing hormone-secretory response to gonadotropin-releasing hormone is completely suppressed by the combined actions of PRL and dopamine in the nonbreeding season [35]. To characterize haplotype diversity among different sheep breeds, we genotyped 17 randomly chosen SNPs in this selection region and identified a 38.2-kb region (34.22–34.26 Mb on chr. 20) spanning the *PRL* section and the entire *LOC443319* encoding placental lactogen, which was highly divergent among the 8 tested sheep breeds (Additional file 24: Figure S7C). As shown in Additional file 24: Figure S7E, the haplotype frequencies of Small-tailed Han sheep, Luzhong Shandi sheep and Hu sheep were significantly higher than those of Mongolian sheep and Duolang sheep (*p* < 0.05). This result is in agreement with the *H*_P_ scores of the 3 resequenced breeds in this region and may demonstrate the selection of *PRL* in Small-tailed Han sheep. *HMGCR* encodes 3-hydroxy-3-methylglutaryl-CoA reductase and is a key enzyme in cholesterol biosynthesis. This gene is related to germ cell migration and gonad development. We screened 16 SNPs across the *HMGCR* region (6.53–6.57 Mb on chr. 7) using 95 individuals from 8 breeds. However, no significant difference was found among the haplotype frequencies of the 8 tested breeds (Additional file 24: Figure S7F). We speculated that the selection on *HMGCR* might be due to genetic variation in other regulatory regions and sequences.

## Discussion

In this study, we performed whole-genome resequencing of 45 sheep from 3 Chinese short fat-tailed breeds. We catalogued millions of SNPs from each breed to advance evolutionary and genetic research. This is the first whole-genome study to characterize genetic polymorphisms in Chinese short fat-tailed sheep breeds. Our selection analyses revealed that Mongolian sheep, Small-tailed Han sheep and Duolang sheep contain many common selection signatures unique to the Chinese short fat-tailed sheep and also many different selection signatures among their genomes. The 3 Chinese indigenous sheep breeds were suggested to have experienced differential selection for nervous system development, ear development and reproductive processes compared with Texel sheep, which were sequenced for the sheep reference genome. The regions of selection identified in Small-tailed Han sheep and Duolang sheep harbor different protein-coding genes with important biological functions, including roles in development, reproduction, growth and stress responses. Furthermore, we used WaferGen Genotyping to genotype 53 randomly chosen SNPs in 95 additional individuals from 8 sheep breeds across 3 genomic regions related to reproduction to validate the selection of these regions.

The most significant phenotypic difference between Mongolian sheep and the 2 subspecies was related to reproduction. In the recent large scale selection signature studies on sheep, reproduction has been identified as one of the selected targets and candidate genes associated with reproduction are detected in selection regions [2, 26, 36]. Kijas (2012) reported 31 genome regions with extreme differentiation among 74 world-wide sheep breeds by computing the globe *F*_ST_ at all SNP in the genome, which included candidate genes related to coat pigmentation, skeletal morphology, body size, growth, and reproduction [2]. Fariello (2014) used a new approach FLK/hapFLK to re-scan the sheep genome for selection based on the Sheep HapMap dataset among 7 broad geographical groups and also identified 71 new selection signatures, with candidate genes related to coloration, morphology or production traits [26]. The candidate gene related to reproduction *TSHR* were both detected in the above two studies and identified as ancestral signatures of selection in the latter one. *TSHR* and other genes such as *HOXA10*, *PRL* and *HMGCR* were also located in the selection regions identified in this research. Besides, we identified genes containing SNPs causing missense mutations or stop gained/loss variants and genes with SNPs located in promoter regions in Mongolian sheep but not in Small-tailed Han sheep or Duolang sheep and vice-versa. The GO term enrichments and KEGG analysis both showed evidences for selection in reproduction and provided important additional candidate genes and pathways associated with reproduction such as genes related to single fertilization, spermatogenesis, response to light stimulus and circadian rhythm and the pathway “Progesterone-mediated oocyte maturation”. For the past several decades, there has been increasing interest in the identification and utilization of major genes for prolificacy in sheep. Genes with mutations that increase the ovulation rate (*BMPR-1B*, *BMP15* and *GDF9*) and related to seasonal reproduction (*MTNR1A*) have been discovered in sheep [37–44]. However, in this study, we found that only *GDF9* showed evidence of selective pressure in Duolang sheep (Z(*H*_P_)_D_ = -4.103). The other genes appeared to not be under selection in Small-tailed Han sheep, Mongolian sheep or Duolang sheep. We checked the *H*_P_ and *F*_ST_ within genes *BMPR1B* and *MTNR1A* (the number of SNPs within *BMP15* and *GDF9* is less than 10) and the results showed the same conclusion (Additional file 25: Table S18). In contrast, according to recent research, the *BMPR1B* gene showed strong evidence of selection in highly prolific breeds, Hu sheep and Large-tailed Han sheep [3].

Besides reproductive performance, we also paid attention to the adaptation of sheep to different environments. The subspecies of Mongolian sheep have adapted to various ecoregions during the domestication process. Correspondingly, we detected several genes located in the selection regions or containing unique function-altering mutations with potential relationship with adaption such as the genes in Table 1. In the precious research, the selection near the *TRPM8* gene was reported to be related to adaptation to cold climate [26] and this gene also showed selection evidence in this study. The adaption process is complex and the potential role of many selection signatures in the adaptation of sheep breeds remains unclear. In the meantime selection response for adaptation and welfare traits may be expected to continue [2]. The findings in this research confirmed the existence of selection during the adaption of Chinese short fat-tailed sheep and provided a large number of variants to be further investigated.

*F*_ST_ is widely used in the selection signature detection of the whole genome of the domestic animals [2, 11, 19, 36, 45]. However, this approach was reported to have potential limitations: first, when applied to hierarchically structured data sets, *F*_ST_ analysis may lead to a large proportion of false positives and false negatives; second, the heterogeneity of effective population size among breeds implies that some breeds are more prone to contribute large locus-specific *F*_ST_ values than others [26, 46, 47]. In view of these limitations, a new strategy to evaluate the haplotype differentiation between populations was proposed to increase the detection power of selective sweeps and also enable to detect soft or incomplete sweeps, FLK/hapFLK [26]. In this study, we performed pooled whole genome resequencing and used three approaches to detect the selection signature on the Chinese short fat-tailed sheep genome, unique function-altering mutations, the pooled heterozygosity (*H*_P_) and the average *F*_ST_ between breeds. We expect the combination of these three approaches could breakthrough the limitation of the single method to a certain extent. The detection results of the three approaches all confirmed that reproduction is important selection target of Chinese short fat-tailed sheep.

Chr. X differs from autosomes in several aspects of population genetics, including reductions in effective population size and recombination rate. Due to genetic drift, these differences on chr. X are expected to cause a greater reduction in the level of genetic variation and increased genetic differentiation among different sheep breeds compared to the autosomes [11]. On chr. X of Chinese short fat-tailed sheep, a remarkably homozygous region (43–78 Mb) was observed in which the number of SNPs and indels was significantly less than other regions (Fig. 2).

Large-tailed Han sheep has a long history, and its origins might lie in the fat-tailed sheep in Ancient Central Asia and West Asia, which were imported to China via the Silk Road [5]. Due to the high reproductive performance of Large-tailed Han sheep, we compared it to 7 breeds of Chinese short fat-tailed sheep in this study. The results indicated that the haplotype frequencies of 3 tested gene regions related to reproduction were not significantly different between Large-tailed Han sheep and Small-tailed Han sheep. We speculate that Large-tailed Han sheep and Small-tailed Han sheep might have been affected by similar selection during the long period of microevolution.

With the advance of high-throughput sequencing technologies, the detection of variants in domesticated animals on a large scale offers great opportunities to study genome evolution in response to phenotypic selection. The present study provided genome scan for selection in Chinese native sheep breeds and is an attempt to investigate unique evolution direction and gene resources. Full use of these resources and searching the valuable genes for sheep genetic improvement will become the targets of future research. In the meantime, more extensive range of breeds, larger population size, more in-depth sequencing, and more advanced statistical methods are the directions of our improvements in this study.

## Conclusions

In summary, this study detected large amounts of genetic variations and different genomic regions under selection in 3 Chinese short fat-tailed sheep breeds. Small-tailed Han sheep is a valued local Chinese variety, famous for its high reproduction performance and strong adaptability. Genome-wide comparison studies revealed genes with unique selective signals that are associated with reproduction and other traits of this sheep breed. Our results are a valuable resource for future studies of genotype-phenotype and for the improvement of sheep breeding.

## Methods

### Animals

We obtained genomic DNA samples from whole blood from 8 sheep populations, with 20 female individuals from each population. Three breeds were used for resequencing: Mongolian sheep from Siziwang Banner in Inner Mongolia province, Small-tailed Han sheep from Jiaxiang in Shandong province, and Duolang sheep from Maigaiti in Xinjiang province. The environment, body size and reproductive performance of these three breeds were shown in Table 2. These three breeds came from different ecoregions distributed over a wide range of geographical distance with different climate and feeding conditions. WaferGen genotyping was performed on all 8 breeds. Except the three breeds used for resequencing, the other five breeds were: Luzhong Shandi sheep from Pingyin in Shandong province, Wadi sheep from Zhanhua in Shandong province, Sishui Fur sheep from Sishui in Shandong province, Large-tailed Han sheep from Linqing in Shandong province and Hu sheep from Huzhou in Zhejiang province.

**Table 2:** Comparison of the environment, body size and productive performance of the breeds used for resequencing

	Mongolian sheep	Small-tailed Han sheep	Duolang sheep
Ecoregion	Mongolian-Manchurian grassland	Huang He Plain mixed forests	Tarim Basin deciduous forests and steppe
Coordinates	41°10′–43°22′N 110°20′–113°E	35°11′–35°38′N 116°06′–116°27′E	38°25′–39°22′N 77°28′–79°05′E
Climate	Plateau sub temperate continental monsoon	Warm temperate monsoon	Temperate continental dry
Feeding conditions	Grazing	Drylot	Drylot
Male adult weight (kg)	51.3–71.1	78.2–129.6	80.6–112
Female adult weight (kg)	44.4–55.2	56–72.8	61.6–86.6
Estrous characteristics	Seasonal	Annual	Annual
Lambing rate (%)	103	267.1	250

The data shown were collected from Animal genetic resources in China: Sheep and Goats

### DNA extraction and sequencing

DNA was extracted from EDTA-preserved blood using a QIAamp DNA Blood Mini Kit (Qiagen). We performed pooled whole-genome resequencing of three sheep breeds. We pooled DNA from 15 individuals of each breed into one pool before library construction (six paired-end sequencing libraries with an insert size of 200–500 bp, two for each breed) and sequencing of 100-bp paired-end reads with a HiSeq2000 instrument (Illumina, USA). The raw sequence reads were filtered by removing the index sequences and low-quality paired reads. Specifically, we filtered sequences in which the single-ended N content exceeded 10 % of the length of the entire read or the single-ended number of bases with less than 5X depth exceeded 50 % of the entire read. Clean reads were mapped to the *Ovis aries* (sheep) genome (USUC oar_ref_Oar_v3.1; http://www.livestockgenomics.csiro.au/sheep) using the Burrows-Wheeler Alignment tool [48]. Duplicate reads were removed.

### SNP identification and annotation

SNPs are small differences but have a great impact on variation in genomes and biological traits [49]. Therefore, we investigated SNP annotations in detail and paid special attention to those in genic regions. To detect genomic regions under selection in Chinese short fat-tailed sheep, we identified SNPs from the 3 pools of resequenced breeds relative to the sheep reference genome, respectively, using SAMTools. Firstly SAMTools collects summary information from the input BAM files (Binary Alignment/Map) and computes the likelihood of data given each possible genotype and stores the likelihoods in the BCF (Binary Variant Call Format). Secondly Bcftools applies the prior and does the SNP calling and converts BCF to VCF (Variant Call Format) which can be used in the following analysis [50]. Additional filters were applied as follows: minimum read depth = 5, minimum read depth for SNP identification = 2, and VarQuality ≥ 30. After SNP identification, we annotated and predicted the effects of the SNPs using SnpEff toolbox [51] and furtherly predicted whether the amino acid substitutions affect protein function using SIFT and Provean [52].

### Selection analysis

Two approaches were used to search the sheep genome for regions that may have been affected by selection during the migration of Chinese short fat-tailed sheep. First, we calculated the average pooled heterozygosity (*H*_P_) in 200-kb windows sliding 100 kb at a time, following the formula described in Rubin et al. [9, 11]. We Z-transformed the distribution of *H*_P_ and extracted putatively selected windows at the extreme ends of the distribution by applying a Z(*H*_P_) < −4 cut-off. We divided the protein-coding genes in the putative regions of selection that met this cut-off of Small-tailed Han sheep into 3 groups: Z(*H*_P_)_S_ < -4, Z(*H*_P_)_M_ < -4 and Z(*H*_P_)_D_ < -4. Genes belonging to one (2 or 3) of the 3 groups were considered under selection in the corresponding breed(s). Second, we calculated *F*_ST_ values between any two breeds for individual SNPs [53, 54]. We averaged *F*_ST_ values across 200-kb windows, sliding 100 kb at a time, and Z-transformed the distribution. Putative selection targets were extracted from the extreme ends of the distribution by applying a Z(*F*_ST_) > 4 cut-off.

Due to the single-window pass cut-off Z(*H*_P_) < −4 and the no-windows pass Z(*F*_ST_) > 4 on chr. X, we performed extraction by applying Z(*H*_P_) < −3 or Z(*F*_ST_) > 3 for this chromosome.

### Gene ontology functional enrichment and KEGG analysis

Ensembl gene annotations were used to identify protein-coding genes located in target regions [55] (extending 100 kb up- and downstream). All of these genes were classified into the categories of molecular function in the GO database using the GOstat program (*P*-Value Cutoff: 0.1, GO-Cluster Cutoff: -1 and Correct-Method: Benjamini) [56] and were mapped to the KEGG database using DAVID Bioinformatics Resources [27, 57].

### Genotyping validation

We used WaferGen genotyping, targeting 53 SNPs located in regions showing a high level of homozygosity or population differentiation. A total of 95 sheep, representing 8 different breeds, were genotyped using standard protocols provided by the manufacturer (WaferGen, USA). Haplotypes were phased using PHASE software [58].

### Multiple sequence alignment and conserved domain analysis

DNAman software was used to perform multiple sequence alignments. Conserved domains in TSHR were detected using the NCBI bioinformatics tools [59].

## Additional files

Additional file 1: Table S1. Numbers and distribution of SNPs in the resequenced sheep breeds. (DOC 34 kb)
Additional file 2: Table S2. Numbers and distribution of indels in the resequenced sheep breeds. (DOC 34 kb)
Additional file 3: Figure S1. The number of SNPs detected from the three Chinese short fat-tailed sheep breeds. The numbers of common SNPs between Mongolian sheep and Small-tailed Han sheep, between Mongolian sheep and Duolang sheep, between Small-tailed Han sheep and Duolang sheep and across the three breeds are 6,292,070/ 6,205,458/ 6,255,570 and 4,381,068, respectively. The numbers of unique SNPs belong to the three breeds are 2,326,015/ 2,530,094/ 2,573,624. (DOC 115 kb)
Additional file 4: Table S3. Ninety-eight percent of SNPs validated using WaferGen technology. (DOC 32 kb)
Additional file 5: Table S4. Numbers and distribution of coding region SNPs and indels in the resequenced sheep breeds. (DOC 32 kb)
Additional file 6: Table S5. Enriched GO terms among genes containing missense SNPs or stop gained/loss variants in Mongolian sheep but not in Small-tailed Han sheep or Duolang sheep. (DOC 673 kb)
Additional file 7: Table S6. Enriched GO terms among genes containing missense SNPs or stop gained/loss variants in both Small-tailed Han sheep and Duolang sheep, but not in Mongolian sheep. (DOC 159 kb)
Additional file 8: Figure S2. Biological processes and KEGG pathway enrichment in genes containing missense SNPs or stop gained/loss variants. A, Biological processes enrichment in gene containing missense SNPs or stop gained/loss variants in Mongolian sheep but not in Small-tailed Han sheep or Duolang sheep. B, Biological processes enrichment in gene containing missense SNPs or stop gained/loss variants in both Small-tailed Han sheep and Duolang sheep, but not in Mongolian sheep. C, KEGG pathway enrichment in genes containing missense SNPs or stop gained/loss variants in Mongolian sheep but not in Small-tailed Han sheep or Duolang sheep. D, KEGG pathway enrichment in genes containing missense SNPs or stop gained/loss variants in both Small-tailed Han sheep and Duolang sheep, but not in Mongolian sheep. Biological processes and KEGG pathway related to reproduction are labeled in red. (DOC 955 kb)
Additional file 9: Table S7. Enriched KEGG pathways among genes containing missense SNPs or stop gained/loss variants in Mongolian sheep but not in Small-tailed Han sheep or Duolang sheep. (DOC 44 kb)
Additional file 10: Table S8. Enriched KEGG pathways among genes containing missense SNPs or stop gained/loss variants in both Small-tailed Han sheep and Duolang sheep, but not in Mongolian sheep. (DOC 34 kb)
Additional file 11: Table S9. Enriched GO terms among genes containing missense SNPs in promoter regions in Mongolian sheep but not in Small-tailed Han sheep or Duolang sheep. (DOC 570 kb)
Additional file 12: Table S10. Enriched KEGG pathways among genes containing missense SNPs in promoter regions in Mongolian sheep but not in Small-tailed Han sheep or Duolang sheep. (DOC 50 kb)
Additional file 13: Table S11. Enriched GO terms among genes containing missense SNPs in promoter regions in both Small-tailed Han sheep and Duolang sheep, but not in Mongolian sheep. (DOC 71 kb)
Additional file 14: Figure S3. Biological processes and KEGG pathway enrichment in genes containing SNPs in promoter regions. A, Biological processes enrichment in genes containing SNPs in promoter regions in Mongolian sheep but not in Small-tailed Han sheep or Duolang sheep. B, Biological processes enrichment in genes containing SNPs in promoter regions in both Small-tailed Han sheep and Duolang sheep, but not in Mongolian sheep. C, KEGG pathway enrichment in genes containing SNPs in promoter regions in Mongolian sheep but not in Small-tailed Han sheep or Duolang sheep. Biological processes related to reproduction are labeled in red. (DOC 1117 kb)
Additional file 15: Table S12. The protein coding SNPs detected by SIFT and Provean analysis. (DOC 29 kb)
Additional file 16: Figure S4. The distributions of heterozygosity (*H*_P_) and the average fixation index (*F*_ST_) between any two of the 3 resequenced breeds for autosomal 200-kb windows. (DOC 107 kb)
Additional file 17: Figure S5. The distributions of heterozygosity (*H*_P_) and the average fixation index (*F*_ST_) between any two of the 3 resequenced breeds and their Z transformations for 200-kb windows on chr. X. (DOC 124 kb)
Additional file 18: Table S13. Genomic regions under selection in Small-tailed Han sheep. (DOC 126 kb)
Additional file 19: Table S14. Genomic regions under selection in Duolang sheep. (DOC 127 kb)
Additional file 20: Table S15. Enriched GO terms among genes located in the selection regions with Z(*F*_ST_)_M-S_ > 4 and Z(*F*_ST_)_M-D_ > 4. (DOC 166 kb)
Additional file 21: Table S16. Enriched GO terms among genes located in the selection regions with Z(*F*_ST_)_M-S_ > 4. (DOC 145 kb)
Additional file 22: Table S17. Enriched GO terms among genes located in the selection regions with Z(*F*_ST_)_M-D_ > 4. (DOC 145 kb)
Additional file 23: Figure S6. Pooled heterozygosity of Duolang sheep, *H*_P_ (in blue), and average fixation index between Mongolian sheep and Duolang sheep, *F*_ST_ (in red), plotted for 200-kb windows spanning the region harboring *TSHR* (in shadow) on chr. 7. (DOC 308 kb)
Additional file 24: Figure S7. Haplotype diversities of genomic regions harboring *PRL* and *HMGCR*. A and B indicate the *H*_P_ of the 3 resequenced breeds, plotted for 200-kb windows spanning the region harboring *PRL* and *HMGCR*. C and D indicate the genetic variation in the region 24.22–24.26 Mb on chr. 20 across *PRL* and *LOC443319* and the region 6.53–6.57 Mb on chr. 7 across *HMGCR*. Individual sheep (95 from 8 breeds) were genotyped using WaferGen genotyping. Dashed horizontal lines separate the 8 breeds. At the bottom of the figure, short tick marks represent individual SNPs. Long tick marks indicate the position in Mb. Red color: homozygous A-allele; green color: heterozygous; purple color: homozygous a-allele, yellow color: missing genotype call. E and F indicate the haplotype frequencies of the 8 sheep breeds at the 2 genomic regions. (DOC 214 kb)
Additional file 25: Table S18. The *H*_P_ and *F*_ST_ within *BMPR1B* and *MTNR1A*. (DOC 32 kb)

## Abbreviations

7tm_1: 7-transmembrane receptor (rhodopsin family)
7TM_GPCR_Srx: Serpentine type seven-transmembrane G-protein-coupled receptor class chemoreceptor Srx
*ADCY5*: Adenylate cyclase 5
*ALOX12*: Arachidonate 12-lipoxygenase
*AR*: Androgen receptor
*ARFRP1*: ADP-ribosylation factor related protein 1
BAM: Binary Alignment/Map
BCF: Binary Variant Call Format
*BMP15*: Bone morphogenetic protein 15
*BMPR-1B*: Bone morphogenetic protein receptor, type 1B
bp: Base pairs
*C20orf195*: Chromosome 20 open reading frame 195
*CCNB2*: Cyclin B2
*CDC25B*: Cell division cycle 25B
CDS: Sequences encoding amino acids in proteins
Chr.: Chromosome
DNA: Deoxyribonucleic acid
EDTA: Ethylene diamine tetraacetic acid
*EEF1A2*: Eukaryotic translation elongation factor 1 alpha 2
*F*_ST_: Fixation index
*F*_ST_-chrA: The average *F*_ST_ on autosomes
*F*_ST_-chrX: The average *F*_ST_ on chromosome X
*GDF9*: Growth differentiation factor 9
*GJB6*: Gap junction protein beta 6
*GMEB2*: Glucocorticoid modulatory element binding protein 2
*GNAI2*: Guanine nucleotide binding protein (G protein), alpha inhibiting 2
GO: Gene ontology
*HMGCR*: 3-hydroxy-3-methylglutaryl-CoA reductase
*HOXA10*: Homeobox A10
*HOXA2*: Homeobox A2
*H*_P_: Heterozygosity
*H*_P_-chrA: The average *H*_P_ on autosomes
*H*_P_-chrX: The average *H*_P_ on chromosome X
indel: Insertion/deletion
KEGG: Kyoto encyclopedia of genes and genomes
*LHFPL5*: Lipoma HMGIC fusion partner-like 5
*LOC443319*: Placental lactogen
*MAD2L1*: MAD2 mitotic arrest deficient-like 1
*MC1R*: Melanocortin 1 receptor
*MSTN*: Myostatin
*MTNR1A*: Melatonin receptor 1A
NCBI: The National Center for Biotechnology Information
*OTX1*: Orthodenticle homolog 1
*OXSR1*: Oxidative Stress Responsive 1
*PIK3R5*: Phosphoinositide-3-kinase, regulatory subunit 5
*POLH*: Polymerase (DNA) Eta
PRL: Prolactin
*PTK6*: Protein tyrosine kinase 6
*RPS6KA3*: Ribosomal protein S6 kinase, 90 kDa, polypeptide 3
*RTEL1*: Regulator of telomere elongation helicase 1
*RXFP2*: Relaxin/insulin-like family peptide receptor 2
SAM: Sequence Alignment/Map
SNP: Single-nucleotide polymorphism
*SOD1*: Superoxide dismutase 1, soluble
*SRMS*: Src-related kinase lacking C-terminal regulatory tyrosine and N-terminal myristylation sites
*STMN3*: Stathmin-like 3
*TGM7*: Transglutaminase 7
*TRPM8*: Transient Receptor Potential Cation Channel Subfamily M Member 8
TSHR: Thyroid stimulating hormone receptor
*UBE2N*: Ubiquitin Conjugating Enzyme E2 N
VCF: Variant Call Format
Z(*F*_ST_): The Z transformation of *F*_ST_
Z(*F*_ST_)_M-S_: The Z transformation of *F*_ST_ between Mongolian sheep and Small-tailed Han sheep
Z(*H*_P_): The Z transformation of *H*_P_
Z(*H*_P_)_D_: The Z transformation of *H*_P_ in Duolang sheep
Z(*H*_P_)_M_: The Z transformation of *H*_P_ in Mongolian sheep
Z(*H*_P_)_S_: The Z transformation of *H*_P_ in Small-tailed Han sheep
*ZBTB46*: Zinc finger and BTB domain containing 46
*ZGPAT*: Zinc finger, CCCH-type with G patch domain
σA: The standard deviation of *H*_P_ or *F*_ST_ on autosomes
σX: The standard deviation of *H*_P_ or *F*_ST_ on chromosome X
