# Supplementary material for: Genome-wide analysis reveals signatures of selection for important traits in domestic sheep from different ecoregions
Source: BMC Genomics. 2016 Nov 3;17:863. doi: 10.1186/s12864-016-3212-2 (PMC5094087; doi:10.1186/s12864-016-3212-2)
Supplement: Additional file 1: Table S1. — Numbers and distribution of SNPs in the resequenced sheep breeds. (DOC 34 kb) [file 12864_2016_3212_MOESM1_ESM.doc]

**Additional file 1: Table S1.** Numbers and distribution of SNPs in the resequenced sheep breeds

| Sample | Mongolian sheep | Small-tailed Han sheep | Duolang sheep |
| --- | --- | --- | --- |
| calling SNPs | 10581081 | 10733216 | 10741007 |
| Excluded the dbSNP | 10492587 | 10646554 | 10653584 |
| CDS Site | 1027 | 1034 | 1069 |
| Splice Site | 61 | 64 | 62 |
| 5' UTR | 60 | 76 | 65 |
| 3' UTR | 370 | 369 | 360 |
| Intron | 78663 | 79441 | 80601 |
| Exon | 1464 | 1477 | 1495 |
| Intergenic | 10412400 | 10565573 | 10571427 |
