# Supplementary material for: Genome-wide analysis reveals signatures of selection for important traits in domestic sheep from different ecoregions
Source: BMC Genomics. 2016 Nov 3;17:863. doi: 10.1186/s12864-016-3212-2 (PMC5094087; doi:10.1186/s12864-016-3212-2)
Supplement: Additional file 2: Table S2. — Numbers and distribution of indels in the resequenced sheep breeds. (DOC 34 kb) [file 12864_2016_3212_MOESM2_ESM.doc]

**Additional file 2: Table S2.** Numbers and distribution of indels in the resequenced sheep breeds

| Sample | Mongolian sheep | Small-tailed Han sheep | Duolang sheep |
| --- | --- | --- | --- |
| Total indels | 1083166 | 910403 | 918562 |
| CDS | 91 | 36 | 42 |
| 5' UTR | 11 | 9 | 8 |
| 3' UTR | 59 | 59 | 60 |
| Splice Site | 39 | 17 | 22 |
| Intron | 8847 | 7199 | 7438 |
| Exon | 136 | 98 | 96 |
| Intergenic | 1074144 | 903089 | 911006 |
