# Supplementary material for: Genome-wide analysis reveals signatures of selection for important traits in domestic sheep from different ecoregions
Source: BMC Genomics. 2016 Nov 3;17:863. doi: 10.1186/s12864-016-3212-2 (PMC5094087; doi:10.1186/s12864-016-3212-2)

**Additional file 3: Figure S1.** The number of SNPs detected from the three Chinese short fat-tailed sheep breeds. The numbers of common SNPs between Mongolian sheep and Small-tailed Han sheep, between Mongolian sheep and Duolang sheep, between Small-tailed Han sheep and Duolang sheep and across the three breeds are 6,292,070/ 6,205,458/ 6,255,570 and 4,381,068, respectively. The numbers of unique SNPs belong to the three breeds are 2,326,015/ 2,530,094/ 2,573,624.


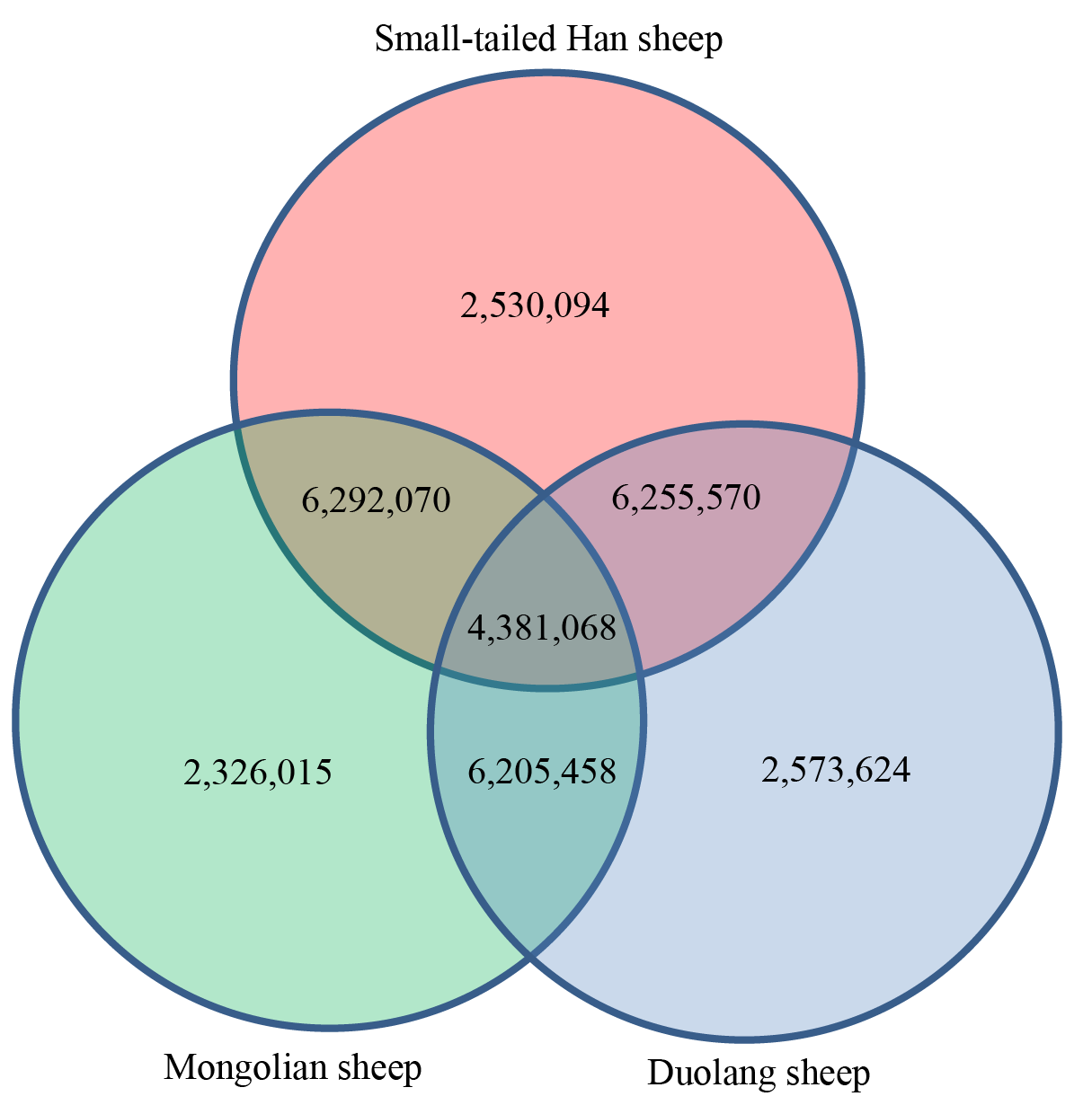

Supplement: Additional file 3: Figure S1. — The number of SNPs detected from the three Chinese short fat-tailed sheep breeds. The numbers of common SNPs between Mongolian sheep and Small-tailed Han sheep, between Mongolian sheep and Duolang sheep, between Small-tailed Han sheep and Duolang sheep and across the three breeds are 6,292,070/ 6,205,458/ 6,255,570 and 4,381,068, respectively. The numbers of unique SNPs belong to the three breeds are 2,326,015/ 2,530,094/ 2,573,624. (DOC 115 kb) [file 12864_2016_3212_MOESM3_ESM.doc]
