# Supplementary material for: Genome-wide analysis reveals signatures of selection for important traits in domestic sheep from different ecoregions
Source: BMC Genomics. 2016 Nov 3;17:863. doi: 10.1186/s12864-016-3212-2 (PMC5094087; doi:10.1186/s12864-016-3212-2)
Supplement: Additional file 4: Table S3. — Ninety-eight percent of SNPs validated using WaferGen technology. (DOC 32 kb) [file 12864_2016_3212_MOESM4_ESM.doc]

**Additional file 4: Table S3.** 98% of SNPs validated using WaferGen technology.

| Assays | Number of SNPs |
| --- | --- |
| Total number of SNP assays | 104 |
| Failed SNPs assay | 0 |
| Excluded SNPs due to low call rates | 2 |
| Erroneous base call in genome assembly | 0 |
| Total number of successful SNP assays | 102 |
| Erroneous base call in resequencing data | 2 |
| Verified SNPs | 100 |
