# Supplementary material for: Genome-wide analysis reveals signatures of selection for important traits in domestic sheep from different ecoregions
Source: BMC Genomics. 2016 Nov 3;17:863. doi: 10.1186/s12864-016-3212-2 (PMC5094087; doi:10.1186/s12864-016-3212-2)
Supplement: Additional file 5: Table S4. — Numbers and distribution of coding region SNPs and indels in the resequenced sheep breeds. (DOC 32 kb) [file 12864_2016_3212_MOESM5_ESM.doc]

**Additional file 5: Table S4.** Numbers and distribution of coding region SNPs and indels in the resequenced sheep breeds.

| Breeds | synonymous SNPs | missense SNPs | genes with missense SNPs | stop-gained SNPs | genes with stop-gained SNPs | SNP Total | indels |
| --- | --- | --- | --- | --- | --- | --- | --- |
| Small-tailed Han | 36,820 | 54,033 | 11,063 | 2,474 | 1,789 | 90,853 | 36 |
| Mongolian | 37,449 | 53,271 | 11,421 | 2,421 | 1,810 | 90,720 | 91 |
| Duolang | 21,029 | 35,775 | 7,724 | 1,786 | 1,192 | 56,804 | 42 |
