# Supplementary material for: Genome-wide analysis reveals signatures of selection for important traits in domestic sheep from different ecoregions
Source: BMC Genomics. 2016 Nov 3;17:863. doi: 10.1186/s12864-016-3212-2 (PMC5094087; doi:10.1186/s12864-016-3212-2)
Supplement: Additional file 7: Table S6. — Enriched GO terms among genes containing missense SNPs or stop gained/loss variants in both Small-tailed Han sheep and Duolang sheep, but not in Mongolian sheep. (DOC 159 kb) [file 12864_2016_3212_MOESM7_ESM.doc]

**Additional file 7: Table S6**. Enriched GO terms among genes containing missense SNPs or stop gained/loss variants in both Small-tailed Han sheep and Duolang sheep, but not in Mongolian sheep.

| GO term | Gene count | P value | genes |
| --- | --- | --- | --- |
| developmental process; | 116#3347 | 1.70197E-15 | pspn;dcn;sgpp1;arhgap24;smpd3;kcnj8;rcan3;ptch2;ntng2;btd;lbx1;ckb;spred2;sox11;tcf12;kif2a;tnfrsf8;krt5;wisp3;psen1;theg;rpe65;edil3;gna11;cand1;ptch1;tbx5;tctn1;mtl5;cidec;acsbg1;dyrk1a;nlgn1;itgb1;angpt2;cdkn1b;krt3;l1cam;kl;krt2;cdc42bpb;cdkn2aip;tmprss6;ash2l;lepr;il4;il27ra;tnfsf10;mark4;vcp;tnni3;lima1;cd5;sema6a;rabep1;rasa1;gfi1;csde1;kcnip2;chuk;gas1;gdf5;caprin2;zar1;gldn;atxn3;fga;serpine2;thbs1;sox6;fzd10;trim28;spata2;hoxa10;psme4;taf7l;pou4f2;rps27a;pou1f1;spesp1;slitrk1;cdh11;pla2g6;fut10;pard3;adam23;dnm1l;diablo;socs5;tbx21;hdac6;sh2b3;ttl;rerg;cylc2;tfap2a;shox2;stat5a;dvl1;rps6ka3;dfna5;prdm6;ripk3;nfe2l1;catsperb;spag16;tcf15;mapk8ip2;alpl;iapp;vsx2;bnip3;dbn1;col9a2;kit;dmp1 |
| anatomical structure development; | 80#2005 | 8.37657E-15 | pspn;dcn;arhgap24;kcnj8;rcan3;ptch2;ntng2;btd;lbx1;ckb;sox11;tcf12;kif2a;krt5;wisp3;rpe65;gna11;ptch1;tbx5;acsbg1;dyrk1a;nlgn1;angpt2;cdkn1b;krt3;l1cam;kl;krt2;cdc42bpb;cdkn2aip;tmprss6;ash2l;il4;il27ra;mark4;tnni3;lima1;sema6a;rasa1;gfi1;csde1;kcnip2;chuk;gas1;gdf5;caprin2;gldn;atxn3;serpine2;fga;thbs1;sox6;pou4f2;rps27a;pou1f1;slitrk1;cdh11;fut10;adam23;pard3;socs5;tbx21;sh2b3;rerg;ttl;tfap2a;shox2;stat5a;dvl1;rps6ka3;dfna5;nfe2l1;spag16;tcf15;alpl;vsx2;dbn1;col9a2;kit;dmp1 |
| multicellular organismal development; | 87#2299 | 1.98848E-14 | pspn;dcn;arhgap24;smpd3;kcnj8;ptch2;ntng2;btd;lbx1;ckb;spred2;sox11;tcf12;kif2a;krt5;theg;edil3;gna11;ptch1;tbx5;tctn1;mtl5;acsbg1;dyrk1a;nlgn1;itgb1;angpt2;cdkn1b;l1cam;kl;krt2;tmprss6;ash2l;lepr;il4;il27ra;mark4;tnni3;sema6a;rasa1;gfi1;csde1;kcnip2;gas1;gdf5;zar1;gldn;atxn3;fga;serpine2;thbs1;sox6;fzd10;hoxa10;spata2;psme4;taf7l;pou4f2;rps27a;pou1f1;spesp1;slitrk1;cdh11;fut10;adam23;dnm1l;pard3;socs5;tbx21;hdac6;sh2b3;ttl;cylc2;tfap2a;shox2;stat5a;dvl1;rps6ka3;dfna5;catsperb;tcf15;alpl;vsx2;dbn1;col9a2;kit;dmp1 |
| multicellular organismal development#system development; | 67#1605 | 9.70362E-14 | pspn;dcn;arhgap24;kcnj8;ptch2;ntng2;btd;lbx1;ckb;sox11;tcf12;kif2a;krt5;gna11;ptch1;tbx5;acsbg1;dyrk1a;nlgn1;angpt2;cdkn1b;l1cam;kl;krt2;tmprss6;ash2l;il4;il27ra;mark4;tnni3;sema6a;rasa1;gfi1;csde1;gas1;kcnip2;gldn;serpine2;fga;atxn3;thbs1;sox6;pou4f2;rps27a;pou1f1;slitrk1;cdh11;fut10;adam23;pard3;socs5;tbx21;sh2b3;ttl;tfap2a;shox2;stat5a;dvl1;rps6ka3;dfna5;tcf15;alpl;vsx2;dbn1;col9a2;kit;dmp1 |
| multicellular organismal process; | 117#3822 | 3.85143E-11 | opn3;pspn;dcn;arhgap24;smpd3;kcnj8;ptch2;ntng2;htr1d;btd;lbx1;ckb;spred2;sox11;tcf12;kif2a;tnfrsf8;krt5;mlnr;theg;rpe65;edil3;gna11;ptch1;tbx5;tctn1;glra1;mtl5;c1qb;acsbg1;dyrk1a;nlgn1;itgb1;angpt2;trpa1;cdkn1b;hcn2;lrat;l1cam;kl;krt2;tmprss6;ash2l;diaph1;kcng2;lepr;il4;hps1;il27ra;mark4;tnni3;gast;kcnmb3;cd5;sema6a;rasa1;gfi1;csde1;kcnip2;gria2;gas1;aldh9a1;gdf5;elovl4;zar1;gldn;atxn3;fga;serpine2;prodh2;thbs1;sox6;fzd10;spata2;hoxa10;psme4;cngb3;taf7l;pou4f2;rps27a;pou1f1;hprt1;spesp1;slitrk1;cdh11;fut10;pard3;adam23;dnm1l;socs5;cald1;tbx21;hdac6;sh2b3;ntsr1;ttl;cylc2;tfap2a;shox2;stat5a;dvl1;rps6ka3;dfna5;gprc5d;atp6ap2;adcy5;catsperb;tcf15;slc6a8;alpl;iapp;vsx2;dbn1;col9a2;kit;pde7b;dmp1 |
| regulation of biological process; | 161#6140 | 2.66214E-09 | rasgrp1;opn3;sox11;tcf12;tnfrsf8;psen1;dnmt3b;gna11;cand1;glra1;mtl5;znf300;homez;ptx3;ewsr1;suv420h2;cdkn1b;uhrf1;mfn2;cdc42bpb;tbc1d22a;cdkn2b;cdkn2aip;rabl3;ell2;taf5;ash2l;znf354b;kcng2;pou2af1;nr2c1;il27ra;tnfsf10;mark4;znf462;tnni3;ncoa3;xrn1;lin9;terf1;rasa1;gfi1;csde1;srebf1;rgl3;kcnip2;mknk2;btf3;mbd1;mtif3;tsc22d2;garnl3;ralgds;ncoa7;atxn3;fga;serpine2;znf653;sox6;iqgap2;taf7l;rps27a;pou1f1;sin3a;znf84;diablo;tbx21;kcnh5;hdac6;rerg;tbc1d10c;tfap2a;shox2;psd3;atp6ap2;dio2;ripk3;iqgap1;nfe2l1;tmem189;tcf15;bnip3;znf711;dbn1;kit;cnksr2;znf546;znf75a;lbx1;spred2;wisp3;rpe65;ptch1;tle6;l3mbtl3;plekhg5;arhgap20;tbx5;cidec;c1qb;nlgn1;rap1gap;atf4;ell;tbc1d21;kl;tbl1xr1;znf358;tmprss6;il16;mynn;dnmt1;il4;rnf4;eif5b;khdrbs1;vcp;tmem101;znf606;znf263;lima1;mad2l1;znf696;cd5;nsd1;gas1;aldh9a1;gdf5;caprin2;rtf1;nfe2l2;chek1;zbtb10;ighmbp2;tceal7;cdc25b;znf576;trim28;hoxa10;pou4f2;fhit;hprt1;trmt6;socs5;ttl;lmo3;ccdc59;fmnl2;stat5a;adamdec1;taf2;zbtb9;prdm6;zbtb7a;mapk8ip2;dnaja2;slc6a8;iapp;vsx2;srebf2;znhit3 |
| multicellular organismal development#system development#organ development; | 47#1141 | 5.40506E-09 | gas1;pspn;dcn;arhgap24;kcnj8;ptch2;btd;lbx1;ckb;fga;tcf12;sox6;krt5;gna11;ptch1;pou4f2;tbx5;rps27a;pou1f1;angpt2;cdh11;fut10;cdkn1b;socs5;sh2b3;kl;krt2;tfap2a;shox2;tmprss6;ash2l;stat5a;dvl1;rps6ka3;dfna5;il4;tcf15;alpl;tnni3;vsx2;sema6a;col9a2;gfi1;csde1;rasa1;kit;dmp1 |
| biological regulation; | 169#6731 | 3.08591E-08 | rasgrp1;opn3;ckb;sox11;tcf12;tnfrsf8;psen1;dnmt3b;gna11;cand1;glra1;mtl5;znf300;acsbg1;homez;ptx3;ewsr1;suv420h2;cdkn1b;uhrf1;mfn2;cdc42bpb;tbc1d22a;cdkn2b;cdkn2aip;rabl3;gnai2;ell2;taf5;ash2l;znf354b;kcng2;pou2af1;nr2c1;il27ra;tnfsf10;mark4;znf462;tnni3;ncoa3;kcnmb3;xrn1;lin9;terf1;rasa1;gfi1;csde1;srebf1;rgl3;kcnip2;mknk2;btf3;mbd1;mtif3;tsc22d2;garnl3;ralgds;ncoa7;atxn3;fga;serpine2;znf653;sox6;iqgap2;taf7l;rps27a;pou1f1;sin3a;znf84;pard3;diablo;tbx21;kcnh5;hdac6;rerg;tbc1d10c;tfap2a;shox2;psd3;atp6ap2;dio2;ripk3;iqgap1;nfe2l1;tmem189;tcf15;bnip3;znf711;dbn1;kit;kcnj8;cnksr2;znf546;znf75a;lbx1;spred2;wisp3;rpe65;ptch1;tle6;l3mbtl3;tbx5;plekhg5;arhgap20;cidec;c1qb;nlgn1;rap1gap;atf4;hcn2;ell;tbc1d21;kl;tbl1xr1;znf358;tmprss6;il16;mynn;dnmt1;il4;rnf4;eif5b;khdrbs1;vcp;tmem101;znf606;znf263;lima1;mad2l1;znf696;cd5;nsd1;gas1;aldh9a1;gdf5;caprin2;rtf1;nfe2l2;chek1;zbtb10;ighmbp2;tceal7;cdc25b;thbs1;znf576;trim28;hoxa10;pou4f2;fhit;hprt1;trmt6;socs5;ttl;lmo3;ccdc59;fmnl2;stat5a;adamdec1;taf2;zbtb9;prdm6;zbtb7a;mapk8ip2;dnaja2;slc6a8;iapp;vsx2;srebf2;znhit3 |
| cellular process; | 391#19591 | 4.29579E-08 | rasgrp1;ptch2;map3k12;htr1d;ckb;timm50;ddx47;ndufa1;mvp;gria4;psma5;kif2a;gpam;psen1;cdca3;il1rl2;dnmt3b;gmds;cand1;prokr2;kin;znf300;acsbg1;epha7;ipo7;tpd52l1;ptx3;angpt2;hbs1l;slc8a3;rpusd3;ptgis;uhrf1;h3f3a;imp3;fcgr1a;eltd1;krt2;cdc42bpb;hal;cdkn2b;rabl3;ell2;hsd3b1;taf5;ash2l;pou2af1;il27ra;tnfsf10;znf462;ncoa3;depdc1b;fap;lin9;rabep1;terf1;rrs1;rasa1;gfi1;mctp1;kcnip2;mknk2;slc7a2;endog;mbd1;mlkl;rfc3;exosc9;tsc22d2;garnl3;rpusd4;ralgds;ncoa7;gldn;znf653;serpine2;ints4;spata2;fpgs;hace1;iqgap2;cngb3;gars;atg4b;galk2;rps27a;pou1f1;slitrk1;pard3;dnm1l;adam23;cdh22;flrt3;kcnh5;dhx35;rerg;ptbp2;pde2a;sf3a3;gpr39;cct6a;rps6ka3;psd3;plcb3;exoc5;ripk3;tomm7;recql;tcf15;ccnb2;znf711;gstk1;fbxl7;dbn1;pde7b;atp5j;rcan3;pigf;cnksr2;znf546;znf75a;spred2;adamts6;wisp3;ptch1;pcsk5;arhgap20;fbxl22;ca8;nlgn1;clec7a;atf4;fkbp11;mrrf;krt3;znf358;tmprss6;il16;diaph1;lepr;gc;hps1;sbk1;sult2b1;ctsl2;coq6;znf606;znf263;gast;mad2l1;nploc4;sema6a;padi6;tgm6;gas1;prc1;gdf5;gpr68;yod1;kif19;ighmbp2;elovl4;fmo2;wdsub1;znf576;vti1a;psmb8;ppil2;hoxa10;itk;pfdn6;etnk1;adam11;pdk3;fhit;hprt1;fut10;clta;zmpste24;cald1;darc;ttl;cylc2;fmnl2;adamdec1;cd79b;dfna5;crisp1;fbxo24;fbxo32;prdm6;dpp4;mapk8ip2;dnaja2;slc6a8;ddx46;opn3;sgpp1;smpd3;ntng2;btd;sox11;stx3;entpd4;upp2;tcf12;tnfrsf8;mlnr;il4i1;gna11;glra1;g6pd;mtl5;dyrk1a;homez;itgb1;ewsr1;suv420h2;actr10;cdkn1b;dok1;rad52;trip10;mfn2;bop1;map3k1;tbc1d22a;zc3hc1;cdkn2aip;gnai2;znf354b;frs3;nr2c1;mark4;tpk1;pmvk;cct2;tnni3;kcnmb3;pex26;xrn1;csde1;ppfibp1;arhgap23;rgl3;srebf1;gria2;cstf3;btf3;mtif3;kif20a;pmm1;aldob;gpr119;atxn3;fga;prodh2;sox6;dnase1l1;igfals;psme4;taf7l;sin3a;srr;pla2g6;mis12;znf84;mthfd1;diablo;tbx21;hdac6;tbc1d10c;dpp3;tfap2a;shox2;fkbp9;rfc5;gprc5d;ust;atp6ap2;dio2;stk31;iqgap1;nfe2l1;catsperb;tmem189;ints10;bnip3;nckipsd;kit;dmp1;cdh5;arhgap24;cd8a;slc29a1;lbx1;bcan;eif2c3;brcc3;ppil4;rpe65;theg;hs3st3b1;snx25;edil3;tle6;l3mbtl3;tbx5;plekhg5;alas1;cidec;c1qb;rap1gap;ubqlnl;hcn2;lrat;pex11b;ell;l1cam;tbc1d21;tbl1xr1;nt5c1a;plcl1;mynn;dnmt1;il4;pcmtd1;rnf4;eif5b;aste1;cdca8;pdp2;khdrbs1;uxt;vcp;tmem101;lima1;znf696;rsad1;cd5;nsd1;ppp2r5e;chuk;aldh9a1;caprin2;rtf1;slc6a14;nfe2l2;chek1;zbtb10;lrrc47;tceal7;wasf1;cdc25b;thbs1;fzd10;trim28;arcn1;tcp1;ddr1;pou4f2;tpst2;gpr83;cdh11;itga2b;trmt6;socs5;sh2b3;myef2;ntsr1;sytl1;lmo3;ccdc59;stat5a;mat2a;taf2;dvl1;zbtb9;adcy5;zbtb7a;mrpl19;spag16;kif2c;cdkl5;oxr1;iapp;vsx2;rbm25;rad51ap1;srebf2;znhit3 |
| anatomical structure morphogenesis; | 42#1047 | 1.67894E-07 | chuk;gas1;gdf5;pspn;dcn;arhgap24;caprin2;kcnj8;rcan3;ntng2;lbx1;fga;wisp3;rpe65;ptch1;pou4f2;tbx5;rps27a;pou1f1;slitrk1;angpt2;cdkn1b;pard3;socs5;krt3;ttl;rerg;krt2;cdc42bpb;cdkn2aip;tmprss6;stat5a;dvl1;nfe2l1;spag16;tnni3;lima1;vsx2;sema6a;dbn1;gfi1;rasa1 |
| cellular developmental process; | 61#1810 | 3.27162E-07 | kcnip2;gas1;gdf5;sgpp1;arhgap24;ntng2;lbx1;gldn;atxn3;serpine2;kif2a;tnfrsf8;trim28;psen1;spata2;theg;rpe65;psme4;gna11;cand1;taf7l;pou4f2;tbx5;mtl5;cidec;rps27a;nlgn1;slitrk1;angpt2;pla2g6;cdkn1b;pard3;dnm1l;diablo;socs5;krt3;sh2b3;ttl;l1cam;cylc2;krt2;stat5a;dfna5;il4;prdm6;ripk3;tnfsf10;catsperb;mark4;mapk8ip2;vcp;tnni3;iapp;vsx2;bnip3;cd5;sema6a;dbn1;rabep1;gfi1;rasa1 |
| cell differentiation; | 61#1810 | 3.27162E-07 | kcnip2;gas1;gdf5;sgpp1;arhgap24;ntng2;lbx1;gldn;atxn3;serpine2;kif2a;tnfrsf8;trim28;psen1;spata2;theg;rpe65;psme4;gna11;cand1;taf7l;pou4f2;tbx5;mtl5;cidec;rps27a;nlgn1;slitrk1;angpt2;pla2g6;cdkn1b;pard3;dnm1l;diablo;socs5;krt3;sh2b3;ttl;l1cam;cylc2;krt2;stat5a;dfna5;il4;prdm6;ripk3;tnfsf10;catsperb;mark4;mapk8ip2;vcp;tnni3;iapp;vsx2;bnip3;cd5;sema6a;dbn1;rabep1;gfi1;rasa1 |
| cellular process#regulation of cellular process; | 144#5704 | 5.4456E-07 | rasgrp1;sox11;tcf12;tnfrsf8;psen1;gna11;cand1;mtl5;znf300;homez;ptx3;ewsr1;suv420h2;cdkn1b;uhrf1;mfn2;cdc42bpb;tbc1d22a;cdkn2b;cdkn2aip;rabl3;ell2;taf5;ash2l;znf354b;pou2af1;nr2c1;tnfsf10;mark4;znf462;ncoa3;xrn1;lin9;terf1;rasa1;gfi1;csde1;srebf1;rgl3;mknk2;btf3;mbd1;mtif3;tsc22d2;garnl3;ralgds;ncoa7;atxn3;fga;serpine2;znf653;sox6;iqgap2;taf7l;rps27a;pou1f1;sin3a;znf84;diablo;tbx21;kcnh5;hdac6;rerg;tbc1d10c;tfap2a;shox2;psd3;atp6ap2;dio2;ripk3;iqgap1;nfe2l1;tcf15;bnip3;znf711;kit;cnksr2;znf546;znf75a;lbx1;spred2;wisp3;ptch1;tle6;l3mbtl3;plekhg5;arhgap20;tbx5;cidec;nlgn1;rap1gap;atf4;ell;tbc1d21;tbl1xr1;znf358;mynn;dnmt1;il4;rnf4;eif5b;khdrbs1;vcp;tmem101;znf606;znf263;lima1;mad2l1;znf696;nsd1;cd5;gas1;gdf5;caprin2;rtf1;nfe2l2;chek1;zbtb10;ighmbp2;tceal7;cdc25b;znf576;trim28;hoxa10;pou4f2;fhit;hprt1;trmt6;socs5;lmo3;fmnl2;ccdc59;stat5a;adamdec1;taf2;zbtb9;prdm6;zbtb7a;mapk8ip2;dnaja2;iapp;vsx2;srebf2;znhit3 |
| mitotic cell cycle; | 19#326 | 6.33815E-07 | gas1;prc1;chek1;zc3hc1;cdkn2b;stat5a;cdc25b;cdca8;cdca3;khdrbs1;kif2c;dnaja2;ccnb2;mad2l1;tpd52l1;mis12;cdkn1b;gfi1;terf1 |
| nervous system development; | 31#716 | 1.64603E-06 | kcnip2;pspn;ttl;l1cam;krt2;ntng2;btd;shox2;lbx1;ckb;sox11;rps6ka3;gldn;atxn3;serpine2;kif2a;thbs1;mark4;pou4f2;acsbg1;rps27a;dyrk1a;pou1f1;vsx2;nlgn1;slitrk1;fut10;sema6a;dbn1;pard3;adam23 |
| transcription from RNA polymerase II promoter; | 28#640 | 6.03208E-06 | btf3;mbd1;ell;nfe2l2;tfap2a;ell2;taf5;ash2l;stat5a;taf2;tcf12;il4;dnmt1;rnf4;pou2af1;trim28;nr2c1;nfe2l1;zbtb7a;pou4f2;tcf15;pou1f1;atf4;nsd1;srebf2;gfi1;uhrf1;srebf1 |
| cellular component organization and biogenesis; | 90#3277 | 1.10189E-05 | ptch2;map3k12;ntng2;timm50;mvp;stx3;kif2a;wisp3;theg;rpe65;glra1;ipo7;nlgn1;ptx3;clec7a;suv420h2;actr10;cdkn1b;h3f3a;imp3;trip10;fcgr1a;krt3;mfn2;pex11b;bop1;cdc42bpb;tbl1xr1;cdkn2aip;tmprss6;taf5;diaph1;hps1;eif5b;mark4;khdrbs1;uxt;lima1;pex26;nsd1;nploc4;sema6a;rabep1;terf1;rrs1;rasa1;kcnip2;prc1;mtif3;caprin2;exosc9;kif19;kif20a;wasf1;ncoa7;sox6;vti1a;arcn1;pou4f2;atg4b;gars;rps27a;pou1f1;hprt1;slitrk1;mis12;fut10;clta;trmt6;dnm1l;pard3;socs5;hdac6;rerg;ttl;sytl1;sf3a3;fmnl2;taf2;crisp1;exoc5;spag16;tomm7;mapk8ip2;kif2c;oxr1;bnip3;dbn1;nckipsd;dmp1 |
| negative regulation of biological process; | 41#1182 | 4.85965E-05 | gas1;mbd1;caprin2;chek1;lbx1;tnfrsf8;psen1;ptch1;pou4f2;tbx5;arhgap20;mtl5;fhit;pou1f1;sin3a;cdkn1b;socs5;mfn2;rerg;cdkn2b;cdkn2aip;tmprss6;stat5a;adamdec1;dnmt1;il4;prdm6;nr2c1;il27ra;zbtb7a;khdrbs1;mapk8ip2;iapp;lima1;vsx2;bnip3;xrn1;nsd1;lin9;gfi1;rasa1 |
| cell cycle process; | 26#625 | 7.26747E-05 | rad52;gas1;prc1;chek1;zc3hc1;cdkn2b;stat5a;cdc25b;cdca8;cdca3;ptch1;khdrbs1;kif2c;arhgap20;dnaja2;vcp;fhit;ccnb2;mad2l1;tpd52l1;mis12;xrn1;lin9;terf1;gfi1;cdkn1b |
| negative regulation of cellular process; | 39#1137 | 0.000126481 | gas1;mbd1;caprin2;chek1;lbx1;tnfrsf8;psen1;ptch1;pou4f2;tbx5;arhgap20;mtl5;fhit;pou1f1;sin3a;cdkn1b;socs5;mfn2;rerg;cdkn2b;cdkn2aip;stat5a;adamdec1;dnmt1;il4;prdm6;nr2c1;zbtb7a;khdrbs1;mapk8ip2;iapp;lima1;vsx2;bnip3;xrn1;nsd1;lin9;gfi1;rasa1 |
| cell cycle phase; | 18#369 | 0.000132132 | rad52;gas1;chek1;zc3hc1;cdkn2b;cdc25b;cdca8;cdca3;khdrbs1;kif2c;dnaja2;ccnb2;mad2l1;tpd52l1;mis12;cdkn1b;gfi1;terf1 |
| cell cycle; | 31#839 | 0.00020298 | rad52;gas1;prc1;hdac6;smpd3;chek1;zc3hc1;cdkn2b;stat5a;cdc25b;cdca8;cdca3;ptch1;khdrbs1;kif2c;arhgap20;dnaja2;vcp;fhit;ccnb2;rps27a;mad2l1;tpd52l1;mis12;xrn1;lin9;terf1;gfi1;cdkn1b;pard3;uhrf1 |
| cell-cell signaling; | 25#640 | 0.000539234 | kcnip2;gria2;aldh9a1;gdf5;ntsr1;smpd3;htr1d;gpr119;atxn3;wisp3;tnfsf10;gna11;pcsk5;tbx5;glra1;slc6a8;acsbg1;iapp;rps27a;hprt1;nlgn1;kcnmb3;dbn1;pde7b;hcn2 |
| neurological system process#transmission of nerve impulse; | 16#330 | 0.00055878 | kcnip2;gria2;aldh9a1;ntsr1;htr1d;atxn3;gna11;glra1;slc6a8;rps27a;acsbg1;nlgn1;hprt1;kcnmb3;dbn1;pde7b |
| cell differentiation#cell development; | 40#1242 | 0.00057306 | kcnip2;gas1;gdf5;sgpp1;ntng2;lbx1;atxn3;tnfrsf8;trim28;psen1;rpe65;pou4f2;tbx5;mtl5;cidec;rps27a;nlgn1;slitrk1;pla2g6;cdkn1b;pard3;dnm1l;diablo;ttl;krt2;stat5a;il4;ripk3;tnfsf10;mark4;mapk8ip2;vcp;iapp;vsx2;bnip3;cd5;dbn1;sema6a;rasa1;rabep1 |
| biological_process; | 447#24743 | 0.000747218 | rasgrp1;ptch2;map3k12;htr1d;ckb;timm50;ddx47;ndufa1;mvp;gria4;psma5;kif2a;gpam;psen1;cdca3;il1rl2;dnmt3b;gmds;cand1;prokr2;kin;znf300;acsbg1;epha7;ipo7;tpd52l1;ptx3;angpt2;hbs1l;slc8a3;rpusd3;ptgis;uhrf1;h3f3a;slc4a2;imp3;fcgr1a;eltd1;krt2;cdc42bpb;hal;cdkn2b;rabl3;ell2;hsd3b1;taf5;ash2l;gnpnat1;kcng2;atp12a;pou2af1;il27ra;tnfsf10;ifit2;znf462;pter;ncoa3;depdc1b;fap;lin9;rabep1;terf1;rrs1;rasa1;gfi1;mctp1;kcnip2;mknk2;slc7a2;endog;mbd1;mlkl;rfc3;exosc9;tsc22d2;garnl3;rpusd4;ralgds;ncoa7;gldn;zar1;znf653;serpine2;mrpl39;slc35f4;ints4;spata2;fpgs;hace1;iqgap2;cngb3;ttyh3;gars;atg4b;galk2;rps27a;pou1f1;slitrk1;pard3;dnm1l;adam23;cdh22;atp11a;flrt3;kcnh5;dhx35;rerg;ptbp2;pde2a;sf3a3;gpr39;cct6a;rps6ka3;psd3;plcb3;slc26a11;exoc5;ripk3;tomm7;recql;tcf15;ccnb2;znf711;gstk1;fbxl7;dbn1;pde7b;atp5j;rcan3;pigf;cnksr2;slc16a6;znf546;znf75a;spred2;slc13a2;adamts6;wisp3;ptch1;pcsk5;bpi;arhgap20;fbxl22;ca8;nlgn1;clec7a;atf4;fkbp11;mrrf;krt3;colec10;znf358;tmprss6;mefv;il16;diaph1;lepr;gc;hps1;sbk1;sult2b1;ctsl2;coq6;znf606;znf263;gast;mad2l1;nploc4;sema6a;padi6;tgm6;gas1;prc1;gdf5;gpr68;yod1;kif19;ighmbp2;elovl4;fmo2;wdsub1;znf576;vti1a;psmb8;ppil2;hoxa10;itk;pfdn6;etnk1;adam11;pdk3;fhit;pomp;hprt1;spesp1;abcb7;fut10;clta;zmpste24;cald1;darc;ttl;cylc2;fmo1;fmnl2;adamdec1;cd79b;dfna5;crisp1;fbxo24;fbxo32;prdm6;dpp4;mapk8ip2;dnaja2;slc6a8;ddx46;alpl;gtdc1;opn3;dcn;pspn;sgpp1;smpd3;ntng2;btd;sox11;stx3;syt3;entpd4;upp2;tcf12;tnfrsf8;mlnr;il4i1;gna11;glra1;g6pd;mtl5;plekha2;dyrk1a;homez;laptm4a;itgb1;ewsr1;suv420h2;actr10;trpa1;cdkn1b;dok1;rad52;trip10;mfn2;abcg2;bop1;map3k1;tbc1d22a;zc3hc1;cdkn2aip;gnai2;slc26a2;znf354b;frs3;nr2c1;mark4;tpk1;pmvk;cct2;tnni3;abcc5;kcnmb3;pex26;xrn1;arhgap23;csde1;ppfibp1;psmc3ip;srebf1;rgl3;gria2;cstf3;btf3;mtif3;hsdl1;kif20a;pmm1;aldob;gpr119;atxn3;fga;prodh2;sox6;dnase1l1;igfals;psme4;taf7l;sin3a;srr;pla2g6;mis12;znf84;mthfd1;diablo;tbx21;myo1f;hdac6;atp11c;tbc1d10c;dpp3;tfap2a;fmr1;shox2;fkbp9;rfc5;gprc5d;rtn4ip1;ust;atp6ap2;dio2;stk31;iqgap1;nfe2l1;catsperb;tmem189;slc14a1;ints10;slc7a3;bnip3;nckipsd;col9a2;kit;dmp1;cdh5;arhgap24;kcnj8;cd8a;slc29a1;lbx1;bcan;eif2c3;brcc3;ppil4;pdcd1lg2;krt5;rpe65;theg;hs3st3b1;snx25;edil3;tle6;l3mbtl3;tbx5;tctn1;plekhg5;alas1;cidec;c1qb;rap1gap;ubqlnl;slc16a9;hcn2;lrat;pex11b;ell;l1cam;tbc1d21;kl;tbl1xr1;nt5c1a;brd2;plcl1;mynn;dnmt1;il4;pcmtd1;rnf4;eif5b;pla2g2c;aste1;cdca8;pdp2;khdrbs1;uxt;vcp;tmem101;lima1;rsad1;znf696;cd5;nsd1;ppp2r5e;sfxn2;chuk;aldh9a1;caprin2;rtf1;slc6a14;nfe2l2;chek1;iyd;zbtb10;lrrc47;pafah2;tceal7;cdc25b;wasf1;thbs1;fzd10;trim28;arcn1;mcoln2;tcp1;ddr1;pou4f2;tpst2;gpr83;kcnj2;cdh11;itga2b;trmt6;socs5;sh2b3;tmed6;ntsr1;myef2;sytl1;lmo3;ccdc59;stat5a;dvl1;mat2a;taf2;zbtb9;adcy5;zbtb7a;mrpl19;spag16;kif2c;cdkl5;oxr1;iapp;vsx2;rbm25;kbtbd7;rad51ap1;srebf2;znhit3 |
| cellular metabolic process; | 255#12668 | 0.000747218 | opn3;sgpp1;smpd3;map3k12;btd;timm50;sox11;ddx47;ndufa1;tcf12;upp2;entpd4;psma5;tnfrsf8;gpam;psen1;cdca3;il4i1;dnmt3b;gmds;gna11;cand1;kin;g6pd;znf300;acsbg1;epha7;homez;dyrk1a;ipo7;ptx3;ewsr1;suv420h2;hbs1l;cdkn1b;rpusd3;ptgis;uhrf1;h3f3a;rad52;imp3;bop1;cdc42bpb;map3k1;zc3hc1;hal;cdkn2b;cdkn2aip;rabl3;ell2;hsd3b1;taf5;ash2l;znf354b;pou2af1;nr2c1;il27ra;mark4;znf462;tpk1;cct2;pmvk;ncoa3;fap;lin9;terf1;rasa1;csde1;gfi1;srebf1;cstf3;mknk2;slc7a2;btf3;endog;mbd1;mtif3;mlkl;rfc3;exosc9;tsc22d2;rpusd4;pmm1;ncoa7;aldob;atxn3;fga;znf653;serpine2;sox6;dnase1l1;ints4;fpgs;hace1;taf7l;gars;atg4b;galk2;rps27a;pou1f1;sin3a;srr;mis12;pla2g6;znf84;mthfd1;adam23;tbx21;kcnh5;hdac6;dhx35;ptbp2;sf3a3;dpp3;tfap2a;fkbp9;shox2;cct6a;rps6ka3;plcb3;rfc5;ust;atp6ap2;dio2;ripk3;stk31;nfe2l1;tmem189;recql;tcf15;ints10;bnip3;znf711;gstk1;fbxl7;kit;atp5j;pigf;slc29a1;znf546;znf75a;lbx1;eif2c3;brcc3;ppil4;adamts6;hs3st3b1;rpe65;ptch1;pcsk5;l3mbtl3;tle6;tbx5;alas1;c1qb;fbxl22;ca8;atf4;ubqlnl;fkbp11;mrrf;lrat;ell;tbl1xr1;znf358;nt5c1a;tmprss6;mynn;gc;pcmtd1;dnmt1;il4;rnf4;sbk1;aste1;eif5b;pdp2;sult2b1;ctsl2;khdrbs1;uxt;coq6;vcp;znf606;znf263;lima1;rsad1;znf696;nsd1;nploc4;padi6;tgm6;chuk;aldh9a1;rtf1;slc6a14;nfe2l2;chek1;yod1;zbtb10;ighmbp2;lrrc47;elovl4;tceal7;cdc25b;wasf1;fmo2;wdsub1;znf576;trim28;psmb8;ppil2;hoxa10;tcp1;itk;pfdn6;ddr1;pou4f2;etnk1;adam11;pdk3;fhit;hprt1;tpst2;fut10;zmpste24;trmt6;socs5;myef2;ttl;lmo3;ccdc59;fmnl2;stat5a;taf2;mat2a;adamdec1;fbxo24;zbtb9;fbxo32;adcy5;prdm6;zbtb7a;dpp4;mrpl19;mapk8ip2;dnaja2;cdkl5;oxr1;ddx46;vsx2;rbm25;rad51ap1;znhit3;srebf2 |
| regulation of isotype switching to IgG isotypes; | 3#4 | 0.000877827 | tbx21;il27ra;il4 |
| isotype switching to IgG isotypes; | 3#4 | 0.000877827 | tbx21;il27ra;il4 |
| cell communication; | 127#5560 | 0.001254753 | rasgrp1;opn3;arhgap24;smpd3;cd8a;rcan3;cnksr2;map3k12;htr1d;spred2;gria4;tnfrsf8;mlnr;wisp3;psen1;il1rl2;snx25;gna11;ptch1;prokr2;pcsk5;tle6;tbx5;arhgap20;plekhg5;glra1;acsbg1;ca8;epha7;ipo7;rap1gap;nlgn1;itgb1;angpt2;clec7a;hbs1l;slc8a3;hcn2;dok1;trip10;fcgr1a;mfn2;eltd1;tbc1d21;cdc42bpb;tbc1d22a;cdkn2b;cdkn2aip;rabl3;gnai2;tmprss6;plcl1;lepr;frs3;rnf4;il27ra;tnfsf10;khdrbs1;vcp;tmem101;ncoa3;gast;kcnmb3;depdc1b;sema6a;arhgap23;ppp2r5e;rasa1;srebf1;mctp1;rgl3;kcnip2;chuk;gria2;mknk2;aldh9a1;gdf5;gpr68;garnl3;ralgds;gpr119;atxn3;fga;fzd10;igfals;iqgap2;cngb3;itk;ddr1;adam11;rps27a;hprt1;gpr83;itga2b;pard3;dnm1l;diablo;socs5;kcnh5;sh2b3;ntsr1;darc;rerg;pde2a;tbc1d10c;gpr39;tfap2a;stat5a;dvl1;adamdec1;rps6ka3;psd3;cd79b;plcb3;gprc5d;atp6ap2;adcy5;ripk3;iqgap1;mapk8ip2;slc6a8;iapp;bnip3;dbn1;nckipsd;pde7b;kit |
| sexual reproduction; | 15#315 | 0.001259637 | spata2;theg;hoxa10;psme4;catsperb;taf7l;cylc2;ptch2;chek1;glra1;mtl5;brd2;fut10;crisp1;kit |
| regulation of transcription from RNA polymerase II promoter; | 18#423 | 0.001789856 | tfap2a;stat5a;tcf12;dnmt1;il4;rnf4;trim28;nr2c1;zbtb7a;tcf15;pou4f2;pou1f1;atf4;nsd1;srebf2;gfi1;uhrf1;srebf1 |
| organelle organization and biogenesis; | 45#1526 | 0.001938879 | prc1;exosc9;map3k12;kif19;timm50;kif20a;wasf1;kif2a;sox6;atg4b;nlgn1;mis12;suv420h2;actr10;cdkn1b;dnm1l;h3f3a;imp3;krt3;trip10;hdac6;pex11b;mfn2;ttl;bop1;cdc42bpb;tbl1xr1;taf5;fmnl2;diaph1;hps1;mark4;uxt;kif2c;lima1;bnip3;pex26;nsd1;dbn1;sema6a;nploc4;nckipsd;rasa1;rrs1;terf1 |
| positive regulation of biological process; | 34#1062 | 0.002632325 | cdc25b;fga;tnfrsf8;trim28;tbx5;plekhg5;cidec;c1qb;rps27a;pou1f1;hprt1;ptx3;atf4;cdkn1b;diablo;kl;cdkn2b;cdkn2aip;stat5a;il4;rnf4;ripk3;il27ra;tnfsf10;mark4;dnaja2;tmem101;vsx2;ncoa3;bnip3;nsd1;cd5;kit;gfi1 |
| interphase of mitotic cell cycle; | 8#85 | 0.003179726 | gas1;khdrbs1;chek1;dnaja2;cdkn2b;tpd52l1;gfi1;cdkn1b |
| cell morphogenesis; | 19#478 | 0.003645423 | socs5;rerg;caprin2;ttl;ntng2;cdc42bpb;cdkn2aip;wisp3;rpe65;spag16;pou4f2;lima1;rps27a;slitrk1;sema6a;dbn1;cdkn1b;rasa1;pard3 |
| cellular structure morphogenesis; | 19#478 | 0.003645423 | socs5;rerg;caprin2;ttl;ntng2;cdc42bpb;cdkn2aip;wisp3;rpe65;spag16;pou4f2;lima1;rps27a;slitrk1;sema6a;dbn1;cdkn1b;rasa1;pard3 |
| positive regulation of cellular process; | 31#954 | 0.003737079 | diablo;cdkn2b;cdkn2aip;stat5a;cdc25b;fga;tnfrsf8;il4;rnf4;trim28;ripk3;tnfsf10;mark4;tbx5;dnaja2;plekhg5;cidec;tmem101;rps27a;ncoa3;pou1f1;vsx2;hprt1;bnip3;ptx3;cd5;atf4;nsd1;cdkn1b;gfi1;kit |
| interphase; | 8#90 | 0.004283122 | gas1;khdrbs1;chek1;dnaja2;cdkn2b;tpd52l1;gfi1;cdkn1b |
| primary metabolic process; | 251#12764 | 0.004376925 | opn3;sgpp1;smpd3;ptch2;map3k12;timm50;sox11;ddx47;tcf12;upp2;entpd4;psma5;tnfrsf8;gpam;psen1;cdca3;il4i1;dnmt3b;gmds;gna11;cand1;kin;glra1;g6pd;znf300;acsbg1;epha7;homez;dyrk1a;ipo7;ewsr1;suv420h2;hbs1l;cdkn1b;rpusd3;ptgis;uhrf1;h3f3a;rad52;imp3;bop1;cdc42bpb;map3k1;zc3hc1;hal;cdkn2aip;rabl3;ell2;hsd3b1;taf5;ash2l;znf354b;pou2af1;nr2c1;il27ra;mark4;znf462;cct2;pmvk;ncoa3;fap;lin9;terf1;rasa1;csde1;gfi1;srebf1;cstf3;mknk2;slc7a2;btf3;endog;mbd1;mtif3;mlkl;rfc3;exosc9;tsc22d2;rpusd4;pmm1;ncoa7;aldob;atxn3;fga;znf653;serpine2;sox6;dnase1l1;ints4;fpgs;hace1;taf7l;gars;atg4b;galk2;rps27a;pou1f1;sin3a;srr;mis12;pla2g6;znf84;mthfd1;pard3;adam23;tbx21;kcnh5;hdac6;dhx35;ptbp2;sf3a3;dpp3;tfap2a;fkbp9;shox2;cct6a;rps6ka3;plcb3;rfc5;ust;atp6ap2;dio2;ripk3;stk31;nfe2l1;tmem189;recql;tcf15;ints10;bnip3;znf711;fbxl7;kit;atp5j;pigf;slc29a1;znf546;znf75a;lbx1;eif2c3;brcc3;ppil4;adamts6;hs3st3b1;rpe65;theg;ptch1;pcsk5;l3mbtl3;tle6;tbx5;c1qb;fbxl22;atf4;ubqlnl;fkbp11;mrrf;ell;kl;tbl1xr1;znf358;nt5c1a;tmprss6;plcl1;mynn;pcmtd1;dnmt1;il4;rnf4;sbk1;aste1;pla2g2c;eif5b;pdp2;sult2b1;ctsl2;khdrbs1;uxt;vcp;znf606;znf263;lima1;znf696;nsd1;nploc4;padi6;tgm6;chuk;aldh9a1;rtf1;slc6a14;nfe2l2;chek1;yod1;zbtb10;ighmbp2;lrrc47;pafah2;elovl4;tceal7;cdc25b;wasf1;wdsub1;znf576;trim28;psmb8;ppil2;hoxa10;arcn1;tcp1;itk;pfdn6;ddr1;pou4f2;etnk1;adam11;pdk3;fhit;hprt1;tpst2;fut10;clta;zmpste24;trmt6;socs5;myef2;ttl;lmo3;ccdc59;fmnl2;stat5a;taf2;mat2a;adamdec1;fbxo24;zbtb9;fbxo32;adcy5;prdm6;zbtb7a;dpp4;mrpl19;mapk8ip2;dnaja2;cdkl5;ddx46;vsx2;rbm25;rad51ap1;znhit3;srebf2 |
| regulation of cell differentiation; | 10#144 | 0.004665385 | prdm6;socs5;gna11;tbx5;lbx1;iapp;nlgn1;stat5a;gfi1;il4 |
| biopolymer metabolic process; | 167#7940 | 0.004665385 | opn3;map3k12;timm50;sox11;ddx47;tcf12;psma5;psen1;cdca3;dnmt3b;gna11;cand1;kin;znf300;epha7;homez;dyrk1a;ewsr1;suv420h2;rpusd3;uhrf1;h3f3a;rad52;imp3;bop1;cdc42bpb;map3k1;zc3hc1;rabl3;ell2;taf5;ash2l;znf354b;pou2af1;nr2c1;il27ra;mark4;znf462;cct2;pmvk;ncoa3;lin9;terf1;rasa1;gfi1;csde1;srebf1;cstf3;mknk2;btf3;endog;mbd1;mlkl;rfc3;exosc9;tsc22d2;rpusd4;ncoa7;atxn3;znf653;sox6;dnase1l1;ints4;hace1;taf7l;atg4b;gars;rps27a;pou1f1;sin3a;znf84;tbx21;kcnh5;hdac6;dhx35;ptbp2;sf3a3;tfap2a;shox2;rps6ka3;rfc5;ust;dio2;ripk3;stk31;nfe2l1;tmem189;recql;tcf15;ints10;bnip3;znf711;fbxl7;kit;pigf;znf546;znf75a;lbx1;brcc3;ptch1;pcsk5;tle6;tbx5;fbxl22;atf4;ubqlnl;ell;tbl1xr1;znf358;mynn;dnmt1;il4;pcmtd1;rnf4;sbk1;aste1;pdp2;khdrbs1;vcp;znf606;znf263;znf696;nsd1;padi6;nploc4;tgm6;chuk;rtf1;nfe2l2;chek1;yod1;zbtb10;ighmbp2;tceal7;cdc25b;wdsub1;znf576;trim28;psmb8;ppil2;hoxa10;itk;ddr1;pou4f2;pdk3;tpst2;fut10;trmt6;socs5;ttl;lmo3;fmnl2;ccdc59;stat5a;taf2;fbxo24;zbtb9;fbxo32;prdm6;zbtb7a;cdkl5;ddx46;vsx2;rbm25;rad51ap1;srebf2;znhit3 |
| G2/M transition of mitotic cell cycle; | 4#17 | 0.004862568 | tpd52l1;khdrbs1;chek1;cdkn2b |
| developmental process#regulation of developmental process; | 13#236 | 0.004888648 | prdm6;socs5;gna11;ttl;tbx5;lbx1;iapp;stat5a;nlgn1;dbn1;rasa1;gfi1;il4 |
| generation of neurons#neuron differentiation; | 12#210 | 0.005988192 | kcnip2;ttl;pou4f2;ntng2;lbx1;rps27a;vsx2;nlgn1;slitrk1;sema6a;dbn1;pard3 |
| generation of neurons; | 13#242 | 0.005988192 | kcnip2;ttl;pou4f2;krt2;ntng2;lbx1;rps27a;vsx2;nlgn1;slitrk1;sema6a;dbn1;pard3 |
| spermatogenesis; | 12#212 | 0.006228574 | spata2;theg;hoxa10;psme4;catsperb;taf7l;cylc2;ptch2;mtl5;brd2;kit;crisp1 |
| male gamete generation; | 12#212 | 0.006228574 | spata2;theg;hoxa10;psme4;catsperb;taf7l;cylc2;ptch2;mtl5;brd2;kit;crisp1 |
| nucleobase, nucleoside, nucleotide and nucleic acid metabolic process; | 128#5848 | 0.006300108 | map3k12;sox11;ddx47;tcf12;upp2;entpd4;dnmt3b;gmds;cand1;kin;g6pd;znf300;homez;ewsr1;suv420h2;rpusd3;uhrf1;h3f3a;rad52;imp3;bop1;rabl3;ell2;taf5;ash2l;znf354b;pou2af1;nr2c1;il27ra;znf462;ncoa3;lin9;terf1;rasa1;gfi1;csde1;srebf1;cstf3;btf3;endog;mbd1;rfc3;exosc9;tsc22d2;rpusd4;ncoa7;atxn3;znf653;sox6;dnase1l1;ints4;fpgs;taf7l;gars;rps27a;pou1f1;sin3a;znf84;mthfd1;tbx21;kcnh5;hdac6;dhx35;ptbp2;sf3a3;tfap2a;shox2;rfc5;nfe2l1;recql;tcf15;ints10;bnip3;znf711;atp5j;znf546;slc29a1;lbx1;znf75a;eif2c3;tle6;l3mbtl3;tbx5;atf4;ell;tbl1xr1;znf358;nt5c1a;mynn;dnmt1;il4;rnf4;aste1;khdrbs1;vcp;znf606;znf263;znf696;nsd1;rtf1;nfe2l2;chek1;zbtb10;ighmbp2;tceal7;znf576;trim28;hoxa10;pou4f2;fhit;hprt1;trmt6;myef2;lmo3;fmnl2;ccdc59;stat5a;taf2;zbtb9;prdm6;adcy5;zbtb7a;ddx46;vsx2;rbm25;rad51ap1;srebf2;znhit3 |
| metabolic process#regulation of metabolic process; | 96#4150 | 0.006611926 | znf546;znf75a;lbx1;sox11;tcf12;tnfrsf8;psen1;cand1;l3mbtl3;tle6;tbx5;znf300;homez;ptx3;atf4;ewsr1;suv420h2;cdkn1b;uhrf1;ell;tbl1xr1;cdkn2b;znf358;rabl3;ell2;taf5;ash2l;znf354b;mynn;il4;dnmt1;rnf4;pou2af1;eif5b;nr2c1;khdrbs1;znf462;znf606;znf263;lima1;ncoa3;znf696;nsd1;terf1;rasa1;gfi1;csde1;srebf1;mknk2;btf3;mbd1;mtif3;rtf1;tsc22d2;nfe2l2;zbtb10;ighmbp2;tceal7;ncoa7;atxn3;serpine2;znf653;znf576;sox6;trim28;hoxa10;taf7l;pou4f2;rps27a;pou1f1;hprt1;sin3a;znf84;trmt6;kcnh5;tbx21;hdac6;lmo3;tfap2a;shox2;ccdc59;fmnl2;stat5a;taf2;atp6ap2;zbtb9;dio2;prdm6;zbtb7a;nfe2l1;tmem189;tcf15;vsx2;znf711;srebf2;znhit3 |
| mitochondrial membrane organization and biogenesis; | 4#20 | 0.008029956 | timm50;bnip3;mfn2;dnm1l |
| isotype switching#regulation of isotype switching; | 3#9 | 0.009632523 | tbx21;il27ra;il4 |
| regulation of cellular metabolic process; | 91#3933 | 0.009632523 | znf546;znf75a;lbx1;sox11;tcf12;tnfrsf8;psen1;cand1;l3mbtl3;tle6;tbx5;znf300;homez;atf4;ewsr1;suv420h2;cdkn1b;uhrf1;ell;tbl1xr1;cdkn2b;znf358;rabl3;ell2;taf5;ash2l;znf354b;mynn;dnmt1;il4;rnf4;pou2af1;eif5b;nr2c1;khdrbs1;znf462;znf606;znf263;ncoa3;znf696;nsd1;terf1;rasa1;gfi1;csde1;srebf1;mknk2;btf3;mbd1;mtif3;rtf1;tsc22d2;nfe2l2;zbtb10;ighmbp2;tceal7;ncoa7;atxn3;znf653;znf576;sox6;trim28;hoxa10;taf7l;pou4f2;rps27a;pou1f1;hprt1;sin3a;znf84;trmt6;kcnh5;tbx21;hdac6;lmo3;tfap2a;shox2;ccdc59;fmnl2;stat5a;taf2;zbtb9;dio2;prdm6;zbtb7a;nfe2l1;tcf15;vsx2;znf711;srebf2;znhit3 |
| RNA metabolic process; | 95#4155 | 0.01067396 | znf546;znf75a;lbx1;sox11;ddx47;tcf12;cand1;kin;tle6;tbx5;znf300;homez;atf4;ewsr1;suv420h2;rpusd3;uhrf1;imp3;ell;bop1;tbl1xr1;znf358;rabl3;ell2;taf5;ash2l;znf354b;mynn;il4;dnmt1;rnf4;pou2af1;nr2c1;khdrbs1;znf462;znf606;znf263;ncoa3;znf696;nsd1;rasa1;gfi1;csde1;srebf1;cstf3;btf3;mbd1;rtf1;exosc9;tsc22d2;nfe2l2;rpusd4;zbtb10;ighmbp2;tceal7;ncoa7;atxn3;znf653;znf576;sox6;trim28;ints4;hoxa10;taf7l;pou4f2;gars;pou1f1;sin3a;znf84;trmt6;kcnh5;tbx21;hdac6;ptbp2;dhx35;lmo3;sf3a3;tfap2a;shox2;ccdc59;fmnl2;stat5a;taf2;zbtb9;prdm6;zbtb7a;nfe2l1;tcf15;ddx46;ints10;vsx2;rbm25;znf711;srebf2;znhit3 |
| nervous system development#neurogenesis; | 13#262 | 0.010776695 | kcnip2;ttl;pou4f2;krt2;ntng2;lbx1;rps27a;vsx2;nlgn1;slitrk1;sema6a;dbn1;pard3 |
| gamete generation; | 13#264 | 0.011375284 | spata2;theg;hoxa10;psme4;catsperb;taf7l;cylc2;ptch2;chek1;mtl5;brd2;kit;crisp1 |
| T-helper 2 type immune response; | 3#10 | 0.012457244 | socs5;il27ra;il4 |
| metabolic process; | 277#14566 | 0.012488851 | ptch2;map3k12;timm50;ddx47;ndufa1;psma5;gpam;psen1;cdca3;dnmt3b;gmds;cand1;kin;znf300;acsbg1;epha7;ipo7;ptx3;hbs1l;rpusd3;ptgis;uhrf1;h3f3a;imp3;cdc42bpb;hal;cdkn2b;rabl3;ell2;hsd3b1;taf5;ash2l;gnpnat1;atp12a;pou2af1;il27ra;znf462;pter;ncoa3;fap;lin9;terf1;rasa1;gfi1;mknk2;slc7a2;endog;mbd1;mlkl;rfc3;exosc9;tsc22d2;rpusd4;ncoa7;znf653;serpine2;ints4;fpgs;hace1;gars;atg4b;galk2;rps27a;pou1f1;pard3;adam23;atp11a;kcnh5;dhx35;ptbp2;sf3a3;cct6a;rps6ka3;plcb3;ripk3;recql;tcf15;znf711;gstk1;fbxl7;atp5j;pigf;znf546;znf75a;adamts6;ptch1;pcsk5;fbxl22;ca8;atf4;fkbp11;mrrf;znf358;tmprss6;lepr;gc;sbk1;sult2b1;ctsl2;coq6;znf606;znf263;nploc4;padi6;tgm6;yod1;ighmbp2;elovl4;fmo2;wdsub1;znf576;psmb8;ppil2;hoxa10;itk;pfdn6;etnk1;adam11;pdk3;fhit;hprt1;fut10;clta;zmpste24;ttl;fmo1;fmnl2;adamdec1;fbxo24;fbxo32;prdm6;dpp4;mapk8ip2;dnaja2;alpl;ddx46;gtdc1;opn3;sgpp1;smpd3;btd;sox11;entpd4;upp2;tcf12;tnfrsf8;il4i1;gna11;glra1;g6pd;dyrk1a;homez;suv420h2;ewsr1;cdkn1b;rad52;bop1;zc3hc1;map3k1;cdkn2aip;znf354b;nr2c1;mark4;tpk1;pmvk;cct2;csde1;srebf1;cstf3;btf3;mtif3;hsdl1;pmm1;aldob;fga;atxn3;sox6;dnase1l1;taf7l;srr;sin3a;pla2g6;mis12;mthfd1;znf84;tbx21;hdac6;atp11c;tfap2a;dpp3;shox2;fkbp9;rfc5;rtn4ip1;ust;atp6ap2;dio2;stk31;nfe2l1;tmem189;ints10;bnip3;kit;slc29a1;lbx1;eif2c3;brcc3;ppil4;theg;rpe65;hs3st3b1;tle6;l3mbtl3;tbx5;c1qb;alas1;ubqlnl;ell;lrat;kl;tbl1xr1;nt5c1a;plcl1;mynn;il4;dnmt1;pcmtd1;rnf4;eif5b;pla2g2c;aste1;pdp2;khdrbs1;uxt;vcp;lima1;znf696;rsad1;nsd1;chuk;aldh9a1;rtf1;slc6a14;nfe2l2;iyd;chek1;zbtb10;pafah2;lrrc47;tceal7;wasf1;cdc25b;trim28;arcn1;tcp1;pou4f2;ddr1;tpst2;trmt6;socs5;myef2;lmo3;ccdc59;stat5a;mat2a;taf2;zbtb9;adcy5;zbtb7a;mrpl19;cdkl5;oxr1;vsx2;rbm25;rad51ap1;srebf2;znhit3 |
| sulfur metabolic process; | 7#86 | 0.012796273 | hs3st3b1;tpst2;mat2a;gstk1;ust;mthfd1;btd |
| vitamin metabolic process; | 7#86 | 0.012796273 | aldh9a1;rpe65;lrat;gc;tpk1;g6pd;btd |
| gene expression#regulation of gene expression; | 88#3833 | 0.014532348 | znf546;znf75a;lbx1;sox11;tcf12;tnfrsf8;dnmt3b;rpe65;cand1;l3mbtl3;tle6;tbx5;znf300;homez;atf4;ewsr1;suv420h2;uhrf1;ell;tbl1xr1;znf358;rabl3;ell2;taf5;ash2l;znf354b;mynn;dnmt1;il4;rnf4;pou2af1;eif5b;nr2c1;khdrbs1;znf462;znf606;znf263;ncoa3;znf696;nsd1;terf1;gfi1;csde1;srebf1;mknk2;btf3;mbd1;mtif3;rtf1;tsc22d2;nfe2l2;zbtb10;ighmbp2;tceal7;ncoa7;atxn3;znf653;znf576;sox6;trim28;hoxa10;taf7l;pou4f2;rps27a;pou1f1;sin3a;znf84;trmt6;kcnh5;tbx21;hdac6;lmo3;tfap2a;shox2;ccdc59;fmnl2;stat5a;taf2;zbtb9;dio2;prdm6;zbtb7a;nfe2l1;tcf15;vsx2;znf711;srebf2;znhit3 |
| intracellular signaling cascade; | 51#1965 | 0.014532712 | chuk;rasgrp1;mknk2;rcan3;map3k12;htr1d;garnl3;spred2;ralgds;psen1;iqgap2;itk;gna11;plekhg5;ca8;rap1gap;clec7a;pard3;dnm1l;diablo;socs5;sh2b3;mfn2;rerg;tbc1d21;tbc1d10c;cdc42bpb;tbc1d22a;rabl3;gnai2;tmprss6;plcl1;stat5a;dvl1;psd3;plcb3;atp6ap2;rnf4;adcy5;iqgap1;tnfsf10;mapk8ip2;vcp;tmem101;ncoa3;bnip3;depdc1b;rasa1;kit;rgl3;mctp1 |
| somatic diversification of immunoglobulins during immune response; | 3#11 | 0.014532712 | tbx21;il27ra;il4 |
| somatic recombination of immunoglobulin genes during immune response; | 3#11 | 0.014532712 | tbx21;il27ra;il4 |
| isotype switching; | 3#11 | 0.014532712 | tbx21;il27ra;il4 |
| immunoglobulin production during immune response; | 3#11 | 0.014532712 | tbx21;il27ra;il4 |
| negative regulation of cell differentiation; | 6#65 | 0.014532712 | lbx1;prdm6;iapp;stat5a;il4;gfi1 |
| multicellular organismal process#regulation of multicellular organismal process; | 13#277 | 0.014532712 | kcnip2;tbx21;il27ra;kl;glra1;c1qb;tnni3;rps27a;iapp;stat5a;kcng2;cd5;il4 |
| regulation of B cell activation; | 4#25 | 0.014532712 | tbx21;stat5a;il27ra;il4 |
| macromolecule metabolic process; | 218#11144 | 0.016661533 | opn3;ptch2;map3k12;timm50;sox11;ddx47;tcf12;psma5;tnfrsf8;psen1;cdca3;dnmt3b;gmds;gna11;cand1;kin;glra1;g6pd;znf300;epha7;homez;dyrk1a;ipo7;ewsr1;suv420h2;hbs1l;cdkn1b;rpusd3;uhrf1;h3f3a;rad52;imp3;bop1;cdc42bpb;map3k1;zc3hc1;cdkn2aip;rabl3;ell2;taf5;ash2l;znf354b;pou2af1;nr2c1;il27ra;mark4;znf462;cct2;pmvk;ncoa3;fap;lin9;terf1;rasa1;csde1;gfi1;srebf1;cstf3;mknk2;btf3;endog;mbd1;mtif3;mlkl;rfc3;exosc9;tsc22d2;rpusd4;pmm1;ncoa7;aldob;atxn3;fga;znf653;serpine2;sox6;dnase1l1;ints4;hace1;taf7l;gars;atg4b;galk2;rps27a;pou1f1;sin3a;mis12;znf84;pard3;adam23;tbx21;kcnh5;hdac6;dhx35;ptbp2;sf3a3;dpp3;tfap2a;shox2;fkbp9;cct6a;rps6ka3;rfc5;ust;atp6ap2;dio2;ripk3;stk31;nfe2l1;tmem189;recql;tcf15;ints10;bnip3;znf711;fbxl7;kit;pigf;znf546;znf75a;lbx1;eif2c3;brcc3;ppil4;adamts6;theg;hs3st3b1;ptch1;pcsk5;tle6;tbx5;c1qb;fbxl22;atf4;ubqlnl;fkbp11;mrrf;ell;kl;tbl1xr1;znf358;tmprss6;mynn;dnmt1;il4;pcmtd1;rnf4;sbk1;aste1;eif5b;pdp2;ctsl2;khdrbs1;uxt;vcp;znf606;znf263;lima1;znf696;nsd1;nploc4;padi6;tgm6;chuk;rtf1;nfe2l2;chek1;yod1;zbtb10;ighmbp2;lrrc47;tceal7;cdc25b;wasf1;wdsub1;znf576;trim28;psmb8;ppil2;hoxa10;arcn1;itk;tcp1;pfdn6;ddr1;pou4f2;adam11;pdk3;hprt1;tpst2;fut10;clta;zmpste24;trmt6;socs5;ttl;lmo3;ccdc59;fmnl2;stat5a;adamdec1;taf2;fbxo24;zbtb9;fbxo32;prdm6;zbtb7a;dpp4;mrpl19;mapk8ip2;dnaja2;cdkl5;ddx46;vsx2;rbm25;rad51ap1;srebf2;znhit3 |
| GO:0048304,positive regulation of isotype switching to IgG isotypes; | 2#3 | 0.016661533 | tbx21;il4 |
| negative regulation of epithelial cell proliferation; | 3#12 | 0.01764803 | gas1;cdkn1b;cdkn2b |
| generation of neurons#neuron differentiation#neuron development; | 9#154 | 0.020079478 | kcnip2;ttl;pou4f2;ntng2;rps27a;slitrk1;sema6a;dbn1;pard3 |
| neurological system process#transmission of nerve impulse#synaptic transmission; | 13#290 | 0.020557373 | kcnip2;gria2;aldh9a1;ntsr1;htr1d;glra1;slc6a8;rps27a;hprt1;nlgn1;atxn3;dbn1;pde7b |
| regulation of cell proliferation; | 17#469 | 0.021194983 | gas1;mfn2;rerg;chek1;cdkn2b;lbx1;stat5a;cdc25b;fga;tnfrsf8;il4;dnaja2;tbx5;vsx2;pou1f1;kit;cdkn1b |
| cell differentiation#cell development#cellular morphogenesis during differentiation; | 8#126 | 0.021194983 | rpe65;ttl;pou4f2;ntng2;rps27a;slitrk1;sema6a;pard3 |
| somatic recombination of immunoglobulin gene segments; | 3#13 | 0.021194983 | tbx21;il27ra;il4 |
| regulation of signal transduction; | 25#800 | 0.021355281 | rasgrp1;socs5;mfn2;tbc1d21;cnksr2;tbc1d10c;cdc42bpb;tbc1d22a;cdkn2b;cdkn2aip;garnl3;ralgds;spred2;psd3;atp6ap2;iqgap1;tnfsf10;iqgap2;ptch1;mapk8ip2;plekhg5;tmem101;rap1gap;rasa1;rgl3 |
| signal transduction; | 111#5142 | 0.022348299 | rasgrp1;opn3;arhgap24;cd8a;rcan3;cnksr2;map3k12;htr1d;spred2;gria4;tnfrsf8;mlnr;wisp3;psen1;il1rl2;gna11;ptch1;prokr2;tle6;arhgap20;plekhg5;glra1;ca8;epha7;ipo7;rap1gap;itgb1;angpt2;clec7a;hbs1l;dok1;trip10;fcgr1a;mfn2;eltd1;tbc1d21;cdc42bpb;cdkn2b;tbc1d22a;cdkn2aip;rabl3;gnai2;tmprss6;plcl1;lepr;frs3;rnf4;il27ra;tnfsf10;khdrbs1;vcp;tmem101;ncoa3;gast;depdc1b;sema6a;arhgap23;ppp2r5e;rasa1;mctp1;rgl3;kcnip2;chuk;gria2;mknk2;gdf5;gpr68;garnl3;ralgds;gpr119;fga;fzd10;igfals;iqgap2;itk;cngb3;ddr1;adam11;gpr83;itga2b;pard3;dnm1l;diablo;socs5;kcnh5;sh2b3;ntsr1;darc;rerg;pde2a;tbc1d10c;gpr39;tfap2a;stat5a;dvl1;adamdec1;rps6ka3;psd3;cd79b;plcb3;gprc5d;atp6ap2;adcy5;ripk3;iqgap1;mapk8ip2;iapp;bnip3;nckipsd;kit;pde7b |
| negative regulation of cell proliferation; | 11#225 | 0.022891459 | gas1;mfn2;rerg;chek1;tbx5;cdkn2b;lbx1;pou1f1;vsx2;cdkn1b;tnfrsf8 |
| heterocycle metabolic process; | 8#130 | 0.024630261 | fpgs;nfe2l1;alas1;btd;hprt1;mat2a;rsad1;mthfd1 |
| system process; | 41#1539 | 0.024641822 | kcnip2;gria2;aldh9a1;opn3;kcnj8;htr1d;elovl4;atxn3;fga;prodh2;mlnr;rpe65;cngb3;gna11;pou4f2;glra1;acsbg1;rps27a;hprt1;nlgn1;trpa1;cdkn1b;hcn2;cald1;ntsr1;lrat;diaph1;kcng2;dfna5;gprc5d;atp6ap2;hps1;adcy5;slc6a8;iapp;tnni3;vsx2;kcnmb3;gast;dbn1;pde7b |
| regulation of transcription, DNA-dependent; | 77#3358 | 0.027447777 | znf546;znf75a;lbx1;sox11;tcf12;cand1;tle6;tbx5;znf300;homez;atf4;ewsr1;suv420h2;uhrf1;ell;tbl1xr1;znf358;rabl3;ell2;taf5;ash2l;znf354b;mynn;dnmt1;il4;rnf4;pou2af1;nr2c1;khdrbs1;znf462;znf606;znf263;ncoa3;znf696;nsd1;gfi1;csde1;srebf1;btf3;mbd1;rtf1;tsc22d2;nfe2l2;zbtb10;ighmbp2;tceal7;ncoa7;znf653;atxn3;znf576;sox6;trim28;hoxa10;taf7l;pou4f2;pou1f1;sin3a;znf84;kcnh5;tbx21;hdac6;lmo3;tfap2a;shox2;ccdc59;fmnl2;stat5a;taf2;zbtb9;prdm6;zbtb7a;nfe2l1;tcf15;vsx2;znf711;srebf2;znhit3 |
| regulation of membrane potential; | 4#31 | 0.027447777 | bnip3;kcnmb3;glra1;hcn2 |
| cytoskeleton organization and biogenesis; | 22#686 | 0.027447777 | prc1;trip10;krt3;ttl;cdc42bpb;kif19;fmnl2;kif20a;wasf1;diaph1;kif2a;mark4;uxt;kif2c;lima1;nlgn1;sema6a;dbn1;actr10;nckipsd;cdkn1b;rasa1 |
| negative regulation of developmental process; | 6#77 | 0.028018959 | lbx1;prdm6;iapp;stat5a;il4;gfi1 |
| somatic cell DNA recombination; | 3#15 | 0.028188282 | tbx21;il27ra;il4 |
| immunoglobulin production#somatic diversification of immunoglobulins; | 3#15 | 0.028188282 | tbx21;il27ra;il4 |
| somatic diversification of immune receptors via germline recombination within a single locus; | 3#15 | 0.028188282 | tbx21;il27ra;il4 |
| generation of neurons#neuron differentiation#neuron development#neurite development; | 8#135 | 0.028188282 | ttl;pou4f2;ntng2;rps27a;slitrk1;sema6a;dbn1;pard3 |
| epithelial cell proliferation; | 4#33 | 0.03142323 | gas1;kit;cdkn1b;cdkn2b |
| cell projection morphogenesis; | 10#204 | 0.03142323 | ttl;spag16;pou4f2;ntng2;rps27a;lima1;slitrk1;sema6a;dbn1;pard3 |
| cell part morphogenesis; | 10#204 | 0.03142323 | ttl;spag16;pou4f2;ntng2;rps27a;lima1;slitrk1;sema6a;dbn1;pard3 |
| cell projection morphogenesis#cell projection organization and biogenesis; | 10#204 | 0.03142323 | ttl;spag16;pou4f2;ntng2;rps27a;lima1;slitrk1;sema6a;dbn1;pard3 |
| immune system development#somatic diversification of immune receptors; | 3#16 | 0.03244168 | tbx21;il27ra;il4 |
| cell proliferation; | 23#745 | 0.03559604 | gas1;mfn2;rerg;krt2;chek1;cdkn2b;lbx1;stat5a;cdc25b;fga;tnfrsf8;il4;ptch1;khdrbs1;kif2c;tbx5;dnaja2;pou1f1;vsx2;cd5;cdkn1b;kit;uhrf1 |
| cell activation; | 10#209 | 0.036328386 | socs5;tbx21;il27ra;cd8a;krt2;stat5a;cd5;clec7a;fga;il4 |
| cell division; | 11#245 | 0.036540118 | cdca3;cdca8;prc1;zc3hc1;ccnb2;cdc25b;mad2l1;mis12;rasa1;terf1;pard3 |
| membrane organization and biogenesis; | 12#284 | 0.038588858 | kcnip2;trip10;fcgr1a;mfn2;timm50;bnip3;ptx3;clec7a;nploc4;crisp1;rabep1;dnm1l |
| camera-type eye development#retina development in camera-type eye; | 2#5 | 0.038588858 | vsx2;pou4f2 |
| axonogenesis; | 7#114 | 0.039540748 | rps27a;slitrk1;ttl;pou4f2;sema6a;ntng2;pard3 |
| sensory organ development; | 6#86 | 0.041471598 | gas1;vsx2;pou4f2;dfna5;cdkn1b;gfi1 |
| positive regulation of B cell activation; | 3#18 | 0.041752487 | tbx21;stat5a;il4 |
| immunoglobulin production; | 3#18 | 0.041752487 | tbx21;il27ra;il4 |
| fat-soluble vitamin metabolic process; | 3#18 | 0.041752487 | rpe65;lrat;gc |
| hexose biosynthetic process; | 4#37 | 0.042367992 | gmds;pmm1;atf4;aldob |
| immune system development; | 9#182 | 0.043066719 | socs5;tbx21;sh2b3;il27ra;stat5a;ash2l;fut10;il4;kit |
| negative regulation of growth; | 5#61 | 0.043669213 | rerg;caprin2;ptch1;cdkn1b;cdkn2aip |
| transcription, DNA-dependent; | 77#3439 | 0.044152346 | znf546;znf75a;lbx1;sox11;tcf12;cand1;tle6;tbx5;znf300;homez;atf4;ewsr1;suv420h2;uhrf1;ell;tbl1xr1;znf358;rabl3;ell2;taf5;ash2l;znf354b;mynn;dnmt1;il4;rnf4;pou2af1;nr2c1;khdrbs1;znf462;znf606;znf263;ncoa3;znf696;nsd1;gfi1;csde1;srebf1;btf3;mbd1;rtf1;tsc22d2;nfe2l2;zbtb10;ighmbp2;tceal7;ncoa7;znf653;atxn3;znf576;sox6;trim28;hoxa10;taf7l;pou4f2;pou1f1;sin3a;znf84;kcnh5;tbx21;hdac6;lmo3;tfap2a;shox2;ccdc59;fmnl2;stat5a;taf2;zbtb9;prdm6;zbtb7a;nfe2l1;tcf15;vsx2;znf711;srebf2;znhit3 |
| monosaccharide biosynthetic process; | 4#38 | 0.044647397 | gmds;pmm1;atf4;aldob |
| alcohol biosynthetic process; | 4#38 | 0.044647397 | gmds;pmm1;atf4;aldob |
| RNA biosynthetic process; | 77#3444 | 0.044877023 | znf546;znf75a;lbx1;sox11;tcf12;cand1;tle6;tbx5;znf300;homez;atf4;ewsr1;suv420h2;uhrf1;ell;tbl1xr1;znf358;rabl3;ell2;taf5;ash2l;znf354b;mynn;dnmt1;il4;rnf4;pou2af1;nr2c1;khdrbs1;znf462;znf606;znf263;ncoa3;znf696;nsd1;gfi1;csde1;srebf1;btf3;mbd1;rtf1;tsc22d2;nfe2l2;zbtb10;ighmbp2;tceal7;ncoa7;znf653;atxn3;znf576;sox6;trim28;hoxa10;taf7l;pou4f2;pou1f1;sin3a;znf84;kcnh5;tbx21;hdac6;lmo3;tfap2a;shox2;ccdc59;fmnl2;stat5a;taf2;zbtb9;prdm6;zbtb7a;nfe2l1;tcf15;vsx2;znf711;srebf2;znhit3 |
| response to external stimulus; | 20#633 | 0.045137247 | opn3;gpr68;cdkn2b;gnai2;tmprss6;mefv;stat5a;il16;aldob;fga;thbs1;il4;nfe2l1;glra1;c1qb;ptx3;clec7a;fut10;gfi1;srebf1 |
| ubiquitin cycle; | 18#549 | 0.045432446 | socs5;yod1;zc3hc1;tbl1xr1;brcc3;wdsub1;fbxo24;fbxo32;cdca3;ppil2;hace1;cand1;atg4b;fbxl22;rps27a;nploc4;fbxl7;uhrf1 |
| generation of neurons#neuron differentiation#neuron development#neuron morphogenesis during differentiation; | 7#120 | 0.045832155 | rps27a;slitrk1;ttl;pou4f2;sema6a;ntng2;pard3 |
| generation of neurons#neuron differentiation#neuron development#neuron morphogenesis during differentiation#neurite morphogenesis; | 7#120 | 0.045832155 | rps27a;slitrk1;ttl;pou4f2;sema6a;ntng2;pard3 |
| positive regulation of isotype switching; | 2#6 | 0.04894201 | tbx21;il4 |
