# Supplementary material for: Genome-wide analysis reveals signatures of selection for important traits in domestic sheep from different ecoregions
Source: BMC Genomics. 2016 Nov 3;17:863. doi: 10.1186/s12864-016-3212-2 (PMC5094087; doi:10.1186/s12864-016-3212-2)

**Additional file 8: Figure S2.** Biological processes and KEGG pathway enrichment in genes containing missense SNPs or stop gained/loss variants. A, Biological processes enrichment in gene containing missense SNPs or stop gained/loss variants in Mongolian sheep but not in Small-tailed Han sheep or Duolang sheep. B, Biological processes enrichment in gene containing missense SNPs or stop gained/loss variants in both Small-tailed Han sheep and Duolang sheep, but not in Mongolian sheep. C, KEGG pathway enrichment in genes containing missense SNPs or stop gained/loss variants in Mongolian sheep but not in Small-tailed Han sheep or Duolang sheep. D, KEGG pathway enrichment in genes containing missense SNPs or stop gained/loss variants in both Small-tailed Han sheep and Duolang sheep, but not in Mongolian sheep. Biological processes and KEGG pathway related to reproduction are labeled in red.


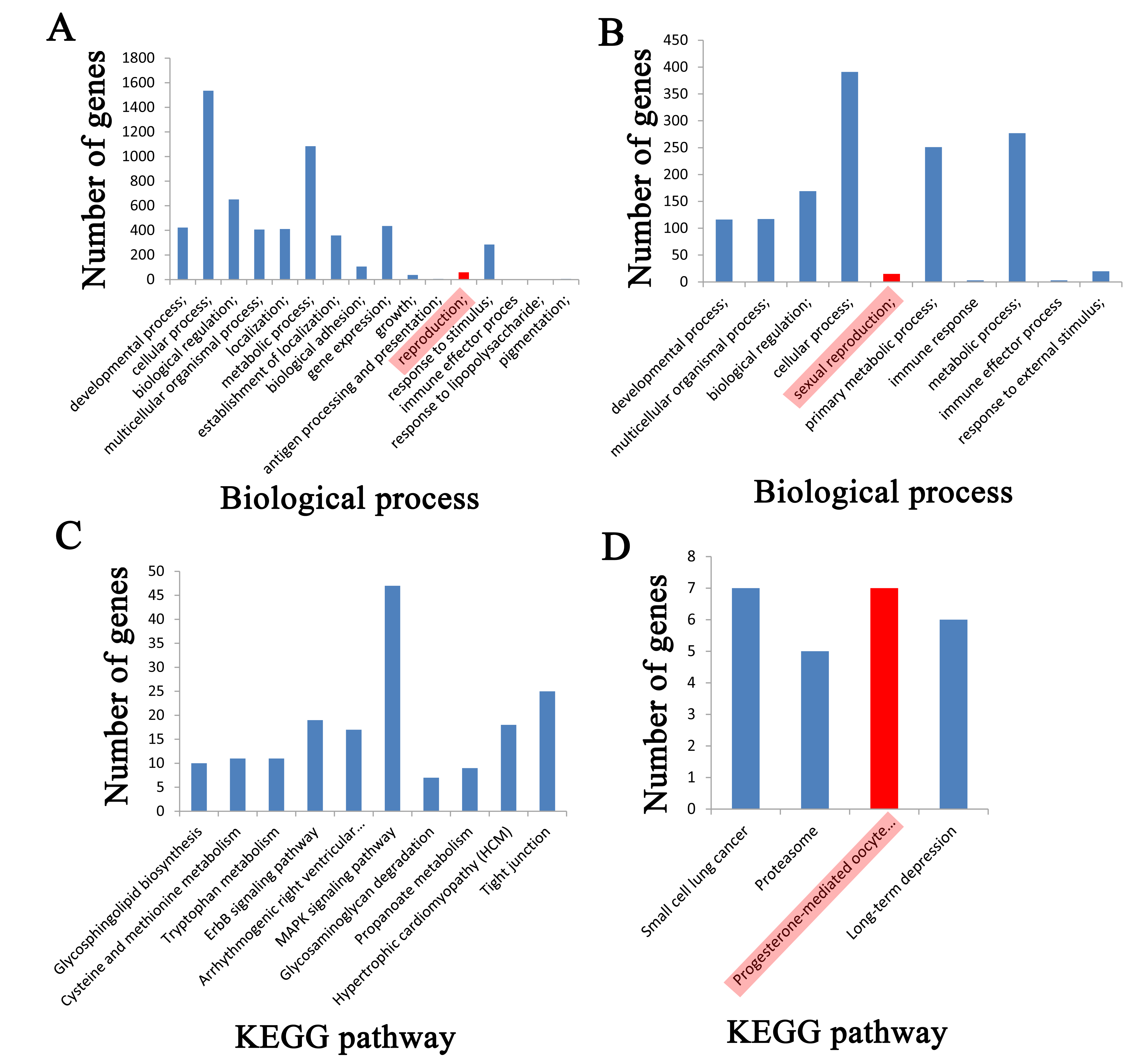

Supplement: Additional file 8: Figure S2. — Biological processes and KEGG pathway enrichment in genes containing missense SNPs or stop gained/loss variants. A, Biological processes enrichment in gene containing missense SNPs or stop gained/loss variants in Mongolian sheep but not in Small-tailed Han sheep or Duolang sheep. B, Biological processes enrichment in gene containing missense SNPs or stop gained/loss variants in both Small-tailed Han sheep and Duolang sheep, but not in Mongolian sheep. C, KEGG pathway enrichment in genes containing missense SNPs or stop gained/loss variants in Mongolian sheep but not in Small-tailed Han sheep or Duolang sheep. D, KEGG pathway enrichment in genes containing missense SNPs or stop gained/loss variants in both Small-tailed Han sheep and Duolang sheep, but not in Mongolian sheep. Biological processes and KEGG pathway related to reproduction are labeled in red. (DOC 955 kb) [file 12864_2016_3212_MOESM8_ESM.doc]
