# Supplementary material for: Genome-wide analysis reveals signatures of selection for important traits in domestic sheep from different ecoregions
Source: BMC Genomics. 2016 Nov 3;17:863. doi: 10.1186/s12864-016-3212-2 (PMC5094087; doi:10.1186/s12864-016-3212-2)
Supplement: Additional file 9: Table S7. — Enriched KEGG pathways among genes containing missense SNPs or stop gained/loss variants in Mongolian sheep but not in Small-tailed Han sheep or Duolang sheep. (DOC 44 kb) [file 12864_2016_3212_MOESM9_ESM.doc]

**Additional file 9: Table S7**. Enriched KEGG pathways among genes containing missense SNPs or stop gained/loss variants in Mongolian sheep but not in Small-tailed Han sheep or Duolang sheep.

| Category | Term | Count | P  Value | Genes |
| --- | --- | --- | --- | --- |
| KEGG_PATHWAY | hsa00601:Glycosphingolipid biosynthesis | 10 | 0.004176 | ST3GAL3, B3GNT5, B3GALT2, B3GALT1, B3GALT5, B3GNT4, ST3GAL4, FUT3, FUT1, B3GNT2 |
| KEGG_PATHWAY | hsa00270:Cysteine and methionine metabolism | 11 | 0.01275 | LDHC, LDHB, LDHA, SRM, BHMT, MTAP, AHCYL2, APIP, AMD1, SMS, CBS |
| KEGG_PATHWAY | hsa00380:Tryptophan metabolism | 11 | 0.038719 | WARS, KYNU, ALDH7A1, MAOA, IDO2, WARS2, KMO, OGDH, HADH, HADHA, AFMID |
| KEGG_PATHWAY | hsa04012:ErbB signaling pathway | 19 | 0.044204 | PRKCA, NRG3, CAMK2G, ELK1, MAPK1, NRAS, PTK2, PAK3, GSK3B, NCK1, ARAF, GAB1, CAMK2D, SHC1, PAK1, AREG, MAP2K7, ABL2, AKT2 |
| KEGG_PATHWAY | hsa05412:Arrhythmogenic right ventricular cardiomyopathy (ARVC) | 17 | 0.048514 | ACTN4, CACNG7, CACNG6, GJA1, CACNB3, CACNG3, CACNA2D3, CTNNA1, CTNNA3, CTNNB1, DES, SGCG, ATP2A2, DSC2, CACNA1F, SGCA, SGCB |
| KEGG_PATHWAY | hsa04010:MAPK signaling pathway | 47 | 0.05275 | FGF6, IL1R2, FGF5, MAPKAPK5, GNA12, MAP4K2, DUSP10, ELK1, CACNB3, TGFB1, ATF2, TGFB2, MAP3K7, HSPA1L, MAP3K4, ELK4, MAP3K2, PLA2G12A, RASGRP2, PAK1, TRAF6, NFATC2, FGF1, MAP2K7, RASA1, AKT2, PRKCA, NTF4, TAOK2, NLK, CACNG7, TAOK3, CACNG6, NR4A1, FGF22, CACNG3, ECSIT, FGF20, CACNA2D3, MAP4K3, MAPK1, NRAS, HSPB1, CACNA1F, MAPK8IP1, CD14, NGF |
| KEGG_PATHWAY | hsa00531:Glycosaminoglycan degradation | 7 | 0.056568 | HGSNAT, GNS, HYAL2, HYAL3, HPSE, HEXA, GALNS |
| KEGG_PATHWAY | hsa00640:Propanoate metabolism | 9 | 0.061214 | LDHC, LDHB, LDHA, MUT, ALDH7A1, MCEE, ACACA, SUCLA2, HADHA |
| KEGG_PATHWAY | hsa05410:Hypertrophic cardiomyopathy (HCM) | 18 | 0.065174 | CACNG7, CACNG6, PRKAG2, CACNB3, CACNG3, CACNA2D3, TPM1, TPM4, TGFB1, TGFB2, DES, SGCG, ATP2A2, PRKAA1, PRKAA2, CACNA1F, SGCA, SGCB |
| KEGG_PATHWAY | hsa04530:Tight junction | 25 | 0.096169 | CLDN17, GNAI3, CLDN14, CTNNB1, EXOC3, PPP2R2B, AKT2, PRKCA, MAGI2, ACTN4, HCLS1, CSNK2B, PRKCI, MPP5, MYLPF, CRB3, MYL12B, CLDN20, CTNNA1, CTNNA3, NRAS, CGN, CLDN1, CLDN2, JAM3 |
