# Supplementary material for: Genome-wide analysis reveals signatures of selection for important traits in domestic sheep from different ecoregions
Source: BMC Genomics. 2016 Nov 3;17:863. doi: 10.1186/s12864-016-3212-2 (PMC5094087; doi:10.1186/s12864-016-3212-2)
Supplement: Additional file 10: Table S8. — Enriched KEGG pathways among genes containing missense SNPs or stop gained/loss variants in both Small-tailed Han sheep and Duolang sheep, but not in Mongolian sheep. (DOC 34 kb) [file 12864_2016_3212_MOESM10_ESM.doc]

**Additional file 10: Table S8**. Enriched KEGG pathways among genes containing missense SNPs or stop gained/loss variants in both Small-tailed Han sheep and Duolang sheep, but not in Mongolian sheep.

| Category | Term | Count | P  Value | Genes |
| --- | --- | --- | --- | --- |
| KEGG_PATHWAY | hsa05222:Small cell lung cancer | 7 | 0.077574 | FHIT, CDKN1B, CDKN2B, PIK3R5, ITGB1, CHUK, ITGA2B |
| KEGG_PATHWAY | hsa03050:Proteasome | 5 | 0.084716 | PSMA5, POMP, PSME4, PSMB8, PSMB11 |
| KEGG_PATHWAY | hsa04914:Progesterone-mediated oocyte maturation | 7 | 0.084766 | RPS6KA3, MAD2L1, CCNB2, GNAI2, ADCY5, PIK3R5, CDC25B |
| KEGG_PATHWAY | hsa04730:Long-term depression | 6 | 0.097968 | PLCB3, GNAI2, GRIA2, GNA11, PLA2G6, PLA2G2C |
