# Supplementary material for: Genome-wide analysis reveals signatures of selection for important traits in domestic sheep from different ecoregions
Source: BMC Genomics. 2016 Nov 3;17:863. doi: 10.1186/s12864-016-3212-2 (PMC5094087; doi:10.1186/s12864-016-3212-2)
Supplement: Additional file 11: Table S9. — Enriched GO terms among genes containing missense SNPs in promoter regions in Mongolian sheep but not in Small-tailed Han sheep or Duolang sheep. (DOC 570 kb) [file 12864_2016_3212_MOESM11_ESM.doc]

**Additional file 11: Table S9**. Enriched GO terms among genes containing missense SNPs in promoter regions in Mongolian sheep but not in Small-tailed Han sheep or Duolang sheep.

| GO term | Gene count | P value | genes |
| --- | --- | --- | --- |
| developmental process; | 399#3347 | 1.13E-41 | fadd;prickle2;naglu;tead4;rnf34;ccr4;psen1;sema4c;pick1;pth;dsg4;ttn;smpd1;mov10l1;mesdc2;bdnf;lhx3;rbp2;casc5;lfng;nr2e1;notch1;myod1;fzd6;il27ra;tpt1;glis2;hoxc10;cav2;capg;mog;vsx1;mef2b;gpr56;brca1;gnpat;sox14;nptn;nudc;edn1;gfra3;myog;chpt1;cd2;zar1;thbd;actn2;cecr2;nkx2-5;dock7;sbf2;spata2;tnn;casp8;bcar1;pou1f1;dip2a;lrp1;fez2;ppm1f;socs6;tia1;ctnnbl1;edf1;vav2;canx;cherp;plxnb2;tnc;sox9;hexb;pbx4;mfng;arfip2;unc13b;lhcgr;boc;cd320;wnt8b;slc25a6;snai1;tcf15;rps3a;smo;ing4;pik3r2;tpd52;dbn1;olfm1;gsk3b;chrm1;phf17;adora2b;fgd5;nck1;ddx1;dyrk3;traf6;spag6;timp1;aldh5a1;pes1;tuft1;pax3;clasp1;dscam;ntf3;fgf1;naip;snca;mc1r;emp3;morf4l1;gpr45;zscan2;prkca;txndc8;pitpnb;sertad3;c8b;sfxn1;chrdl2;gadd45a;b3gnt2;col12a1;stat3;avil;dag1;dlg1;brms1l;spata18;dclk1;pcdhb2;gaa;mlxipl;clec3a;smad5;sirt2;gdf5;npas2;cspg4;plcg1;clptm1;hoxd8;atrn;hoxb13;chl1;als2;duox2;trim35;kif3b;igsf10;pitx2;igfbp2;pdcd1;pex13;ide;isl2;il19;wnt9a;ndrg4;spesp1;socs7;trim15;fut10;ift57;sox13;malt1;hoxb4;spred1;itgb7;steap3;mylip;cylc2;fblim1;ccar1;cdsn;hsd11b1;notch2;mrpl40;akt1s1;bcl2a1;napa;bub1b;mapk8ip2;bcl2l13;rufy3;hpcal4;mmp11;nppb;pcdhb14;trim13;wfs1;shroom1;ift88;sdcbp;sgpp1;ntng2;cd3d;corin;pogk;foxn1;megf8;piwil2;gna11;ppp1r13b;egln1;evpl;cxcl1;znrd1;map2k1;taok2;smarcb1;brd8;pdpn;adrb1;tcf25;pln;gata3;atp10a;wnt4;st6gal2;pcolce;il17c;casp8ap2;pycard;bmp10;tnni3;cideb;aven;krt4;sh3gl2;armc10;arhgap5;cyp11a1;krt13;tmem176b;ercc3;wnt3a;proc;ambn;parp4;ube2b;atxn3;rttn;tbx10;traf1;rtn4;bmpr1a;dyrk2;gap43;atpif1;unc45b;pou2f3;dapk2;asb1;kctd11;srpk2;sema4f;btg4;eya2;psen2;sh2d2a;prelp;cda;obscn;lmo2;dmrtb1;cdkl2;nod1;hes5;pcdhb11;rnf216;cchcr1;ephb2;ehf;ccm2;ereg;znf22;gda;acrv1;pdcd10;gal3st1;sptbn2;ripk1;srf;faslg;cryga;wwp1;pcdha1;meox1;notch4;scube1;angpt1;poll;ptprr;camk1;rab3d;rad21;tubb3;hsf4;eif5a;lep;mreg;hoxc8;ugcg;mef2d;sftpb;cidec;lhfpl5;krt14;erg;fcrla;prrx2;dkk1;rnf111;nrcam;pou5f1;stambp;mkks;nanos3;gypc;gdf10;crispld2;nkx2-6;siva1;barx1;nts;ezh1;unc5a;c1galt1;piwil1;ctnnal1;ptprc;hspd1;brca2;tbr1;plxnc1;nedd9;stx2;nhej1;eef1a2;emp2;sfrp1;api5;ncoa6;aldh3a2;htatip2;alg5;phlda1;tlr3;ppt1;sphk1;ssh1;sfn;nrsn1;tnfrsf11a;lhb;rffl;trim28;crx;shank3;ddx41;suv39h1;pacs2;serpini1;pde3b;yeats4;bik;casp14;bard1;tnfrsf21;bmp6;gzf1;cdkn1a;fgf7;sh2b3;mfrp;alb;atg12;chodl;msh4;emp1;elavl1;angptl3;mmp19;ext2;rere;cd164;bicc1;bves;sycp3;hap1;trim10;ahnak;atf5;nod2;ush1c;msi1 |
| anatomical structure development; | 264#2005 | 1.17E-35 | prickle2;naglu;tead4;ccr4;sema4c;pick1;pth;dsg4;ttn;smpd1;mesdc2;bdnf;lhx3;rbp2;casc5;lfng;nr2e1;notch1;myod1;fzd6;il27ra;glis2;hoxc10;cav2;capg;mog;mef2b;gpr56;gnpat;sox14;nptn;edn1;gfra3;myog;chpt1;cd2;actn2;nkx2-5;dock7;sbf2;tnn;bcar1;pou1f1;fez2;socs6;vav2;canx;cherp;tnc;sox9;hexb;pbx4;arfip2;lhcgr;boc;cd320;wnt8b;snai1;tcf15;smo;tpd52;dbn1;olfm1;adora2b;phf17;chrm1;fgd5;nck1;dyrk3;spag6;timp1;pes1;aldh5a1;tuft1;pax3;clasp1;dscam;ntf3;fgf1;naip;snca;emp3;morf4l1;gpr45;sertad3;sfxn1;chrdl2;b3gnt2;col12a1;stat3;avil;dlg1;dag1;brms1l;dclk1;pcdhb2;gaa;mlxipl;clec3a;smad5;gdf5;sirt2;npas2;cspg4;hoxb13;als2;chl1;duox2;igsf10;pitx2;igfbp2;pex13;ndrg4;socs7;trim15;fut10;sox13;mylip;fblim1;cdsn;hsd11b1;notch2;mrpl40;napa;rufy3;hpcal4;mmp11;nppb;pcdhb14;trim13;wfs1;shroom1;ift88;sdcbp;ntng2;cd3d;corin;foxn1;gna11;egln1;evpl;cxcl1;znrd1;map2k1;taok2;brd8;smarcb1;pdpn;adrb1;pln;tcf25;atp10a;gata3;il17c;bmp10;tnni3;krt4;sh3gl2;armc10;arhgap5;krt13;cyp11a1;tmem176b;ercc3;wnt3a;ambn;ube2b;atxn3;tbx10;rtn4;bmpr1a;gap43;atpif1;unc45b;pou2f3;asb1;sema4f;kctd11;btg4;eya2;sh2d2a;cda;prelp;hes5;pcdhb11;ephb2;ccm2;ehf;ereg;znf22;gda;gal3st1;srf;cryga;wwp1;pcdha1;scube1;notch4;poll;angpt1;rab3d;camk1;tubb3;hsf4;mreg;lep;hoxc8;mef2d;ugcg;sftpb;lhfpl5;krt14;dkk1;nrcam;pou5f1;mkks;gypc;gdf10;nkx2-6;crispld2;barx1;nts;ezh1;c1galt1;ptprc;brca2;tbr1;nedd9;stx2;nhej1;sfrp1;ncoa6;aldh3a2;htatip2;tlr3;ppt1;sphk1;nrsn1;sfn;ssh1;tnfrsf11a;lhb;shank3;crx;pde3b;serpini1;yeats4;casp14;bmp6;gzf1;fgf7;cdkn1a;sh2b3;chodl;msh4;emp1;angptl3;mmp19;ext2;bves;trim10;ahnak;ush1c;msi1 |
| multicellular organismal process; | 406#3822 | 1.56E-28 | naglu;slc8a1;mertk;tead4;cfb;myo3a;ccr4;sema4c;tph1;baat;mst1;pick1;pth;c1qc;adamts13;dsg4;trpv2;ttn;smpd1;mov10l1;oprd1;mesdc2;bdnf;lhx3;tlr9;rbp2;lfng;epb41;nr2e1;notch1;c4a;cldn14;myod1;fzd6;il27ra;cdh23;masp1;glis2;hoxc10;cav2;mog;vsx1;mef2b;mmp7;gpr56;ednra;gnpat;sox14;strn4;myh4;nptn;nudc;edn1;gfra3;myog;cd2;zar1;thbd;htr3a;bbs10;nkx2-5;dock7;sbf2;crybb1;spata2;tnn;cplx1;acsl1;bcar1;pou1f1;ptafr;dip2a;lrp1;fez2;rtp3;edf1;canx;cherp;plxnb2;tnc;sox9;hexb;pbx4;pag1;mfng;unc13b;rho;ppef2;slc26a3;ace;dhrs3;lhcgr;boc;cd47;scn11a;wnt8b;coch;snai1;tcf15;smo;gcgr;tpd52;dbn1;foxp3;olfm1;chrm1;adora2b;rbp4;rpgrip1;nck1;ddx1;soat1;aqp1;dyrk3;traf6;vwf;timp1;aldh5a1;tuft1;pax3;sstr2;dscam;ntf3;fgf1;naip;snca;tbxas1;arg2;kcnma1;stx1b;prph2;grhpr;mc1r;emp3;gpr45;zscan2;prkca;myl3;txndc8;pitpnb;c8b;atp1a2;sfxn1;chrdl2;b3gnt2;col12a1;ebi3;actb;stat3;gad2;avil;prpf3;dag1;spata18;p2ry1;dclk1;pcdhb2;gaa;clec3a;smad5;sirt2;gdf5;npas2;cspg4;plcg1;f2rl1;clptm1;atrn;hoxd8;mmrn1;hoxb13;chl1;als2;kif3b;igsf10;tacr2;pitx2;clcn5;pdcd1;pex13;isl2;il19;wnt9a;gabrr3;ndrg4;spesp1;trim15;fut10;cald1;malt1;hoxb4;spred1;itgb7;cntnap2;mylip;cylc2;cdsn;hsd11b1;notch2;adamts14;napa;nyx;rufy3;hpcal4;mmp11;nppb;pcdhb14;wfs1;ift88;sdcbp;ntng2;cd3d;corin;pogk;foxn1;megf8;scnn1a;piwil2;nptx2;cxcl12;lamb2;tmod4;gna11;mylk2;egln1;evpl;stx1a;cxcl1;kif5a;amph;znrd1;map2k1;smarcb1;pdpn;adrb1;tcf25;pln;gata3;wnt4;st6gal2;pcolce;ifnk;il17c;pycard;bmp10;bbs4;tnni3;cplx3;sh3gl2;adcy3;cyp11a1;krt13;cfi;tmem176b;rabggta;ercc3;ncam1;wnt3a;proc;ambn;atxn3;rttn;rtn4;bmpr1a;gap43;myo15a;slc34a1;atpif1;unc45b;pou2f3;ppy;tlr6;asb1;kctd11;sema4f;btg4;lat;eya2;sh2d2a;prelp;obscn;grik5;lmo2;nod1;gprc5d;hes5;pcdhb11;avpr1a;cchcr1;ephb2;ehf;ccm2;ereg;mtnr1b;znf22;gda;acrv1;rgr;gal3st1;srf;nmur1;cryga;wwp1;pcdha1;meox1;notch4;slc22a4;scube1;angpt1;poll;ptprr;camk1;rab3d;tubb3;hsf4;rtp4;lep;mreg;hoxc8;ugcg;mef2d;sftpb;lhfpl5;aqp3;krt14;f2rl3;erg;rbp3;prrx2;dkk1;rnf111;nrcam;mkks;nanos3;cacng1;kcnq1;gypc;gdf10;crispld2;akap9;nkx2-6;barx1;nts;best1;unc5a;c1galt1;piwil1;ptprc;brca2;tbr1;plxnc1;stx2;nhej1;emp2;sfrp1;aldh3a2;ncoa6;htatip2;alg5;abcg5;tlr3;ppt1;sphk1;pcdh15;sfn;nrsn1;tnfrsf11a;lhb;ucp3;crx;shank3;ddx41;gria1;serpini1;pde3b;casp14;bard1;bmp6;gzf1;cdkn1a;fgf7;sh2b3;mfrp;chodl;msh4;emp1;kcnip1;mmp19;elavl1;angptl3;nqo1;ext2;rere;cd164;bicc1;tmprss11d;chat;bves;hap1;fgb;trim10;ahnak;nod2;ush1c;npbwr1;msi1 |
| multicellular organismal development; | 276#2299 | 1.68E-28 | naglu;tead4;ccr4;sema4c;pick1;pth;dsg4;ttn;smpd1;mov10l1;mesdc2;bdnf;lhx3;rbp2;lfng;nr2e1;notch1;myod1;fzd6;il27ra;glis2;hoxc10;cav2;mog;vsx1;mef2b;gpr56;gnpat;sox14;nptn;nudc;edn1;gfra3;myog;cd2;zar1;thbd;nkx2-5;dock7;sbf2;spata2;tnn;pou1f1;dip2a;lrp1;fez2;edf1;canx;cherp;plxnb2;tnc;sox9;hexb;pbx4;mfng;lhcgr;boc;wnt8b;snai1;tcf15;smo;tpd52;dbn1;olfm1;chrm1;adora2b;ddx1;dyrk3;timp1;aldh5a1;tuft1;pax3;dscam;ntf3;fgf1;naip;snca;mc1r;emp3;gpr45;zscan2;txndc8;pitpnb;sfxn1;chrdl2;b3gnt2;col12a1;stat3;avil;spata18;dclk1;pcdhb2;gaa;clec3a;smad5;gdf5;sirt2;npas2;cspg4;plcg1;clptm1;hoxd8;atrn;hoxb13;als2;chl1;kif3b;igsf10;pitx2;pdcd1;pex13;isl2;wnt9a;ndrg4;spesp1;trim15;fut10;hoxb4;spred1;itgb7;mylip;cylc2;cdsn;hsd11b1;notch2;napa;rufy3;hpcal4;mmp11;nppb;pcdhb14;wfs1;ift88;ntng2;cd3d;pogk;megf8;foxn1;piwil2;gna11;egln1;evpl;cxcl1;znrd1;map2k1;smarcb1;pdpn;adrb1;pln;tcf25;wnt4;gata3;st6gal2;pcolce;il17c;bmp10;tnni3;sh3gl2;cyp11a1;krt13;tmem176b;ercc3;wnt3a;ambn;atxn3;rttn;rtn4;bmpr1a;gap43;atpif1;unc45b;pou2f3;asb1;sema4f;kctd11;btg4;eya2;sh2d2a;obscn;prelp;lmo2;hes5;pcdhb11;cchcr1;ephb2;ccm2;ehf;ereg;znf22;gda;acrv1;gal3st1;srf;cryga;wwp1;pcdha1;meox1;notch4;scube1;poll;ptprr;angpt1;camk1;rab3d;tubb3;hsf4;mreg;lep;hoxc8;mef2d;ugcg;sftpb;lhfpl5;krt14;erg;prrx2;dkk1;rnf111;nrcam;mkks;nanos3;gypc;gdf10;nkx2-6;crispld2;barx1;nts;piwil1;c1galt1;unc5a;ptprc;brca2;tbr1;plxnc1;stx2;nhej1;emp2;sfrp1;ncoa6;aldh3a2;htatip2;alg5;tlr3;ppt1;sphk1;nrsn1;sfn;tnfrsf11a;lhb;ddx41;shank3;crx;pde3b;serpini1;casp14;bmp6;gzf1;fgf7;sh2b3;mfrp;chodl;msh4;emp1;elavl1;angptl3;mmp19;ext2;rere;cd164;bicc1;bves;hap1;trim10;ahnak;ush1c;msi1 |
| cellular process; | 1487#19591 | 7.7E-28 | sacs;fadd;prickle2;naglu;map3k12;fbxo10;usp30;znf648;myo3a;psmc2;znf566;lrrfip2;tbrg1;baat;abca12;lrp10;pth;l3mbtl2;adamts13;dsg4;znf187;zfpm2;psmf1;oprd1;znf644;tec;plek;dak;pdpr;arih1;dsg2;phkg2;myod1;fzd6;il27ra;glis2;pstk;hoxc10;cav2;vsx1;mmp7;dtd1;gnpat;sox14;endog;wdr5b;sept3;mapk10;mael;rfc3;edn1;myog;tomm40;psenen;cpb2;fmo4;gas8;dmtf1;taf1b;pnrc2;gckr;dhodh;cntrob;cdh16;rpl8;cd33;bcar1;mc3r;pou1f1;pdzd3;fez2;pdia6;ccna1;ctnnbl1;dhx35;tomm34;tnc;snx27;tbc1d23;mtmr2;pbx4;rapgef2;tmed10;gprc5a;dhrs3;lhcgr;boc;cd47;scn11a;pkd2l1;snai1;bcar3;pik3r2;fbxl7;dbn1;ddx1;ccr5;man2b2;dyrk3;fibcd1;slc25a1;col5a1;bcl9;rrbp1;spag6;gpr22;gck;mocs3;st6galnac1;b3gat1;dscam;reck;fabp6;znf280d;rpl6;tbxas1;arg2;kcnma1;asgr2;stk38l;tom1;ly6d;zbtb32;l2hgdh;gk;txndc8;rab17;pglyrp1;cacnb4;kiss1r;gadd45a;glt25d2;sf3a1;actb;rps5;avil;prpf3;akap7;tram1;dlg1;prdm15;dalrd3;fancb;znf629;spata18;cbfa2t2;cabp2;pcdhb2;arid4b;lepre1;mlxipl;smad5;sirt2;pomgnt1;mycl1;ralgps2;cetn3;usp7;gbx1;chl1;znf576;duox2;jazf1;osbpl2;trim35;tacr2;wwp2;gpr158;igfbp2;amdhd2;clec11a;cks2;ide;il19;arpc3;znf713;grin2c;gabrr3;socs7;fut10;proca1;islr;hoxb4;spred1;itgb7;darc;hmgcl;fblim1;mrpl9;tbc1d17;sept14;tsr2;notch2;trip11;bcl2a1;utp18;znf81;fam83d;mapk8ip2;pds5b;med6;clpx;lypla1;usp53;znf672;ift88;nfasc;nek10;aaas;prepl;mrpl30;zfp37;lyzl1;ms4a2;znf212;gna15;cpa5;fkbp10;piwil2;gys2;upp2;pdzk1;ppp1r13b;ca3;gna11;yaf2;tpp2;ttf1;stx1a;cntnap4;amph;map2k1;pdpn;rif1;tcf25;fth1;nmb;atp10a;pofut2;asb16;ddc;znf683;coro1a;il17c;hibadh;bmp10;pycard;ulk3;tnni3;znf624;loxl1;rassf4;a4galt;cyp11a1;gtf2h2;nrip2;manba;atg7;lrsam1;znf250;cpsf1;usp46;rtn4;traf1;sp2;dyrk2;znf202;gap43;slc34a1;ndufb2;tlr6;mthfd1;il18rap;srpk2;gcnt3;pcdhga5;lat;grik5;gucy2c;tubgcp6;nod1;gprc5d;pcdhb11;ccm2;tubg1;znf22;lrp1b;pdcd10;ripk1;osbpl5;fcgbp;slc3a1;tfeb;nkx1-2;ltb4r2;tsn;prkacb;polr2f;phf20;mcfd2;serinc2;hs3st3b1;rbm15;zkscan1;rtp4;retnlb;lep;nudt1;tbc1d1;krt14;usp2;papolb;atp5g2;stk33;fcrla;nrcam;rnase6;taok3;ipo8;lyg2;sae1;akap9;nkx2-6;trim32;nts;txk;qtrtd1;parp1;unc5a;c1galt1;dgkg;ptprc;isy1;hspd1;rsad1;foxd2;lamb4;emp2;htatip2;alg5;spc25;pcdh15;paip2b;tnfrsf11a;fyb;arcn1;ttyh1;suv39h1;rcbtb1;pacs2;pde3b;yeats4;tep1;rasgrp3;arf4;prpsap1;scap;trmt6;dnajc13;fgf7;sh2b3;mlh3;sdad1;alb;atg12;mdm2;pnpla8;ccdc59;icam4;nqo1;napg;dhps;caskin1;mtfmt;chat;sycp3;hap1;mgat2;gosr1;ly96;mcm6;dusp12;sardh;zfp62;slc8a1;mertk;rnf34;b3gnt3;sod3;ears2;stk25;psen1;ndc80;arid1b;rpl28;dnah3;epha7;cilp;ttn;calcoco1;rps9;vps72;afmid;sytl3;rars;tlr9;rpl12;epb41;asf1a;cldn14;ublcp1;cdh23;tbc1d2;prmt2;ncoa3;coq3;tk1;myh4;strn4;ptpn4;chpt1;me3;cxadr;cd2;ptgir;actn2;bbs10;napsa;apc;spata2;ecd;dnaja1;smg5;kif26b;gpr4;lrp1;spc24;foxj3;ppm1f;txnl4b;supt3h;fbxo43;tia1;canx;arntl2;cpt1b;sox9;pag1;mrpl24;psd3;etv2;pla2g12b;cd320;usp48;map2;wnt8b;recql;rap2a;rps3a;irf2;dlg3;uevld;phb2;gtf2e1;gsk3b;nck1;soat1;cd69;lias;thoc3;cdkl3;pitpnm3;vwf;aldh5a1;znf641;pax3;clasp1;cacna1i;coro2b;dnajc17;tagap;gpr142;astl;galnt5;cytl1;hivep1;ulk2;lcat;b3galt5;gucy1a2;prkca;itgal;clgn;smarcad1;znf667;ppbp;sfxn1;znf175;rin2;ebi3;anxa3;tmem48;dag1;sohlh2;il18r1;ect2;pex19;dpf3;e2f6;gdf5;cspg4;plcg1;fanca;clptm1;ighmbp2;mmrn1;atrip;ube2d3;med18;nrbf2;kif3b;pdcd1;mrps5;isl2;ndrg4;mus81;trim15;qdpr;ptp4a3;malt1;pkp2;idi1;mrpl49;nek9;ndufs8;cdc37l1;nphp4;ctnna3;p2rx4;tbc1d10b;adamts14;akt1s1;osbpl7;bcl2l13;aurkb;pop1;nppb;adprhl1;pcdhga10;atp6v1a;ccr9;tbk1;cpd;foxn1;sdk1;qars;znf35;nptx2;cxcl12;ly6e;ilkap;znf23;ppp1r2;nr0b2;rab35;tert;noto;chst3;ldha;grk4;sptlc1;cd6;sh3gl2;tmprss12;rgl3;smarcd2;crnkl1;rps6kb2;psat1;arhgap5;cfi;ercc3;irak2;proc;parp4;rfx2;rbck1;sar1a;gpr31;pisd;bmpr1a;atp6v0b;prpf4b;znf16;vprbp;hnrnpl;yes1;atpif1;znf569;pou2f3;dapk2;rnf149;ppy;nup210;skiv2l2;asb1;sema4f;btg4;rpap1;eya2;gtf2h5;atp5b;rps15a;znf770;rfc5;hes5;ccl19;iqgap1;rnf216;ephb2;sh3bp4;mtnr1b;zmiz2;klf8;gng12;atp5f1;msh5;znf238;kif13b;pgk2;nfyc;shprh;tom1l2;cx3cr1;homer3;hivep2;rpl7l1;pkn3;meox1;eif2c3;abtb1;ptprr;poll;tfdp2;pars2;prss22;elovl7;aire;eif5a;sirt6;mef2d;lhfpl5;ppp1r1b;rbp3;erg;arhgef5;dnase1l2;ubqlnl;snx21;dkk1;prrx2;rnf111;pou5f1;lonrf2;nanos3;sds;abca7;gypc;nfkbie;gdf10;pfkp;esco2;grb10;krt7;naaladl1;efcab6;tigd2;rasgef1a;nup160;plch2;brca2;elp4;ncf1;usp29;tpx2;sec11a;pigb;exosc8;rassf8;pcdhgb6;ppt1;cdc25b;nup98;pold2;parvb;trim23;crx;shank3;ddx41;slco4a1;nr1h3;dchs2;gan;ehd1;immp1l;atg3;psma6;bik;polr3d;stxbp1;mapk8ip1;tnfrsf21;casp14;apba3;tmprss9;slc25a37;klrc2;gpr114;col8a2;nab1;acads;snrpd3;mat2a;thnsl1;mmp19;dmgdh;ext2;sept12;pdap1;cd164;kif23;nars;gsta1;gmps;rassf3;wdr19;pigq;exosc10;galnt1;tead4;cfb;pla2g12a;rab18;timm50;dld;ccr4;arsb;gphn;gldc;sema4c;furin;tph1;drg2;mst1;farsb;znf300;il15ra;znf275;larp7;ptgis;smpd1;mov10l1;atp6v1e2;pcdhga8;suclg1;hal;rbp2;psmb6;nr2e1;rxrg;notch1;c4a;clec1a;poln;pop5;eif4ebp1;masp1;leo1;lypla2;dnhd1;csk;vgll3;rad54b;mog;gpsm2;mef2b;gpr56;arhgef1;ccr3;hars2;ly9;zdhhc1;mettl4;nudc;ung;gfra3;pou5f2;sos1;znf32;aff4;nr1d1;dock7;upf1;acsl1;pnliprp3;ptafr;rarres2;setd7;socs6;rtp3;ppp2r2d;arpc1b;rdbp;edf1;vav2;cherp;scrn3;arfip2;mars;zdhhc17;ccna2;rasa2;nr4a1;rho;adck1;ace;insrr;slc25a36;rps14;ndufb5;tcf15;gcgr;dsn1;smo;ak3;crnn;tpd52;foxp3;gnb1;adora2b;itga3;phf17;chrm1;fgd5;plod3;traf6;mrpl43;asb17;pqbp1;irf5;sdf2;hsd17b11;pes1;diras1;sstr2;flt3;ntf3;col27a1;naip;snca;fkbp2;cdc23;stx1b;cldn17;fdft1;prph2;tcf23;mc1r;stx11;phkg1;emp3;morf4l1;adam7;gpr45;zscan2;flrt2;suv420h1;mms19;cd97;pcbp2;sec61a1;kdelr2;chrdl2;adam21;col12a1;stard5;capns1;stat3;atg4c;vps54;gad2;sept2;brms1l;mad2l1;limk2;padi6;p2ry1;npas2;mapkapk2;f2rl1;kif1a;hoxd8;usp10;mmp24;als2;sf3b3;mrpl17;igsf10;clcn5;pex13;klhl3;gtf2i;mcm10;mcf2l;pabpc1;tlr5;prpf40a;stx18;ift57;adipoq;sox13;taf1a;cald1;dr1;gga1;ccar1;adam33;znf329;pla2g4b;ppfibp2;cdsn;znf555;ssbp2;lifr;hsd11b1;mast3;bub1b;hk1;trmu;rufy3;mmp11;ltc4s;sphar;pcdhb14;shroom1;rpl5;tbc1d24;sdcbp;lars;atp1a1;ntng2;corin;pogk;slc20a1;ccl27;lamb2;epha5;gpaa1;egln1;foxr1;sh3rf2;znf34;epn2;sult1c2;cxcl1;hipk4;rxfp1;kif5a;tox3;ppm1g;znrd1;trip10;taok2;sgms2;gata3;wnt4;sart3;znf470;ifnk;rab8b;rapgef1;gpr20;hp;znf142;gpr84;fen1;xrn1;zcchc2;adcy3;tmem176b;rabggta;wnt3a;ndufb7;vamp4;aldob;dph3;tbx10;kif27;rgs1;il22ra2;kiaa1109;tsen2;epb41l2;pbrm1;unc45b;stoml3;mapk8ip3;shc2;dmrtb1;cdkl2;klk9;guk1;avpr1a;ehf;tasp1;ereg;cct6b;ik;mfge8;lipc;pxn;ap4s1;sptbn2;bckdhb;cks1b;mpdu1;srf;abhd5;uap1;nmur1;alpk1;wwp1;notch4;scube1;elavl2;pvrl3;rad21;hsf4;dio1;ftcd;rpp14;l3mbtl3;stx16;alas1;ube3b;foxd4l1;psip1;limch1;stambp;map2k2;ubl7;pfdn1;siva1;cd19;aoc3;ezh1;hat1;rps23;piwil1;ptprh;ctnnal1;use1;tob2;cercam;tbr1;zhx2;nedd9;lrp3;api5;aldh3a2;mto1;ephb6;pigs;nfatc1;sphk1;ssh1;ssh2;lhb;rffl;trim28;trappc1;ucp3;dhx38;iscu;gria1;polr3g;cdkn1a;lrrk2;pvrl4;eif4g3;smarce1;kcnip1;elavl1;ppig;sec13;aspm;arhgap28;zbtb7a;cry2;ppme1;lgmn;impa1;fgb;atm;nod2;lsm10;adamts16;trex1;ptk2;pde1a;acaca;ppil3;snrpa;izumo1;fmod;trim29;cd96;adamts17;il1rl2;fignl1;pick1;clk4;c1qc;anxa9;itpka;glyctk;npffr1;mdc1;rbms2;rce1;lhx3;taf5;nkiras2;znf181;casc5;wdr33;rasal1;mccc1;ptpn18;rgs8;nom1;tpt1;sept11;zbtb5;capg;krt8;rims4;slc9a3;fbxo15;ednra;brca1;rhov;nptn;pcdhgb5;usp35;cdc25c;sp100;sult1b1;cecr2;htr3a;nkx2-5;sbf2;pfdn5;mtmr7;gne;tnn;cd53;casp8;cplx1;htr5a;skap1;psmb4;znf532;carhsp1;nek3;dsc1;pik3c2a;stard13;pcdhga1;gal3st3;nfyb;znf529;plxnb2;bcl11b;hexb;anp32a;sec24a;unc13b;plcb3;ppef2;ca6;fbxl3;rfc1;slc25a6;prkar2b;sec61g;wdr7;gfpt2;ing4;arl8b;nr1h4;clcn6;rpl18a;rab38;cdc73;pcdhgb4;cpt1c;il15;kiss1;timp1;anapc11;slc34a3;msc;serpina5;gas2l3;fgf1;spcs3;fxc1;ubqln4;ecm1;hsd17b12;dbp;thrsp;wbp2nl;rps6kb1;sertad3;asgr1;stk24;cpa4;fbxw11;lzts1;senp3;rpl39;c8b;atp1a2;il16;znf584;ap1s3;nudt21;dgki;b3gnt2;ftsj2;mbtps1;otud7b;vps41;dynlrb2;tle4;snap29;znf710;c1s;dst;csnk1g2;dclk1;mycbp2;ttll2;hsd17b2;gaa;zkscan5;usp26;pank4;ncoa1;ppm1m;nup153;mapk14;rpp38;gpr85;cd37;hoxb13;txnrd3;pitx2;fut11;wnt9a;pola2;mphosph6;steap3;cntnap2;kif18a;mylip;cylc2;med11;ptpn3;acaa2;strn3;gtf3a;pum2;napa;rbm17;znf646;plcd4;il12rb1;usp45;pigz;irak1;msh2;mrpl11;gpr171;trim13;idua;mapre1;acot9;sgpp1;ric8b;rhobtb2;alg2;cd3d;pcdhga11;gk2;znf524;lonrf3;gcg;dusp7;sept7;mylk2;klk11;icam5;evpl;chmp1a;fkbp5;znf345;lnx1;grhl3;rasd1;arhgap25;rad52;dok1;smarcb1;brd8;arl6ip1;fbxo8;aarsd1;adamts8;adrb1;map3k1;herc1;pln;urm1;prss21;vrk1;st6gal2;znf688;znf395;casp8ap2;nr4a3;vapb;bbs4;hdgf;tmlhe;cenpj;usp49;rabgap1;cideb;aven;gpr141;cplx3;krt4;rexo2;zbtb3;armc10;ptpn1;epdr1;rab7l1;rpl23;ncam1;gabpa;tceb1;ube2b;atxn3;uimc1;mmp25;katnb1;zcchc4;cetn1;fut3;txn;eif4a1;pias4;mat2b;mif4gd;acsl5;kctd11;jdp2;psen2;gcnt4;sh2d2a;dnajb5;lcmt1;cda;obscn;cchcr1;ilf3;gda;clp1;rgr;rbak;gal3st1;nat6;faslg;slu7;pcgf5;dis3;pcdha1;angpt1;gopc;flad1;sgsm3;fabp3;camk1;rab3d;pnpt1;oasl;tubb3;snapc4;dmwd;rgs22;mreg;ugcg;hoxc8;dctd;sftpb;cidec;crbn;cdh26;f2rl3;gba3;foxn2;exosc7;mctp2;mkks;zmat5;agpat4;gpr15;rplp1;barx1;prmt7;pcmtd1;cask;rab19;hmgcs1;slc27a3;cog2;terf2;plxnc1;mgea5;stx2;nhej1;eef1a2;gng8;sfrp1;ncoa6;chek1;capn10;ccng1;phlda1;tlr3;gpbar1;sfn;nid1;pou2f2;hnrnpr;atf2;sgk2;med24;cldn4;bard1;klk14;vipr2;bmp6;gzf1;ulk4;smurf2;rps27;msh4;emp1;plcl2;angptl3;wdr67;got1;rere;tmprss11d;galt;mrps14;sec31a;atf5;etv1;ush1c;npbwr1;ssbp1 |
| multicellular organismal development#system development; | 205#1605 | 4.93E-25 | naglu;ntng2;cd3d;tead4;foxn1;ccr4;sema4c;gna11;egln1;evpl;pick1;pth;dsg4;ttn;cxcl1;smpd1;znrd1;map2k1;mesdc2;pdpn;bdnf;tcf25;lhx3;pln;gata3;rbp2;lfng;nr2e1;notch1;myod1;fzd6;il27ra;glis2;il17c;bmp10;cav2;mog;tnni3;mef2b;gpr56;sh3gl2;gnpat;sox14;cyp11a1;krt13;nptn;edn1;tmem176b;gfra3;ercc3;myog;wnt3a;cd2;ambn;atxn3;rtn4;nkx2-5;dock7;sbf2;bmpr1a;tnn;gap43;pou1f1;atpif1;unc45b;pou2f3;fez2;asb1;kctd11;sema4f;btg4;eya2;canx;cherp;sh2d2a;prelp;tnc;sox9;hexb;pbx4;hes5;pcdhb11;lhcgr;boc;ephb2;wnt8b;ccm2;snai1;tcf15;ereg;znf22;smo;gda;gal3st1;dbn1;tpd52;olfm1;srf;adora2b;chrm1;cryga;wwp1;dyrk3;pcdha1;notch4;scube1;angpt1;poll;timp1;camk1;rab3d;aldh5a1;tuft1;tubb3;pax3;hsf4;dscam;ntf3;lep;mreg;fgf1;ugcg;mef2d;hoxc8;naip;snca;sftpb;lhfpl5;krt14;nrcam;gpr45;mkks;gypc;gdf10;crispld2;sfxn1;nkx2-6;chrdl2;barx1;b3gnt2;col12a1;stat3;c1galt1;avil;ptprc;brca2;tbr1;dclk1;pcdhb2;stx2;nhej1;gaa;clec3a;smad5;sirt2;aldh3a2;npas2;ncoa6;cspg4;htatip2;tlr3;ppt1;sphk1;hoxb13;sfn;als2;chl1;nrsn1;tnfrsf11a;lhb;crx;igsf10;pitx2;pex13;serpini1;pde3b;trim15;fut10;casp14;bmp6;gzf1;fgf7;sh2b3;mylip;chodl;msh4;emp1;cdsn;mmp19;angptl3;hsd11b1;ext2;notch2;napa;bves;rufy3;hpcal4;trim10;nppb;ahnak;pcdhb14;ush1c;wfs1;ift88;msi1 |
| negative regulation of biological process; | 163#1182 | 2.34E-24 | mapre1;psen1;furin;tbrg1;ppp1r13b;yaf2;chmp1a;ttf1;znf345;cxcl1;ilkap;ppm1g;vps72;smarcb1;nr0b2;adamts8;adrb1;tcf25;pln;fth1;ifnk;notch1;rgs8;il27ra;eif4ebp1;tpt1;glis2;csk;bmp10;pycard;capg;cav2;aven;krt4;xrn1;rassf4;brca1;edn1;gas8;proc;rtn4;dph3;uimc1;apc;rgs1;nkx2-5;katnb1;znf202;cd33;pou1f1;atpif1;pdzd3;pias4;socs6;asb1;kctd11;stard13;btg4;cda;sox9;hes5;rps14;ilf3;map2;ephb2;ereg;smo;irf2;ing4;znf238;dlg3;sptbn2;pik3r2;foxp3;phb2;nr1h4;gsk3b;nat6;phf17;cdc73;wwp1;notch4;sgsm3;gopc;timp1;fabp3;sstr2;gck;clasp1;reck;ntf3;serpina5;gas2l3;fabp6;lep;sirt6;rgs22;naip;snca;dkk1;emp3;stambp;zbtb32;prkca;sertad3;taok3;nfkbie;lzts1;kiss1r;barx1;gadd45a;otud7b;stat3;avil;ptprc;dlg1;tob2;brca2;zhx2;dst;terf2;e2f6;lepre1;eef1a2;sirt2;sfrp1;api5;htatip2;chek1;tlr3;ppt1;sphk1;paip2b;jazf1;trim35;socs7;adipoq;bard1;scap;gzf1;malt1;cdkn1a;smurf2;dr1;alb;mdm2;smarce1;nab1;ext2;notch2;akt1s1;zbtb7a;cd164;bcl2a1;bub1b;mapk8ip2;pds5b;sycp3;msh2;nppb;atm;atf5;trim13 |
| negative regulation of cellular process; | 158#1137 | 4.19E-24 | mapre1;psen1;furin;tbrg1;ppp1r13b;yaf2;chmp1a;ttf1;znf345;cxcl1;ilkap;ppm1g;vps72;smarcb1;nr0b2;adamts8;tcf25;fth1;ifnk;notch1;rgs8;eif4ebp1;tpt1;glis2;csk;bmp10;pycard;capg;cav2;aven;krt4;xrn1;rassf4;brca1;edn1;gas8;proc;rtn4;uimc1;apc;rgs1;nkx2-5;katnb1;znf202;cd33;atpif1;pou1f1;pdzd3;pias4;socs6;asb1;kctd11;stard13;btg4;cda;sox9;hes5;rps14;ilf3;map2;ephb2;ereg;smo;irf2;ing4;znf238;dlg3;sptbn2;pik3r2;foxp3;phb2;nr1h4;gsk3b;nat6;phf17;cdc73;wwp1;notch4;sgsm3;gopc;timp1;fabp3;sstr2;gck;clasp1;reck;ntf3;serpina5;gas2l3;fabp6;sirt6;rgs22;naip;snca;dkk1;emp3;stambp;zbtb32;prkca;sertad3;taok3;nfkbie;lzts1;kiss1r;barx1;gadd45a;otud7b;stat3;avil;dlg1;ptprc;tob2;brca2;zhx2;dst;terf2;e2f6;lepre1;eef1a2;sirt2;sfrp1;api5;htatip2;chek1;tlr3;ppt1;sphk1;paip2b;jazf1;trim35;socs7;adipoq;bard1;scap;gzf1;malt1;cdkn1a;smurf2;dr1;alb;mdm2;smarce1;nab1;ext2;notch2;akt1s1;zbtb7a;cd164;bcl2a1;bub1b;mapk8ip2;pds5b;sycp3;msh2;nppb;atm;atf5;trim13 |
| biological regulation; | 602#6731 | 5.4E-21 | fadd;tead4;cfb;rab18;znf648;znf566;dld;ccr4;furin;tbrg1;tph1;abca12;mst1;znf300;pth;l3mbtl2;adamts13;znf275;znf187;zfpm2;znf644;nr2e1;notch1;rxrg;c4a;myod1;il27ra;eif4ebp1;masp1;glis2;pstk;hoxc10;leo1;csk;cav2;vgll3;rad54b;vsx1;mef2b;ccr3;arhgef1;sox14;mael;edn1;pou5f2;sos1;myog;znf32;psenen;aff4;gas8;dmtf1;taf1b;nr1d1;pnrc2;upf1;gckr;cd33;bcar1;pou1f1;slc9a1;pdzd3;pdia6;socs6;ccna1;rdbp;edf1;vav2;tbc1d23;pbx4;zdhhc17;rapgef2;ccna2;nr4a1;rasa2;ace;boc;cd47;rps14;tcf15;smo;gcgr;pik3r2;dbn1;foxp3;chrm1;phf17;adora2b;fgd5;ddx1;traf6;tkt;pqbp1;irf5;gck;sstr2;flt3;reck;ntf3;fabp6;znf280d;naip;snca;rpl6;tbxas1;arg2;kcnma1;cdc23;stk38l;tcf23;emp3;morf4l1;zscan2;zbtb32;suv420h1;mms19;txndc8;cacnb4;kiss1r;gadd45a;capns1;stat3;gad2;avil;prdm15;dlg1;znf629;brms1l;mad2l1;cbfa2t2;p2ry1;arid4b;lepre1;mlxipl;smad5;sirt2;mycl1;npas2;f2rl1;gbx1;hoxd8;als2;znf576;jazf1;trim35;igfbp2;clec11a;cks2;il19;arpc3;znf713;gtf2i;socs7;mcf2l;pabpc1;adipoq;ift57;sox13;taf1a;hoxb4;spred1;dr1;fblim1;znf329;znf555;ssbp2;lifr;tbc1d17;notch2;bcl2a1;znf81;bub1b;mapk8ip2;pds5b;med6;znf672;ift88;tbc1d24;atp1a1;aaas;zfp37;corin;znf212;pogk;gna15;slc20a1;gna11;ppp1r13b;yaf2;egln1;foxr1;ttf1;stx1a;znf34;cxcl1;tox3;znrd1;ppm1g;taok2;pdpn;tcf25;gata3;fth1;atp10a;ifnk;znf470;rapgef1;rab8b;znf683;hp;bmp10;pycard;tnni3;znf142;xrn1;znf624;rassf4;gtf2h2;atg7;znf250;wnt3a;rtn4;traf1;tbx10;dph3;sp2;rgs1;dyrk2;il22ra2;znf202;gap43;pbrm1;slc34a1;tlr6;lat;als2cr12;mapk8ip3;dmrtb1;nod1;avpr1a;ehf;ereg;znf22;sptbn2;cks1b;ripk1;srf;nmur1;tfeb;wwp1;nkx1-2;notch4;slc22a4;scube1;elavl2;ltb4r2;polr2f;phf20;serinc2;rbm15;zkscan1;hsf4;lep;l3mbtl3;tbc1d1;psip1;foxd4l1;nrcam;stambp;taok3;kcnq1;nkx2-6;siva1;nts;ezh1;dgkg;ptprc;tob2;hspd1;foxd2;tbr1;zhx2;nedd9;api5;htatip2;nfatc1;sphk1;pcdh15;paip2b;tnfrsf11a;trim28;suv39h1;rcbtb1;polr3g;pde3b;yeats4;rasgrp3;scap;trmt6;cdkn1a;fgf7;alb;eif4g3;mdm2;ccdc59;smarce1;elavl1;napg;zbtb7a;dhps;cry2;chat;sycp3;fgb;atm;nod2;mcm6;zfp62;psen1;arid1b;pick1;c1qc;ttn;vps72;lhx3;taf5;tlr9;znf181;asf1a;rasal1;rgs8;cdh23;tpt1;tbc1d2;zbtb5;capg;ncoa3;slc9a3;ednra;brca1;nptn;chpt1;cdc25c;cd2;sp100;thbd;actn2;apc;nkx2-5;sbf2;pfdn5;ecd;tnn;casp8;smg5;znf532;carhsp1;foxj3;supt3h;tia1;stard13;nfyb;znf529;arntl2;bcl11b;sox9;hexb;pag1;psd3;unc13b;etv2;cd320;rfc1;map2;rps3a;irf2;ing4;dlg3;uevld;nr1h4;phb2;gsk3b;clcn6;gtf2e1;cdc73;nck1;il15;timp1;vwf;aldh5a1;znf641;pax3;slc34a3;clasp1;msc;serpina5;gas2l3;ecm1;hivep1;dbp;thrsp;prkca;smarcad1;sertad3;lzts1;znf667;c8b;atp1a2;il16;znf584;dgki;znf175;ebi3;mbtps1;otud7b;tle4;sohlh2;ect2;znf710;dst;dpf3;e2f6;mycbp2;gdf5;zkscan5;ncoa1;ighmbp2;mmrn1;atrip;hoxb13;ube2d3;med18;nrbf2;txnrd3;pitx2;isl2;ndrg4;malt1;med11;gtf3a;tbc1d10b;akt1s1;pum2;znf646;il12rb1;bcl2l13;irak1;msh2;nppb;ccr9;trim13;tbk1;mapre1;ric8b;foxn1;znf524;znf35;cxcl12;chmp1a;znf345;grhl3;ilkap;ppp1r2;znf23;smarcb1;brd8;fbxo8;nr0b2;rab35;adamts8;adrb1;pln;noto;grk4;znf688;znf395;casp8ap2;nr4a3;vapb;rabgap1;cideb;cplx3;krt4;aven;zbtb3;armc10;smarcd2;rgl3;pkia;arhgap5;cfi;ercc3;cav1;gabpa;proc;tceb1;rfx2;ube2b;atxn3;uimc1;bmpr1a;katnb1;znf16;znf569;atpif1;pou2f3;dapk2;txn;pias4;asb1;kctd11;btg4;jdp2;psen2;eya2;obscn;cda;gtf2h5;znf770;ccl19;hes5;iqgap1;ephb2;ilf3;zmiz2;cutc;klf8;znf238;rbak;gal3st1;nat6;faslg;nfyc;pcgf5;hivep2;meox1;sgsm3;gopc;tfdp2;fabp3;rab3d;snapc4;aire;eif5a;sirt6;rgs22;mef2d;hoxc8;sftpb;cidec;f2rl3;erg;arhgef5;prrx2;foxn2;dkk1;pou5f1;mkks;nanos3;nfkbie;barx1;prmt7;krt7;tigd2;efcab6;rasgef1a;brca2;terf2;elp4;nhej1;eef1a2;mall;sfrp1;ncoa6;chek1;ccng1;tlr3;ppt1;cdc25b;sfn;crx;nr1h3;pou2f2;atf2;med24;bik;mapk8ip1;bard1;bmp6;gzf1;smurf2;emp1;nab1;angptl3;wdr67;ext2;rere;cd164;etv1;atf5;ush1c;npbwr1 |
| cellular developmental process; | 212#1810 | 6.41E-20 | fadd;sgpp1;ntng2;cd3d;rnf34;foxn1;piwil2;ccr4;psen1;sema4c;ppp1r13b;gna11;evpl;pick1;pth;dsg4;ttn;mov10l1;znrd1;map2k1;taok2;smarcb1;adrb1;gata3;casc5;notch1;myod1;tpt1;glis2;il17c;casp8ap2;pycard;cav2;tnni3;cideb;aven;krt4;brca1;cyp11a1;nptn;edn1;tmem176b;ercc3;myog;wnt3a;cd2;proc;parp4;ube2b;atxn3;actn2;cecr2;rtn4;traf1;nkx2-5;dock7;spata2;bmpr1a;dyrk2;tnn;casp8;gap43;unc45b;bcar1;dapk2;fez2;ppm1f;kctd11;sema4f;tia1;srpk2;ctnnbl1;edf1;btg4;eya2;psen2;sh2d2a;tnc;obscn;sox9;unc13b;nod1;hes5;boc;rnf216;cchcr1;ehf;slc25a6;ccm2;ereg;rps3a;smo;pdcd10;ing4;sptbn2;pik3r2;dbn1;tpd52;ripk1;gsk3b;phf17;faslg;dyrk3;traf6;notch4;scube1;spag6;angpt1;timp1;camk1;rab3d;rad21;tubb3;pax3;hsf4;ntf3;eif5a;lep;mreg;fgf1;hoxc8;naip;snca;cidec;lhfpl5;krt14;fcrla;nrcam;emp3;stambp;zscan2;prkca;nanos3;mkks;txndc8;c8b;sfxn1;siva1;chrdl2;barx1;b3gnt2;gadd45a;unc5a;c1galt1;piwil1;ctnnal1;ptprc;hspd1;brca2;spata18;dclk1;stx2;nhej1;eef1a2;smad5;gdf5;emp2;sfrp1;api5;ncoa6;cspg4;htatip2;clptm1;phlda1;tlr3;ppt1;sphk1;sfn;als2;chl1;rffl;trim35;trim28;crx;ddx41;igsf10;suv39h1;pdcd1;pacs2;pex13;il19;bik;ndrg4;trim15;casp14;ift57;bard1;tnfrsf21;bmp6;malt1;cdkn1a;steap3;sh2b3;cylc2;alb;atg12;ccar1;emp1;cdsn;mmp19;notch2;akt1s1;napa;bcl2a1;bub1b;mapk8ip2;sycp3;bcl2l13;rufy3;atf5;nod2;ush1c |
| cell differentiation; | 212#1810 | 6.41E-20 | fadd;sgpp1;ntng2;cd3d;rnf34;foxn1;piwil2;ccr4;psen1;sema4c;ppp1r13b;gna11;evpl;pick1;pth;dsg4;ttn;mov10l1;znrd1;map2k1;taok2;smarcb1;adrb1;gata3;casc5;notch1;myod1;tpt1;glis2;il17c;casp8ap2;pycard;cav2;tnni3;cideb;aven;krt4;brca1;cyp11a1;nptn;edn1;tmem176b;ercc3;myog;wnt3a;cd2;proc;parp4;ube2b;atxn3;actn2;cecr2;rtn4;traf1;nkx2-5;dock7;spata2;bmpr1a;dyrk2;tnn;casp8;gap43;unc45b;bcar1;dapk2;fez2;ppm1f;kctd11;sema4f;tia1;srpk2;ctnnbl1;edf1;btg4;eya2;psen2;sh2d2a;tnc;obscn;sox9;unc13b;nod1;hes5;boc;rnf216;cchcr1;ehf;slc25a6;ccm2;ereg;rps3a;smo;pdcd10;ing4;sptbn2;pik3r2;dbn1;tpd52;ripk1;gsk3b;phf17;faslg;dyrk3;traf6;notch4;scube1;spag6;angpt1;timp1;camk1;rab3d;rad21;tubb3;pax3;hsf4;ntf3;eif5a;lep;mreg;fgf1;hoxc8;naip;snca;cidec;lhfpl5;krt14;fcrla;nrcam;emp3;stambp;zscan2;prkca;nanos3;mkks;txndc8;c8b;sfxn1;siva1;chrdl2;barx1;b3gnt2;gadd45a;unc5a;c1galt1;piwil1;ctnnal1;ptprc;hspd1;brca2;spata18;dclk1;stx2;nhej1;eef1a2;smad5;gdf5;emp2;sfrp1;api5;ncoa6;cspg4;htatip2;clptm1;phlda1;tlr3;ppt1;sphk1;sfn;als2;chl1;rffl;trim35;trim28;crx;ddx41;igsf10;suv39h1;pdcd1;pacs2;pex13;il19;bik;ndrg4;trim15;casp14;ift57;bard1;tnfrsf21;bmp6;malt1;cdkn1a;steap3;sh2b3;cylc2;alb;atg12;ccar1;emp1;cdsn;mmp19;notch2;akt1s1;napa;bcl2a1;bub1b;mapk8ip2;sycp3;bcl2l13;rufy3;atf5;nod2;ush1c |
| biological_process; | 1750#24743 | 6.95E-20 | sacs;fadd;prickle2;naglu;map3k12;fbxo10;usp30;znf648;col23a1;myo3a;slc35a5;psmc2;znf566;mfsd11;pbld;lrrfip2;tbrg1;baat;abca12;chmp7;lrp10;pth;l3mbtl2;adamts13;dsg4;trpv2;znf187;zfpm2;psmf1;oprd1;znf644;mitd1;tec;bdnf;plek;dak;pdpr;cacng7;arih1;dsg2;phkg2;myod1;fzd6;il27ra;glis2;pstk;hoxc10;cav2;vsx1;mmp7;dtd1;gnpat;sox14;endog;wdr5b;sept3;mapk10;mael;rfc3;edn1;myog;tomm40;psenen;cpb2;fmo4;gas8;dmtf1;taf1b;slc35f4;pnrc2;gckr;dhodh;cntrob;cdh16;rpl8;cd33;bcar1;mc3r;pou1f1;ccdc85b;slc9a1;pdzd3;fez2;pdia6;ccna1;ctnnbl1;dhx35;kctd18;tomm34;tnc;snx27;tbc1d23;mtmr2;pbx4;rapgef2;tmed10;gprc5a;dhrs3;lhcgr;boc;cd47;scn11a;pkd2l1;snai1;bcar3;fsd1l;pik3r2;emid2;fbxl7;c1qtnf5;dbn1;sys1;rbp4;ddx1;ccr5;man2b2;bet3l;dyrk3;fibcd1;slc25a1;col5a1;bcl9;rrbp1;spag6;gpr22;gck;mocs3;st6galnac1;b3gat1;dscam;reck;fabp6;znf280d;rpl6;tbxas1;khk;arg2;kcnma1;asgr2;stk38l;micall1;kcnj10;tom1;ly6d;zbtb32;l2hgdh;gk;txndc8;rab17;pglyrp1;cacnb4;lct;slc4a10;kiss1r;gadd45a;glt25d2;slc13a5;sf3a1;actb;rps5;cog7;avil;prpf3;akap7;tram1;dlg1;prdm15;dalrd3;fancb;znf629;spata18;cbfa2t2;cabp2;pcdhb2;arid4b;aftph;ccdc91;lepre1;mlxipl;clec3a;smad5;sirt2;pomgnt1;mycl1;slc22a16;slc39a13;ralgps2;cetn3;usp7;gbx1;rcl1;chl1;znf576;duox2;jazf1;osbpl2;trim35;tacr2;wwp2;gpr158;igfbp2;amdhd2;clec11a;cks2;ide;il19;arpc3;znf713;grin2c;gabrr3;socs7;fut10;proca1;islr;hoxb4;spred1;itgb7;darc;sdc2;hmgcl;fblim1;mrpl9;tbc1d17;sept14;tsr2;notch2;trip11;bcl2a1;utp18;znf81;fam83d;mapk8ip2;pds5b;med6;clpx;lypla1;zbp1;usp53;znf672;ift88;nfasc;nek10;aaas;prepl;mrpl30;zfp37;lyzl1;slc35b3;ms4a2;znf212;gna15;cpa5;fkbp10;piwil2;gys2;upp2;pdzk1;tmod4;ppp1r13b;ca3;gna11;yaf2;slc28a3;slc26a9;tpp2;ttf1;stx1a;cntnap4;amph;map2k1;pdpn;rif1;tcf25;casc3;fth1;nmb;atp10a;cmtm8;pofut2;asb16;dhrs7c;ddc;znf683;aanat;coro1a;il17c;hibadh;bmp10;pycard;ulk3;tnni3;slc7a14;znf624;sh3tc1;loxl1;rassf4;a4galt;cyp11a1;gtf2h2;slc6a12;nrip2;manba;atg7;lrsam1;znf250;cpsf1;usp46;rttn;rtn4;traf1;sp2;dyrk2;znf202;gap43;myo15a;slc34a1;slc25a34;ndufb2;tlr6;mthfd1;il18rap;srpk2;gcnt3;pcdhga5;lat;als2cr12;grik5;gucy2c;tubgcp6;nod1;gprc5d;gltscr2;pcdhb11;ccm2;tubg1;znf22;lrp1b;glb1l;pdcd10;ripk1;osbpl5;fcgbp;slc3a1;tfeb;nkx1-2;slc22a4;slc16a14;ltb4r2;tsn;prkacb;polr2f;phf20;mcfd2;abca3;slc12a3;serinc2;hs3st3b1;rbm15;zkscan1;rtp4;retnlb;lep;kiaa0247;nudt1;tbc1d1;krt14;usp2;papolb;tm6sf2;atp5g2;stk33;fcrla;nrcam;rnase6;taok3;kcnq1;ipo8;lyg2;sae1;akap9;nkx2-6;trim32;slc30a10;nts;txk;abcc9;qtrtd1;parp1;unc5a;c1galt1;arsi;dgkg;ptprc;isy1;hspd1;rsad1;foxd2;lamb4;emp2;htatip2;alg5;spc25;pcdh15;paip2b;tnfrsf11a;fyb;arcn1;ttyh1;slc5a9;p2rx2;suv39h1;rcbtb1;ppp1r7;pacs2;pde3b;yeats4;tep1;rasgrp3;arf4;prpsap1;scap;trmt6;dnajc13;fgf7;sh2b3;mlh3;sdad1;alb;atg12;mdm2;pnpla8;ccdc59;icam4;nqo1;napg;dhps;caskin1;crisp3;mtfmt;bicc1;chat;sycp3;hap1;mgat2;ahnak;slc19a1;tnfrsf4;gosr1;btbd10;ly96;mcm6;dusp12;sardh;il18bp;zfp62;slc8a1;mertk;rnf34;b3gnt3;sod3;ears2;stk25;psen1;ndc80;arid1b;rpl28;dnah3;epha7;scn2a;cilp;ttn;calcoco1;rps9;vps72;afmid;sytl3;rars;tlr9;rpl12;epb41;asf1a;cldn14;ublcp1;apod;cdh23;tbc1d2;prmt2;ncoa3;coq3;gfod1;tk1;strn4;myh4;ptpn4;chpt1;me3;cxadr;bloc1s1;cd2;ptgir;zar1;thbd;actn2;napsa;bbs10;cog6;apc;spata2;ecd;dnaja1;kif26b;smg5;slc1a4;gpr4;lrp1;spc24;foxj3;ppm1f;txnl4b;supt3h;fbxo43;tia1;eps8l1;canx;arntl2;syt2;cpt1b;sox9;pag1;mrpl24;psd3;etv2;ganab;slc26a3;pla2g12b;cd320;usp48;map2;defb116;wnt8b;recql;coch;rap2a;rps3a;irf2;dlg3;uevld;phb2;gtf2e1;ccdc11;gsk3b;rpgrip1;nck1;soat1;cd69;aqp1;lias;thoc3;cdkl3;pitpnm3;vwf;aldh5a1;znf641;syt9;sv2a;pax3;clasp1;bpi;cacna1i;coro2b;dnajc17;tagap;gpr142;astl;galnt5;cytl1;tcn1;hivep1;ulk2;lcat;b3galt5;gucy1a2;prkca;itgal;clgn;pitpnb;smarcad1;znf667;ppbp;sfxn1;slc22a20;znf175;rin2;ebi3;anxa3;tmem48;dag1;sohlh2;il18r1;ect2;pex19;dpf3;e2f6;gdf5;cspg4;plcg1;fanca;clptm1;ighmbp2;actr1a;atrn;mmrn1;atrip;ube2d3;med18;nrbf2;kif3b;pdcd1;mrps5;isl2;slc6a19;ndrg4;mus81;trim15;qdpr;slc25a22;ptp4a3;malt1;pkp2;idi1;mrpl49;nek9;ndufs8;abcb8;cdc37l1;nphp4;ctnna3;p2rx4;mrpl40;tbc1d10b;adamts14;akt1s1;abca5;osbpl7;bcl2l13;nyx;hpcal4;aurkb;pop1;nppb;adprhl1;pcdhga10;atp6v1a;ccr9;wfs1;tbk1;cpd;foxn1;megf8;sdk1;qars;znf35;nptx2;cxcl12;slc2a6;ly6e;cd1b;ilkap;znf23;ppp1r2;nr0b2;rab35;tert;noto;chst3;ldha;pcolce;slc25a13;grk4;clcn2;sptlc1;cd6;samhd1;sh3gl2;tmprss12;crnkl1;rgl3;smarcd2;rps6kb2;psat1;arhgap5;tm4sf1;cfi;ercc3;irak2;proc;parp4;rfx2;rbck1;sar1a;gpr31;pisd;bmpr1a;atp6v0b;prpf4b;znf16;vprbp;hnrnpl;yes1;znf569;atpif1;pou2f3;dapk2;rnf149;ppy;nup210;skiv2l2;hmgcll1;asb1;sema4f;btg4;rpap1;eya2;kcnk6;gtf2h5;atp5b;rps15a;znf770;rfc5;hes5;ccl19;iqgap1;rnf216;ephb2;sh3bp4;mtnr1b;tmem38a;zmiz2;cutc;klf8;gng12;atp5f1;msh5;znf238;kif13b;pgk2;nfyc;shprh;cryga;tom1l2;cx3cr1;homer3;hivep2;rpl7l1;hebp1;amacr;pkn3;meox1;eif2c3;abtb1;ptprr;poll;tfdp2;pars2;prss22;elovl7;aire;eif5a;sirt6;mef2d;lhfpl5;ppp1r1b;kctd16;rbp3;erg;arhgef5;dnase1l2;ubqlnl;snx21;dkk1;prrx2;rnf111;pou5f1;slc39a11;lonrf2;nanos3;sds;cacng1;abca7;gypc;nfkbie;gdf10;pfkp;esco2;grb10;slc18a1;best1;krt7;naaladl1;cecr5;efcab6;tigd2;rasgef1a;nup160;plch2;brca2;elp4;slc16a1;ncf1;usp29;tpx2;mall;sec11a;pigb;exosc8;col25a1;rassf8;pcdhgb6;pafah2;ppt1;cdc25b;nup98;pold2;parvb;trim23;crx;shank3;slco4a1;ddx41;nr1h3;dchs2;kctd10;gan;ehd1;tnpo3;immp1l;serpini1;atg3;psma6;bik;lman2;polr3d;stxbp1;mapk8ip1;tnfrsf21;casp14;apba3;slc25a37;tmprss9;klrc2;gpr114;col8a2;nab1;acads;snrpd3;mat2a;thnsl1;mmp19;dmgdh;ext2;sept12;pdap1;cd164;kif23;slc13a4;nars;gsta1;thnsl2;gmps;rassf3;wdr19;slco4c1;pigq;slc24a5;exosc10;galnt1;tead4;cfb;pla2g12a;rab18;timm50;dld;ccr4;arsb;gphn;sema4c;gldc;furin;atp5s;tph1;drg2;mst1;farsb;znf300;il15ra;znf275;larp7;ptgis;smpd1;slc39a12;mov10l1;atp6v1e2;pcdhga8;suclg1;hal;gltp;rbp2;psmb6;lfng;nr2e1;notch1;rxrg;clec1a;c4a;poln;pop5;eif4ebp1;masp1;leo1;lypla2;dnhd1;csk;vgll3;rad54b;mog;gpsm2;mef2b;gpr56;arhgef1;ccr3;hars2;ly9;zdhhc1;mettl4;nudc;ung;gfra3;pou5f2;sos1;znf32;aff4;nr1d1;dock7;upf1;acsl1;pnliprp3;atp10b;ptafr;rarres2;setd7;socs6;rtp3;ppp2r2d;arpc1b;rdbp;edf1;vav2;cherp;scrn3;arfip2;zdhhc17;mars;ccna2;rasa2;nr4a1;rho;adck1;ace;insrr;slc25a36;rps14;ndufb5;tcf15;smo;gcgr;dsn1;ak3;tm9sf2;crnn;tpd52;foxp3;gnb1;adora2b;itga3;phf17;chrm1;fgd5;plod3;traf6;mrpl43;kcna7;tkt;asb17;pqbp1;irf5;sdf2;hsd17b11;pes1;diras1;sstr2;emid1;flt3;ntf3;col27a1;naip;snca;fkbp2;cdc23;stx1b;cldn17;fdft1;tcf23;surf4;prph2;mc1r;stx11;phkg1;emp3;fuca2;morf4l1;adam7;gpr45;zscan2;flrt2;suv420h1;myl3;mms19;cd97;pcbp2;sec61a1;kdelr2;chrdl2;adam21;col12a1;stard5;capns1;zp4;vps54;stat3;atg4c;gad2;hspb9;sept2;brms1l;mad2l1;limk2;padi6;p2ry1;nit2;bdh1;npas2;mapkapk2;f2rl1;kif1a;hoxd8;usp10;mmp24;als2;sf3b3;pafah1b2;mrpl17;igsf10;clcn5;pex13;klhl3;gtf2i;mcm10;mcf2l;pabpc1;tlr5;prpf40a;stx18;ift57;adipoq;sox13;chic2;cald1;taf1a;ifit1;dr1;gga1;ccar1;adam33;znf329;pla2g4b;ppfibp2;cdsn;znf555;ssbp2;lifr;hsd11b1;mast3;bub1b;hk1;trmu;rufy3;mmp11;ltc4s;sphar;pcdhb14;shroom1;rpl5;tbc1d24;sdcbp;lars;atp1a1;ntng2;s100a8;corin;pogk;slc20a1;ccl27;scnn1a;svop;lamb2;epha5;bfsp1;gpaa1;egln1;foxr1;sh3rf2;sult1c2;znf34;epn2;cxcl1;hipk4;rxfp1;kif5a;tox3;ppm1g;znrd1;trip10;taok2;sgms2;gata3;wnt4;sart3;trim2;znf470;ifnk;rapgef1;rab8b;gpr20;hp;ddhd2;slc25a12;isoc1;gpr84;znf142;abcc5;fen1;xrn1;zcchc2;obfc1;adcy3;krt13;tmem176b;ctdspl;rabggta;ndufb7;wnt3a;vamp4;kcnj11;aldob;acsbg2;dph3;tbx10;kif27;rgs1;il22ra2;kiaa1109;tsen2;epb41l2;pbrm1;unc45b;stoml3;slc7a13;myo1f;mapk8ip3;vps37a;shc2;lmo2;dmrtb1;slc37a3;cdkl2;klk9;cst11;guk1;avpr1a;ehf;tasp1;ereg;cct6b;ik;mfge8;lipc;pxn;acrv1;ap4s1;sptbn2;bckdhb;cks1b;mpdu1;srf;abhd5;uap1;nmur1;alpk1;wwp1;duoxa2;notch4;scube1;elavl2;glt6d1;pvrl3;rad21;hsf4;dio1;ftcd;rpp14;l3mbtl3;stx16;alas1;aqp3;ube3b;foxd4l1;psip1;limch1;map2k2;stambp;ubl7;pfdn1;crispld2;siva1;cd19;pla2g2c;aoc3;ezh1;hat1;rps23;piwil1;ptprh;ctnnal1;tob2;use1;cercam;clec4e;tbr1;zhx2;nedd9;greb1;gpatch8;lrp3;api5;aldh3a2;mto1;ephb6;pigs;abcg5;nfatc1;sphk1;ssh1;ssh2;lhb;rffl;trim28;trappc1;ucp3;dhx38;iscu;gria1;polr3g;tbl2;slc35f2;slc1a5;cmtm7;cdkn1a;lrrk2;pvrl4;slco2b1;mfrp;slc35f3;eif4g3;smarce1;kcnip1;elavl1;ppig;acp6;sec13;aspm;arhgap28;zbtb7a;cry2;ppme1;lgmn;impa1;fgb;trim10;atm;nod2;lsm10;chmp6;adamts16;trex1;ptk2;armc1;pde1a;acaca;ppil3;snrpa;izumo1;fmod;trim29;cd96;kcnab1;adamts17;il1rl2;fignl1;pick1;clk4;c1qc;ccbl2;anxa9;itpka;glyctk;npffr1;mdc1;rbms2;rce1;mesdc2;lhx3;pyroxd1;taf5;syt17;nkiras2;znf181;slc45a3;casc5;wdr33;rasal1;mccc1;slc17a7;ptpn18;nom1;rgs8;tpt1;sept11;zbtb5;capg;krt8;tmem68;ccdc130;rims4;slc9a3;msmb;fbxo15;slc31a2;ednra;brca1;rhov;nptn;pcdhgb5;usp35;cdc25c;sp100;cecr2;sult1b1;htr3a;nkx2-5;sbf2;pfdn5;crybb1;mtmr7;gne;tnn;atp1b2;cd53;casp8;cplx1;htr5a;skap1;psmb4;dip2a;znf532;carhsp1;kbtbd6;defb124;nek3;fdx1l;dsc1;pik3c2a;stard13;pcdhga1;gal3st3;nfyb;znf529;plxnb2;bcl11b;hexb;mfng;sec24a;anp32a;unc13b;plcb3;ppef2;fbxl3;ca6;rfc1;acsf2;vps28;prkar2b;slc25a6;sec61g;wdr7;gfpt2;hcn1;ing4;arl8b;nr1h4;olfm1;rpl18a;clcn6;rab38;cdc73;pcdhgb4;cpt1c;il15;kiss1;timp1;anapc11;tuft1;slc34a3;msc;serpina5;gas2l3;fgf1;spcs3;fxc1;ubqln4;ecm1;grhpr;hsd17b12;dbp;thrsp;wbp2nl;rps6kb1;cpa4;asgr1;stk24;sertad3;fbxw11;lzts1;senp3;rpl39;c8b;cryba2;mefv;atp1a2;il16;znf584;lctl;ap1s3;nudt21;dgki;b3gnt2;ftsj2;mbtps1;otud7b;vps41;dynlrb2;tle4;hbq1;snap29;znf710;c1s;dst;csnk1g2;dclk1;mycbp2;ttll2;hsd17b2;gaa;zkscan5;usp26;pank4;ncoa1;ppm1m;nup153;nub1;mapk14;rpp38;gpr85;cd37;hoxb13;ofd1;txnrd3;pitx2;fut11;chmp4a;wnt9a;spesp1;pola2;mphosph6;steap3;cntnap2;gfod2;kif18a;mylip;cylc2;med11;ptpn3;acaa2;strn3;gtf3a;pum2;napa;rbm17;znf646;il12rb1;plcd4;usp45;pigz;irak1;msh2;abcf2;mrpl11;gpr171;trim13;fam53b;idua;mapre1;acot9;ric8b;sgpp1;rhobtb2;alg2;cd3d;pcdhga11;gk2;znf524;lonrf3;gcg;dusp7;sept7;mylk2;icam5;klk11;ghitm;aqp8;evpl;chmp1a;fkbp5;znf345;lnx1;grhl3;rasd1;arhgap25;dok1;rad52;smarcb1;brd8;arl6ip1;fbxo8;aarsd1;adamts8;adrb1;map3k1;herc1;pln;urm1;prss21;vrk1;st6gal2;znf688;znf395;casp8ap2;nr4a3;vapb;bbs4;hdgf;tmlhe;cenpj;usp49;rabgap1;cideb;aven;krt4;gpr141;cplx3;rexo2;armc10;zbtb3;ptpn1;pkia;epdr1;rab7l1;rpl23;slc11a1;ncam1;cav1;gabpa;twf2;ambn;ube2b;tceb1;atxn3;uimc1;mmp25;neu2;katnb1;zcchc4;cetn1;fut3;txn;eif4a1;pias4;mat2b;mif4gd;acsl5;kctd11;atp4a;jdp2;psen2;gcnt4;sh2d2a;dnajb5;lcmt1;prelp;obscn;cda;cacng3;cchcr1;ilf3;gda;clp1;rgr;rbak;gal3st1;nat6;faslg;slu7;pcgf5;dis3;pcdha1;srd5a3;angpt1;gopc;sgsm3;flad1;fabp3;camk1;pnpt1;rab3d;oasl;tubb3;snapc4;dmwd;rgs22;mreg;ugcg;hoxc8;dctd;sftpb;cidec;crbn;f2rl3;syt6;cdh26;gba3;foxn2;exosc7;chordc1;mctp2;mkks;zmat5;agpat4;gpr15;rplp1;barx1;prmt7;pcmtd1;gmppa;cask;slc22a14;rab19;hmgcs1;slc27a3;cog2;pnpla4;plxnc1;terf2;mgea5;stx2;nhej1;eef1a2;gng8;sfrp1;ncoa6;chek1;capn10;ccng1;phlda1;tlr3;gpbar1;sfn;nrsn1;nid1;pou2f2;hnrnpr;atf2;sgk2;fam129a;tspan18;med24;cldn4;sypl2;cd248;bard1;vipr2;cmtm4;klk14;bmp6;gzf1;ulk4;csn2;smurf2;rps27;folr2;chodl;msh4;emp1;nacad;gabrp;plcl2;angptl3;wdr67;got1;cacna2d2;rere;tmprss11d;galt;mrps14;bves;sec31a;atf5;etv1;ush1c;npbwr1;ssbp1;msi1 |
| cell cycle process; | 96#625 | 1.96E-18 | nat6;cdc73;mapre1;sgsm3;piwil2;rad21;pes1;anapc11;tubb3;clasp1;reck;tbrg1;gas2l3;ppp1r13b;dmwd;ndc80;chmp1a;cdc23;ttn;ilkap;wbp2nl;ppm1g;rad52;map2k1;smarcb1;lzts1;gadd45a;coro1a;krt7;ptprc;pycard;dlg1;brca2;mad2l1;xrn1;dst;nedd9;rassf4;brca1;adcy3;sirt2;tpx2;nudc;htatip2;chek1;ercc3;spc25;cetn3;ccng1;cdc25c;sphk1;cdc25b;atrip;sfn;apc;katnb1;cntrob;cetn1;pbrm1;cks2;yeats4;spc24;bard1;nek3;mphosph6;txnl4b;ccna1;kctd11;fbxo43;cdkn1a;stard13;mlh3;btg4;msh4;nek9;ccna2;ext2;notch2;aspm;kif23;ephb2;ilf3;bub1b;ereg;tubg1;pds5b;sycp3;dsn1;aurkb;msh2;atm;msh5;ing4;ush1c;cks1b;trim13 |
| cell cycle; | 118#839 | 2.28E-18 | nat6;mcm6;cdc73;mapre1;sgsm3;tfdp2;piwil2;rad21;pes1;anapc11;tubb3;sept7;clasp1;reck;tbrg1;gas2l3;ppp1r13b;dmwd;ndc80;chmp1a;cdc23;ttn;ilkap;wbp2nl;ppm1g;rad52;mdc1;map2k1;smarcb1;lzts1;rif1;pfdn1;gadd45a;esco2;coro1a;krt7;sept11;ptprc;pycard;dlg1;sept2;brca2;rabgap1;mad2l1;xrn1;dst;terf2;nedd9;rassf4;brca1;e2f6;adcy3;sept3;sirt2;tpx2;nudc;htatip2;chek1;spc25;ercc3;cetn3;ccng1;cdc25c;sphk1;cdc25b;atrip;sfn;upf1;apc;katnb1;suv39h1;cntrob;rcbtb1;cetn1;pbrm1;cks2;yeats4;spc24;bard1;nek3;mphosph6;txnl4b;ccna1;kctd11;fbxo43;cdkn1a;stard13;steap3;mlh3;btg4;msh4;ccar1;nek9;sept14;ccna2;ext2;strn3;sept12;notch2;aspm;kif23;ilf3;ephb2;bub1b;ereg;sh3bp4;tubg1;sycp3;pds5b;dsn1;aurkb;msh2;atm;ing4;msh5;ush1c;cks1b;trim13 |
| cellular component organization and biogenesis; | 328#3277 | 7.38E-18 | exosc10;ptk2;prickle2;map3k12;izumo1;rab18;timm50;fmod;arsb;tbrg1;ndc80;arid1b;lrp10;pick1;l3mbtl2;ttn;vps72;sytl3;taf5;epb41;asf1a;cldn14;eif4ebp1;sept11;dnhd1;cav2;capg;krt8;rims4;slc9a3;brca1;myh4;chpt1;tomm40;cd2;actn2;cecr2;apc;upf1;sbf2;gckr;tnn;cntrob;cplx1;kif26b;smg5;bcar1;pou1f1;lrp1;fez2;setd7;socs6;supt3h;rtp3;vav2;canx;tomm34;tnc;hexb;pbx4;arfip2;sec24a;anp32a;tmed10;unc13b;cd320;rfc1;rps14;slc25a36;map2;slc25a6;rps3a;sec61g;dbn1;foxp3;gtf2e1;gsk3b;rab38;phf17;fgd5;nck1;ddx1;slc25a1;rrbp1;spag6;kiss1;vwf;aldh5a1;pes1;clasp1;serpina5;coro2b;snca;kcnma1;stx1b;fxc1;cdc23;asgr2;stk38l;tom1;stx11;emp3;wbp2nl;morf4l1;suv420h1;cpa4;smarcad1;asgr1;sertad3;sec61a1;kdelr2;ap1s3;b3gnt2;ftsj2;col12a1;rin2;sf3a1;mbtps1;vps54;atg4c;tmem48;vps41;avil;dynlrb2;dag1;dlg1;prdm15;tram1;brms1l;snap29;pex19;dst;dclk1;pcdhb2;arid4b;gaa;sirt2;fanca;cetn3;nup153;kif1a;chl1;als2;sf3b3;kif3b;clcn5;igfbp2;pex13;cks2;arpc3;gtf2i;ndrg4;pola2;socs7;stx18;fut10;adipoq;malt1;kif18a;gga1;fblim1;ndufs8;nphp4;tsr2;notch2;utp18;napa;bub1b;mapk8ip2;pds5b;irak1;msh2;nppb;pcdhb14;shroom1;mapre1;sdcbp;ntng2;aaas;lyzl1;cxcl12;sept7;gpaa1;chmp1a;ttf1;stx1a;epn2;cxcl1;kif5a;amph;trip10;taok2;smarcb1;brd8;arl6ip1;pdpn;rab35;tert;atp10a;pycard;bmp10;bbs4;cenpj;cplx3;krt4;sh3gl2;armc10;smarcd2;crnkl1;a4galt;arhgap5;gtf2h2;atg7;rpl23;wnt3a;vamp4;ube2b;dph3;traf1;rtn4;kif27;sar1a;katnb1;cetn1;epb41l2;gap43;pbrm1;atpif1;ppy;nup210;kctd11;srpk2;sema4f;lat;cda;tubgcp6;nod1;pcdhb11;sh3bp4;tubg1;cct6b;lrp1b;pxn;ap4s1;sptbn2;kif13b;osbpl5;slu7;shprh;nmur1;tom1l2;dis3;homer3;scube1;gopc;camk1;rab3d;tubb3;eif5a;rtp4;sirt6;stx16;lhfpl5;exosc7;nrcam;limch1;mkks;nfkbie;abca7;lyg2;ipo8;prmt7;krt7;use1;hspd1;nup160;brca2;cog2;terf2;nedd9;stx2;lrp3;ncoa6;htatip2;exosc8;ppt1;sphk1;nup98;ssh1;sfn;paip2b;ssh2;fyb;rffl;trappc1;ucp3;arcn1;suv39h1;rcbtb1;ehd1;med24;yeats4;tep1;stxbp1;bard1;trmt6;cdkn1a;slc25a37;sdad1;alb;atg12;msh4;mdm2;emp1;eif4g3;smarce1;col8a2;napg;sec13;rere;kif23;cry2;sycp3;sec31a;nod2;gosr1;ush1c;wdr19 |
| regulation of biological process; | 541#6140 | 5.59E-17 | fadd;tead4;cfb;rab18;znf648;znf566;furin;tbrg1;tph1;znf300;pth;l3mbtl2;znf275;znf187;zfpm2;znf644;nr2e1;notch1;rxrg;c4a;myod1;il27ra;eif4ebp1;masp1;glis2;pstk;hoxc10;leo1;csk;cav2;vgll3;rad54b;vsx1;mef2b;arhgef1;sox14;mael;edn1;pou5f2;sos1;myog;znf32;aff4;gas8;dmtf1;taf1b;nr1d1;pnrc2;upf1;cd33;bcar1;pou1f1;pdzd3;socs6;ccna1;rdbp;edf1;vav2;tbc1d23;pbx4;zdhhc17;rapgef2;ccna2;nr4a1;rasa2;boc;cd47;rps14;tcf15;smo;pik3r2;dbn1;foxp3;chrm1;phf17;adora2b;fgd5;ddx1;traf6;tkt;pqbp1;irf5;gck;sstr2;flt3;reck;ntf3;fabp6;znf280d;naip;snca;rpl6;arg2;kcnma1;cdc23;stk38l;tcf23;emp3;morf4l1;zscan2;zbtb32;suv420h1;mms19;cacnb4;kiss1r;gadd45a;capns1;stat3;gad2;avil;prdm15;dlg1;znf629;brms1l;mad2l1;cbfa2t2;arid4b;lepre1;mlxipl;smad5;sirt2;mycl1;npas2;gbx1;hoxd8;als2;znf576;jazf1;trim35;igfbp2;clec11a;cks2;il19;arpc3;znf713;gtf2i;socs7;mcf2l;pabpc1;adipoq;ift57;sox13;taf1a;hoxb4;spred1;dr1;fblim1;znf329;znf555;ssbp2;lifr;tbc1d17;notch2;bcl2a1;znf81;bub1b;mapk8ip2;pds5b;med6;znf672;ift88;tbc1d24;aaas;zfp37;znf212;pogk;slc20a1;gna11;ppp1r13b;yaf2;egln1;foxr1;ttf1;stx1a;znf34;cxcl1;tox3;ppm1g;znrd1;taok2;pdpn;tcf25;gata3;atp10a;fth1;ifnk;znf470;rapgef1;rab8b;znf683;bmp10;pycard;tnni3;znf142;xrn1;znf624;rassf4;gtf2h2;atg7;znf250;wnt3a;rtn4;traf1;dph3;tbx10;sp2;rgs1;dyrk2;il22ra2;znf202;gap43;pbrm1;tlr6;lat;mapk8ip3;dmrtb1;nod1;ehf;ereg;znf22;sptbn2;cks1b;ripk1;srf;tfeb;wwp1;nkx1-2;notch4;elavl2;polr2f;phf20;rbm15;zkscan1;hsf4;lep;l3mbtl3;tbc1d1;psip1;foxd4l1;nrcam;stambp;taok3;kcnq1;nkx2-6;siva1;ezh1;ptprc;tob2;hspd1;foxd2;tbr1;zhx2;nedd9;api5;htatip2;nfatc1;sphk1;paip2b;tnfrsf11a;trim28;suv39h1;rcbtb1;polr3g;pde3b;yeats4;rasgrp3;scap;trmt6;cdkn1a;fgf7;alb;eif4g3;mdm2;ccdc59;smarce1;elavl1;zbtb7a;dhps;cry2;chat;sycp3;fgb;atm;nod2;mcm6;zfp62;psen1;arid1b;pick1;c1qc;ttn;vps72;lhx3;taf5;tlr9;znf181;asf1a;rasal1;rgs8;tpt1;tbc1d2;zbtb5;capg;ncoa3;slc9a3;brca1;nptn;chpt1;cdc25c;cd2;sp100;actn2;apc;nkx2-5;pfdn5;ecd;casp8;smg5;znf532;carhsp1;foxj3;supt3h;tia1;stard13;nfyb;znf529;arntl2;bcl11b;sox9;hexb;pag1;psd3;unc13b;etv2;cd320;rfc1;map2;rps3a;irf2;ing4;dlg3;uevld;nr1h4;phb2;gsk3b;gtf2e1;cdc73;nck1;il15;timp1;aldh5a1;znf641;pax3;clasp1;msc;gas2l3;serpina5;ecm1;hivep1;dbp;thrsp;prkca;smarcad1;sertad3;lzts1;znf667;c8b;atp1a2;znf584;il16;znf175;ebi3;mbtps1;otud7b;tle4;sohlh2;ect2;znf710;dpf3;dst;e2f6;mycbp2;gdf5;zkscan5;ncoa1;ighmbp2;atrip;hoxb13;ube2d3;med18;nrbf2;pitx2;isl2;malt1;med11;gtf3a;tbc1d10b;akt1s1;pum2;znf646;il12rb1;bcl2l13;irak1;msh2;nppb;trim13;tbk1;mapre1;ric8b;foxn1;znf524;znf35;cxcl12;chmp1a;znf345;grhl3;ilkap;ppp1r2;znf23;brd8;fbxo8;nr0b2;smarcb1;adamts8;rab35;adrb1;pln;noto;grk4;znf688;znf395;casp8ap2;nr4a3;vapb;rabgap1;cideb;cplx3;krt4;aven;zbtb3;armc10;smarcd2;rgl3;cfi;ercc3;gabpa;proc;tceb1;rfx2;ube2b;atxn3;uimc1;bmpr1a;katnb1;znf16;znf569;atpif1;dapk2;pou2f3;pias4;asb1;kctd11;btg4;jdp2;eya2;obscn;cda;gtf2h5;znf770;hes5;iqgap1;ephb2;ilf3;zmiz2;klf8;znf238;rbak;nat6;faslg;nfyc;pcgf5;hivep2;meox1;sgsm3;gopc;tfdp2;fabp3;rab3d;snapc4;aire;eif5a;sirt6;rgs22;mef2d;hoxc8;cidec;erg;arhgef5;dkk1;prrx2;foxn2;pou5f1;nanos3;nfkbie;barx1;krt7;efcab6;tigd2;rasgef1a;brca2;terf2;elp4;eef1a2;sfrp1;ncoa6;chek1;ccng1;tlr3;ppt1;cdc25b;sfn;crx;nr1h3;pou2f2;atf2;med24;bik;bard1;mapk8ip1;bmp6;gzf1;smurf2;nab1;angptl3;wdr67;ext2;rere;cd164;etv1;atf5;npbwr1 |
| cellular process#regulation of cellular process; | 503#5704 | 1.38E-15 | mcm6;fadd;zfp62;tead4;rab18;znf648;znf566;psen1;furin;tbrg1;arid1b;znf300;pth;l3mbtl2;znf275;ttn;znf187;zfpm2;znf644;vps72;lhx3;taf5;tlr9;znf181;asf1a;rasal1;nr2e1;notch1;rxrg;myod1;rgs8;eif4ebp1;tpt1;glis2;pstk;tbc1d2;hoxc10;leo1;zbtb5;csk;cav2;capg;vgll3;rad54b;vsx1;mef2b;ncoa3;slc9a3;arhgef1;brca1;sox14;mael;edn1;pou5f2;sos1;myog;znf32;chpt1;aff4;cdc25c;gas8;sp100;cd2;dmtf1;actn2;taf1b;nr1d1;pnrc2;nkx2-5;apc;upf1;pfdn5;casp8;smg5;cd33;bcar1;pou1f1;znf532;carhsp1;pdzd3;foxj3;socs6;supt3h;ccna1;tia1;stard13;rdbp;edf1;vav2;nfyb;znf529;arntl2;bcl11b;sox9;tbc1d23;hexb;pbx4;zdhhc17;rapgef2;psd3;unc13b;ccna2;nr4a1;rasa2;etv2;boc;cd320;cd47;rfc1;rps14;map2;tcf15;rps3a;smo;irf2;ing4;dlg3;pik3r2;foxp3;phb2;nr1h4;gtf2e1;gsk3b;chrm1;phf17;fgd5;cdc73;nck1;ddx1;traf6;pqbp1;il15;irf5;timp1;znf641;gck;sstr2;pax3;flt3;clasp1;msc;reck;ntf3;serpina5;fabp6;gas2l3;znf280d;naip;snca;rpl6;cdc23;ecm1;hivep1;stk38l;tcf23;thrsp;dbp;emp3;morf4l1;zscan2;zbtb32;prkca;suv420h1;mms19;sertad3;smarcad1;lzts1;znf667;cacnb4;kiss1r;znf584;gadd45a;znf175;ebi3;capns1;mbtps1;stat3;otud7b;avil;prdm15;dlg1;sohlh2;tle4;znf629;brms1l;ect2;znf710;mad2l1;cbfa2t2;dst;dpf3;arid4b;e2f6;mycbp2;lepre1;mlxipl;smad5;sirt2;gdf5;zkscan5;npas2;mycl1;ncoa1;ighmbp2;gbx1;hoxd8;hoxb13;atrip;als2;znf576;jazf1;med18;trim35;nrbf2;pitx2;igfbp2;clec11a;cks2;isl2;il19;arpc3;znf713;gtf2i;socs7;mcf2l;pabpc1;adipoq;ift57;sox13;taf1a;malt1;hoxb4;spred1;dr1;znf329;med11;znf555;ssbp2;lifr;tbc1d17;gtf3a;notch2;tbc1d10b;akt1s1;pum2;bcl2a1;znf81;bub1b;mapk8ip2;znf646;il12rb1;bcl2l13;pds5b;irak1;msh2;med6;nppb;trim13;znf672;ift88;tbk1;tbc1d24;mapre1;ric8b;aaas;zfp37;znf212;pogk;foxn1;slc20a1;znf524;znf35;cxcl12;gna11;ppp1r13b;yaf2;egln1;foxr1;chmp1a;ttf1;znf345;znf34;cxcl1;grhl3;ilkap;tox3;ppm1g;znrd1;znf23;ppp1r2;taok2;smarcb1;nr0b2;fbxo8;brd8;pdpn;rab35;adamts8;tcf25;pln;gata3;noto;fth1;ifnk;znf470;rapgef1;rab8b;znf683;grk4;znf688;znf395;nr4a3;casp8ap2;bmp10;pycard;znf142;rabgap1;cideb;aven;krt4;xrn1;znf624;armc10;zbtb3;rgl3;smarcd2;rassf4;gtf2h2;ercc3;znf250;wnt3a;proc;gabpa;rfx2;tceb1;atxn3;rtn4;traf1;uimc1;tbx10;sp2;rgs1;bmpr1a;katnb1;dyrk2;il22ra2;znf202;gap43;znf16;pbrm1;atpif1;znf569;pou2f3;dapk2;pias4;tlr6;asb1;kctd11;jdp2;btg4;eya2;mapk8ip3;cda;obscn;gtf2h5;dmrtb1;znf770;nod1;hes5;iqgap1;ilf3;ephb2;ehf;ereg;znf22;zmiz2;klf8;znf238;rbak;sptbn2;cks1b;ripk1;srf;nat6;faslg;nfyc;tfeb;pcgf5;wwp1;hivep2;meox1;nkx1-2;notch4;elavl2;gopc;sgsm3;fabp3;tfdp2;polr2f;rab3d;phf20;snapc4;aire;rbm15;zkscan1;hsf4;eif5a;rgs22;sirt6;lep;l3mbtl3;hoxc8;mef2d;cidec;tbc1d1;erg;arhgef5;psip1;foxd4l1;foxn2;prrx2;dkk1;nrcam;pou5f1;stambp;nanos3;taok3;nfkbie;nkx2-6;siva1;barx1;ezh1;krt7;ptprc;tigd2;efcab6;tob2;rasgef1a;hspd1;brca2;foxd2;tbr1;zhx2;terf2;nedd9;elp4;eef1a2;sfrp1;api5;ncoa6;htatip2;chek1;ccng1;nfatc1;tlr3;ppt1;sphk1;cdc25b;sfn;paip2b;tnfrsf11a;trim28;crx;nr1h3;pou2f2;suv39h1;atf2;rcbtb1;polr3g;yeats4;med24;bik;rasgrp3;mapk8ip1;bard1;scap;trmt6;bmp6;gzf1;cdkn1a;fgf7;smurf2;alb;eif4g3;mdm2;nab1;ccdc59;smarce1;angptl3;elavl1;wdr67;ext2;rere;cd164;zbtb7a;dhps;cry2;sycp3;fgb;atm;atf5;nod2;etv1 |
| M phase; | 56#306 | 1.51E-15 | adcy3;mapre1;sirt2;tpx2;nudc;chek1;spc25;cetn3;ccng1;cdc25c;cdc25b;piwil2;rad21;pes1;anapc11;tubb3;clasp1;katnb1;dmwd;ndc80;cetn1;pbrm1;cks2;chmp1a;yeats4;cdc23;spc24;ttn;mphosph6;nek3;txnl4b;ccna1;fbxo43;wbp2nl;rad52;map2k1;mlh3;msh4;nek9;ccna2;aspm;coro1a;kif23;ilf3;bub1b;ereg;dlg1;pds5b;sycp3;dsn1;aurkb;brca2;atm;mad2l1;msh5;nedd9 |
| multicellular organismal development#system development#organ development; | 139#1141 | 1.82E-14 | cd3d;tead4;foxn1;gna11;egln1;evpl;pth;dsg4;ttn;znrd1;mesdc2;pdpn;tcf25;lhx3;pln;rbp2;lfng;notch1;myod1;fzd6;il17c;bmp10;cav2;tnni3;mef2b;gpr56;gnpat;cyp11a1;krt13;tmem176b;edn1;ercc3;myog;wnt3a;cd2;ambn;nkx2-5;bmpr1a;atpif1;pou1f1;unc45b;pou2f3;asb1;eya2;canx;sh2d2a;prelp;tnc;sox9;hexb;pbx4;hes5;lhcgr;boc;ccm2;snai1;tcf15;ereg;znf22;smo;tpd52;srf;adora2b;cryga;dyrk3;notch4;scube1;angpt1;timp1;rab3d;tuft1;pax3;hsf4;mreg;fgf1;lep;ugcg;mef2d;sftpb;lhfpl5;krt14;mkks;gypc;gdf10;sfxn1;nkx2-6;crispld2;chrdl2;barx1;col12a1;c1galt1;ptprc;brca2;tbr1;stx2;gaa;nhej1;sirt2;smad5;clec3a;ncoa6;aldh3a2;htatip2;cspg4;tlr3;ppt1;sphk1;hoxb13;sfn;als2;tnfrsf11a;lhb;igsf10;crx;pitx2;pex13;pde3b;trim15;fut10;casp14;bmp6;gzf1;fgf7;sh2b3;chodl;msh4;emp1;cdsn;angptl3;mmp19;hsd11b1;ext2;notch2;napa;bves;trim10;nppb;ush1c;ift88 |
| anatomical structure morphogenesis; | 130#1047 | 2.58E-14 | sdcbp;prickle2;ntng2;corin;foxn1;egln1;evpl;taok2;brd8;pdpn;adrb1;lhx3;gata3;atp10a;casc5;lfng;fzd6;hoxc10;bmp10;capg;cav2;tnni3;krt4;armc10;gnpat;arhgap5;tmem176b;edn1;ercc3;chpt1;wnt3a;ambn;ube2b;actn2;rtn4;tbx10;nkx2-5;bmpr1a;tnn;gap43;pou1f1;atpif1;bcar1;fez2;socs6;kctd11;sema4f;eya2;vav2;canx;sh2d2a;cda;arfip2;cd320;ehf;ccm2;snai1;ereg;znf22;smo;tpd52;dbn1;adora2b;phf17;srf;fgd5;nck1;notch4;angpt1;spag6;pes1;tuft1;pax3;clasp1;fgf1;sftpb;lhfpl5;krt14;dkk1;nrcam;pou5f1;emp3;morf4l1;mkks;sertad3;gypc;barx1;b3gnt2;nts;ezh1;c1galt1;dlg1;dag1;brms1l;nedd9;stx2;gaa;gdf5;mlxipl;sfrp1;htatip2;cspg4;ppt1;sphk1;chl1;als2;ssh1;shank3;crx;pitx2;igfbp2;yeats4;ndrg4;trim15;socs7;sox13;gzf1;cdkn1a;fblim1;emp1;angptl3;mmp19;notch2;mrpl40;mmp11;nppb;ush1c;trim13;shroom1;ift88 |
| cell division; | 47#245 | 2.86E-14 | stx2;mapre1;sept3;sirt2;nudc;spc25;cetn3;ccng1;wnt3a;cdc25c;cdc25b;rad21;anapc11;cecr2;sept7;clasp1;katnb1;ndc80;cntrob;cetn1;cks2;bcar1;cdc23;spc24;nek3;ccna1;hoxb4;rab35;nek9;sept14;cdc37l1;ccna2;sept12;aspm;kif23;bub1b;sept11;pds5b;sycp3;sept2;dsn1;aurkb;cenpj;brca2;mad2l1;cks1b;nedd9 |
| cell cycle phase; | 61#369 | 1.12E-13 | adcy3;mapre1;sirt2;tpx2;nudc;chek1;spc25;cetn3;ccng1;cdc25c;cdc25b;piwil2;rad21;pes1;anapc11;tubb3;clasp1;katnb1;dmwd;ndc80;cetn1;pbrm1;cks2;chmp1a;yeats4;cdc23;spc24;ttn;ilkap;mphosph6;nek3;txnl4b;ccna1;fbxo43;wbp2nl;rad52;cdkn1a;map2k1;mlh3;msh4;nek9;ccna2;aspm;coro1a;kif23;ilf3;krt7;bub1b;ereg;ptprc;dlg1;pds5b;sycp3;dsn1;aurkb;brca2;atm;mad2l1;msh5;ush1c;nedd9 |
| protein localization; | 119#961 | 6.05E-13 | rab38;sys1;sdcbp;tom1l2;homer3;duoxa2;rrbp1;rab18;timm50;gopc;rab3d;mcfd2;rtp4;chmp7;gpaa1;stx16;pick1;chmp1a;stx1b;fxc1;stx1a;snx21;tom1;stx11;mitd1;taok2;rab17;arl6ip1;rab35;nfkbie;sytl3;ipo8;sec61a1;kdelr2;ap1s3;rab8b;mbtps1;vps54;atg4c;cog7;tmem48;vps41;akap7;pycard;tram1;use1;nup160;hspd1;snap29;cog2;pex19;aftph;stx2;ccdc91;atg7;lrsam1;rpl23;rab7l1;ercc3;nup153;tomm40;ppt1;nup98;als2;dph3;fyb;rffl;cog6;sar1a;arcn1;gckr;katnb1;chmp4a;tnpo3;pex13;atg3;pola2;lman2;ppy;nup210;stx18;stxbp1;fut10;arf4;bard1;apba3;rtp3;sdad1;kif18a;canx;gga1;tomm34;vps37a;snx27;nacad;sec24a;zdhhc17;tmed10;angptl3;napg;sec13;rere;napa;cry2;vps28;sec61g;lrp1b;sec31a;clpx;ap4s1;hcn1;nod2;chmp6;uevld;gosr1;foxp3;kif13b;wdr19;gsk3b |
| macromolecule localization; | 123#1012 | 1.18E-12 | rab38;sys1;sdcbp;tom1l2;homer3;duoxa2;thoc3;rrbp1;rab18;timm50;gopc;rab3d;mcfd2;rtp4;chmp7;gpaa1;stx16;pick1;chmp1a;stx1b;fxc1;stx1a;snx21;tom1;stx11;mitd1;taok2;rab17;arl6ip1;rab35;nfkbie;casc3;sytl3;ipo8;sec61a1;kdelr2;ap1s3;rab8b;mbtps1;vps54;atg4c;cog7;tmem48;vps41;akap7;pycard;tram1;use1;nup160;hspd1;snap29;cog2;pex19;aftph;stx2;ccdc91;atg7;ercc3;lrsam1;rpl23;rab7l1;nup153;tomm40;ppt1;nup98;als2;dph3;fyb;rffl;cog6;upf1;sar1a;arcn1;gckr;katnb1;chmp4a;tnpo3;pex13;smg5;atg3;pola2;lman2;ppy;nup210;stx18;stxbp1;fut10;arf4;bard1;apba3;rtp3;sdad1;kif18a;canx;gga1;tomm34;vps37a;snx27;nacad;sec24a;zdhhc17;angptl3;tmed10;napg;sec13;rere;napa;cry2;vps28;sec61g;lrp1b;sec31a;clpx;ap4s1;hcn1;nod2;chmp6;uevld;gosr1;foxp3;kif13b;wdr19;gsk3b |
| positive regulation of biological process; | 127#1062 | 1.94E-12 | srf;chrm1;faslg;nck1;fadd;traf6;cfb;notch4;il15;slc20a1;timp1;gck;flt3;hsf4;ntf3;ppp1r13b;arid1b;lep;yaf2;cidec;pth;c1qc;ttf1;ecm1;nrcam;stambp;prkca;taok2;mms19;sertad3;pdpn;smarcad1;adrb1;taok3;gata3;c8b;tlr9;siva1;ifnk;asf1a;ebi3;capns1;c4a;il27ra;masp1;glis2;hoxc10;casp8ap2;vapb;ptprc;pycard;cav2;ncoa3;ect2;brca2;cideb;brca1;smad5;cfi;npas2;nptn;ncoa6;edn1;htatip2;ncoa1;ercc3;myog;tlr3;ppt1;cd2;sphk1;cdc25b;sfn;tnfrsf11a;trim35;trim28;nkx2-5;crx;bmpr1a;katnb1;dyrk2;casp8;clec11a;il19;yeats4;bik;pou1f1;bcar1;dapk2;tlr6;bard1;adipoq;scap;bmp6;tia1;malt1;cdkn1a;fgf7;sox9;lifr;zdhhc17;unc13b;angptl3;ccna2;nod1;notch2;cd47;boc;ilf3;dhps;ehf;ereg;il12rb1;rps3a;smo;bcl2l13;zmiz2;irak1;fgb;med6;nod2;foxp3;trim13;ripk1;tbk1;gsk3b;ift88 |
| localization; | 398#4481 | 2.31E-12 | slc24a5;btbd10;slc8a1;armc1;rab18;timm50;col23a1;slc35a5;ccr4;arsb;kcnab1;atp5s;tbrg1;abca12;chmp7;lrp10;pick1;dnah3;c1qc;scn2a;trpv2;slc39a12;atp6v1e2;mitd1;gltp;sytl3;rbp2;syt17;slc45a3;wdr33;cacng7;slc17a7;apod;tpt1;dnhd1;cav2;rims4;slc9a3;ednra;slc31a2;myh4;edn1;tomm40;gas8;cd2;cecr2;slc35f4;htr3a;cog6;upf1;gckr;tnn;atp1b2;cplx1;kif26b;smg5;bcar1;pou1f1;slc1a4;atp10b;slc9a1;lrp1;pdzd3;fez2;rtp3;arpc1b;kctd18;vav2;canx;tomm34;syt2;snx27;cpt1b;arfip2;sec24a;zdhhc17;anp32a;tmed10;unc13b;slc26a3;scn11a;slc25a36;pkd2l1;vps28;slc25a6;sec61g;smo;tm9sf2;hcn1;uevld;emid2;c1qtnf5;tpd52;foxp3;clcn6;gsk3b;rab38;sys1;adora2b;rbp4;nck1;bet3l;aqp1;slc25a1;kcna7;col5a1;thoc3;rrbp1;pitpnm3;syt9;gck;slc34a3;sv2a;emid1;ntf3;fabp6;col27a1;cacna1i;snca;kcnma1;stx1b;fxc1;cdc23;tcn1;asgr2;kcnj10;tom1;lcat;grhpr;stx11;prkca;itgal;rab17;pitpnb;cd97;asgr1;ppbp;cacnb4;atp1a2;sec61a1;slc4a10;sfxn1;kiss1r;kdelr2;il16;ap1s3;b3gnt2;slc22a20;slc13a5;col12a1;rin2;stard5;actb;mbtps1;vps54;atg4c;stat3;cog7;tmem48;vps41;dynlrb2;akap7;tram1;hbq1;snap29;pex19;dclk1;aftph;ccdc91;gaa;cspg4;slc22a16;slc39a13;nup153;kif1a;mapk14;actr1a;chl1;als2;osbpl2;kif3b;tacr2;clcn5;chmp4a;pex13;arpc3;grin2c;slc6a19;gabrr3;pola2;stx18;fut10;slc25a22;adipoq;cald1;malt1;steap3;mylip;kif18a;gga1;abcb8;p2rx4;abca5;osbpl7;napa;clpx;nppb;abcf2;atp6v1a;ift88;sdcbp;atp1a1;aaas;slc35b3;slc20a1;scnn1a;svop;pdzk1;slc2a6;slc26a9;slc28a3;aqp8;gpaa1;chmp1a;stx1a;epn2;kif5a;amph;trip10;map2k1;taok2;arl6ip1;pdpn;rab35;pln;casc3;atp10a;fth1;rab8b;slc25a13;coro1a;clcn2;slc25a12;pycard;bmp10;slc7a14;abcc5;cplx3;sh3gl2;slc6a12;atg7;lrsam1;rpl23;rab7l1;ercc3;slc11a1;cav1;vamp4;kcnj11;parp4;dph3;rtn4;kif27;sar1a;katnb1;atp6v0b;slc34a1;slc25a34;yes1;ppy;nup210;txn;slc7a13;kctd11;atp4a;sema4f;lat;kcnk6;mapk8ip3;vps37a;grik5;atp5b;cacng3;slc37a3;sh3bp4;tmem38a;lrp1b;lipc;pxn;ap4s1;znf238;atp5f1;sptbn2;kif13b;osbpl5;slc3a1;nmur1;tom1l2;homer3;duoxa2;slc22a4;slc16a14;ltb4r2;gopc;fabp3;camk1;rab3d;tubb3;abca3;mcfd2;slc12a3;rtp4;stx16;aqp3;kctd16;syt6;rbp3;atp5g2;snx21;nrcam;slc39a11;cacng1;abca7;nfkbie;kcnq1;ipo8;akap9;slc30a10;abcc9;slc18a1;best1;slc22a14;ptprc;use1;cercam;hspd1;nup160;cog2;stx2;slc16a1;lrp3;htatip2;col25a1;abcg5;ppt1;sphk1;nup98;fyb;rffl;trappc1;ucp3;slco4a1;arcn1;ttyh1;p2rx2;slc5a9;kctd10;gria1;tnpo3;ehd1;tspan18;pde3b;atg3;slc35f2;lman2;slc1a5;sypl2;stxbp1;arf4;mapk8ip1;bard1;apba3;csn2;slc25a37;slco2b1;sdad1;folr2;alb;slc35f3;col8a2;nacad;gabrp;kcnip1;angptl3;napg;sec13;cacna2d2;rere;kif23;cry2;slc13a4;sec31a;slc19a1;nod2;chmp6;gosr1;wdr19;slco4c1 |
| protein localization#establishment of protein localization; | 114#922 | 2.31E-12 | rab38;sys1;sdcbp;tom1l2;homer3;duoxa2;rrbp1;rab18;timm50;gopc;rab3d;mcfd2;rtp4;chmp7;stx16;pick1;chmp1a;stx1b;fxc1;stx1a;snx21;tom1;stx11;mitd1;taok2;rab17;arl6ip1;rab35;nfkbie;sytl3;ipo8;sec61a1;kdelr2;ap1s3;rab8b;mbtps1;vps54;atg4c;cog7;tmem48;vps41;pycard;tram1;use1;nup160;hspd1;snap29;cog2;pex19;aftph;stx2;ccdc91;atg7;lrsam1;rpl23;rab7l1;nup153;tomm40;ppt1;nup98;dph3;fyb;rffl;cog6;sar1a;arcn1;gckr;katnb1;chmp4a;tnpo3;pex13;atg3;pola2;lman2;ppy;nup210;stx18;stxbp1;fut10;arf4;bard1;apba3;rtp3;sdad1;kif18a;canx;gga1;tomm34;vps37a;snx27;nacad;sec24a;zdhhc17;tmed10;angptl3;napg;sec13;rere;napa;cry2;vps28;sec61g;lrp1b;sec31a;clpx;ap4s1;nod2;chmp6;uevld;gosr1;foxp3;kif13b;wdr19;gsk3b |
| primary metabolic process; | 967#12764 | 4.29E-12 | pigq;sacs;exosc10;galnt1;naglu;map3k12;tead4;fbxo10;cfb;pla2g12a;usp30;rab18;timm50;znf648;myo3a;psmc2;znf566;arsb;gldc;furin;tbrg1;tph1;baat;mst1;farsb;lrp10;znf300;pth;l3mbtl2;adamts13;znf275;larp7;znf187;zfpm2;smpd1;ptgis;psmf1;atp6v1e2;znf644;tec;hal;suclg1;psmb6;pdpr;arih1;nr2e1;notch1;rxrg;c4a;phkg2;poln;pop5;myod1;il27ra;eif4ebp1;masp1;glis2;pstk;hoxc10;leo1;csk;lypla2;cav2;vgll3;rad54b;vsx1;mef2b;mmp7;dtd1;hars2;mettl4;zdhhc1;gnpat;sox14;endog;wdr5b;mapk10;mael;ung;rfc3;edn1;pou5f2;myog;znf32;psenen;cpb2;aff4;dmtf1;taf1b;nr1d1;pnrc2;upf1;dhodh;acsl1;rpl8;pnliprp3;pou1f1;pdzd3;rarres2;pdia6;setd7;socs6;rdbp;dhx35;edf1;cherp;scrn3;mtmr2;pbx4;mars;ccna2;rho;nr4a1;adck1;ace;dhrs3;insrr;rps14;tcf15;ak3;fbxl7;foxp3;chrm1;phf17;ddx1;man2b2;plod3;dyrk3;traf6;mrpl43;rrbp1;asb17;pqbp1;irf5;sdf2;hsd17b11;gck;st6galnac1;flt3;b3gat1;fabp6;znf280d;snca;rpl6;tbxas1;khk;fkbp2;arg2;cdc23;fdft1;stk38l;tcf23;phkg1;fuca2;morf4l1;adam7;zscan2;zbtb32;flrt2;suv420h1;l2hgdh;mms19;gk;pglyrp1;pcbp2;lct;gadd45a;glt25d2;adam21;stard5;sf3a1;atg4c;stat3;rps5;gad2;avil;prpf3;prdm15;dalrd3;fancb;znf629;brms1l;limk2;cbfa2t2;padi6;arid4b;lepre1;mlxipl;smad5;sirt2;pomgnt1;mycl1;npas2;mapkapk2;usp7;gbx1;hoxd8;usp10;mmp24;als2;znf576;jazf1;sf3b3;osbpl2;pafah1b2;mrpl17;igsf10;wwp2;klhl3;pex13;amdhd2;ide;il19;arpc3;znf713;gtf2i;mcm10;socs7;pabpc1;prpf40a;fut10;proca1;adipoq;sox13;taf1a;hoxb4;hmgcl;dr1;gga1;adam33;znf329;pla2g4b;ppfibp2;znf555;ssbp2;mrpl9;tsr2;hsd11b1;mast3;notch2;trip11;utp18;znf81;bub1b;fam83d;mapk8ip2;hk1;trmu;mmp11;med6;clpx;ltc4s;lypla1;sphar;usp53;znf672;ift88;nek10;rpl5;lars;prepl;mrpl30;zfp37;corin;znf212;pogk;gna15;cpa5;fkbp10;gys2;upp2;epha5;gna11;yaf2;egln1;gpaa1;foxr1;tpp2;sh3rf2;ttf1;znf34;hipk4;tox3;znrd1;ppm1g;map2k1;taok2;tcf25;sgms2;gata3;sart3;pofut2;asb16;ifnk;znf470;rab8b;ddc;znf683;hp;ddhd2;hibadh;pycard;ulk3;znf142;fen1;znf624;loxl1;a4galt;adcy3;cyp11a1;gtf2h2;nrip2;manba;atg7;rabggta;lrsam1;znf250;vamp4;cpsf1;usp46;aldob;traf1;tbx10;dph3;sp2;tsen2;kiaa1109;dyrk2;il22ra2;znf202;pbrm1;tlr6;mthfd1;srpk2;gcnt3;dmrtb1;gucy2c;cdkl2;nod1;klk9;guk1;ehf;tasp1;ereg;tubg1;cct6b;znf22;pxn;glb1l;lipc;ap4s1;sptbn2;bckdhb;mpdu1;ripk1;osbpl5;slc3a1;srf;abhd5;uap1;tfeb;alpk1;wwp1;nkx1-2;notch4;elavl2;scube1;tsn;prkacb;polr2f;glt6d1;phf20;rad21;serinc2;hs3st3b1;rbm15;zkscan1;hsf4;dio1;lep;rpp14;ftcd;l3mbtl3;nudt1;usp2;papolb;atp5g2;ube3b;stk33;psip1;foxd4l1;map2k2;stambp;rnase6;ubl7;taok3;pfdn1;ipo8;lyg2;sae1;nkx2-6;trim32;txk;qtrtd1;parp1;pla2g2c;ezh1;hat1;rps23;piwil1;ptprh;ptprc;isy1;use1;cercam;hspd1;foxd2;tbr1;zhx2;aldh3a2;htatip2;alg5;mto1;ephb6;pigs;nfatc1;sphk1;ssh1;paip2b;ssh2;fyb;rffl;trim28;ucp3;dhx38;arcn1;suv39h1;rcbtb1;polr3g;yeats4;tep1;prpsap1;scap;trmt6;dnajc13;lrrk2;mlh3;atg12;eif4g3;mdm2;pnpla8;smarce1;ccdc59;elavl1;napg;ppig;acp6;zbtb7a;dhps;mtfmt;cry2;ppme1;lgmn;sycp3;impa1;fgb;mgat2;atm;nod2;adamts16;lsm10;trex1;ptk2;mcm6;dusp12;sardh;zfp62;mertk;ppil3;acaca;snrpa;rnf34;b3gnt3;fmod;trim29;ears2;stk25;adamts17;psen1;arid1b;fignl1;rpl28;pick1;epha7;c1qc;clk4;cilp;ttn;itpka;calcoco1;rps9;glyctk;mdc1;vps72;rbms2;afmid;rce1;lhx3;rars;taf5;tlr9;znf181;rpl12;wdr33;asf1a;mccc1;ptpn18;cldn14;ublcp1;nom1;apod;sept11;zbtb5;prmt2;capg;ncoa3;fbxo15;brca1;tk1;ptpn4;usp35;chpt1;me3;cdc25c;sp100;actn2;cecr2;sult1b1;bbs10;napsa;apc;nkx2-5;sbf2;pfdn5;mtmr7;ecd;gne;dnaja1;casp8;smg5;psmb4;znf532;lrp1;carhsp1;foxj3;ppm1f;nek3;txnl4b;supt3h;pik3c2a;fbxo43;gal3st3;nfyb;canx;znf529;arntl2;bcl11b;cpt1b;sox9;hexb;mrpl24;plcb3;etv2;ppef2;ganab;fbxl3;pla2g12b;rfc1;usp48;map2;prkar2b;recql;rps3a;wdr7;irf2;gfpt2;ing4;uevld;phb2;nr1h4;gtf2e1;gsk3b;rpl18a;cdc73;nck1;soat1;cpt1c;thoc3;cdkl3;pitpnm3;vwf;timp1;anapc11;aldh5a1;znf641;pax3;clasp1;msc;serpina5;dnajc17;spcs3;astl;galnt5;ubqln4;hivep1;ulk2;lcat;hsd17b12;b3galt5;dbp;thrsp;gucy1a2;prkca;rps6kb1;clgn;cpa4;smarcad1;stk24;sertad3;pitpnb;lzts1;fbxw11;znf667;senp3;rpl39;c8b;znf584;ap1s3;lctl;nudt21;b3gnt2;znf175;ftsj2;ebi3;mbtps1;otud7b;tmem48;dag1;sohlh2;tle4;znf710;csnk1g2;dpf3;dclk1;e2f6;ttll2;mycbp2;hsd17b2;gaa;zkscan5;usp26;plcg1;ncoa1;fanca;ppm1m;ighmbp2;mapk14;rpp38;cd37;hoxb13;atrip;ube2d3;med18;nrbf2;pitx2;fut11;mrps5;isl2;mus81;pola2;qdpr;ptp4a3;malt1;mylip;idi1;mrpl49;nek9;med11;ndufs8;ptpn3;acaa2;gtf3a;adamts14;osbpl7;pum2;rbm17;znf646;plcd4;usp45;pigz;irak1;aurkb;msh2;pop1;adprhl1;mrpl11;atp6v1a;tbk1;idua;mapre1;acot9;cpd;sgpp1;alg2;gk2;foxn1;znf524;lonrf3;qars;znf35;cxcl12;dusp7;sept7;mylk2;klk11;evpl;chmp1a;fkbp5;znf345;lnx1;grhl3;ilkap;rad52;znf23;ppp1r2;smarcb1;nr0b2;fbxo8;brd8;aarsd1;rab35;adamts8;map3k1;tert;herc1;noto;urm1;chst3;prss21;vrk1;st6gal2;ldha;grk4;znf688;znf395;sptlc1;casp8ap2;nr4a3;tmlhe;cenpj;usp49;rexo2;tmprss12;zbtb3;ptpn1;smarcd2;crnkl1;rps6kb2;psat1;cfi;ercc3;rpl23;irak2;gabpa;proc;parp4;ube2b;rfx2;tceb1;rbck1;atxn3;uimc1;mmp25;pisd;bmpr1a;katnb1;prpf4b;atp6v0b;znf16;vprbp;hnrnpl;fut3;atpif1;znf569;yes1;pou2f3;dapk2;rnf149;eif4a1;pias4;mat2b;skiv2l2;mif4gd;asb1;acsl5;jdp2;rpap1;eya2;psen2;gcnt4;dnajb5;lcmt1;obscn;cda;gtf2h5;atp5b;rps15a;znf770;rfc5;hes5;rnf216;ilf3;ephb2;zmiz2;gda;klf8;clp1;atp5f1;znf238;msh5;rgr;rbak;gal3st1;pgk2;slu7;shprh;nfyc;pcgf5;hivep2;dis3;rpl7l1;pkn3;meox1;srd5a3;abtb1;eif2c3;gopc;ptprr;poll;fabp3;tfdp2;camk1;pnpt1;pars2;oasl;prss22;elovl7;tubb3;snapc4;aire;eif5a;sirt6;mef2d;ugcg;hoxc8;dctd;sftpb;crbn;erg;rbp3;gba3;dnase1l2;ubqlnl;prrx2;foxn2;exosc7;rnf111;pou5f1;lonrf2;nanos3;mkks;sds;gypc;zmat5;agpat4;pfkp;rplp1;barx1;prmt7;esco2;pcmtd1;cask;krt7;naaladl1;tigd2;efcab6;hmgcs1;slc27a3;plch2;brca2;cog2;pnpla4;terf2;mgea5;elp4;nhej1;eef1a2;usp29;ncoa6;sec11a;chek1;capn10;pigb;exosc8;phlda1;tlr3;pafah2;ppt1;cdc25b;nup98;pold2;trim23;crx;ddx41;slco4a1;nid1;nr1h3;pou2f2;hnrnpr;atf2;gan;sgk2;immp1l;atg3;med24;psma6;polr3d;casp14;bard1;klk14;bmp6;gzf1;ulk4;tmprss9;rps27;smurf2;msh4;nab1;acads;snrpd3;mat2a;plcl2;thnsl1;mmp19;angptl3;dmgdh;got1;ext2;rere;tmprss11d;galt;mrps14;nars;gmps;atf5;etv1;ssbp1 |
| protein transport; | 108#866 | 5.22E-12 | rab38;sys1;sdcbp;tom1l2;homer3;duoxa2;rrbp1;rab18;timm50;gopc;rab3d;mcfd2;rtp4;chmp7;stx16;pick1;chmp1a;stx1b;fxc1;stx1a;snx21;tom1;stx11;mitd1;taok2;rab17;arl6ip1;rab35;nfkbie;sytl3;ipo8;sec61a1;kdelr2;ap1s3;rab8b;mbtps1;vps54;atg4c;cog7;tmem48;vps41;tram1;use1;nup160;hspd1;snap29;cog2;pex19;aftph;stx2;ccdc91;atg7;lrsam1;rpl23;rab7l1;nup153;tomm40;ppt1;nup98;fyb;rffl;cog6;sar1a;arcn1;gckr;katnb1;chmp4a;tnpo3;pex13;atg3;pola2;lman2;nup210;stx18;stxbp1;fut10;arf4;bard1;apba3;rtp3;sdad1;kif18a;gga1;tomm34;vps37a;snx27;nacad;sec24a;zdhhc17;tmed10;angptl3;napg;sec13;rere;napa;cry2;vps28;sec61g;lrp1b;sec31a;clpx;ap4s1;chmp6;uevld;gosr1;kif13b;wdr19;gsk3b |
| positive regulation of cellular process; | 116#954 | 5.54E-12 | srf;chrm1;faslg;nck1;fadd;traf6;notch4;il15;slc20a1;timp1;gck;flt3;hsf4;ntf3;ppp1r13b;arid1b;lep;yaf2;cidec;pth;ttf1;ecm1;nrcam;stambp;prkca;taok2;mms19;sertad3;smarcad1;pdpn;taok3;gata3;tlr9;siva1;asf1a;ebi3;capns1;glis2;hoxc10;casp8ap2;ptprc;pycard;cav2;ncoa3;ect2;brca2;cideb;brca1;smad5;npas2;ncoa6;edn1;htatip2;myog;ercc3;ncoa1;tlr3;ppt1;cd2;sphk1;cdc25b;sfn;tnfrsf11a;trim35;trim28;nkx2-5;crx;bmpr1a;katnb1;dyrk2;casp8;clec11a;il19;yeats4;bik;pou1f1;bcar1;dapk2;tlr6;bard1;adipoq;scap;bmp6;tia1;malt1;cdkn1a;fgf7;sox9;lifr;zdhhc17;unc13b;angptl3;ccna2;nod1;notch2;cd47;boc;ilf3;dhps;ehf;ereg;il12rb1;rps3a;smo;bcl2l13;zmiz2;irak1;fgb;med6;nod2;foxp3;trim13;ripk1;ift88;gsk3b;tbk1 |
| cellular metabolic process; | 959#12668 | 7.1E-12 | pigq;sacs;exosc10;galnt1;naglu;map3k12;tead4;fbxo10;cfb;pla2g12a;usp30;rab18;timm50;znf648;myo3a;psmc2;znf566;arsb;gphn;gldc;furin;tbrg1;tph1;baat;mst1;farsb;znf300;pth;l3mbtl2;adamts13;znf275;larp7;znf187;zfpm2;smpd1;ptgis;psmf1;atp6v1e2;znf644;tec;hal;suclg1;rbp2;psmb6;dak;pdpr;arih1;nr2e1;notch1;rxrg;c4a;phkg2;poln;pop5;myod1;il27ra;eif4ebp1;masp1;glis2;pstk;hoxc10;leo1;csk;lypla2;cav2;vgll3;rad54b;vsx1;mef2b;mmp7;dtd1;hars2;mettl4;zdhhc1;gnpat;sox14;endog;wdr5b;mapk10;mael;ung;rfc3;edn1;pou5f2;myog;znf32;psenen;cpb2;aff4;fmo4;dmtf1;taf1b;nr1d1;pnrc2;upf1;dhodh;acsl1;rpl8;pnliprp3;pou1f1;pdzd3;rarres2;pdia6;setd7;socs6;rdbp;dhx35;edf1;cherp;scrn3;mtmr2;pbx4;mars;ccna2;rho;nr4a1;adck1;ace;dhrs3;insrr;rps14;ndufb5;tcf15;ak3;fbxl7;foxp3;chrm1;phf17;ddx1;man2b2;plod3;dyrk3;traf6;mrpl43;rrbp1;asb17;pqbp1;irf5;sdf2;hsd17b11;gck;mocs3;st6galnac1;flt3;b3gat1;fabp6;znf280d;snca;rpl6;tbxas1;fkbp2;arg2;cdc23;fdft1;stk38l;tcf23;phkg1;morf4l1;adam7;zscan2;zbtb32;flrt2;suv420h1;l2hgdh;mms19;gk;pcbp2;gadd45a;glt25d2;adam21;stard5;sf3a1;atg4c;stat3;rps5;gad2;avil;prpf3;prdm15;dalrd3;fancb;znf629;brms1l;limk2;cbfa2t2;padi6;arid4b;mlxipl;smad5;sirt2;pomgnt1;mycl1;npas2;mapkapk2;usp7;gbx1;hoxd8;usp10;mmp24;als2;znf576;duox2;jazf1;sf3b3;osbpl2;mrpl17;igsf10;wwp2;klhl3;pex13;amdhd2;ide;il19;arpc3;znf713;gtf2i;mcm10;socs7;pabpc1;prpf40a;fut10;proca1;adipoq;sox13;taf1a;hoxb4;hmgcl;dr1;adam33;znf329;pla2g4b;ppfibp2;znf555;ssbp2;mrpl9;tsr2;hsd11b1;mast3;notch2;trip11;utp18;znf81;bub1b;fam83d;mapk8ip2;hk1;trmu;mmp11;med6;clpx;ltc4s;lypla1;sphar;usp53;znf672;ift88;nek10;rpl5;lars;prepl;mrpl30;zfp37;lyzl1;corin;znf212;pogk;gna15;slc20a1;cpa5;fkbp10;gys2;upp2;epha5;gna11;ca3;yaf2;egln1;gpaa1;foxr1;tpp2;sh3rf2;ttf1;sult1c2;znf34;hipk4;tox3;znrd1;ppm1g;map2k1;taok2;tcf25;sgms2;gata3;sart3;pofut2;asb16;ifnk;znf470;rab8b;ddc;znf683;hp;hibadh;pycard;ulk3;znf142;fen1;znf624;loxl1;a4galt;adcy3;cyp11a1;gtf2h2;nrip2;manba;atg7;rabggta;lrsam1;znf250;ndufb7;cpsf1;usp46;aldob;tbx10;dph3;sp2;tsen2;kiaa1109;dyrk2;il22ra2;znf202;pbrm1;slc34a1;ndufb2;tlr6;mthfd1;srpk2;gcnt3;dmrtb1;gucy2c;cdkl2;nod1;klk9;guk1;ehf;tasp1;ereg;tubg1;cct6b;znf22;pxn;lipc;sptbn2;bckdhb;mpdu1;ripk1;osbpl5;slc3a1;srf;abhd5;uap1;tfeb;alpk1;wwp1;nkx1-2;notch4;elavl2;tsn;prkacb;polr2f;phf20;rad21;mcfd2;serinc2;hs3st3b1;rbm15;zkscan1;hsf4;dio1;lep;rpp14;ftcd;l3mbtl3;alas1;nudt1;usp2;papolb;atp5g2;ube3b;stk33;psip1;foxd4l1;map2k2;stambp;rnase6;ubl7;taok3;pfdn1;ipo8;lyg2;sae1;nkx2-6;trim32;txk;qtrtd1;parp1;aoc3;ezh1;hat1;rps23;piwil1;ptprh;ptprc;isy1;cercam;hspd1;rsad1;foxd2;tbr1;zhx2;aldh3a2;htatip2;alg5;mto1;ephb6;pigs;nfatc1;sphk1;ssh1;paip2b;ssh2;fyb;rffl;trim28;dhx38;iscu;suv39h1;rcbtb1;polr3g;yeats4;tep1;prpsap1;scap;trmt6;dnajc13;cdkn1a;lrrk2;mlh3;atg12;eif4g3;mdm2;pnpla8;smarce1;ccdc59;elavl1;nqo1;napg;ppig;zbtb7a;dhps;mtfmt;cry2;ppme1;lgmn;chat;sycp3;impa1;fgb;mgat2;atm;nod2;adamts16;lsm10;trex1;ptk2;mcm6;dusp12;sardh;zfp62;mertk;ppil3;acaca;snrpa;rnf34;b3gnt3;fmod;sod3;trim29;ears2;stk25;adamts17;psen1;arid1b;fignl1;rpl28;pick1;epha7;c1qc;clk4;cilp;ttn;itpka;calcoco1;rps9;glyctk;mdc1;vps72;rbms2;afmid;rce1;lhx3;rars;taf5;tlr9;znf181;rpl12;wdr33;asf1a;mccc1;ptpn18;ublcp1;nom1;zbtb5;prmt2;capg;ncoa3;fbxo15;coq3;brca1;tk1;ptpn4;usp35;chpt1;me3;cdc25c;sp100;cecr2;sult1b1;bbs10;napsa;apc;nkx2-5;sbf2;pfdn5;mtmr7;ecd;gne;dnaja1;casp8;smg5;psmb4;znf532;carhsp1;foxj3;ppm1f;nek3;txnl4b;supt3h;pik3c2a;fbxo43;gal3st3;nfyb;canx;znf529;arntl2;bcl11b;cpt1b;sox9;hexb;mrpl24;plcb3;etv2;ppef2;fbxl3;ca6;pla2g12b;rfc1;usp48;map2;prkar2b;recql;rps3a;wdr7;irf2;gfpt2;ing4;uevld;phb2;nr1h4;gtf2e1;gsk3b;rpl18a;cdc73;nck1;soat1;cpt1c;lias;thoc3;cdkl3;pitpnm3;timp1;anapc11;aldh5a1;znf641;pax3;clasp1;msc;serpina5;dnajc17;spcs3;astl;galnt5;ubqln4;hivep1;ulk2;lcat;hsd17b12;b3galt5;dbp;thrsp;gucy1a2;prkca;rps6kb1;clgn;cpa4;smarcad1;stk24;sertad3;lzts1;fbxw11;znf667;senp3;rpl39;c8b;znf584;nudt21;b3gnt2;znf175;ftsj2;ebi3;mbtps1;otud7b;tmem48;sohlh2;tle4;znf710;csnk1g2;dpf3;dclk1;e2f6;ttll2;mycbp2;hsd17b2;gaa;zkscan5;usp26;pank4;ncoa1;fanca;ppm1m;ighmbp2;mapk14;rpp38;cd37;hoxb13;atrip;ube2d3;med18;nrbf2;txnrd3;pitx2;fut11;mrps5;isl2;mus81;pola2;qdpr;ptp4a3;malt1;mylip;idi1;mrpl49;nek9;med11;ndufs8;ptpn3;acaa2;gtf3a;adamts14;osbpl7;pum2;rbm17;znf646;usp45;pigz;irak1;aurkb;msh2;pop1;adprhl1;mrpl11;atp6v1a;tbk1;idua;mapre1;acot9;cpd;sgpp1;alg2;gk2;foxn1;znf524;lonrf3;qars;znf35;cxcl12;dusp7;mylk2;klk11;evpl;chmp1a;fkbp5;znf345;lnx1;grhl3;ilkap;rad52;znf23;ppp1r2;smarcb1;nr0b2;fbxo8;brd8;aarsd1;rab35;adamts8;map3k1;tert;herc1;noto;urm1;chst3;prss21;vrk1;st6gal2;ldha;grk4;znf688;znf395;sptlc1;casp8ap2;nr4a3;tmlhe;cenpj;usp49;rexo2;tmprss12;zbtb3;ptpn1;smarcd2;crnkl1;rps6kb2;psat1;cfi;ercc3;rpl23;irak2;gabpa;proc;parp4;ube2b;rfx2;tceb1;rbck1;atxn3;uimc1;mmp25;pisd;bmpr1a;katnb1;zcchc4;prpf4b;atp6v0b;znf16;vprbp;hnrnpl;fut3;atpif1;znf569;yes1;pou2f3;dapk2;rnf149;eif4a1;pias4;mat2b;skiv2l2;mif4gd;asb1;acsl5;jdp2;rpap1;eya2;psen2;gcnt4;dnajb5;lcmt1;obscn;cda;gtf2h5;atp5b;rps15a;znf770;rfc5;hes5;rnf216;ilf3;ephb2;zmiz2;gda;klf8;clp1;znf238;msh5;atp5f1;rgr;rbak;gal3st1;pgk2;slu7;shprh;nfyc;pcgf5;hivep2;dis3;rpl7l1;pkn3;meox1;abtb1;eif2c3;ptprr;flad1;poll;fabp3;tfdp2;camk1;pnpt1;pars2;oasl;prss22;elovl7;tubb3;snapc4;aire;eif5a;sirt6;mef2d;ugcg;hoxc8;dctd;sftpb;crbn;erg;rbp3;gba3;dnase1l2;ubqlnl;prrx2;foxn2;exosc7;rnf111;pou5f1;lonrf2;nanos3;mkks;sds;gypc;zmat5;agpat4;pfkp;rplp1;barx1;prmt7;esco2;pcmtd1;cask;krt7;naaladl1;tigd2;efcab6;hmgcs1;slc27a3;brca2;cog2;terf2;mgea5;elp4;nhej1;eef1a2;ncf1;usp29;ncoa6;sec11a;chek1;capn10;exosc8;pigb;phlda1;tlr3;ppt1;cdc25b;nup98;pold2;trim23;crx;ddx41;slco4a1;nid1;nr1h3;pou2f2;hnrnpr;atf2;gan;sgk2;immp1l;atg3;med24;psma6;polr3d;casp14;bard1;klk14;bmp6;gzf1;ulk4;tmprss9;rps27;smurf2;msh4;nab1;acads;snrpd3;mat2a;thnsl1;mmp19;angptl3;dmgdh;got1;ext2;rere;tmprss11d;galt;mrps14;nars;gsta1;gmps;atf5;etv1;ssbp1 |
| cell differentiation#cell development; | 140#1242 | 2.31E-11 | fadd;sgpp1;ntng2;rnf34;ccr4;psen1;ppp1r13b;pick1;pth;ttn;mov10l1;map2k1;taok2;casc5;notch1;myod1;tpt1;casp8ap2;pycard;cav2;cideb;aven;brca1;nptn;edn1;ercc3;myog;wnt3a;cd2;proc;parp4;ube2b;atxn3;actn2;cecr2;rtn4;traf1;dyrk2;tnn;casp8;bcar1;dapk2;fez2;ppm1f;sema4f;tia1;ctnnbl1;btg4;psen2;tnc;sox9;unc13b;nod1;hes5;boc;rnf216;slc25a6;ereg;rps3a;smo;pdcd10;ing4;pik3r2;sptbn2;dbn1;ripk1;gsk3b;phf17;faslg;traf6;spag6;timp1;rab3d;rad21;tubb3;pax3;ntf3;hsf4;eif5a;hoxc8;naip;snca;cidec;lhfpl5;nrcam;emp3;stambp;prkca;mkks;c8b;siva1;b3gnt2;gadd45a;unc5a;ctnnal1;ptprc;hspd1;brca2;emp2;gdf5;eef1a2;sfrp1;api5;htatip2;phlda1;ppt1;sphk1;chl1;sfn;als2;trim35;rffl;trim28;ddx41;pdcd1;pacs2;pex13;il19;bik;tnfrsf21;casp14;bard1;ift57;malt1;cdkn1a;steap3;alb;atg12;ccar1;emp1;notch2;akt1s1;bcl2a1;napa;bub1b;mapk8ip2;sycp3;bcl2l13;nod2;atf5 |
| cell proliferation; | 95#745 | 3E-11 | chrm1;mapre1;nck1;ms4a2;foxn1;il15;fabp3;gcg;timp1;pes1;pdzk1;sstr2;flt3;hsf4;tbrg1;fabp6;retnlb;fgf1;il15ra;erg;cxcl1;dbp;emp3;stambp;map2k1;smarcb1;adamts8;ppbp;fth1;kiss1r;ifnk;ebi3;capns1;hoxc10;csk;ptprc;bmp10;dlg1;cav2;hdgf;tob2;brca2;krt4;arhgef1;ednra;brca1;lepre1;emp2;tpx2;nudc;edn1;chek1;cdc25c;gas8;sphk1;cdc25b;sfn;tnfrsf11a;trim35;apc;bmpr1a;clec11a;cks2;cd33;atpif1;pou1f1;bcar1;lrp1;txn;kctd11;cdkn1a;fgf7;btg4;rps27;emp1;mdm2;sox9;lifr;pdap1;notch2;cd47;cd164;dhps;ehf;bub1b;ereg;il12rb1;pds5b;smo;fgb;irf2;ing4;dlg3;cks1b;foxp3 |
| regulation of biological quality; | 114#953 | 3.4E-11 | srf;phf17;nck1;atp1a1;corin;slc22a4;gna15;scube1;vwf;dld;ccr4;cxcl12;gck;slc34a3;ntf3;tbrg1;gna11;lep;abca12;mst1;snca;egln1;sftpb;tbxas1;pth;f2rl3;adamts13;emp3;morf4l1;prkca;taok2;txndc8;mkks;brd8;sertad3;adrb1;pln;fth1;atp1a2;nts;hp;cdh23;tpt1;avil;ptprc;bmp10;capg;cav2;tnni3;brms1l;slc9a3;ccr3;armc10;ednra;p2ry1;nedd9;arhgap5;mall;nptn;edn1;f2rl1;chpt1;cav1;proc;ppt1;mmrn1;sphk1;pcdh15;thbd;als2;sbf2;txnrd3;gckr;tnn;igfbp2;gap43;pde3b;yeats4;arpc3;slc34a1;bcar1;ndrg4;socs7;pabpc1;slc9a1;txn;pdia6;adipoq;bard1;socs6;kctd11;cdkn1a;emp1;cda;hexb;elavl1;angptl3;napg;ace;ccl19;notch2;cd320;avpr1a;gcgr;fgb;cutc;nppb;gal3st1;sptbn2;ush1c;dbn1;ccr9;foxp3;clcn6 |
| M phase of mitotic cell cycle; | 43#245 | 6.55E-11 | mapre1;sirt2;tpx2;nudc;spc25;cetn3;ccng1;cdc25c;cdc25b;rad21;pes1;anapc11;tubb3;clasp1;katnb1;ndc80;cetn1;pbrm1;chmp1a;yeats4;cdc23;spc24;ttn;mphosph6;nek3;txnl4b;ccna1;map2k1;nek9;ccna2;aspm;coro1a;kif23;bub1b;ereg;dlg1;pds5b;sycp3;dsn1;aurkb;atm;mad2l1;nedd9 |
| cell-cell signaling; | 84#640 | 8.94E-11 | chrm1;faslg;sdcbp;nmur1;mertk;ccl27;il15;nptx2;aldh5a1;cxcl12;gck;sstr2;lamb2;furin;ntf3;gna11;tph1;mylk2;lep;fgf1;snca;kcnma1;pth;stx1b;stx1a;kif5a;amph;cd97;gata3;wnt4;nmb;akap9;barx1;grb10;il17c;gad2;cav2;mog;gpr56;cplx3;pcdhb2;gdf5;strn4;nptn;edn1;ncam1;wnt3a;ppt1;ptgir;als2;atxn3;tnfrsf11a;lhb;htr3a;sbf2;gria1;pde3b;ide;cplx1;wnt9a;cd33;gabrr3;txn;vipr2;sema4f;fgf7;cntnap2;grik5;hexb;kcnip1;nqo1;pcdhb11;scn11a;wnt8b;ereg;chat;mtnr1b;smo;hap1;ik;pcdhb14;gal3st1;dbn1;npbwr1 |
| mitosis; | 42#239 | 1.09E-10 | mapre1;sirt2;tpx2;nudc;spc25;cetn3;ccng1;cdc25c;cdc25b;rad21;pes1;anapc11;tubb3;clasp1;katnb1;ndc80;cetn1;pbrm1;chmp1a;yeats4;cdc23;spc24;ttn;nek3;txnl4b;ccna1;map2k1;nek9;ccna2;aspm;coro1a;kif23;bub1b;ereg;dlg1;pds5b;sycp3;dsn1;aurkb;atm;mad2l1;nedd9 |
| biopolymer metabolic process; | 633#7940 | 1.53E-10 | pigq;sacs;exosc10;galnt1;map3k12;tead4;fbxo10;usp30;rab18;timm50;znf648;myo3a;psmc2;znf566;furin;tbrg1;farsb;znf300;l3mbtl2;adamts13;znf275;larp7;znf187;zfpm2;psmf1;znf644;tec;psmb6;arih1;nr2e1;notch1;rxrg;phkg2;poln;pop5;myod1;il27ra;glis2;pstk;hoxc10;leo1;csk;cav2;rad54b;vsx1;mef2b;mmp7;hars2;zdhhc1;sox14;wdr5b;endog;mapk10;mael;ung;rfc3;pou5f2;myog;znf32;psenen;aff4;dmtf1;taf1b;nr1d1;pnrc2;upf1;pou1f1;setd7;socs6;rdbp;dhx35;edf1;cherp;mtmr2;pbx4;mars;rho;nr4a1;adck1;insrr;rps14;tcf15;fbxl7;foxp3;chrm1;phf17;ddx1;plod3;dyrk3;traf6;asb17;pqbp1;irf5;sdf2;gck;st6galnac1;flt3;b3gat1;znf280d;rpl6;cdc23;stk38l;phkg1;morf4l1;zscan2;zbtb32;suv420h1;mms19;pcbp2;gadd45a;glt25d2;sf3a1;atg4c;stat3;prpf3;prdm15;dalrd3;fancb;znf629;brms1l;limk2;cbfa2t2;padi6;arid4b;mlxipl;smad5;sirt2;pomgnt1;mycl1;npas2;mapkapk2;usp7;gbx1;hoxd8;usp10;znf576;jazf1;sf3b3;igsf10;wwp2;klhl3;znf713;gtf2i;mcm10;socs7;pabpc1;prpf40a;fut10;sox13;taf1a;hoxb4;dr1;znf329;ppfibp2;znf555;tsr2;mast3;notch2;trip11;utp18;znf81;bub1b;fam83d;trmu;mmp11;med6;sphar;usp53;znf672;nek10;lars;zfp37;znf212;pogk;gna15;gys2;epha5;gna11;yaf2;gpaa1;foxr1;sh3rf2;ttf1;znf34;hipk4;tox3;ppm1g;map2k1;taok2;tcf25;gata3;sart3;asb16;znf470;rab8b;znf683;ulk3;znf142;fen1;znf624;loxl1;gtf2h2;manba;atg7;rabggta;lrsam1;znf250;cpsf1;usp46;tbx10;dph3;sp2;tsen2;dyrk2;il22ra2;znf202;pbrm1;srpk2;gcnt3;dmrtb1;gucy2c;cdkl2;ehf;ereg;znf22;lipc;mpdu1;ripk1;srf;tfeb;alpk1;wwp1;nkx1-2;notch4;elavl2;tsn;prkacb;polr2f;phf20;rad21;zkscan1;hsf4;rpp14;nudt1;usp2;papolb;ube3b;stk33;psip1;foxd4l1;map2k2;stambp;rnase6;ubl7;taok3;sae1;nkx2-6;trim32;txk;qtrtd1;parp1;ezh1;hat1;ptprh;ptprc;isy1;use1;cercam;foxd2;tbr1;zhx2;htatip2;alg5;mto1;ephb6;pigs;nfatc1;ssh1;ssh2;fyb;rffl;trim28;dhx38;suv39h1;rcbtb1;polr3g;yeats4;tep1;scap;trmt6;dnajc13;lrrk2;mlh3;atg12;eif4g3;mdm2;ccdc59;smarce1;elavl1;ppig;zbtb7a;dhps;cry2;ppme1;sycp3;mgat2;atm;lsm10;trex1;dusp12;mcm6;ptk2;zfp62;ppil3;mertk;snrpa;rnf34;b3gnt3;trim29;ears2;stk25;psen1;arid1b;pick1;clk4;epha7;ttn;glyctk;mdc1;vps72;rbms2;lhx3;rars;taf5;znf181;asf1a;wdr33;ptpn18;nom1;ublcp1;prmt2;zbtb5;ncoa3;fbxo15;brca1;tk1;usp35;ptpn4;cdc25c;sp100;cecr2;nkx2-5;sbf2;pfdn5;mtmr7;ecd;gne;smg5;psmb4;znf532;carhsp1;foxj3;ppm1f;nek3;txnl4b;supt3h;pik3c2a;fbxo43;gal3st3;nfyb;znf529;arntl2;bcl11b;sox9;etv2;ppef2;fbxl3;rfc1;usp48;recql;prkar2b;irf2;ing4;uevld;nr1h4;phb2;gsk3b;gtf2e1;cdkl3;thoc3;anapc11;znf641;pax3;msc;spcs3;ubqln4;hivep1;ulk2;lcat;dbp;thrsp;b3galt5;rps6kb1;prkca;smarcad1;stk24;sertad3;cpa4;fbxw11;lzts1;znf667;senp3;znf584;nudt21;b3gnt2;znf175;ftsj2;otud7b;tle4;znf710;dpf3;csnk1g2;e2f6;dclk1;ttll2;mycbp2;gaa;zkscan5;usp26;fanca;ncoa1;ppm1m;ighmbp2;mapk14;rpp38;cd37;atrip;hoxb13;ube2d3;med18;nrbf2;pitx2;fut11;isl2;mus81;pola2;ptp4a3;malt1;mylip;nek9;med11;ptpn3;gtf3a;adamts14;rbm17;znf646;pigz;usp45;irak1;aurkb;msh2;pop1;adprhl1;tbk1;alg2;foxn1;znf524;qars;znf35;dusp7;mylk2;chmp1a;evpl;znf345;lnx1;grhl3;ilkap;rad52;znf23;ppp1r2;smarcb1;brd8;fbxo8;nr0b2;rab35;aarsd1;map3k1;tert;herc1;urm1;noto;vrk1;st6gal2;grk4;znf688;znf395;casp8ap2;nr4a3;usp49;zbtb3;crnkl1;smarcd2;ptpn1;rps6kb2;ercc3;irak2;gabpa;parp4;rbck1;tceb1;rfx2;ube2b;atxn3;uimc1;bmpr1a;prpf4b;znf16;vprbp;hnrnpl;fut3;znf569;yes1;pou2f3;rnf149;dapk2;pias4;skiv2l2;mat2b;asb1;mif4gd;jdp2;gcnt4;psen2;eya2;lcmt1;obscn;gtf2h5;znf770;rfc5;hes5;rnf216;ephb2;ilf3;zmiz2;klf8;clp1;znf238;msh5;rgr;rbak;gal3st1;slu7;shprh;nfyc;pcgf5;dis3;hivep2;meox1;pkn3;poll;ptprr;tfdp2;camk1;pnpt1;oasl;pars2;snapc4;aire;sirt6;mef2d;hoxc8;erg;dnase1l2;ubqlnl;prrx2;foxn2;exosc7;rnf111;pou5f1;zmat5;gypc;barx1;rplp1;prmt7;esco2;pcmtd1;cask;krt7;efcab6;brca2;cog2;terf2;elp4;nhej1;eef1a2;usp29;ncoa6;sec11a;chek1;exosc8;pigb;ppt1;cdc25b;nup98;pold2;trim23;crx;ddx41;nid1;nr1h3;pou2f2;hnrnpr;atf2;gan;sgk2;med24;atg3;psma6;polr3d;bard1;bmp6;ulk4;gzf1;smurf2;msh4;nab1;snrpd3;mmp19;rere;nars;atf5;etv1;ssbp1 |
| negative regulation of metabolic process; | 62#436 | 9.31E-10 | sirt2;wwp1;edn1;timp1;paip2b;jazf1;uimc1;gck;nkx2-5;apc;furin;clasp1;serpina5;katnb1;sirt6;yaf2;znf202;chmp1a;atpif1;ttf1;znf345;pdzd3;pias4;bard1;adipoq;scap;asb1;gzf1;cdkn1a;zbtb32;prkca;vps72;smurf2;nr0b2;dr1;tcf25;mdm2;cda;sox9;nab1;smarce1;stat3;zbtb7a;rps14;eif4ebp1;ilf3;map2;glis2;avil;ereg;capg;irf2;brca2;ing4;znf238;sptbn2;zhx2;foxp3;phb2;nr1h4;e2f6;brca1 |
| localization#establishment of localization; | 358#4135 | 1.89E-09 | slc24a5;btbd10;slc8a1;armc1;rab18;timm50;col23a1;slc35a5;arsb;kcnab1;atp5s;tbrg1;abca12;chmp7;lrp10;pick1;c1qc;scn2a;trpv2;slc39a12;atp6v1e2;mitd1;gltp;sytl3;rbp2;syt17;slc45a3;wdr33;cacng7;slc17a7;apod;tpt1;dnhd1;cav2;rims4;slc9a3;ednra;slc31a2;myh4;edn1;tomm40;cecr2;slc35f4;htr3a;cog6;upf1;gckr;atp1b2;cplx1;kif26b;smg5;pou1f1;slc1a4;atp10b;slc9a1;lrp1;pdzd3;rtp3;kctd18;canx;tomm34;syt2;snx27;cpt1b;sec24a;zdhhc17;anp32a;tmed10;unc13b;slc26a3;scn11a;slc25a36;pkd2l1;vps28;slc25a6;sec61g;tm9sf2;hcn1;uevld;emid2;c1qtnf5;tpd52;foxp3;clcn6;gsk3b;rab38;sys1;adora2b;rbp4;bet3l;aqp1;slc25a1;col5a1;kcna7;thoc3;rrbp1;pitpnm3;syt9;gck;slc34a3;sv2a;emid1;fabp6;col27a1;cacna1i;snca;kcnma1;stx1b;fxc1;cdc23;tcn1;asgr2;kcnj10;tom1;grhpr;lcat;stx11;prkca;rab17;pitpnb;asgr1;ppbp;cacnb4;atp1a2;sec61a1;slc4a10;sfxn1;kdelr2;ap1s3;slc22a20;slc13a5;col12a1;rin2;stard5;mbtps1;vps54;atg4c;cog7;tmem48;vps41;dynlrb2;akap7;tram1;hbq1;snap29;pex19;dclk1;aftph;ccdc91;slc22a16;slc39a13;nup153;kif1a;actr1a;als2;osbpl2;kif3b;tacr2;clcn5;chmp4a;pex13;grin2c;slc6a19;gabrr3;pola2;stx18;fut10;slc25a22;adipoq;malt1;steap3;kif18a;gga1;abcb8;p2rx4;abca5;osbpl7;napa;clpx;nppb;abcf2;atp6v1a;ift88;sdcbp;atp1a1;aaas;slc35b3;slc20a1;scnn1a;svop;pdzk1;slc2a6;slc26a9;slc28a3;aqp8;chmp1a;stx1a;epn2;kif5a;amph;trip10;taok2;arl6ip1;rab35;pln;casc3;atp10a;fth1;rab8b;slc25a13;coro1a;clcn2;slc25a12;pycard;slc7a14;abcc5;cplx3;sh3gl2;slc6a12;atg7;lrsam1;rpl23;rab7l1;slc11a1;cav1;vamp4;kcnj11;parp4;dph3;kif27;sar1a;katnb1;atp6v0b;slc25a34;yes1;slc34a1;ppy;nup210;txn;slc7a13;kctd11;atp4a;lat;kcnk6;mapk8ip3;vps37a;grik5;atp5b;cacng3;slc37a3;sh3bp4;tmem38a;lrp1b;lipc;ap4s1;znf238;atp5f1;sptbn2;kif13b;osbpl5;slc3a1;nmur1;tom1l2;duoxa2;homer3;slc22a4;slc16a14;gopc;fabp3;camk1;rab3d;tubb3;abca3;mcfd2;slc12a3;rtp4;stx16;aqp3;syt6;kctd16;rbp3;atp5g2;snx21;slc39a11;cacng1;nfkbie;kcnq1;abca7;ipo8;akap9;slc30a10;abcc9;slc18a1;best1;slc22a14;use1;hspd1;nup160;cog2;stx2;slc16a1;lrp3;htatip2;abcg5;col25a1;ppt1;nup98;fyb;rffl;trappc1;ucp3;slco4a1;arcn1;ttyh1;p2rx2;slc5a9;kctd10;tnpo3;ehd1;gria1;tspan18;pde3b;atg3;slc35f2;slc1a5;lman2;sypl2;stxbp1;arf4;mapk8ip1;bard1;apba3;csn2;slc25a37;slco2b1;sdad1;folr2;alb;slc35f3;col8a2;nacad;gabrp;kcnip1;angptl3;napg;sec13;cacna2d2;rere;kif23;cry2;slc13a4;sec31a;slc19a1;nod2;chmp6;gosr1;slco4c1;wdr19 |
| lipid metabolic process; | 109#946 | 1.89E-09 | pigq;abhd5;acot9;sgpp1;soat1;cpt1c;acaca;corin;pla2g12a;srd5a3;pitpnm3;fabp3;aldh5a1;hsd17b11;elovl7;serinc2;fabp6;lep;baat;ugcg;snca;gpaa1;sftpb;tbxas1;lrp10;fdft1;rbp3;lcat;hsd17b12;thrsp;smpd1;ptgis;flrt2;nr0b2;pitpnb;sgms2;agpat4;glt25d2;stard5;pla2g2c;mbtps1;apod;ddhd2;sptlc1;lypla2;hmgcs1;slc27a3;cercam;plch2;pnpla4;brca1;gnpat;a4galt;hsd17b2;cyp11a1;aldh3a2;plcg1;chpt1;pigs;pigb;pafah2;ppt1;sphk1;sult1b1;osbpl2;pafah1b2;ucp3;sbf2;mtmr7;pisd;gne;znf202;pex13;acsl1;pnliprp3;lrp1;rarres2;proca1;adipoq;scap;bmp6;acsl5;pik3c2a;edf1;idi1;pla2g4b;pnpla8;cpt1b;hexb;acads;mtmr2;acaa2;plcl2;angptl3;plcb3;hsd11b1;acp6;pla2g12b;dhrs3;osbpl7;plcd4;pigz;impa1;lipc;ltc4s;lypla1;gal3st1;nr1h4;osbpl5 |
| mitotic cell cycle; | 50#326 | 1.95E-09 | mapre1;sirt2;tpx2;nudc;chek1;spc25;cetn3;ccng1;cdc25c;cdc25b;sphk1;rad21;pes1;anapc11;tubb3;clasp1;katnb1;ndc80;cetn1;pbrm1;chmp1a;yeats4;cdc23;spc24;ttn;ilkap;mphosph6;nek3;txnl4b;ccna1;cdkn1a;map2k1;btg4;nek9;ccna2;aspm;coro1a;kif23;bub1b;ereg;dlg1;pds5b;sycp3;dsn1;aurkb;brca2;atm;mad2l1;ush1c;nedd9 |
| nervous system development; | 88#716 | 2.5E-09 | chrm1;naglu;wwp1;ntng2;pcdha1;notch4;camk1;aldh5a1;ccr4;tubb3;sema4c;pax3;ntf3;dscam;naip;hoxc8;snca;pick1;cxcl1;nrcam;smpd1;gpr45;map2k1;bdnf;gata3;b3gnt2;nr2e1;stat3;fzd6;glis2;avil;cav2;mog;brca2;gpr56;sh3gl2;tbr1;dclk1;pcdhb2;sox14;nhej1;cyp11a1;npas2;nptn;aldh3a2;ncoa6;gfra3;wnt3a;ppt1;als2;atxn3;chl1;nrsn1;rtn4;dock7;sbf2;tnn;pex13;gap43;serpini1;pou1f1;fez2;fut10;kctd11;sema4f;btg4;mylip;cherp;hexb;pbx4;hes5;notch2;pcdhb11;ephb2;napa;wnt8b;snai1;smo;rufy3;hpcal4;gda;ahnak;pcdhb14;gal3st1;dbn1;wfs1;olfm1;msi1 |
| response to external stimulus; | 80#633 | 3.34E-09 | ly96;cx3cr1;cfb;s100a8;scube1;ccl27;ltb4r2;vwf;ccr4;cxcl12;sstr2;lep;mst1;tbxas1;chmp1a;c1qc;adamts13;f2rl3;cxcl1;prkca;map2k1;itgal;mkks;pglyrp1;cd97;ppbp;lct;c8b;tlr9;cmtm8;mefv;il16;aoc3;c4a;stat3;masp1;il17c;brca2;ccr3;p2ry1;cyp11a1;cfi;f2rl1;irak2;abcg5;tlr3;mapk14;proc;mmrn1;parp4;atrn;thbd;aldob;hoxb13;mmp25;gap43;ptafr;tlr5;cmtm7;tlr6;fut10;il18rap;cmtm4;bmp6;cdkn1a;fgf7;alb;arntl2;pla2g4b;acads;rho;nod1;ccl19;avpr1a;ereg;gcgr;fgb;nod2;rgr;ccr9 |
| response to stress; | 132#1222 | 3.34E-09 | trex1;ly96;cfb;s100a8;sod3;stk25;ccr4;cxcl12;mst1;egln1;c1qc;adamts13;cxcl1;rad52;mdc1;map2k1;taok2;adrb1;rif1;tlr9;wdr33;asf1a;c4a;poln;masp1;il17c;vapb;rad54b;fen1;cideb;ccr3;brca1;cyp11a1;gtf2h2;cfi;rfc3;ung;edn1;ercc3;irak2;proc;parp4;ube2b;aldob;thbd;atxn3;mmp25;upf1;apc;dnaja1;dyrk2;gap43;ptafr;tlr6;il18rap;dnajb5;gtf2h5;ccna2;rfc5;nod1;ccl19;avpr1a;recql;ereg;crnn;msh5;gsk3b;phf17;shprh;cx3cr1;scube1;poll;vwf;rad21;hsf4;tbxas1;nudt1;f2rl3;prkca;mms19;itgal;cd97;c8b;lct;mefv;gadd45a;esco2;parp1;stat3;aoc3;avil;hspb9;hspd1;fancb;brca2;p2ry1;nhej1;sirt2;ncoa6;fanca;f2rl1;chek1;tlr3;mapk14;ighmbp2;atrn;mmrn1;atrip;hoxb13;sfn;als2;duox2;sgk2;ndrg4;tlr5;mus81;fut10;bard1;bmp6;scap;fgf7;cdkn1a;mlh3;alb;msh4;pla2g4b;ndufs8;acads;cry2;fgb;msh2;atm |
| negative regulation of cellular metabolic process; | 55#381 | 5.45E-09 | sirt2;wwp1;edn1;timp1;paip2b;jazf1;uimc1;gck;nkx2-5;furin;serpina5;sirt6;yaf2;znf202;chmp1a;atpif1;ttf1;znf345;pdzd3;pias4;bard1;adipoq;scap;asb1;gzf1;cdkn1a;zbtb32;prkca;vps72;smurf2;nr0b2;dr1;tcf25;mdm2;cda;sox9;nab1;smarce1;stat3;zbtb7a;rps14;eif4ebp1;ilf3;glis2;ereg;irf2;brca2;ing4;znf238;zhx2;foxp3;phb2;nr1h4;e2f6;brca1 |
| neurological system process#transmission of nerve impulse; | 49#330 | 1.51E-08 | chrm1;sdcbp;strn4;nmur1;nptn;edn1;ncam1;ppt1;nptx2;aldh5a1;atxn3;als2;htr3a;lamb2;sbf2;ntf3;gna11;tph1;mylk2;gria1;snca;cplx1;kcnma1;stx1b;gabrr3;stx1a;kif5a;amph;cntnap2;gata3;grik5;hexb;akap9;kcnip1;nqo1;pcdhb11;scn11a;gad2;chat;cav2;mtnr1b;mog;hap1;pcdhb14;cplx3;gal3st1;npbwr1;dbn1;pcdhb2 |
| transport; | 345#4035 | 2.13E-08 | slc24a5;btbd10;slc8a1;armc1;rab18;timm50;col23a1;slc35a5;arsb;kcnab1;atp5s;tbrg1;abca12;chmp7;lrp10;pick1;c1qc;scn2a;trpv2;slc39a12;atp6v1e2;mitd1;gltp;sytl3;rbp2;syt17;slc45a3;wdr33;cacng7;slc17a7;apod;tpt1;dnhd1;cav2;rims4;slc9a3;ednra;slc31a2;myh4;edn1;tomm40;cecr2;slc35f4;htr3a;cog6;upf1;gckr;atp1b2;cplx1;kif26b;smg5;pou1f1;slc1a4;atp10b;slc9a1;lrp1;pdzd3;rtp3;kctd18;tomm34;syt2;snx27;cpt1b;sec24a;zdhhc17;anp32a;tmed10;unc13b;slc26a3;scn11a;slc25a36;pkd2l1;vps28;slc25a6;sec61g;tm9sf2;hcn1;uevld;emid2;c1qtnf5;clcn6;gsk3b;rab38;sys1;rbp4;bet3l;aqp1;slc25a1;col5a1;kcna7;thoc3;rrbp1;pitpnm3;syt9;gck;slc34a3;sv2a;emid1;fabp6;col27a1;cacna1i;snca;kcnma1;stx1b;fxc1;tcn1;asgr2;kcnj10;tom1;lcat;stx11;prkca;rab17;pitpnb;asgr1;ppbp;cacnb4;atp1a2;sec61a1;slc4a10;sfxn1;kdelr2;ap1s3;slc22a20;slc13a5;col12a1;rin2;stard5;mbtps1;vps54;atg4c;cog7;tmem48;vps41;dynlrb2;akap7;tram1;hbq1;snap29;pex19;dclk1;aftph;ccdc91;slc22a16;slc39a13;nup153;kif1a;actr1a;als2;osbpl2;kif3b;clcn5;chmp4a;pex13;grin2c;slc6a19;gabrr3;pola2;stx18;fut10;slc25a22;adipoq;malt1;steap3;kif18a;gga1;abcb8;p2rx4;abca5;osbpl7;napa;clpx;abcf2;atp6v1a;sdcbp;atp1a1;aaas;slc35b3;slc20a1;scnn1a;svop;pdzk1;slc2a6;slc26a9;slc28a3;aqp8;chmp1a;stx1a;epn2;kif5a;amph;trip10;taok2;arl6ip1;rab35;pln;casc3;atp10a;fth1;rab8b;slc25a13;coro1a;clcn2;slc25a12;slc7a14;abcc5;cplx3;sh3gl2;slc6a12;atg7;lrsam1;rpl23;rab7l1;slc11a1;cav1;vamp4;kcnj11;parp4;kif27;sar1a;katnb1;atp6v0b;slc25a34;yes1;slc34a1;nup210;txn;slc7a13;kctd11;atp4a;lat;kcnk6;mapk8ip3;vps37a;grik5;atp5b;cacng3;slc37a3;sh3bp4;tmem38a;lrp1b;lipc;ap4s1;znf238;atp5f1;sptbn2;kif13b;osbpl5;slc3a1;nmur1;tom1l2;duoxa2;homer3;slc22a4;slc16a14;gopc;fabp3;camk1;rab3d;tubb3;abca3;mcfd2;slc12a3;rtp4;stx16;aqp3;syt6;kctd16;rbp3;atp5g2;snx21;slc39a11;cacng1;nfkbie;kcnq1;abca7;ipo8;akap9;slc30a10;abcc9;slc18a1;best1;slc22a14;use1;hspd1;nup160;cog2;stx2;slc16a1;lrp3;htatip2;abcg5;col25a1;ppt1;nup98;fyb;rffl;trappc1;ucp3;slco4a1;arcn1;ttyh1;p2rx2;slc5a9;kctd10;tnpo3;ehd1;gria1;tspan18;pde3b;atg3;slc35f2;slc1a5;lman2;sypl2;stxbp1;arf4;mapk8ip1;bard1;apba3;csn2;slc25a37;slco2b1;sdad1;folr2;alb;slc35f3;col8a2;nacad;gabrp;kcnip1;angptl3;napg;sec13;cacna2d2;rere;kif23;cry2;slc13a4;sec31a;slc19a1;chmp6;gosr1;slco4c1;wdr19 |
| macromolecule metabolic process; | 833#11144 | 2.16E-08 | pigq;sacs;exosc10;galnt1;naglu;map3k12;tead4;fbxo10;cfb;usp30;rab18;timm50;znf648;myo3a;psmc2;znf566;arsb;furin;tbrg1;mst1;farsb;znf300;l3mbtl2;adamts13;znf275;larp7;znf187;zfpm2;psmf1;znf644;tec;suclg1;psmb6;arih1;nr2e1;notch1;rxrg;c4a;phkg2;poln;pop5;myod1;il27ra;eif4ebp1;masp1;glis2;pstk;hoxc10;leo1;csk;cav2;rad54b;vsx1;mef2b;mmp7;hars2;zdhhc1;sox14;endog;wdr5b;mapk10;mael;ung;rfc3;pou5f2;myog;znf32;psenen;cpb2;aff4;dmtf1;taf1b;nr1d1;pnrc2;upf1;rpl8;pou1f1;pdia6;setd7;socs6;rdbp;dhx35;edf1;cherp;scrn3;mtmr2;pbx4;mars;rho;nr4a1;adck1;ace;insrr;rps14;tcf15;fbxl7;foxp3;chrm1;phf17;ddx1;man2b2;plod3;dyrk3;traf6;mrpl43;rrbp1;asb17;pqbp1;irf5;sdf2;gck;st6galnac1;flt3;b3gat1;znf280d;rpl6;khk;fkbp2;cdc23;stk38l;phkg1;fuca2;morf4l1;adam7;zscan2;zbtb32;suv420h1;l2hgdh;mms19;gk;pglyrp1;pcbp2;lct;gadd45a;glt25d2;adam21;sf3a1;atg4c;stat3;rps5;gad2;avil;prpf3;prdm15;dalrd3;fancb;znf629;brms1l;limk2;cbfa2t2;padi6;arid4b;lepre1;mlxipl;smad5;sirt2;pomgnt1;mycl1;npas2;mapkapk2;usp7;gbx1;hoxd8;usp10;mmp24;als2;znf576;jazf1;sf3b3;mrpl17;igsf10;wwp2;klhl3;pex13;amdhd2;ide;il19;arpc3;znf713;gtf2i;mcm10;socs7;pabpc1;prpf40a;fut10;adipoq;sox13;taf1a;hoxb4;dr1;gga1;adam33;znf329;ppfibp2;znf555;mrpl9;tsr2;mast3;notch2;trip11;utp18;znf81;bub1b;fam83d;mapk8ip2;hk1;trmu;mmp11;med6;clpx;sphar;usp53;znf672;ift88;nek10;rpl5;lars;prepl;mrpl30;zfp37;corin;znf212;pogk;gna15;cpa5;fkbp10;gys2;epha5;gna11;yaf2;egln1;gpaa1;foxr1;tpp2;sh3rf2;ttf1;znf34;hipk4;tox3;ppm1g;map2k1;taok2;tcf25;gata3;sart3;pofut2;asb16;znf470;rab8b;znf683;hp;hibadh;pycard;ulk3;znf142;fen1;znf624;loxl1;gtf2h2;nrip2;manba;atg7;rabggta;lrsam1;znf250;vamp4;cpsf1;usp46;aldob;traf1;tbx10;dph3;sp2;tsen2;kiaa1109;dyrk2;il22ra2;znf202;pbrm1;tlr6;srpk2;gcnt3;dmrtb1;gucy2c;cdkl2;nod1;klk9;ehf;tasp1;ereg;tubg1;cct6b;znf22;pxn;glb1l;lipc;ap4s1;sptbn2;mpdu1;ripk1;slc3a1;srf;abhd5;uap1;tfeb;alpk1;wwp1;nkx1-2;notch4;scube1;elavl2;tsn;prkacb;polr2f;glt6d1;phf20;rad21;hs3st3b1;zkscan1;hsf4;lep;rpp14;nudt1;usp2;papolb;ube3b;stk33;psip1;foxd4l1;map2k2;stambp;rnase6;ubl7;taok3;pfdn1;ipo8;lyg2;sae1;nkx2-6;trim32;txk;qtrtd1;parp1;ezh1;hat1;rps23;ptprh;ptprc;isy1;use1;cercam;hspd1;foxd2;tbr1;zhx2;htatip2;alg5;mto1;ephb6;pigs;nfatc1;ssh1;paip2b;ssh2;fyb;rffl;trim28;dhx38;arcn1;suv39h1;rcbtb1;polr3g;yeats4;tep1;scap;trmt6;dnajc13;lrrk2;mlh3;atg12;eif4g3;mdm2;ccdc59;smarce1;elavl1;napg;ppig;zbtb7a;dhps;mtfmt;cry2;ppme1;lgmn;sycp3;impa1;fgb;mgat2;atm;nod2;adamts16;lsm10;trex1;ptk2;dusp12;mcm6;zfp62;mertk;ppil3;snrpa;rnf34;b3gnt3;fmod;trim29;ears2;stk25;adamts17;psen1;arid1b;rpl28;pick1;epha7;c1qc;clk4;ttn;itpka;rps9;glyctk;mdc1;vps72;rbms2;rce1;lhx3;rars;taf5;tlr9;znf181;rpl12;asf1a;wdr33;cldn14;ptpn18;ublcp1;nom1;sept11;prmt2;zbtb5;capg;ncoa3;fbxo15;brca1;tk1;usp35;ptpn4;me3;cdc25c;sp100;actn2;cecr2;bbs10;napsa;apc;nkx2-5;sbf2;pfdn5;mtmr7;ecd;dnaja1;gne;casp8;smg5;psmb4;znf532;carhsp1;foxj3;ppm1f;nek3;txnl4b;supt3h;pik3c2a;fbxo43;gal3st3;nfyb;canx;znf529;arntl2;bcl11b;sox9;hexb;mrpl24;etv2;ppef2;ganab;fbxl3;rfc1;map2;usp48;prkar2b;recql;rps3a;wdr7;irf2;gfpt2;ing4;uevld;phb2;nr1h4;gsk3b;rpl18a;gtf2e1;nck1;thoc3;cdkl3;vwf;timp1;anapc11;aldh5a1;znf641;pax3;clasp1;msc;serpina5;dnajc17;spcs3;astl;galnt5;ubqln4;hivep1;ulk2;lcat;dbp;thrsp;b3galt5;prkca;rps6kb1;clgn;cpa4;smarcad1;stk24;sertad3;fbxw11;lzts1;znf667;senp3;rpl39;c8b;znf584;ap1s3;lctl;nudt21;b3gnt2;znf175;ftsj2;ebi3;mbtps1;otud7b;tmem48;dag1;tle4;znf710;csnk1g2;dpf3;dclk1;e2f6;ttll2;mycbp2;gaa;zkscan5;usp26;fanca;ncoa1;ppm1m;ighmbp2;mapk14;rpp38;cd37;hoxb13;atrip;ube2d3;med18;nrbf2;pitx2;fut11;mrps5;isl2;mus81;pola2;ptp4a3;malt1;mylip;mrpl49;nek9;med11;ndufs8;ptpn3;gtf3a;adamts14;pum2;rbm17;znf646;usp45;pigz;irak1;aurkb;msh2;pop1;adprhl1;mrpl11;tbk1;idua;mapre1;cpd;alg2;gk2;foxn1;znf524;lonrf3;qars;znf35;cxcl12;dusp7;sept7;mylk2;klk11;evpl;chmp1a;fkbp5;znf345;lnx1;grhl3;ilkap;rad52;znf23;ppp1r2;smarcb1;brd8;fbxo8;nr0b2;aarsd1;rab35;adamts8;map3k1;tert;herc1;noto;urm1;chst3;prss21;vrk1;st6gal2;ldha;grk4;znf688;znf395;casp8ap2;nr4a3;cenpj;usp49;tmprss12;zbtb3;ptpn1;smarcd2;crnkl1;rps6kb2;cfi;rpl23;ercc3;irak2;gabpa;proc;parp4;ube2b;rfx2;tceb1;rbck1;atxn3;uimc1;mmp25;bmpr1a;katnb1;prpf4b;znf16;vprbp;hnrnpl;fut3;atpif1;znf569;yes1;pou2f3;dapk2;rnf149;eif4a1;pias4;mat2b;skiv2l2;mif4gd;asb1;jdp2;eya2;psen2;gcnt4;dnajb5;lcmt1;obscn;cda;gtf2h5;rps15a;znf770;rfc5;hes5;rnf216;ilf3;ephb2;zmiz2;klf8;clp1;znf238;msh5;rgr;rbak;gal3st1;pgk2;slu7;shprh;nfyc;pcgf5;dis3;hivep2;rpl7l1;meox1;pkn3;abtb1;eif2c3;gopc;ptprr;poll;tfdp2;camk1;pnpt1;pars2;oasl;prss22;tubb3;snapc4;aire;eif5a;sirt6;mef2d;hoxc8;crbn;erg;rbp3;gba3;dnase1l2;ubqlnl;prrx2;foxn2;exosc7;rnf111;pou5f1;lonrf2;nanos3;mkks;sds;zmat5;gypc;pfkp;rplp1;barx1;prmt7;esco2;pcmtd1;cask;krt7;naaladl1;efcab6;brca2;cog2;terf2;mgea5;elp4;nhej1;eef1a2;usp29;ncoa6;sec11a;chek1;capn10;exosc8;pigb;phlda1;tlr3;ppt1;cdc25b;nup98;pold2;trim23;crx;ddx41;nid1;nr1h3;pou2f2;hnrnpr;atf2;gan;sgk2;immp1l;med24;atg3;psma6;polr3d;casp14;bard1;klk14;bmp6;gzf1;ulk4;tmprss9;smurf2;rps27;msh4;nab1;snrpd3;mmp19;angptl3;ext2;rere;tmprss11d;galt;mrps14;nars;atf5;etv1;ssbp1 |
| regulation of progression through cell cycle; | 51#353 | 2.45E-08 | nat6;cdc73;sirt2;htatip2;chek1;ercc3;ccng1;cdc25c;sgsm3;sphk1;atrip;sfn;apc;reck;tbrg1;gas2l3;ppp1r13b;cks2;chmp1a;cdc23;ilkap;bard1;ccna1;kctd11;ppm1g;cdkn1a;stard13;btg4;smarcb1;lzts1;gadd45a;ccna2;ext2;notch2;ephb2;bub1b;ereg;ptprc;dlg1;pycard;msh2;brca2;atm;mad2l1;ing4;xrn1;cks1b;dst;trim13;brca1;rassf4 |
| protein modification process; | 245#2704 | 4.32E-08 | pigq;sacs;nek10;ptk2;dusp12;galnt1;map3k12;alg2;mertk;fbxo10;usp30;rnf34;gna15;timm50;b3gnt3;myo3a;stk25;dusp7;psen1;furin;epha5;gna11;mylk2;gpaa1;evpl;sh3rf2;pick1;epha7;clk4;adamts13;ttn;hipk4;lnx1;ilkap;glyctk;ppm1g;map2k1;taok2;tec;map3k1;herc1;urm1;vrk1;st6gal2;asb16;arih1;grk4;ptpn18;phkg2;ublcp1;myod1;prmt2;csk;cav2;ulk3;usp49;fbxo15;loxl1;zdhhc1;ptpn1;brca1;rps6kb2;wdr5b;mapk10;manba;atg7;rabggta;ptpn4;usp35;lrsam1;irak2;psenen;cdc25c;usp46;parp4;ube2b;rbck1;tceb1;dph3;mtmr7;bmpr1a;dyrk2;il22ra2;prpf4b;vprbp;fut3;yes1;dapk2;rnf149;ppm1f;pias4;socs6;nek3;asb1;fbxo43;srpk2;gcnt3;psen2;gcnt4;lcmt1;obscn;mtmr2;gucy2c;cdkl2;rho;adck1;ppef2;fbxl3;insrr;rnf216;usp48;ephb2;prkar2b;lipc;ing4;rgr;uevld;gal3st1;fbxl7;mpdu1;ripk1;gsk3b;chrm1;shprh;alpk1;wwp1;plod3;dyrk3;traf6;pkn3;asb17;cdkl3;ptprr;prkacb;camk1;sdf2;anapc11;oasl;st6galnac1;flt3;b3gat1;sirt6;spcs3;cdc23;ubqln4;usp2;erg;stk38l;ube3b;stk33;ulk2;ubqlnl;rnf111;lcat;b3galt5;phkg1;map2k2;stambp;prkca;rps6kb1;cpa4;stk24;ubl7;taok3;gypc;fbxw11;senp3;sae1;trim32;prmt7;b3gnt2;txk;pcmtd1;parp1;sf3a1;atg4c;otud7b;cask;hat1;ptprh;ptprc;limk2;cog2;padi6;csnk1g2;dclk1;ttll2;mycbp2;sirt2;usp29;usp26;pomgnt1;sec11a;chek1;mapkapk2;alg5;ephb6;pigs;ppm1m;pigb;usp7;mapk14;ppt1;cdc25b;cd37;usp10;ssh1;ube2d3;ssh2;fyb;rffl;trim23;igsf10;nid1;wwp2;fut11;gan;sgk2;klhl3;atg3;socs7;fut10;bard1;ptp4a3;ulk4;malt1;lrrk2;smurf2;mylip;atg12;nek9;mdm2;ptpn3;mast3;dhps;cry2;bub1b;ppme1;usp45;pigz;irak1;aurkb;mgat2;adprhl1;tbk1 |
| regulation of cell proliferation; | 62#469 | 4.65E-08 | chrm1;lepre1;nck1;edn1;chek1;il15;gas8;fabp3;timp1;cdc25b;sphk1;tnfrsf11a;trim35;sstr2;apc;flt3;hsf4;bmpr1a;tbrg1;fabp6;clec11a;cd33;atpif1;pou1f1;cxcl1;dbp;emp3;kctd11;cdkn1a;stambp;fgf7;btg4;smarcb1;adamts8;mdm2;sox9;fth1;kiss1r;lifr;ifnk;notch2;ebi3;capns1;cd47;cd164;dhps;hoxc10;ereg;csk;ptprc;il12rb1;cav2;smo;pds5b;tob2;fgb;brca2;ing4;dlg3;krt4;foxp3;brca1 |
| regulation of cell cycle; | 51#359 | 5.41E-08 | nat6;cdc73;sirt2;htatip2;chek1;ercc3;ccng1;cdc25c;sgsm3;sphk1;atrip;sfn;apc;reck;tbrg1;gas2l3;ppp1r13b;cks2;chmp1a;cdc23;ilkap;bard1;ccna1;kctd11;ppm1g;cdkn1a;stard13;btg4;smarcb1;lzts1;gadd45a;ccna2;ext2;notch2;ephb2;bub1b;ereg;ptprc;dlg1;pycard;msh2;brca2;atm;mad2l1;ing4;xrn1;cks1b;dst;trim13;brca1;rassf4 |
| cellular localization; | 120#1126 | 6.23E-08 | sdcbp;nmur1;tom1l2;homer3;aaas;slc25a1;rrbp1;timm50;gopc;camk1;rab3d;arsb;tubb3;tbrg1;rtp4;snca;gpaa1;stx16;pick1;kcnma1;stx1b;fxc1;cdc23;stx1a;tom1;stx11;kif5a;taok2;arl6ip1;rab35;nfkbie;sytl3;ipo8;sec61a1;kdelr2;ap1s3;mbtps1;vps54;atg4c;tmem48;vps41;dnhd1;dynlrb2;pycard;tram1;use1;rims4;nup160;hspd1;snap29;cog2;cplx3;pex19;dclk1;stx2;myh4;htatip2;rpl23;nup153;tomm40;kif1a;cd2;nup98;als2;dph3;fyb;rffl;kif27;upf1;trappc1;ucp3;sar1a;kif3b;arcn1;gckr;katnb1;ehd1;pex13;cplx1;smg5;kif26b;pou1f1;pola2;ppy;nup210;stx18;fut10;stxbp1;bard1;rtp3;malt1;slc25a37;kif18a;lat;canx;alb;gga1;tomm34;sec24a;anp32a;tmed10;unc13b;napg;sec13;rere;kif23;slc25a36;napa;cry2;slc25a6;sec61g;sec31a;ap4s1;nod2;gosr1;foxp3;kif13b;osbpl5;wdr19;gsk3b |
| cell death; | 101#908 | 8.97E-08 | phf17;faslg;fadd;sgpp1;traf6;rnf34;rad21;psen1;pax3;ntf3;eif5a;ppp1r13b;naip;snca;cidec;pth;emp3;stambp;prkca;taok2;c8b;siva1;gadd45a;tpt1;unc5a;casp8ap2;ctnnal1;ptprc;pycard;hspd1;brca2;cideb;aven;brca1;eef1a2;gdf5;emp2;sfrp1;api5;htatip2;ercc3;phlda1;proc;ppt1;cd2;parp4;sphk1;sfn;atxn3;actn2;traf1;rtn4;cecr2;rffl;trim35;ddx41;dyrk2;pdcd1;casp8;pacs2;il19;bik;bcar1;dapk2;ppm1f;ift57;bard1;casp14;tnfrsf21;tia1;malt1;cdkn1a;steap3;ctnnbl1;psen2;alb;atg12;ccar1;emp1;sox9;unc13b;nod1;notch2;akt1s1;rnf216;bcl2a1;bub1b;slc25a6;mapk8ip2;rps3a;smo;sycp3;bcl2l13;pdcd10;atf5;nod2;ing4;pik3r2;sptbn2;ripk1;gsk3b |
| death; | 101#908 | 8.97E-08 | phf17;faslg;fadd;sgpp1;traf6;rnf34;rad21;psen1;pax3;ntf3;eif5a;ppp1r13b;naip;snca;cidec;pth;emp3;stambp;prkca;taok2;c8b;siva1;gadd45a;tpt1;unc5a;casp8ap2;ctnnal1;ptprc;pycard;hspd1;brca2;cideb;aven;brca1;eef1a2;gdf5;emp2;sfrp1;api5;htatip2;ercc3;phlda1;proc;ppt1;cd2;parp4;sphk1;sfn;atxn3;actn2;traf1;rtn4;cecr2;rffl;trim35;ddx41;dyrk2;pdcd1;casp8;pacs2;il19;bik;bcar1;dapk2;ppm1f;ift57;bard1;casp14;tnfrsf21;tia1;malt1;cdkn1a;steap3;ctnnbl1;psen2;alb;atg12;ccar1;emp1;sox9;unc13b;nod1;notch2;akt1s1;rnf216;bcl2a1;bub1b;slc25a6;mapk8ip2;rps3a;smo;sycp3;bcl2l13;pdcd10;atf5;nod2;ing4;pik3r2;sptbn2;ripk1;gsk3b |
| establishment of cellular localization; | 117#1098 | 9.81E-08 | sdcbp;nmur1;tom1l2;homer3;aaas;slc25a1;rrbp1;timm50;gopc;camk1;rab3d;arsb;tubb3;tbrg1;rtp4;snca;stx16;pick1;kcnma1;stx1b;fxc1;cdc23;stx1a;tom1;stx11;kif5a;taok2;arl6ip1;rab35;nfkbie;sytl3;ipo8;sec61a1;kdelr2;ap1s3;mbtps1;vps54;atg4c;tmem48;vps41;dnhd1;dynlrb2;pycard;tram1;use1;rims4;nup160;hspd1;snap29;cog2;cplx3;pex19;dclk1;stx2;myh4;htatip2;rpl23;nup153;tomm40;kif1a;nup98;als2;dph3;fyb;rffl;kif27;upf1;trappc1;ucp3;sar1a;kif3b;arcn1;gckr;katnb1;ehd1;pex13;cplx1;smg5;kif26b;pou1f1;pola2;ppy;nup210;stx18;stxbp1;fut10;bard1;rtp3;malt1;slc25a37;kif18a;lat;canx;gga1;tomm34;sec24a;anp32a;tmed10;unc13b;napg;sec13;rere;kif23;slc25a36;napa;cry2;slc25a6;sec61g;sec31a;ap4s1;nod2;gosr1;foxp3;kif13b;osbpl5;wdr19;gsk3b |
| transcription from RNA polymerase II promoter; | 77#640 | 1.21E-07 | srf;nfyc;tead4;foxn1;trim29;tfdp2;polr2f;snapc4;pax3;msc;mef2d;ttf1;znf345;zfpm2;thrsp;dbp;zbtb32;vps72;smarcb1;nr0b2;brd8;tcf25;gata3;taf5;parp1;stat3;myod1;vsx1;mef2b;e2f6;brca1;smarcd2;elp4;smad5;npas2;ncoa6;htatip2;myog;ercc3;aff4;nfatc1;gabpa;tceb1;tbx10;jazf1;sp2;trim28;nkx2-5;crx;ecd;pou2f2;znf202;med24;pou1f1;gtf2i;pou2f3;pias4;supt3h;bmp6;gzf1;taf1a;dr1;mdm2;sox9;smarce1;pbx4;zbtb7a;trip11;tcf15;zmiz2;irf2;med6;atf5;znf238;etv1;nr1h4;gtf2e1 |
| response to chemical stimulus; | 72#589 | 1.7E-07 | cx3cr1;cfb;ccl27;ltb4r2;stk25;gys2;ccr4;pdzk1;abca3;cxcl12;gck;sstr2;rtp4;snca;nudt1;chmp1a;cxcl1;lcat;prkca;map2k1;mms19;ppbp;gata3;lct;cmtm8;il16;slc25a13;slc18a1;slc25a12;vapb;cav2;hspd1;brca2;ccr3;brca1;cyp11a1;sirt2;ncoa6;ercc3;abcg5;fmo4;tlr3;mapk14;kcnj11;parp4;thbd;aldob;als2;duox2;dnaja1;sgk2;ptafr;cmtm7;pdzd3;adipoq;cmtm4;rtp3;dnajb5;ndufs8;snx27;acads;mat2a;kcnip1;nqo1;ccl19;scn11a;bcar3;gcgr;crnn;nod2;ccr9;nr1h4 |
| neurological system process#transmission of nerve impulse#synaptic transmission; | 43#290 | 1.73E-07 | chrm1;sdcbp;strn4;nptn;edn1;ncam1;ppt1;nptx2;aldh5a1;atxn3;als2;htr3a;lamb2;ntf3;tph1;mylk2;gria1;snca;cplx1;kcnma1;stx1b;gabrr3;stx1a;kif5a;amph;gata3;grik5;akap9;kcnip1;nqo1;pcdhb11;scn11a;gad2;chat;cav2;mtnr1b;mog;hap1;pcdhb14;cplx3;dbn1;npbwr1;pcdhb2 |
| ubiquitin cycle; | 68#549 | 2.3E-07 | shprh;wwp1;traf6;fbxo10;usp30;rnf34;asb17;anapc11;sh3rf2;cdc23;usp2;ube3b;lnx1;rnf111;stambp;ubl7;fbxw11;senp3;herc1;urm1;sae1;trim32;asb16;arih1;atg4c;otud7b;usp49;fbxo15;brca1;mycbp2;wdr5b;usp29;usp26;lrsam1;usp35;atg7;usp7;usp46;tceb1;rbck1;ube2b;usp10;ube2d3;rffl;trim23;wwp2;gan;klhl3;vprbp;atg3;rnf149;socs7;pias4;bard1;socs6;asb1;fbxo43;malt1;smurf2;mylip;atg12;mdm2;fbxl3;rnf216;usp48;usp45;uevld;fbxl7 |
| regulation of transcription from RNA polymerase II promoter; | 56#423 | 2.55E-07 | srf;elp4;smad5;nfyc;ncoa6;npas2;htatip2;ercc3;myog;tead4;foxn1;gabpa;tfdp2;tceb1;jazf1;tbx10;sp2;trim28;nkx2-5;crx;znf202;pou1f1;pou2f3;ttf1;znf345;pias4;zfpm2;thrsp;bmp6;supt3h;dbp;gzf1;zbtb32;vps72;smarcb1;nr0b2;brd8;dr1;tcf25;mdm2;sox9;smarce1;pbx4;stat3;zbtb7a;myod1;tcf15;zmiz2;irf2;med6;atf5;znf238;nr1h4;brca1;e2f6;smarcd2 |
| biological adhesion; | 104#960 | 2.6E-07 | nfasc;itga3;pcdhgb4;cx3cr1;pcdha1;pcdhga11;col5a1;izumo1;cd96;vwf;sdk1;pvrl3;cxcl12;psen1;lamb2;dscam;col27a1;icam5;fxc1;cldn17;anxa9;cdh26;adamts13;dsg4;prph2;cntnap4;ly6d;nrcam;flrt2;pcdhga8;taok2;itgal;cd97;col12a1;aoc3;cask;dsg2;cldn14;fzd6;cdh23;cd6;ctnnal1;dlg1;cercam;gpr56;ccr3;lamb4;ly9;dst;plxnc1;nedd9;pcdhb2;arhgap5;nptn;epdr1;pcdhgb5;ncam1;cxadr;pcdhgb6;cd2;mmrn1;pcdh15;chl1;actn2;parvb;apc;nid1;ttyh1;dchs2;gne;tnn;cdh16;cd33;bcar1;cldn4;dsc1;islr;pcdhga1;itgb7;pvrl4;cntnap2;pcdhga5;pkp2;fblim1;sox9;tnc;cdsn;col8a2;angptl3;icam4;nphp4;ctnna3;hes5;pcdhb11;cd47;boc;cd164;snai1;mfge8;pxn;crnn;pcdhb14;pcdhga10;fcgbp |
| cell adhesion; | 104#960 | 2.6E-07 | nfasc;itga3;pcdhgb4;cx3cr1;pcdha1;pcdhga11;col5a1;izumo1;cd96;vwf;sdk1;pvrl3;cxcl12;psen1;lamb2;dscam;col27a1;icam5;fxc1;cldn17;anxa9;cdh26;adamts13;dsg4;prph2;cntnap4;ly6d;nrcam;flrt2;pcdhga8;taok2;itgal;cd97;col12a1;aoc3;cask;dsg2;cldn14;fzd6;cdh23;cd6;ctnnal1;dlg1;cercam;gpr56;ccr3;lamb4;ly9;dst;plxnc1;nedd9;pcdhb2;arhgap5;nptn;epdr1;pcdhgb5;ncam1;cxadr;pcdhgb6;cd2;mmrn1;pcdh15;chl1;actn2;parvb;apc;nid1;ttyh1;dchs2;gne;tnn;cdh16;cd33;bcar1;cldn4;dsc1;islr;pcdhga1;itgb7;pvrl4;cntnap2;pcdhga5;pkp2;fblim1;sox9;tnc;cdsn;col8a2;angptl3;icam4;nphp4;ctnna3;hes5;pcdhb11;cd47;boc;cd164;snai1;mfge8;pxn;crnn;pcdhb14;pcdhga10;fcgbp |
| second-messenger-mediated signaling; | 40#268 | 4.48E-07 | chrm1;adora2b;nmur1;edn1;gna15;ltb4r2;prkacb;ptgir;sphk1;sstr2;rgs1;gna11;ndc80;gap43;cks2;pick1;pth;mc3r;ptafr;f2rl3;pdzd3;mc1r;pik3c2a;oprd1;mctp2;lat;adrb1;pla2g4b;rapgef2;dgki;lhcgr;avpr1a;bub1b;dgkg;mtnr1b;gcgr;fen1;ccr3;ednra;p2ry1 |
| intracellular transport; | 99#910 | 4.62E-07 | sdcbp;tom1l2;homer3;aaas;slc25a1;rrbp1;timm50;gopc;camk1;arsb;tubb3;tbrg1;rtp4;stx16;pick1;stx1b;fxc1;stx1a;tom1;stx11;kif5a;taok2;arl6ip1;rab35;nfkbie;sytl3;ipo8;sec61a1;kdelr2;ap1s3;mbtps1;vps54;atg4c;tmem48;vps41;dnhd1;dynlrb2;tram1;use1;nup160;hspd1;cog2;pex19;dclk1;stx2;myh4;htatip2;rpl23;nup153;tomm40;kif1a;nup98;als2;fyb;rffl;kif27;upf1;trappc1;ucp3;sar1a;kif3b;arcn1;gckr;katnb1;ehd1;pex13;smg5;kif26b;pou1f1;pola2;nup210;stx18;fut10;bard1;rtp3;malt1;slc25a37;kif18a;tomm34;gga1;sec24a;anp32a;tmed10;napg;sec13;rere;kif23;slc25a36;napa;cry2;slc25a6;sec61g;sec31a;ap4s1;gosr1;kif13b;osbpl5;wdr19;gsk3b |
| biopolymer modification; | 248#2815 | 4.62E-07 | pigq;sacs;nek10;ptk2;dusp12;galnt1;map3k12;alg2;mertk;fbxo10;usp30;rnf34;gna15;timm50;b3gnt3;myo3a;stk25;dusp7;psen1;furin;epha5;gna11;mylk2;gpaa1;evpl;sh3rf2;pick1;epha7;clk4;adamts13;ttn;hipk4;lnx1;ilkap;glyctk;ppm1g;map2k1;taok2;tec;map3k1;herc1;urm1;vrk1;st6gal2;asb16;arih1;grk4;ptpn18;phkg2;ublcp1;myod1;prmt2;csk;cav2;ulk3;usp49;fbxo15;loxl1;zdhhc1;ptpn1;brca1;rps6kb2;wdr5b;mapk10;manba;atg7;rabggta;ptpn4;usp35;lrsam1;irak2;psenen;cdc25c;usp46;parp4;ube2b;rbck1;tceb1;dph3;sbf2;mtmr7;bmpr1a;dyrk2;il22ra2;prpf4b;vprbp;fut3;yes1;dapk2;rnf149;ppm1f;pias4;socs6;nek3;asb1;pik3c2a;fbxo43;srpk2;gcnt3;psen2;gcnt4;lcmt1;obscn;mtmr2;gucy2c;cdkl2;rho;adck1;ppef2;fbxl3;insrr;rnf216;usp48;ephb2;prkar2b;lipc;ing4;rgr;uevld;gal3st1;fbxl7;mpdu1;ripk1;gsk3b;chrm1;shprh;alpk1;wwp1;plod3;dyrk3;traf6;pkn3;asb17;cdkl3;ptprr;prkacb;camk1;sdf2;anapc11;oasl;st6galnac1;flt3;b3gat1;sirt6;spcs3;cdc23;ubqln4;usp2;erg;stk38l;ube3b;stk33;ulk2;ubqlnl;rnf111;lcat;b3galt5;phkg1;map2k2;stambp;prkca;rps6kb1;cpa4;stk24;ubl7;taok3;gypc;fbxw11;senp3;sae1;trim32;prmt7;b3gnt2;txk;qtrtd1;pcmtd1;parp1;sf3a1;atg4c;otud7b;cask;hat1;ptprh;ptprc;limk2;cog2;padi6;csnk1g2;dclk1;ttll2;mycbp2;sirt2;usp29;usp26;pomgnt1;sec11a;chek1;mapkapk2;alg5;ephb6;pigs;ppm1m;pigb;usp7;mapk14;ppt1;cdc25b;cd37;usp10;ssh1;ube2d3;ssh2;fyb;rffl;trim23;igsf10;nid1;wwp2;fut11;gan;sgk2;klhl3;atg3;socs7;fut10;bard1;ptp4a3;ulk4;malt1;lrrk2;smurf2;mylip;atg12;nek9;mdm2;ptpn3;mast3;dhps;cry2;bub1b;ppme1;usp45;pigz;irak1;aurkb;mgat2;adprhl1;tbk1 |
| metabolic process; | 1043#14566 | 4.8E-07 | sacs;naglu;map3k12;fbxo10;usp30;znf648;myo3a;psmc2;znf566;pbld;tbrg1;baat;lrp10;pth;l3mbtl2;adamts13;znf187;zfpm2;psmf1;znf644;tec;dak;pdpr;arih1;phkg2;myod1;il27ra;glis2;pstk;hoxc10;cav2;vsx1;mmp7;dtd1;gnpat;sox14;endog;wdr5b;mapk10;mael;rfc3;edn1;myog;psenen;cpb2;fmo4;dmtf1;taf1b;pnrc2;dhodh;rpl8;pou1f1;pdzd3;pdia6;dhx35;mtmr2;pbx4;dhrs3;fbxl7;ddx1;man2b2;dyrk3;rrbp1;gck;mocs3;st6galnac1;b3gat1;fabp6;znf280d;rpl6;tbxas1;khk;arg2;kcnma1;stk38l;micall1;zbtb32;l2hgdh;gk;pglyrp1;lct;gadd45a;glt25d2;sf3a1;rps5;avil;prpf3;prdm15;dalrd3;fancb;znf629;cbfa2t2;arid4b;lepre1;mlxipl;smad5;sirt2;pomgnt1;mycl1;usp7;gbx1;znf576;duox2;jazf1;osbpl2;wwp2;amdhd2;ide;il19;arpc3;znf713;socs7;fut10;proca1;hoxb4;hmgcl;mrpl9;tsr2;notch2;trip11;utp18;znf81;fam83d;mapk8ip2;med6;clpx;lypla1;usp53;znf672;ift88;nek10;prepl;mrpl30;zfp37;lyzl1;znf212;gna15;cpa5;fkbp10;gys2;upp2;ca3;gna11;yaf2;tpp2;ttf1;map2k1;tcf25;pofut2;asb16;dhrs7c;ddc;znf683;aanat;hibadh;pycard;ulk3;znf624;loxl1;a4galt;cyp11a1;gtf2h2;nrip2;manba;atg7;lrsam1;znf250;cpsf1;usp46;traf1;sp2;dyrk2;znf202;slc34a1;ndufb2;tlr6;mthfd1;srpk2;gcnt3;gucy2c;nod1;tubg1;znf22;glb1l;ripk1;osbpl5;slc3a1;tfeb;nkx1-2;tsn;prkacb;polr2f;phf20;mcfd2;serinc2;hs3st3b1;rbm15;zkscan1;lep;nudt1;usp2;papolb;atp5g2;stk33;rnase6;taok3;ipo8;lyg2;sae1;nkx2-6;trim32;txk;qtrtd1;parp1;arsi;ptprc;isy1;hspd1;rsad1;foxd2;htatip2;alg5;paip2b;fyb;arcn1;suv39h1;rcbtb1;yeats4;tep1;prpsap1;scap;trmt6;dnajc13;mlh3;atg12;mdm2;pnpla8;ccdc59;nqo1;napg;dhps;mtfmt;chat;sycp3;mgat2;mcm6;dusp12;sardh;zfp62;mertk;rnf34;b3gnt3;sod3;ears2;stk25;psen1;arid1b;rpl28;epha7;cilp;ttn;calcoco1;rps9;vps72;afmid;rars;tlr9;rpl12;asf1a;cldn14;ublcp1;apod;prmt2;ncoa3;coq3;gfod1;tk1;ptpn4;chpt1;me3;actn2;bbs10;napsa;apc;ecd;dnaja1;smg5;lrp1;foxj3;ppm1f;txnl4b;supt3h;fbxo43;canx;arntl2;cpt1b;sox9;mrpl24;etv2;ganab;pla2g12b;usp48;map2;recql;rps3a;irf2;uevld;phb2;gtf2e1;gsk3b;nck1;soat1;lias;thoc3;cdkl3;pitpnm3;vwf;aldh5a1;znf641;pax3;clasp1;dnajc17;astl;galnt5;hivep1;ulk2;lcat;b3galt5;gucy1a2;prkca;clgn;smarcad1;pitpnb;znf667;znf175;ebi3;tmem48;dag1;sohlh2;dpf3;e2f6;plcg1;fanca;ighmbp2;atrip;ube2d3;med18;nrbf2;mrps5;isl2;mus81;qdpr;ptp4a3;malt1;idi1;mrpl49;nek9;ndufs8;tbc1d10b;adamts14;osbpl7;aurkb;pop1;adprhl1;atp6v1a;wfs1;tbk1;cpd;foxn1;qars;znf35;cxcl12;ilkap;znf23;ppp1r2;nr0b2;rab35;tert;noto;chst3;ldha;grk4;slc25a13;sptlc1;tmprss12;smarcd2;crnkl1;rps6kb2;psat1;cfi;ercc3;irak2;proc;parp4;rfx2;rbck1;pisd;bmpr1a;atp6v0b;prpf4b;znf16;vprbp;hnrnpl;atpif1;znf569;yes1;pou2f3;dapk2;rnf149;skiv2l2;hmgcll1;asb1;rpap1;eya2;gtf2h5;atp5b;rps15a;znf770;rfc5;hes5;rnf216;ephb2;zmiz2;klf8;atp5f1;znf238;msh5;pgk2;nfyc;shprh;hivep2;rpl7l1;amacr;pkn3;meox1;eif2c3;abtb1;ptprr;poll;tfdp2;pars2;prss22;elovl7;aire;eif5a;sirt6;mef2d;rbp3;erg;dnase1l2;ubqlnl;prrx2;rnf111;pou5f1;lonrf2;nanos3;sds;gypc;pfkp;esco2;krt7;cecr5;naaladl1;tigd2;efcab6;plch2;brca2;elp4;ncf1;usp29;sec11a;pigb;exosc8;pafah2;ppt1;cdc25b;nup98;pold2;trim23;crx;ddx41;slco4a1;nr1h3;gan;immp1l;atg3;psma6;polr3d;casp14;tmprss9;nab1;acads;snrpd3;mat2a;thnsl1;mmp19;dmgdh;ext2;nars;gsta1;thnsl2;gmps;pigq;exosc10;galnt1;tead4;cfb;rab18;pla2g12a;timm50;dld;arsb;gldc;gphn;furin;tph1;farsb;mst1;znf300;znf275;larp7;ptgis;smpd1;atp6v1e2;suclg1;hal;rbp2;psmb6;nr2e1;rxrg;notch1;c4a;pop5;poln;eif4ebp1;masp1;lypla2;csk;leo1;vgll3;rad54b;mef2b;hars2;zdhhc1;mettl4;ung;pou5f2;znf32;aff4;nr1d1;upf1;acsl1;pnliprp3;rarres2;socs6;setd7;edf1;rdbp;cherp;scrn3;mars;nr4a1;rho;ccna2;adck1;ace;insrr;rps14;ndufb5;tcf15;gcgr;ak3;crnn;foxp3;phf17;chrm1;traf6;plod3;mrpl43;asb17;tkt;pqbp1;irf5;sdf2;hsd17b11;flt3;snca;fkbp2;cdc23;fdft1;tcf23;phkg1;fuca2;adam7;morf4l1;suv420h1;flrt2;zscan2;mms19;pcbp2;adam21;stard5;stat3;atg4c;gad2;brms1l;limk2;padi6;nit2;npas2;bdh1;mapkapk2;hoxd8;mmp24;usp10;als2;sf3b3;pafah1b2;mrpl17;igsf10;pex13;klhl3;gtf2i;mcm10;prpf40a;pabpc1;adipoq;sox13;taf1a;dr1;gga1;znf329;adam33;ppfibp2;pla2g4b;znf555;cdsn;ssbp2;hsd11b1;mast3;bub1b;hk1;trmu;ltc4s;mmp11;sphar;rpl5;lars;sdcbp;atp1a1;corin;pogk;slc20a1;epha5;gpaa1;egln1;foxr1;sh3rf2;znf34;sult1c2;hipk4;tox3;ppm1g;znrd1;taok2;sgms2;gata3;sart3;znf470;ifnk;rab8b;hp;ddhd2;slc25a12;isoc1;znf142;fen1;adcy3;rabggta;ndufb7;vamp4;aldob;acsbg2;dph3;tbx10;il22ra2;kiaa1109;tsen2;pbrm1;dmrtb1;cdkl2;klk9;guk1;avpr1a;tasp1;ehf;ereg;cct6b;lipc;pxn;ap4s1;sptbn2;bckdhb;mpdu1;uap1;abhd5;srf;alpk1;wwp1;scube1;elavl2;notch4;glt6d1;rad21;dio1;hsf4;ftcd;rpp14;l3mbtl3;alas1;ube3b;foxd4l1;psip1;stambp;map2k2;ubl7;pfdn1;pla2g2c;ezh1;aoc3;rps23;hat1;piwil1;ptprh;use1;cercam;tbr1;zhx2;aldh3a2;ephb6;mto1;pigs;nfatc1;sphk1;ssh1;ssh2;rffl;ucp3;trim28;dhx38;iscu;polr3g;lrrk2;cdkn1a;eif4g3;smarce1;elavl1;ppig;acp6;zbtb7a;cry2;lgmn;ppme1;fgb;impa1;lsm10;adamts16;nod2;atm;trex1;ptk2;acaca;ppil3;snrpa;fmod;trim29;adamts17;fignl1;pick1;clk4;c1qc;ccbl2;glyctk;itpka;mdc1;rbms2;rce1;lhx3;pyroxd1;taf5;znf181;wdr33;mccc1;ptpn18;nom1;sept11;zbtb5;capg;tmem68;fbxo15;brca1;usp35;cdc25c;sp100;sult1b1;cecr2;nkx2-5;pfdn5;sbf2;mtmr7;gne;casp8;psmb4;dip2a;carhsp1;znf532;nek3;fdx1l;pik3c2a;gal3st3;nfyb;znf529;bcl11b;hexb;plcb3;ppef2;ca6;fbxl3;rfc1;acsf2;prkar2b;wdr7;gfpt2;ing4;nr1h4;rpl18a;cdc73;cpt1c;timp1;anapc11;msc;serpina5;spcs3;ubqln4;grhpr;thrsp;dbp;hsd17b12;rps6kb1;sertad3;stk24;cpa4;fbxw11;lzts1;senp3;rpl39;c8b;atp1a2;lctl;ap1s3;znf584;b3gnt2;nudt21;ftsj2;mbtps1;otud7b;tle4;znf710;csnk1g2;dclk1;mycbp2;ttll2;gaa;hsd17b2;zkscan5;usp26;pank4;ncoa1;ppm1m;rpp38;mapk14;hoxb13;cd37;txnrd3;pitx2;fut11;pola2;steap3;gfod2;mylip;med11;ptpn3;acaa2;gtf3a;pum2;rbm17;plcd4;znf646;pigz;usp45;irak1;msh2;mrpl11;idua;acot9;mapre1;sgpp1;alg2;gk2;znf524;lonrf3;sept7;dusp7;mylk2;klk11;fkbp5;chmp1a;evpl;znf345;grhl3;lnx1;rad52;brd8;fbxo8;smarcb1;adamts8;aarsd1;map3k1;adrb1;herc1;urm1;prss21;st6gal2;vrk1;znf688;znf395;casp8ap2;nr4a3;tmlhe;usp49;cenpj;rexo2;zbtb3;ptpn1;rpl23;gabpa;tceb1;ube2b;atxn3;uimc1;neu2;mmp25;zcchc4;katnb1;fut3;txn;pias4;eif4a1;mat2b;mif4gd;atp4a;acsl5;jdp2;gcnt4;psen2;dnajb5;cda;obscn;lcmt1;ilf3;gda;clp1;rgr;gal3st1;rbak;nat6;slu7;pcgf5;dis3;srd5a3;flad1;gopc;fabp3;pnpt1;camk1;oasl;snapc4;tubb3;hoxc8;ugcg;dctd;sftpb;crbn;gba3;exosc7;foxn2;mkks;zmat5;agpat4;barx1;rplp1;prmt7;pcmtd1;gmppa;cask;slc27a3;hmgcs1;cog2;pnpla4;terf2;mgea5;nhej1;eef1a2;ncoa6;chek1;capn10;phlda1;tlr3;nid1;hnrnpr;pou2f2;atf2;sgk2;med24;bard1;bmp6;klk14;ulk4;gzf1;smurf2;rps27;msh4;plcl2;angptl3;got1;rere;galt;tmprss11d;mrps14;etv1;atf5;npbwr1;ssbp1 |
| apoptosis; | 94#855 | 5.75E-07 | phf17;faslg;fadd;sgpp1;traf6;rnf34;rad21;psen1;pax3;ntf3;eif5a;ppp1r13b;naip;snca;cidec;pth;stambp;prkca;taok2;siva1;gadd45a;tpt1;unc5a;casp8ap2;ctnnal1;ptprc;pycard;hspd1;brca2;cideb;aven;brca1;eef1a2;gdf5;sfrp1;api5;htatip2;ercc3;phlda1;proc;ppt1;cd2;sphk1;sfn;actn2;traf1;rtn4;cecr2;rffl;trim35;ddx41;dyrk2;pdcd1;casp8;pacs2;il19;bcar1;bik;dapk2;ppm1f;casp14;ift57;bard1;tnfrsf21;tia1;malt1;cdkn1a;steap3;ctnnbl1;psen2;alb;atg12;ccar1;sox9;unc13b;nod1;notch2;akt1s1;rnf216;bcl2a1;bub1b;slc25a6;mapk8ip2;rps3a;sycp3;bcl2l13;smo;pdcd10;atf5;nod2;ing4;pik3r2;ripk1;gsk3b |
| cell communication; | 443#5560 | 8.22E-07 | ly96;ptk2;fadd;map3k12;slc8a1;pde1a;mertk;rab18;fmod;stk25;ccr4;psen1;il1rl2;furin;lrrfip2;ndc80;tph1;drg2;pick1;pth;epha7;il15ra;adamts13;ttn;itpka;calcoco1;npffr1;smpd1;oprd1;tec;plek;tlr9;nkiras2;rasal1;notch1;clec1a;rgs8;fzd6;il27ra;eif4ebp1;tbc1d2;prmt2;cav2;gpsm2;mog;ncoa3;gpr56;ccr3;arhgef1;ednra;brca1;rhov;strn4;mapk10;nptn;edn1;gfra3;sos1;psenen;cd2;ptgir;cecr2;htr3a;apc;sbf2;tnn;casp8;cd53;cplx1;htr5a;cd33;skap1;bcar1;mc3r;ptafr;gpr4;carhsp1;pdzd3;fez2;socs6;ppp2r2d;pik3c2a;stard13;vav2;arntl2;plxnb2;tnc;snx27;tbc1d23;hexb;pag1;arfip2;zdhhc17;rapgef2;anp32a;psd3;unc13b;plcb3;rho;rasa2;nr4a1;gprc5a;lhcgr;insrr;cd47;scn11a;pkd2l1;wnt8b;prkar2b;rap2a;bcar3;smo;gcgr;arl8b;pik3r2;dbn1;gnb1;nr1h4;clcn6;gsk3b;rab38;chrm1;adora2b;itga3;fgd5;nck1;ccr5;cd69;fibcd1;traf6;bcl9;asb17;rrbp1;il15;gpr22;aldh5a1;diras1;gck;sstr2;flt3;ntf3;fgf1;cacna1i;snca;tagap;gpr142;kcnma1;cytl1;stx1b;ecm1;asgr2;stk38l;mc1r;gucy1a2;gpr45;prkca;rps6kb1;mms19;itgal;rab17;cd97;stk24;fbxw11;kiss1r;dgki;rin2;anxa3;otud7b;stat3;gad2;akap7;tle4;il18r1;ect2;c1s;csnk1g2;cabp2;p2ry1;dclk1;pcdhb2;smad5;gdf5;npas2;cspg4;plcg1;mapkapk2;f2rl1;ncoa1;ralgps2;mapk14;gpr85;chl1;als2;duox2;tacr2;gpr158;igfbp2;cks2;ide;il19;wnt9a;grin2c;gtf2i;gabrr3;socs7;mcf2l;tlr5;ift57;adipoq;malt1;spred1;itgb7;cntnap2;darc;pla2g4b;ppfibp2;lifr;tbc1d17;nphp4;p2rx4;notch2;tbc1d10b;bub1b;mapk8ip2;il12rb1;plcd4;bcl2l13;irak1;nppb;pcdhb14;gpr171;ccr9;trim13;tbk1;ift88;tbc1d24;sdcbp;ric8b;rhobtb2;cd3d;ms4a2;gna15;slc20a1;ccl27;gcg;nptx2;cxcl12;lamb2;epha5;ly6e;gna11;mylk2;stx1a;cxcl1;rxfp1;cntnap4;rasd1;arhgap25;kif5a;amph;dok1;trip10;ppp1r2;map2k1;taok2;brd8;fbxo8;rab35;adrb1;gata3;nmb;wnt4;asb16;ifnk;rab8b;rapgef1;gpr20;grk4;il17c;casp8ap2;vapb;pycard;hdgf;gpr84;fen1;rabgap1;gpr141;cplx3;sh3gl2;zcchc2;ptpn1;rgl3;rassf4;rps6kb2;arhgap5;adcy3;rab7l1;ncam1;irak2;wnt3a;atxn3;traf1;gpr31;rgs1;sar1a;bmpr1a;il22ra2;gap43;stoml3;dapk2;txn;tlr6;il18rap;asb1;srpk2;sema4f;lat;psen2;sh2d2a;mapk8ip3;obscn;cda;shc2;grik5;gucy2c;cdkl2;nod1;gprc5d;ccl19;pcdhb11;avpr1a;iqgap1;ephb2;ccm2;ereg;mtnr1b;ik;pxn;gng12;rgr;gal3st1;kif13b;ripk1;srf;faslg;nmur1;wwp1;cx3cr1;homer3;pkn3;notch4;ltb4r2;angpt1;sgsm3;prkacb;camk1;rab3d;rgs22;lep;tbc1d1;ppp1r1b;f2rl3;erg;arhgef5;snx21;dkk1;stambp;mctp2;taok3;gdf10;ipo8;gpr15;akap9;barx1;nts;grb10;cd19;unc5a;rab19;dgkg;ptprc;rasgef1a;plch2;plxnc1;nedd9;stx2;gng8;ncf1;sfrp1;ncoa6;ephb6;rassf8;nfatc1;tlr3;ppt1;sphk1;gpbar1;sfn;tnfrsf11a;lhb;fyb;trim23;shank3;sgk2;gria1;pde3b;med24;bik;rasgrp3;arf4;mapk8ip1;tnfrsf21;scap;apba3;vipr2;bmp6;cdkn1a;fgf7;lrrk2;sh2b3;klrc2;smurf2;rps27;alb;gpr114;plcl2;kcnip1;angptl3;wdr67;nqo1;ext2;pdap1;arhgap28;cd164;caskin1;chat;impa1;hap1;fgb;atm;nod2;npbwr1;rassf3 |
| programmed cell death; | 94#862 | 9.15E-07 | phf17;faslg;fadd;sgpp1;traf6;rnf34;rad21;psen1;pax3;ntf3;eif5a;ppp1r13b;naip;snca;cidec;pth;stambp;prkca;taok2;siva1;gadd45a;tpt1;unc5a;casp8ap2;ctnnal1;ptprc;pycard;hspd1;brca2;cideb;aven;brca1;eef1a2;gdf5;sfrp1;api5;htatip2;ercc3;phlda1;proc;ppt1;cd2;sphk1;sfn;actn2;traf1;rtn4;cecr2;rffl;trim35;ddx41;dyrk2;pdcd1;casp8;pacs2;il19;bcar1;bik;dapk2;ppm1f;casp14;ift57;bard1;tnfrsf21;tia1;malt1;cdkn1a;steap3;ctnnbl1;psen2;alb;atg12;ccar1;sox9;unc13b;nod1;notch2;akt1s1;rnf216;bcl2a1;bub1b;slc25a6;mapk8ip2;rps3a;sycp3;bcl2l13;smo;pdcd10;atf5;nod2;ing4;pik3r2;ripk1;gsk3b |
| intracellular signaling cascade; | 182#1965 | 1.13E-06 | tbc1d24;sdcbp;fadd;map3k12;rhobtb2;rab18;gna15;slc20a1;psen1;gna11;ndc80;pick1;pth;ttn;cxcl1;rasd1;calcoco1;oprd1;taok2;fbxo8;rab35;tec;adrb1;plek;nkiras2;asb16;rapgef1;rab8b;rasal1;tbc1d2;casp8ap2;vapb;pycard;ncoa3;fen1;rabgap1;ccr3;arhgef1;ednra;brca1;rgl3;rps6kb2;arhgap5;adcy3;rhov;mapk10;edn1;sos1;rab7l1;irak2;ptgir;cecr2;sar1a;rgs1;il22ra2;casp8;gap43;mc3r;dapk2;ptafr;carhsp1;pdzd3;tlr6;socs6;asb1;pik3c2a;srpk2;lat;psen2;vav2;mapk8ip3;obscn;shc2;tbc1d23;pag1;arfip2;zdhhc17;rapgef2;anp32a;psd3;gucy2c;unc13b;plcb3;rasa2;nod1;lhcgr;avpr1a;iqgap1;prkar2b;ccm2;rap2a;bcar3;mtnr1b;gcgr;arl8b;gnb1;ripk1;gsk3b;rab38;adora2b;chrm1;faslg;fgd5;nmur1;traf6;asb17;ltb4r2;prkacb;sgsm3;rab3d;diras1;sstr2;tbc1d1;f2rl3;ecm1;stk38l;arhgef5;mc1r;gucy1a2;stambp;prkca;mctp2;rab17;taok3;dgki;rin2;otud7b;stat3;dgkg;rab19;akap7;rasgef1a;ect2;plch2;p2ry1;dclk1;ncoa6;plcg1;mapkapk2;ncoa1;ralgps2;nfatc1;tlr3;mapk14;ppt1;sphk1;sfn;als2;fyb;trim23;shank3;sgk2;cks2;med24;bik;socs7;mcf2l;rasgrp3;arf4;mapk8ip1;ift57;adipoq;scap;apba3;malt1;lrrk2;spred1;sh2b3;pla2g4b;tbc1d17;plcl2;wdr67;notch2;tbc1d10b;bub1b;mapk8ip2;plcd4;bcl2l13;irak1;nod2;trim13;tbk1 |
| catabolic process; | 103#973 | 1.18E-06 | exosc10;sardh;lyzl1;pla2g12a;usp30;psmc2;pnpt1;aldh5a1;hsd17b11;upp2;gck;gldc;arg2;cdc23;usp2;adamts13;gba3;dnase1l2;exosc7;smpd1;psmf1;rnase6;flrt2;fbxo8;pglyrp1;afmid;suclg1;hal;pfkp;lyg2;psmb6;ldha;pdpr;arih1;pla2g2c;mccc1;ddhd2;gad2;hibadh;use1;mmp7;dtd1;usp49;plch2;pnpla4;zhx2;gaa;usp29;usp26;plcg1;usp35;ercc3;usp7;ppt1;usp46;pafah2;sphk1;usp10;ube2b;tceb1;aldob;ube2d3;duox2;cecr2;pafah1b2;upf1;ecd;smg5;pnliprp3;psma6;psmb4;qdpr;proca1;fut10;mthfd1;bard1;smurf2;mdm2;pla2g4b;pnpla8;cda;hexb;mmp19;elavl1;angptl3;plcb3;dmgdh;got1;pla2g12b;adamts14;dhps;usp48;hk1;plcd4;usp45;mmp11;pop1;lipc;uevld;usp53;bckdhb;fbxl7;pgk2 |
| response to endogenous stimulus; | 64#529 | 1.8E-06 | trex1;shprh;poll;rad21;gys2;gck;nudt1;lcat;rad52;mdc1;map2k1;mms19;rif1;gata3;lct;gadd45a;esco2;wdr33;asf1a;parp1;poln;cav2;rad54b;fancb;fen1;brca2;cideb;brca1;nhej1;gtf2h2;cyp11a1;rfc3;ncoa6;ung;fanca;ercc3;chek1;ighmbp2;parp4;atrip;aldob;ube2b;sfn;atxn3;apc;upf1;dyrk2;mus81;adipoq;bard1;cdkn1a;mlh3;msh4;gtf2h5;acads;mat2a;rfc5;ccna2;recql;cry2;msh2;msh5;atm;nr1h4 |
| sensory perception of smell; | 3#540 | -1.9E-06 | adcy3;mkks;b3gnt2 |
| negative regulation of nucleobase, nucleoside, nucleotide and nucleic acid metabolic process; | 44#319 | 2.02E-06 | sirt2;wwp1;edn1;jazf1;uimc1;nkx2-5;sirt6;yaf2;znf202;chmp1a;atpif1;ttf1;znf345;pdzd3;pias4;bard1;gzf1;zbtb32;vps72;smurf2;nr0b2;dr1;tcf25;mdm2;cda;sox9;nab1;smarce1;stat3;zbtb7a;rps14;ilf3;glis2;ereg;irf2;brca2;ing4;znf238;zhx2;foxp3;nr1h4;phb2;brca1;e2f6 |
| cell morphogenesis; | 59#478 | 2.4E-06 | stx2;phf17;arhgap5;fgd5;sdcbp;nck1;prickle2;ntng2;chpt1;wnt3a;spag6;ppt1;sphk1;ube2b;ssh1;chl1;als2;actn2;rtn4;clasp1;tnn;igfbp2;gap43;lhfpl5;yeats4;bcar1;ndrg4;socs7;fez2;socs6;nrcam;emp3;kctd11;sema4f;cdkn1a;morf4l1;taok2;vav2;brd8;mkks;pdpn;sertad3;fblim1;emp1;cda;atp10a;arfip2;b3gnt2;notch2;cd320;bmp10;capg;dlg1;brms1l;nppb;dbn1;armc10;nedd9;shroom1 |
| cellular structure morphogenesis; | 59#478 | 2.4E-06 | stx2;phf17;arhgap5;fgd5;sdcbp;nck1;prickle2;ntng2;chpt1;wnt3a;spag6;ppt1;sphk1;ube2b;ssh1;chl1;als2;actn2;rtn4;clasp1;tnn;igfbp2;gap43;lhfpl5;yeats4;bcar1;ndrg4;socs7;fez2;socs6;nrcam;emp3;kctd11;sema4f;cdkn1a;morf4l1;taok2;vav2;brd8;mkks;pdpn;sertad3;fblim1;emp1;cda;atp10a;arfip2;b3gnt2;notch2;cd320;bmp10;capg;dlg1;brms1l;nppb;dbn1;armc10;nedd9;shroom1 |
| protein metabolic process; | 460#5858 | 2.52E-06 | pigq;sacs;ptk2;dusp12;galnt1;map3k12;mertk;ppil3;fbxo10;cfb;usp30;rnf34;timm50;fmod;b3gnt3;myo3a;ears2;psmc2;stk25;adamts17;psen1;furin;tbrg1;mst1;farsb;rpl28;pick1;epha7;clk4;c1qc;adamts13;ttn;glyctk;rps9;psmf1;rce1;tec;rars;tlr9;psmb6;rpl12;arih1;ptpn18;c4a;cldn14;phkg2;ublcp1;myod1;eif4ebp1;masp1;pstk;sept11;prmt2;csk;cav2;capg;mmp7;fbxo15;hars2;zdhhc1;brca1;wdr5b;mapk10;ptpn4;usp35;psenen;cpb2;cdc25c;actn2;napsa;bbs10;apc;upf1;sbf2;pfdn5;mtmr7;dnaja1;casp8;rpl8;psmb4;pdia6;ppm1f;socs6;nek3;fbxo43;gal3st3;canx;scrn3;hexb;mtmr2;mars;mrpl24;rho;adck1;ppef2;ace;fbxl3;insrr;rps14;usp48;map2;prkar2b;rps3a;wdr7;ing4;uevld;fbxl7;foxp3;rpl18a;gsk3b;chrm1;nck1;ddx1;plod3;dyrk3;traf6;mrpl43;rrbp1;asb17;cdkl3;vwf;timp1;sdf2;aldh5a1;anapc11;st6galnac1;flt3;clasp1;b3gat1;serpina5;dnajc17;spcs3;rpl6;fkbp2;astl;cdc23;ubqln4;stk38l;ulk2;lcat;b3galt5;phkg1;adam7;prkca;rps6kb1;l2hgdh;clgn;cpa4;stk24;smarcad1;fbxw11;senp3;rpl39;c8b;ap1s3;b3gnt2;adam21;sf3a1;ebi3;mbtps1;atg4c;otud7b;rps5;tmem48;avil;dag1;dalrd3;limk2;padi6;csnk1g2;dclk1;mycbp2;ttll2;lepre1;sirt2;usp26;pomgnt1;mapkapk2;fanca;ppm1m;usp7;mapk14;cd37;usp10;mmp24;ube2d3;als2;sf3b3;mrpl17;igsf10;wwp2;fut11;mrps5;pex13;klhl3;ide;il19;arpc3;socs7;fut10;adipoq;ptp4a3;malt1;mylip;mrpl49;gga1;adam33;nek9;ndufs8;ptpn3;mrpl9;mast3;adamts14;pum2;bub1b;fam83d;mapk8ip2;usp45;pigz;irak1;aurkb;mmp11;clpx;adprhl1;mrpl11;usp53;ift88;tbk1;nek10;rpl5;mapre1;lars;cpd;prepl;alg2;mrpl30;corin;gna15;cpa5;fkbp10;lonrf3;qars;cxcl12;dusp7;sept7;epha5;gna11;mylk2;klk11;gpaa1;egln1;tpp2;evpl;sh3rf2;fkbp5;ttf1;lnx1;hipk4;ilkap;ppm1g;map2k1;taok2;fbxo8;aarsd1;adamts8;map3k1;herc1;gata3;urm1;chst3;prss21;vrk1;st6gal2;asb16;grk4;hp;pycard;ulk3;cenpj;usp49;tmprss12;loxl1;ptpn1;rps6kb2;cfi;nrip2;manba;atg7;rabggta;lrsam1;rpl23;irak2;vamp4;proc;usp46;parp4;ube2b;tceb1;rbck1;traf1;dph3;mmp25;bmpr1a;katnb1;dyrk2;il22ra2;kiaa1109;prpf4b;vprbp;fut3;atpif1;yes1;dapk2;rnf149;eif4a1;pias4;tlr6;asb1;srpk2;gcnt3;psen2;gcnt4;dnajb5;lcmt1;cda;obscn;rps15a;gucy2c;cdkl2;nod1;klk9;rnf216;ephb2;tasp1;ereg;tubg1;cct6b;lipc;pxn;ap4s1;rgr;gal3st1;sptbn2;mpdu1;ripk1;abhd5;shprh;nfyc;alpk1;wwp1;rpl7l1;pkn3;scube1;abtb1;eif2c3;gopc;ptprr;prkacb;camk1;pars2;oasl;prss22;tubb3;hs3st3b1;eif5a;sirt6;crbn;usp2;erg;ube3b;rbp3;stk33;ubqlnl;rnf111;map2k2;stambp;lonrf2;nanos3;mkks;ubl7;taok3;gypc;pfdn1;ipo8;sae1;trim32;rplp1;prmt7;txk;pcmtd1;parp1;cask;rps23;hat1;krt7;ptprh;ptprc;naaladl1;use1;hspd1;cog2;mgea5;eef1a2;usp29;sec11a;chek1;alg5;ephb6;capn10;pigs;pigb;phlda1;tlr3;ppt1;cdc25b;nup98;ssh1;paip2b;ssh2;fyb;rffl;trim23;nid1;arcn1;gan;sgk2;immp1l;atg3;psma6;casp14;bard1;scap;trmt6;klk14;dnajc13;ulk4;lrrk2;tmprss9;smurf2;rps27;atg12;eif4g3;mdm2;smarce1;mmp19;angptl3;napg;ppig;dhps;mtfmt;cry2;tmprss11d;ppme1;mrps14;lgmn;nars;fgb;mgat2;nod2;adamts16 |
| negative regulation of progression through cell cycle; | 34#225 | 3.3E-06 | nat6;cdc73;htatip2;sgsm3;apc;reck;tbrg1;gas2l3;ppp1r13b;chmp1a;ilkap;bard1;ppm1g;kctd11;cdkn1a;stard13;btg4;smarcb1;lzts1;gadd45a;ext2;notch2;ephb2;bub1b;pycard;dlg1;msh2;ing4;atm;xrn1;trim13;dst;rassf4;brca1 |
| negative regulation of cell proliferation; | 34#225 | 3.3E-06 | lepre1;chek1;gas8;fabp3;trim35;apc;sstr2;tbrg1;fabp6;cd33;atpif1;pou1f1;cxcl1;emp3;cdkn1a;btg4;smarcb1;adamts8;mdm2;fth1;kiss1r;ifnk;notch2;cd164;csk;ereg;cav2;pds5b;tob2;brca2;ing4;krt4;dlg3;foxp3 |
| protein kinase cascade; | 49#376 | 3.69E-06 | rps6kb2;adora2b;faslg;fadd;mapk10;map3k12;edn1;traf6;mapkapk2;irak2;slc20a1;tlr3;mapk14;fyb;shank3;il22ra2;sgk2;casp8;dapk2;ecm1;stk38l;rasgrp3;tlr6;mapk8ip1;socs6;adipoq;srpk2;malt1;stambp;spred1;prkca;taok2;tec;taok3;mapk8ip3;nkiras2;rapgef2;zdhhc17;nod1;stat3;otud7b;ccm2;mapk8ip2;irak1;ect2;nod2;trim13;ripk1;tbk1 |
| cellular lipid metabolic process; | 84#768 | 4E-06 | pigq;acot9;sgpp1;soat1;cpt1c;acaca;pla2g12a;pitpnm3;fabp3;aldh5a1;hsd17b11;elovl7;serinc2;fabp6;lep;baat;ugcg;snca;gpaa1;sftpb;tbxas1;fdft1;lcat;hsd17b12;smpd1;ptgis;flrt2;nr0b2;sgms2;agpat4;glt25d2;stard5;mbtps1;sptlc1;lypla2;hmgcs1;slc27a3;cercam;brca1;gnpat;a4galt;hsd17b2;cyp11a1;pigs;chpt1;pigb;ppt1;sphk1;sult1b1;osbpl2;sbf2;mtmr7;pisd;gne;pex13;acsl1;rarres2;proca1;adipoq;scap;bmp6;acsl5;pik3c2a;idi1;pnpla8;cpt1b;pla2g4b;hexb;acads;mtmr2;acaa2;plcb3;hsd11b1;pla2g12b;dhrs3;osbpl7;pigz;impa1;lipc;ltc4s;lypla1;gal3st1;nr1h4;osbpl5 |
| glycoprotein metabolic process; | 32#208 | 4.29E-06 | gcnt3;galnt1;psen2;manba;gcnt4;pomgnt1;alg2;alg5;gypc;psenen;pigb;phlda1;b3gnt3;st6gal2;cd37;sdf2;b3gnt2;psen1;st6galnac1;b3gat1;tasp1;fut11;fut3;mgat2;lipc;adamts13;gal3st1;cog2;fut10;mpdu1;b3galt5;mgea5 |
| protein oligomerization; | 17#62 | 5.53E-06 | malt1;smarcad1;cda;scube1;gopc;vwf;aldh5a1;actn2;nod1;sept7;sbf2;sept11;cav2;irak1;atpif1;nod2;adipoq |
| post-translational protein modification; | 199#2235 | 5.66E-06 | nek10;ptk2;dusp12;map3k12;mertk;fbxo10;usp30;rnf34;gna15;timm50;myo3a;stk25;dusp7;psen1;furin;epha5;gna11;mylk2;gpaa1;evpl;sh3rf2;pick1;epha7;clk4;adamts13;ttn;hipk4;lnx1;ilkap;glyctk;ppm1g;map2k1;taok2;tec;map3k1;herc1;urm1;vrk1;asb16;arih1;grk4;ptpn18;phkg2;myod1;prmt2;csk;cav2;ulk3;usp49;fbxo15;ptpn1;brca1;rps6kb2;wdr5b;mapk10;atg7;ptpn4;usp35;lrsam1;irak2;psenen;cdc25c;usp46;parp4;ube2b;rbck1;tceb1;mtmr7;bmpr1a;dyrk2;il22ra2;prpf4b;vprbp;yes1;dapk2;rnf149;ppm1f;pias4;socs6;nek3;asb1;fbxo43;srpk2;psen2;lcmt1;obscn;mtmr2;gucy2c;cdkl2;rho;adck1;ppef2;fbxl3;insrr;rnf216;usp48;ephb2;prkar2b;rgr;uevld;fbxl7;ripk1;gsk3b;shprh;alpk1;wwp1;dyrk3;traf6;pkn3;asb17;cdkl3;prkacb;ptprr;camk1;anapc11;flt3;sirt6;spcs3;cdc23;usp2;erg;stk38l;ube3b;stk33;ulk2;rnf111;lcat;phkg1;stambp;map2k2;prkca;rps6kb1;stk24;cpa4;ubl7;taok3;fbxw11;senp3;sae1;trim32;prmt7;txk;parp1;otud7b;cask;atg4c;ptprh;ptprc;limk2;csnk1g2;dclk1;mycbp2;sirt2;usp29;usp26;sec11a;chek1;mapkapk2;ephb6;pigs;ppm1m;usp7;mapk14;cdc25b;usp10;ssh1;ube2d3;ssh2;fyb;rffl;trim23;nid1;igsf10;wwp2;gan;sgk2;klhl3;atg3;socs7;ptp4a3;bard1;ulk4;malt1;lrrk2;smurf2;mylip;atg12;nek9;mdm2;ptpn3;mast3;cry2;bub1b;ppme1;usp45;irak1;aurkb;adprhl1;tbk1 |
| sensory perception of chemical stimulus; | 6#584 | -6.3E-06 | adcy3;scnn1a;rtp4;mkks;b3gnt2;rtp3 |
| secretion; | 49#384 | 8.22E-06 | adora2b;nmur1;aqp1;edn1;slc22a4;gopc;scnn1a;rab3d;dph3;gck;sar1a;trappc1;tacr2;clcn5;snca;cplx1;pde3b;stx16;pick1;aqp3;kcnma1;slc34a1;stx1a;ppy;stx18;stxbp1;grhpr;lat;canx;sec61a1;sec24a;unc13b;tmed10;napg;slc26a3;napa;pycard;sec31a;rims4;snap29;nppb;nod2;gosr1;cplx3;cog2;tpd52;foxp3;osbpl5;ift88 |
| cellular macromolecule metabolic process; | 442#5656 | 8.32E-06 | pigq;sacs;ptk2;dusp12;galnt1;map3k12;mertk;ppil3;fbxo10;cfb;usp30;rnf34;timm50;fmod;b3gnt3;myo3a;ears2;psmc2;stk25;adamts17;psen1;furin;tbrg1;mst1;farsb;rpl28;pick1;epha7;clk4;c1qc;adamts13;ttn;glyctk;rps9;psmf1;rce1;tec;rars;tlr9;psmb6;rpl12;arih1;ptpn18;c4a;phkg2;ublcp1;myod1;eif4ebp1;masp1;pstk;prmt2;csk;cav2;capg;mmp7;fbxo15;hars2;zdhhc1;brca1;wdr5b;mapk10;ptpn4;usp35;psenen;cpb2;cdc25c;napsa;bbs10;apc;upf1;pfdn5;mtmr7;gne;dnaja1;casp8;rpl8;psmb4;pdia6;ppm1f;socs6;nek3;fbxo43;gal3st3;canx;scrn3;hexb;mtmr2;mrpl24;mars;rho;adck1;ppef2;ace;fbxl3;insrr;rps14;usp48;map2;prkar2b;rps3a;wdr7;ing4;uevld;fbxl7;foxp3;rpl18a;gsk3b;chrm1;nck1;ddx1;plod3;dyrk3;traf6;mrpl43;rrbp1;asb17;cdkl3;timp1;sdf2;anapc11;gck;st6galnac1;flt3;clasp1;b3gat1;serpina5;dnajc17;spcs3;rpl6;fkbp2;astl;cdc23;ubqln4;stk38l;ulk2;lcat;b3galt5;phkg1;adam7;prkca;rps6kb1;l2hgdh;clgn;cpa4;stk24;fbxw11;senp3;rpl39;c8b;glt25d2;b3gnt2;adam21;sf3a1;ebi3;mbtps1;atg4c;otud7b;rps5;tmem48;avil;dalrd3;limk2;padi6;csnk1g2;dclk1;ttll2;mycbp2;gaa;sirt2;usp26;pomgnt1;mapkapk2;ppm1m;usp7;mapk14;cd37;usp10;mmp24;ube2d3;als2;mrpl17;igsf10;wwp2;fut11;mrps5;pex13;klhl3;ide;il19;arpc3;socs7;fut10;ptp4a3;malt1;mylip;mrpl49;adam33;nek9;ndufs8;ptpn3;mrpl9;mast3;adamts14;pum2;bub1b;fam83d;mapk8ip2;usp45;pigz;irak1;aurkb;mmp11;clpx;adprhl1;mrpl11;usp53;tbk1;ift88;nek10;rpl5;mapre1;lars;cpd;prepl;mrpl30;alg2;corin;gna15;cpa5;fkbp10;lonrf3;qars;gys2;cxcl12;dusp7;epha5;gna11;mylk2;klk11;gpaa1;tpp2;evpl;sh3rf2;fkbp5;ttf1;lnx1;hipk4;ilkap;ppm1g;ppp1r2;map2k1;taok2;fbxo8;aarsd1;adamts8;map3k1;herc1;gata3;urm1;chst3;prss21;vrk1;st6gal2;asb16;grk4;hp;pycard;ulk3;cenpj;usp49;tmprss12;loxl1;ptpn1;rps6kb2;cfi;nrip2;manba;atg7;rabggta;lrsam1;rpl23;irak2;proc;usp46;parp4;ube2b;tceb1;rbck1;dph3;mmp25;bmpr1a;katnb1;dyrk2;il22ra2;kiaa1109;prpf4b;vprbp;fut3;yes1;dapk2;rnf149;eif4a1;pias4;tlr6;mat2b;asb1;srpk2;gcnt3;psen2;gcnt4;dnajb5;lcmt1;obscn;rps15a;gucy2c;cdkl2;nod1;klk9;rnf216;ephb2;tasp1;ereg;tubg1;cct6b;lipc;pxn;rgr;gal3st1;sptbn2;mpdu1;ripk1;abhd5;shprh;nfyc;alpk1;wwp1;rpl7l1;pkn3;abtb1;eif2c3;ptprr;prkacb;camk1;pars2;oasl;prss22;tubb3;hs3st3b1;eif5a;sirt6;crbn;usp2;erg;ube3b;rbp3;stk33;ubqlnl;rnf111;map2k2;stambp;lonrf2;nanos3;mkks;ubl7;taok3;gypc;pfdn1;ipo8;sae1;trim32;rplp1;prmt7;txk;pcmtd1;parp1;cask;rps23;hat1;krt7;ptprh;ptprc;naaladl1;cercam;hspd1;cog2;mgea5;eef1a2;usp29;sec11a;chek1;alg5;ephb6;capn10;pigs;pigb;phlda1;tlr3;ppt1;cdc25b;nup98;ssh1;paip2b;ssh2;fyb;rffl;trim23;nid1;gan;sgk2;immp1l;atg3;psma6;casp14;bard1;scap;trmt6;klk14;dnajc13;ulk4;lrrk2;tmprss9;smurf2;rps27;atg12;eif4g3;mdm2;smarce1;mmp19;angptl3;napg;ppig;dhps;mtfmt;cry2;tmprss11d;ppme1;mrps14;lgmn;nars;fgb;mgat2;adamts16 |
| meiotic cell cycle; | 18#71 | 8.37E-06 | fbxo43;wbp2nl;rad52;adcy3;mlh3;chek1;msh4;piwil2;rad21;dmwd;ereg;tubg1;sycp3;cks2;brca2;atm;msh5;ccna1 |
| intracellular protein transport; | 62#529 | 1.05E-05 | stx2;sdcbp;tom1l2;homer3;rpl23;nup153;tomm40;rrbp1;timm50;nup98;fyb;rffl;sar1a;arcn1;gckr;katnb1;rtp4;pex13;stx16;pick1;stx1b;fxc1;stx1a;pola2;nup210;stx18;tom1;fut10;bard1;stx11;rtp3;taok2;arl6ip1;gga1;tomm34;nfkbie;sytl3;ipo8;sec61a1;kdelr2;sec24a;ap1s3;napg;sec13;mbtps1;rere;atg4c;tmem48;napa;cry2;vps41;tram1;sec61g;hspd1;nup160;ap4s1;gosr1;pex19;cog2;kif13b;wdr19;gsk3b |
| antigen processing and presentation; | 1#415 | -1.6E-05 | cd1b |
| protein complex assembly; | 44#340 | 2.02E-05 | ptk2;nck1;fanca;scube1;vamp4;fmod;gopc;vwf;nup98;aldh5a1;actn2;traf1;sf3b3;sept7;apc;sbf2;arcn1;pex13;gpaa1;pick1;atpif1;adipoq;malt1;smarcad1;gga1;mdm2;ndufs8;cda;ipo8;ap1s3;napg;nod1;cldn14;tmem48;sept11;mapk8ip2;cav2;capg;dag1;cct6b;irak1;pxn;ap4s1;nod2 |
| vesicle-mediated transport; | 68#606 | 2.04E-05 | bet3l;rab18;gopc;rab3d;mcfd2;snca;stx16;lrp10;pick1;chmp1a;stx1b;asgr2;stx1a;epn2;tom1;stx11;amph;trip10;asgr1;rab35;abca7;kdelr2;ap1s3;rin2;vps54;vps41;use1;rims4;snap29;slc9a3;cog2;cplx3;sh3gl2;dclk1;stx2;lrp3;vamp4;ppt1;actr1a;als2;cecr2;trappc1;sar1a;arcn1;clcn5;ehd1;cplx1;lrp1;stx18;stxbp1;mapk8ip1;arf4;lat;gga1;mapk8ip3;sec24a;tmed10;unc13b;napg;napa;sh3bp4;lrp1b;sec31a;ap4s1;sptbn2;gosr1;osbpl5;wdr19 |
| defense response; | 66#584 | 2.18E-05 | adora2b;ly96;cd69;cx3cr1;cfb;s100a8;scube1;foxn1;ccr4;cxcl12;il1rl2;ly6e;bpi;c1qc;cxcl1;rnase6;prkca;itgal;pglyrp1;cd97;ppbp;gata3;c8b;tlr9;mefv;ifnk;cd19;aoc3;clec1a;stat3;c4a;il27ra;hp;masp1;il17c;ptprc;il18r1;ccr3;ncf1;cfi;irak2;tlr3;parp4;atrn;als2;mmp25;ptafr;tlr5;tlr6;defb124;il18rap;socs6;bmp6;malt1;darc;klrc2;pla2g4b;nod1;cst11;ccl19;crisp3;defb116;ereg;nod2;ccr9;tbk1 |
| growth; | 40#300 | 2.36E-05 | phf17;gdf5;chpt1;tkt;ppt1;sphk1;hoxb13;tnn;mreg;igfbp2;gap43;yeats4;bcar1;ndrg4;socs7;dsg4;socs6;bmp6;emp3;kctd11;cdkn1a;morf4l1;taok2;brd8;sertad3;adrb1;emp1;cda;st6gal2;notch2;cd320;ereg;bmp10;smo;brms1l;brca2;nppb;ing4;armc10;nedd9 |
| M phase of meiotic cell cycle; | 17#69 | 2.54E-05 | fbxo43;wbp2nl;rad52;adcy3;mlh3;chek1;msh4;piwil2;rad21;dmwd;ereg;sycp3;cks2;brca2;msh5;atm;ccna1 |
| meiosis; | 17#69 | 2.54E-05 | fbxo43;wbp2nl;rad52;adcy3;mlh3;chek1;msh4;piwil2;rad21;dmwd;ereg;sycp3;cks2;brca2;msh5;atm;ccna1 |
| cellular protein metabolic process; | 433#5584 | 2.65E-05 | pigq;sacs;ptk2;dusp12;galnt1;map3k12;mertk;ppil3;fbxo10;cfb;usp30;rnf34;timm50;fmod;b3gnt3;myo3a;ears2;psmc2;stk25;adamts17;psen1;furin;tbrg1;mst1;farsb;rpl28;pick1;epha7;clk4;c1qc;adamts13;ttn;glyctk;rps9;psmf1;rce1;tec;rars;tlr9;psmb6;rpl12;arih1;ptpn18;c4a;phkg2;ublcp1;myod1;eif4ebp1;masp1;pstk;prmt2;csk;cav2;capg;mmp7;fbxo15;hars2;zdhhc1;brca1;wdr5b;mapk10;ptpn4;usp35;psenen;cpb2;cdc25c;napsa;bbs10;apc;upf1;pfdn5;mtmr7;dnaja1;casp8;rpl8;psmb4;pdia6;ppm1f;socs6;nek3;fbxo43;gal3st3;canx;scrn3;hexb;mtmr2;mrpl24;mars;rho;adck1;ppef2;ace;fbxl3;insrr;rps14;usp48;map2;prkar2b;rps3a;wdr7;ing4;uevld;fbxl7;foxp3;rpl18a;gsk3b;chrm1;nck1;ddx1;plod3;dyrk3;traf6;mrpl43;rrbp1;asb17;cdkl3;timp1;sdf2;anapc11;st6galnac1;flt3;clasp1;b3gat1;serpina5;dnajc17;spcs3;rpl6;fkbp2;astl;cdc23;ubqln4;stk38l;ulk2;lcat;b3galt5;phkg1;adam7;prkca;rps6kb1;l2hgdh;clgn;cpa4;stk24;fbxw11;senp3;rpl39;c8b;b3gnt2;adam21;sf3a1;ebi3;mbtps1;atg4c;otud7b;rps5;tmem48;avil;dalrd3;limk2;padi6;csnk1g2;dclk1;ttll2;mycbp2;sirt2;usp26;pomgnt1;mapkapk2;ppm1m;usp7;mapk14;cd37;usp10;mmp24;ube2d3;als2;mrpl17;igsf10;wwp2;fut11;mrps5;pex13;klhl3;ide;il19;arpc3;socs7;fut10;ptp4a3;malt1;mylip;mrpl49;adam33;nek9;ndufs8;ptpn3;mrpl9;mast3;adamts14;pum2;bub1b;fam83d;mapk8ip2;usp45;pigz;irak1;aurkb;mmp11;clpx;adprhl1;mrpl11;usp53;tbk1;ift88;nek10;rpl5;mapre1;lars;cpd;prepl;mrpl30;alg2;corin;gna15;cpa5;fkbp10;lonrf3;qars;dusp7;epha5;gna11;mylk2;klk11;gpaa1;tpp2;evpl;sh3rf2;fkbp5;ttf1;lnx1;hipk4;ilkap;ppm1g;map2k1;taok2;fbxo8;aarsd1;adamts8;map3k1;herc1;gata3;urm1;chst3;prss21;vrk1;st6gal2;asb16;grk4;hp;pycard;ulk3;cenpj;usp49;tmprss12;loxl1;ptpn1;rps6kb2;cfi;nrip2;manba;atg7;rabggta;lrsam1;rpl23;irak2;proc;usp46;parp4;ube2b;tceb1;rbck1;dph3;mmp25;bmpr1a;katnb1;dyrk2;il22ra2;kiaa1109;prpf4b;vprbp;fut3;yes1;dapk2;rnf149;eif4a1;pias4;tlr6;asb1;srpk2;gcnt3;psen2;gcnt4;dnajb5;lcmt1;obscn;rps15a;gucy2c;cdkl2;nod1;klk9;rnf216;ephb2;tasp1;ereg;tubg1;cct6b;lipc;pxn;rgr;gal3st1;sptbn2;mpdu1;ripk1;abhd5;shprh;nfyc;alpk1;wwp1;rpl7l1;pkn3;abtb1;eif2c3;ptprr;prkacb;camk1;pars2;oasl;prss22;tubb3;hs3st3b1;eif5a;sirt6;crbn;usp2;erg;ube3b;rbp3;stk33;ubqlnl;rnf111;map2k2;stambp;lonrf2;nanos3;mkks;ubl7;taok3;gypc;pfdn1;ipo8;sae1;trim32;rplp1;prmt7;txk;pcmtd1;parp1;cask;rps23;hat1;krt7;ptprh;ptprc;naaladl1;hspd1;cog2;mgea5;eef1a2;usp29;sec11a;chek1;alg5;ephb6;capn10;pigs;pigb;phlda1;tlr3;ppt1;cdc25b;nup98;ssh1;paip2b;ssh2;fyb;rffl;trim23;nid1;gan;sgk2;immp1l;atg3;psma6;casp14;bard1;scap;trmt6;klk14;dnajc13;ulk4;lrrk2;tmprss9;smurf2;rps27;atg12;eif4g3;mdm2;smarce1;mmp19;angptl3;napg;ppig;dhps;mtfmt;cry2;tmprss11d;ppme1;mrps14;lgmn;nars;fgb;mgat2;adamts16 |
| lipid catabolic process; | 24#146 | 2.74E-05 | flrt2;plcg1;pla2g4b;pla2g12a;pnpla8;hexb;pafah2;ppt1;sphk1;angptl3;plcb3;aldh5a1;hsd17b11;pafah1b2;pla2g12b;pla2g2c;ddhd2;plcd4;pnliprp3;plch2;lipc;proca1;pnpla4;smpd1 |
| carbohydrate biosynthetic process; | 23#138 | 3.24E-05 | uap1;gal3st3;sds;chst3;aldob;glt25d2;gys2;ext2;gck;hs3st3b1;phkg2;dyrk2;gne;galnt5;mgat2;lipc;cercam;gfpt2;cog2;mpdu1;adipoq;mat2b;phkg1 |
| response to wounding; | 51#423 | 3.93E-05 | ly96;cfi;cx3cr1;f2rl1;cfb;irak2;s100a8;scube1;tlr3;proc;vwf;atrn;parp4;mmrn1;hoxb13;thbd;ccr4;cxcl12;mmp25;mst1;gap43;tbxas1;c1qc;ptafr;adamts13;f2rl3;tlr5;cxcl1;fut10;tlr6;il18rap;bmp6;fgf7;prkca;itgal;cd97;pla2g4b;c8b;tlr9;mefv;nod1;ccl19;aoc3;stat3;c4a;masp1;il17c;ereg;fgb;ccr3;p2ry1 |
| regulation of apoptosis; | 64#570 | 3.99E-05 | faslg;fadd;traf6;psen1;ntf3;ppp1r13b;naip;snca;cidec;pth;stambp;prkca;siva1;tpt1;casp8ap2;ptprc;pycard;hspd1;brca2;cideb;aven;brca1;gdf5;eef1a2;sfrp1;api5;htatip2;ercc3;proc;ppt1;cd2;sphk1;sfn;traf1;rtn4;actn2;trim35;dyrk2;casp8;il19;bcar1;bik;dapk2;ift57;bard1;tia1;cdkn1a;malt1;alb;sox9;unc13b;nod1;notch2;akt1s1;bcl2a1;mapk8ip2;rps3a;sycp3;bcl2l13;smo;nod2;atf5;pik3r2;gsk3b |
| positive regulation of metabolic process; | 48#391 | 4.18E-05 | srf;smad5;nck1;ncoa6;npas2;ncoa1;ercc3;myog;notch4;tlr3;trim28;gck;nkx2-5;crx;dyrk2;arid1b;yaf2;yeats4;pou1f1;ttf1;tlr6;bard1;adipoq;scap;bmp6;prkca;mms19;smarcad1;sertad3;adrb1;gata3;sox9;tlr9;angptl3;ccna2;asf1a;ebi3;ilf3;glis2;ehf;ereg;zmiz2;irak1;ncoa3;med6;foxp3;brca1;ift88 |
| RNA metabolic process; | 332#4155 | 4.47E-05 | exosc10;mcm6;zfp62;tead4;ppil3;snrpa;rab18;znf648;ears2;trim29;znf566;arid1b;farsb;znf300;l3mbtl2;znf275;larp7;znf187;zfpm2;znf644;vps72;rbms2;lhx3;rars;taf5;znf181;asf1a;nr2e1;notch1;rxrg;nom1;pop5;myod1;glis2;hoxc10;leo1;zbtb5;rad54b;vsx1;mef2b;ncoa3;hars2;brca1;sox14;mael;pou5f2;myog;znf32;aff4;sp100;dmtf1;taf1b;nr1d1;pnrc2;nkx2-5;upf1;pfdn5;ecd;smg5;pou1f1;znf532;carhsp1;foxj3;txnl4b;supt3h;rdbp;dhx35;edf1;nfyb;znf529;cherp;arntl2;bcl11b;sox9;pbx4;mars;nr4a1;etv2;rfc1;rps14;tcf15;irf2;ing4;foxp3;phb2;nr1h4;gtf2e1;phf17;ddx1;thoc3;pqbp1;irf5;znf641;pax3;msc;znf280d;rpl6;hivep1;thrsp;dbp;morf4l1;zscan2;zbtb32;suv420h1;mms19;sertad3;lzts1;znf667;pcbp2;znf584;nudt21;znf175;ftsj2;sf3a1;stat3;prpf3;prdm15;dalrd3;tle4;znf629;brms1l;znf710;cbfa2t2;dpf3;arid4b;e2f6;mycbp2;mlxipl;smad5;sirt2;zkscan5;npas2;mycl1;ncoa1;ighmbp2;rpp38;gbx1;hoxd8;hoxb13;znf576;jazf1;med18;sf3b3;nrbf2;pitx2;isl2;znf713;gtf2i;pabpc1;prpf40a;sox13;taf1a;hoxb4;dr1;znf329;med11;znf555;tsr2;notch2;gtf3a;trip11;utp18;znf81;fam83d;rbm17;znf646;trmu;med6;pop1;znf672;lars;zfp37;pogk;znf212;foxn1;znf524;qars;znf35;yaf2;foxr1;chmp1a;ttf1;znf345;znf34;grhl3;tox3;znf23;smarcb1;brd8;nr0b2;aarsd1;rab35;tcf25;gata3;noto;sart3;znf470;rab8b;znf683;znf688;znf395;nr4a3;casp8ap2;znf142;znf624;zbtb3;smarcd2;crnkl1;gtf2h2;ercc3;znf250;gabpa;cpsf1;tceb1;rfx2;atxn3;uimc1;tbx10;sp2;tsen2;znf202;prpf4b;znf16;pbrm1;hnrnpl;znf569;pou2f3;pias4;skiv2l2;mif4gd;srpk2;jdp2;eya2;gtf2h5;dmrtb1;znf770;hes5;ilf3;ehf;ereg;znf22;zmiz2;klf8;clp1;znf238;rbak;srf;slu7;nfyc;tfeb;pcgf5;dis3;hivep2;meox1;nkx1-2;notch4;elavl2;tfdp2;pnpt1;polr2f;phf20;pars2;snapc4;aire;zkscan1;hsf4;rpp14;sirt6;hoxc8;mef2d;papolb;erg;psip1;foxd4l1;foxn2;prrx2;exosc7;pou5f1;rnase6;zmat5;nkx2-6;barx1;parp1;qtrtd1;ezh1;isy1;efcab6;brca2;foxd2;zhx2;tbr1;elp4;ncoa6;htatip2;mto1;exosc8;nfatc1;trim28;crx;dhx38;ddx41;nr1h3;pou2f2;suv39h1;hnrnpr;rcbtb1;atf2;polr3g;med24;yeats4;polr3d;bard1;scap;trmt6;bmp6;gzf1;dnajc13;mdm2;eif4g3;smarce1;nab1;ccdc59;snrpd3;elavl1;ppig;rere;zbtb7a;cry2;nars;atf5;lsm10;etv1 |
| programmed cell death#regulation of programmed cell death; | 64#576 | 6.06E-05 | faslg;fadd;traf6;psen1;ntf3;ppp1r13b;naip;snca;cidec;pth;stambp;prkca;siva1;tpt1;casp8ap2;ptprc;pycard;hspd1;brca2;cideb;aven;brca1;gdf5;eef1a2;sfrp1;api5;htatip2;ercc3;proc;ppt1;cd2;sphk1;sfn;traf1;rtn4;actn2;trim35;dyrk2;casp8;il19;bcar1;bik;dapk2;ift57;bard1;tia1;cdkn1a;malt1;alb;sox9;unc13b;nod1;notch2;akt1s1;bcl2a1;mapk8ip2;rps3a;sycp3;bcl2l13;smo;nod2;atf5;pik3r2;gsk3b |
| protein targeting; | 31#218 | 6.68E-05 | sdcbp;taok2;arl6ip1;homer3;nfkbie;tomm34;rpl23;tomm40;ipo8;nup98;fyb;mbtps1;rere;atg4c;gckr;katnb1;rtp4;cry2;pex13;tram1;sec61g;pick1;hspd1;fxc1;pola2;pex19;fut10;bard1;kif13b;gsk3b;rtp3 |
| signal transduction; | 398#5142 | 9.81E-05 | ly96;ptk2;fadd;map3k12;pde1a;mertk;rab18;fmod;stk25;ccr4;psen1;il1rl2;lrrfip2;ndc80;drg2;pick1;pth;epha7;il15ra;adamts13;ttn;itpka;calcoco1;npffr1;smpd1;oprd1;tec;plek;tlr9;nkiras2;rasal1;notch1;clec1a;rgs8;fzd6;il27ra;eif4ebp1;tbc1d2;prmt2;gpsm2;ncoa3;gpr56;ccr3;arhgef1;ednra;brca1;rhov;strn4;mapk10;edn1;gfra3;sos1;psenen;cd2;ptgir;cecr2;apc;tnn;casp8;cd53;htr5a;cd33;skap1;bcar1;mc3r;ptafr;gpr4;carhsp1;pdzd3;fez2;socs6;ppp2r2d;pik3c2a;stard13;vav2;arntl2;plxnb2;tnc;snx27;tbc1d23;pag1;arfip2;zdhhc17;rapgef2;anp32a;psd3;unc13b;plcb3;rho;rasa2;nr4a1;gprc5a;lhcgr;insrr;cd47;pkd2l1;wnt8b;prkar2b;rap2a;bcar3;smo;gcgr;arl8b;pik3r2;gnb1;nr1h4;clcn6;gsk3b;rab38;chrm1;adora2b;itga3;fgd5;nck1;ccr5;cd69;fibcd1;traf6;bcl9;asb17;rrbp1;il15;gpr22;diras1;sstr2;flt3;ntf3;fgf1;cacna1i;tagap;gpr142;cytl1;ecm1;asgr2;stk38l;mc1r;gucy1a2;gpr45;prkca;rps6kb1;mms19;itgal;rab17;cd97;stk24;fbxw11;kiss1r;dgki;rin2;anxa3;otud7b;stat3;akap7;tle4;il18r1;ect2;c1s;csnk1g2;cabp2;p2ry1;dclk1;smad5;gdf5;npas2;cspg4;plcg1;mapkapk2;f2rl1;ncoa1;ralgps2;mapk14;gpr85;chl1;als2;duox2;tacr2;gpr158;igfbp2;cks2;ide;il19;wnt9a;grin2c;gtf2i;gabrr3;socs7;mcf2l;tlr5;ift57;adipoq;malt1;spred1;itgb7;cntnap2;darc;pla2g4b;lifr;tbc1d17;nphp4;p2rx4;notch2;tbc1d10b;bub1b;mapk8ip2;il12rb1;plcd4;bcl2l13;irak1;nppb;gpr171;ccr9;trim13;tbk1;ift88;tbc1d24;sdcbp;ric8b;rhobtb2;cd3d;ms4a2;gna15;slc20a1;gcg;cxcl12;epha5;ly6e;gna11;cxcl1;rxfp1;cntnap4;rasd1;arhgap25;dok1;trip10;ppp1r2;map2k1;taok2;brd8;fbxo8;rab35;adrb1;nmb;wnt4;asb16;ifnk;rab8b;rapgef1;gpr20;grk4;il17c;casp8ap2;vapb;pycard;hdgf;gpr84;fen1;rabgap1;gpr141;sh3gl2;ptpn1;rgl3;rassf4;rps6kb2;arhgap5;adcy3;rab7l1;irak2;wnt3a;traf1;gpr31;rgs1;sar1a;bmpr1a;il22ra2;gap43;stoml3;dapk2;txn;tlr6;il18rap;asb1;srpk2;lat;psen2;sh2d2a;mapk8ip3;obscn;cda;shc2;cdkl2;gucy2c;nod1;gprc5d;ccl19;iqgap1;avpr1a;ephb2;ccm2;ereg;mtnr1b;pxn;gng12;rgr;kif13b;ripk1;srf;faslg;nmur1;wwp1;cx3cr1;homer3;pkn3;notch4;ltb4r2;angpt1;prkacb;sgsm3;camk1;rab3d;rgs22;lep;tbc1d1;ppp1r1b;f2rl3;erg;arhgef5;dkk1;stambp;mctp2;taok3;gdf10;gpr15;ipo8;akap9;barx1;nts;grb10;cd19;unc5a;rab19;dgkg;ptprc;rasgef1a;plch2;plxnc1;nedd9;stx2;gng8;sfrp1;ncoa6;ephb6;rassf8;nfatc1;tlr3;ppt1;sphk1;gpbar1;sfn;tnfrsf11a;lhb;fyb;trim23;shank3;sgk2;gria1;pde3b;med24;bik;rasgrp3;arf4;mapk8ip1;tnfrsf21;scap;apba3;vipr2;bmp6;fgf7;lrrk2;sh2b3;smurf2;rps27;klrc2;gpr114;plcl2;kcnip1;angptl3;wdr67;ext2;pdap1;arhgap28;cd164;caskin1;impa1;fgb;atm;nod2;npbwr1;rassf3 |
| cell-cell adhesion; | 43#347 | 0.000108 | pcdhgb4;nptn;pcdhgb5;pcdha1;pcdhga11;pcdhgb6;cd2;pcdh15;pvrl3;dchs2;cdh16;icam5;cldn17;anxa9;cdh26;cldn4;dsg4;nrcam;dsc1;pcdhga1;pkp2;pcdhga5;pcdhga8;sox9;cdsn;col8a2;icam4;nphp4;ctnna3;dsg2;cldn14;cd47;pcdhb11;cd164;fzd6;cdh23;snai1;dlg1;crnn;cercam;pcdhb14;pcdhga10;pcdhb2 |
| amine metabolic process; | 68#636 | 0.000144 | uap1;idua;sardh;lars;sgpp1;naglu;ears2;qars;aldh5a1;pars2;arsb;gldc;dio1;tph1;baat;ftcd;farsb;snca;arg2;galnt5;sult1c2;prkca;afmid;sds;aarsd1;hal;gata3;rars;chst3;pdpr;ddc;mccc1;aoc3;gad2;hibadh;dalrd3;tmlhe;dtd1;hars2;padi6;psat1;sphk1;sult1b1;slco4a1;il22ra2;gne;amdhd2;qdpr;mat2b;mthfd1;dnajc13;gal3st3;hmgcl;hexb;mars;mat2a;thnsl1;got1;dmgdh;ext2;dhps;fam83d;nars;lipc;gfpt2;gmps;bckdhb;slc3a1 |
| multicellular organismal development#system development#organ development#organ morphogenesis; | 44#362 | 0.000158 | gnpat;srf;stx2;adora2b;gaa;cspg4;htatip2;edn1;tmem176b;notch4;wnt3a;foxn1;angpt1;ambn;sphk1;tuft1;pax3;nkx2-5;crx;pitx2;fgf1;egln1;sftpb;lhfpl5;atpif1;pou1f1;canx;sh2d2a;gypc;lhx3;angptl3;mmp19;lfng;notch2;fzd6;ccm2;c1galt1;ereg;znf22;smo;tnni3;nppb;ush1c;ift88 |
| heart development; | 17#94 | 0.000162 | srf;ncoa6;mkks;edn1;tcf25;pln;scube1;wnt3a;sox9;nkx2-6;nkx2-5;bmpr1a;gna11;egln1;bmp10;smo;tnni3 |
| phosphoinositide-mediated signaling; | 21#129 | 0.000164 | pik3c2a;chrm1;nmur1;edn1;gna15;sphk1;dgki;avpr1a;gna11;ndc80;bub1b;dgkg;gap43;cks2;pick1;mc3r;fen1;f2rl3;ptafr;ednra;p2ry1 |
| excretion; | 12#42 | 0.000167 | adora2b;tacr2;clcn5;aqp1;edn1;aqp3;nppb;scnn1a;unc13b;grhpr;slc26a3;ift88 |
| organelle organization and biogenesis; | 138#1526 | 0.000178 | exosc10;mapre1;sdcbp;map3k12;timm50;arsb;cxcl12;ndc80;arid1b;chmp1a;l3mbtl2;ttf1;ttn;cxcl1;kif5a;trip10;vps72;taok2;smarcb1;brd8;tert;taf5;epb41;asf1a;dnhd1;capg;cav2;bbs4;krt8;cenpj;krt4;smarcd2;brca1;myh4;tomm40;ube2b;cecr2;kif27;apc;katnb1;cntrob;cetn1;epb41l2;pbrm1;kif26b;bcar1;setd7;tomm34;hexb;arfip2;tmed10;tubgcp6;rfc1;rps14;slc25a36;map2;slc25a6;tubg1;sptbn2;dbn1;foxp3;kif13b;rab38;fgd5;nck1;shprh;dis3;slc25a1;kiss1;pes1;tubb3;clasp1;sirt6;coro2b;cdc23;fxc1;exosc7;wbp2nl;limch1;morf4l1;suv420h1;smarcad1;cpa4;prmt7;ftsj2;atg4c;tmem48;krt7;avil;dlg1;prdm15;dynlrb2;hspd1;brca2;snap29;pex19;cog2;dst;terf2;nedd9;arid4b;gaa;sirt2;cetn3;exosc8;kif1a;ppt1;nup98;sfn;als2;ssh1;ssh2;ucp3;kif3b;suv39h1;rcbtb1;pex13;cks2;yeats4;arpc3;tep1;slc25a37;kif18a;sdad1;atg12;msh4;ndufs8;smarce1;tsr2;nphp4;kif23;utp18;bub1b;pds5b;sycp3;msh2;ush1c;shroom1 |
| cell morphogenesis#regulation of cell size; | 29#206 | 0.00018 | emp3;kctd11;phf17;cdkn1a;arhgap5;morf4l1;taok2;brd8;sertad3;chpt1;emp1;cda;ppt1;sphk1;notch2;cd320;tnn;igfbp2;bmp10;gap43;yeats4;bcar1;brms1l;ndrg4;nppb;socs7;armc10;socs6;nedd9 |
| metabolic process#regulation of metabolic process; | 327#4150 | 0.000185 | mcm6;zfp62;tead4;rab18;znf648;znf566;psen1;furin;arid1b;znf300;l3mbtl2;znf275;znf187;zfpm2;znf644;vps72;lhx3;taf5;tlr9;znf181;asf1a;nr2e1;notch1;rxrg;myod1;eif4ebp1;glis2;pstk;hoxc10;leo1;zbtb5;capg;vgll3;rad54b;vsx1;mef2b;ncoa3;brca1;sox14;mael;edn1;pou5f2;myog;znf32;aff4;sp100;dmtf1;taf1b;nr1d1;pnrc2;nkx2-5;apc;upf1;pfdn5;ecd;smg5;pou1f1;znf532;carhsp1;pdzd3;foxj3;supt3h;rdbp;edf1;nfyb;znf529;arntl2;bcl11b;sox9;hexb;pbx4;ccna2;nr4a1;etv2;rfc1;rps14;map2;tcf15;irf2;ing4;uevld;foxp3;phb2;nr1h4;gtf2e1;phf17;nck1;ddx1;pqbp1;irf5;timp1;znf641;gck;pax3;clasp1;msc;serpina5;znf280d;rpl6;arg2;hivep1;tcf23;thrsp;dbp;morf4l1;zscan2;zbtb32;prkca;suv420h1;mms19;smarcad1;sertad3;lzts1;znf667;znf584;znf175;ebi3;stat3;avil;prdm15;sohlh2;tle4;znf629;brms1l;znf710;cbfa2t2;dpf3;arid4b;e2f6;mycbp2;mlxipl;smad5;sirt2;zkscan5;npas2;mycl1;ncoa1;ighmbp2;gbx1;hoxd8;hoxb13;ube2d3;znf576;jazf1;med18;nrbf2;pitx2;isl2;arpc3;znf713;gtf2i;pabpc1;adipoq;sox13;taf1a;hoxb4;dr1;znf329;med11;znf555;ssbp2;notch2;gtf3a;pum2;znf81;znf646;irak1;med6;znf672;ift88;zfp37;pogk;znf212;foxn1;znf524;znf35;cxcl12;yaf2;egln1;foxr1;chmp1a;ttf1;znf345;znf34;grhl3;tox3;znrd1;znf23;smarcb1;brd8;nr0b2;rab35;adrb1;tcf25;gata3;noto;znf470;ifnk;rab8b;znf683;znf688;znf395;nr4a3;casp8ap2;pycard;znf142;zbtb3;znf624;smarcd2;gtf2h2;ercc3;znf250;gabpa;tceb1;rfx2;ube2b;atxn3;uimc1;tbx10;sp2;katnb1;dyrk2;il22ra2;znf202;znf16;pbrm1;atpif1;znf569;pou2f3;pias4;tlr6;asb1;jdp2;eya2;cda;gtf2h5;dmrtb1;znf770;hes5;ilf3;ehf;ereg;znf22;zmiz2;klf8;znf238;rbak;sptbn2;srf;nfyc;tfeb;pcgf5;wwp1;hivep2;meox1;nkx1-2;notch4;elavl2;tfdp2;polr2f;phf20;snapc4;aire;zkscan1;rbm15;hsf4;eif5a;lep;sirt6;hoxc8;mef2d;l3mbtl3;erg;psip1;foxd4l1;foxn2;prrx2;pou5f1;nanos3;nkx2-6;barx1;ezh1;krt7;tigd2;efcab6;brca2;foxd2;zhx2;tbr1;terf2;elp4;ncoa6;htatip2;nfatc1;tlr3;paip2b;trim28;crx;nr1h3;pou2f2;suv39h1;rcbtb1;atf2;polr3g;med24;yeats4;bard1;scap;trmt6;bmp6;gzf1;cdkn1a;smurf2;mdm2;eif4g3;smarce1;nab1;ccdc59;elavl1;angptl3;rere;zbtb7a;cry2;atf5;nod2;etv1;npbwr1 |
| regulation of transferase activity; | 33#247 | 0.000187 | chrm1;adora2b;edn1;traf6;chek1;ccng1;cdc25c;tlr3;sphk1;als2;sfn;serinc2;apc;gap43;cks2;pick1;tlr6;ccna1;spred1;cdkn1a;malt1;prkca;taok2;vav2;dgki;gadd45a;ccna2;dgkg;ereg;ptprc;irak1;cks1b;pkia |
| positive regulation of cellular metabolic process; | 44#365 | 0.000194 | srf;smad5;ncoa6;npas2;ncoa1;ercc3;myog;notch4;tlr3;trim28;gck;nkx2-5;crx;dyrk2;arid1b;yaf2;yeats4;pou1f1;ttf1;tlr6;adipoq;scap;bmp6;prkca;mms19;smarcad1;sertad3;sox9;gata3;tlr9;ccna2;asf1a;ebi3;ilf3;glis2;ehf;ereg;zmiz2;irak1;ncoa3;med6;foxp3;brca1;ift88 |
| nitrogen compound metabolic process; | 71#678 | 0.000194 | uap1;idua;sardh;lars;sgpp1;naglu;ears2;qars;aldh5a1;pars2;arsb;gldc;dio1;tph1;baat;ftcd;farsb;snca;arg2;galnt5;sult1c2;prkca;afmid;sds;aarsd1;hal;gata3;rars;chst3;pdpr;ddc;mccc1;aoc3;gad2;hibadh;dalrd3;tmlhe;dtd1;hars2;padi6;psat1;nit2;sphk1;sult1b1;slco4a1;iscu;il22ra2;gne;amdhd2;qdpr;mat2b;mthfd1;dnajc13;gal3st3;hmgcl;hexb;mars;mat2a;thnsl1;got1;nqo1;dmgdh;ext2;dhps;fam83d;nars;lipc;gfpt2;gmps;bckdhb;slc3a1 |
| negative regulation of transcription; | 37#291 | 0.00023 | sirt2;wwp1;jazf1;uimc1;nkx2-5;sirt6;yaf2;znf202;chmp1a;znf345;pias4;gzf1;zbtb32;vps72;smurf2;nr0b2;dr1;mdm2;tcf25;sox9;nab1;smarce1;stat3;rps14;zbtb7a;ilf3;glis2;ereg;irf2;znf238;ing4;zhx2;foxp3;nr1h4;phb2;brca1;e2f6 |
| regulation of a molecular function; | 70#671 | 0.000261 | chrm1;adora2b;fgd5;tbc1d24;nmur1;traf6;gna15;ltb4r2;sgsm3;psen1;serinc2;egln1;arg2;pick1;tbc1d1;ppp1r2;prkca;taok2;adrb1;prmt7;gadd45a;dgki;tbc1d2;casp8ap2;dgkg;ptprc;pycard;rabgap1;ednra;pkia;nhej1;edn1;chek1;ccng1;psenen;cdc25c;tlr3;sphk1;als2;sfn;dph3;apc;gap43;cks2;atpif1;rasgrp3;tlr6;ift57;ccna1;spred1;cdkn1a;malt1;edf1;vav2;psen2;als2cr12;tbc1d23;tbc1d17;angptl3;wdr67;ccna2;tbc1d10b;avpr1a;ereg;bcl2l13;smo;irak1;nod2;cks1b;foxp3 |
| response to DNA damage stimulus; | 51#448 | 0.000272 | trex1;nhej1;gtf2h2;shprh;ung;ncoa6;rfc3;chek1;ercc3;fanca;ighmbp2;poll;parp4;ube2b;atrip;rad21;atxn3;sfn;upf1;apc;dyrk2;nudt1;mus81;bard1;rad52;mdc1;cdkn1a;mlh3;mms19;rif1;msh4;gtf2h5;gadd45a;ccna2;esco2;rfc5;wdr33;asf1a;parp1;poln;cry2;recql;rad54b;msh2;fancb;fen1;brca2;atm;cideb;msh5;brca1 |
| cell growth; | 28#201 | 0.000329 | emp3;kctd11;phf17;cdkn1a;morf4l1;taok2;brd8;sertad3;chpt1;emp1;cda;ppt1;sphk1;notch2;cd320;tnn;igfbp2;bmp10;gap43;yeats4;bcar1;brms1l;ndrg4;nppb;socs7;armc10;socs6;nedd9 |
| regulation of kinase activity; | 32#242 | 0.000332 | chrm1;adora2b;edn1;traf6;chek1;ccng1;cdc25c;tlr3;sphk1;als2;sfn;apc;gap43;cks2;pick1;tlr6;ccna1;spred1;cdkn1a;malt1;prkca;taok2;vav2;dgki;gadd45a;ccna2;dgkg;ereg;ptprc;irak1;cks1b;pkia |
| localization of cell#cell motility; | 45#383 | 0.000335 | sdcbp;nck1;atp1a1;cspg4;ltb4r2;mapk14;gas8;sphk1;chl1;ccr4;rtn4;ntf3;tnn;pex13;arpc3;dnah3;bcar1;fez2;txn;nrcam;sema4f;cald1;arpc1b;prkca;map2k1;mylip;taok2;itgal;vav2;cd97;pdpn;atp1a2;kiss1r;il16;arfip2;b3gnt2;angptl3;actb;stat3;coro1a;cav2;bmp10;smo;pxn;cercam |
| localization of cell; | 45#383 | 0.000335 | sdcbp;nck1;atp1a1;cspg4;ltb4r2;mapk14;gas8;sphk1;chl1;ccr4;rtn4;ntf3;tnn;pex13;arpc3;dnah3;bcar1;fez2;txn;nrcam;sema4f;cald1;arpc1b;prkca;map2k1;mylip;taok2;itgal;vav2;cd97;pdpn;atp1a2;kiss1r;il16;arfip2;b3gnt2;angptl3;actb;stat3;coro1a;cav2;bmp10;smo;pxn;cercam |
| negative regulation of transcription, DNA-dependent; | 28#203 | 0.000413 | gzf1;zbtb32;sirt2;vps72;nr0b2;dr1;tcf25;mdm2;sox9;smarce1;jazf1;nkx2-5;stat3;zbtb7a;rps14;ilf3;sirt6;znf202;chmp1a;irf2;ing4;znf238;znf345;zhx2;pias4;foxp3;nr1h4;e2f6 |
| regulation of cellular metabolic process; | 309#3933 | 0.000417 | mcm6;zfp62;tead4;rab18;znf648;znf566;psen1;furin;arid1b;znf300;l3mbtl2;znf275;znf187;zfpm2;znf644;vps72;lhx3;taf5;tlr9;znf181;asf1a;nr2e1;notch1;rxrg;myod1;eif4ebp1;glis2;pstk;hoxc10;leo1;zbtb5;vgll3;rad54b;vsx1;mef2b;ncoa3;brca1;sox14;mael;edn1;pou5f2;myog;znf32;aff4;sp100;dmtf1;taf1b;nr1d1;pnrc2;nkx2-5;upf1;pfdn5;smg5;pou1f1;znf532;carhsp1;pdzd3;foxj3;supt3h;rdbp;edf1;nfyb;znf529;arntl2;bcl11b;sox9;hexb;pbx4;ccna2;nr4a1;etv2;rfc1;rps14;tcf15;irf2;ing4;foxp3;phb2;nr1h4;gtf2e1;phf17;ddx1;pqbp1;irf5;timp1;znf641;gck;pax3;msc;serpina5;znf280d;rpl6;hivep1;tcf23;thrsp;dbp;morf4l1;zscan2;zbtb32;prkca;suv420h1;mms19;smarcad1;sertad3;lzts1;znf667;znf584;znf175;ebi3;stat3;prdm15;sohlh2;tle4;znf629;brms1l;znf710;cbfa2t2;dpf3;arid4b;e2f6;mycbp2;mlxipl;smad5;sirt2;zkscan5;npas2;mycl1;ncoa1;ighmbp2;gbx1;hoxd8;hoxb13;znf576;jazf1;med18;nrbf2;pitx2;isl2;znf713;gtf2i;pabpc1;adipoq;sox13;taf1a;hoxb4;dr1;med11;znf329;znf555;ssbp2;notch2;gtf3a;pum2;znf81;znf646;irak1;med6;znf672;ift88;zfp37;pogk;znf212;foxn1;znf524;znf35;yaf2;egln1;foxr1;chmp1a;ttf1;znf345;znf34;grhl3;tox3;znrd1;znf23;smarcb1;brd8;nr0b2;rab35;tcf25;noto;gata3;znf470;ifnk;rab8b;znf683;znf688;znf395;nr4a3;casp8ap2;pycard;znf142;zbtb3;znf624;smarcd2;gtf2h2;ercc3;znf250;gabpa;tceb1;rfx2;atxn3;uimc1;tbx10;sp2;dyrk2;il22ra2;znf202;znf16;pbrm1;atpif1;znf569;pou2f3;pias4;tlr6;asb1;jdp2;eya2;cda;gtf2h5;dmrtb1;znf770;hes5;ilf3;ehf;ereg;znf22;zmiz2;klf8;znf238;rbak;srf;nfyc;tfeb;pcgf5;wwp1;hivep2;meox1;nkx1-2;notch4;elavl2;tfdp2;polr2f;phf20;snapc4;aire;zkscan1;rbm15;hsf4;eif5a;sirt6;hoxc8;mef2d;l3mbtl3;erg;psip1;foxd4l1;foxn2;prrx2;pou5f1;nanos3;nkx2-6;barx1;ezh1;krt7;tigd2;efcab6;brca2;foxd2;zhx2;tbr1;terf2;elp4;ncoa6;htatip2;nfatc1;tlr3;paip2b;trim28;crx;nr1h3;pou2f2;suv39h1;rcbtb1;atf2;polr3g;med24;yeats4;bard1;scap;trmt6;bmp6;gzf1;cdkn1a;smurf2;mdm2;eif4g3;smarce1;nab1;ccdc59;elavl1;angptl3;rere;zbtb7a;cry2;atf5;etv1;nod2 |
| positive regulation of I-kappaB kinase/NF-kappaB cascade; | 17#99 | 0.00043 | malt1;faslg;fadd;traf6;slc20a1;tlr3;zdhhc17;nod1;casp8;ect2;ecm1;nod2;tlr6;ripk1;adipoq;trim13;tbk1 |
| regulation of I-kappaB kinase/NF-kappaB cascade; | 18#108 | 0.000444 | malt1;faslg;fadd;traf6;slc20a1;tlr3;zdhhc17;nod1;otud7b;casp8;ect2;ecm1;nod2;tlr6;ripk1;adipoq;trim13;tbk1 |
| nucleobase, nucleoside, nucleotide and nucleic acid metabolic process; | 440#5848 | 0.00046 | trex1;exosc10;mcm6;zfp62;map3k12;tead4;ppil3;snrpa;rab18;znf648;ears2;trim29;znf566;tbrg1;arid1b;farsb;fignl1;znf300;pick1;pth;l3mbtl2;znf275;cilp;larp7;znf187;zfpm2;calcoco1;mdc1;atp6v1e2;znf644;vps72;rbms2;lhx3;rars;taf5;znf181;wdr33;asf1a;nr2e1;notch1;rxrg;poln;nom1;pop5;myod1;il27ra;glis2;hoxc10;leo1;zbtb5;vgll3;rad54b;vsx1;mef2b;ncoa3;hars2;mettl4;brca1;sox14;endog;tk1;mael;ung;rfc3;edn1;pou5f2;myog;znf32;aff4;sp100;dmtf1;cecr2;taf1b;nr1d1;pnrc2;nkx2-5;upf1;pfdn5;ecd;dhodh;gne;smg5;pou1f1;znf532;carhsp1;pdzd3;foxj3;setd7;txnl4b;supt3h;rdbp;dhx35;edf1;nfyb;znf529;cherp;arntl2;bcl11b;sox9;pbx4;mars;ccna2;nr4a1;etv2;rfc1;rps14;recql;tcf15;irf2;ak3;ing4;foxp3;phb2;nr1h4;gtf2e1;phf17;cdc73;ddx1;thoc3;pqbp1;irf5;znf641;pax3;msc;znf280d;rpl6;hivep1;tcf23;thrsp;dbp;morf4l1;gucy1a2;zscan2;zbtb32;suv420h1;mms19;cpa4;sertad3;smarcad1;lzts1;znf667;pcbp2;znf584;nudt21;gadd45a;znf175;ftsj2;sf3a1;stat3;prpf3;prdm15;dalrd3;sohlh2;tle4;fancb;znf629;brms1l;znf710;cbfa2t2;dpf3;arid4b;e2f6;mycbp2;mlxipl;smad5;sirt2;zkscan5;npas2;mycl1;ncoa1;fanca;ighmbp2;rpp38;gbx1;hoxd8;hoxb13;atrip;znf576;jazf1;sf3b3;med18;nrbf2;pitx2;isl2;znf713;gtf2i;mcm10;mus81;pola2;pabpc1;prpf40a;sox13;taf1a;hoxb4;dr1;znf329;med11;ppfibp2;znf555;ssbp2;tsr2;notch2;gtf3a;trip11;utp18;znf81;fam83d;rbm17;znf646;trmu;irak1;msh2;med6;pop1;sphar;atp6v1a;znf672;lars;zfp37;znf212;pogk;foxn1;znf524;qars;znf35;upp2;yaf2;egln1;foxr1;chmp1a;ttf1;znf345;znf34;grhl3;tox3;rad52;znrd1;znf23;smarcb1;nr0b2;brd8;aarsd1;rab35;tert;tcf25;gata3;noto;sart3;ifnk;znf470;rab8b;znf683;znf688;znf395;nr4a3;casp8ap2;hibadh;pycard;znf142;fen1;rexo2;znf624;zbtb3;smarcd2;crnkl1;adcy3;gtf2h2;ercc3;znf250;cpsf1;gabpa;parp4;ube2b;rfx2;tceb1;atxn3;uimc1;tbx10;sp2;tsen2;znf202;prpf4b;atp6v0b;znf16;pbrm1;hnrnpl;atpif1;znf569;pou2f3;pias4;mthfd1;skiv2l2;mif4gd;srpk2;jdp2;rpap1;eya2;cda;gtf2h5;atp5b;dmrtb1;gucy2c;znf770;rfc5;hes5;guk1;ilf3;ehf;ereg;znf22;zmiz2;gda;klf8;clp1;znf238;msh5;atp5f1;rbak;srf;uap1;slu7;shprh;nfyc;tfeb;pcgf5;wwp1;dis3;hivep2;meox1;nkx1-2;notch4;elavl2;eif2c3;tsn;poll;tfdp2;polr2f;pnpt1;phf20;rad21;pars2;snapc4;aire;zkscan1;rbm15;hsf4;sirt6;rpp14;l3mbtl3;hoxc8;mef2d;dctd;nudt1;papolb;atp5g2;erg;psip1;foxd4l1;dnase1l2;foxn2;prrx2;exosc7;pou5f1;rnase6;zmat5;nkx2-6;barx1;prmt7;esco2;parp1;qtrtd1;ezh1;hat1;krt7;piwil1;isy1;tigd2;efcab6;brca2;foxd2;zhx2;tbr1;terf2;elp4;nhej1;ncoa6;htatip2;chek1;mto1;exosc8;nfatc1;tlr3;ppt1;nup98;pold2;trim28;crx;ddx41;dhx38;nr1h3;pou2f2;hnrnpr;suv39h1;atf2;rcbtb1;polr3g;yeats4;med24;tep1;polr3d;prpsap1;bard1;scap;trmt6;bmp6;gzf1;dnajc13;mlh3;smurf2;msh4;eif4g3;mdm2;nab1;ccdc59;smarce1;snrpd3;elavl1;ppig;rere;zbtb7a;cry2;sycp3;nars;gmps;atm;atf5;nod2;etv1;lsm10;ssbp1 |
| meiosis I; | 10#33 | 0.000471 | rad52;mlh3;chek1;msh4;cks2;sycp3;piwil2;msh5;atm;rad21 |
| regulation of protein kinase activity; | 31#237 | 0.000576 | chrm1;adora2b;malt1;cdkn1a;spred1;prkca;taok2;edn1;traf6;chek1;ccng1;cdc25c;tlr3;sphk1;gadd45a;dgki;ccna2;als2;sfn;apc;ereg;dgkg;ptprc;gap43;cks2;irak1;pick1;tlr6;cks1b;pkia;ccna1 |
| regulation of biosynthetic process; | 29#217 | 0.000626 | ddx1;nanos3;edn1;eif4g3;gata3;tlr9;tlr3;paip2b;ebi3;gck;upf1;furin;pum2;eif4ebp1;eif5a;krt7;dyrk2;pstk;ereg;arg2;pdzd3;tlr6;foxp3;adipoq;scap;asb1;brca1;trmt6;bmp6 |
| regulation of catalytic activity; | 63#605 | 0.000725 | chrm1;adora2b;fgd5;nhej1;tbc1d24;nmur1;edn1;traf6;chek1;psenen;ccng1;gna15;ltb4r2;cdc25c;tlr3;sgsm3;sphk1;als2;sfn;psen1;serinc2;apc;gap43;cks2;arg2;pick1;tbc1d1;atpif1;rasgrp3;tlr6;ift57;ccna1;malt1;cdkn1a;spred1;ppp1r2;prkca;taok2;psen2;vav2;als2cr12;adrb1;tbc1d23;tbc1d17;dgki;angptl3;gadd45a;ccna2;wdr67;tbc1d10b;avpr1a;tbc1d2;casp8ap2;ereg;dgkg;ptprc;pycard;bcl2l13;irak1;rabgap1;cks1b;ednra;pkia |
| I-kappaB kinase/NF-kappaB cascade; | 21#139 | 0.000767 | malt1;faslg;fadd;traf6;irak2;slc20a1;tlr3;nkiras2;zdhhc17;nod1;otud7b;casp8;irak1;ect2;ecm1;nod2;tlr6;adipoq;ripk1;trim13;tbk1 |
| circadian rhythm; | 9#29 | 0.000944 | aanat;crx;cry2;npas2;hebp1;arntl2;slc9a3;mat2a;nr1d1 |
| response to extracellular stimulus; | 14#66 | 0.000992 | sstr2;cdkn1a;cyp11a1;avpr1a;lep;alb;cfb;gcgr;chmp1a;lct;abcg5;acads;brca2;aldob |
| cellular catabolic process; | 79#809 | 0.001009 | exosc10;sardh;lyzl1;usp30;pnpt1;aldh5a1;hsd17b11;upp2;gck;gldc;arg2;cdc23;usp2;adamts13;gba3;dnase1l2;exosc7;smpd1;psmf1;rnase6;fbxo8;afmid;suclg1;hal;pfkp;lyg2;psmb6;ldha;pdpr;arih1;mccc1;gad2;hibadh;dtd1;usp49;zhx2;gaa;usp29;usp26;usp35;ercc3;usp7;ppt1;usp46;sphk1;usp10;tceb1;aldob;ube2b;ube2d3;cecr2;duox2;upf1;ecd;smg5;psma6;psmb4;qdpr;fut10;mthfd1;smurf2;cda;pla2g4b;hexb;elavl1;plcb3;dmgdh;got1;usp48;dhps;hk1;usp45;pop1;lipc;uevld;usp53;bckdhb;fbxl7;pgk2 |
| growth#regulation of growth; | 27#201 | 0.001042 | kctd11;phf17;cdkn1a;morf4l1;taok2;brd8;sertad3;adrb1;chpt1;tkt;cda;ppt1;sphk1;hoxb13;cd320;igfbp2;gap43;bmp10;yeats4;bcar1;brms1l;nppb;socs7;ing4;armc10;socs6;nedd9 |
| induction of apoptosis; | 28#212 | 0.001117 | tia1;cdkn1a;faslg;prkca;fadd;htatip2;ercc3;cd2;siva1;unc13b;sfn;trim35;notch2;dyrk2;ppp1r13b;casp8ap2;ptprc;rps3a;pycard;cidec;bcl2l13;il19;pth;bik;brca2;dapk2;cideb;brca1 |
| glycoprotein biosynthetic process; | 25#182 | 0.001224 | gcnt3;galnt1;gcnt4;pomgnt1;alg2;alg5;gypc;pigb;phlda1;b3gnt3;st6gal2;cd37;sdf2;b3gnt2;st6galnac1;b3gat1;fut11;fut3;mgat2;lipc;gal3st1;cog2;fut10;mpdu1;b3galt5 |
| regulation of cell growth; | 23#162 | 0.001224 | kctd11;phf17;cdkn1a;morf4l1;taok2;brd8;sertad3;chpt1;cda;ppt1;sphk1;cd320;igfbp2;gap43;bmp10;yeats4;bcar1;brms1l;nppb;socs7;socs6;armc10;nedd9 |
| induction of programmed cell death; | 28#213 | 0.001224 | tia1;cdkn1a;faslg;prkca;fadd;htatip2;ercc3;cd2;siva1;unc13b;sfn;trim35;notch2;dyrk2;ppp1r13b;casp8ap2;ptprc;rps3a;pycard;cidec;bcl2l13;il19;pth;bik;brca2;dapk2;cideb;brca1 |
| innate immune response; | 18#114 | 0.001224 | cfi;pglyrp1;cfb;c8b;tlr9;tlr3;ifnk;il1rl2;crisp3;masp1;ereg;il18r1;c1qc;tlr5;nod2;tlr6;il18rap;tbk1 |
| response to steroid hormone stimulus; | 10#37 | 0.001272 | cyp11a1;map2k1;cav2;lct;gata3;acads;brca2;aldob;lcat;brca1 |
| positive regulation of catalytic activity; | 32#256 | 0.001274 | chrm1;adora2b;nhej1;nmur1;edn1;traf6;psenen;gna15;tlr3;sphk1;als2;psen1;serinc2;gap43;pick1;tlr6;ift57;malt1;taok2;vav2;psen2;adrb1;dgki;avpr1a;casp8ap2;dgkg;ereg;pycard;ptprc;bcl2l13;irak1;ednra |
| positive regulation of nucleobase, nucleoside, nucleotide and nucleic acid metabolic process; | 35#289 | 0.001305 | srf;smad5;ncoa6;npas2;ncoa1;ercc3;myog;notch4;trim28;nkx2-5;crx;arid1b;yaf2;yeats4;pou1f1;ttf1;scap;bmp6;mms19;smarcad1;sertad3;sox9;gata3;ccna2;asf1a;ilf3;glis2;ehf;ereg;zmiz2;irak1;med6;ncoa3;foxp3;brca1 |
| positive regulation of transcription; | 34#279 | 0.001421 | srf;smad5;ncoa6;npas2;ncoa1;ercc3;myog;notch4;trim28;nkx2-5;crx;arid1b;yaf2;yeats4;pou1f1;ttf1;scap;bmp6;mms19;smarcad1;sertad3;sox9;gata3;ccna2;asf1a;ilf3;ehf;glis2;zmiz2;irak1;med6;ncoa3;foxp3;brca1 |
| G-protein signaling, coupled to IP3 second messenger (phospholipase C activating); | 15#88 | 0.00146 | chrm1;avpr1a;gna11;nmur1;dgkg;edn1;gap43;pick1;gna15;mc3r;f2rl3;sphk1;dgki;p2ry1;ednra |
| protein amino acid glycosylation; | 24#174 | 0.001518 | gcnt3;galnt1;gcnt4;pomgnt1;alg2;alg5;gypc;pigb;b3gnt3;st6gal2;cd37;b3gnt2;sdf2;st6galnac1;b3gat1;fut11;fut3;mgat2;lipc;gal3st1;cog2;fut10;mpdu1;b3galt5 |
| ectoderm development; | 21#146 | 0.001962 | stx2;fgf7;krt13;aldh3a2;ercc3;emp1;sox9;cdsn;foxn1;rbp2;hoxb13;sfn;bmpr1a;mreg;snai1;ugcg;evpl;krt14;pou2f3;dsg4;casp14 |
| multicellular organismal development#system development#organ development#tissue development; | 36#305 | 0.001962 | stx2;krt13;aldh3a2;edn1;ercc3;wnt3a;foxn1;ambn;hoxb13;sfn;tnfrsf11a;tuft1;igsf10;bmpr1a;mreg;ugcg;evpl;krt14;pou2f3;dsg4;trim15;casp14;bmp6;znrd1;fgf7;eya2;mesdc2;emp1;sox9;cdsn;rbp2;chrdl2;barx1;tcf15;snai1;smo |
| nuclear transport; | 22#156 | 0.001966 | malt1;aaas;htatip2;nfkbie;rpl23;ipo8;anp32a;camk1;nup98;fyb;upf1;mbtps1;rere;gckr;tbrg1;cry2;smg5;pou1f1;nup160;pola2;bard1;gsk3b |
| membrane lipid metabolic process; | 33#272 | 0.001984 | pigq;a4galt;sgpp1;chpt1;pigs;pla2g12a;pigb;ppt1;pitpnm3;fabp3;sphk1;aldh5a1;serinc2;pisd;ugcg;snca;sftpb;gpaa1;proca1;smpd1;pik3c2a;flrt2;sgms2;agpat4;pla2g4b;hexb;plcb3;pla2g12b;sptlc1;pigz;impa1;lipc;gal3st1 |
| homeostatic process; | 51#480 | 0.00209 | srf;mall;atp1a1;edn1;f2rl1;cav1;gna15;ppt1;pcdh15;dld;ccr4;cxcl12;gck;slc34a3;txnrd3;gckr;abca12;egln1;sftpb;pde3b;pth;slc9a1;txn;pdia6;bard1;adipoq;prkca;mkks;txndc8;adrb1;pln;fth1;hexb;atp1a2;angptl3;ccl19;avpr1a;hp;cdh23;tpt1;ptprc;tnni3;cutc;nppb;slc9a3;ccr3;ush1c;ccr9;foxp3;ednra;clcn6 |
| biopolymer glycosylation; | 24#177 | 0.002126 | gcnt3;galnt1;gcnt4;pomgnt1;alg2;alg5;gypc;pigb;b3gnt3;st6gal2;cd37;b3gnt2;sdf2;st6galnac1;b3gat1;fut11;fut3;mgat2;lipc;gal3st1;cog2;fut10;mpdu1;b3galt5 |
| membrane organization and biogenesis; | 34#284 | 0.002126 | stx2;a4galt;lrp3;atg7;izumo1;rab18;timm50;cd2;ppt1;serpina5;clcn5;ehd1;lrp10;pick1;fxc1;asgr2;lrp1;epn2;tom1;nrcam;stx11;amph;trip10;asgr1;abca7;ap1s3;napg;rin2;napa;sh3bp4;lrp1b;slc9a3;snap29;sh3gl2 |
| response to drug; | 13#63 | 0.002136 | slc18a1;scn11a;bcar3;snca;cav2;lct;snx27;abcg5;kcnj11;mat2a;parp4;pdzk1;abca3 |
| regulation of cellular biosynthetic process; | 26#199 | 0.002428 | ddx1;nanos3;eif4g3;gata3;tlr9;tlr3;paip2b;ebi3;gck;upf1;furin;pum2;eif4ebp1;eif5a;krt7;dyrk2;pstk;ereg;tlr6;foxp3;adipoq;scap;asb1;brca1;trmt6;bmp6 |
| monocarboxylic acid metabolic process; | 34#287 | 0.002724 | gnpat;acot9;cpt1c;acaca;me3;aldob;aldh5a1;elovl7;gck;lep;baat;ftcd;pex13;tbxas1;acsl1;mthfd1;adipoq;ptgis;acsl5;sds;pnpla8;cpt1b;pla2g4b;acads;acaa2;mccc1;lypla2;tmlhe;slc27a3;ltc4s;lipc;lypla1;nr1h4;brca1 |
| carboxylic acid metabolic process; | 76#795 | 0.002744 | sardh;acot9;lars;cpt1c;lias;acaca;ears2;qars;aldh5a1;pars2;elovl7;mcfd2;gck;gldc;tph1;lep;baat;ftcd;farsb;tbxas1;arg2;ptgis;prkca;afmid;sds;aarsd1;hal;rars;pdpr;ddc;mccc1;gad2;hibadh;lypla2;dalrd3;slc27a3;tmlhe;dtd1;hars2;padi6;brca1;gnpat;psat1;me3;aldob;il22ra2;gne;pex13;acsl1;qdpr;adipoq;mat2b;mthfd1;acsl5;dnajc13;hmgcl;pnpla8;cpt1b;pla2g4b;acads;acaa2;mars;mat2a;thnsl1;got1;dmgdh;fam83d;nars;lipc;ltc4s;gfpt2;lypla1;gmps;bckdhb;nr1h4;slc3a1 |
| multicellular organismal process#regulation of multicellular organismal process; | 33#277 | 0.002977 | nck1;cfi;edn1;traf6;cfb;cd2;sphk1;ntf3;lep;c1qc;bcar1;cdkn1a;malt1;prkca;lat;kcnq1;adrb1;pln;c8b;atp1a2;pag1;ifnk;ebi3;c4a;cd47;il27ra;masp1;ereg;ptprc;tnni3;nppb;nod2;foxp3 |
| regulation of cyclin-dependent protein kinase activity; | 11#49 | 0.003078 | apc;cdkn1a;chek1;cks2;ccng1;cdc25c;gadd45a;ccna2;sfn;cks1b;ccna1 |
| organic acid metabolic process; | 76#798 | 0.003078 | sardh;acot9;lars;cpt1c;lias;acaca;ears2;qars;aldh5a1;pars2;elovl7;mcfd2;gck;gldc;tph1;lep;baat;ftcd;farsb;tbxas1;arg2;ptgis;prkca;afmid;sds;aarsd1;hal;rars;pdpr;ddc;mccc1;gad2;hibadh;lypla2;dalrd3;slc27a3;tmlhe;dtd1;hars2;padi6;brca1;gnpat;psat1;me3;aldob;il22ra2;gne;pex13;acsl1;qdpr;adipoq;mat2b;mthfd1;acsl5;dnajc13;hmgcl;pnpla8;cpt1b;pla2g4b;acads;acaa2;mars;mat2a;thnsl1;got1;dmgdh;fam83d;nars;lipc;ltc4s;gfpt2;lypla1;gmps;bckdhb;nr1h4;slc3a1 |
| DNA repair; | 43#392 | 0.003078 | trex1;nhej1;gtf2h2;shprh;ung;ncoa6;rfc3;chek1;ercc3;fanca;ighmbp2;poll;parp4;ube2b;atrip;rad21;atxn3;upf1;nudt1;mus81;rad52;mdc1;mlh3;mms19;msh4;gtf2h5;gadd45a;rfc5;esco2;asf1a;parp1;wdr33;poln;cry2;recql;rad54b;msh2;fancb;fen1;brca2;atm;msh5;brca1 |
| sensory perception of light stimulus; | 28#223 | 0.003103 | rbp4;rpgrip1;cryga;mfrp;mkks;rabggta;mertk;myo3a;ppt1;pcdh15;rho;gprc5d;ppef2;bbs10;crx;dhrs3;crybb1;best1;cdh23;prpf3;bbs4;vsx1;nyx;rbp3;rgr;ush1c;prph2;wfs1 |
| visual perception; | 28#223 | 0.003103 | rbp4;rpgrip1;cryga;mfrp;mkks;rabggta;mertk;myo3a;ppt1;pcdh15;rho;gprc5d;ppef2;bbs10;crx;dhrs3;crybb1;best1;cdh23;prpf3;bbs4;vsx1;nyx;rbp3;rgr;ush1c;prph2;wfs1 |
| biosynthetic process; | 198#2454 | 0.003146 | pigq;rpl5;lars;galnt1;alg2;mrpl30;acaca;b3gnt3;ears2;qars;gys2;gphn;pbld;furin;tph1;farsb;rpl28;gpaa1;ccbl2;rps9;ptgis;atp6v1e2;aarsd1;hal;sgms2;gata3;rars;chst3;tlr9;rpl12;st6gal2;ddc;phkg2;eif4ebp1;sptlc1;pstk;tmlhe;coq3;hars2;zdhhc1;brca1;rps6kb2;psat1;a4galt;adcy3;cyp11a1;edn1;atg7;rabggta;rpl23;chpt1;aldob;dph3;upf1;pisd;dhodh;gne;dyrk2;atp6v0b;rpl8;fut3;pdzd3;eif4a1;tlr6;mthfd1;mat2b;asb1;pik3c2a;gcnt3;gal3st3;gcnt4;hexb;atp5b;rps15a;mars;mrpl24;gucy2c;nod1;guk1;rps14;ereg;rps3a;gfpt2;lipc;atp5f1;gal3st1;foxp3;mpdu1;rpl18a;uap1;ddx1;rpl7l1;lias;mrpl43;rrbp1;abtb1;eif2c3;flad1;fabp3;sdf2;pars2;hsd17b11;elovl7;gck;mocs3;st6galnac1;hs3st3b1;b3gat1;dio1;eif5a;ugcg;dctd;snca;rpl6;alas1;tbxas1;arg2;galnt5;fdft1;atp5g2;micall1;lcat;hsd17b12;phkg1;b3galt5;gucy1a2;nanos3;sds;gypc;agpat4;rpl39;rplp1;b3gnt2;glt25d2;qtrtd1;ebi3;stard5;gmppa;rps5;rps23;krt7;gad2;ptprc;dalrd3;hmgcs1;cercam;rsad1;cog2;padi6;hsd17b2;eef1a2;pank4;pomgnt1;alg5;pigs;pigb;phlda1;tlr3;cd37;paip2b;duox2;mrpl17;fut11;mrps5;il19;qdpr;fut10;prpsap1;adipoq;scap;trmt6;bmp6;dnajc13;rps27;idi1;mrpl49;eif4g3;mrpl9;acaa2;mat2a;thnsl1;nqo1;got1;ext2;pum2;dhps;mtfmt;fam83d;mrps14;chat;pigz;nars;impa1;mgat2;ltc4s;gmps;mrpl11;atp6v1a |
| RNA processing; | 54#525 | 0.003303 | exosc10;slu7;ddx1;dis3;mto1;ppil3;snrpa;thoc3;exosc8;rpp38;cpsf1;pnpt1;sf3b3;upf1;dhx38;ddx41;hnrnpr;tsen2;prpf4b;rpp14;hnrnpl;papolb;pabpc1;prpf40a;larp7;exosc7;skiv2l2;bard1;txnl4b;trmt6;srpk2;dhx35;rbms2;cherp;zmat5;sart3;snrpd3;nudt21;tsr2;ftsj2;ppig;qtrtd1;sf3a1;rps14;pop5;utp18;rbm17;prpf3;isy1;trmu;pop1;clp1;lsm10;crnkl1 |
| negative regulation of nucleotide metabolic process; | 4#6 | 0.00334 | cda;atpif1;pdzd3;edn1 |
| multicellular organismal development#embryonic development; | 29#235 | 0.003409 | sh2b3;smad5;gdf5;smarcb1;eya2;mfrp;ncoa6;edn1;pitpnb;plcg1;notch4;wnt3a;nkx2-6;pbx4;ptprr;hoxd8;thbd;nkx2-5;shank3;fzd6;egln1;lhfpl5;pick1;brca2;trim15;ush1c;dkk1;fut10;ift88 |
| chemical homeostasis; | 35#302 | 0.003493 | mall;atp1a1;edn1;f2rl1;cav1;gna15;ppt1;ccr4;cxcl12;gck;slc34a3;gckr;egln1;pde3b;pth;slc9a1;adipoq;prkca;pln;fth1;hexb;atp1a2;angptl3;ccl19;hp;avpr1a;tpt1;ptprc;tnni3;cutc;slc9a3;nppb;ccr3;ccr9;ednra |
| immune system development; | 24#182 | 0.003494 | nhej1;sh2b3;smad5;ncoa6;dyrk3;cd3d;notch4;wnt3a;sfxn1;tlr3;cd2;poll;timp1;rab3d;tnfrsf11a;notch2;nkx2-5;il27ra;il17c;lep;ptprc;trim10;tpd52;fut10 |
| inflammatory response; | 34#291 | 0.003539 | ly96;cfi;cfb;irak2;s100a8;scube1;tlr3;atrn;parp4;ccr4;cxcl12;mmp25;c1qc;ptafr;tlr5;cxcl1;tlr6;il18rap;bmp6;prkca;itgal;cd97;c8b;pla2g4b;mefv;tlr9;nod1;ccl19;c4a;stat3;aoc3;masp1;il17c;ccr3 |
| cyclic-nucleotide-mediated signaling; | 18#122 | 0.003717 | adora2b;oprd1;adrb1;ltb4r2;prkacb;ptgir;rapgef2;sstr2;rgs1;lhcgr;mtnr1b;gcgr;mc3r;pth;ccr3;pdzd3;ednra;mc1r |
| female meiosis; | 3#3 | 0.003775 | adcy3;ereg;sycp3 |
| male meiosis#male meiosis I; | 3#3 | 0.003775 | brca2;ccna1;sycp3 |
| amine catabolic process; | 15#94 | 0.003915 | mccc1;gldc;sardh;dhps;gad2;afmid;hal;arg2;dtd1;qdpr;bckdhb;dmgdh;got1;mthfd1;pdpr |
| chromosome segregation; | 12#59 | 0.003925 | psen1;apc;ndc80;psen2;dsn1;pds5b;sycp3;chmp1a;cdc23;arl8b;rad21;brca1 |
| positive regulation of immune response; | 14#85 | 0.003964 | malt1;c4a;il27ra;cfi;masp1;ereg;traf6;ptprc;cfb;c8b;c1qc;bcar1;nod2;ifnk |
| anatomical structure morphogenesis#anatomical structure formation; | 21#153 | 0.004196 | adora2b;eya2;canx;cspg4;htatip2;edn1;sh2d2a;notch4;wnt3a;angpt1;sphk1;casc5;mmp19;angptl3;fgf1;c1galt1;ereg;tnni3;atpif1;nppb;trim15 |
| meiotic recombination; | 7#22 | 0.004196 | rad52;mlh3;atm;msh5;rad21;chek1;msh4 |
| negative regulation of apoptosis; | 28#227 | 0.004204 | malt1;cdkn1a;stambp;eef1a2;sfrp1;api5;alb;htatip2;ppt1;proc;sphk1;rtn4;notch2;psen1;akt1s1;ntf3;tpt1;bcl2a1;mapk8ip2;naip;snca;smo;sycp3;atf5;aven;pik3r2;bard1;gsk3b |
| protein modification by small protein conjugation; | 14#77 | 0.004204 | fbxl3;trim23;mylip;wwp1;traf6;fbxw11;fbxo10;mdm2;atg3;sae1;pias4;anapc11;bard1;brca1 |
| response to nutrient levels; | 12#60 | 0.00448 | sstr2;cyp11a1;lep;alb;cfb;gcgr;chmp1a;lct;abcg5;acads;brca2;aldob |
| positive regulation of immune system process; | 14#86 | 0.004641 | malt1;c4a;il27ra;cfi;masp1;ereg;traf6;ptprc;cfb;c8b;c1qc;bcar1;nod2;ifnk |
| positive regulation of multicellular organismal process; | 16#105 | 0.004925 | malt1;cfi;traf6;adrb1;cfb;c8b;sphk1;ifnk;c4a;il27ra;masp1;ereg;ptprc;bcar1;c1qc;nod2 |
| nitrogen compound catabolic process; | 15#96 | 0.005268 | mccc1;gldc;sardh;dhps;gad2;afmid;hal;arg2;dtd1;qdpr;bckdhb;dmgdh;got1;mthfd1;pdpr |
| activation of protein kinase activity; | 13#70 | 0.005356 | chrm1;malt1;taok2;dgkg;edn1;traf6;gap43;irak1;pick1;tlr3;sphk1;dgki;tlr6 |
| epidermis development; | 19#135 | 0.005356 | fgf7;krt13;aldh3a2;ercc3;emp1;sox9;cdsn;foxn1;rbp2;hoxb13;sfn;mreg;snai1;ugcg;evpl;krt14;pou2f3;dsg4;casp14 |
| negative regulation of programmed cell death; | 28#230 | 0.005399 | malt1;cdkn1a;stambp;eef1a2;sfrp1;api5;alb;htatip2;ppt1;proc;sphk1;rtn4;notch2;psen1;akt1s1;ntf3;tpt1;bcl2a1;mapk8ip2;naip;snca;smo;sycp3;atf5;aven;pik3r2;bard1;gsk3b |
| amino acid catabolic process; | 14#87 | 0.005413 | mccc1;gldc;sardh;gad2;afmid;hal;arg2;dtd1;qdpr;bckdhb;dmgdh;got1;mthfd1;pdpr |
| cellular calcium ion homeostasis; | 17#116 | 0.005863 | prkca;edn1;f2rl1;pln;gna15;hexb;ccr4;ccl19;cxcl12;avpr1a;tpt1;ptprc;tnni3;pth;ccr3;ccr9;ednra |
| calcium ion homeostasis; | 17#116 | 0.005863 | prkca;edn1;f2rl1;pln;gna15;hexb;ccr4;ccl19;cxcl12;avpr1a;tpt1;ptprc;tnni3;pth;ccr3;ccr9;ednra |
| system process; | 130#1539 | 0.006123 | sdcbp;slc8a1;mertk;corin;myo3a;scnn1a;nptx2;cxcl12;lamb2;tmod4;gna11;tph1;mylk2;stx1a;trpv2;ttn;kif5a;amph;oprd1;adrb1;pln;gata3;epb41;cldn14;cdh23;cav2;bbs4;mog;vsx1;tnni3;cplx3;ednra;adcy3;myh4;strn4;nptn;edn1;rabggta;ercc3;ncam1;atxn3;htr3a;bbs10;sbf2;crybb1;cplx1;myo15a;rtp3;grik5;hexb;unc13b;rho;gprc5d;ppef2;slc26a3;ace;dhrs3;pcdhb11;avpr1a;scn11a;coch;mtnr1b;gcgr;rgr;gal3st1;dbn1;adora2b;chrm1;rpgrip1;rbp4;cryga;nmur1;aqp1;soat1;aldh5a1;pax3;ntf3;rtp4;lep;snca;kcnma1;aqp3;arg2;lhfpl5;stx1b;rbp3;prph2;grhpr;prkca;myl3;mkks;kcnq1;cacng1;atp1a2;akap9;b3gnt2;nts;actb;best1;gad2;prpf3;dag1;pcdhb2;gaa;abcg5;ppt1;sphk1;pcdh15;als2;tnfrsf11a;crx;tacr2;clcn5;gria1;gabrr3;cald1;cntnap2;mfrp;kcnip1;nqo1;chat;nyx;fgb;hap1;nppb;pcdhb14;npbwr1;ush1c;wfs1;ift88 |
| postreplication repair; | 4#7 | 0.00643 | msh2;ube2b;wdr33;brca1 |
| blood vessel development#blood vessel morphogenesis; | 20#147 | 0.006582 | adora2b;canx;cspg4;htatip2;edn1;sh2d2a;notch4;angpt1;sphk1;mmp19;angptl3;nkx2-5;ccm2;fgf1;c1galt1;ereg;smo;tnni3;atpif1;nppb |
| negative regulation of transcription from RNA polymerase II promoter; | 18#127 | 0.006747 | gzf1;zbtb32;vps72;nr0b2;dr1;tcf25;mdm2;jazf1;nkx2-5;stat3;zbtb7a;znf202;irf2;znf345;znf238;pias4;nr1h4;e2f6 |
| immune response#regulation of immune response; | 15#98 | 0.00696 | malt1;c4a;il27ra;cfi;masp1;ereg;traf6;ptprc;cfb;c8b;c1qc;bcar1;nod2;ifnk;foxp3 |
| I-kappaB kinase/NF-kappaB cascade#activation of NF-kappaB-inducing kinase; | 5#12 | 0.007153 | irak1;malt1;tlr3;traf6;tlr6 |
| behavioral interaction between organisms; | 5#12 | 0.007153 | hexb;cyp11a1;avpr1a;mkks;pex13 |
| Golgi vesicle transport; | 14#89 | 0.007353 | sar1a;trappc1;napa;stx16;sec31a;pick1;gopc;sec24a;gosr1;stx18;tmed10;cog2;napg;osbpl5 |
| positive regulation of transcription, DNA-dependent; | 27#223 | 0.007396 | srf;smad5;mms19;ncoa6;npas2;ncoa1;ercc3;myog;notch4;gata3;sox9;asf1a;trim28;nkx2-5;crx;ilf3;ehf;arid1b;zmiz2;yeats4;ncoa3;med6;pou1f1;ttf1;scap;brca1;bmp6 |
| protein ubiquitination; | 13#73 | 0.007545 | fbxl3;trim23;mylip;wwp1;traf6;fbxw11;fbxo10;mdm2;atg3;sae1;anapc11;bard1;brca1 |
| determination of symmetry; | 6#18 | 0.007545 | wnt3a;kif3b;pitx2;alg5;smo;ift88 |
| glycoprotein catabolic process; | 6#18 | 0.007545 | psenen;psen1;psen2;manba;tasp1;mgea5 |
| determination of bilateral symmetry; | 6#18 | 0.007545 | wnt3a;kif3b;pitx2;alg5;smo;ift88 |
| centrosome organization and biogenesis; | 6#18 | 0.007545 | brca2;cntrob;cetn1;bbs4;brca1;cetn3 |
| determination of left/right symmetry; | 6#18 | 0.007545 | wnt3a;kif3b;pitx2;alg5;smo;ift88 |
| response to carbohydrate stimulus; | 6#18 | 0.007545 | gck;lct;aldob;gys2;nr1h4;adipoq |
| microtubule organizing center organization and biogenesis; | 6#18 | 0.007545 | brca2;cntrob;cetn1;bbs4;brca1;cetn3 |
| fatty acid metabolic process; | 24#191 | 0.007595 | gnpat;acsl5;acot9;cpt1c;acaca;pla2g4b;cpt1b;pnpla8;acads;acaa2;aldh5a1;elovl7;baat;lypla2;pex13;tbxas1;acsl1;slc27a3;lipc;ltc4s;lypla1;adipoq;brca1;ptgis |
| sexual reproduction; | 35#315 | 0.007807 | chek1;izumo1;spag6;piwil2;ube2b;pafah1b2;spata2;serpina5;pick1;fut10;ccna1;wbp2nl;mov10l1;zscan2;cylc2;nanos3;mkks;txndc8;clgn;msh4;hexb;casc5;wdr33;adam21;zp4;piwil1;ereg;cct6b;tob2;sycp3;mfge8;brca2;spata18;gal3st1;fcgbp |
| gene expression#regulation of gene expression; | 290#3833 | 0.007951 | mcm6;zfp62;tead4;rab18;znf648;znf566;furin;arid1b;znf300;pick1;l3mbtl2;znf275;znf187;zfpm2;znf644;vps72;lhx3;taf5;tlr9;znf181;asf1a;nr2e1;notch1;rxrg;myod1;eif4ebp1;glis2;pstk;hoxc10;leo1;zbtb5;vgll3;rad54b;vsx1;mef2b;ncoa3;brca1;sox14;mael;pou5f2;myog;znf32;aff4;sp100;dmtf1;taf1b;nr1d1;pnrc2;nkx2-5;upf1;pfdn5;pou1f1;znf532;carhsp1;foxj3;supt3h;rdbp;edf1;nfyb;znf529;arntl2;bcl11b;sox9;pbx4;ccna2;nr4a1;etv2;rfc1;rps14;tcf15;irf2;ing4;foxp3;phb2;nr1h4;gtf2e1;phf17;ddx1;pqbp1;irf5;znf641;pax3;msc;znf280d;rpl6;hivep1;tcf23;thrsp;dbp;morf4l1;zscan2;zbtb32;suv420h1;mms19;smarcad1;sertad3;lzts1;znf667;znf584;znf175;ebi3;stat3;prdm15;sohlh2;tle4;znf629;brms1l;znf710;cbfa2t2;dpf3;arid4b;e2f6;mycbp2;mlxipl;smad5;sirt2;zkscan5;npas2;mycl1;ncoa1;ighmbp2;gbx1;hoxd8;hoxb13;znf576;jazf1;med18;nrbf2;pitx2;isl2;znf713;gtf2i;sox13;taf1a;hoxb4;dr1;med11;znf329;znf555;ssbp2;notch2;gtf3a;pum2;znf81;znf646;irak1;med6;znf672;zfp37;pogk;znf212;foxn1;znf524;znf35;yaf2;egln1;foxr1;chmp1a;ttf1;znf345;znf34;grhl3;tox3;znrd1;znf23;smarcb1;brd8;nr0b2;rab35;tcf25;noto;gata3;znf470;ifnk;rab8b;znf683;znf688;znf395;nr4a3;casp8ap2;pycard;znf142;zbtb3;znf624;smarcd2;gtf2h2;ercc3;znf250;gabpa;tceb1;rfx2;atxn3;uimc1;tbx10;sp2;znf202;znf16;pbrm1;znf569;pou2f3;pias4;tlr6;asb1;jdp2;eya2;gtf2h5;dmrtb1;znf770;hes5;ilf3;ehf;ereg;znf22;zmiz2;klf8;znf238;rbak;srf;nfyc;tfeb;pcgf5;wwp1;hivep2;meox1;nkx1-2;notch4;elavl2;tfdp2;polr2f;phf20;snapc4;aire;zkscan1;rbm15;hsf4;eif5a;sirt6;hoxc8;mef2d;l3mbtl3;erg;psip1;foxd4l1;foxn2;prrx2;pou5f1;nanos3;nkx2-6;barx1;ezh1;krt7;tigd2;efcab6;brca2;foxd2;zhx2;tbr1;terf2;elp4;ncoa6;htatip2;nfatc1;tlr3;paip2b;trim28;crx;nr1h3;suv39h1;pou2f2;rcbtb1;atf2;polr3g;med24;yeats4;scap;trmt6;bmp6;gzf1;smurf2;mdm2;eif4g3;smarce1;nab1;ccdc59;rere;zbtb7a;cry2;etv1;nod2;atf5 |
| locomotory behavior; | 23#181 | 0.008087 | oprd1;prkca;map2k1;cx3cr1;npas2;ppbp;cmtm8;ccl27;ltb4r2;hexb;mapk14;il16;als2;ccr4;ccl19;cxcl12;pex13;ptafr;cmtm7;ccr3;ccr9;cxcl1;cmtm4 |
| cellular polysaccharide biosynthetic process; | 9#40 | 0.008673 | gck;cercam;phkg2;gne;dyrk2;glt25d2;gys2;mat2b;phkg1 |
| polysaccharide biosynthetic process; | 9#40 | 0.008673 | gck;cercam;phkg2;gne;dyrk2;glt25d2;gys2;mat2b;phkg1 |
| regulation of immune system process; | 15#100 | 0.008752 | malt1;c4a;il27ra;cfi;masp1;ereg;traf6;ptprc;cfb;c8b;c1qc;bcar1;nod2;ifnk;foxp3 |
| hemopoiesis; | 21#161 | 0.009081 | nhej1;sh2b3;smad5;ncoa6;dyrk3;cd3d;notch4;wnt3a;sfxn1;tlr3;cd2;timp1;rab3d;notch2;nkx2-5;il17c;lep;ptprc;trim10;tpd52;fut10 |
| mRNA metabolic process; | 37#342 | 0.009712 | exosc10;slu7;ddx1;ppil3;snrpa;thoc3;cpsf1;pnpt1;sf3b3;upf1;ddx41;dhx38;hnrnpr;tsen2;prpf4b;hnrnpl;smg5;pabpc1;prpf40a;bard1;skiv2l2;txnl4b;srpk2;dhx35;zmat5;pcbp2;snrpd3;nudt21;elavl1;sf3a1;prpf3;rbm17;isy1;clp1;lsm10;zhx2;crnkl1 |
| reproduction; | 53#537 | 0.009712 | cyp11a1;wwp1;chek1;izumo1;spag6;piwil2;ube2b;thbd;lhb;pafah1b2;spata2;bmpr1a;serpina5;eif5a;wwp2;ide;pick1;pth;fut10;asb1;ccna1;mov10l1;wbp2nl;zscan2;smarcb1;cylc2;nanos3;mkks;txndc8;clgn;msh4;pla2g4b;sox9;hexb;dmrtb1;casc5;cdkl2;adam21;wdr33;zp4;lhcgr;piwil1;ereg;vapb;cct6b;cav2;tob2;sycp3;mfge8;brca2;spata18;gal3st1;fcgbp |
| regulation of JNK cascade; | 6#19 | 0.00993 | tlr3;taok2;mapk8ip2;taok3;mapk8ip1;mapk8ip3 |
| response to ionizing radiation; | 5#13 | 0.009944 | abcg5;nhej1;brca2;atm;thbd |
| protein homooligomerization; | 9#41 | 0.010142 | smarcad1;cda;scube1;atpif1;gopc;vwf;aldh5a1;adipoq;actn2 |
| response to hormone stimulus; | 13#76 | 0.010142 | cyp11a1;map2k1;mms19;ncoa6;cav2;gata3;lct;acads;brca2;mat2a;aldob;lcat;brca1 |
| adaptive immune response; | 13#76 | 0.010142 | malt1;il27ra;il18bp;cfi;masp1;traf6;ptprc;c8b;c1qc;tlr6;ly9;foxp3;ebi3 |
| adaptive immune response based on somatic recombination of immune receptors built from immunoglobulin superfamily domains; | 13#76 | 0.010142 | malt1;il27ra;il18bp;cfi;masp1;traf6;ptprc;c8b;c1qc;tlr6;ly9;foxp3;ebi3 |
| hemopoietic or lymphoid organ development; | 22#173 | 0.010142 | nhej1;sh2b3;smad5;ncoa6;dyrk3;cd3d;notch4;wnt3a;sfxn1;tlr3;cd2;timp1;rab3d;tnfrsf11a;notch2;nkx2-5;il17c;lep;ptprc;trim10;tpd52;fut10 |
| secretion by cell; | 33#297 | 0.010714 | nmur1;gopc;rab3d;dph3;sar1a;trappc1;snca;cplx1;stx16;pick1;kcnma1;stx1a;ppy;stx18;stxbp1;lat;canx;sec61a1;sec24a;tmed10;unc13b;napg;napa;pycard;rims4;sec31a;snap29;nod2;cplx3;cog2;gosr1;foxp3;osbpl5 |
| lipid biosynthetic process; | 36#333 | 0.011244 | pigq;a4galt;hsd17b2;cyp11a1;acaca;chpt1;pigs;pigb;fabp3;hsd17b11;elovl7;pisd;gne;ugcg;gpaa1;tbxas1;fdft1;scap;hsd17b12;bmp6;ptgis;pik3c2a;idi1;sgms2;agpat4;hexb;acaa2;glt25d2;stard5;pigz;hmgcs1;impa1;cercam;ltc4s;gal3st1;brca1 |
| multi-organism process; | 36#333 | 0.011244 | cyp11a1;wwp1;edn1;slc11a1;tlr3;twf2;thbd;duox2;cxcl12;wwp2;bpi;pex13;cldn4;tlr6;defb124;mkks;pglyrp1;alb;ppbp;pla2g4b;hexb;tlr9;ifnk;znf175;nod1;cst11;ccl19;il27ra;avpr1a;defb116;vapb;ptprc;ccdc130;krt8;nod2;tbk1 |
| biopolymer catabolic process; | 46#454 | 0.01135 | exosc10;gaa;usp29;usp26;ercc3;usp35;usp7;usp30;usp46;ppt1;psmc2;usp10;ube2b;pnpt1;tceb1;ube2d3;cecr2;upf1;smg5;psma6;cdc23;psmb4;usp2;dnase1l2;exosc7;bard1;psmf1;rnase6;smurf2;fbxo8;mdm2;psmb6;elavl1;mmp19;arih1;adamts14;usp48;usp45;use1;mmp11;mmp7;pop1;usp49;usp53;zhx2;fbxl7 |
| positive regulation of signal transduction; | 20#153 | 0.01135 | malt1;faslg;fadd;taok2;traf6;taok3;slc20a1;tlr3;zdhhc17;nod1;casp8;ereg;ect2;ecm1;nod2;tlr6;ripk1;adipoq;trim13;tbk1 |
| regulation of nucleobase, nucleoside, nucleotide and nucleic acid metabolic process; | 280#3713 | 0.011501 | mcm6;zfp62;tead4;rab18;znf648;znf566;arid1b;znf300;l3mbtl2;znf275;znf187;zfpm2;znf644;vps72;lhx3;taf5;znf181;asf1a;nr2e1;notch1;rxrg;myod1;glis2;hoxc10;leo1;zbtb5;vgll3;rad54b;vsx1;mef2b;ncoa3;brca1;sox14;mael;edn1;pou5f2;myog;znf32;aff4;sp100;dmtf1;taf1b;nr1d1;pnrc2;nkx2-5;pfdn5;pou1f1;znf532;carhsp1;pdzd3;foxj3;supt3h;rdbp;edf1;nfyb;znf529;arntl2;bcl11b;sox9;pbx4;ccna2;nr4a1;etv2;rps14;rfc1;tcf15;irf2;ing4;foxp3;phb2;nr1h4;gtf2e1;phf17;pqbp1;irf5;znf641;pax3;msc;znf280d;rpl6;hivep1;tcf23;thrsp;dbp;morf4l1;zscan2;zbtb32;suv420h1;mms19;smarcad1;sertad3;lzts1;znf667;znf584;znf175;stat3;prdm15;sohlh2;tle4;znf629;brms1l;znf710;cbfa2t2;dpf3;arid4b;e2f6;mycbp2;mlxipl;smad5;sirt2;zkscan5;npas2;mycl1;ncoa1;ighmbp2;gbx1;hoxd8;hoxb13;znf576;jazf1;med18;nrbf2;pitx2;isl2;znf713;gtf2i;pabpc1;sox13;taf1a;hoxb4;dr1;med11;znf329;znf555;ssbp2;notch2;gtf3a;znf81;znf646;irak1;med6;znf672;zfp37;pogk;znf212;foxn1;znf524;znf35;yaf2;egln1;foxr1;chmp1a;ttf1;znf345;znf34;grhl3;tox3;znrd1;znf23;brd8;nr0b2;smarcb1;rab35;tcf25;noto;gata3;znf470;ifnk;rab8b;znf683;znf688;znf395;nr4a3;casp8ap2;pycard;znf142;zbtb3;znf624;smarcd2;gtf2h2;ercc3;znf250;gabpa;tceb1;rfx2;atxn3;uimc1;tbx10;sp2;znf202;znf16;pbrm1;atpif1;znf569;pou2f3;pias4;jdp2;eya2;cda;gtf2h5;dmrtb1;znf770;hes5;ilf3;ehf;ereg;znf22;zmiz2;klf8;znf238;rbak;srf;nfyc;tfeb;pcgf5;wwp1;hivep2;meox1;nkx1-2;notch4;elavl2;tfdp2;polr2f;phf20;snapc4;aire;zkscan1;rbm15;hsf4;sirt6;hoxc8;mef2d;l3mbtl3;erg;psip1;foxd4l1;foxn2;prrx2;pou5f1;nkx2-6;barx1;ezh1;tigd2;efcab6;brca2;foxd2;zhx2;tbr1;terf2;elp4;ncoa6;htatip2;nfatc1;tlr3;trim28;crx;nr1h3;suv39h1;pou2f2;rcbtb1;atf2;polr3g;med24;yeats4;bard1;scap;bmp6;gzf1;smurf2;mdm2;smarce1;nab1;ccdc59;elavl1;rere;zbtb7a;cry2;etv1;nod2;atf5 |
| cytokinesis; | 8#34 | 0.011501 | stx2;sept7;sept3;cntrob;rab35;aurkb;brca2;cecr2 |
| sphingolipid metabolic process; | 12#68 | 0.011501 | a4galt;sgpp1;sptlc1;ugcg;sftpb;sgms2;hexb;ppt1;sphk1;gal3st1;aldh5a1;smpd1 |
| chromatin modification; | 26#219 | 0.012078 | morf4l1;suv420h1;sirt2;vps72;smarcb1;brd8;cpa4;map3k12;smarcad1;taf5;smarce1;prmt7;cecr2;asf1a;suv39h1;arid1b;sirt6;rcbtb1;pbrm1;sycp3;yeats4;l3mbtl2;ttf1;foxp3;setd7;smarcd2 |
| nucleocytoplasmic transport; | 20#154 | 0.012431 | malt1;aaas;htatip2;nfkbie;rpl23;ipo8;anp32a;camk1;nup98;fyb;upf1;mbtps1;rere;gckr;cry2;smg5;nup160;pola2;bard1;gsk3b |
| cellular metal ion homeostasis; | 17#123 | 0.012506 | prkca;edn1;f2rl1;pln;gna15;hexb;ccr4;ccl19;cxcl12;avpr1a;tpt1;ptprc;tnni3;pth;ccr3;ccr9;ednra |
| metal ion homeostasis; | 17#123 | 0.012506 | prkca;edn1;f2rl1;pln;gna15;hexb;ccr4;ccl19;cxcl12;avpr1a;tpt1;ptprc;tnni3;pth;ccr3;ccr9;ednra |
| cytokine production; | 16#113 | 0.012547 | malt1;traf6;gata3;tlr9;tlr3;nod1;ebi3;il27ra;ereg;pycard;il19;ptafr;nod2;tlr6;foxp3;asb1 |
| membrane lipid biosynthetic process; | 16#113 | 0.012547 | pigq;pik3c2a;a4galt;chpt1;pigs;agpat4;sgms2;pigb;hexb;fabp3;pisd;ugcg;gpaa1;pigz;impa1;gal3st1 |
| response to abiotic stimulus; | 21#165 | 0.012666 | cdkn1a;nhej1;gtf2h2;mkks;adrb1;ercc3;abcg5;tlr3;mat2a;thbd;nphp4;rho;avpr1a;atpif1;crnn;fen1;brca2;trpv2;atm;rgr;mc1r |
| cellular polysaccharide metabolic process; | 13#84 | 0.012893 | gck;ppp1r2;gaa;phkg2;gne;gal3st3;dyrk2;cercam;glt25d2;gys2;mat2b;gsk3b;phkg1 |
| centrosome cycle; | 5#14 | 0.013363 | brca2;cntrob;cetn1;brca1;cetn3 |
| vasculature development; | 21#166 | 0.013896 | adora2b;canx;cspg4;htatip2;edn1;pdpn;sh2d2a;notch4;angpt1;sphk1;mmp19;angptl3;nkx2-5;ccm2;fgf1;c1galt1;ereg;smo;tnni3;atpif1;nppb |
| positive regulation of apoptosis; | 29#256 | 0.014835 | tia1;cdkn1a;faslg;prkca;fadd;htatip2;ercc3;cd2;siva1;unc13b;sfn;trim35;notch2;dyrk2;ppp1r13b;casp8ap2;ptprc;rps3a;pycard;cidec;bcl2l13;il19;pth;bik;brca2;dapk2;cideb;bard1;brca1 |
| gene expression; | 396#5454 | 0.015399 | exosc10;mcm6;zfp62;tead4;ppil3;snrpa;rab18;znf648;ears2;trim29;znf566;psen1;furin;arid1b;farsb;rpl28;znf300;pick1;l3mbtl2;znf275;larp7;znf187;zfpm2;calcoco1;rps9;znf644;vps72;rbms2;lhx3;rars;taf5;tlr9;znf181;rpl12;asf1a;nr2e1;notch1;rxrg;pop5;myod1;eif4ebp1;glis2;pstk;hoxc10;leo1;zbtb5;vgll3;rad54b;vsx1;mef2b;ncoa3;hars2;brca1;sox14;mael;pou5f2;myog;znf32;psenen;aff4;sp100;dmtf1;taf1b;nr1d1;pnrc2;nkx2-5;upf1;pfdn5;ecd;rpl8;pou1f1;znf532;carhsp1;foxj3;txnl4b;supt3h;rdbp;dhx35;edf1;nfyb;znf529;cherp;arntl2;bcl11b;sox9;pbx4;mrpl24;mars;ccna2;nr4a1;etv2;rfc1;rps14;tcf15;rps3a;irf2;ing4;foxp3;phb2;nr1h4;gtf2e1;rpl18a;phf17;cdc73;ddx1;mrpl43;thoc3;rrbp1;pqbp1;irf5;znf641;pax3;msc;znf280d;rpl6;hivep1;tcf23;thrsp;dbp;morf4l1;zscan2;zbtb32;suv420h1;mms19;sertad3;smarcad1;lzts1;znf667;rpl39;znf584;nudt21;znf175;ftsj2;sf3a1;ebi3;stat3;rps5;prpf3;prdm15;dalrd3;sohlh2;tle4;znf629;brms1l;znf710;cbfa2t2;dpf3;arid4b;e2f6;mycbp2;mlxipl;smad5;sirt2;zkscan5;npas2;mycl1;ncoa1;ighmbp2;rpp38;gbx1;hoxd8;hoxb13;znf576;jazf1;sf3b3;med18;mrpl17;nrbf2;pitx2;mrps5;isl2;znf713;gtf2i;pabpc1;prpf40a;sox13;taf1a;hoxb4;dr1;mrpl49;znf329;med11;znf555;mrpl9;ssbp2;tsr2;notch2;gtf3a;pum2;trip11;utp18;znf81;fam83d;rbm17;znf646;trmu;irak1;med6;pop1;mrpl11;znf672;rpl5;lars;mrpl30;zfp37;znf212;pogk;foxn1;znf524;qars;znf35;yaf2;egln1;foxr1;chmp1a;ttf1;znf345;znf34;grhl3;tox3;znrd1;znf23;smarcb1;brd8;nr0b2;aarsd1;rab35;tcf25;gata3;noto;sart3;znf470;ifnk;rab8b;znf683;znf688;znf395;nr4a3;casp8ap2;pycard;znf142;znf624;zbtb3;smarcd2;crnkl1;rps6kb2;gtf2h2;rpl23;ercc3;znf250;cpsf1;gabpa;rfx2;tceb1;atxn3;uimc1;tbx10;sp2;tsen2;znf202;prpf4b;znf16;pbrm1;hnrnpl;znf569;pou2f3;eif4a1;pias4;tlr6;skiv2l2;asb1;srpk2;jdp2;rpap1;eya2;psen2;gtf2h5;rps15a;dmrtb1;znf770;hes5;ilf3;ehf;ereg;znf22;zmiz2;klf8;clp1;znf238;rbak;srf;slu7;nfyc;tfeb;pcgf5;wwp1;dis3;hivep2;rpl7l1;meox1;nkx1-2;notch4;elavl2;abtb1;eif2c3;tfdp2;polr2f;pnpt1;phf20;pars2;snapc4;aire;zkscan1;rbm15;hsf4;eif5a;sirt6;rpp14;l3mbtl3;hoxc8;mef2d;papolb;erg;psip1;foxd4l1;foxn2;prrx2;exosc7;pou5f1;nanos3;zmat5;nkx2-6;rplp1;barx1;parp1;qtrtd1;ezh1;rps23;krt7;isy1;tigd2;efcab6;brca2;foxd2;zhx2;tbr1;terf2;elp4;eef1a2;ncoa6;htatip2;mto1;exosc8;nfatc1;tlr3;paip2b;trim28;crx;ddx41;dhx38;nr1h3;pou2f2;suv39h1;hnrnpr;rcbtb1;atf2;polr3g;med24;yeats4;polr3d;bard1;scap;trmt6;bmp6;gzf1;dnajc13;smurf2;rps27;mdm2;eif4g3;smarce1;nab1;ccdc59;snrpd3;ppig;rere;zbtb7a;dhps;mtfmt;cry2;mrps14;nars;atf5;nod2;lsm10;etv1 |
| macromolecule catabolic process; | 59#625 | 0.015399 | exosc10;gaa;usp29;usp26;ercc3;usp35;usp7;usp30;usp46;ppt1;psmc2;usp10;ube2b;pnpt1;aldob;tceb1;ube2d3;cecr2;gck;upf1;ecd;smg5;psma6;cdc23;psmb4;usp2;gba3;dnase1l2;fut10;exosc7;bard1;psmf1;rnase6;smurf2;fbxo8;pglyrp1;mdm2;pfkp;lyg2;psmb6;ldha;mmp19;elavl1;arih1;adamts14;usp48;hibadh;hk1;usp45;use1;mmp11;mmp7;pop1;usp49;uevld;usp53;zhx2;fbxl7;pgk2 |
| protein homotetramerization; | 4#9 | 0.016421 | cda;atpif1;aldh5a1;actn2 |
| response to organic substance; | 11#62 | 0.016552 | gck;cyp11a1;lct;brca2;mat2a;thbd;aldob;gys2;duox2;adipoq;nr1h4 |
| positive regulation of programmed cell death; | 29#258 | 0.016971 | tia1;cdkn1a;faslg;prkca;fadd;htatip2;ercc3;cd2;siva1;unc13b;sfn;trim35;notch2;dyrk2;ppp1r13b;casp8ap2;ptprc;rps3a;pycard;cidec;bcl2l13;il19;pth;bik;brca2;dapk2;cideb;bard1;brca1 |
| polysaccharide metabolic process; | 13#86 | 0.017224 | gck;ppp1r2;gaa;phkg2;gne;gal3st3;dyrk2;cercam;glt25d2;gys2;mat2b;gsk3b;phkg1 |
| positive regulation of transferase activity; | 19#147 | 0.017325 | chrm1;adora2b;malt1;taok2;vav2;edn1;traf6;tlr3;sphk1;dgki;als2;serinc2;dgkg;ereg;gap43;ptprc;irak1;pick1;tlr6 |
| response to stimulus; | 267#3553 | 0.017872 | trex1;slc24a5;ly96;il18bp;mertk;cfb;myo3a;sod3;cd96;stk25;ccr4;il1rl2;mst1;c1qc;adamts13;trpv2;oprd1;mdc1;tlr9;wdr33;asf1a;notch1;clec1a;c4a;poln;il27ra;cdh23;masp1;cav2;rad54b;krt8;vsx1;ccdc130;ccr3;ly9;brca1;ung;rfc3;edn1;fmo4;thbd;bbs10;upf1;apc;dnaja1;skap1;bcar1;ptafr;pdzd3;defb124;socs6;rtp3;arntl2;snx27;hexb;pag1;ccna2;rho;ppef2;scn11a;defb116;recql;bcar3;gcgr;crnn;foxp3;nr1h4;gsk3b;adora2b;phf17;rpgrip1;rbp4;cd69;traf6;il15;vwf;gck;sstr2;bpi;snca;tbxas1;prph2;lcat;mc1r;prkca;mms19;itgal;pglyrp1;cd97;ppbp;lct;c8b;mefv;kiss1r;il16;gadd45a;znf175;ebi3;stat3;avil;hspb9;prpf3;il18r1;fancb;p2ry1;sirt2;npas2;f2rl1;fanca;ighmbp2;mapk14;mmrn1;atrn;hoxb13;atrip;als2;chl1;duox2;pdcd1;pex13;il19;ndrg4;mus81;tlr5;fut10;adipoq;malt1;darc;ndufs8;pla2g4b;nphp4;nyx;msh2;ccr9;tbk1;s100a8;ms4a2;ccl27;foxn1;scnn1a;gcg;gys2;pdzk1;cxcl12;ly6e;egln1;chmp1a;cd1b;cxcl1;rad52;map2k1;taok2;rif1;adrb1;fth1;gata3;cmtm8;ifnk;slc25a13;hp;slc25a12;il17c;cd6;vapb;bbs4;fen1;samhd1;cideb;adcy3;gtf2h2;cyp11a1;cfi;ercc3;slc11a1;irak2;proc;kcnj11;parp4;twf2;aldob;ube2b;atxn3;sp2;mmp25;rgs1;dyrk2;gap43;atpif1;tlr6;il18rap;lat;dnajb5;gtf2h5;rfc5;nod1;cst11;ccl19;avpr1a;ereg;ik;msh5;rgr;faslg;shprh;cx3cr1;scube1;ltb4r2;poll;oasl;rad21;abca3;aire;hsf4;rtp4;lep;nudt1;f2rl3;rnase6;mkks;esco2;cd19;parp1;slc18a1;aoc3;best1;ptprc;hspd1;brca2;clec4e;nhej1;ncf1;ncoa6;chek1;abcg5;tlr3;ppt1;pcdh15;sfn;tnfrsf11a;fyb;crx;pou2f2;sgk2;cmtm7;bard1;bmp6;cmtm4;scap;fgf7;cdkn1a;mlh3;klrc2;mfrp;alb;msh4;acads;mat2a;kcnip1;nqo1;cd164;crisp3;cry2;fgb;nod2;atm;tnfrsf4;ush1c |
| RNA splicing; | 26#225 | 0.018673 | srpk2;slu7;dhx35;ddx1;zmat5;ppil3;snrpa;thoc3;snrpd3;ppig;sf3b3;sf3a1;ddx41;dhx38;hnrnpr;tsen2;prpf4b;rbm17;prpf3;isy1;pabpc1;lsm10;prpf40a;skiv2l2;txnl4b;crnkl1 |
| transcription, DNA-dependent; | 259#3439 | 0.018711 | mcm6;zfp62;tead4;rab18;znf648;trim29;znf566;arid1b;znf300;l3mbtl2;znf275;znf187;zfpm2;znf644;vps72;lhx3;taf5;znf181;asf1a;nr2e1;notch1;rxrg;myod1;glis2;hoxc10;leo1;zbtb5;rad54b;vsx1;mef2b;ncoa3;brca1;sox14;mael;pou5f2;myog;znf32;aff4;sp100;dmtf1;taf1b;nr1d1;pnrc2;nkx2-5;pfdn5;ecd;pou1f1;znf532;carhsp1;foxj3;supt3h;rdbp;edf1;nfyb;znf529;arntl2;bcl11b;sox9;pbx4;nr4a1;etv2;rps14;rfc1;tcf15;irf2;ing4;foxp3;phb2;nr1h4;gtf2e1;phf17;pqbp1;irf5;znf641;pax3;msc;znf280d;rpl6;hivep1;dbp;thrsp;morf4l1;zscan2;zbtb32;suv420h1;mms19;sertad3;lzts1;znf667;znf584;znf175;stat3;prdm15;tle4;znf629;brms1l;znf710;cbfa2t2;dpf3;arid4b;e2f6;mycbp2;mlxipl;smad5;sirt2;zkscan5;npas2;mycl1;ncoa1;ighmbp2;gbx1;hoxd8;hoxb13;znf576;jazf1;med18;nrbf2;pitx2;isl2;znf713;gtf2i;sox13;taf1a;hoxb4;dr1;med11;znf329;znf555;notch2;gtf3a;trip11;znf81;znf646;med6;znf672;zfp37;pogk;znf212;foxn1;znf524;znf35;yaf2;foxr1;chmp1a;ttf1;znf345;znf34;grhl3;tox3;znf23;brd8;nr0b2;smarcb1;rab35;tcf25;noto;gata3;znf470;rab8b;znf683;znf688;znf395;nr4a3;casp8ap2;znf142;zbtb3;znf624;smarcd2;gtf2h2;ercc3;znf250;gabpa;tceb1;rfx2;atxn3;uimc1;tbx10;sp2;znf202;znf16;pbrm1;znf569;pou2f3;pias4;jdp2;eya2;gtf2h5;dmrtb1;znf770;hes5;ilf3;ehf;ereg;znf22;zmiz2;klf8;znf238;rbak;srf;nfyc;tfeb;pcgf5;hivep2;nkx1-2;meox1;elavl2;notch4;tfdp2;polr2f;phf20;snapc4;aire;zkscan1;hsf4;sirt6;hoxc8;mef2d;erg;psip1;foxd4l1;foxn2;prrx2;pou5f1;nkx2-6;barx1;parp1;ezh1;efcab6;brca2;foxd2;zhx2;tbr1;elp4;ncoa6;htatip2;nfatc1;trim28;crx;nr1h3;suv39h1;pou2f2;rcbtb1;atf2;polr3g;med24;yeats4;polr3d;bmp6;scap;gzf1;mdm2;smarce1;ccdc59;nab1;rere;zbtb7a;cry2;etv1;atf5 |
| membrane fusion; | 8#37 | 0.018885 | stx2;serpina5;napa;atg7;izumo1;snap29;napg;stx11 |
| G-protein signaling, coupled to cyclic nucleotide second messenger; | 16#117 | 0.019763 | rgs1;sstr2;adora2b;oprd1;lhcgr;adrb1;mtnr1b;gcgr;pth;ltb4r2;mc3r;prkacb;ptgir;ccr3;ednra;mc1r |
| oligosaccharide metabolic process; | 6#22 | 0.019763 | mgat2;hexb;gal3st3;st6gal2;cog2;mpdu1 |
| response to estrogen stimulus; | 6#22 | 0.019763 | gata3;lct;cyp11a1;brca2;cav2;brca1 |
| RNA biosynthetic process; | 259#3444 | 0.019999 | mcm6;zfp62;tead4;rab18;znf648;trim29;znf566;arid1b;znf300;l3mbtl2;znf275;znf187;zfpm2;znf644;vps72;lhx3;taf5;znf181;asf1a;nr2e1;notch1;rxrg;myod1;glis2;hoxc10;leo1;zbtb5;rad54b;vsx1;mef2b;ncoa3;brca1;sox14;mael;pou5f2;myog;znf32;aff4;sp100;dmtf1;taf1b;nr1d1;pnrc2;nkx2-5;pfdn5;ecd;pou1f1;znf532;carhsp1;foxj3;supt3h;rdbp;edf1;nfyb;znf529;arntl2;bcl11b;sox9;pbx4;nr4a1;etv2;rps14;rfc1;tcf15;irf2;ing4;foxp3;phb2;nr1h4;gtf2e1;phf17;pqbp1;irf5;znf641;pax3;msc;znf280d;rpl6;hivep1;dbp;thrsp;morf4l1;zscan2;zbtb32;suv420h1;mms19;sertad3;lzts1;znf667;znf584;znf175;stat3;prdm15;tle4;znf629;brms1l;znf710;cbfa2t2;dpf3;arid4b;e2f6;mycbp2;mlxipl;smad5;sirt2;zkscan5;npas2;mycl1;ncoa1;ighmbp2;gbx1;hoxd8;hoxb13;znf576;jazf1;med18;nrbf2;pitx2;isl2;znf713;gtf2i;sox13;taf1a;hoxb4;dr1;med11;znf329;znf555;notch2;gtf3a;trip11;znf81;znf646;med6;znf672;zfp37;pogk;znf212;foxn1;znf524;znf35;yaf2;foxr1;chmp1a;ttf1;znf345;znf34;grhl3;tox3;znf23;brd8;nr0b2;smarcb1;rab35;tcf25;noto;gata3;znf470;rab8b;znf683;znf688;znf395;nr4a3;casp8ap2;znf142;zbtb3;znf624;smarcd2;gtf2h2;ercc3;znf250;gabpa;tceb1;rfx2;atxn3;uimc1;tbx10;sp2;znf202;znf16;pbrm1;znf569;pou2f3;pias4;jdp2;eya2;gtf2h5;dmrtb1;znf770;hes5;ilf3;ehf;ereg;znf22;zmiz2;klf8;znf238;rbak;srf;nfyc;tfeb;pcgf5;hivep2;nkx1-2;meox1;elavl2;notch4;tfdp2;polr2f;phf20;snapc4;aire;zkscan1;hsf4;sirt6;hoxc8;mef2d;erg;psip1;foxd4l1;foxn2;prrx2;pou5f1;nkx2-6;barx1;parp1;ezh1;efcab6;brca2;foxd2;zhx2;tbr1;elp4;ncoa6;htatip2;nfatc1;trim28;crx;nr1h3;suv39h1;pou2f2;rcbtb1;atf2;polr3g;med24;yeats4;polr3d;bmp6;scap;gzf1;mdm2;smarce1;ccdc59;nab1;rere;zbtb7a;cry2;etv1;atf5 |
| phospholipid metabolic process; | 25#215 | 0.020338 | pigq;pik3c2a;flrt2;chpt1;pigs;agpat4;sgms2;pla2g4b;pla2g12a;pigb;hexb;pitpnm3;fabp3;plcb3;aldh5a1;serinc2;pla2g12b;pisd;snca;gpaa1;pigz;impa1;lipc;proca1;smpd1 |
| glycerophospholipid biosynthetic process; | 9#46 | 0.020397 | pigq;pik3c2a;gpaa1;chpt1;pigs;pigz;pigb;impa1;fabp3 |
| RNA catabolic process; | 9#46 | 0.020397 | upf1;exosc10;rnase6;smg5;pop1;elavl1;pnpt1;zhx2;exosc7 |
| mRNA processing; | 30#273 | 0.020624 | srpk2;slu7;dhx35;ddx1;zmat5;ppil3;snrpa;thoc3;cpsf1;snrpd3;nudt21;sf3b3;sf3a1;ddx41;dhx38;hnrnpr;tsen2;prpf4b;rbm17;prpf3;isy1;hnrnpl;clp1;pabpc1;prpf40a;lsm10;bard1;skiv2l2;txnl4b;crnkl1 |
| energy reserve metabolic process; | 10#55 | 0.020765 | gck;ppp1r2;gaa;phkg2;dyrk2;lep;gfpt2;gys2;gsk3b;phkg1 |
| regulation of transcription; | 273#3654 | 0.021127 | mcm6;zfp62;tead4;rab18;znf648;znf566;arid1b;znf300;l3mbtl2;znf275;znf187;zfpm2;znf644;vps72;lhx3;taf5;znf181;asf1a;nr2e1;notch1;rxrg;myod1;glis2;hoxc10;leo1;zbtb5;vgll3;rad54b;vsx1;mef2b;ncoa3;brca1;sox14;mael;pou5f2;myog;znf32;aff4;sp100;dmtf1;taf1b;nr1d1;pnrc2;nkx2-5;pfdn5;pou1f1;znf532;carhsp1;foxj3;supt3h;rdbp;edf1;nfyb;znf529;arntl2;bcl11b;sox9;pbx4;ccna2;nr4a1;etv2;rps14;rfc1;tcf15;irf2;ing4;foxp3;phb2;nr1h4;gtf2e1;phf17;pqbp1;irf5;znf641;pax3;msc;znf280d;rpl6;hivep1;tcf23;dbp;thrsp;morf4l1;zscan2;zbtb32;suv420h1;mms19;smarcad1;sertad3;lzts1;znf667;znf584;znf175;stat3;prdm15;sohlh2;tle4;znf629;brms1l;znf710;cbfa2t2;dpf3;arid4b;e2f6;mycbp2;mlxipl;smad5;sirt2;zkscan5;npas2;mycl1;ncoa1;ighmbp2;gbx1;hoxd8;hoxb13;znf576;jazf1;med18;nrbf2;pitx2;isl2;znf713;gtf2i;sox13;taf1a;hoxb4;dr1;med11;znf329;znf555;ssbp2;notch2;gtf3a;znf81;znf646;irak1;med6;znf672;zfp37;pogk;znf212;foxn1;znf524;znf35;yaf2;egln1;foxr1;chmp1a;ttf1;znf345;znf34;grhl3;tox3;znrd1;znf23;brd8;nr0b2;smarcb1;rab35;tcf25;noto;gata3;znf470;ifnk;rab8b;znf683;znf688;znf395;nr4a3;casp8ap2;pycard;znf142;zbtb3;znf624;smarcd2;gtf2h2;ercc3;znf250;gabpa;tceb1;rfx2;atxn3;uimc1;tbx10;sp2;znf202;znf16;pbrm1;znf569;pou2f3;pias4;jdp2;eya2;gtf2h5;dmrtb1;znf770;hes5;ilf3;ehf;ereg;znf22;zmiz2;klf8;znf238;rbak;srf;nfyc;tfeb;pcgf5;wwp1;hivep2;meox1;nkx1-2;elavl2;notch4;tfdp2;polr2f;phf20;snapc4;aire;zkscan1;rbm15;hsf4;sirt6;hoxc8;mef2d;l3mbtl3;erg;psip1;foxd4l1;foxn2;prrx2;pou5f1;nkx2-6;barx1;ezh1;tigd2;efcab6;brca2;foxd2;zhx2;tbr1;terf2;elp4;ncoa6;htatip2;nfatc1;tlr3;trim28;crx;nr1h3;suv39h1;pou2f2;rcbtb1;atf2;polr3g;med24;yeats4;bmp6;scap;gzf1;smurf2;mdm2;smarce1;ccdc59;nab1;rere;zbtb7a;cry2;etv1;nod2;atf5 |
| cytoskeleton organization and biogenesis; | 63#686 | 0.021208 | fgd5;mapre1;sdcbp;nck1;myh4;kif1a;kiss1;ube2b;ssh1;cecr2;ssh2;tubb3;kif27;cxcl12;apc;kif3b;clasp1;katnb1;ndc80;cntrob;coro2b;pex13;epb41l2;cks2;arpc3;kif26b;bcar1;ttn;cxcl1;kif5a;limch1;trip10;kif18a;taok2;arfip2;tmed10;nphp4;epb41;tubgcp6;kif23;map2;krt7;bub1b;avil;dnhd1;dynlrb2;tubg1;cav2;capg;prdm15;dlg1;krt8;cenpj;snap29;krt4;sptbn2;ush1c;dbn1;dst;kif13b;nedd9;shroom1;brca1 |
| rhythmic process; | 12#74 | 0.02142 | aanat;crx;cry2;npas2;ereg;hebp1;msh4;arntl2;slc9a3;mat2a;nr1d1;dbp |
| keratinocyte differentiation; | 7#30 | 0.021678 | evpl;cdsn;foxn1;map2k1;dsg4;ereg;sfn |
| T cell cytokine production; | 3#5 | 0.022312 | malt1;traf6;foxp3 |
| regulation of T cell cytokine production; | 3#5 | 0.022312 | malt1;traf6;foxp3 |
| blood vessel development#blood vessel morphogenesis#angiogenesis; | 17#129 | 0.022792 | adora2b;canx;cspg4;htatip2;edn1;sh2d2a;notch4;angpt1;sphk1;mmp19;angptl3;fgf1;c1galt1;ereg;tnni3;atpif1;nppb |
| signal complex assembly; | 5#16 | 0.023306 | pick1;ptk2;pxn;nck1;mapk8ip2 |
| neurotransmitter biosynthetic process; | 5#16 | 0.023306 | gata3;tph1;gad2;chat;snca |
| response to other organism; | 24#206 | 0.023324 | pglyrp1;slc11a1;ppbp;tlr9;tlr3;twf2;ifnk;thbd;znf175;duox2;nod1;ccl19;cst11;cxcl12;il27ra;defb116;bpi;ptprc;ccdc130;krt8;nod2;defb124;tlr6;tbk1 |
| regulation of nucleotide metabolic process; | 4#10 | 0.023324 | cda;atpif1;pdzd3;edn1 |
| T cell mediated immunity; | 4#10 | 0.023324 | malt1;traf6;foxp3;ptprc |
| amino acid and derivative metabolic process; | 52#548 | 0.024324 | psat1;sardh;lars;ears2;qars;aldh5a1;pars2;sult1b1;gldc;slco4a1;dio1;il22ra2;tph1;baat;ftcd;farsb;snca;arg2;qdpr;mthfd1;mat2b;dnajc13;prkca;afmid;hmgcl;sds;aarsd1;hal;gata3;rars;mars;mat2a;thnsl1;dmgdh;got1;pdpr;ddc;mccc1;dhps;gad2;fam83d;hibadh;dalrd3;nars;tmlhe;dtd1;gfpt2;gmps;bckdhb;hars2;padi6;slc3a1 |
| regulation of protein metabolic process; | 42#422 | 0.024428 | nck1;ddx1;tlr3;timp1;ube2b;ube2d3;paip2b;cxcl12;upf1;apc;furin;clasp1;serpina5;katnb1;eif5a;il22ra2;arpc3;tlr6;bard1;scap;trmt6;asb1;prkca;nanos3;mdm2;eif4g3;gata3;tlr9;angptl3;ebi3;pum2;eif4ebp1;krt7;map2;pstk;avil;ereg;capg;uevld;sptbn2;foxp3;ift88 |
| mitochondrion organization and biogenesis; | 13#89 | 0.024428 | ucp3;slc25a37;slc25a36;slc25a6;slc25a1;tomm34;cav2;ndufs8;tomm40;timm50;hspd1;fxc1;sfn |
| regulation of transcription, DNA-dependent; | 252#3358 | 0.024436 | mcm6;zfp62;zfp37;tead4;znf212;pogk;rab18;foxn1;znf648;znf524;znf35;znf566;arid1b;yaf2;foxr1;znf300;chmp1a;l3mbtl2;ttf1;znf275;znf345;znf34;znf187;grhl3;zfpm2;tox3;znf23;znf644;vps72;smarcb1;nr0b2;brd8;rab35;tcf25;lhx3;gata3;noto;taf5;znf181;znf470;rab8b;asf1a;znf683;nr2e1;notch1;znf688;rxrg;myod1;znf395;glis2;casp8ap2;hoxc10;nr4a3;leo1;zbtb5;rad54b;vsx1;mef2b;znf142;ncoa3;znf624;zbtb3;smarcd2;brca1;sox14;gtf2h2;mael;pou5f2;ercc3;myog;znf32;znf250;aff4;sp100;gabpa;dmtf1;rfx2;tceb1;atxn3;taf1b;tbx10;uimc1;nr1d1;sp2;pnrc2;nkx2-5;pfdn5;znf202;znf16;pbrm1;znf569;pou1f1;pou2f3;znf532;carhsp1;foxj3;pias4;supt3h;rdbp;jdp2;edf1;eya2;nfyb;znf529;arntl2;bcl11b;sox9;gtf2h5;pbx4;dmrtb1;znf770;nr4a1;etv2;hes5;rps14;rfc1;ilf3;ehf;tcf15;znf22;zmiz2;irf2;klf8;ing4;znf238;rbak;foxp3;phb2;nr1h4;gtf2e1;srf;phf17;nfyc;tfeb;pcgf5;hivep2;meox1;nkx1-2;notch4;elavl2;pqbp1;irf5;tfdp2;phf20;znf641;snapc4;aire;pax3;zkscan1;hsf4;msc;sirt6;znf280d;mef2d;hoxc8;rpl6;erg;hivep1;psip1;foxd4l1;prrx2;foxn2;pou5f1;dbp;thrsp;morf4l1;zscan2;zbtb32;suv420h1;mms19;sertad3;lzts1;znf667;nkx2-6;znf584;barx1;znf175;stat3;ezh1;prdm15;efcab6;tle4;brms1l;znf629;brca2;znf710;foxd2;cbfa2t2;tbr1;zhx2;dpf3;arid4b;e2f6;elp4;mycbp2;mlxipl;smad5;sirt2;zkscan5;npas2;mycl1;ncoa6;htatip2;ncoa1;nfatc1;ighmbp2;gbx1;hoxd8;hoxb13;znf576;jazf1;med18;trim28;nrbf2;crx;nr1h3;pou2f2;pitx2;suv39h1;atf2;rcbtb1;polr3g;isl2;yeats4;med24;znf713;gtf2i;scap;sox13;bmp6;gzf1;taf1a;hoxb4;dr1;mdm2;med11;znf329;znf555;nab1;ccdc59;smarce1;notch2;gtf3a;rere;zbtb7a;znf81;cry2;znf646;med6;atf5;etv1;znf672 |
| protein processing; | 14#99 | 0.024436 | psen1;furin;psen2;map3k12;sec11a;taok3;cav2;spcs3;irak1;psenen;yes1;myo3a;adamts13;ttn |
| regulation of lymphocyte activation; | 12#76 | 0.025532 | malt1;cdkn1a;cd47;il27ra;nck1;lat;traf6;ptprc;pag1;cd2;foxp3;ebi3 |
| response to biotic stimulus; | 31#289 | 0.025532 | pglyrp1;slc11a1;dnajb5;ppbp;cfb;tlr9;tlr3;twf2;ifnk;thbd;znf175;duox2;nod1;ccl19;cst11;cxcl12;il27ra;dnaja1;defb116;bpi;vapb;ptprc;krt8;ccdc130;hspd1;crnn;nod2;tlr6;defb124;tbk1;gsk3b |
| mitochondrial transport; | 9#48 | 0.025532 | ucp3;slc25a37;slc25a36;slc25a6;slc25a1;tomm34;tomm40;hspd1;fxc1 |
| cellular lipid catabolic process; | 9#48 | 0.025532 | pla2g4b;lipc;hexb;ppt1;sphk1;hsd17b11;aldh5a1;plcb3;smpd1 |
| transcription; | 282#3803 | 0.025546 | mcm6;zfp62;tead4;rab18;znf648;trim29;znf566;arid1b;znf300;l3mbtl2;znf275;znf187;zfpm2;calcoco1;znf644;vps72;lhx3;taf5;znf181;asf1a;nr2e1;notch1;rxrg;myod1;glis2;hoxc10;leo1;zbtb5;vgll3;rad54b;vsx1;mef2b;ncoa3;brca1;sox14;mael;pou5f2;myog;znf32;aff4;sp100;dmtf1;taf1b;nr1d1;pnrc2;nkx2-5;pfdn5;ecd;pou1f1;znf532;carhsp1;foxj3;supt3h;rdbp;edf1;nfyb;znf529;arntl2;bcl11b;sox9;pbx4;ccna2;nr4a1;etv2;rps14;rfc1;tcf15;irf2;ing4;foxp3;phb2;nr1h4;gtf2e1;phf17;cdc73;pqbp1;irf5;znf641;pax3;msc;znf280d;rpl6;hivep1;tcf23;thrsp;dbp;morf4l1;zscan2;zbtb32;suv420h1;mms19;smarcad1;sertad3;lzts1;znf667;znf584;znf175;stat3;prdm15;sohlh2;tle4;znf629;brms1l;znf710;cbfa2t2;dpf3;arid4b;e2f6;mycbp2;mlxipl;smad5;sirt2;zkscan5;npas2;mycl1;ncoa1;ighmbp2;gbx1;hoxd8;hoxb13;znf576;jazf1;med18;nrbf2;pitx2;isl2;znf713;gtf2i;sox13;taf1a;hoxb4;dr1;med11;znf329;znf555;ssbp2;notch2;gtf3a;trip11;znf81;znf646;irak1;med6;znf672;zfp37;pogk;znf212;foxn1;znf524;znf35;yaf2;egln1;foxr1;chmp1a;ttf1;znf345;znf34;grhl3;tox3;znrd1;znf23;brd8;nr0b2;smarcb1;rab35;tcf25;noto;gata3;znf470;ifnk;rab8b;znf683;znf688;znf395;nr4a3;casp8ap2;pycard;znf142;zbtb3;znf624;smarcd2;gtf2h2;ercc3;znf250;gabpa;tceb1;rfx2;atxn3;uimc1;tbx10;sp2;znf202;znf16;pbrm1;znf569;pou2f3;pias4;rpap1;jdp2;eya2;gtf2h5;dmrtb1;znf770;hes5;ilf3;ehf;ereg;znf22;zmiz2;klf8;znf238;rbak;srf;nfyc;tfeb;pcgf5;wwp1;hivep2;meox1;nkx1-2;notch4;elavl2;tfdp2;polr2f;phf20;snapc4;aire;zkscan1;rbm15;hsf4;sirt6;hoxc8;mef2d;l3mbtl3;papolb;erg;psip1;foxd4l1;foxn2;prrx2;pou5f1;nkx2-6;barx1;parp1;ezh1;tigd2;efcab6;brca2;foxd2;zhx2;tbr1;terf2;elp4;ncoa6;htatip2;nfatc1;tlr3;trim28;crx;nr1h3;suv39h1;pou2f2;rcbtb1;atf2;polr3g;med24;yeats4;polr3d;scap;bmp6;gzf1;smurf2;mdm2;smarce1;nab1;ccdc59;rere;zbtb7a;cry2;etv1;nod2;atf5 |
| regulation of cell activation; | 12#80 | 0.026583 | malt1;cdkn1a;cd47;il27ra;nck1;lat;traf6;ptprc;pag1;cd2;foxp3;ebi3 |
| detection of stimulus; | 12#80 | 0.026583 | gck;rtp4;pglyrp1;mkks;tlr3;nod2;rgr;kcnip1;rho;ppef2;nod1;rtp3 |
| cellular component assembly; | 72#813 | 0.026942 | ptk2;slu7;shprh;nck1;ddx1;scube1;fmod;gopc;vwf;aldh5a1;sept7;eif5a;sirt6;gpaa1;pick1;ttf1;ttn;smarcad1;ipo8;taf5;ap1s3;asf1a;sf3a1;cldn14;tmem48;eif4ebp1;sept11;dag1;cav2;capg;crnkl1;sirt2;gtf2h2;ncoa6;fanca;vamp4;ube2b;nup98;paip2b;actn2;sf3b3;traf1;apc;sbf2;arcn1;pex13;med24;atpif1;gtf2i;adipoq;trmt6;supt3h;srpk2;malt1;gga1;mdm2;eif4g3;ndufs8;cda;pbx4;napg;nod1;rps14;mapk8ip2;rps3a;cct6b;sycp3;irak1;pxn;ap4s1;nod2;gtf2e1 |
| regulation of cellular component organization and biogenesis; | 14#100 | 0.027084 | cxcl12;apc;clasp1;mapre1;nck1;katnb1;map2;avil;cav2;capg;arpc3;stk38l;sptbn2;terf2 |
| amino acid derivative metabolic process; | 13#90 | 0.027084 | slco4a1;dio1;dhps;tph1;afmid;snca;gata3;tmlhe;mat2a;aldh5a1;mat2b;sult1b1;ddc |
| endosome transport; | 8#40 | 0.027849 | vps54;gosr1;tom1;ehd1;rab35;als2;dclk1;stx16 |
| blood vessel development; | 20#164 | 0.027986 | adora2b;canx;cspg4;htatip2;edn1;sh2d2a;notch4;angpt1;sphk1;mmp19;angptl3;nkx2-5;ccm2;fgf1;c1galt1;ereg;smo;tnni3;atpif1;nppb |
| fertilization; | 10#58 | 0.028049 | wbp2nl;zp4;serpina5;clgn;izumo1;mfge8;hexb;fut10;adam21;fcgbp |
| membrane lipid catabolic process; | 6#24 | 0.028049 | pla2g4b;hexb;ppt1;sphk1;plcb3;smpd1 |
| intra-Golgi vesicle-mediated transport; | 5#17 | 0.028767 | napa;gosr1;cog2;napg;stx16 |
| protein tetramerization; | 5#17 | 0.028767 | cda;sbf2;atpif1;aldh5a1;actn2 |
| microtubule polymerization or depolymerization; | 7#32 | 0.028767 | apc;cenpj;clasp1;mapre1;katnb1;map2;cav2 |
| regulation of progression through mitotic cell cycle; | 7#32 | 0.028767 | chmp1a;cdc25c;brca2;btg4;sphk1;ilkap;dlg1 |
| sensory perception of mechanical stimulus; | 17#132 | 0.028767 | mkks;ercc3;kcnq1;gata3;hexb;myo3a;pcdh15;tnfrsf11a;pax3;actb;cldn14;cdh23;coch;myo15a;lhfpl5;ush1c;wfs1 |
| sensory perception of sound; | 17#132 | 0.028767 | mkks;ercc3;kcnq1;gata3;hexb;myo3a;pcdh15;tnfrsf11a;pax3;actb;cldn14;cdh23;coch;myo15a;lhfpl5;ush1c;wfs1 |
| positive regulation of cell proliferation; | 26#233 | 0.029279 | chrm1;cdkn1a;stambp;fgf7;nck1;edn1;il15;lifr;timp1;sphk1;cdc25b;tnfrsf11a;ebi3;capns1;flt3;cd47;hsf4;bmpr1a;dhps;hoxc10;ereg;ptprc;il12rb1;clec11a;smo;fgb |
| sex differentiation; | 12#81 | 0.029279 | lhcgr;bmpr1a;mkks;ereg;msh4;ide;sox9;brca2;dmrtb1;cdkl2;lhb;asb1 |
| response to radiation; | 14#101 | 0.029279 | cdkn1a;nhej1;gtf2h2;ercc3;abcg5;fen1;brca2;atm;mat2a;rgr;thbd;nphp4;rho;mc1r |
| glycerophospholipid metabolic process; | 14#101 | 0.029279 | pigq;pik3c2a;serinc2;gpaa1;chpt1;pigs;pigz;impa1;pla2g4b;pigb;lipc;pitpnm3;fabp3;aldh5a1 |
| cholesterol metabolic process; | 12#78 | 0.029279 | mbtps1;cyp11a1;soat1;lep;nr0b2;idi1;hmgcs1;fdft1;acaa2;lcat;osbpl5;scap |
| muscle contraction; | 20#165 | 0.029279 | cald1;gaa;prkca;myl3;myh4;nmur1;slc8a1;cacng1;kcnq1;pln;atp1a2;sphk1;tmod4;tacr2;dag1;tnni3;kcnma1;arg2;ttn;ednra |
| muscle system process; | 20#165 | 0.029279 | cald1;gaa;prkca;myl3;myh4;nmur1;slc8a1;cacng1;kcnq1;pln;atp1a2;sphk1;tmod4;tacr2;dag1;tnni3;kcnma1;arg2;ttn;ednra |
| regulation of T cell activation; | 10#59 | 0.030329 | malt1;cd47;nck1;lat;traf6;ptprc;pag1;cd2;foxp3;ebi3 |
| secretion#regulation of secretion; | 10#59 | 0.030329 | gck;edn1;snca;pycard;pde3b;nod2;stx1a;cplx3;foxp3;dph3 |
| reproductive process; | 32#305 | 0.030372 | cyp11a1;wwp1;izumo1;thbd;lhb;bmpr1a;serpina5;eif5a;wwp2;ide;pth;fut10;asb1;wbp2nl;mkks;smarcb1;clgn;msh4;sox9;pla2g4b;hexb;dmrtb1;cdkl2;adam21;zp4;lhcgr;vapb;ereg;cav2;mfge8;brca2;fcgbp |
| maintenance of localization; | 8#41 | 0.03059 | gaa;alb;ptprc;nfkbie;gpaa1;fth1;kdelr2;gopc |
| response to cAMP; | 4#11 | 0.03059 | cyp11a1;mat2a;thbd;duox2 |
| cofactor transport; | 4#11 | 0.03059 | slc19a1;ppt1;folr2;slc22a16 |
| receptor metabolic process; | 4#11 | 0.03059 | pick1;furin;als2;scap |
| cell projection biogenesis; | 9#50 | 0.030687 | fgd5;sdcbp;nck1;vav2;mkks;capg;arfip2;ube2b;actn2 |
| in utero embryonic development; | 9#50 | 0.030687 | smarcb1;ncoa6;edn1;pitpnb;plcg1;egln1;wnt3a;brca2;ptprr |
| chemotaxis; | 18#144 | 0.030741 | prkca;map2k1;cx3cr1;ppbp;cmtm8;ltb4r2;ccl27;mapk14;il16;ccr4;ccl19;cxcl12;ptafr;ccr3;cmtm7;ccr9;cxcl1;cmtm4 |
| taxis; | 18#144 | 0.030741 | prkca;map2k1;cx3cr1;ppbp;cmtm8;ltb4r2;ccl27;mapk14;il16;ccr4;ccl19;cxcl12;ptafr;ccr3;cmtm7;ccr9;cxcl1;cmtm4 |
| positive regulation of kinase activity; | 18#144 | 0.030741 | chrm1;adora2b;malt1;taok2;vav2;edn1;traf6;tlr3;sphk1;dgki;als2;dgkg;ereg;gap43;ptprc;pick1;irak1;tlr6 |
| cellular protein complex assembly; | 11#69 | 0.030741 | ptk2;nck1;tmem48;mapk8ip2;pex13;ndufs8;ipo8;pick1;fmod;pxn;nup98 |
| intracellular protein transport across a membrane; | 11#69 | 0.030741 | tmem48;tram1;sec61g;nup153;rrbp1;fxc1;nup160;sec61a1;timm50;nup98;nup210 |
| behavior; | 31#294 | 0.031266 | oprd1;cyp11a1;prkca;map2k1;cx3cr1;mkks;npas2;ppbp;cmtm8;ccl27;ltb4r2;hexb;mapk14;kiss1r;il16;gcg;nphp4;chl1;als2;ccr4;ccl19;cxcl12;avpr1a;lep;pex13;ptafr;cmtm7;ccr3;cxcl1;ccr9;cmtm4 |
| microtubule cytoskeleton organization and biogenesis; | 14#102 | 0.031503 | apc;clasp1;mapre1;kif23;katnb1;map2;ndc80;bub1b;tubg1;cav2;cks2;cenpj;ube2b;tubgcp6 |
| phospholipid biosynthetic process; | 13#92 | 0.031526 | pigq;pik3c2a;pisd;gpaa1;chpt1;pigs;agpat4;sgms2;pigz;impa1;pigb;hexb;fabp3 |
| negative regulation of gluconeogenesis; | 2#2 | 0.031526 | gck;adipoq |
| pyrimidine ribonucleoside metabolic process; | 2#2 | 0.031526 | cda;upp2 |
| centrosome duplication#centriole replication; | 2#2 | 0.031526 | cntrob;brca1 |
| sulfur amino acid biosynthetic process#S-adenosylmethionine biosynthetic process; | 2#2 | 0.031526 | mat2a;mat2b |
| sphingosine metabolic process; | 2#2 | 0.031526 | sphk1;sgpp1 |
| DNA damage response, signal transduction by p53 class mediator resulting in transcription of p21 class mediator; | 2#2 | 0.031526 | brca2;brca1 |
| malate-aspartate shuttle; | 2#2 | 0.031526 | slc25a12;slc25a13 |
| lipoprotein transport; | 2#2 | 0.031526 | zdhhc17;angptl3 |
| dolichol-linked oligosaccharide biosynthetic process; | 2#2 | 0.031526 | alg2;mpdu1 |
| DNA damage response, signal transduction resulting in transcription; | 2#2 | 0.031526 | brca2;brca1 |
| nuclear pore organization and biogenesis; | 2#2 | 0.031526 | tmem48;nup98 |
| macromolecular complex assembly; | 67#756 | 0.032634 | ptk2;slu7;shprh;nck1;ddx1;scube1;fmod;gopc;vwf;aldh5a1;sept7;eif5a;gpaa1;pick1;ttf1;smarcad1;ipo8;taf5;ap1s3;asf1a;sf3a1;cldn14;tmem48;eif4ebp1;sept11;dag1;cav2;capg;crnkl1;gtf2h2;ncoa6;fanca;vamp4;nup98;paip2b;sf3b3;traf1;actn2;apc;sbf2;arcn1;pex13;med24;atpif1;gtf2i;adipoq;trmt6;supt3h;srpk2;malt1;gga1;mdm2;eif4g3;cda;ndufs8;pbx4;nod1;napg;rps14;mapk8ip2;rps3a;cct6b;irak1;pxn;nod2;ap4s1;gtf2e1 |
| negative regulation of biosynthetic process; | 9#51 | 0.03268 | furin;eif4ebp1;pdzd3;edn1;paip2b;foxp3;scap;brca1;asb1 |
| response to nutrient; | 8#42 | 0.03268 | sstr2;cyp11a1;cfb;gcgr;chmp1a;lct;abcg5;brca2 |
| skeletal muscle development#skeletal muscle fiber development; | 8#42 | 0.03268 | notch1;boc;myod1;myog;cav2;tnc;ttn;als2 |
| muscle development#muscle fiber development; | 8#42 | 0.03268 | notch1;boc;myod1;myog;cav2;tnc;ttn;als2 |
| detection of chemical stimulus; | 5#18 | 0.03268 | gck;nod2;rtp4;kcnip1;rtp3 |
| cholesterol transport; | 5#18 | 0.03268 | cav1;lipc;angptl3;lcat;osbpl5 |
| sterol transport; | 5#18 | 0.03268 | cav1;lipc;angptl3;lcat;osbpl5 |
| interferon-gamma production; | 5#18 | 0.03268 | tlr9;tlr3;il27ra;foxp3;ebi3 |
| regulation of T cell mediated immunity; | 3#6 | 0.033043 | malt1;traf6;foxp3 |
| regulation of carbohydrate biosynthetic process; | 3#6 | 0.033043 | gck;dyrk2;adipoq |
| Notch receptor processing; | 3#6 | 0.033043 | psenen;psen1;psen2 |
| regulation of interleukin-2 production; | 3#6 | 0.033043 | malt1;traf6;foxp3 |
| activation of immune response; | 11#71 | 0.035453 | malt1;c4a;cfi;masp1;traf6;ptprc;cfb;c8b;c1qc;bcar1;nod2 |
| carbohydrate metabolic process; | 70#800 | 0.035995 | pigq;uap1;idua;man2b2;naglu;gk2;pnpt1;glt6d1;gys2;arsb;gck;hs3st3b1;b3gat1;lep;khk;galnt5;gba3;itpka;phkg1;fuca2;ppp1r2;gk;pglyrp1;sds;suclg1;pfkp;lyg2;lct;chst3;pofut2;st6gal2;lctl;ldha;glt25d2;phkg2;gad2;hibadh;cercam;cog2;gaa;manba;me3;aldob;fyb;ecd;dyrk2;gne;amdhd2;fut3;fut10;adipoq;mat2b;gcnt3;gal3st3;gcnt4;hexb;ganab;ext2;galt;hk1;impa1;lipc;glb1l;gfpt2;mgat2;uevld;mpdu1;pgk2;gsk3b;slc3a1 |
| protein amino acid ADP-ribosylation; | 6#26 | 0.036196 | gna15;sirt2;parp4;gna11;sirt6;parp1 |
| negative regulation of cellular component organization and biogenesis; | 9#52 | 0.036213 | apc;clasp1;mapre1;katnb1;map2;avil;capg;sptbn2;terf2 |
| cation homeostasis; | 23#203 | 0.036213 | prkca;atp1a1;edn1;f2rl1;pln;fth1;gna15;atp1a2;hexb;ppt1;ccr4;ccl19;cxcl12;hp;avpr1a;tpt1;ptprc;tnni3;cutc;pth;ccr3;ccr9;ednra |
| regulation of body fluid levels; | 16#125 | 0.036419 | edn1;f2rl1;scube1;slc22a4;proc;vwf;mmrn1;thbd;mst1;tbxas1;fgb;slc34a1;f2rl3;adamts13;nppb;p2ry1 |
| DNA damage response, signal transduction; | 8#43 | 0.036552 | brca2;cideb;dyrk2;atrip;ccna2;sfn;chek1;brca1 |
| negative regulation of protein metabolic process; | 14#104 | 0.036686 | apc;furin;clasp1;eif4ebp1;serpina5;katnb1;map2;avil;capg;timp1;sptbn2;paip2b;foxp3;asb1 |
| chromatin remodeling; | 10#62 | 0.037982 | sirt2;smarcb1;arid1b;sirt6;pbrm1;sycp3;ttf1;foxp3;asf1a;smarcd2 |
| T-helper 1 type immune response; | 4#12 | 0.038085 | il27ra;il18bp;tlr6;ebi3 |
| response to glucocorticoid stimulus; | 4#12 | 0.038085 | acads;map2k1;aldob;lcat |
| nucleosome assembly; | 2#187 | -0.03809 | shprh;asf1a |
| cell part morphogenesis; | 23#204 | 0.038085 | stx2;sema4f;fgd5;sdcbp;nck1;vav2;mkks;ntng2;wnt3a;spag6;arfip2;ube2b;b3gnt2;chl1;als2;actn2;rtn4;tnn;capg;lhfpl5;fez2;dbn1;nrcam |
| cell projection morphogenesis#cell projection organization and biogenesis; | 23#204 | 0.038085 | stx2;sema4f;fgd5;sdcbp;nck1;vav2;mkks;ntng2;wnt3a;spag6;arfip2;ube2b;b3gnt2;chl1;als2;actn2;rtn4;tnn;capg;lhfpl5;fez2;dbn1;nrcam |
| cell projection morphogenesis; | 23#204 | 0.038085 | stx2;sema4f;fgd5;sdcbp;nck1;vav2;mkks;ntng2;wnt3a;spag6;arfip2;ube2b;b3gnt2;chl1;als2;actn2;rtn4;tnn;capg;lhfpl5;fez2;dbn1;nrcam |
| anti-apoptosis; | 20#170 | 0.038861 | malt1;stambp;eef1a2;sfrp1;api5;htatip2;sphk1;rtn4;notch2;psen1;akt1s1;ntf3;tpt1;bcl2a1;naip;mapk8ip2;snca;atf5;pik3r2;aven |
| DNA damage response, signal transduction resulting in induction of apoptosis; | 5#19 | 0.039579 | brca2;cideb;dyrk2;sfn;brca1 |
| spindle organization and biogenesis; | 5#19 | 0.039579 | kif23;ndc80;bub1b;tubg1;cks2 |
| developmental maturation; | 9#53 | 0.039579 | pick1;brca2;timp1;dsg4;ttn;mreg;ereg;nrcam;hes5 |
| phosphoinositide biosynthetic process; | 7#35 | 0.039709 | pigq;pik3c2a;impa1;pigb;gpaa1;pigs;pigz |
| secretory pathway; | 26#240 | 0.039939 | lat;nmur1;gopc;sec24a;unc13b;rab3d;tmed10;napg;trappc1;sar1a;napa;snca;cplx1;stx16;rims4;sec31a;kcnma1;pick1;snap29;stx1a;gosr1;stx18;cplx3;cog2;stxbp1;osbpl5 |
| production of molecular mediator of immune response; | 6#27 | 0.04187 | malt1;poll;il27ra;traf6;foxp3;ptprc |
| response to bacterium; | 14#106 | 0.04464 | il27ra;defb116;pglyrp1;bpi;slc11a1;ppbp;tlr9;tlr3;nod2;thbd;tlr6;defb124;nod1;cst11 |
| muscle development; | 20#172 | 0.045124 | srf;gaa;sirt2;myog;tead4;chodl;pln;tnc;als2;nkx2-5;notch1;boc;myod1;mef2d;bves;cav2;tnni3;mef2b;unc45b;ttn |
| induction of apoptosis by intracellular signals; | 7#36 | 0.04581 | cdkn1a;prkca;brca2;cideb;dyrk2;sfn;brca1 |
| protein targeting to membrane; | 7#36 | 0.04581 | atg4c;sdcbp;taok2;rtp4;arl6ip1;tram1;rtp3 |
| double-strand break repair; | 7#36 | 0.04581 | rad52;nhej1;fen1;brca2;rad21;rad54b;brca1 |
| multicellular organismal development#tube development; | 15#117 | 0.045958 | gzf1;nkx2-5;shank3;fzd6;bmpr1a;edn1;pdpn;adrb1;cav2;notch4;crispld2;nkx2-6;nppb;nts;hsd11b1 |
| regulation of translation; | 21#184 | 0.046678 | ddx1;nanos3;eif4g3;gata3;tlr9;tlr3;paip2b;ebi3;upf1;furin;pum2;eif4ebp1;krt7;eif5a;pstk;ereg;tlr6;foxp3;asb1;trmt6;scap |
| lipoprotein biosynthetic process; | 9#55 | 0.048646 | pigq;atg7;rabggta;gpaa1;pigs;pigz;pigb;zdhhc1;lcat |
| blood circulation#regulation of blood pressure; | 9#55 | 0.048646 | avpr1a;edn1;adrb1;gcgr;corin;fgb;tnni3;nppb;ace |
| regulation of lipid metabolic process; | 6#28 | 0.048646 | edf1;angptl3;adipoq;scap;bmp6;brca1 |
| transcription from RNA polymerase III promoter; | 6#28 | 0.048646 | znf345;polr3d;polr3g;snapc4;brca1;gtf3a |
| humoral immune response; | 11#75 | 0.048646 | aire;c4a;pou2f2;cfi;masp1;pdcd1;cfb;ms4a2;c8b;c1qc;ebi3 |
| response to corticosteroid stimulus; | 4#13 | 0.048646 | acads;map2k1;aldob;lcat |
| membrane protein ectodomain proteolysis; | 4#13 | 0.048646 | psenen;psen1;timp1;psen2 |
| regulation of adaptive immune response based on somatic recombination of immune receptors built from immunoglobulin superfamily domains.; | 4#13 | 0.048646 | malt1;il27ra;traf6;foxp3 |
| regulation of adaptive immune response; | 4#13 | 0.048646 | malt1;il27ra;traf6;foxp3 |
| membrane protein proteolysis; | 4#13 | 0.048646 | psenen;psen1;timp1;psen2 |
| response to starvation; | 4#13 | 0.048646 | lct;acads;aldob;alb |
| centrosome duplication; | 3#7 | 0.048667 | brca2;cntrob;brca1 |
| male meiosis; | 3#7 | 0.048667 | brca2;ccna1;sycp3 |
| mesoderm morphogenesis#mesoderm formation#mesodermal cell differentiation; | 3#7 | 0.048667 | wnt3a;trim15;eya2 |
| mesoderm morphogenesis#mesoderm formation#mesodermal cell differentiation#mesodermal cell fate commitment; | 3#7 | 0.048667 | wnt3a;trim15;eya2 |
| regulation of transcription from RNA polymerase III promoter; | 3#7 | 0.048667 | znf345;polr3g;brca1 |
| phosphatidylcholine metabolic process; | 3#7 | 0.048667 | lipc;fabp3;chpt1 |
| regulation of the force of heart contraction; | 3#7 | 0.048667 | prkca;adrb1;pln |
