# Supplementary material for: Genome-wide analysis reveals signatures of selection for important traits in domestic sheep from different ecoregions
Source: BMC Genomics. 2016 Nov 3;17:863. doi: 10.1186/s12864-016-3212-2 (PMC5094087; doi:10.1186/s12864-016-3212-2)
Supplement: Additional file 12: Table S10. — Enriched KEGG pathways among genes containing missense SNPs in promoter regions in Mongolian sheep but not in Small-tailed Han sheep or Duolang sheep. (DOC 50 kb) [file 12864_2016_3212_MOESM12_ESM.doc]

**Additional file 12: Table S10**. Enriched KEGG pathways among genes containing missense SNPs in promoter regions in Mongolian sheep but not in Small-tailed Han sheep or Duolang sheep.

| Category | Term | Count | P  Value | Genes |
| --- | --- | --- | --- | --- |
| KEGG_PATHWAY | hsa04370:VEGF signaling pathway | 19 | 0.01255 | PRKCA, MAP2K1, MAP2K2, SPHK1, PPP3R2, MAPKAPK2, PXN, SH2D2A, PTK2, PLCG1, MAPK14, PLA2G12A, PLA2G12B, PIK3R5, PLA2G2C, PLA2G4B, SHC2, PIK3R2, NFATC1 |
| KEGG_PATHWAY | hsa04020:Calcium signaling pathway | 35 | 0.02623 | ADCY3, GNA15, ADORA2B, TACR2, GNA11, LHCGR, PPP3R2, ITPKA, EDNRA, PLCB3, GRIN2C, PDE1A, PLCD4, CALML6, PRKACB, HTR5A, PRKCA, SLC8A1, PHKG1, SLC25A6, MYLK3, PHKG2, SPHK1, CACNA1I, MYLK2, P2RX4, ADRB1, PLCG1, CHRM1, PLN, LTB4R2, P2RX2, AVPR1A, CALM3, PTAFR |
| KEGG_PATHWAY | hsa04270:Vascular smooth muscle contraction | 24 | 0.032542 | PRKCA, ADCY3, KCNMA1, ARHGEF1, ADORA2B, MAP2K1, MAP2K2, GNA11, MYLK3, CALD1, MRVI1, MYLK2, EDNRA, PLCB3, PTGIR, PLA2G12A, PLA2G12B, GUCY1A2, AVPR1A, CALM3, PLA2G2C, CALML6, PRKACB, PLA2G4B |
| KEGG_PATHWAY | hsa04722:Neurotrophin signaling pathway | 26 | 0.032693 | NFKBIE, FASLG, MAPKAPK2, BDNF, MAP3K1, SOS1, SH2B3, CALML6, PIK3R5, CSK, TRAF6, SHC2, RAPGEF1, PIK3R2, IRAK2, IRAK1, NTF4, NTF3, MAP2K1, MAP2K2, MAPK10, PSEN1, PLCG1, MAPK14, GSK3B, CALM3 |
| KEGG_PATHWAY | hsa00564:Glycerophospholipid metabolism | 16 | 0.043543 | CPT1B, NAT6, LYPLA2, LYPLA1, PISD, DGKI, CHPT1, DGKG, LCAT, PLA2G12A, PLA2G12B, GNPAT, PLA2G2C, AGPAT4, PLA2G4B, CHAT |
| KEGG_PATHWAY | hsa00512:O-Glycan biosynthesis | 9 | 0.047456 | GCNT4, GALNT1, GCNT3, GALNTL4, GALNT5, GALNT11, GALNT12, C1GALT1, ST6GALNAC1 |
| KEGG_PATHWAY | hsa04910:Insulin signaling pathway | 27 | 0.049276 | HK1, PDE3B, RPS6KB2, RPS6KB1, PRKAR2B, EIF4EBP1, PPP1R3B, SOS1, GYS2, PIK3R5, CALML6, PRKACB, TRIP10, SHC2, RAPGEF1, PIK3R2, MAP2K1, PHKG1, MAP2K2, PHKG2, ACACA, MAPK10, RPTOR, GCK, GSK3B, CALM3, PTPN1 |
| KEGG_PATHWAY | hsa00970:Aminoacyl-tRNA biosynthesis | 11 | 0.049952 | NARS, PSTK, PARS2, RARS, HARS2, LARS, FARSB, QARS, MTFMT, MARS, DTD1, EARS2 |
| KEGG_PATHWAY | hsa04670:Leukocyte transendothelial migration | 24 | 0.055075 | PRKCA, ACTB, F11R, ITGAL, CLDN17, CLDN4, NCF1, BCAR1, MYL12B, ACTN2, MYL10, VAV2, CXCL12, PXN, CTNNA3, CLDN14, PTK2, ARHGAP5, PLCG1, MAPK14, PIK3R5, TXK, JAM3, PIK3R2 |
| KEGG_PATHWAY | hsa00260:Glycine, serine and threonine metabolism | 9 | 0.056488 | GLYCTK, ALAS1, SDS, DLD, DMGDH, PSAT1, SARDH, GLDC, AOC3 |
| KEGG_PATHWAY | hsa00511:Other glycan degradation | 6 | 0.059994 | MAN2B2, HEXB, ENGASE, FUCA2, NEU2, MANBA |
| KEGG_PATHWAY | hsa04130:SNARE interactions in vesicular transport | 10 | 0.071852 | SNAP29, STX1A, STX2, STX18, STX16, USE1, VAMP4, GOSR1, STX11, STX1B |
| KEGG_PATHWAY | hsa00600:Sphingolipid metabolism | 10 | 0.082445 | SPTLC1, SGMS2, SGPP1, SGPP2, ACER1, SPHK1, UGCG, SMPD1, NEU2, GAL3ST1 |
| KEGG_PATHWAY | hsa03430:Mismatch repair | 7 | 0.08894 | RFC5, RFC3, RFC1, SSBP1, MSH2, POLD2, MLH3 |
