# Supplementary material for: Genome-wide analysis reveals signatures of selection for important traits in domestic sheep from different ecoregions
Source: BMC Genomics. 2016 Nov 3;17:863. doi: 10.1186/s12864-016-3212-2 (PMC5094087; doi:10.1186/s12864-016-3212-2)
Supplement: Additional file 13: Table S11. — Enriched GO terms among genes containing missense SNPs in promoter regions in both Small-tailed Han sheep and Duolang sheep, but not in Mongolian sheep. (DOC 71 kb) [file 12864_2016_3212_MOESM13_ESM.doc]

**Additional file 13: Table S11**. Enriched GO terms among genes containing missense SNPs in promoter regions in both Small-tailed Han sheep and Duolang sheep, but not in Mongolian sheep.

| GO term | Gene count | P value | genes |
| --- | --- | --- | --- |
| cell morphogenesis#establishment and/or maintenance of cell polarity; | 2#29 | 0.055675 | ccl4;map7 |
| fermentation#glycerol biosynthetic process; | 1#1 | 0.055675 | pck1 |
| positive regulation of odontogenesis of dentine-containing teeth; | 1#1 | 0.055675 | ngfr |
| response to cobalt ion; | 1#1 | 0.055675 | d2hgdh |
| polyol biosynthetic process; | 1#1 | 0.055675 | pck1 |
| positive regulation of odontogenesis; | 1#1 | 0.055675 | ngfr |
| negative regulation of muscle development; | 1#1 | 0.055675 | ngfr |
| response to manganese ion; | 1#1 | 0.055675 | d2hgdh |
| triacylglycerol biosynthetic process#glycerol biosynthetic process from pyruvate; | 1#1 | 0.055675 | pck1 |
| fermentation; | 1#1 | 0.055675 | pck1 |
| detection of abiotic stimulus; | 2#45 | 0.068052 | tub;ngfr |
| response to external stimulus; | 5#633 | 0.06895 | tub;ngfr;chst4;ccl4;rnase2 |
| detection of external stimulus; | 2#50 | 0.070924 | tub;ngfr |
| leucine catabolic process; | 1#2 | 0.074181 | mccc2 |
| odontogenesis#regulation of odontogenesis; | 1#2 | 0.074181 | ngfr |
| negative regulation of T cell receptor signaling pathway; | 1#3 | 0.083395 | elf1 |
| negative regulation of antigen receptor-mediated signaling pathway; | 1#3 | 0.083395 | elf1 |
| odontogenesis of dentine-containing teeth#regulation of odontogenesis of dentine-containing teeth; | 1#3 | 0.083395 | ngfr |
| leucine metabolic process; | 1#3 | 0.083395 | mccc2 |
| muscle development#regulation of muscle development; | 1#3 | 0.083395 | ngfr |
| fatty acid biosynthetic process; | 2#73 | 0.085473 | mgst2;fa2h |
| negative regulation of developmental process; | 2#77 | 0.085473 | ngfr;ush2a |
| triacylglycerol biosynthetic process; | 1#4 | 0.085473 | pck1 |
| detection of temperature stimulus; | 1#4 | 0.085473 | ngfr |
| nervous system development#nerve development; | 1#4 | 0.085473 | ngfr |
| multicellular organismal development#system development#organ development#maintenance of organ identity; | 1#4 | 0.085473 | ush2a |
| detection of stimulus; | 2#80 | 0.085688 | tub;ngfr |
| organic acid biosynthetic process; | 2#83 | 0.085688 | mgst2;fa2h |
| carboxylic acid biosynthetic process; | 2#83 | 0.085688 | mgst2;fa2h |
| mitochondrial depolarization; | 1#5 | 0.086747 | aifm3 |
| single strand break repair; | 1#5 | 0.086747 | xrcc1 |
| response to zinc ion; | 1#5 | 0.086747 | d2hgdh |
| monocarboxylic acid metabolic process; | 3#287 | 0.095107 | mgst2;fa2h;pck1 |
| skin development; | 1#6 | 0.095107 | ngfr |
| T cell receptor signaling pathway#regulation of T cell receptor signaling pathway; | 1#6 | 0.095107 | elf1 |
| neutral lipid biosynthetic process; | 1#7 | 0.097697 | pck1 |
| acylglycerol biosynthetic process; | 1#7 | 0.097697 | pck1 |
| response to chemical stimulus; | 4#589 | 0.097697 | ccl4;d2hgdh;angptl7;rnase2 |
| generation of precursor metabolites and energy; | 5#938 | 0.097697 | aifm3;fa2h;pck1;atp5g1;ndufa2 |
| glycerol ether biosynthetic process; | 1#8 | 0.097697 | pck1 |
| protein amino acid sulfation; | 1#8 | 0.097697 | chst4 |
| glycerolipid biosynthetic process; | 1#8 | 0.097697 | pck1 |
| sulfation; | 1#8 | 0.097697 | chst4 |
| response to stimulus; | 11#3553 | 0.097697 | tub;chst4;ush2a;xrcc1;elf1;ngfr;ccl4;d2hgdh;ada;angptl7;rnase2 |
| biological_process; | 44#24743 | 0.097697 | znf599;chst4;ubtd2;rsc1a1;amica1;elf1;ptpdc1;myh8;pck1;zim3;ints8;fgfbp1;mccc2;usp28;tub;mgst2;fa2h;golt1a;slc2a12;ngfr;selp;ccl4;angptl7;atp5g1;prkcz;u2af1l4;irx1;galnt7;a1bg;aifm3;etnk2;ada;rnase2;ush2a;xrcc1;pdzd2;mt1e;map7;d2hgdh;ecel1;eif3f;hmgn3;ndufa2;tgm6 |
| biological adhesion; | 5#960 | 0.097697 | pdzd2;chst4;selp;ccl4;amica1 |
| cell adhesion; | 5#960 | 0.097697 | pdzd2;chst4;selp;ccl4;amica1 |
| lipid biosynthetic process; | 3#333 | 0.097697 | mgst2;fa2h;pck1 |
| caspase activation via cytochrome c; | 1#9 | 0.097697 | aifm3 |
| branched chain family amino acid catabolic process; | 1#9 | 0.097697 | mccc2 |
| membrane depolarization; | 1#9 | 0.097697 | aifm3 |
