# Supplementary material for: Genome-wide analysis reveals signatures of selection for important traits in domestic sheep from different ecoregions
Source: BMC Genomics. 2016 Nov 3;17:863. doi: 10.1186/s12864-016-3212-2 (PMC5094087; doi:10.1186/s12864-016-3212-2)

**Additional file 14: Figure S3.** Biological processes and KEGG pathway enrichment in genes containing SNPs in promoter regions. A, Biological processes enrichment in genes containing SNPs in promoter regions in Mongolian sheep but not in Small-tailed Han sheep or Duolang sheep. B, Biological processes enrichment in genes containing SNPs in promoter regions in both Small-tailed Han sheep and Duolang sheep, but not in Mongolian sheep. C, KEGG pathway enrichment in genes containing SNPs in promoter regions in Mongolian sheep but not in Small-tailed Han sheep or Duolang sheep. Biological processes related to reproduction are labeled in red.


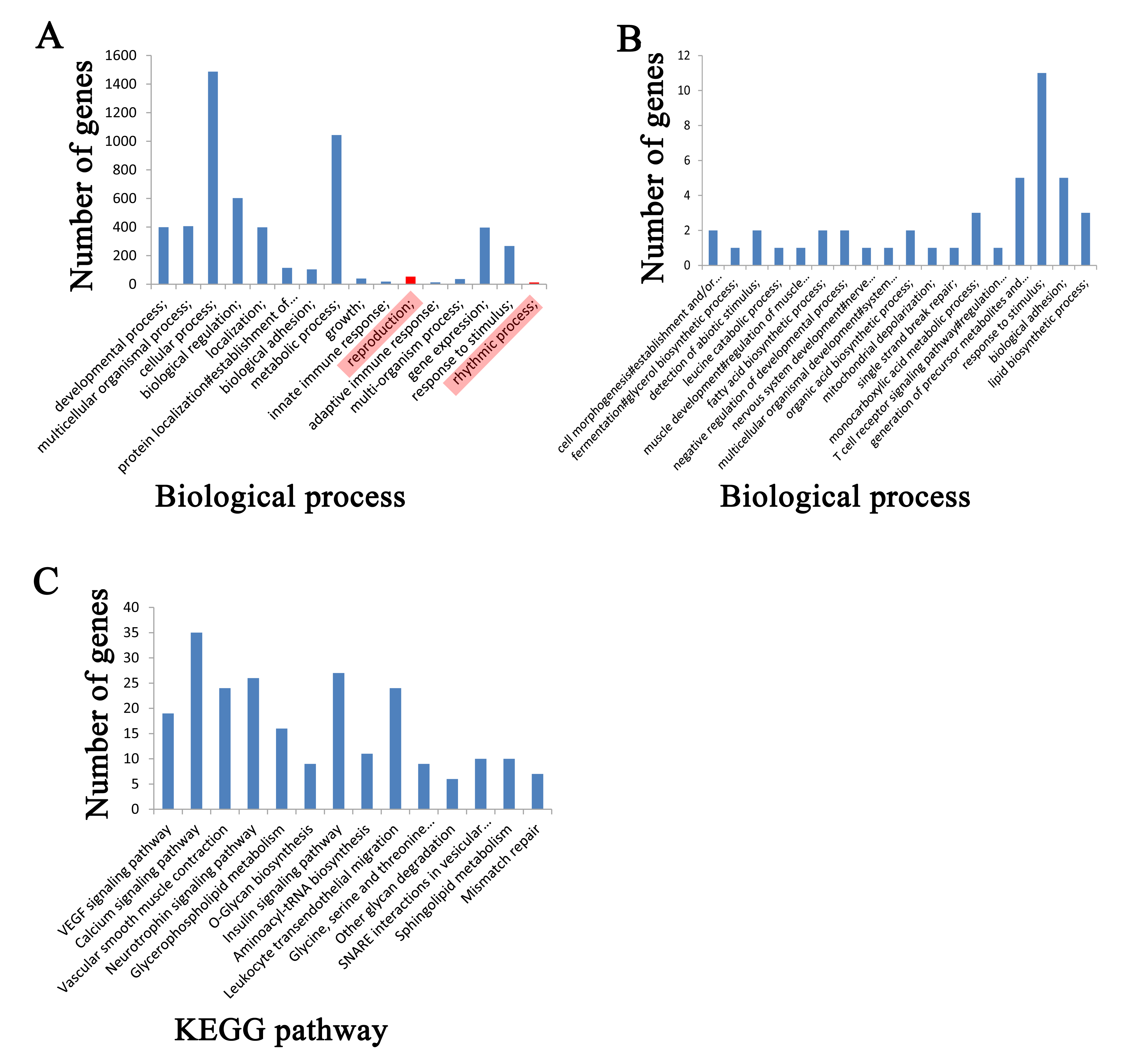

Supplement: Additional file 14: Figure S3. — Biological processes and KEGG pathway enrichment in genes containing SNPs in promoter regions. A, Biological processes enrichment in genes containing SNPs in promoter regions in Mongolian sheep but not in Small-tailed Han sheep or Duolang sheep. B, Biological processes enrichment in genes containing SNPs in promoter regions in both Small-tailed Han sheep and Duolang sheep, but not in Mongolian sheep. C, KEGG pathway enrichment in genes containing SNPs in promoter regions in Mongolian sheep but not in Small-tailed Han sheep or Duolang sheep. Biological processes related to reproduction are labeled in red. (DOC 1117 kb) [file 12864_2016_3212_MOESM14_ESM.doc]
