# Supplementary material for: Genome-wide analysis reveals signatures of selection for important traits in domestic sheep from different ecoregions
Source: BMC Genomics. 2016 Nov 3;17:863. doi: 10.1186/s12864-016-3212-2 (PMC5094087; doi:10.1186/s12864-016-3212-2)
Supplement: Additional file 15: Table S12. — The protein coding SNPs detected by SIFT and Provean analysis. (DOC 29 kb) [file 12864_2016_3212_MOESM15_ESM.doc]

**Additional file 15: Table S12.** The protein coding SNPs detected by SIFT and Provean analysis.

| breeds | Total | Not_foundin_dbSNP | PROVEAN_neutral | PROVEAN_deleterious | PROVEAN_NA | SIFT_  tolerated | SIFT_  damaging | SIFT_NA |
| --- | --- | --- | --- | --- | --- | --- | --- | --- |
| Mongolian sheep | 565 | 565 | 77 | 38 | 450 | 60 | 52 | 453 |
| Small-tailed Han sheep | 490 | 490 | 73 | 38 | 379 | 60 | 39 | 391 |
| Duolang sheep | 320 | 319 | 42 | 21 | 257 | 34 | 28 | 258 |
