# Supplementary material for: Genome-wide analysis reveals signatures of selection for important traits in domestic sheep from different ecoregions
Source: BMC Genomics. 2016 Nov 3;17:863. doi: 10.1186/s12864-016-3212-2 (PMC5094087; doi:10.1186/s12864-016-3212-2)

**Additional file 16: Figure S4.** The distributions of heterozygosity (*H*_P_) and the average fixation index (*F*_ST_) between any two of the 3 resequenced breeds for autosomal 200-kb windows.


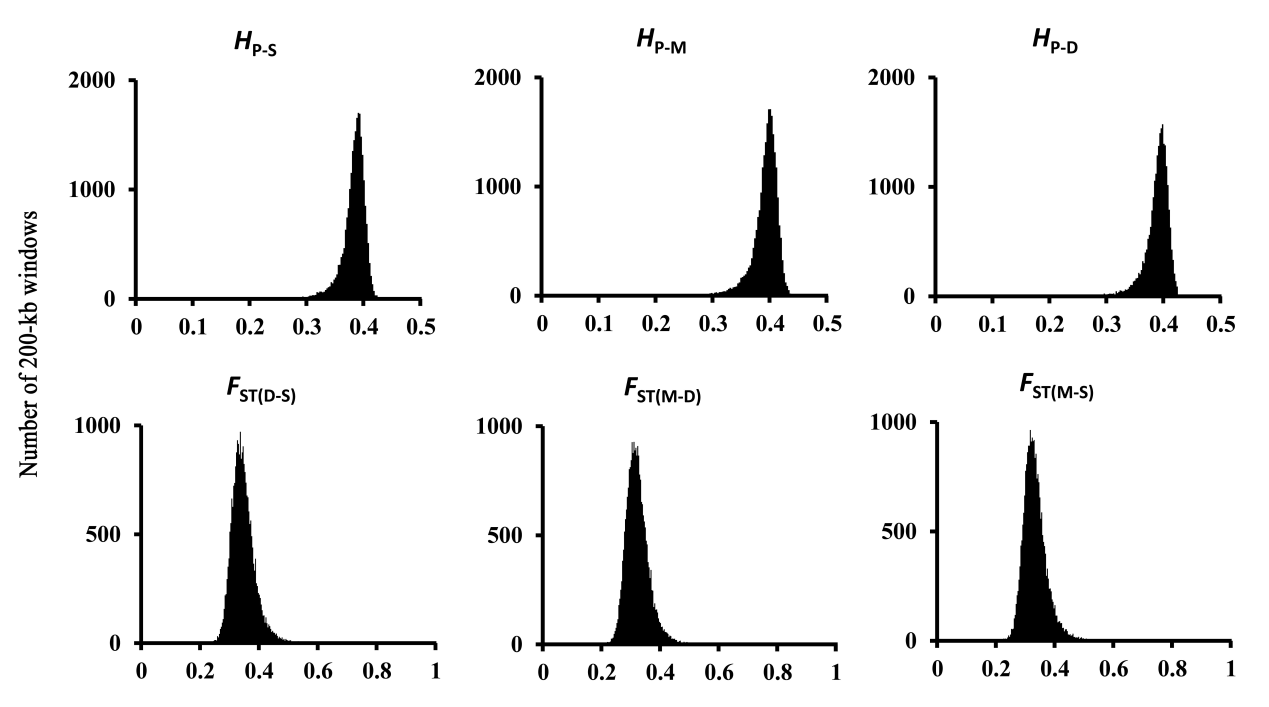

Supplement: Additional file 16: Figure S4. — The distributions of heterozygosity (H P) and the average fixation index (F ST) between any two of the 3 resequenced breeds for autosomal 200-kb windows. (DOC 107 kb) [file 12864_2016_3212_MOESM16_ESM.doc]
