# Supplementary material for: Genome-wide analysis reveals signatures of selection for important traits in domestic sheep from different ecoregions
Source: BMC Genomics. 2016 Nov 3;17:863. doi: 10.1186/s12864-016-3212-2 (PMC5094087; doi:10.1186/s12864-016-3212-2)
Supplement: Additional file 18: Table S13. — Genomic regions under selection in Small-tailed Han sheep. (DOC 126 kb) [file 12864_2016_3212_MOESM18_ESM.doc]

**Additional file 18: Table S13.** Genomic regions under selection in Small-tailed Han sheep

| Number | Chromosome | Start | End |
| --- | --- | --- | --- |
| 1 | 1 | 10600000 | 10800000 |
| 2 | 1 | 24900000 | 25300000 |
| 3 | 1 | 27800000 | 28000000 |
| 4 | 1 | 94900000 | 95200000 |
| 5 | 1 | 118000000 | 118700000 |
| 6 | 1 | 119400000 | 119700000 |
| 7 | 1 | 266600000 | 266800000 |
| 8 | 2 | 31300000 | 31500000 |
| 9 | 2 | 58500000 | 58800000 |
| 10 | 2 | 64200000 | 64500000 |
| 11 | 2 | 71000000 | 71300000 |
| 12 | 2 | 74200000 | 74400000 |
| 13 | 2 | 82200000 | 82400000 |
| 14 | 2 | 90800000 | 91000000 |
| 15 | 2 | 104100000 | 104900000 |
| 16 | 2 | 111400000 | 112400000 |
| 17 | 2 | 115100000 | 115300000 |
| 18 | 2 | 117900000 | 118100000 |
| 19 | 2 | 122000000 | 122700000 |
| 20 | 2 | 194600000 | 194800000 |
| 21 | 2 | 214300000 | 214500000 |
| 22 | 2 | 218500000 | 218700000 |
| 23 | 2 | 219400000 | 219700000 |
| 24 | 2 | 234400000 | 234600000 |
| 25 | 2 | 248000000 | 248200000 |
| 26 | 3 | 44800000 | 45200000 |
| 27 | 3 | 105300000 | 105600000 |
| 28 | 3 | 106200000 | 106400000 |
| 29 | 3 | 106700000 | 107100000 |
| 30 | 3 | 121100000 | 121300000 |
| 31 | 3 | 124800000 | 125000000 |
| 32 | 3 | 170800000 | 171000000 |
| 33 | 3 | 213400000 | 213600000 |
| 34 | 4 | 29200000 | 29400000 |
| 35 | 4 | 63100000 | 63300000 |
| 36 | 4 | 68700000 | 69000000 |
| 37 | 4 | 92800000 | 93000000 |
| 38 | 4 | 101500000 | 102100000 |
| 39 | 4 | 103700000 | 103900000 |
| 40 | 5 | 40800000 | 41700000 |
| 41 | 5 | 51500000 | 51700000 |
| 42 | 5 | 57900000 | 58100000 |
| 43 | 5 | 107200000 | 108000000 |
| 44 | 6 | 17200000 | 17400000 |
| 45 | 6 | 19900000 | 20100000 |
| 46 | 6 | 24700000 | 25000000 |
| 47 | 6 | 37400000 | 37600000 |
| 48 | 6 | 75900000 | 76300000 |
| 49 | 6 | 78700000 | 78900000 |
| 50 | 6 | 79900000 | 80400000 |
| 51 | 6 | 88500000 | 88800000 |
| 52 | 6 | 116100000 | 117100000 |
| 53 | 7 | 6500000 | 6800000 |
| 54 | 7 | 21300000 | 21500000 |
| 55 | 7 | 28400000 | 28600000 |
| 56 | 7 | 50000000 | 50300000 |
| 57 | 7 | 56400000 | 56600000 |
| 58 | 7 | 57100000 | 57300000 |
| 59 | 7 | 62900000 | 63100000 |
| 60 | 7 | 71300000 | 71500000 |
| 61 | 7 | 76900000 | 77100000 |
| 62 | 7 | 82300000 | 82700000 |
| 63 | 7 | 89300000 | 89600000 |
| 64 | 9 | 0 | 200000 |
| 65 | 9 | 20500000 | 20800000 |
| 66 | 9 | 30700000 | 31100000 |
| 67 | 9 | 59000000 | 59200000 |
| 68 | 9 | 68300000 | 68700000 |
| 69 | 10 | 7300000 | 7700000 |
| 70 | 10 | 26200000 | 26500000 |
| 71 | 10 | 29400000 | 29700000 |
| 72 | 10 | 31900000 | 32100000 |
| 73 | 10 | 35400000 | 35700000 |
| 74 | 10 | 37400000 | 38200000 |
| 75 | 10 | 42800000 | 43100000 |
| 76 | 10 | 64300000 | 64500000 |
| 77 | 11 | 26500000 | 27200000 |
| 78 | 11 | 28800000 | 29100000 |
| 79 | 11 | 34400000 | 34600000 |
| 80 | 11 | 36200000 | 36400000 |
| 81 | 11 | 45900001 | 46200000 |
| 82 | 11 | 55200001 | 55400000 |
| 83 | 12 | 41700000 | 41900000 |
| 84 | 12 | 42700000 | 43000000 |
| 85 | 12 | 49100000 | 49400000 |
| 86 | 12 | 53000000 | 53400000 |
| 87 | 12 | 78400000 | 79100000 |
| 88 | 13 | 23300000 | 23500000 |
| 89 | 13 | 49200000 | 49400000 |
| 90 | 13 | 49800000 | 50600000 |
| 91 | 13 | 51300000 | 51500000 |
| 92 | 13 | 53000000 | 53700000 |
| 93 | 13 | 56300000 | 56500000 |
| 94 | 13 | 63100001 | 63300000 |
| 95 | 14 | 10600000 | 10900000 |
| 96 | 15 | 2800000 | 3100000 |
| 97 | 15 | 3300000 | 3900000 |
| 98 | 15 | 40100000 | 40400000 |
| 99 | 15 | 60700000 | 60900000 |
| 100 | 15 | 80600000 | 80900000 |
| 101 | 16 | 3400000 | 3600000 |
| 102 | 16 | 33100000 | 33300000 |
| 103 | 16 | 40500000 | 40800000 |
| 104 | 16 | 43200000 | 43400000 |
| 105 | 16 | 70500000 | 71000000 |
| 106 | 16 | 71600000 | 71800000 |
| 107 | 17 | 44600000 | 44800000 |
| 108 | 17 | 52100000 | 52500000 |
| 109 | 17 | 53300000 | 53500000 |
| 110 | 17 | 61700001 | 61900000 |
| 111 | 17 | 70600001 | 70800000 |
| 112 | 18 | 4000001 | 4200000 |
| 113 | 18 | 5900000 | 6100000 |
| 114 | 18 | 22600000 | 22800000 |
| 115 | 18 | 23600000 | 23900000 |
| 116 | 18 | 32200001 | 32500000 |
| 117 | 18 | 45200000 | 45600000 |
| 118 | 18 | 66000000 | 66200000 |
| 119 | 19 | 21200000 | 21400000 |
| 120 | 20 | 15100000 | 15300000 |
| 121 | 20 | 17000000 | 17200000 |
| 122 | 20 | 25400001 | 25800000 |
| 123 | 20 | 34100000 | 34300000 |
| 124 | 20 | 50900001 | 51100000 |
| 125 | 20 | 49800000 | 50400000 |
| 126 | 21 | 41100000 | 41400000 |
| 127 | 21 | 49400000 | 49900000 |
| 128 | 22 | 22000001 | 22200000 |
| 129 | 23 | 44200001 | 44400000 |
| 130 | 24 | 10300000 | 10500000 |
| 131 | 24 | 34600000 | 34800000 |
| 132 | 24 | 36100000 | 36300000 |
| 133 | 24 | 41600000 | 42000000 |
| 134 | 26 | 32800001 | 33100000 |
| 135 | X | 32100000 | 32400000 |
| 136 | X | 44800000 | 45200000 |
| 137 | X | 52500000 | 52800000 |
| 138 | X | 56500000 | 58000000 |
| 139 | X | 66000000 | 66300000 |
| 140 | X | 67400000 | 67600000 |
| 141 | X | 68300000 | 68800000 |
| 142 | X | 70500000 | 70700000 |
| 143 | X | 93200000 | 93400000 |
