# Supplementary material for: Genome-wide analysis reveals signatures of selection for important traits in domestic sheep from different ecoregions
Source: BMC Genomics. 2016 Nov 3;17:863. doi: 10.1186/s12864-016-3212-2 (PMC5094087; doi:10.1186/s12864-016-3212-2)
Supplement: Additional file 19: Table S14. — Genomic regions under selection in Duolang sheep. (DOC 127 kb) [file 12864_2016_3212_MOESM19_ESM.doc]

**Additional file 19: Table S14. Genomic regions under selection in Duolang sheep.**

| Number | Chromosome | Start | End |
| --- | --- | --- | --- |
| 1 | 1 | 25000000 | 25300000 |
| 2 | 1 | 27700000 | 27900000 |
| 3 | 1 | 50800000 | 51000000 |
| 4 | 1 | 111200000 | 111400000 |
| 5 | 1 | 118000000 | 118700000 |
| 6 | 1 | 119200000 | 119700000 |
| 7 | 1 | 204700000 | 205200000 |
| 8 | 1 | 250500000 | 250900000 |
| 9 | 1 | 263800000 | 264100000 |
| 10 | 2 | 49600000 | 49900000 |
| 11 | 2 | 51200000 | 52100000 |
| 12 | 2 | 52100000 | 52900000 |
| 13 | 2 | 53600000 | 54800000 |
| 14 | 2 | 55800000 | 56000000 |
| 15 | 2 | 71000000 | 71300000 |
| 16 | 2 | 73600000 | 73800000 |
| 17 | 2 | 104100000 | 104900000 |
| 18 | 2 | 111700000 | 112600000 |
| 19 | 2 | 115100000 | 115300000 |
| 20 | 2 | 122500000 | 122700000 |
| 21 | 2 | 136500000 | 136700000 |
| 22 | 2 | 160800000 | 161000000 |
| 23 | 2 | 173200000 | 173400000 |
| 24 | 2 | 188000000 | 188200000 |
| 25 | 2 | 194600000 | 194800000 |
| 26 | 2 | 214200000 | 214500000 |
| 27 | 2 | 218400000 | 218700000 |
| 28 | 2 | 219400000 | 219700000 |
| 29 | 3 | 100000 | 200000 |
| 30 | 3 | 18200000 | 18400000 |
| 31 | 3 | 24200000 | 24500000 |
| 32 | 3 | 31200000 | 31400000 |
| 33 | 3 | 39300000 | 39600000 |
| 34 | 3 | 44800000 | 45200000 |
| 35 | 3 | 105400000 | 105600000 |
| 36 | 3 | 106800000 | 107100000 |
| 37 | 3 | 124800000 | 125000000 |
| 38 | 3 | 154200000 | 154600000 |
| 39 | 3 | 213200000 | 213500000 |
| 40 | 3 | 220300000 | 220600000 |
| 41 | 4 | 26300000 | 26500000 |
| 42 | 4 | 29200000 | 29400000 |
| 43 | 4 | 46200000 | 46400000 |
| 44 | 4 | 68700000 | 69000000 |
| 45 | 4 | 85600000 | 85800000 |
| 46 | 4 | 92600000 | 92900000 |
| 47 | 4 | 101200000 | 101900000 |
| 48 | 5 | 100000 | 200000 |
| 49 | 5 | 40900000 | 42000000 |
| 50 | 5 | 51400000 | 51700000 |
| 51 | 5 | 57800000 | 58100000 |
| 52 | 5 | 70800000 | 71000000 |
| 53 | 5 | 78400000 | 78600000 |
| 54 | 5 | 107100000 | 108000000 |
| 55 | 6 | 19400000 | 19600000 |
| 56 | 6 | 24700000 | 24900000 |
| 57 | 6 | 31000000 | 31200000 |
| 58 | 6 | 33000000 | 33200000 |
| 59 | 6 | 36200000 | 36500000 |
| 60 | 6 | 54600000 | 54800000 |
| 61 | 6 | 69900000 | 70600000 |
| 62 | 6 | 78800000 | 79300000 |
| 63 | 6 | 79900000 | 80400000 |
| 64 | 6 | 88500000 | 88800000 |
| 65 | 6 | 116100000 | 117000000 |
| 66 | 7 | 100000 | 200000 |
| 67 | 7 | 49200000 | 50400000 |
| 68 | 7 | 79000000 | 79200000 |
| 69 | 7 | 89300000 | 89500000 |
| 70 | 8 | 87700000 | 88000000 |
| 71 | 8 | 30700000 | 30900000 |
| 72 | 8 | 81400000 | 81600000 |
| 73 | 8 | 66600000 | 66800000 |
| 74 | 9 | 20500000 | 20800000 |
| 75 | 9 | 100000 | 200000 |
| 76 | 10 | 1300000 | 1500000 |
| 77 | 10 | 7300000 | 7600000 |
| 78 | 10 | 19100000 | 19300000 |
| 79 | 10 | 26200000 | 26500000 |
| 80 | 10 | 29500000 | 29700000 |
| 81 | 10 | 35400000 | 35700000 |
| 82 | 10 | 37400000 | 38100000 |
| 83 | 10 | 42800000 | 43100000 |
| 84 | 11 | 24500000 | 24800000 |
| 85 | 11 | 26500000 | 27700000 |
| 86 | 11 | 28800000 | 29100000 |
| 87 | 11 | 36200000 | 36400000 |
| 88 | 11 | 46000000 | 46200000 |
| 89 | 11 | 55200000 | 55400000 |
| 90 | 12 | 49100000 | 49400000 |
| 91 | 12 | 51800000 | 52000000 |
| 92 | 12 | 53000000 | 53400000 |
| 93 | 12 | 78300000 | 79100000 |
| 94 | 13 | 29600000 | 29800000 |
| 95 | 13 | 49200000 | 50600000 |
| 96 | 13 | 53000000 | 53600000 |
| 97 | 13 | 56300000 | 56500000 |
| 98 | 13 | 62300000 | 62500000 |
| 99 | 14 | 35500000 | 35700000 |
| 100 | 14 | 23700000 | 23900000 |
| 101 | 14 | 18500000 | 18700000 |
| 102 | 15 | 3300000 | 3900000 |
| 103 | 15 | 40200000 | 40400000 |
| 104 | 15 | 80600000 | 80900000 |
| 105 | 16 | 3400000 | 3600000 |
| 106 | 16 | 43200000 | 43400000 |
| 107 | 16 | 31800000 | 32000000 |
| 108 | 17 | 100000 | 300000 |
| 109 | 17 | 33000000 | 33200000 |
| 110 | 17 | 52100000 | 52300000 |
| 111 | 17 | 53500000 | 53800000 |
| 112 | 17 | 61700000 | 61900000 |
| 113 | 18 | 4000000 | 4200000 |
| 114 | 18 | 14900000 | 15100000 |
| 115 | 18 | 22600000 | 22800000 |
| 116 | 18 | 32200000 | 32500000 |
| 117 | 18 | 54100000 | 54300000 |
| 118 | 18 | 65900000 | 66100000 |
| 119 | 19 | 11500000 | 11700000 |
| 120 | 20 | 25400000 | 25800000 |
| 121 | 20 | 15100000 | 15400000 |
| 122 | 20 | 49800000 | 51100000 |
| 123 | 21 | 300000 | 600000 |
| 124 | 21 | 18000000 | 18600000 |
| 125 | 21 | 49600000 | 49900000 |
| 126 | 22 | 15300000 | 15500000 |
| 127 | 23 | 44200000 | 44400000 |
| 128 | 24 | 10400000 | 10700000 |
| 129 | 24 | 3500000 | 3700000 |
| 130 | 24 | 34600000 | 34800000 |
| 131 | 24 | 41600000 | 42000000 |
| 132 | 25 | 7400000 | 7600000 |
| 133 | 25 | 19700000 | 19900000 |
| 134 | 26 | 32800000 | 33100000 |
| 135 | X | 44800000 | 45300000 |
| 136 | X | 46100000 | 46400000 |
| 137 | X | 52500000 | 52800000 |
| 138 | X | 56400000 | 58000000 |
| 139 | X | 58800000 | 59000000 |
| 140 | X | 67400000 | 68800000 |
| 141 | X | 69200000 | 69700000 |
| 142 | X | 77300000 | 77700000 |
| 143 | X | 80100000 | 80300000 |
