# Supplementary material for: Genome-wide analysis reveals signatures of selection for important traits in domestic sheep from different ecoregions
Source: BMC Genomics. 2016 Nov 3;17:863. doi: 10.1186/s12864-016-3212-2 (PMC5094087; doi:10.1186/s12864-016-3212-2)
Supplement: Additional file 20: Table S15. — Enriched GO terms among genes located in the selection regions with Z(F ST)M-S > 4 and Z(F ST)M-D > 4. (DOC 166 kb) [file 12864_2016_3212_MOESM20_ESM.doc]

**Additional file 20: Table S15.** Enriched GO terms among genes located in the selection regions with Z(*F*_ST_)_M-S_ > 4 and Z(*F*_ST_)_M-D_ > 4.

| GO term | Gene count | P value | genes |
| --- | --- | --- | --- |
| multicellular organismal development; | 47#2299 | 1.10E-06 | tp53;dvl2;chrna4;cdkn2c;cspg4;hoxa6;alox12b;elf3;hras;sox15;hoxa10;hoxa5;fgf11;stk36;tcf3;hoxa3;tnfsf12;mab21l1;cux1;hoxa4;hoxa1;stmn3;dazap1;kctd11;myt1;maea;hoxa13;dusp22;nav1;evx1;dvl1;gamt;foxf2;adra1d;alox15b;spon2;odf3;kcnq2;letm1;efnb3;fgfr3;irf4;sod1;dlg4;hoxa2;otx1;nlgn2 |
| developmental process; | 61#3347 | 1.10E-06 | faf1;tp53;eef1a2;dvl2;deaf1;chrna4;oca2;cdkn2c;cspg4;hoxa6;alox12b;alox12;elf3;hras;sox15;tbx19;hoxa10;hoxa5;fgf11;eif5a;stk36;tcf3;hoxa3;tnfsf12;mab21l1;cux1;hoxa4;hoxa1;stmn3;dazap1;birc7;kctd11;myt1;rqcd1;maea;hoxa13;tnfrsf18;dusp22;nav1;fgfrl1;evx1;dvl1;gamt;foxf2;adra1d;alox15b;ctsb;spon2;spag16;odf3;kcnq2;letm1;rtel1;irf4;efnb3;fgfr3;sod1;dlg4;hoxa2;otx1;nlgn2 |
| anatomical structure development; | 42#2005 | 1.69E-06 | tp53;dvl2;deaf1;chrna4;cdkn2c;cspg4;alox12b;alox12;elf3;hras;sox15;tbx19;hoxa5;fgf11;tcf3;tnfsf12;mab21l1;cux1;hoxa4;stmn3;kctd11;myt1;maea;hoxa13;fgfrl1;nav1;dvl1;gamt;foxf2;adra1d;alox15b;spon2;kcnq2;spag16;fgfr3;efnb3;irf4;sod1;dlg4;hoxa2;otx1;nlgn2 |
| multicellular organismal process; | 66#3822 | 1.69E-06 | gucy2d;dvl2;hoxa6;chrnb1;hras;sox15;hoxa5;hoxa3;tnfsf12;hoxa4;hoxa1;dazap1;serpinc1;evx1;adra1d;oprl1;alox15b;spon2;camk4;letm1;sod1;otx1;gabarap;nlgn2;scnn1d;akr1d1;tp53;chrna4;cdkn2c;cspg4;alox12b;glra3;sh2b2;elf3;hoxa10;tas1r3;cfd;fgf11;stk36;tcf3;wdr36;cplx1;tnni1;tnnt2;cux1;mab21l1;stmn3;kctd11;myt1;maea;hoxa13;gabrg3;dusp22;nav1;bcs1l;dvl1;gamt;foxf2;npbwr2;kcnq2;odf3;fgfr3;efnb3;irf4;dlg4;hoxa2 |
| anatomical structure morphogenesis; | 27#1047 | 1.69E-06 | kctd11;tp53;dvl2;deaf1;cdkn2c;cspg4;fgfrl1;alox12;dvl1;gamt;foxf2;elf3;adra1d;hras;alox15b;sox15;tbx19;spon2;spag16;efnb3;fgfr3;sod1;hoxa2;otx1;tnfsf12;mab21l1;hoxa4 |
| multicellular organismal development#system development; | 33#1605 | 0.000108292 | dvl2;chrna4;cdkn2c;cspg4;alox12b;elf3;hras;sox15;hoxa5;fgf11;tcf3;tnfsf12;cux1;stmn3;kctd11;myt1;maea;hoxa13;nav1;dvl1;gamt;foxf2;alox15b;spon2;kcnq2;fgfr3;efnb3;irf4;sod1;dlg4;hoxa2;otx1;nlgn2 |
| leukotriene biosynthetic process; | 5#22 | 0.000163798 | aloxe3;alox12;rnpep;alox15b;alox12b |
| alkene biosynthetic process; | 5#22 | 0.000163798 | aloxe3;alox12;rnpep;alox15b;alox12b |
| negative regulation of biological process; | 26#1182 | 0.000243879 | kctd11;faf1;tp53;eef1a2;cdkn2c;aurkaip1;tnfrsf18;rfx3;kiss1r;alox12;foxf2;adra1d;alox15b;sox15;stk11;irf7;rtel1;sod1;vil1;sin3a;ctbp1;cux1;rgs19;sirt3;bcl6b;birc7 |
| negative regulation of cellular process; | 25#1137 | 0.000354881 | kctd11;faf1;tp53;eef1a2;cdkn2c;aurkaip1;tnfrsf18;rfx3;kiss1r;alox12;foxf2;alox15b;sox15;stk11;irf7;rtel1;sod1;vil1;sin3a;cux1;ctbp1;rgs19;sirt3;bcl6b;birc7 |
| transcription from RNA polymerase II promoter; | 17#640 | 0.000354881 | sox15;tbx19;tp53;deaf1;chd3;irf7;prpf6;polr2a;med16;ybx2;otx1;polr2e;cux1;foxf2;gmeb2;bcl6b;elf3 |
| cell-cell signaling; | 17#640 | 0.000354881 | chrna4;gabrg3;glra3;chrnb1;adra1d;npbwr2;camk4;efna2;fgf11;grb2;kcnq2;efnb3;pdgfa;sod1;cplx1;dlg4;gabarap |
| leukotriene metabolic process; | 5#31 | 0.000565716 | aloxe3;alox12;rnpep;alox15b;alox12b |
| alkene metabolic process; | 5#31 | 0.000565716 | aloxe3;alox12;rnpep;alox15b;alox12b |
| carboxylic acid biosynthetic process; | 7#83 | 0.000621942 | aloxe3;akr1d1;alox12;rnpep;prkag3;alox15b;alox12b |
| organic acid biosynthetic process; | 7#83 | 0.000621942 | aloxe3;akr1d1;alox12;rnpep;prkag3;alox15b;alox12b |
| icosanoid biosynthetic process; | 5#33 | 0.000624751 | aloxe3;alox12;rnpep;alox15b;alox12b |
| cellular component organization and biogenesis; | 52#3277 | 0.000624751 | faf1;tp53;erp29;cdkn2c;ict1;grin3b;bet1l;gga3;alox12;sh2b2;elf3;trappc1;sox15;apc2;eif5a;cntrob;wdr36;tnnt2;cplx1;herc2;vil1;asgr2;cnn2;birc7;kctd11;asgr1;med16;abca7;adam2;fgfrl1;bcs1l;ndufs7;vps26b;denr;foxf2;clec10a;spon2;chd3;camk4;spag16;prpf6;snupn;klhl20;efnb3;fgfr3;sod1;dlg4;ipo9;gabarap;exoc2;nlgn2;sirt3 |
| fatty acid biosynthetic process; | 6#73 | 0.002671045 | aloxe3;alox12;rnpep;prkag3;alox15b;alox12b |
| biological regulation; | 88#6731 | 0.002765758 | faf1;arid3a;aurkaip1;ube2j2;nkx1-2;hoxa6;chrnb1;alox12;rassf7;sox15;tbx19;hoxa5;stk11;eif5a;polr2a;ybx2;hoxa3;zbtb37;tnfsf12;ctbp1;hoxa4;hoxa1;birc7;serpinc1;slc2a4;med16;arfgap1;evx1;kiss1r;gmeb2;adra1d;oprl1;alox15b;chd3;irf7;rtel1;sod1;znf142;otx1;sirt3;bcl6b;rexo1;acad8;zbtb46;ube2t;tp53;eef1a2;deaf1;cdkn2c;grin3b;creb3l2;dgkq;sh2b2;znf512b;elf3;gps2;hoxa10;cfd;stk36;mbd3;tcf3;tnnt2;tnni1;tcea2;vil1;pcgf3;uncx;ccnl2;sin3a;cux1;kctd11;myt1;hoxa13;tnfrsf18;dusp22;zbtb4;fgfrl1;rfx3;gamt;foxf2;ctsb;prpf6;fgfr3;irf4;pdgfa;mib2;hoxa2;rgs19 |
| icosanoid metabolic process; | 5#50 | 0.004098518 | aloxe3;alox12;rnpep;alox15b;alox12b |
| regulation of biological process; | 80#6140 | 0.007715708 | faf1;arid3a;aurkaip1;ube2j2;nkx1-2;hoxa6;alox12;rassf7;sox15;tbx19;hoxa5;stk11;eif5a;polr2a;ybx2;hoxa3;zbtb37;tnfsf12;ctbp1;hoxa4;hoxa1;birc7;med16;arfgap1;evx1;kiss1r;gmeb2;adra1d;alox15b;chd3;irf7;rtel1;sod1;znf142;otx1;sirt3;bcl6b;rexo1;acad8;zbtb46;ube2t;tp53;eef1a2;deaf1;cdkn2c;grin3b;creb3l2;sh2b2;znf512b;elf3;hoxa10;cfd;mbd3;stk36;tcf3;tnnt2;tnni1;tcea2;vil1;pcgf3;uncx;ccnl2;sin3a;cux1;kctd11;myt1;hoxa13;tnfrsf18;zbtb4;fgfrl1;rfx3;gamt;foxf2;ctsb;prpf6;irf4;pdgfa;mib2;hoxa2;rgs19 |
| neurological system process#transmission of nerve impulse; | 11#330 | 0.009018628 | npbwr2;chrna4;camk4;kcnq2;gabrg3;sod1;cplx1;dlg4;glra3;gabarap;chrnb1 |
| lipid biosynthetic process; | 11#333 | 0.009342192 | aloxe3;akr1d1;ptdss2;rnpep;sod1;prkag3;alox12b;fdft1;alox12;stard4;alox15b |
| neurological system process#transmission of nerve impulse#synaptic transmission; | 10#290 | 0.012319221 | npbwr2;chrna4;camk4;kcnq2;gabrg3;cplx1;dlg4;glra3;gabarap;chrnb1 |
| fatty acid metabolic process; | 8#191 | 0.013267786 | aloxe3;hao1;alox12;rnpep;prkag3;alox15b;alox12b;acadvl |
| growth#regulation of growth; | 8#201 | 0.01785929 | kctd11;fgfrl1;tp53;alox12;gamt;cdkn2c;sod1;alox15b |
| metabolic process#regulation of metabolic process; | 57#4150 | 0.01785929 | zbtb46;faf1;arid3a;ube2t;tp53;deaf1;cdkn2c;aurkaip1;ube2j2;nkx1-2;hoxa6;creb3l2;sh2b2;znf512b;elf3;rassf7;sox15;tbx19;hoxa10;hoxa5;eif5a;stk36;mbd3;tcf3;polr2a;ybx2;tcea2;vil1;pcgf3;hoxa3;uncx;sin3a;ccnl2;zbtb37;cux1;hoxa4;hoxa1;myt1;hoxa13;med16;zbtb4;rfx3;evx1;foxf2;gmeb2;chd3;irf7;prpf6;irf4;sod1;znf142;hoxa2;otx1;sirt3;bcl6b;rexo1;acad8 |
| ear development; | 4#42 | 0.01785929 | hoxa2;otx1;cux1;sod1 |
| cellular developmental process; | 30#1810 | 0.01785929 | kctd11;faf1;myt1;tp53;maea;eef1a2;deaf1;cdkn2c;tnfrsf18;cspg4;dusp22;nav1;alox12;elf3;alox15b;sox15;ctsb;spon2;eif5a;odf3;tcf3;rtel1;efnb3;irf4;sod1;hoxa2;tnfsf12;cux1;dazap1;birc7 |
| cell differentiation; | 30#1810 | 0.01785929 | kctd11;faf1;myt1;tp53;maea;eef1a2;deaf1;cdkn2c;tnfrsf18;cspg4;dusp22;nav1;alox12;elf3;alox15b;sox15;ctsb;spon2;eif5a;odf3;tcf3;rtel1;efnb3;irf4;sod1;hoxa2;tnfsf12;cux1;dazap1;birc7 |
| sexual reproduction; | 10#315 | 0.018380062 | hoxa10;deaf1;odf3;ybx2;sod1;adam2;abcb9;adam29;gamt;dazap1 |
| gamete generation; | 9#264 | 0.019657528 | hoxa10;deaf1;odf3;ybx2;sod1;abcb9;adam29;gamt;dazap1 |
| spermatogenesis; | 8#212 | 0.019657528 | hoxa10;odf3;ybx2;sod1;abcb9;adam29;gamt;dazap1 |
| male gamete generation; | 8#212 | 0.019657528 | hoxa10;odf3;ybx2;sod1;abcb9;adam29;gamt;dazap1 |
| cellular process#regulation of cellular process; | 73#5704 | 0.019833842 | faf1;arid3a;aurkaip1;nkx1-2;hoxa6;alox12;rassf7;sox15;tbx19;hoxa5;stk11;eif5a;polr2a;ybx2;hoxa3;zbtb37;tnfsf12;ctbp1;hoxa4;hoxa1;birc7;med16;arfgap1;evx1;kiss1r;gmeb2;adra1d;alox15b;chd3;irf7;rtel1;sod1;znf142;otx1;sirt3;bcl6b;rexo1;acad8;zbtb46;tp53;eef1a2;deaf1;cdkn2c;grin3b;creb3l2;znf512b;elf3;hoxa10;mbd3;stk36;tcf3;tcea2;vil1;pcgf3;uncx;ccnl2;sin3a;cux1;kctd11;myt1;hoxa13;tnfrsf18;zbtb4;fgfrl1;rfx3;foxf2;ctsb;prpf6;irf4;pdgfa;mib2;hoxa2;rgs19 |
| multicellular organismal development#system development#organ development; | 21#1141 | 0.024221439 | maea;dvl2;hoxa13;cspg4;alox12b;dvl1;gamt;foxf2;elf3;hras;alox15b;sox15;hoxa5;tcf3;fgfr3;irf4;sod1;hoxa2;otx1;tnfsf12;cux1 |
| nervous system development; | 15#716 | 0.028360456 | kctd11;myt1;spon2;fgf11;dvl2;chrna4;kcnq2;cdkn2c;efnb3;sod1;nav1;dlg4;otx1;nlgn2;stmn3 |
| primary metabolic process; | 141#12764 | 0.028573776 | idua;faf1;gucy2d;erp29;dnajc5;ict1;ube2j2;hoxa6;neil1;dars2;nadk;sox15;man2c1;gmds;stk11;polr2a;ogfod2;ybx2;adam29;ctbp1;hoxa4;b3galt6;tspan8;mmp23b;ttll10;gmeb2;adra1d;alox15b;chd3;irf7;znf142;sirt3;tp53;deaf1;pcsk4;ddi1;znf512b;aloxe3;ptk6;ptpn9;cfd;stk36;mrpl20;tcf3;wdr36;herc2;sin3a;ccnl2;eif4a1;glb1l3;mif4gd;adam32;hoxa13;midn;dusp22;adam2;atp5h;ndufs7;prdx6;foxf2;ptdss2;prpf6;klhl20;hoxa2;mpdu1;arid3a;aurkaip1;gak;nkx1-2;rnf25;alox12;gga3;pusl1;rassf7;tbx19;b3gat1;hoxa5;eif5a;prkag3;ptpn7;hoxa3;fdft1;zbtb37;hoxa1;mapkapk5;birc7;slc2a4;ssu72;htra4;med16;evx1;usp37;athl1;ttll4;camk4;sod1;ipo9;otx1;csnk1g2;bcl6b;acad8;rexo1;zbtb46;ube2t;akr1d1;eef1a2;atp5d;alox12b;acadvl;creb3l2;elf3;hoxa10;apc2;tnk1;mbd3;tcea2;vil1;pcgf3;uncx;adam9;cux1;srms;stard4;mrps7;myt1;nt5c;rnpep;slbp;zbtb4;rfx3;bcs1l;hao1;polr2e;gamt;denr;ctsb;fgfr3;irf4;plcd4;mib2;dlg4 |
| transcription, DNA-dependent; | 48#3439 | 0.028573776 | zbtb46;arid3a;tp53;deaf1;nkx1-2;hoxa6;creb3l2;znf512b;elf3;rassf7;sox15;tbx19;hoxa10;hoxa5;mbd3;tcf3;polr2a;ybx2;tcea2;pcgf3;hoxa3;uncx;sin3a;ccnl2;zbtb37;cux1;hoxa4;hoxa1;myt1;hoxa13;med16;zbtb4;rfx3;evx1;polr2e;foxf2;gmeb2;chd3;irf7;prpf6;irf4;znf142;hoxa2;otx1;sirt3;bcl6b;acad8;rexo1 |
| RNA biosynthetic process; | 48#3444 | 0.028573776 | zbtb46;arid3a;tp53;deaf1;nkx1-2;hoxa6;creb3l2;znf512b;elf3;rassf7;sox15;tbx19;hoxa10;hoxa5;mbd3;tcf3;polr2a;ybx2;tcea2;pcgf3;hoxa3;uncx;sin3a;ccnl2;zbtb37;cux1;hoxa4;hoxa1;myt1;hoxa13;med16;zbtb4;rfx3;evx1;polr2e;foxf2;gmeb2;chd3;irf7;prpf6;irf4;znf142;hoxa2;otx1;sirt3;bcl6b;acad8;rexo1 |
| monocarboxylic acid metabolic process; | 9#287 | 0.028573776 | aloxe3;akr1d1;rnpep;prkag3;alox12b;acadvl;alox12;hao1;alox15b |
| regulation of transcription, DNA-dependent; | 47#3358 | 0.028736401 | zbtb46;arid3a;tp53;deaf1;nkx1-2;hoxa6;creb3l2;znf512b;elf3;rassf7;sox15;tbx19;hoxa10;hoxa5;mbd3;tcf3;polr2a;ybx2;tcea2;pcgf3;hoxa3;uncx;sin3a;ccnl2;zbtb37;cux1;hoxa4;hoxa1;myt1;hoxa13;med16;zbtb4;rfx3;evx1;foxf2;gmeb2;chd3;irf7;prpf6;irf4;znf142;hoxa2;otx1;sirt3;bcl6b;acad8;rexo1 |
| growth%multicellular organism growth; | 3#24 | 0.029417067 | hoxa5;gamt;sod1 |
| localization; | 59#4481 | 0.030779852 | faf1;tp53;erp29;chrna4;oca2;cspg4;slc11a1;grin3b;col20a1;atp5d;mfsd7;bet1l;glra3;gga3;chrnb1;alox12;trappc1;ptpn9;kcnab3;atp1b2;tnnt2;slc16a13;cplx1;herc2;asgr2;tnfsf12;stard4;kctd11;atp8b3;sft2d2;slc2a4;slc25a19;slc16a5;asgr1;gabrg3;abca7;arfgap1;dnah2;shbg;atp5h;kiss1r;vps37b;vps26b;alox15b;clec10a;pitpnm2;spon2;kctd2;camk4;kcnq2;snupn;slc16a11;efnb3;abcb9;gabarap;ipo9;exoc2;scnn1d;scamp4 |
| coenzyme A biosynthetic process; | 2#7 | 0.036975647 | pank2;ppcdc |
| negative regulation of metabolic process; | 11#436 | 0.043383909 | sox15;irf7;cdkn2c;sod1;vil1;rfx3;sin3a;cux1;foxf2;bcl6b;sirt3 |
| fatty acid oxidation; | 3#29 | 0.047116737 | hao1;alox12;acadvl |
| negative regulation of cellular metabolic process; | 10#381 | 0.049533677 | sox15;irf7;cdkn2c;sod1;rfx3;sin3a;cux1;foxf2;bcl6b;sirt3 |
| negative regulation of transcription, DNA-dependent; | 7#203 | 0.049533677 | sox15;sin3a;cux1;irf7;foxf2;sirt3;bcl6b |
| morphogenesis of an epithelium; | 4#63 | 0.049882954 | dvl2;foxf2;elf3;alox15b |
| ear development#ear morphogenesis; | 3#31 | 0.051968194 | hoxa2;otx1;sod1 |
| multicellular organismal development; | 47#2299 | 1.10E-06 | tp53;dvl2;chrna4;cdkn2c;cspg4;hoxa6;alox12b;elf3;hras;sox15;hoxa10;hoxa5;fgf11;stk36;tcf3;hoxa3;tnfsf12;mab21l1;cux1;hoxa4;hoxa1;stmn3;dazap1;kctd11;myt1;maea;hoxa13;dusp22;nav1;evx1;dvl1;gamt;foxf2;adra1d;alox15b;spon2;odf3;kcnq2;letm1;efnb3;fgfr3;irf4;sod1;dlg4;hoxa2;otx1;nlgn2 |
| developmental process; | 61#3347 | 1.10E-06 | faf1;tp53;eef1a2;dvl2;deaf1;chrna4;oca2;cdkn2c;cspg4;hoxa6;alox12b;alox12;elf3;hras;sox15;tbx19;hoxa10;hoxa5;fgf11;eif5a;stk36;tcf3;hoxa3;tnfsf12;mab21l1;cux1;hoxa4;hoxa1;stmn3;dazap1;birc7;kctd11;myt1;rqcd1;maea;hoxa13;tnfrsf18;dusp22;nav1;fgfrl1;evx1;dvl1;gamt;foxf2;adra1d;alox15b;ctsb;spon2;spag16;odf3;kcnq2;letm1;rtel1;irf4;efnb3;fgfr3;sod1;dlg4;hoxa2;otx1;nlgn2 |
| anatomical structure development; | 42#2005 | 1.69E-06 | tp53;dvl2;deaf1;chrna4;cdkn2c;cspg4;alox12b;alox12;elf3;hras;sox15;tbx19;hoxa5;fgf11;tcf3;tnfsf12;mab21l1;cux1;hoxa4;stmn3;kctd11;myt1;maea;hoxa13;fgfrl1;nav1;dvl1;gamt;foxf2;adra1d;alox15b;spon2;kcnq2;spag16;fgfr3;efnb3;irf4;sod1;dlg4;hoxa2;otx1;nlgn2 |
| multicellular organismal process; | 66#3822 | 1.69E-06 | gucy2d;dvl2;hoxa6;chrnb1;hras;sox15;hoxa5;hoxa3;tnfsf12;hoxa4;hoxa1;dazap1;serpinc1;evx1;adra1d;oprl1;alox15b;spon2;camk4;letm1;sod1;otx1;gabarap;nlgn2;scnn1d;akr1d1;tp53;chrna4;cdkn2c;cspg4;alox12b;glra3;sh2b2;elf3;hoxa10;tas1r3;cfd;fgf11;stk36;tcf3;wdr36;cplx1;tnni1;tnnt2;cux1;mab21l1;stmn3;kctd11;myt1;maea;hoxa13;gabrg3;dusp22;nav1;bcs1l;dvl1;gamt;foxf2;npbwr2;kcnq2;odf3;fgfr3;efnb3;irf4;dlg4;hoxa2 |
| anatomical structure morphogenesis; | 27#1047 | 1.69E-06 | kctd11;tp53;dvl2;deaf1;cdkn2c;cspg4;fgfrl1;alox12;dvl1;gamt;foxf2;elf3;adra1d;hras;alox15b;sox15;tbx19;spon2;spag16;efnb3;fgfr3;sod1;hoxa2;otx1;tnfsf12;mab21l1;hoxa4 |
| multicellular organismal development#system development; | 33#1605 | 0.000108292 | dvl2;chrna4;cdkn2c;cspg4;alox12b;elf3;hras;sox15;hoxa5;fgf11;tcf3;tnfsf12;cux1;stmn3;kctd11;myt1;maea;hoxa13;nav1;dvl1;gamt;foxf2;alox15b;spon2;kcnq2;fgfr3;efnb3;irf4;sod1;dlg4;hoxa2;otx1;nlgn2 |
| leukotriene biosynthetic process; | 5#22 | 0.000163798 | aloxe3;alox12;rnpep;alox15b;alox12b |
| alkene biosynthetic process; | 5#22 | 0.000163798 | aloxe3;alox12;rnpep;alox15b;alox12b |
| negative regulation of biological process; | 26#1182 | 0.000243879 | kctd11;faf1;tp53;eef1a2;cdkn2c;aurkaip1;tnfrsf18;rfx3;kiss1r;alox12;foxf2;adra1d;alox15b;sox15;stk11;irf7;rtel1;sod1;vil1;sin3a;ctbp1;cux1;rgs19;sirt3;bcl6b;birc7 |
| negative regulation of cellular process; | 25#1137 | 0.000354881 | kctd11;faf1;tp53;eef1a2;cdkn2c;aurkaip1;tnfrsf18;rfx3;kiss1r;alox12;foxf2;alox15b;sox15;stk11;irf7;rtel1;sod1;vil1;sin3a;cux1;ctbp1;rgs19;sirt3;bcl6b;birc7 |
| transcription from RNA polymerase II promoter; | 17#640 | 0.000354881 | sox15;tbx19;tp53;deaf1;chd3;irf7;prpf6;polr2a;med16;ybx2;otx1;polr2e;cux1;foxf2;gmeb2;bcl6b;elf3 |
| cell-cell signaling; | 17#640 | 0.000354881 | chrna4;gabrg3;glra3;chrnb1;adra1d;npbwr2;camk4;efna2;fgf11;grb2;kcnq2;efnb3;pdgfa;sod1;cplx1;dlg4;gabarap |
| leukotriene metabolic process; | 5#31 | 0.000565716 | aloxe3;alox12;rnpep;alox15b;alox12b |
| alkene metabolic process; | 5#31 | 0.000565716 | aloxe3;alox12;rnpep;alox15b;alox12b |
| carboxylic acid biosynthetic process; | 7#83 | 0.000621942 | aloxe3;akr1d1;alox12;rnpep;prkag3;alox15b;alox12b |
| organic acid biosynthetic process; | 7#83 | 0.000621942 | aloxe3;akr1d1;alox12;rnpep;prkag3;alox15b;alox12b |
| icosanoid biosynthetic process; | 5#33 | 0.000624751 | aloxe3;alox12;rnpep;alox15b;alox12b |
| cellular component organization and biogenesis; | 52#3277 | 0.000624751 | faf1;tp53;erp29;cdkn2c;ict1;grin3b;bet1l;gga3;alox12;sh2b2;elf3;trappc1;sox15;apc2;eif5a;cntrob;wdr36;tnnt2;cplx1;herc2;vil1;asgr2;cnn2;birc7;kctd11;asgr1;med16;abca7;adam2;fgfrl1;bcs1l;ndufs7;vps26b;denr;foxf2;clec10a;spon2;chd3;camk4;spag16;prpf6;snupn;klhl20;efnb3;fgfr3;sod1;dlg4;ipo9;gabarap;exoc2;nlgn2;sirt3 |
| fatty acid biosynthetic process; | 6#73 | 0.002671045 | aloxe3;alox12;rnpep;prkag3;alox15b;alox12b |
| biological regulation; | 88#6731 | 0.002765758 | faf1;arid3a;aurkaip1;ube2j2;nkx1-2;hoxa6;chrnb1;alox12;rassf7;sox15;tbx19;hoxa5;stk11;eif5a;polr2a;ybx2;hoxa3;zbtb37;tnfsf12;ctbp1;hoxa4;hoxa1;birc7;serpinc1;slc2a4;med16;arfgap1;evx1;kiss1r;gmeb2;adra1d;oprl1;alox15b;chd3;irf7;rtel1;sod1;znf142;otx1;sirt3;bcl6b;rexo1;acad8;zbtb46;ube2t;tp53;eef1a2;deaf1;cdkn2c;grin3b;creb3l2;dgkq;sh2b2;znf512b;elf3;gps2;hoxa10;cfd;stk36;mbd3;tcf3;tnnt2;tnni1;tcea2;vil1;pcgf3;uncx;ccnl2;sin3a;cux1;kctd11;myt1;hoxa13;tnfrsf18;dusp22;zbtb4;fgfrl1;rfx3;gamt;foxf2;ctsb;prpf6;fgfr3;irf4;pdgfa;mib2;hoxa2;rgs19 |
| icosanoid metabolic process; | 5#50 | 0.004098518 | aloxe3;alox12;rnpep;alox15b;alox12b |
| regulation of biological process; | 80#6140 | 0.007715708 | faf1;arid3a;aurkaip1;ube2j2;nkx1-2;hoxa6;alox12;rassf7;sox15;tbx19;hoxa5;stk11;eif5a;polr2a;ybx2;hoxa3;zbtb37;tnfsf12;ctbp1;hoxa4;hoxa1;birc7;med16;arfgap1;evx1;kiss1r;gmeb2;adra1d;alox15b;chd3;irf7;rtel1;sod1;znf142;otx1;sirt3;bcl6b;rexo1;acad8;zbtb46;ube2t;tp53;eef1a2;deaf1;cdkn2c;grin3b;creb3l2;sh2b2;znf512b;elf3;hoxa10;cfd;mbd3;stk36;tcf3;tnnt2;tnni1;tcea2;vil1;pcgf3;uncx;ccnl2;sin3a;cux1;kctd11;myt1;hoxa13;tnfrsf18;zbtb4;fgfrl1;rfx3;gamt;foxf2;ctsb;prpf6;irf4;pdgfa;mib2;hoxa2;rgs19 |
| neurological system process#transmission of nerve impulse; | 11#330 | 0.009018628 | npbwr2;chrna4;camk4;kcnq2;gabrg3;sod1;cplx1;dlg4;glra3;gabarap;chrnb1 |
| lipid biosynthetic process; | 11#333 | 0.009342192 | aloxe3;akr1d1;ptdss2;rnpep;sod1;prkag3;alox12b;fdft1;alox12;stard4;alox15b |
| neurological system process#transmission of nerve impulse#synaptic transmission; | 10#290 | 0.012319221 | npbwr2;chrna4;camk4;kcnq2;gabrg3;cplx1;dlg4;glra3;gabarap;chrnb1 |
| fatty acid metabolic process; | 8#191 | 0.013267786 | aloxe3;hao1;alox12;rnpep;prkag3;alox15b;alox12b;acadvl |
| growth#regulation of growth; | 8#201 | 0.01785929 | kctd11;fgfrl1;tp53;alox12;gamt;cdkn2c;sod1;alox15b |
| metabolic process#regulation of metabolic process; | 57#4150 | 0.01785929 | zbtb46;faf1;arid3a;ube2t;tp53;deaf1;cdkn2c;aurkaip1;ube2j2;nkx1-2;hoxa6;creb3l2;sh2b2;znf512b;elf3;rassf7;sox15;tbx19;hoxa10;hoxa5;eif5a;stk36;mbd3;tcf3;polr2a;ybx2;tcea2;vil1;pcgf3;hoxa3;uncx;sin3a;ccnl2;zbtb37;cux1;hoxa4;hoxa1;myt1;hoxa13;med16;zbtb4;rfx3;evx1;foxf2;gmeb2;chd3;irf7;prpf6;irf4;sod1;znf142;hoxa2;otx1;sirt3;bcl6b;rexo1;acad8 |
| ear development; | 4#42 | 0.01785929 | hoxa2;otx1;cux1;sod1 |
| cellular developmental process; | 30#1810 | 0.01785929 | kctd11;faf1;myt1;tp53;maea;eef1a2;deaf1;cdkn2c;tnfrsf18;cspg4;dusp22;nav1;alox12;elf3;alox15b;sox15;ctsb;spon2;eif5a;odf3;tcf3;rtel1;efnb3;irf4;sod1;hoxa2;tnfsf12;cux1;dazap1;birc7 |
| cell differentiation; | 30#1810 | 0.01785929 | kctd11;faf1;myt1;tp53;maea;eef1a2;deaf1;cdkn2c;tnfrsf18;cspg4;dusp22;nav1;alox12;elf3;alox15b;sox15;ctsb;spon2;eif5a;odf3;tcf3;rtel1;efnb3;irf4;sod1;hoxa2;tnfsf12;cux1;dazap1;birc7 |
| sexual reproduction; | 10#315 | 0.018380062 | hoxa10;deaf1;odf3;ybx2;sod1;adam2;abcb9;adam29;gamt;dazap1 |
| gamete generation; | 9#264 | 0.019657528 | hoxa10;deaf1;odf3;ybx2;sod1;abcb9;adam29;gamt;dazap1 |
| spermatogenesis; | 8#212 | 0.019657528 | hoxa10;odf3;ybx2;sod1;abcb9;adam29;gamt;dazap1 |
| male gamete generation; | 8#212 | 0.019657528 | hoxa10;odf3;ybx2;sod1;abcb9;adam29;gamt;dazap1 |
| cellular process#regulation of cellular process; | 73#5704 | 0.019833842 | faf1;arid3a;aurkaip1;nkx1-2;hoxa6;alox12;rassf7;sox15;tbx19;hoxa5;stk11;eif5a;polr2a;ybx2;hoxa3;zbtb37;tnfsf12;ctbp1;hoxa4;hoxa1;birc7;med16;arfgap1;evx1;kiss1r;gmeb2;adra1d;alox15b;chd3;irf7;rtel1;sod1;znf142;otx1;sirt3;bcl6b;rexo1;acad8;zbtb46;tp53;eef1a2;deaf1;cdkn2c;grin3b;creb3l2;znf512b;elf3;hoxa10;mbd3;stk36;tcf3;tcea2;vil1;pcgf3;uncx;ccnl2;sin3a;cux1;kctd11;myt1;hoxa13;tnfrsf18;zbtb4;fgfrl1;rfx3;foxf2;ctsb;prpf6;irf4;pdgfa;mib2;hoxa2;rgs19 |
| multicellular organismal development#system development#organ development; | 21#1141 | 0.024221439 | maea;dvl2;hoxa13;cspg4;alox12b;dvl1;gamt;foxf2;elf3;hras;alox15b;sox15;hoxa5;tcf3;fgfr3;irf4;sod1;hoxa2;otx1;tnfsf12;cux1 |
| nervous system development; | 15#716 | 0.028360456 | kctd11;myt1;spon2;fgf11;dvl2;chrna4;kcnq2;cdkn2c;efnb3;sod1;nav1;dlg4;otx1;nlgn2;stmn3 |
| primary metabolic process; | 141#12764 | 0.028573776 | idua;faf1;gucy2d;erp29;dnajc5;ict1;ube2j2;hoxa6;neil1;dars2;nadk;sox15;man2c1;gmds;stk11;polr2a;ogfod2;ybx2;adam29;ctbp1;hoxa4;b3galt6;tspan8;mmp23b;ttll10;gmeb2;adra1d;alox15b;chd3;irf7;znf142;sirt3;tp53;deaf1;pcsk4;ddi1;znf512b;aloxe3;ptk6;ptpn9;cfd;stk36;mrpl20;tcf3;wdr36;herc2;sin3a;ccnl2;eif4a1;glb1l3;mif4gd;adam32;hoxa13;midn;dusp22;adam2;atp5h;ndufs7;prdx6;foxf2;ptdss2;prpf6;klhl20;hoxa2;mpdu1;arid3a;aurkaip1;gak;nkx1-2;rnf25;alox12;gga3;pusl1;rassf7;tbx19;b3gat1;hoxa5;eif5a;prkag3;ptpn7;hoxa3;fdft1;zbtb37;hoxa1;mapkapk5;birc7;slc2a4;ssu72;htra4;med16;evx1;usp37;athl1;ttll4;camk4;sod1;ipo9;otx1;csnk1g2;bcl6b;acad8;rexo1;zbtb46;ube2t;akr1d1;eef1a2;atp5d;alox12b;acadvl;creb3l2;elf3;hoxa10;apc2;tnk1;mbd3;tcea2;vil1;pcgf3;uncx;adam9;cux1;srms;stard4;mrps7;myt1;nt5c;rnpep;slbp;zbtb4;rfx3;bcs1l;hao1;polr2e;gamt;denr;ctsb;fgfr3;irf4;plcd4;mib2;dlg4 |
| transcription, DNA-dependent; | 48#3439 | 0.028573776 | zbtb46;arid3a;tp53;deaf1;nkx1-2;hoxa6;creb3l2;znf512b;elf3;rassf7;sox15;tbx19;hoxa10;hoxa5;mbd3;tcf3;polr2a;ybx2;tcea2;pcgf3;hoxa3;uncx;sin3a;ccnl2;zbtb37;cux1;hoxa4;hoxa1;myt1;hoxa13;med16;zbtb4;rfx3;evx1;polr2e;foxf2;gmeb2;chd3;irf7;prpf6;irf4;znf142;hoxa2;otx1;sirt3;bcl6b;acad8;rexo1 |
| RNA biosynthetic process; | 48#3444 | 0.028573776 | zbtb46;arid3a;tp53;deaf1;nkx1-2;hoxa6;creb3l2;znf512b;elf3;rassf7;sox15;tbx19;hoxa10;hoxa5;mbd3;tcf3;polr2a;ybx2;tcea2;pcgf3;hoxa3;uncx;sin3a;ccnl2;zbtb37;cux1;hoxa4;hoxa1;myt1;hoxa13;med16;zbtb4;rfx3;evx1;polr2e;foxf2;gmeb2;chd3;irf7;prpf6;irf4;znf142;hoxa2;otx1;sirt3;bcl6b;acad8;rexo1 |
| monocarboxylic acid metabolic process; | 9#287 | 0.028573776 | aloxe3;akr1d1;rnpep;prkag3;alox12b;acadvl;alox12;hao1;alox15b |
| regulation of transcription, DNA-dependent; | 47#3358 | 0.028736401 | zbtb46;arid3a;tp53;deaf1;nkx1-2;hoxa6;creb3l2;znf512b;elf3;rassf7;sox15;tbx19;hoxa10;hoxa5;mbd3;tcf3;polr2a;ybx2;tcea2;pcgf3;hoxa3;uncx;sin3a;ccnl2;zbtb37;cux1;hoxa4;hoxa1;myt1;hoxa13;med16;zbtb4;rfx3;evx1;foxf2;gmeb2;chd3;irf7;prpf6;irf4;znf142;hoxa2;otx1;sirt3;bcl6b;acad8;rexo1 |
| growth%multicellular organism growth; | 3#24 | 0.029417067 | hoxa5;gamt;sod1 |
| localization; | 59#4481 | 0.030779852 | faf1;tp53;erp29;chrna4;oca2;cspg4;slc11a1;grin3b;col20a1;atp5d;mfsd7;bet1l;glra3;gga3;chrnb1;alox12;trappc1;ptpn9;kcnab3;atp1b2;tnnt2;slc16a13;cplx1;herc2;asgr2;tnfsf12;stard4;kctd11;atp8b3;sft2d2;slc2a4;slc25a19;slc16a5;asgr1;gabrg3;abca7;arfgap1;dnah2;shbg;atp5h;kiss1r;vps37b;vps26b;alox15b;clec10a;pitpnm2;spon2;kctd2;camk4;kcnq2;snupn;slc16a11;efnb3;abcb9;gabarap;ipo9;exoc2;scnn1d;scamp4 |
| coenzyme A biosynthetic process; | 2#7 | 0.036975647 | pank2;ppcdc |
| negative regulation of metabolic process; | 11#436 | 0.043383909 | sox15;irf7;cdkn2c;sod1;vil1;rfx3;sin3a;cux1;foxf2;bcl6b;sirt3 |
| fatty acid oxidation; | 3#29 | 0.047116737 | hao1;alox12;acadvl |
| negative regulation of cellular metabolic process; | 10#381 | 0.049533677 | sox15;irf7;cdkn2c;sod1;rfx3;sin3a;cux1;foxf2;bcl6b;sirt3 |
| negative regulation of transcription, DNA-dependent; | 7#203 | 0.049533677 | sox15;sin3a;cux1;irf7;foxf2;sirt3;bcl6b |
| morphogenesis of an epithelium; | 4#63 | 0.049882954 | dvl2;foxf2;elf3;alox15b |
| ear development#ear morphogenesis; | 3#31 | 0.051968194 | hoxa2;otx1;sod1 |
| multicellular organismal development; | 47#2299 | 1.10E-06 | tp53;dvl2;chrna4;cdkn2c;cspg4;hoxa6;alox12b;elf3;hras;sox15;hoxa10;hoxa5;fgf11;stk36;tcf3;hoxa3;tnfsf12;mab21l1;cux1;hoxa4;hoxa1;stmn3;dazap1;kctd11;myt1;maea;hoxa13;dusp22;nav1;evx1;dvl1;gamt;foxf2;adra1d;alox15b;spon2;odf3;kcnq2;letm1;efnb3;fgfr3;irf4;sod1;dlg4;hoxa2;otx1;nlgn2 |
| developmental process; | 61#3347 | 1.10E-06 | faf1;tp53;eef1a2;dvl2;deaf1;chrna4;oca2;cdkn2c;cspg4;hoxa6;alox12b;alox12;elf3;hras;sox15;tbx19;hoxa10;hoxa5;fgf11;eif5a;stk36;tcf3;hoxa3;tnfsf12;mab21l1;cux1;hoxa4;hoxa1;stmn3;dazap1;birc7;kctd11;myt1;rqcd1;maea;hoxa13;tnfrsf18;dusp22;nav1;fgfrl1;evx1;dvl1;gamt;foxf2;adra1d;alox15b;ctsb;spon2;spag16;odf3;kcnq2;letm1;rtel1;irf4;efnb3;fgfr3;sod1;dlg4;hoxa2;otx1;nlgn2 |
| anatomical structure development; | 42#2005 | 1.69E-06 | tp53;dvl2;deaf1;chrna4;cdkn2c;cspg4;alox12b;alox12;elf3;hras;sox15;tbx19;hoxa5;fgf11;tcf3;tnfsf12;mab21l1;cux1;hoxa4;stmn3;kctd11;myt1;maea;hoxa13;fgfrl1;nav1;dvl1;gamt;foxf2;adra1d;alox15b;spon2;kcnq2;spag16;fgfr3;efnb3;irf4;sod1;dlg4;hoxa2;otx1;nlgn2 |
| multicellular organismal process; | 66#3822 | 1.69E-06 | gucy2d;dvl2;hoxa6;chrnb1;hras;sox15;hoxa5;hoxa3;tnfsf12;hoxa4;hoxa1;dazap1;serpinc1;evx1;adra1d;oprl1;alox15b;spon2;camk4;letm1;sod1;otx1;gabarap;nlgn2;scnn1d;akr1d1;tp53;chrna4;cdkn2c;cspg4;alox12b;glra3;sh2b2;elf3;hoxa10;tas1r3;cfd;fgf11;stk36;tcf3;wdr36;cplx1;tnni1;tnnt2;cux1;mab21l1;stmn3;kctd11;myt1;maea;hoxa13;gabrg3;dusp22;nav1;bcs1l;dvl1;gamt;foxf2;npbwr2;kcnq2;odf3;fgfr3;efnb3;irf4;dlg4;hoxa2 |
| anatomical structure morphogenesis; | 27#1047 | 1.69E-06 | kctd11;tp53;dvl2;deaf1;cdkn2c;cspg4;fgfrl1;alox12;dvl1;gamt;foxf2;elf3;adra1d;hras;alox15b;sox15;tbx19;spon2;spag16;efnb3;fgfr3;sod1;hoxa2;otx1;tnfsf12;mab21l1;hoxa4 |
| multicellular organismal development#system development; | 33#1605 | 0.000108292 | dvl2;chrna4;cdkn2c;cspg4;alox12b;elf3;hras;sox15;hoxa5;fgf11;tcf3;tnfsf12;cux1;stmn3;kctd11;myt1;maea;hoxa13;nav1;dvl1;gamt;foxf2;alox15b;spon2;kcnq2;fgfr3;efnb3;irf4;sod1;dlg4;hoxa2;otx1;nlgn2 |
| leukotriene biosynthetic process; | 5#22 | 0.000163798 | aloxe3;alox12;rnpep;alox15b;alox12b |
| alkene biosynthetic process; | 5#22 | 0.000163798 | aloxe3;alox12;rnpep;alox15b;alox12b |
| negative regulation of biological process; | 26#1182 | 0.000243879 | kctd11;faf1;tp53;eef1a2;cdkn2c;aurkaip1;tnfrsf18;rfx3;kiss1r;alox12;foxf2;adra1d;alox15b;sox15;stk11;irf7;rtel1;sod1;vil1;sin3a;ctbp1;cux1;rgs19;sirt3;bcl6b;birc7 |
| negative regulation of cellular process; | 25#1137 | 0.000354881 | kctd11;faf1;tp53;eef1a2;cdkn2c;aurkaip1;tnfrsf18;rfx3;kiss1r;alox12;foxf2;alox15b;sox15;stk11;irf7;rtel1;sod1;vil1;sin3a;cux1;ctbp1;rgs19;sirt3;bcl6b;birc7 |
| transcription from RNA polymerase II promoter; | 17#640 | 0.000354881 | sox15;tbx19;tp53;deaf1;chd3;irf7;prpf6;polr2a;med16;ybx2;otx1;polr2e;cux1;foxf2;gmeb2;bcl6b;elf3 |
| cell-cell signaling; | 17#640 | 0.000354881 | chrna4;gabrg3;glra3;chrnb1;adra1d;npbwr2;camk4;efna2;fgf11;grb2;kcnq2;efnb3;pdgfa;sod1;cplx1;dlg4;gabarap |
| leukotriene metabolic process; | 5#31 | 0.000565716 | aloxe3;alox12;rnpep;alox15b;alox12b |
| alkene metabolic process; | 5#31 | 0.000565716 | aloxe3;alox12;rnpep;alox15b;alox12b |
| carboxylic acid biosynthetic process; | 7#83 | 0.000621942 | aloxe3;akr1d1;alox12;rnpep;prkag3;alox15b;alox12b |
| organic acid biosynthetic process; | 7#83 | 0.000621942 | aloxe3;akr1d1;alox12;rnpep;prkag3;alox15b;alox12b |
| icosanoid biosynthetic process; | 5#33 | 0.000624751 | aloxe3;alox12;rnpep;alox15b;alox12b |
| cellular component organization and biogenesis; | 52#3277 | 0.000624751 | faf1;tp53;erp29;cdkn2c;ict1;grin3b;bet1l;gga3;alox12;sh2b2;elf3;trappc1;sox15;apc2;eif5a;cntrob;wdr36;tnnt2;cplx1;herc2;vil1;asgr2;cnn2;birc7;kctd11;asgr1;med16;abca7;adam2;fgfrl1;bcs1l;ndufs7;vps26b;denr;foxf2;clec10a;spon2;chd3;camk4;spag16;prpf6;snupn;klhl20;efnb3;fgfr3;sod1;dlg4;ipo9;gabarap;exoc2;nlgn2;sirt3 |
| fatty acid biosynthetic process; | 6#73 | 0.002671045 | aloxe3;alox12;rnpep;prkag3;alox15b;alox12b |
| biological regulation; | 88#6731 | 0.002765758 | faf1;arid3a;aurkaip1;ube2j2;nkx1-2;hoxa6;chrnb1;alox12;rassf7;sox15;tbx19;hoxa5;stk11;eif5a;polr2a;ybx2;hoxa3;zbtb37;tnfsf12;ctbp1;hoxa4;hoxa1;birc7;serpinc1;slc2a4;med16;arfgap1;evx1;kiss1r;gmeb2;adra1d;oprl1;alox15b;chd3;irf7;rtel1;sod1;znf142;otx1;sirt3;bcl6b;rexo1;acad8;zbtb46;ube2t;tp53;eef1a2;deaf1;cdkn2c;grin3b;creb3l2;dgkq;sh2b2;znf512b;elf3;gps2;hoxa10;cfd;stk36;mbd3;tcf3;tnnt2;tnni1;tcea2;vil1;pcgf3;uncx;ccnl2;sin3a;cux1;kctd11;myt1;hoxa13;tnfrsf18;dusp22;zbtb4;fgfrl1;rfx3;gamt;foxf2;ctsb;prpf6;fgfr3;irf4;pdgfa;mib2;hoxa2;rgs19 |
| icosanoid metabolic process; | 5#50 | 0.004098518 | aloxe3;alox12;rnpep;alox15b;alox12b |
| regulation of biological process; | 80#6140 | 0.007715708 | faf1;arid3a;aurkaip1;ube2j2;nkx1-2;hoxa6;alox12;rassf7;sox15;tbx19;hoxa5;stk11;eif5a;polr2a;ybx2;hoxa3;zbtb37;tnfsf12;ctbp1;hoxa4;hoxa1;birc7;med16;arfgap1;evx1;kiss1r;gmeb2;adra1d;alox15b;chd3;irf7;rtel1;sod1;znf142;otx1;sirt3;bcl6b;rexo1;acad8;zbtb46;ube2t;tp53;eef1a2;deaf1;cdkn2c;grin3b;creb3l2;sh2b2;znf512b;elf3;hoxa10;cfd;mbd3;stk36;tcf3;tnnt2;tnni1;tcea2;vil1;pcgf3;uncx;ccnl2;sin3a;cux1;kctd11;myt1;hoxa13;tnfrsf18;zbtb4;fgfrl1;rfx3;gamt;foxf2;ctsb;prpf6;irf4;pdgfa;mib2;hoxa2;rgs19 |
| neurological system process#transmission of nerve impulse; | 11#330 | 0.009018628 | npbwr2;chrna4;camk4;kcnq2;gabrg3;sod1;cplx1;dlg4;glra3;gabarap;chrnb1 |
| lipid biosynthetic process; | 11#333 | 0.009342192 | aloxe3;akr1d1;ptdss2;rnpep;sod1;prkag3;alox12b;fdft1;alox12;stard4;alox15b |
| neurological system process#transmission of nerve impulse#synaptic transmission; | 10#290 | 0.012319221 | npbwr2;chrna4;camk4;kcnq2;gabrg3;cplx1;dlg4;glra3;gabarap;chrnb1 |
| fatty acid metabolic process; | 8#191 | 0.013267786 | aloxe3;hao1;alox12;rnpep;prkag3;alox15b;alox12b;acadvl |
| growth#regulation of growth; | 8#201 | 0.01785929 | kctd11;fgfrl1;tp53;alox12;gamt;cdkn2c;sod1;alox15b |
| metabolic process#regulation of metabolic process; | 57#4150 | 0.01785929 | zbtb46;faf1;arid3a;ube2t;tp53;deaf1;cdkn2c;aurkaip1;ube2j2;nkx1-2;hoxa6;creb3l2;sh2b2;znf512b;elf3;rassf7;sox15;tbx19;hoxa10;hoxa5;eif5a;stk36;mbd3;tcf3;polr2a;ybx2;tcea2;vil1;pcgf3;hoxa3;uncx;sin3a;ccnl2;zbtb37;cux1;hoxa4;hoxa1;myt1;hoxa13;med16;zbtb4;rfx3;evx1;foxf2;gmeb2;chd3;irf7;prpf6;irf4;sod1;znf142;hoxa2;otx1;sirt3;bcl6b;rexo1;acad8 |
| ear development; | 4#42 | 0.01785929 | hoxa2;otx1;cux1;sod1 |
| cellular developmental process; | 30#1810 | 0.01785929 | kctd11;faf1;myt1;tp53;maea;eef1a2;deaf1;cdkn2c;tnfrsf18;cspg4;dusp22;nav1;alox12;elf3;alox15b;sox15;ctsb;spon2;eif5a;odf3;tcf3;rtel1;efnb3;irf4;sod1;hoxa2;tnfsf12;cux1;dazap1;birc7 |
| cell differentiation; | 30#1810 | 0.01785929 | kctd11;faf1;myt1;tp53;maea;eef1a2;deaf1;cdkn2c;tnfrsf18;cspg4;dusp22;nav1;alox12;elf3;alox15b;sox15;ctsb;spon2;eif5a;odf3;tcf3;rtel1;efnb3;irf4;sod1;hoxa2;tnfsf12;cux1;dazap1;birc7 |
| sexual reproduction; | 10#315 | 0.018380062 | hoxa10;deaf1;odf3;ybx2;sod1;adam2;abcb9;adam29;gamt;dazap1 |
| gamete generation; | 9#264 | 0.019657528 | hoxa10;deaf1;odf3;ybx2;sod1;abcb9;adam29;gamt;dazap1 |
| spermatogenesis; | 8#212 | 0.019657528 | hoxa10;odf3;ybx2;sod1;abcb9;adam29;gamt;dazap1 |
| male gamete generation; | 8#212 | 0.019657528 | hoxa10;odf3;ybx2;sod1;abcb9;adam29;gamt;dazap1 |
| cellular process#regulation of cellular process; | 73#5704 | 0.019833842 | faf1;arid3a;aurkaip1;nkx1-2;hoxa6;alox12;rassf7;sox15;tbx19;hoxa5;stk11;eif5a;polr2a;ybx2;hoxa3;zbtb37;tnfsf12;ctbp1;hoxa4;hoxa1;birc7;med16;arfgap1;evx1;kiss1r;gmeb2;adra1d;alox15b;chd3;irf7;rtel1;sod1;znf142;otx1;sirt3;bcl6b;rexo1;acad8;zbtb46;tp53;eef1a2;deaf1;cdkn2c;grin3b;creb3l2;znf512b;elf3;hoxa10;mbd3;stk36;tcf3;tcea2;vil1;pcgf3;uncx;ccnl2;sin3a;cux1;kctd11;myt1;hoxa13;tnfrsf18;zbtb4;fgfrl1;rfx3;foxf2;ctsb;prpf6;irf4;pdgfa;mib2;hoxa2;rgs19 |
| multicellular organismal development#system development#organ development; | 21#1141 | 0.024221439 | maea;dvl2;hoxa13;cspg4;alox12b;dvl1;gamt;foxf2;elf3;hras;alox15b;sox15;hoxa5;tcf3;fgfr3;irf4;sod1;hoxa2;otx1;tnfsf12;cux1 |
| nervous system development; | 15#716 | 0.028360456 | kctd11;myt1;spon2;fgf11;dvl2;chrna4;kcnq2;cdkn2c;efnb3;sod1;nav1;dlg4;otx1;nlgn2;stmn3 |
| primary metabolic process; | 141#12764 | 0.028573776 | idua;faf1;gucy2d;erp29;dnajc5;ict1;ube2j2;hoxa6;neil1;dars2;nadk;sox15;man2c1;gmds;stk11;polr2a;ogfod2;ybx2;adam29;ctbp1;hoxa4;b3galt6;tspan8;mmp23b;ttll10;gmeb2;adra1d;alox15b;chd3;irf7;znf142;sirt3;tp53;deaf1;pcsk4;ddi1;znf512b;aloxe3;ptk6;ptpn9;cfd;stk36;mrpl20;tcf3;wdr36;herc2;sin3a;ccnl2;eif4a1;glb1l3;mif4gd;adam32;hoxa13;midn;dusp22;adam2;atp5h;ndufs7;prdx6;foxf2;ptdss2;prpf6;klhl20;hoxa2;mpdu1;arid3a;aurkaip1;gak;nkx1-2;rnf25;alox12;gga3;pusl1;rassf7;tbx19;b3gat1;hoxa5;eif5a;prkag3;ptpn7;hoxa3;fdft1;zbtb37;hoxa1;mapkapk5;birc7;slc2a4;ssu72;htra4;med16;evx1;usp37;athl1;ttll4;camk4;sod1;ipo9;otx1;csnk1g2;bcl6b;acad8;rexo1;zbtb46;ube2t;akr1d1;eef1a2;atp5d;alox12b;acadvl;creb3l2;elf3;hoxa10;apc2;tnk1;mbd3;tcea2;vil1;pcgf3;uncx;adam9;cux1;srms;stard4;mrps7;myt1;nt5c;rnpep;slbp;zbtb4;rfx3;bcs1l;hao1;polr2e;gamt;denr;ctsb;fgfr3;irf4;plcd4;mib2;dlg4 |
| transcription, DNA-dependent; | 48#3439 | 0.028573776 | zbtb46;arid3a;tp53;deaf1;nkx1-2;hoxa6;creb3l2;znf512b;elf3;rassf7;sox15;tbx19;hoxa10;hoxa5;mbd3;tcf3;polr2a;ybx2;tcea2;pcgf3;hoxa3;uncx;sin3a;ccnl2;zbtb37;cux1;hoxa4;hoxa1;myt1;hoxa13;med16;zbtb4;rfx3;evx1;polr2e;foxf2;gmeb2;chd3;irf7;prpf6;irf4;znf142;hoxa2;otx1;sirt3;bcl6b;acad8;rexo1 |
| RNA biosynthetic process; | 48#3444 | 0.028573776 | zbtb46;arid3a;tp53;deaf1;nkx1-2;hoxa6;creb3l2;znf512b;elf3;rassf7;sox15;tbx19;hoxa10;hoxa5;mbd3;tcf3;polr2a;ybx2;tcea2;pcgf3;hoxa3;uncx;sin3a;ccnl2;zbtb37;cux1;hoxa4;hoxa1;myt1;hoxa13;med16;zbtb4;rfx3;evx1;polr2e;foxf2;gmeb2;chd3;irf7;prpf6;irf4;znf142;hoxa2;otx1;sirt3;bcl6b;acad8;rexo1 |
| monocarboxylic acid metabolic process; | 9#287 | 0.028573776 | aloxe3;akr1d1;rnpep;prkag3;alox12b;acadvl;alox12;hao1;alox15b |
| regulation of transcription, DNA-dependent; | 47#3358 | 0.028736401 | zbtb46;arid3a;tp53;deaf1;nkx1-2;hoxa6;creb3l2;znf512b;elf3;rassf7;sox15;tbx19;hoxa10;hoxa5;mbd3;tcf3;polr2a;ybx2;tcea2;pcgf3;hoxa3;uncx;sin3a;ccnl2;zbtb37;cux1;hoxa4;hoxa1;myt1;hoxa13;med16;zbtb4;rfx3;evx1;foxf2;gmeb2;chd3;irf7;prpf6;irf4;znf142;hoxa2;otx1;sirt3;bcl6b;acad8;rexo1 |
| growth%multicellular organism growth; | 3#24 | 0.029417067 | hoxa5;gamt;sod1 |
| localization; | 59#4481 | 0.030779852 | faf1;tp53;erp29;chrna4;oca2;cspg4;slc11a1;grin3b;col20a1;atp5d;mfsd7;bet1l;glra3;gga3;chrnb1;alox12;trappc1;ptpn9;kcnab3;atp1b2;tnnt2;slc16a13;cplx1;herc2;asgr2;tnfsf12;stard4;kctd11;atp8b3;sft2d2;slc2a4;slc25a19;slc16a5;asgr1;gabrg3;abca7;arfgap1;dnah2;shbg;atp5h;kiss1r;vps37b;vps26b;alox15b;clec10a;pitpnm2;spon2;kctd2;camk4;kcnq2;snupn;slc16a11;efnb3;abcb9;gabarap;ipo9;exoc2;scnn1d;scamp4 |
| coenzyme A biosynthetic process; | 2#7 | 0.036975647 | pank2;ppcdc |
