# Supplementary material for: Genome-wide analysis reveals signatures of selection for important traits in domestic sheep from different ecoregions
Source: BMC Genomics. 2016 Nov 3;17:863. doi: 10.1186/s12864-016-3212-2 (PMC5094087; doi:10.1186/s12864-016-3212-2)
Supplement: Additional file 21: Table S16. — Enriched GO terms among genes located in the selection regions with Z(F ST)M-S > 4. (DOC 145 kb) [file 12864_2016_3212_MOESM21_ESM.doc]

**Additional file 21: Table S16.** Enriched GO terms among genes located in the selection regions with Z(*F*_ST_)_M-S_ > 4.

| GO term | Gene count | P value | genes |
| --- | --- | --- | --- |
| vesicle-mediated transport; | 7#606 | 0.052367683 | slc9a3;exoc3;actr1a;tmed2;azu1;gbf1;nkd2 |
| collagen catabolic process; | 2#20 | 0.052367683 | mmp11;prtn3 |
| multicellular organismal macromolecule metabolic process; | 2#20 | 0.052367683 | mmp11;prtn3 |
| multicellular organismal protein catabolic process; | 2#20 | 0.052367683 | mmp11;prtn3 |
| multicellular organismal protein metabolic process; | 2#20 | 0.052367683 | mmp11;prtn3 |
| protein digestion; | 2#20 | 0.052367683 | mmp11;prtn3 |
| multicellular organismal macromolecule catabolic process; | 2#20 | 0.052367683 | mmp11;prtn3 |
| multicellular organismal catabolic process; | 2#21 | 0.052367683 | mmp11;prtn3 |
| collagen metabolic process; | 2#21 | 0.052367683 | mmp11;prtn3 |
| multicellular organismal metabolic process; | 2#23 | 0.056623875 | mmp11;prtn3 |
| regulation of protein polymerization; | 1#1 | 0.058711078 | tppp |
| positive regulation of cellular protein metabolic process; | 1#1 | 0.058711078 | tppp |
| boron transport; | 1#1 | 0.058711078 | slc4a11 |
| positive regulation of protein polymerization; | 1#1 | 0.058711078 | tppp |
| regulation of cellular protein metabolic process; | 1#1 | 0.058711078 | tppp |
| regulation of pH; | 2#33 | 0.058711078 | slc9a3;atp6v0a4 |
| cellular component organization and biogenesis; | 15#3277 | 0.058711078 | rcor1;h2afz;smarcb1;nup37;nolc1;nkd2;slc9a3;palm;exoc3;itch;azu1;gbf1;tppp;cabin1;hps6 |
| anatomical structure development; | 11#2005 | 0.058711078 | bnc1;ldb1;smarcb1;pitx3;flt1;mmp11;sufu;palm;atp6v0a4;itch;azu1 |
| chemical homeostasis; | 4#302 | 0.058711078 | slc9a3;slc4a11;atp6v0a4;oxt |
| reproductive process; | 4#305 | 0.058711078 | flt1;itch;smarcb1;oxt |
| interleukin-1 production#interleukin-1 biosynthetic process; | 1#2 | 0.058711078 | azu1 |
| interleukin-1 beta biosynthetic process; | 1#2 | 0.058711078 | azu1 |
| positive regulation of interleukin-1 beta biosynthetic process; | 1#2 | 0.058711078 | azu1 |
| vesicle-mediated transport; | 7#606 | 0.052367683 | slc9a3;exoc3;actr1a;tmed2;azu1;gbf1;nkd2 |
| collagen catabolic process; | 2#20 | 0.052367683 | mmp11;prtn3 |
| multicellular organismal macromolecule metabolic process; | 2#20 | 0.052367683 | mmp11;prtn3 |
| multicellular organismal protein catabolic process; | 2#20 | 0.052367683 | mmp11;prtn3 |
| multicellular organismal protein metabolic process; | 2#20 | 0.052367683 | mmp11;prtn3 |
| protein digestion; | 2#20 | 0.052367683 | mmp11;prtn3 |
| multicellular organismal macromolecule catabolic process; | 2#20 | 0.052367683 | mmp11;prtn3 |
| multicellular organismal catabolic process; | 2#21 | 0.052367683 | mmp11;prtn3 |
| collagen metabolic process; | 2#21 | 0.052367683 | mmp11;prtn3 |
| multicellular organismal metabolic process; | 2#23 | 0.056623875 | mmp11;prtn3 |
| regulation of protein polymerization; | 1#1 | 0.058711078 | tppp |
| positive regulation of cellular protein metabolic process; | 1#1 | 0.058711078 | tppp |
| boron transport; | 1#1 | 0.058711078 | slc4a11 |
| positive regulation of protein polymerization; | 1#1 | 0.058711078 | tppp |
| regulation of cellular protein metabolic process; | 1#1 | 0.058711078 | tppp |
| regulation of pH; | 2#33 | 0.058711078 | slc9a3;atp6v0a4 |
| cellular component organization and biogenesis; | 15#3277 | 0.058711078 | rcor1;h2afz;smarcb1;nup37;nolc1;nkd2;slc9a3;palm;exoc3;itch;azu1;gbf1;tppp;cabin1;hps6 |
| anatomical structure development; | 11#2005 | 0.058711078 | bnc1;ldb1;smarcb1;pitx3;flt1;mmp11;sufu;palm;atp6v0a4;itch;azu1 |
| chemical homeostasis; | 4#302 | 0.058711078 | slc9a3;slc4a11;atp6v0a4;oxt |
| reproductive process; | 4#305 | 0.058711078 | flt1;itch;smarcb1;oxt |
| interleukin-1 production#interleukin-1 biosynthetic process; | 1#2 | 0.058711078 | azu1 |
| interleukin-1 beta biosynthetic process; | 1#2 | 0.058711078 | azu1 |
| positive regulation of interleukin-1 beta biosynthetic process; | 1#2 | 0.058711078 | azu1 |
| vesicle-mediated transport; | 7#606 | 0.052367683 | slc9a3;exoc3;actr1a;tmed2;azu1;gbf1;nkd2 |
| collagen catabolic process; | 2#20 | 0.052367683 | mmp11;prtn3 |
| multicellular organismal macromolecule metabolic process; | 2#20 | 0.052367683 | mmp11;prtn3 |
| multicellular organismal protein catabolic process; | 2#20 | 0.052367683 | mmp11;prtn3 |
| multicellular organismal protein metabolic process; | 2#20 | 0.052367683 | mmp11;prtn3 |
| protein digestion; | 2#20 | 0.052367683 | mmp11;prtn3 |
| multicellular organismal macromolecule catabolic process; | 2#20 | 0.052367683 | mmp11;prtn3 |
| multicellular organismal catabolic process; | 2#21 | 0.052367683 | mmp11;prtn3 |
| collagen metabolic process; | 2#21 | 0.052367683 | mmp11;prtn3 |
| multicellular organismal metabolic process; | 2#23 | 0.056623875 | mmp11;prtn3 |
| regulation of protein polymerization; | 1#1 | 0.058711078 | tppp |
| positive regulation of cellular protein metabolic process; | 1#1 | 0.058711078 | tppp |
| boron transport; | 1#1 | 0.058711078 | slc4a11 |
| positive regulation of protein polymerization; | 1#1 | 0.058711078 | tppp |
| regulation of cellular protein metabolic process; | 1#1 | 0.058711078 | tppp |
| regulation of pH; | 2#33 | 0.058711078 | slc9a3;atp6v0a4 |
| cellular component organization and biogenesis; | 15#3277 | 0.058711078 | rcor1;h2afz;smarcb1;nup37;nolc1;nkd2;slc9a3;palm;exoc3;itch;azu1;gbf1;tppp;cabin1;hps6 |
| anatomical structure development; | 11#2005 | 0.058711078 | bnc1;ldb1;smarcb1;pitx3;flt1;mmp11;sufu;palm;atp6v0a4;itch;azu1 |
| chemical homeostasis; | 4#302 | 0.058711078 | slc9a3;slc4a11;atp6v0a4;oxt |
| reproductive process; | 4#305 | 0.058711078 | flt1;itch;smarcb1;oxt |
| interleukin-1 production#interleukin-1 biosynthetic process; | 1#2 | 0.058711078 | azu1 |
| interleukin-1 beta biosynthetic process; | 1#2 | 0.058711078 | azu1 |
| positive regulation of interleukin-1 beta biosynthetic process; | 1#2 | 0.058711078 | azu1 |
| vesicle-mediated transport; | 7#606 | 0.052367683 | slc9a3;exoc3;actr1a;tmed2;azu1;gbf1;nkd2 |
| collagen catabolic process; | 2#20 | 0.052367683 | mmp11;prtn3 |
| multicellular organismal macromolecule metabolic process; | 2#20 | 0.052367683 | mmp11;prtn3 |
| multicellular organismal protein catabolic process; | 2#20 | 0.052367683 | mmp11;prtn3 |
| multicellular organismal protein metabolic process; | 2#20 | 0.052367683 | mmp11;prtn3 |
| protein digestion; | 2#20 | 0.052367683 | mmp11;prtn3 |
| multicellular organismal macromolecule catabolic process; | 2#20 | 0.052367683 | mmp11;prtn3 |
| multicellular organismal catabolic process; | 2#21 | 0.052367683 | mmp11;prtn3 |
| collagen metabolic process; | 2#21 | 0.052367683 | mmp11;prtn3 |
| multicellular organismal metabolic process; | 2#23 | 0.056623875 | mmp11;prtn3 |
| regulation of protein polymerization; | 1#1 | 0.058711078 | tppp |
| positive regulation of cellular protein metabolic process; | 1#1 | 0.058711078 | tppp |
| boron transport; | 1#1 | 0.058711078 | slc4a11 |
| positive regulation of protein polymerization; | 1#1 | 0.058711078 | tppp |
| regulation of cellular protein metabolic process; | 1#1 | 0.058711078 | tppp |
| regulation of pH; | 2#33 | 0.058711078 | slc9a3;atp6v0a4 |
| cellular component organization and biogenesis; | 15#3277 | 0.058711078 | rcor1;h2afz;smarcb1;nup37;nolc1;nkd2;slc9a3;palm;exoc3;itch;azu1;gbf1;tppp;cabin1;hps6 |
| anatomical structure development; | 11#2005 | 0.058711078 | bnc1;ldb1;smarcb1;pitx3;flt1;mmp11;sufu;palm;atp6v0a4;itch;azu1 |
| chemical homeostasis; | 4#302 | 0.058711078 | slc9a3;slc4a11;atp6v0a4;oxt |
| reproductive process; | 4#305 | 0.058711078 | flt1;itch;smarcb1;oxt |
| interleukin-1 production#interleukin-1 biosynthetic process; | 1#2 | 0.058711078 | azu1 |
| interleukin-1 beta biosynthetic process; | 1#2 | 0.058711078 | azu1 |
| positive regulation of interleukin-1 beta biosynthetic process; | 1#2 | 0.058711078 | azu1 |
| vesicle-mediated transport; | 7#606 | 0.052367683 | slc9a3;exoc3;actr1a;tmed2;azu1;gbf1;nkd2 |
| collagen catabolic process; | 2#20 | 0.052367683 | mmp11;prtn3 |
| multicellular organismal macromolecule metabolic process; | 2#20 | 0.052367683 | mmp11;prtn3 |
| multicellular organismal protein catabolic process; | 2#20 | 0.052367683 | mmp11;prtn3 |
| multicellular organismal protein metabolic process; | 2#20 | 0.052367683 | mmp11;prtn3 |
| protein digestion; | 2#20 | 0.052367683 | mmp11;prtn3 |
| multicellular organismal macromolecule catabolic process; | 2#20 | 0.052367683 | mmp11;prtn3 |
| multicellular organismal catabolic process; | 2#21 | 0.052367683 | mmp11;prtn3 |
| collagen metabolic process; | 2#21 | 0.052367683 | mmp11;prtn3 |
| multicellular organismal metabolic process; | 2#23 | 0.056623875 | mmp11;prtn3 |
| regulation of protein polymerization; | 1#1 | 0.058711078 | tppp |
| positive regulation of cellular protein metabolic process; | 1#1 | 0.058711078 | tppp |
| boron transport; | 1#1 | 0.058711078 | slc4a11 |
| positive regulation of protein polymerization; | 1#1 | 0.058711078 | tppp |
| regulation of cellular protein metabolic process; | 1#1 | 0.058711078 | tppp |
| regulation of pH; | 2#33 | 0.058711078 | slc9a3;atp6v0a4 |
| cellular component organization and biogenesis; | 15#3277 | 0.058711078 | rcor1;h2afz;smarcb1;nup37;nolc1;nkd2;slc9a3;palm;exoc3;itch;azu1;gbf1;tppp;cabin1;hps6 |
| anatomical structure development; | 11#2005 | 0.058711078 | bnc1;ldb1;smarcb1;pitx3;flt1;mmp11;sufu;palm;atp6v0a4;itch;azu1 |
| chemical homeostasis; | 4#302 | 0.058711078 | slc9a3;slc4a11;atp6v0a4;oxt |
| reproductive process; | 4#305 | 0.058711078 | flt1;itch;smarcb1;oxt |
| interleukin-1 production#interleukin-1 biosynthetic process; | 1#2 | 0.058711078 | azu1 |
| interleukin-1 beta biosynthetic process; | 1#2 | 0.058711078 | azu1 |
| positive regulation of interleukin-1 beta biosynthetic process; | 1#2 | 0.058711078 | azu1 |
| vesicle-mediated transport; | 7#606 | 0.052367683 | slc9a3;exoc3;actr1a;tmed2;azu1;gbf1;nkd2 |
| collagen catabolic process; | 2#20 | 0.052367683 | mmp11;prtn3 |
| multicellular organismal macromolecule metabolic process; | 2#20 | 0.052367683 | mmp11;prtn3 |
| multicellular organismal protein catabolic process; | 2#20 | 0.052367683 | mmp11;prtn3 |
| multicellular organismal protein metabolic process; | 2#20 | 0.052367683 | mmp11;prtn3 |
| protein digestion; | 2#20 | 0.052367683 | mmp11;prtn3 |
| multicellular organismal macromolecule catabolic process; | 2#20 | 0.052367683 | mmp11;prtn3 |
| multicellular organismal catabolic process; | 2#21 | 0.052367683 | mmp11;prtn3 |
| collagen metabolic process; | 2#21 | 0.052367683 | mmp11;prtn3 |
| multicellular organismal metabolic process; | 2#23 | 0.056623875 | mmp11;prtn3 |
| regulation of protein polymerization; | 1#1 | 0.058711078 | tppp |
| positive regulation of cellular protein metabolic process; | 1#1 | 0.058711078 | tppp |
| boron transport; | 1#1 | 0.058711078 | slc4a11 |
| positive regulation of protein polymerization; | 1#1 | 0.058711078 | tppp |
| regulation of cellular protein metabolic process; | 1#1 | 0.058711078 | tppp |
| regulation of pH; | 2#33 | 0.058711078 | slc9a3;atp6v0a4 |
| cellular component organization and biogenesis; | 15#3277 | 0.058711078 | rcor1;h2afz;smarcb1;nup37;nolc1;nkd2;slc9a3;palm;exoc3;itch;azu1;gbf1;tppp;cabin1;hps6 |
| anatomical structure development; | 11#2005 | 0.058711078 | bnc1;ldb1;smarcb1;pitx3;flt1;mmp11;sufu;palm;atp6v0a4;itch;azu1 |
| chemical homeostasis; | 4#302 | 0.058711078 | slc9a3;slc4a11;atp6v0a4;oxt |
| reproductive process; | 4#305 | 0.058711078 | flt1;itch;smarcb1;oxt |
| interleukin-1 production#interleukin-1 biosynthetic process; | 1#2 | 0.058711078 | azu1 |
| interleukin-1 beta biosynthetic process; | 1#2 | 0.058711078 | azu1 |
| positive regulation of interleukin-1 beta biosynthetic process; | 1#2 | 0.058711078 | azu1 |
| vesicle-mediated transport; | 7#606 | 0.052367683 | slc9a3;exoc3;actr1a;tmed2;azu1;gbf1;nkd2 |
| collagen catabolic process; | 2#20 | 0.052367683 | mmp11;prtn3 |
| multicellular organismal macromolecule metabolic process; | 2#20 | 0.052367683 | mmp11;prtn3 |
| multicellular organismal protein catabolic process; | 2#20 | 0.052367683 | mmp11;prtn3 |
| multicellular organismal protein metabolic process; | 2#20 | 0.052367683 | mmp11;prtn3 |
| protein digestion; | 2#20 | 0.052367683 | mmp11;prtn3 |
| multicellular organismal macromolecule catabolic process; | 2#20 | 0.052367683 | mmp11;prtn3 |
| multicellular organismal catabolic process; | 2#21 | 0.052367683 | mmp11;prtn3 |
| collagen metabolic process; | 2#21 | 0.052367683 | mmp11;prtn3 |
| multicellular organismal metabolic process; | 2#23 | 0.056623875 | mmp11;prtn3 |
| regulation of protein polymerization; | 1#1 | 0.058711078 | tppp |
| positive regulation of cellular protein metabolic process; | 1#1 | 0.058711078 | tppp |
