# Supplementary material for: Genome-wide analysis reveals signatures of selection for important traits in domestic sheep from different ecoregions
Source: BMC Genomics. 2016 Nov 3;17:863. doi: 10.1186/s12864-016-3212-2 (PMC5094087; doi:10.1186/s12864-016-3212-2)
Supplement: Additional file 22: Table S17. — Enriched GO terms among genes located in the selection regions with Z(F ST)M-D > 4. (DOC 145 kb) [file 12864_2016_3212_MOESM22_ESM.doc]

**Additional file 22: Table S17.** Enriched GO terms among genes located in the selection regions with Z(*F*_ST_)_M-D_ > 4.

| GO term | Gene count | P value | genes |
| --- | --- | --- | --- |
| sexual reproduction; | 5#315 | 0.004804868 | pax5;adam20;ccin;kit;adam21 |
| post-translational protein modification; | 9#2235 | 0.034487806 | uhrf2;herc5;tulp4;map3k9;ppm1k;flt4;herc3;melk;kit |
| transcription from RNA polymerase II promoter; | 5#640 | 0.034487806 | tarbp1;pax5;med6;t;ppara |
| positive regulation of fatty acid beta-oxidation; | 1#1 | 0.034487806 | ppara |
| spermatogenesis; | 3#212 | 0.034487806 | pax5;ccin;kit |
| male gamete generation; | 3#212 | 0.034487806 | pax5;ccin;kit |
| single fertilization; | 2#56 | 0.034487806 | adam20;adam21 |
| protein modification process; | 9#2704 | 0.034487806 | uhrf2;herc5;tulp4;map3k9;ppm1k;flt4;herc3;melk;kit |
| fertilization; | 2#58 | 0.034487806 | adam20;adam21 |
| positive regulation of cell proliferation; | 3#233 | 0.034487806 | flt4;kit;t |
| regulation of protein kinase activity; | 3#237 | 0.034487806 | map3k9;herc5;kit |
| regulation of fatty acid beta-oxidation; | 1#2 | 0.034487806 | ppara |
| regulation of kinase activity; | 3#242 | 0.034487806 | map3k9;herc5;kit |
| biopolymer modification; | 9#2815 | 0.034487806 | uhrf2;herc5;tulp4;map3k9;ppm1k;flt4;herc3;melk;kit |
| positive regulation of MAP kinase activity; | 2#66 | 0.034487806 | map3k9;kit |
| regulation of transferase activity; | 3#247 | 0.034487806 | map3k9;herc5;kit |
| ubiquitin cycle; | 4#549 | 0.035078313 | tulp4;uhrf2;herc5;herc3 |
| gamete generation; | 3#264 | 0.035078313 | pax5;ccin;kit |
| positive regulation of cellular process; | 5#954 | 0.035078313 | med6;flt4;kit;t;ppara |
| N-acetylneuraminate metabolic process; | 1#3 | 0.035078313 | gne |
| positive regulation of fatty acid oxidation; | 1#3 | 0.035078313 | ppara |
| protein autoubiquitination; | 1#3 | 0.035078313 | uhrf2 |
| cellular macromolecule metabolic process; | 13#5656 | 0.037880194 | uhrf2;herc5;gne;tulp4;ppm1k;map3k9;adam20;flt4;herc3;melk;ttc9;adam21;kit |
| notochord development; | 1#4 | 0.041087442 | t |
| regulation of fatty acid oxidation; | 1#4 | 0.041087442 | ppara |
| regulation of MAP kinase activity; | 2#93 | 0.041087442 | map3k9;kit |
| positive regulation of biological process; | 5#1062 | 0.044306224 | med6;flt4;kit;t;ppara |
| biopolymer metabolic process; | 16#7940 | 0.044306224 | pax5;herc5;tulp4;tarbp1;ppm1k;gtf2h5;map3k9;t;gne;uhrf2;ppara;med6;melk;herc3;flt4;kit |
| UDP-N-acetylglucosamine metabolic process; | 1#5 | 0.044306224 | gne |
| protein metabolic process; | 13#5858 | 0.044353911 | uhrf2;herc5;tulp4;ppm1k;map3k9;adam20;flt4;herc3;melk;ttc9;clta;adam21;kit |
| macromolecule metabolic process; | 20#11144 | 0.047288977 | pax5;herc5;tulp4;tarbp1;ppm1k;map3k9;gtf2h5;adam20;ttc9;adam21;t;uhrf2;gne;ppara;med6;herc3;flt4;melk;clta;kit |
| positive regulation of fatty acid metabolic process; | 1#6 | 0.047288977 | ppara |
| regulation of cell cycle; | 3#359 | 0.047288977 | reck;uhrf2;herc5 |
| tripartite regional subdivision#determination of anterior/posterior axis, embryo; | 1#7 | 0.047288977 | t |
| fatty acid transport; | 1#7 | 0.047288977 | ppara |
| blastoderm segmentation; | 1#7 | 0.047288977 | t |
| embryonic axis specification; | 1#7 | 0.047288977 | t |
| tripartite regional subdivision; | 1#7 | 0.047288977 | t |
| cell proliferation; | 4#745 | 0.048259512 | uhrf2;flt4;kit;t |
| sexual reproduction; | 5#315 | 0.004804868 | pax5;adam20;ccin;kit;adam21 |
| post-translational protein modification; | 9#2235 | 0.034487806 | uhrf2;herc5;tulp4;map3k9;ppm1k;flt4;herc3;melk;kit |
| transcription from RNA polymerase II promoter; | 5#640 | 0.034487806 | tarbp1;pax5;med6;t;ppara |
| positive regulation of fatty acid beta-oxidation; | 1#1 | 0.034487806 | ppara |
| spermatogenesis; | 3#212 | 0.034487806 | pax5;ccin;kit |
| male gamete generation; | 3#212 | 0.034487806 | pax5;ccin;kit |
| single fertilization; | 2#56 | 0.034487806 | adam20;adam21 |
| protein modification process; | 9#2704 | 0.034487806 | uhrf2;herc5;tulp4;map3k9;ppm1k;flt4;herc3;melk;kit |
| fertilization; | 2#58 | 0.034487806 | adam20;adam21 |
| positive regulation of cell proliferation; | 3#233 | 0.034487806 | flt4;kit;t |
| regulation of protein kinase activity; | 3#237 | 0.034487806 | map3k9;herc5;kit |
| regulation of fatty acid beta-oxidation; | 1#2 | 0.034487806 | ppara |
| regulation of kinase activity; | 3#242 | 0.034487806 | map3k9;herc5;kit |
| biopolymer modification; | 9#2815 | 0.034487806 | uhrf2;herc5;tulp4;map3k9;ppm1k;flt4;herc3;melk;kit |
| positive regulation of MAP kinase activity; | 2#66 | 0.034487806 | map3k9;kit |
| regulation of transferase activity; | 3#247 | 0.034487806 | map3k9;herc5;kit |
| ubiquitin cycle; | 4#549 | 0.035078313 | tulp4;uhrf2;herc5;herc3 |
| gamete generation; | 3#264 | 0.035078313 | pax5;ccin;kit |
| positive regulation of cellular process; | 5#954 | 0.035078313 | med6;flt4;kit;t;ppara |
| N-acetylneuraminate metabolic process; | 1#3 | 0.035078313 | gne |
| positive regulation of fatty acid oxidation; | 1#3 | 0.035078313 | ppara |
| protein autoubiquitination; | 1#3 | 0.035078313 | uhrf2 |
| cellular macromolecule metabolic process; | 13#5656 | 0.037880194 | uhrf2;herc5;gne;tulp4;ppm1k;map3k9;adam20;flt4;herc3;melk;ttc9;adam21;kit |
| notochord development; | 1#4 | 0.041087442 | t |
| regulation of fatty acid oxidation; | 1#4 | 0.041087442 | ppara |
| regulation of MAP kinase activity; | 2#93 | 0.041087442 | map3k9;kit |
| positive regulation of biological process; | 5#1062 | 0.044306224 | med6;flt4;kit;t;ppara |
| biopolymer metabolic process; | 16#7940 | 0.044306224 | pax5;herc5;tulp4;tarbp1;ppm1k;gtf2h5;map3k9;t;gne;uhrf2;ppara;med6;melk;herc3;flt4;kit |
| UDP-N-acetylglucosamine metabolic process; | 1#5 | 0.044306224 | gne |
| protein metabolic process; | 13#5858 | 0.044353911 | uhrf2;herc5;tulp4;ppm1k;map3k9;adam20;flt4;herc3;melk;ttc9;clta;adam21;kit |
| macromolecule metabolic process; | 20#11144 | 0.047288977 | pax5;herc5;tulp4;tarbp1;ppm1k;map3k9;gtf2h5;adam20;ttc9;adam21;t;uhrf2;gne;ppara;med6;herc3;flt4;melk;clta;kit |
| positive regulation of fatty acid metabolic process; | 1#6 | 0.047288977 | ppara |
| regulation of cell cycle; | 3#359 | 0.047288977 | reck;uhrf2;herc5 |
| tripartite regional subdivision#determination of anterior/posterior axis, embryo; | 1#7 | 0.047288977 | t |
| fatty acid transport; | 1#7 | 0.047288977 | ppara |
| blastoderm segmentation; | 1#7 | 0.047288977 | t |
| embryonic axis specification; | 1#7 | 0.047288977 | t |
| tripartite regional subdivision; | 1#7 | 0.047288977 | t |
| cell proliferation; | 4#745 | 0.048259512 | uhrf2;flt4;kit;t |
| sexual reproduction; | 5#315 | 0.004804868 | pax5;adam20;ccin;kit;adam21 |
| post-translational protein modification; | 9#2235 | 0.034487806 | uhrf2;herc5;tulp4;map3k9;ppm1k;flt4;herc3;melk;kit |
| transcription from RNA polymerase II promoter; | 5#640 | 0.034487806 | tarbp1;pax5;med6;t;ppara |
| positive regulation of fatty acid beta-oxidation; | 1#1 | 0.034487806 | ppara |
| spermatogenesis; | 3#212 | 0.034487806 | pax5;ccin;kit |
| male gamete generation; | 3#212 | 0.034487806 | pax5;ccin;kit |
| single fertilization; | 2#56 | 0.034487806 | adam20;adam21 |
| protein modification process; | 9#2704 | 0.034487806 | uhrf2;herc5;tulp4;map3k9;ppm1k;flt4;herc3;melk;kit |
| fertilization; | 2#58 | 0.034487806 | adam20;adam21 |
| positive regulation of cell proliferation; | 3#233 | 0.034487806 | flt4;kit;t |
| regulation of protein kinase activity; | 3#237 | 0.034487806 | map3k9;herc5;kit |
| regulation of fatty acid beta-oxidation; | 1#2 | 0.034487806 | ppara |
| regulation of kinase activity; | 3#242 | 0.034487806 | map3k9;herc5;kit |
| biopolymer modification; | 9#2815 | 0.034487806 | uhrf2;herc5;tulp4;map3k9;ppm1k;flt4;herc3;melk;kit |
| positive regulation of MAP kinase activity; | 2#66 | 0.034487806 | map3k9;kit |
| regulation of transferase activity; | 3#247 | 0.034487806 | map3k9;herc5;kit |
| ubiquitin cycle; | 4#549 | 0.035078313 | tulp4;uhrf2;herc5;herc3 |
| gamete generation; | 3#264 | 0.035078313 | pax5;ccin;kit |
| positive regulation of cellular process; | 5#954 | 0.035078313 | med6;flt4;kit;t;ppara |
| N-acetylneuraminate metabolic process; | 1#3 | 0.035078313 | gne |
| positive regulation of fatty acid oxidation; | 1#3 | 0.035078313 | ppara |
| protein autoubiquitination; | 1#3 | 0.035078313 | uhrf2 |
| cellular macromolecule metabolic process; | 13#5656 | 0.037880194 | uhrf2;herc5;gne;tulp4;ppm1k;map3k9;adam20;flt4;herc3;melk;ttc9;adam21;kit |
| notochord development; | 1#4 | 0.041087442 | t |
| regulation of fatty acid oxidation; | 1#4 | 0.041087442 | ppara |
| regulation of MAP kinase activity; | 2#93 | 0.041087442 | map3k9;kit |
| positive regulation of biological process; | 5#1062 | 0.044306224 | med6;flt4;kit;t;ppara |
| biopolymer metabolic process; | 16#7940 | 0.044306224 | pax5;herc5;tulp4;tarbp1;ppm1k;gtf2h5;map3k9;t;gne;uhrf2;ppara;med6;melk;herc3;flt4;kit |
| UDP-N-acetylglucosamine metabolic process; | 1#5 | 0.044306224 | gne |
| protein metabolic process; | 13#5858 | 0.044353911 | uhrf2;herc5;tulp4;ppm1k;map3k9;adam20;flt4;herc3;melk;ttc9;clta;adam21;kit |
| macromolecule metabolic process; | 20#11144 | 0.047288977 | pax5;herc5;tulp4;tarbp1;ppm1k;map3k9;gtf2h5;adam20;ttc9;adam21;t;uhrf2;gne;ppara;med6;herc3;flt4;melk;clta;kit |
| positive regulation of fatty acid metabolic process; | 1#6 | 0.047288977 | ppara |
| regulation of cell cycle; | 3#359 | 0.047288977 | reck;uhrf2;herc5 |
| tripartite regional subdivision#determination of anterior/posterior axis, embryo; | 1#7 | 0.047288977 | t |
| fatty acid transport; | 1#7 | 0.047288977 | ppara |
| blastoderm segmentation; | 1#7 | 0.047288977 | t |
| embryonic axis specification; | 1#7 | 0.047288977 | t |
| tripartite regional subdivision; | 1#7 | 0.047288977 | t |
| cell proliferation; | 4#745 | 0.048259512 | uhrf2;flt4;kit;t |
| sexual reproduction; | 5#315 | 0.004804868 | pax5;adam20;ccin;kit;adam21 |
| post-translational protein modification; | 9#2235 | 0.034487806 | uhrf2;herc5;tulp4;map3k9;ppm1k;flt4;herc3;melk;kit |
| transcription from RNA polymerase II promoter; | 5#640 | 0.034487806 | tarbp1;pax5;med6;t;ppara |
| positive regulation of fatty acid beta-oxidation; | 1#1 | 0.034487806 | ppara |
| spermatogenesis; | 3#212 | 0.034487806 | pax5;ccin;kit |
| male gamete generation; | 3#212 | 0.034487806 | pax5;ccin;kit |
| single fertilization; | 2#56 | 0.034487806 | adam20;adam21 |
| protein modification process; | 9#2704 | 0.034487806 | uhrf2;herc5;tulp4;map3k9;ppm1k;flt4;herc3;melk;kit |
| fertilization; | 2#58 | 0.034487806 | adam20;adam21 |
| positive regulation of cell proliferation; | 3#233 | 0.034487806 | flt4;kit;t |
| regulation of protein kinase activity; | 3#237 | 0.034487806 | map3k9;herc5;kit |
| regulation of fatty acid beta-oxidation; | 1#2 | 0.034487806 | ppara |
| regulation of kinase activity; | 3#242 | 0.034487806 | map3k9;herc5;kit |
| biopolymer modification; | 9#2815 | 0.034487806 | uhrf2;herc5;tulp4;map3k9;ppm1k;flt4;herc3;melk;kit |
| positive regulation of MAP kinase activity; | 2#66 | 0.034487806 | map3k9;kit |
| regulation of transferase activity; | 3#247 | 0.034487806 | map3k9;herc5;kit |
| ubiquitin cycle; | 4#549 | 0.035078313 | tulp4;uhrf2;herc5;herc3 |
| gamete generation; | 3#264 | 0.035078313 | pax5;ccin;kit |
| positive regulation of cellular process; | 5#954 | 0.035078313 | med6;flt4;kit;t;ppara |
| N-acetylneuraminate metabolic process; | 1#3 | 0.035078313 | gne |
| positive regulation of fatty acid oxidation; | 1#3 | 0.035078313 | ppara |
| protein autoubiquitination; | 1#3 | 0.035078313 | uhrf2 |
| cellular macromolecule metabolic process; | 13#5656 | 0.037880194 | uhrf2;herc5;gne;tulp4;ppm1k;map3k9;adam20;flt4;herc3;melk;ttc9;adam21;kit |
| notochord development; | 1#4 | 0.041087442 | t |
| regulation of fatty acid oxidation; | 1#4 | 0.041087442 | ppara |
| regulation of MAP kinase activity; | 2#93 | 0.041087442 | map3k9;kit |
| positive regulation of biological process; | 5#1062 | 0.044306224 | med6;flt4;kit;t;ppara |
| biopolymer metabolic process; | 16#7940 | 0.044306224 | pax5;herc5;tulp4;tarbp1;ppm1k;gtf2h5;map3k9;t;gne;uhrf2;ppara;med6;melk;herc3;flt4;kit |
| UDP-N-acetylglucosamine metabolic process; | 1#5 | 0.044306224 | gne |
| protein metabolic process; | 13#5858 | 0.044353911 | uhrf2;herc5;tulp4;ppm1k;map3k9;adam20;flt4;herc3;melk;ttc9;clta;adam21;kit |
| macromolecule metabolic process; | 20#11144 | 0.047288977 | pax5;herc5;tulp4;tarbp1;ppm1k;map3k9;gtf2h5;adam20;ttc9;adam21;t;uhrf2;gne;ppara;med6;herc3;flt4;melk;clta;kit |
| positive regulation of fatty acid metabolic process; | 1#6 | 0.047288977 | ppara |
| regulation of cell cycle; | 3#359 | 0.047288977 | reck;uhrf2;herc5 |
