# Supplementary material for: Genome-wide analysis reveals signatures of selection for important traits in domestic sheep from different ecoregions
Source: BMC Genomics. 2016 Nov 3;17:863. doi: 10.1186/s12864-016-3212-2 (PMC5094087; doi:10.1186/s12864-016-3212-2)

**Additional file 23: Figure S6.** Pooled heterozygosity of Duolang sheep, *H*_P_ (in blue), and average fixation index between Mongolian sheep and Duolang sheep, *F*_ST_ (in red), plotted for 200-kb windows spanning the region harboring *TSHR* (in shadow) on chr. 7.


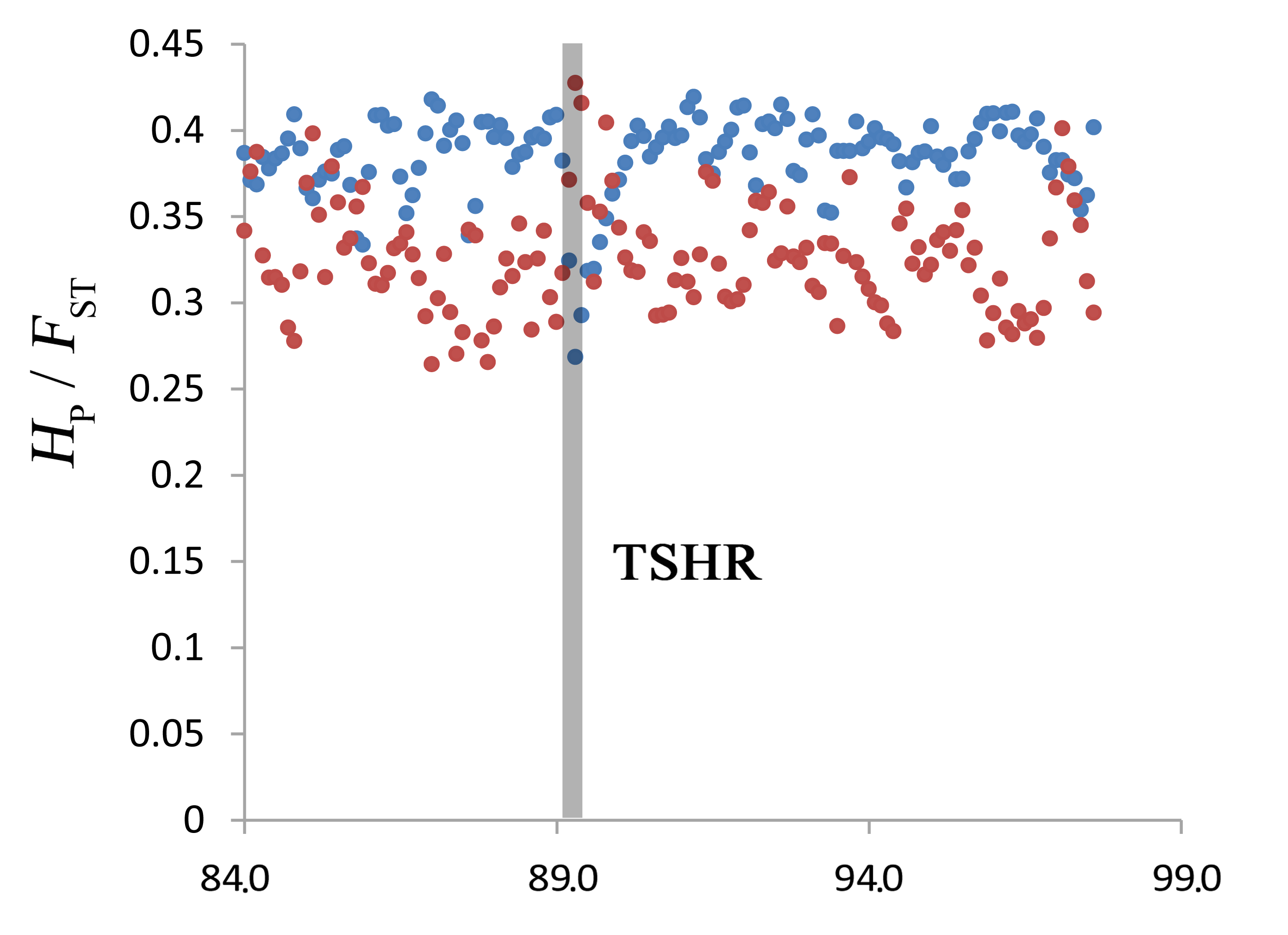

Supplement: Additional file 23: Figure S6. — Pooled heterozygosity of Duolang sheep, H P (in blue), and average fixation index between Mongolian sheep and Duolang sheep, F ST (in red), plotted for 200-kb windows spanning the region harboring TSHR (in shadow) on chr. 7. (DOC 308 kb) [file 12864_2016_3212_MOESM23_ESM.doc]
