# Supplementary material for: Genome-wide analysis reveals signatures of selection for important traits in domestic sheep from different ecoregions
Source: BMC Genomics. 2016 Nov 3;17:863. doi: 10.1186/s12864-016-3212-2 (PMC5094087; doi:10.1186/s12864-016-3212-2)

**Additional file 24: Figure S7.** Haplotype diversities of genomic regions harboring *PRL* and *HMGCR*. A and B indicate the *H*_P_ of the 3 resequenced breeds, plotted for 200-kb windows spanning the region harboring *PRL* and *HMGCR*. C and D indicate the genetic variation in the region 24.22-24.26 Mb on chr. 20 across *PRL* and *LOC443319* and the region 6.53-6.57 Mb on chr. 7 across *HMGCR*. Individual sheep (95 from 8 breeds) were genotyped using WaferGen genotyping. Dashed horizontal lines separate the 8 breeds. At the bottom of the figure, short tick marks represent individual SNPs. Long tick marks indicate the position in Mb. Red color: homozygous A-allele; green color: heterozygous; purple color: homozygous a-allele, yellow color: missing genotype call. E and F indicate the haplotype frequencies of the 8 sheep breeds at the 2 genomic regions.


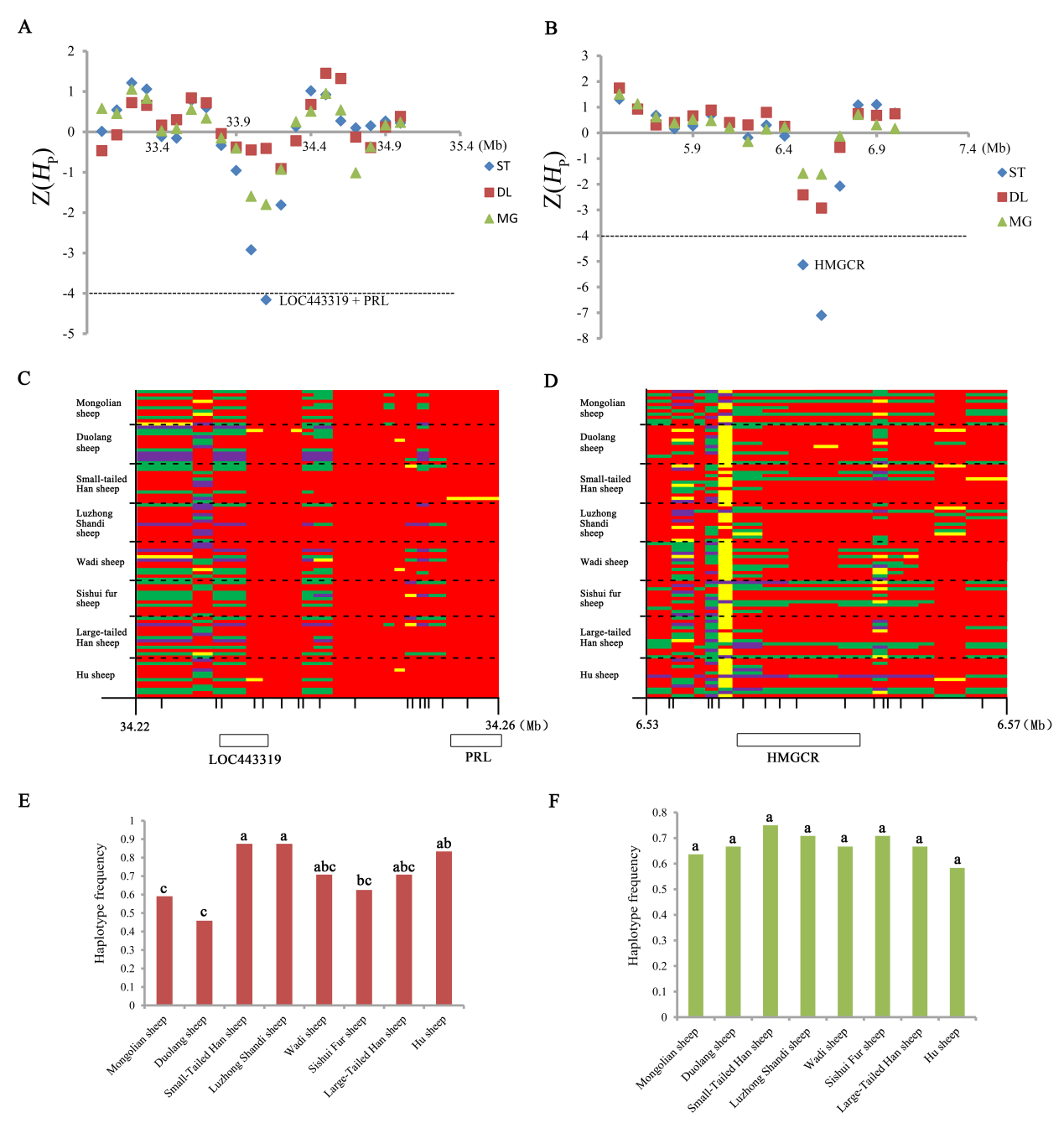

Supplement: Additional file 24: Figure S7. — Haplotype diversities of genomic regions harboring PRL and HMGCR. A and B indicate the H P of the 3 resequenced breeds, plotted for 200-kb windows spanning the region harboring PRL and HMGCR. C and D indicate the genetic variation in the region 24.22–24.26 Mb on chr. 20 across PRL and LOC443319 and the region 6.53–6.57 Mb on chr. 7 across HMGCR. Individual sheep (95 from 8 breeds) were genotyped using WaferGen genotyping. Dashed horizontal lines separate the 8 breeds. At the bottom of the figure, short tick marks represent individual SNPs. Long tick marks indicate the position in Mb. Red color: homozygous A-allele; green color: heterozygous; purple color: homozygous a-allele, yellow color: missing genotype call. E and F indicate the haplotype frequencies of the 8 sheep breeds at the 2 genomic regions. (DOC 214 kb) [file 12864_2016_3212_MOESM24_ESM.doc]
