# Supplementary material for: Genome-wide analysis reveals signatures of selection for important traits in domestic sheep from different ecoregions
Source: BMC Genomics. 2016 Nov 3;17:863. doi: 10.1186/s12864-016-3212-2 (PMC5094087; doi:10.1186/s12864-016-3212-2)
Supplement: Additional file 25: Table S18. — The H P and F ST within BMPR1B and MTNR1A. (DOC 32 kb) [file 12864_2016_3212_MOESM25_ESM.doc]

***Additional file 25: Table S18.*** *The H_P_ and F_ST_ within BMPR1B and MTNR1A*

|  | BMPR1B | MTNR1A |
| --- | --- | --- |
| *H*_P_-Mongolian sheep | 0.4024 | 0.4048 |
| *H*_P_-Small-tailed Han sheep | 0.4147 | 0.4377 |
| *H*_P_-Duolang sheep | 0.4017 | 0.4033 |
| *F*_ST_ between Mongolian sheep and Small-tailed Han sheep | 0.2595 | 0.2463 |
| *F*_ST_ between Mongolian sheep and Duolang sheep | 0.2498 | 0.2691 |
| *F*_ST_ between Duolang sheep and Small-tailed Han sheep | 0.2798 | 0.2192 |
