# Supplementary material for: Genome-wide analysis reveals signatures of selection for important traits in domestic sheep from different ecoregions
Source: BMC Genomics. 2016 Nov 3;17:863. doi: 10.1186/s12864-016-3212-2 (PMC5094087; doi:10.1186/s12864-016-3212-2)
Supplement: Additional file 6: Table S5. — Enriched GO terms among genes containing missense SNPs or stop gained/loss variants in Mongolian sheep but not in Small-tailed Han sheep or Duolang sheep. (DOC 673 kb) [file 12864_2016_3212_MOESM6_ESM.doc]

**Additional file 6: Table S5**. Enriched GO terms among genes containing missense SNPs or stop gained/loss variants in Mongolian sheep but not in Small-tailed Han sheep or Duolang sheep.

| GO term | Gene count | P value | genes |
| --- | --- | --- | --- |
| developmental process; | 422#3347 | 1.99923E-49 | kiaa1967;prickle2;ikzf1;sap30bp;cops3;pcdh18;hoxd1;tshz3;dmc1;mark2;myl4;nr4a2;prkra;tyr;tfip11;dkk2;plekhf1;pparg;fhl1;bcl2l11;fez1;nr5a1;tnfrsf25;ablim1;cdc42bpb;il3;pdcd2;sqstm1;traf7;cdc42ep5;neurod6;myl1;axin2;tbcb;sparc;rbm45;ciapin1;glis2;ets2;pcdh1;capg;vsx1;cdx2;lalba;iqcb1;aplp1;cylc1;gna13;serpinf1;rasa1;plp1;gp1ba;gnpat;nrp1;dpysl3;cd70;edn1;traf3;cdh13;gfra3;myog;sfrp2;ern1;keap1;cd2;donson;thbd;clic4;thoc5;elmo1;dlx2;dip2a;dnm1l;tshb;msx1;pou3f2;adam12;tlr2;vps33a;dffa;arhgdia;kcne1;sox9;pou4f3;asah2;map4k2;eif4g2;lrp6;kcnmb4;mobp;kcnq2;klk7;snai1;alx1;sirt1;cck;twist1;gsk3b;nrg3;adora2b;hey2;nck1;trim54;shc1;traf6;spata9;alox12;aldh5a1;srd5a1;vax1;trib3;uhrf2;atrnl1;fgf1;akap4;ca9;hoxa3;sema4d;jmjd6;fgd1;net1;prkca;nras;mmp9;ccin;fgf6;bcl6;gsc;apip;adam10;cyfip1;chrm2;tlx3;b3gnt2;tinag;stat3;pcdhb6;wnt5b;adm;maff;socs1;brms1l;wnt11;doc2a;snrk;wnt2;dlx5;pth2r;mmd;gaa;mlxipl;chrna4;vegfa;foxc1;ripk2;mbp;lyz;tgfb2;hoxd8;azu1;il20;mpped2;znf346;kif3b;spock1;ing3;madd;bclaf1;pdgfc;whsc2;ihh;nr5a2;ngfr;igf2bp3;rhcg;mcam;cux1;nde1;zbtb16;hoxc9;six6;igfbp6;ppp2r2b;malt1;nle1;prkaa1;eif2ak2;rassf5;papss1;ccar1;cln5;ostf1;optn;hells;prrx1;srpk1;cdk6;sgcg;hoxc12;mrpl40;trpc5;hcls1;bub1b;hcn4;scml1;fgf20;rufy3;pts;plagl2;drp2;hexa;trim13;ift88;runx3;pax8;sectm1;hunk;cd14;meox2;amtn;eya3;actn4;dscaml1;pogk;asz1;foxn1;dnm2;camk2d;ikzf3;sox15;sema3a;chst8;capza3;ripply1;klf1;dctn1;comp;sema3d;mdga2;dazap1;vdac1;evi5;taok2;gas2;cdkn2aip;casp7;pcdhb5;pdcd5;scmh1;pim1;dcbld2;phc3;ndufs1;tp53bp2;arhgef6;cited2;ap3d1;tgm5;tnp1;itgb1bp2;fgf5;rs1;ebag9;krt13;ldb2;shroom2;gja1;scrg1;cdkn2d;piwil3;tbx10;traf1;slc5a3;sox6;trim3;birc3;atp2c1;plod1;slitrk3;nlrp2;crim1;arhgdib;npr3;clec3b;prkci;pak1;nanog;ctnna1;dhrs9;atp2a2;tgfb1;rqcd1;mapk1;spin1;mid1;dusp22;ppp1r15a;mut;fgf22;acvr2b;cda;prelp;ghsr;cdkl2;sgca;tfap2c;pdcd4;pcdhb11;drgx;adra1b;efnb3;vgf;ankrd2;clcf1;egln2;rragc;minpp1;mixl1;hus1;eya4;pdia2;ctnnb1;fxyd5;matn1;prdx1;bnipl;tnfaip3;bmf;kng1;mdga1;pafah1b3;krt5;rax;lep;bpgm;ugcg;b3gnt5;scml2;hdac9;flt1;btk;krt20;ift81;spry1;dcc;ipmk;nrcam;adamtsl4;bcl7c;hspb1;utp3;hoxb3;npas1;il17a;gpx4;pmch;nts;dedd;tnp2;eef1e1;sgpl1;pak3;fzd9;clul1;klf5;slitrk4;htra1;rhob;cep57;hspd1;faim;sema4a;crem;tns4;hps6;krt9;ltbr;cd28;npm2;kiaa1598;cd74;fzd5;sim2;cdc42ep4;egr2;sfn;elf3;th;mcl1;crx;kitlg;pacs2;sgcb;eln;krt1;cacna1f;bik;ing1;onecut2;gzf1;tspyl2;ca2;alb;slc30a1;wif1;creg1;stat1;atg12;wisp1;psmc5;cd164;ndrg2;runx1;gch1;dlx3;esm1;bves;sycp3;tax1bp1;thbs4;adad1;eda2r |
| anatomical structure development; | 264#2005 | 2.1069E-33 | prickle2;ikzf1;pcdh18;dmc1;mark2;myl4;nr4a2;prkra;tfip11;fhl1;fez1;nr5a1;ablim1;cdc42bpb;il3;traf7;cdc42ep5;neurod6;myl1;tbcb;sparc;rbm45;glis2;ets2;pcdh1;capg;cdx2;iqcb1;aplp1;gna13;serpinf1;rasa1;plp1;gp1ba;gnpat;nrp1;dpysl3;edn1;cdh13;gfra3;myog;keap1;cd2;dlx2;tshb;msx1;pou3f2;adam12;vps33a;kcne1;sox9;pou4f3;map4k2;kcnmb4;mobp;kcnq2;klk7;snai1;alx1;sirt1;cck;twist1;nrg3;adora2b;hey2;nck1;shc1;alox12;aldh5a1;fgf1;akap4;ca9;sema4d;jmjd6;fgd1;net1;nras;mmp9;fgf6;bcl6;gsc;cyfip1;chrm2;tlx3;b3gnt2;tinag;stat3;pcdhb6;adm;maff;socs1;brms1l;wnt11;doc2a;snrk;wnt2;dlx5;pth2r;gaa;mlxipl;chrna4;vegfa;foxc1;mbp;tgfb2;azu1;il20;mpped2;spock1;ing3;pdgfc;ihh;nr5a2;ngfr;igf2bp3;rhcg;mcam;cux1;nde1;zbtb16;igfbp6;six6;nle1;papss1;ostf1;cln5;cdk6;sgcg;mrpl40;trpc5;hcls1;scml1;fgf20;rufy3;pts;drp2;hexa;trim13;pax8;ift88;sectm1;amtn;eya3;asz1;dscaml1;foxn1;camk2d;ikzf3;sox15;sema3a;chst8;klf1;dctn1;comp;sema3d;mdga2;taok2;gas2;cdkn2aip;pcdhb5;scmh1;dcbld2;cited2;ap3d1;tgm5;itgb1bp2;fgf5;ebag9;krt13;gja1;shroom2;scrg1;cdkn2d;slc5a3;tbx10;sox6;trim3;atp2c1;plod1;slitrk3;crim1;npr3;clec3b;prkci;ctnna1;dhrs9;tgfb1;atp2a2;mapk1;mid1;cda;prelp;ghsr;sgca;pcdhb11;adra1b;efnb3;ankrd2;vgf;clcf1;rragc;egln2;minpp1;eya4;fxyd5;ctnnb1;matn1;prdx1;kng1;pafah1b3;mdga1;krt5;lep;bpgm;ugcg;b3gnt5;flt1;hdac9;scml2;btk;spry1;nrcam;ipmk;dcc;utp3;hoxb3;npas1;nts;klf5;fzd9;slitrk4;htra1;rhob;sema4a;krt9;cd74;fzd5;sim2;cdc42ep4;egr2;sfn;elf3;th;crx;kitlg;sgcb;eln;krt1;cacna1f;ing1;onecut2;gzf1;ca2;tspyl2;creg1;wisp1;ndrg2;gch1;runx1;dlx3;bves;esm1;thbs4;eda2r |
| cellular process; | 1535#19591 | 1.01583E-31 | ttbk1;stx10;fkbp3;chml;prickle2;rsu1;ikzf1;amica1;nox1;fbxo10;tigd3;usp30;mybpc2;hoxd1;myo3a;psmc2;znf566;fut1;scarb2;traf3ip2;lrp10;fhl1;pde11a;klf11;e4f1;sec61a2;tec;necap1;plek;angptl5;il3;naprt1;pdcd2;plod2;adam20;vps26b;adra2b;arih1;st3gal3;phkg2;rbm45;rbbp6;glis2;pstk;vsx1;cdx2;lalba;apobec2;aplp1;hgsnat;serpinf1;rasa1;gnpat;kpna2;edn1;traf3;akt2;cdh13;myog;xpo5;mex3b;iqgap2;rspo1;cstf1;calr3;nrxn1;cdh16;cntrob;thoc5;rpl8;cno;wdr36;opcml;mrpl44;rbm23;abl2;msx1;zbtb25;vps33a;dyrk1b;usp15;fbxl8;kcne1;snx27;impa2;map4k2;eif4g2;kremen1;pkd2l1;klk7;snai1;pex12;alx1;tfrc;sirt1;gin1;hectd2;gabbr1;mrpl3;f7;h2afz;dcbld1;ccr5;rom1;fibcd1;elf5;spata9;lysmd3;ran;ykt6;tbcc;srd5a1;trib3;cdc14b;nfkbib;asgr2;hsp90aa1;jmjd6;nxf1;fubp3;pdxdc1;net1;zbtb32;l2hgdh;nras;tmub2;ints5;ppm1d;bcl6;senp2;gsc;apip;kiss1r;gnb2;usf1;rabep2;pcdhb6;camk4;tgfbi;wnt5b;gpr55;c4bpb;socs1;cbfa2t2;oxgr1;ddb1;gnat3;mmd;mlxipl;fbxo45;stac;kpna6;xrcc2;azu1;osbpl2;ezh2;znf346;pnoc;ctsw;ppic;tsfm;nbl1;yap1;ihh;ngfr;igf2bp3;tspan2;rhcg;mcam;nde1;proca1;igfbp6;islr;ppp2r2b;irx3;sfpq;slbp;znf581;cln5;optn;cdk6;skap2;fance;insig1;hcls1;creb3;gns;pds5a;inpp1;ndufv1;pts;hyal2;senp8;dph1;pygl;ift88;sectm1;hunk;srgap3;daam2;utp6;mrpl30;prepl;kif9;ckm;eya3;susd5;dscaml1;kcnk2;cpa5;prkag2;asb2;pitpnc1;rapgef4;ikzf3;camk2d;dnm2;tmod1;hamp;apeh;ca3;scamp2;depdc7;dnm3;zscan21;sergef;comp;mmab;adprh;gle1;hadha;tktl1;csnk2b;pvrl2;syncrip;esrrg;galns;cdkn2aip;pim1;tp53bp2;samd4a;coro1a;omg;cited2;ap3d1;hibadh;galnt6;ulk3;adat1;zbtb20;qpctl;ebag9;duxa;mpp1;shroom2;atg7;zfx;med8;traf1;sp2;atp2c1;nlk;galnt9;rab3c;ppp4c;syvn1;znf496;pygo1;kif3a;prkci;pak1;tp53rk;tgfb1;ebna1bp2;mapk1;dusp22;ace2;slmap;elk4;dync1i1;ghsr;rps29;gucy2c;cnih4;gprc5d;pkp3;cad;pcdhb11;drgx;tubg1;efnb3;slc23a2;ppp1cc;tceal8;aldh1a1;timp3;rnf41;egln2;mixl1;tox4;hus1;dhx15;tysnd1;matn1;polr2f;znf354a;capza2;znf451;grsf1;mrpl16;fa2h;snx25;arhgap1;sc5dl;rtp4;lep;bpgm;scnn1b;tspyl1;plaur;tgds;usp2;nrcam;pnpla3;pglyrp3;adamtsl4;bcl7c;scarf1;gpr19;trhr;ppargc1b;taok3;nts;mrpl37;oma1;pias1;set;ccr2;clul1;klf5;rab8a;slitrk4;tnip1;htra1;rhob;hspd1;lrrc29;snx14;agtr1;ltbr;mysm1;entpd1;tead1;nap1l5;sim2;lgals9;egr2;gfpt1;mrpl47;rad54l;mcl1;atp6v1g3;pgd;ampd1;pacs2;eln;eif3e;tpm4;sarm1;ing1;slamf1;onecut2;scap;ttc1;ncaph;ptges;alb;fbxo47;atg12;stat1;mln;pycr1;napg;mns1;gdpd3;ndrg2;runx1;ldhc;slc6a5;sycp3;gosr1;mrpl35;eda2r;ncapg;foxp4;sap30bp;ears2;spcs2;znf558;qprt;dusp10;znf33b;nr4a2;tyr;tfip11;negr1;uts2;map3k7;fkbp6;ndc80;cdk2ap1;plekhf1;pparg;cdc16;cadm3;sumo1;znf414;ctsa;atp5o;vps72;fbxl17;gabrb2;hapln3;afmid;ablim1;rgs17;cdc42bpb;b3gnt4;ramp1;sqstm1;traf7;neurod6;pak1ip1;sparc;hook3;ddah1;cldn14;prmt2;pcdh1;iqcb1;cylc1;gna13;rasa4;csnk1g3;cxcl14;plp1;abcf1;cpa3;acsm5;strn4;chaf1a;ikzf4;acadl;ern1;cxadr;rasl11a;cd2;med20;paics;kynu;cpxm1;foxj3;mars2;ampd3;des;zfp42;ccnt2;pwp1;nupl2;atf3;tshb;pou3f2;rbm14;tlr2;cdc37;arhgdia;cnih2;znf214;sox9;wars2;utf1;pou4f3;pag1;ppm1k;lrp6;ugdh;kcnmb4;vgll4;pcsk7;pou2f1;sncb;polr2d;mrps18b;znf605;pja2;prkaa2;gsk3b;diras3;gtf2e1;coro2a;hey2;nck1;trim54;neu3;sdha;soat1;shc1;rasgrp2;psmd14;mrpl10;aldh5a1;lrrk1;uso1;tmem115;dnajc17;iqsec2;tagap;akap4;gpr142;hoxa3;sema4d;pygo2;dera;mvd;rgs11;b3galt5;wars;slc25a10;prkca;nhp2l1;plcz1;pram1;mthfd1l;arfgap1;npffr2;mettl1;adam10;gnai3;tubgcp3;sh2b1;znf175;sugt1;grip1;tmem48;taf4;ajap1;adm;cops2;sohlh2;wnt11;usp11;sntg1;dlx5;araf;vegfa;slc7a7;papd5;eid2;fbxo9;cdkn3;dsc2;tbc1d14;cldn2;slc30a9;med18;lrpap1;kif3b;nxph4;sf3b2;ppie;ing3;pdgfc;cd93;whsc2;nr5a2;taf6;agxt2l1;lrrc14;qdpr;six6;cmas;fbxw8;malt1;nle1;klhl2;rassf5;papss1;picalm;hars;sp5;hells;ctnna3;zbtb12;sec24b;sgcg;hoxc12;klf15;osbpl7;chst1;aurkb;shank1;plagl2;drp2;hexa;ccr9;runx3;pax8;lcorl;fbln5;upf3a;amtn;actn4;foxn1;neil1;map3k9;drd3;arfip1;supt16h;chst8;klf1;hpse;nfkbil1;sms;dcp1a;pdss2;pepd;lancl2;nr0b2;nfatc2ip;casp7;htr4;sh3pxd2a;dbnl;znf786;ldha;pdcd5;sars;b3galt2;ptpn21;senp1;herc6;eif4h;arfgef2;aipl1;crnkl1;pigu;cfi;prdm12;eef1d;il1rap;copg2;cdkn2d;rbck1;chst12;sox6;birc3;pcna;aloxe3;setdb2;atp6v0b;sirt5;hnrnpl;arhgdib;pknox1;skiv2l2;ddx19b;nusap1;imp4;atp2a2;axl;rad51;ppp1r15a;gpr3;gpr139;klhl15;aco1;mark1;atp5b;cdc27;tfap2c;trpv5;fbxo38;aldh7a1;slc38a4;mtnr1b;hif3a;snip1;clcf1;pgk2;pnn;rragc;aurkaip1;anapc7;lin7a;idh3a;prdx1;gga3;cgref1;twf1;elovl7;asphd1;lrrc41;grhl2;rcc1;dgka;scml2;ilf2;btk;rem1;ppp1r1b;ift81;psma8;mfap4;rps3;spint2;rpp30;pole3;nt5c1a;npas1;farsa;ftsj1;pmch;tnp2;grb10;sgpl1;psmb5;rnpc3;cabp4;krba2;alg3;pip4k2a;usp33;rab42;st3gal4;rasal2;znf689;sema4a;mchr1;tns4;chrne;gpr88;dnali1;cd74;znf131;cdc42ep4;pigb;rassf8;fstl3;elf3;wee2;parvb;crx;trub2;znf398;vav3;kitlg;gan;snd1;dmpk;bik;rpl9;fbp2;gtf2a1;acly;stxbp1;mapk8ip1;jak1;tubb6;ca2;rsl1d1;nab1;col8a2;hk3;gnal;epc2;nuak1;cd164;zbtb38;pdha1;gch1;nxph2;nars;cdt1;dot1l;gmps;kcna5;dbh;scnm1;kiaa1967;pigq;snx15;exosc10;rgs4;cfb;pla2g12a;mcee;pcdh18;nrbp1;tshz3;nsf;slc27a2;msrb3;gphn;arg1;sucla2;st13;arhgap9;grap2;cdk9;tspan8;sf3a2;chst14;hif1an;axin2;ccl28;tbcb;st6galnac4;rrm2b;plekho1;clec1a;pop5;ets2;rnf139;gpr82;znf41;pcdhb7;sirt3;eif3f;zbtb33;phka2;dpysl3;nrp1;pcdhga2;cd70;smad6;gfra3;sfrp2;stx4;runx1t1;xpo6;uchl5;prdm14;snrpe;clic4;mki67ip;usp39;zxdc;elmo1;tpm1;usp32;akap8;aprt;rtp3;arpc1b;dcp2;adam12;rdbp;dffa;smap1;bace2;zcrb1;asah2;nr4a1;mrps35;supt4h1;kcnq2;rgs21;ldhb;arhgap21;usp13;twist1;gnb1;rbl2;adora2b;bhmt;gfra2;traf6;ogt;galr1;ms4a7;epha3;irf5;ncam2;pcbp3;sdf2;ucn2;tst;diras1;lats2;ldlrap1;myd88;cables1;fbxl22;ca9;hdac10;cldn17;st6gal1;fgd1;tcf23;phkg1;zbtb45;ccnd3;flrt2;mmp9;dlst;ccin;slc6a2;sec61a1;angpt4;atf7;adam21;stard5;grpel2;sp1;stat3;gnpda1;pfkfb2;cpb1;maff;atp6v1d;brms1l;znf501;b3galt1;rae1;snrk;pdc;clk3;pth2r;junb;chrna4;gpr68;foxc1;ripk2;lyz;pgam2;htr1a;hoxd8;tgfb2;cbs;spock1;clcn5;meis3;bclaf1;kpna3;mrps9;me2;cux1;hoxc9;zbtb16;znf768;pacsin1;polh;mtf1;mag;ccar1;dio3;mmp12;psd4;serinc1;prrx1;srpk1;trpc5;hcn4;bub1b;trmu;rufy3;rnf2;sphar;arv1;arhgap15;cd14;klk13;ankrd27;ssrp1;mrps18c;pogk;slc20a1;brs3;cdca7;sema3a;capza3;cx3cl1;ripply1;gpaa1;g6pd;nup35;hmgb4;sh3rf2;dctn1;mkl1;rpgr;tox3;inppl1;vdac1;taok2;znf446;rcor2;rfx5;tlk1;pcdhb5;acsl3;rapgef1;tox;ndufs1;ddr2;zdhhc7;trim38;gpr84;fen1;fgf5;ccnb1ip1;zcchc2;ttll5;dek;man1a1;maoa;gper;rabggta;ppp4r2;ap3s1;tbx10;mrpl22;rgs1;mtap;plod1;dnah12;slitrk3;chdh;enpp2;pbrm1;tor2a;gpr128;znfx1;gnptg;ppp1cb;pam;eif1ax;map3k2;ctnna1;fdps;trpv1;fgf22;trip13;exoc3;ppm1l;gpr174;hacl1;cdyl2;cdkl2;phtf1;klk9;pdgfd;slc27a4;ncoa2;adra1b;mia;ppp2r5b;cct6b;mfge8;mgat4a;ogdh;bckdhb;eif5;amd1;uap1;arl6;stac3;eya4;cxcl16;ctnnb1;elavl2;fxyd5;bnipl;scrn1;uqcrh;mdga1;rax;efna2;rpp14;l3mbtl3;rdh11;tubgcp5;stx16;hdac9;flt1;krt20;cpa6;rundc3a;dcc;mapkapk5;rnps1;utp3;uroc1;hoxb3;adat3;rps10;il17a;nedd4l;csnk1a1;gna12;gpx4;eef1e1;fzd9;scfd1;ranbp9;agmat;cep57;cercam;cdk10;tsc22d4;ralgps1;hps6;krt9;spaca3;pigs;elf1;ppp1r12b;ugt1a9;rad23a;gabpb2;th;cnga3;trappc1;hectd3;dhx38;pcgf1;slc25a17;a4gnt;aoah;sgcb;cd34;krt1;cacna1f;cdh12;olr1;arid5a;srms;atg4a;adk;upp1;cacnb3;slc30a1;creg1;wisp1;c1qtnf2;psmc5;aldh1l1;arhgap28;dlx3;esm1;alkbh1;impa1;capn2;acadsb;tax1bp1;thbs4;fasn;ptk2;pde1a;ppil3;acaca;cops3;izumo1;rbm3;dmc1;mark2;prkra;znf548;dkk2;map3k4;cxcr7;fdxr;vps16;iqsec3;c1qc;ctss;bcl2l11;lcor;glyctk;acox2;fez1;gab1;tomm22;nr5a1;tnfrsf25;rce1;acoxl;cdc42ep5;fancf;gmeb2;cds1;ptpn18;chd3;ciapin1;pabpc4;capg;kifc3;fubp1;klk12;n6amt2;gp1ba;gpr124;rrp9;znf664;keap1;nfatc2;zfp2;cldn1;fech;cnbp;eif1b;mtmr7;npr2;cd53;sesn1;arl16;hnrpll;dlx2;polr3c;hmgn2;ptpla;fbxl4;dnm1l;sord;flrt3;bcl11b;fscn1;gdpd1;rab10;rab37;aurkc;tgm7;rab33a;dpp8;lonp1;pdss1;gfpt2;cck;clock;nrg3;cdc73;xpr1;ddx54;elk1;znf546;cnot6;alcam;srm;sucnr1;ero1lb;alox12;med23;vax1;znf197;klf12;serpina5;uhrf2;fgf1;ubqln4;srp72;ctbp2;abcd3;stx7;dgkb;cpa4;fgf6;ssu72;snph;senp3;chrm2;cyfip1;ap1s3;cept1;tlx3;b3gnt2;ftsj2;tinag;ap1g1;zfp1;nubp1;vps41;scn1b;psap;doc2a;znf710;wnt2;ccrl1;ube2m;oxsr1;gaa;grid2;mrpl36;pank4;ndufb9;dus1l;mbp;tfe3;bub3;rpp38;fbxl13;il20;scd;st3gal1;itk;lonrf1;rps8;madd;qsox2;hapln2;tinf2;slc8a2;rnf14;arhgef9;zscan29;prkaa1;kif18a;eif2ak2;abi2;map4k3;ostf1;snx22;cysltr2;hdx;znf771;pum2;cldn20;stag1;scml1;arl4c;rbm17;ero1l;vsig4;fgf20;agk;rarb;rrm2;pja1;trim13;ovol2;ugt1a6;brf2;tpst1;acot9;habp4;meox2;ict1;asz1;znf524;grm8;sox15;bcat2;icam5;znf787;sema3d;mdga2;dazap1;evi5;dgkz;pde1c;gas2;pex16;appl1;vrk1;pdk2;scmh1;dcbld2;znf623;znf688;chrna5;arhgef6;mttp;vapb;sh2d3c;ywhab;ocrl;prps2;hdgf;psph;tgm5;tnp1;itgb1bp2;tnks;gp6;cd274;rexo2;rs1;gja1;trim24;gabpa;mrpl27;apobec1;uimc1;tacr3;rbm5;crim1;nlrp2;fut3;ltb4r;mad2l2;srp54;npr3;nanog;dhrs9;nxph3;adam32;gcnt4;stac2;spin1;mid1;pld2;map2k7;lcmt1;acvr2b;cda;dusp13;fip1l1;vps26a;pdcd4;ilf3;prpf6;adrm1;opn1sw;clp1;znf24;stk39;ccr8;cnot4;ubtd2;nek8;minpp1;slc17a4;spsb3;ephx1;pdia2;ccr7;tnfaip3;bmf;kng1;abt1;synj2bp;rgs22;b3gnt5;ugcg;camk2g;ctcf;tcea3;cckbr;exosc7;spry1;isca1;ipmk;nxn;hspb1;gfer;ncln;cxcl11;zmat5;pcgf2;cryl1;cndp2;rplp1;dedd;nmbr;isg20;sh3bp1;tbpl2;areg;pak3;cdc40;il13;hadh;crebzf;trub1;smc5;cog2;faim;crem;nlgn4x;casz1;terf2;cog5;ca13;dck;ttll9;sirt4;cd28;kif1c;npm2;fzd5;ccng1;sox5;prdm4;sfn;mkln1;hnrnpr;snx5;dbx2;atf2;znf454;nr6a1;prtn3;epha1;igsf6;cxxc4;med24;tubgcp4;cnksr1;pskh1;gzf1;ubqln2;lman1;tspyl2;igsf1;wif1;pxk;nsun2;rfx3;brsk1;cstf2t;denr;lmbr1l;eif3i;trove2;rab9b;arl9;mecp2;sac3d1;sgol1;pnmt;st8sia5;dnaja4;rnaseh1;adad1;rad1;fbxl6 |
| biological regulation; | 651#6731 | 1.03223E-30 | chml;rgs4;ikzf1;cfb;tigd3;hoxd1;tshz3;znf566;traf3ip2;fhl1;klf11;cdk9;hif1an;il3;ccl28;plekho1;glis2;pstk;rnf139;ets2;vsx1;cdx2;lalba;aplp1;serpinf1;znf41;rasa1;sirt3;eif3f;zbtb33;nrp1;cd70;kpna2;smad6;edn1;traf3;cdh13;myog;runx1t1;prdm14;clic4;iqgap2;zxdc;tpm1;abl2;msx1;rdbp;zbtb25;vps33a;dyrk1b;dffa;kcne1;smap1;bace2;eif4g2;nr4a1;supt4h1;alx1;rgs21;tfrc;sirt1;gabbr1;f7;rbl2;twist1;adora2b;bhmt;traf6;galr1;elf5;irf5;ran;lats2;trib3;ldlrap1;myd88;cables1;hdac10;hsp90aa1;fgd1;tcf23;fubp3;zbtb45;ccnd3;serpinc1;net1;zbtb32;nras;mmp9;ppm1d;bcl6;senp2;gsc;kiss1r;usf1;atf7;stat3;sp1;tgfbi;c4bpb;maff;socs1;brms1l;znf501;cbfa2t2;ddb1;pth2r;junb;mlxipl;foxc1;ripk2;htr1a;tgfb2;hoxd8;azu1;ezh2;meis3;bclaf1;nbl1;yap1;ihh;ngfr;igf2bp3;rhcg;cux1;zbtb16;hoxc9;znf768;igfbp6;polh;irx3;sfpq;mtf1;znf581;cln5;dio3;slc9a7;serinc1;psd4;prrx1;srpk1;cdk6;skap2;hcls1;bub1b;hcn4;creb3;rnf2;dph1;ift88;sectm1;ssrp1;eya3;pogk;slc20a1;rapgef4;brs3;ikzf3;dnm2;camk2d;cdca7;hamp;capza3;cx3cl1;ripply1;zscan21;sergef;hmgb4;mkl1;tox3;procr;taok2;esrrg;znf446;rcor2;rfx5;cdkn2aip;tlk1;pim1;tox;rapgef1;ndufs1;tp53bp2;samd4a;ddr2;cited2;trim38;ap3d1;zbtb20;ebag9;dek;duxa;maoa;atg7;zfx;tbx10;traf1;med8;sp2;rgs1;atp2c1;nlk;pbrm1;znfx1;znf496;pak1;map3k2;ctnna1;tgfb1;mapk1;als2cr12;dusp22;elk4;ghsr;phtf1;mrcl3;ncoa2;drgx;adra1b;tceal8;eif5;egln2;mixl1;tox4;eya4;hus1;ctnnb1;fxyd5;elavl2;slc22a4;bnipl;polr2f;znf354a;capza2;rax;znf451;arhgap1;lep;l3mbtl3;scnn1b;plaur;flt1;hdac9;krt20;dcc;nrcam;adamtsl4;hoxb3;tfpi2;ppargc1b;kcnq1;taok3;nedd4l;gna12;nts;gpx4;eef1e1;pias1;set;ccr2;klf5;slc9a8;rab8a;tnip1;htra1;rhob;cdk10;hspd1;tsc22d4;agtr1;ltbr;mysm1;entpd1;tead1;spaca3;kcnj12;sim2;ppp1r12b;elf1;lgals9;egr2;gabpb2;th;mcl1;hectd3;pcgf1;aoah;krt1;cacna1f;eif3e;ing1;arid5a;slamf1;onecut2;scap;cacnb3;slc30a1;alb;stat1;creg1;wisp1;psmc5;c1qtnf2;napg;dlx3;runx1;esm1;sycp3;tax1bp1;eda2r;foxp4;sap30bp;znf558;znf33b;prkra;nr4a2;znf548;dkk2;map3k7;uts2;map3k4;plekhf1;cdk2ap1;c1qc;iqsec3;pparg;cdc16;bcl2l11;sumo1;znf414;lcor;vps72;gab1;nr5a1;tnfrsf25;rgs17;cdc42bpb;ramp1;sqstm1;traf7;neurod6;cdc42ep5;pak1ip1;gmeb2;chd3;ciapin1;pabpc4;capg;fubp1;iqcb1;gna13;rasa4;plp1;gp1ba;chaf1a;ikzf4;ern1;znf664;keap1;cd2;med20;thbd;nfatc2;zfp2;eif1b;cnbp;npr2;sesn1;polr3c;dlx2;hmgn2;foxj3;des;zfp42;ccnt2;atf3;pou3f2;rbm14;cdc37;tlr2;arhgdia;znf214;bcl11b;sox9;utf1;pou4f3;pag1;rab10;rab37;kcnmb4;vgll4;pou2f1;sncb;clock;znf605;gsk3b;nrg3;gtf2e1;diras3;hey2;cdc73;trim54;nck1;ddx54;elk1;shc1;znf546;cnot6;rasgrp2;alox12;med23;aldh5a1;vax1;klf12;znf197;uhrf2;serpina5;iqsec2;hoxa3;sema4d;rgs11;ctbp2;wars;dgkb;prkca;pram1;snph;arfgap1;cyfip1;chrm2;adam10;gnai3;tlx3;znf175;grip1;zfp1;taf4;adm;sohlh2;znf710;wnt2;dlx5;ube2m;vegfa;eid2;mbp;tfe3;bub3;cdkn3;tbc1d14;med18;slc30a9;il20;ppie;ing3;madd;qsox2;whsc2;cd93;nr5a2;taf6;rnf14;six6;arhgef9;malt1;eif2ak2;ecsit;prkaa1;zscan29;rassf5;hdx;znf771;sp5;f3;hells;zbtb12;hoxc12;klf15;pum2;scml1;vsig4;fgf20;agk;rarb;plagl2;hexa;ccr9;trim13;ovol2;pax8;runx3;brf2;lcorl;habp4;meox2;actn4;asz1;foxn1;map3k9;znf524;grm8;supt16h;sox15;arfip1;klf1;znf787;nfkbil1;evi5;nr0b2;dgkz;lancl2;gas2;nfatc2ip;znf786;dbnl;pdcd5;scmh1;dcbld2;znf623;znf688;arhgef6;vapb;tnp1;gp6;cd274;eif4h;arfgef2;pigu;cfi;gja1;prdm12;eef1d;cdkn2d;trim24;gabpa;uimc1;slc5a3;sox6;pcna;birc3;rbm5;crim1;nlrp2;sirt5;arhgdib;mad2l2;pknox1;npr3;nusap1;nanog;slc9a5;atp2a2;rad51;pld2;ppp1r15a;cda;acvr2b;tfap2c;pdcd4;ilf3;prpf6;hif3a;znf24;snip1;clcf1;ccr8;cnot4;pnn;rragc;aurkaip1;lin7a;pdia2;ccr7;cgref1;tnfaip3;kng1;abt1;synj2bp;grhl2;rcc1;rgs22;dgka;scml2;ilf2;btk;ctcf;tcea3;cckbr;spry1;nxn;hspb1;apoh;pcgf2;npas1;dedd;tbpl2;krba2;crebzf;faim;rasal2;znf689;mchr1;crem;casz1;terf2;chrne;cd28;sirt4;npm2;cd74;znf131;cdc42ep4;ccng1;sox5;prdm4;fstl3;sfn;elf3;znf398;crx;vav3;dbx2;atf2;znf454;nr6a1;prtn3;snd1;cxxc4;med24;dmpk;bik;gtf2a1;mapk8ip1;gzf1;lman1;tspyl2;igsf1;rfx3;nab1;pxk;brsk1;gnal;epc2;eif3i;cd164;zbtb38;gch1;mecp2;cdt1;kcna5;rad1 |
| negative regulation of biological process; | 175#1182 | 7.95588E-30 | rgs4;actn4;asz1;grm8;prkra;sox15;dkk2;capza3;ripply1;cdk2ap1;sergef;nfkbil1;sumo1;lcor;klf11;vps72;lancl2;nr0b2;rgs17;gas2;cdkn2aip;il3;scmh1;pak1ip1;pim1;dcbld2;tp53bp2;plekho1;ciapin1;glis2;capg;iqcb1;tnp1;cd274;rasa1;sirt3;gp1ba;edn1;cdh13;gja1;ern1;cdkn2d;uimc1;birc3;rgs1;rbm5;clic4;nlk;sesn1;sirt5;arhgdib;ctnna1;atp2a2;tgfb1;msx1;mapk1;dffa;arhgdia;pld2;ppp1r15a;sox9;cda;ghsr;eif4g2;pdcd4;ncoa2;supt4h1;pou2f1;ilf3;adra1b;rgs21;sirt1;znf24;clcf1;rbl2;twist1;gsk3b;cdc73;pnn;trim54;aurkaip1;hus1;ctnnb1;fxyd5;bnipl;cgref1;tnfaip3;alox12;kng1;capza2;lats2;klf12;serpina5;lep;rgs22;ctcf;sema4d;rgs11;dcc;ctbp2;wars;hspb1;prkca;zbtb32;nras;bcl6;ppm1d;taok3;npas1;adam10;kiss1r;dedd;tlx3;eef1e1;set;stat3;crebzf;tgfbi;tnip1;htra1;rhob;socs1;cdk10;faim;wnt2;terf2;pth2r;cd28;sirt4;vegfa;foxc1;cd74;sim2;eid2;cdkn3;tgfb2;azu1;fstl3;mcl1;bclaf1;nr6a1;nbl1;ihh;cxxc4;krt1;ngfr;cux1;zbtb16;ing1;igfbp6;arid5a;scap;gzf1;malt1;tspyl2;eif2ak2;prkaa1;rassf5;alb;rfx3;nab1;hells;psmc5;cdk6;skap2;cd164;bub1b;mecp2;vsig4;sycp3;cdt1;tax1bp1;dph1;trim13;runx3 |
| negative regulation of cellular process; | 168#1137 | 1.82486E-28 | rgs4;actn4;asz1;grm8;prkra;sox15;dkk2;capza3;ripply1;cdk2ap1;nfkbil1;sumo1;lcor;klf11;vps72;lancl2;nr0b2;rgs17;gas2;cdkn2aip;il3;scmh1;pak1ip1;pim1;dcbld2;tp53bp2;plekho1;ciapin1;glis2;capg;iqcb1;tnp1;cd274;rasa1;sirt3;edn1;cdh13;gja1;ern1;cdkn2d;uimc1;birc3;rgs1;rbm5;clic4;nlk;sesn1;sirt5;arhgdib;ctnna1;tgfb1;msx1;mapk1;dffa;arhgdia;pld2;ppp1r15a;sox9;cda;ghsr;eif4g2;pdcd4;ncoa2;supt4h1;pou2f1;ilf3;adra1b;rgs21;sirt1;znf24;clcf1;rbl2;twist1;gsk3b;cdc73;trim54;pnn;aurkaip1;hus1;fxyd5;ctnnb1;bnipl;cgref1;tnfaip3;alox12;kng1;capza2;lats2;klf12;serpina5;rgs22;ctcf;sema4d;rgs11;dcc;ctbp2;wars;hspb1;prkca;zbtb32;nras;bcl6;ppm1d;taok3;npas1;adam10;kiss1r;dedd;tlx3;eef1e1;set;stat3;crebzf;tgfbi;htra1;rhob;socs1;cdk10;faim;wnt2;terf2;pth2r;cd28;sirt4;vegfa;foxc1;cd74;sim2;eid2;cdkn3;tgfb2;azu1;fstl3;mcl1;bclaf1;nr6a1;nbl1;ihh;cxxc4;cux1;zbtb16;ing1;igfbp6;arid5a;scap;gzf1;malt1;tspyl2;eif2ak2;prkaa1;rassf5;alb;rfx3;nab1;hells;psmc5;cdk6;skap2;cd164;bub1b;mecp2;vsig4;sycp3;cdt1;tax1bp1;dph1;trim13;runx3 |
| multicellular organismal development; | 280#2299 | 1.84885E-28 | ikzf1;cops3;pcdh18;hoxd1;tshz3;dmc1;mark2;myl4;nr4a2;prkra;tfip11;dkk2;fhl1;fez1;nr5a1;ablim1;il3;traf7;neurod6;myl1;axin2;tbcb;sparc;rbm45;glis2;ets2;pcdh1;vsx1;cdx2;iqcb1;aplp1;cylc1;gna13;serpinf1;rasa1;plp1;gnpat;nrp1;dpysl3;edn1;cdh13;gfra3;myog;sfrp2;keap1;cd2;donson;thbd;dlx2;dip2a;dnm1l;msx1;pou3f2;adam12;vps33a;sox9;pou4f3;map4k2;lrp6;kcnmb4;mobp;kcnq2;klk7;snai1;alx1;sirt1;cck;twist1;nrg3;adora2b;hey2;trim54;spata9;aldh5a1;vax1;atrnl1;fgf1;hoxa3;sema4d;jmjd6;fgd1;nras;mmp9;ccin;fgf6;bcl6;gsc;cyfip1;chrm2;adam10;tlx3;b3gnt2;tinag;stat3;pcdhb6;wnt5b;adm;maff;wnt11;doc2a;snrk;wnt2;dlx5;pth2r;gaa;chrna4;vegfa;foxc1;mbp;tgfb2;hoxd8;azu1;il20;mpped2;kif3b;spock1;pdgfc;ihh;whsc2;nr5a2;ngfr;cux1;nde1;zbtb16;hoxc9;six6;nle1;papss1;cln5;ostf1;hells;prrx1;cdk6;sgcg;hoxc12;trpc5;hcls1;hcn4;rufy3;pts;drp2;hexa;ift88;pax8;sectm1;hunk;meox2;amtn;eya3;pogk;asz1;dscaml1;foxn1;ikzf3;sox15;sema3a;chst8;ripply1;klf1;dctn1;comp;sema3d;mdga2;dazap1;evi5;pcdhb5;scmh1;pim1;phc3;cited2;ap3d1;tnp1;tgm5;itgb1bp2;fgf5;rs1;krt13;ldb2;gja1;shroom2;scrg1;piwil3;slc5a3;sox6;trim3;atp2c1;plod1;slitrk3;crim1;arhgdib;npr3;clec3b;prkci;nanog;ctnna1;tgfb1;atp2a2;mapk1;spin1;mid1;dusp22;mut;prelp;acvr2b;ghsr;sgca;pcdhb11;adra1b;drgx;efnb3;ankrd2;vgf;clcf1;mixl1;minpp1;eya4;hus1;ctnnb1;matn1;prdx1;kng1;pafah1b3;mdga1;krt5;rax;lep;bpgm;ugcg;b3gnt5;hdac9;flt1;btk;spry1;ift81;nrcam;ipmk;dcc;utp3;hoxb3;npas1;gpx4;pmch;nts;tnp2;pak3;klf5;fzd9;slitrk4;rhob;sema4a;crem;krt9;npm2;cd74;kiaa1598;fzd5;sim2;egr2;sfn;elf3;mcl1;crx;kitlg;sgcb;eln;krt1;cacna1f;onecut2;gzf1;slc30a1;creg1;wif1;cd164;ndrg2;gch1;runx1;dlx3;bves;adad1;eda2r |
| multicellular organismal process; | 406#3822 | 1.86698E-26 | chml;ikzf1;cfb;cops3;pcdh18;mybpc2;hoxd1;tshz3;myo3a;qprt;dmc1;mark2;myl4;nr4a2;prkra;tyr;tfip11;dkk2;uts2;map3k7;c1qc;fhl1;fez1;gabrb2;nr5a1;ablim1;il3;traf7;neurod6;myl1;axin2;tbcb;sparc;cds1;cldn14;rbm45;glis2;ets2;pabpc4;pcdh1;kifc3;vsx1;cdx2;iqcb1;aplp1;cylc1;gna13;serpinf1;rasa1;plp1;gp1ba;gnpat;nrp1;dpysl3;strn4;edn1;cdh13;gfra3;myog;sfrp2;keap1;cd2;donson;crygd;thbd;tff3;npr2;wdr36;tpm1;dlx2;dip2a;des;dnm1l;rtp3;sord;msx1;pou3f2;adam12;tlr2;vps33a;kcne1;sox9;pou4f3;pag1;map4k2;lrp6;kcnmb4;mobp;sncb;kcnq2;klk7;snai1;alx1;sirt1;cck;f7;twist1;nrg3;adora2b;hey2;nck1;trim54;soat1;aqp1;rom1;traf6;galr1;spata9;aldh5a1;ucn2;vax1;sntb1;myd88;atrnl1;fgf1;pde6d;hoxa3;sema4d;jmjd6;fgd1;efemp1;serpinc1;prkca;nras;mmp9;ccin;fgf6;snph;bcl6;slc6a2;gsc;adam10;cyfip1;chrm2;tlx3;b3gnt2;tinag;stat3;pcdhb6;camk4;wnt5b;tgfbi;scn1b;adm;c4bpb;maff;wnt11;doc2a;snrk;pdc;wnt2;dlx5;pth2r;myom1;gaa;chrna4;vegfa;foxc1;ndufb9;mbp;pgam2;tgfb2;hoxd8;azu1;il20;mpped2;pnoc;crybb2;kif3b;spock1;clcn5;pdgfc;cd93;whsc2;ihh;nr5a2;ngfr;cux1;nde1;zbtb16;hoxc9;six6;malt1;nle1;papss1;cln5;ostf1;f3;hells;prrx1;cdk6;sgcg;hoxc12;trpc5;hcls1;hcn4;vsig4;rufy3;pts;drp2;hexa;ift88;pax8;sectm1;hunk;meox2;amtn;crybb3;eya3;dscaml1;pogk;asz1;foxn1;grm8;marveld2;drd3;brs3;ikzf3;bcat2;sox15;sema3a;chst8;ripply1;klf1;dctn1;comp;sema3d;mdga2;rpgr;dazap1;evi5;procr;pepd;nfatc2ip;pcdhb5;scmh1;pim1;phc3;chrna5;cited2;ap3d1;tgm5;tnp1;itgb1bp2;gp6;fgf5;tff1;cd274;rs1;slc22a18;aipl1;krt13;maoa;cfi;ldb2;shroom2;gja1;rabggta;scrg1;piwil3;slc5a3;sox6;trim3;atp2c1;stbd1;plod1;kcnv2;slitrk3;nlrp2;crim1;arhgdib;ltb4r;npr3;clec3b;prkci;rd3;nanog;ctnna1;atp2a2;tgfb1;mapk1;spin1;mid1;dusp22;mut;acvr2b;prelp;slmap;bbs9;ghsr;sgca;gprc5d;mrcl3;aldh7a1;pcdhb11;drgx;adra1b;mtnr1b;opn1sw;efnb3;vgf;ankrd2;clcf1;timp3;minpp1;mixl1;hus1;eya4;lin7a;ctnnb1;slc22a4;matn1;prdx1;znf354a;kng1;mdga1;pafah1b3;krt5;rax;rtp4;lep;bpgm;ugcg;b3gnt5;scnn1b;plaur;hdac9;flt1;btk;snta1;cckbr;ift81;spry1;dcc;ipmk;nrcam;utp3;hoxb3;tfpi2;apoh;kcnq1;npas1;nedd4l;gna12;gpx4;pmch;nts;tnp2;pak3;fzd9;klf5;best1;slitrk4;cabp4;rhob;sema4a;agtr1;crem;krt9;chrne;cd28;npm2;entpd1;kiaa1598;cd74;spaca3;fzd5;sim2;kcnj12;elf1;ppp1r12b;egr2;sfn;elf3;th;cnga3;mcl1;crx;kitlg;sgcb;eln;prtn3;dmpk;krt1;cacna1f;fxyd1;olr1;onecut2;gzf1;lman1;cacnb3;slc30a1;wif1;creg1;pxk;gnal;cd164;ndrg2;runx1;gch1;dlx3;bves;slc6a5;dbh;adad1;eda2r |
| cellular component organization and biogenesis; | 358#3277 | 1.29524E-25 | stx10;snx15;exosc10;ptk2;chml;prickle2;izumo1;nrbp1;mark2;ndc80;vps16;lrp10;fhl1;ctsa;cdk9;fez1;sf3a2;vps72;tomm22;sec61a2;ablim1;necap1;cdc42bpb;il3;ramp1;sqstm1;traf7;cdc42ep5;vps26b;hook3;cldn14;chd3;kifc3;capg;aplp1;gna13;rasa1;sirt3;eif3f;gp1ba;nrp1;kpna2;rrp9;chaf1a;cdh13;cd2;stx4;xpo6;eif1b;snrpe;mki67ip;cntrob;usp39;cno;wdr36;elmo1;hmgn2;akap8;des;dnm1l;rtp3;nupl2;pou3f2;adam12;rbm14;cdc37;vps33a;dffa;fscn1;bace2;pou4f3;map4k2;eif4g2;lrp6;kcnmb4;supt4h1;pex12;tfrc;sirt1;cck;rbl2;gsk3b;nrg3;gtf2e1;nck1;trim54;h2afz;shc1;lysmd3;alox12;ran;med23;aldh5a1;ykt6;ldlrap1;uso1;serpina5;akap4;hdac10;asgr2;srp72;sema4d;hsp90aa1;fgd1;nxf1;stx7;abcd3;slc25a10;net1;nhp2l1;nras;mmp9;pram1;cpa4;snph;senp2;bcl6;sec61a1;cyfip1;tubgcp3;ap1s3;b3gnt2;ftsj2;ap1g1;rabep2;pcdhb6;tmem48;camk4;nubp1;taf4;vps41;psap;socs1;brms1l;rae1;doc2a;wnt2;dlx5;gaa;foxc1;slc7a7;eid2;lyz;bub3;kpna6;tgfb2;azu1;ezh2;kif3b;clcn5;ing3;kpna3;cd93;tinf2;ngfr;taf6;nde1;igfbp6;pacsin1;malt1;kif18a;klhl2;abi2;cln5;picalm;optn;hells;sec24b;sgcg;skap2;bub1b;hcn4;fgf20;shank1;drp2;hexa;brf2;cd14;ankrd27;daam2;utp6;kif9;ict1;actn4;asz1;dscaml1;rapgef4;dnm2;camk2d;tmod1;supt16h;sox15;arfip1;scamp2;capza3;dnm3;gpaa1;nup35;klf1;sergef;nfkbil1;gle1;rpgr;taok2;gas2;cdkn2aip;tlk1;pcdhb5;pex16;dbnl;dcbld2;ap3d1;ywhab;tnp1;tnks;eif4h;arfgef2;ebag9;crnkl1;shroom2;gja1;atg7;il1rap;copg2;cdkn2d;ap3s1;traf1;sox6;pcna;atp2c1;setdb2;slitrk3;crim1;nlrp2;sirt5;pbrm1;arhgdib;ppp4c;gnptg;srp54;eif1ax;kif3a;prkci;pak1;ddx19b;ctnna1;nusap1;imp4;tgfb1;ebna1bp2;rad51;mid1;pld2;cda;dync1i1;exoc3;ghsr;mark1;cdyl2;vps26a;trpv5;pcdhb11;adra1b;mia;prpf6;adrm1;cct6b;efnb3;tubg1;eif5;rragc;egln2;cxcl16;lin7a;pdia2;ctnnb1;fxyd5;matn1;gga3;scrn1;capza2;synj2bp;arhgap1;rtp4;rcc1;tubgcp5;tspyl1;stx16;hdac9;krt20;exosc7;dcc;nrcam;hspb1;utp3;scarf1;nedd4l;ftsj1;gpx4;tnp2;set;tbpl2;klf5;scfd1;ranbp9;slitrk4;htra1;rhob;cep57;hspd1;cog2;terf2;hps6;cog5;krt9;kif1c;sirt4;npm2;cd74;spaca3;nap1l5;cdc42ep4;sfn;elf3;trappc1;slc25a17;sgcb;med24;cacna1f;eif3e;gtf2a1;tubgcp4;ing1;stxbp1;atg4a;ncaph;lman1;tspyl2;tubb6;alb;creg1;stat1;atg12;col8a2;wisp1;c1qtnf2;denr;napg;epc2;lmbr1l;eif3i;rab9b;esm1;sycp3;dot1l;gosr1;thbs4;ncapg |
| positive regulation of biological process; | 155#1062 | 1.77081E-25 | sectm1;cfb;actn4;sap30bp;asz1;slc20a1;dnm2;nr4a2;prkra;sox15;map3k7;cx3cl1;plekhf1;traf3ip2;pparg;c1qc;bcl2l11;taok2;esrrg;tnfrsf25;cdkn2aip;il3;sqstm1;traf7;cdc42ep5;pdcd5;tp53bp2;samd4a;ddr2;glis2;cited2;trim38;vapb;lalba;serpinf1;nrp1;cd70;cfi;edn1;traf3;cdh13;gja1;myog;eef1d;ern1;cd2;nfatc2;atp2c1;nlrp2;dlx2;ctnna1;nusap1;tgfb1;pou3f2;rbm14;mapk1;tlr2;rad51;dyrk1b;acvr2b;sox9;utf1;ghsr;ncoa2;supt4h1;ilf3;adra1b;prpf6;clock;clcf1;gsk3b;nck1;egln2;elk1;shc1;aurkaip1;traf6;ctnnb1;alox12;ran;kng1;ldlrap1;myd88;lep;ilf2;flt1;btk;ctcf;sema4d;cckbr;hsp90aa1;dcc;nrcam;adamtsl4;ccnd3;prkca;nras;mmp9;ppargc1b;bcl6;taok3;nedd4l;dedd;eef1e1;pias1;klf5;grip1;sp1;adm;c4bpb;wnt2;dlx5;pth2r;ltbr;cd28;vegfa;foxc1;spaca3;ripk2;elf1;lgals9;htr1a;tgfb2;azu1;sfn;il20;crx;bclaf1;prtn3;ihh;yap1;krt1;ngfr;bik;rnf14;slamf1;scap;malt1;cacnb3;prkaa1;dio3;c1qtnf2;psmc5;cdk6;klf15;zbtb38;hcls1;gch1;runx1;plagl2;eda2r;trim13;runx3;pax8;ift88 |
| cellular developmental process; | 227#1810 | 4.83168E-25 | kiaa1967;cd14;actn4;sap30bp;dscaml1;asz1;foxn1;mark2;dnm2;nr4a2;prkra;sox15;sema3a;capza3;plekhf1;klf1;pparg;sema3d;fhl1;mdga2;bcl2l11;dazap1;fez1;vdac1;taok2;tnfrsf25;gas2;casp7;il3;pdcd2;sqstm1;traf7;neurod6;pdcd5;pim1;tbcb;ndufs1;tp53bp2;arhgef6;rbm45;ciapin1;glis2;ap3d1;cdx2;lalba;iqcb1;tnp1;aplp1;cylc1;gna13;serpinf1;rasa1;ebag9;nrp1;cd70;edn1;traf3;cdh13;gja1;myog;sfrp2;ern1;cdkn2d;keap1;cd2;traf1;birc3;clic4;slitrk3;thoc5;nlrp2;elmo1;dlx2;prkci;pak1;dnm1l;ctnna1;nanog;dhrs9;tgfb1;pou3f2;adam12;mapk1;tlr2;vps33a;dffa;arhgdia;dusp22;ppp1r15a;kcne1;fgf22;sox9;pou4f3;asah2;map4k2;eif4g2;tfap2c;pdcd4;efnb3;sirt1;cck;clcf1;twist1;gsk3b;trim54;rragc;mixl1;traf6;pdia2;ctnnb1;spata9;bnipl;tnfaip3;alox12;bmf;kng1;mdga1;srd5a1;trib3;uhrf2;lep;fgf1;bpgm;hdac9;flt1;btk;krt20;sema4d;jmjd6;ift81;spry1;dcc;nrcam;adamtsl4;bcl7c;hspb1;prkca;nras;mmp9;ccin;fgf6;bcl6;apip;cyfip1;il17a;tlx3;dedd;b3gnt2;pmch;tnp2;eef1e1;sgpl1;fzd9;clul1;slitrk4;rhob;maff;cep57;socs1;hspd1;snrk;faim;wnt2;crem;sema4a;tns4;hps6;dlx5;pth2r;mmd;ltbr;cd28;vegfa;npm2;foxc1;cd74;fzd5;ripk2;sim2;lyz;tgfb2;azu1;sfn;elf3;il20;znf346;mcl1;crx;madd;bclaf1;pacs2;ihh;ngfr;cacna1f;bik;rhcg;cux1;nde1;zbtb16;ppp2r2b;malt1;nle1;prkaa1;eif2ak2;rassf5;alb;stat1;atg12;ccar1;cln5;optn;psmc5;srpk1;cdk6;hcls1;ndrg2;runx1;bub1b;gch1;sycp3;rufy3;plagl2;tax1bp1;adad1;eda2r;pax8;runx3 |
| cell differentiation; | 227#1810 | 4.83168E-25 | kiaa1967;cd14;actn4;sap30bp;dscaml1;asz1;foxn1;mark2;dnm2;nr4a2;prkra;sox15;sema3a;capza3;plekhf1;klf1;pparg;sema3d;fhl1;mdga2;bcl2l11;dazap1;fez1;vdac1;taok2;tnfrsf25;gas2;casp7;il3;pdcd2;sqstm1;traf7;neurod6;pdcd5;pim1;tbcb;ndufs1;tp53bp2;arhgef6;rbm45;ciapin1;glis2;ap3d1;cdx2;lalba;iqcb1;tnp1;aplp1;cylc1;gna13;serpinf1;rasa1;ebag9;nrp1;cd70;edn1;traf3;cdh13;gja1;myog;sfrp2;ern1;cdkn2d;keap1;cd2;traf1;birc3;clic4;slitrk3;thoc5;nlrp2;elmo1;dlx2;prkci;pak1;dnm1l;ctnna1;nanog;dhrs9;tgfb1;pou3f2;adam12;mapk1;tlr2;vps33a;dffa;arhgdia;dusp22;ppp1r15a;kcne1;fgf22;sox9;pou4f3;asah2;map4k2;eif4g2;tfap2c;pdcd4;efnb3;sirt1;cck;clcf1;twist1;gsk3b;trim54;rragc;mixl1;traf6;pdia2;ctnnb1;spata9;bnipl;tnfaip3;alox12;bmf;kng1;mdga1;srd5a1;trib3;uhrf2;lep;fgf1;bpgm;hdac9;flt1;btk;krt20;sema4d;jmjd6;ift81;spry1;dcc;nrcam;adamtsl4;bcl7c;hspb1;prkca;nras;mmp9;ccin;fgf6;bcl6;apip;cyfip1;il17a;tlx3;dedd;b3gnt2;pmch;tnp2;eef1e1;sgpl1;fzd9;clul1;slitrk4;rhob;maff;cep57;socs1;hspd1;snrk;faim;wnt2;crem;sema4a;tns4;hps6;dlx5;pth2r;mmd;ltbr;cd28;vegfa;npm2;foxc1;cd74;fzd5;ripk2;sim2;lyz;tgfb2;azu1;sfn;elf3;il20;znf346;mcl1;crx;madd;bclaf1;pacs2;ihh;ngfr;cacna1f;bik;rhcg;cux1;nde1;zbtb16;ppp2r2b;malt1;nle1;prkaa1;eif2ak2;rassf5;alb;stat1;atg12;ccar1;cln5;optn;psmc5;srpk1;cdk6;hcls1;ndrg2;runx1;bub1b;gch1;sycp3;rufy3;plagl2;tax1bp1;adad1;eda2r;pax8;runx3 |
| regulation of biological process; | 584#6140 | 4.83168E-25 | rgs4;ikzf1;cfb;tigd3;hoxd1;tshz3;znf566;traf3ip2;klf11;cdk9;hif1an;il3;plekho1;glis2;pstk;rnf139;ets2;vsx1;cdx2;lalba;aplp1;serpinf1;znf41;rasa1;sirt3;eif3f;zbtb33;nrp1;cd70;kpna2;smad6;edn1;traf3;cdh13;myog;runx1t1;prdm14;clic4;iqgap2;zxdc;tpm1;msx1;rdbp;zbtb25;vps33a;dyrk1b;dffa;kcne1;smap1;bace2;eif4g2;nr4a1;supt4h1;alx1;rgs21;sirt1;rbl2;twist1;adora2b;bhmt;traf6;elf5;irf5;ran;lats2;trib3;ldlrap1;myd88;cables1;hdac10;hsp90aa1;fgd1;tcf23;fubp3;zbtb45;ccnd3;net1;zbtb32;nras;mmp9;ppm1d;bcl6;senp2;gsc;kiss1r;usf1;atf7;stat3;sp1;tgfbi;c4bpb;maff;socs1;brms1l;znf501;cbfa2t2;ddb1;pth2r;junb;mlxipl;foxc1;ripk2;htr1a;tgfb2;hoxd8;azu1;ezh2;meis3;bclaf1;nbl1;yap1;ihh;ngfr;igf2bp3;cux1;zbtb16;hoxc9;znf768;igfbp6;polh;irx3;sfpq;mtf1;znf581;dio3;psd4;prrx1;srpk1;cdk6;skap2;hcls1;bub1b;hcn4;creb3;rnf2;dph1;ift88;sectm1;ssrp1;eya3;pogk;slc20a1;rapgef4;ikzf3;dnm2;camk2d;cdca7;capza3;cx3cl1;ripply1;zscan21;sergef;hmgb4;mkl1;tox3;taok2;esrrg;znf446;rcor2;rfx5;cdkn2aip;tlk1;pim1;tox;rapgef1;tp53bp2;samd4a;ddr2;cited2;trim38;ap3d1;zbtb20;ebag9;dek;duxa;maoa;atg7;zfx;tbx10;traf1;med8;sp2;rgs1;atp2c1;nlk;pbrm1;znfx1;znf496;ctnna1;tgfb1;mapk1;elk4;ghsr;phtf1;mrcl3;ncoa2;drgx;adra1b;tceal8;eif5;egln2;mixl1;tox4;hus1;eya4;ctnnb1;fxyd5;elavl2;bnipl;polr2f;znf354a;capza2;rax;znf451;lep;l3mbtl3;scnn1b;plaur;flt1;hdac9;krt20;dcc;nrcam;adamtsl4;hoxb3;ppargc1b;kcnq1;taok3;nedd4l;gpx4;eef1e1;pias1;set;klf5;rab8a;tnip1;htra1;rhob;cdk10;hspd1;tsc22d4;ltbr;mysm1;tead1;spaca3;kcnj12;sim2;ppp1r12b;elf1;lgals9;egr2;gabpb2;th;mcl1;hectd3;pcgf1;aoah;krt1;eif3e;ing1;arid5a;slamf1;onecut2;scap;cacnb3;alb;stat1;creg1;wisp1;psmc5;c1qtnf2;dlx3;runx1;esm1;sycp3;tax1bp1;eda2r;foxp4;sap30bp;znf558;znf33b;prkra;nr4a2;znf548;dkk2;map3k7;plekhf1;cdk2ap1;c1qc;iqsec3;pparg;cdc16;bcl2l11;sumo1;znf414;lcor;vps72;gab1;nr5a1;tnfrsf25;rgs17;cdc42bpb;ramp1;sqstm1;traf7;neurod6;cdc42ep5;gmeb2;pak1ip1;chd3;ciapin1;capg;fubp1;iqcb1;gna13;rasa4;gp1ba;chaf1a;ikzf4;ern1;znf664;keap1;cd2;med20;nfatc2;zfp2;eif1b;cnbp;sesn1;polr3c;dlx2;hmgn2;foxj3;des;zfp42;ccnt2;atf3;pou3f2;cdc37;tlr2;rbm14;arhgdia;znf214;bcl11b;sox9;pou4f3;utf1;pag1;rab10;rab37;kcnmb4;vgll4;pou2f1;sncb;clock;znf605;gsk3b;nrg3;gtf2e1;diras3;hey2;cdc73;trim54;nck1;ddx54;elk1;shc1;znf546;cnot6;rasgrp2;alox12;med23;aldh5a1;klf12;znf197;vax1;uhrf2;serpina5;iqsec2;hoxa3;sema4d;ctbp2;rgs11;wars;prkca;pram1;snph;arfgap1;cyfip1;chrm2;adam10;tlx3;znf175;grip1;zfp1;taf4;adm;sohlh2;znf710;wnt2;dlx5;ube2m;vegfa;eid2;tfe3;cdkn3;bub3;tbc1d14;med18;slc30a9;il20;ppie;ing3;madd;whsc2;nr5a2;taf6;rnf14;six6;arhgef9;malt1;eif2ak2;prkaa1;zscan29;rassf5;hdx;znf771;sp5;hells;zbtb12;hoxc12;klf15;pum2;scml1;vsig4;rarb;plagl2;trim13;ovol2;pax8;runx3;brf2;lcorl;habp4;meox2;actn4;asz1;foxn1;znf524;grm8;supt16h;sox15;arfip1;klf1;znf787;nfkbil1;evi5;nr0b2;lancl2;gas2;nfatc2ip;znf786;pdcd5;scmh1;dcbld2;znf623;znf688;arhgef6;vapb;tnp1;cd274;eif4h;arfgef2;pigu;cfi;gja1;prdm12;eef1d;cdkn2d;trim24;gabpa;uimc1;slc5a3;sox6;pcna;birc3;rbm5;crim1;nlrp2;sirt5;arhgdib;mad2l2;pknox1;nusap1;nanog;atp2a2;rad51;pld2;ppp1r15a;cda;acvr2b;tfap2c;pdcd4;ilf3;prpf6;hif3a;znf24;snip1;clcf1;cnot4;pnn;aurkaip1;lin7a;tnfaip3;cgref1;kng1;synj2bp;abt1;grhl2;rcc1;rgs22;scml2;ilf2;btk;ctcf;tcea3;cckbr;spry1;hspb1;apoh;pcgf2;npas1;dedd;tbpl2;krba2;crebzf;faim;rasal2;znf689;crem;casz1;terf2;cd28;sirt4;npm2;cd74;znf131;cdc42ep4;ccng1;sox5;prdm4;fstl3;sfn;elf3;znf398;crx;vav3;dbx2;atf2;znf454;nr6a1;prtn3;snd1;cxxc4;med24;dmpk;bik;gtf2a1;mapk8ip1;gzf1;tspyl2;igsf1;nab1;rfx3;pxk;brsk1;epc2;eif3i;cd164;zbtb38;gch1;mecp2;cdt1;rad1 |
| biological_process; | 1803#24743 | 1.8144E-23 | ttbk1;stx10;fkbp3;chml;prickle2;rsu1;ikzf1;amica1;nox1;fbxo10;tigd3;usp30;mybpc2;hoxd1;myo3a;psmc2;znf566;myl4;fut1;scarb2;chmp7;traf3ip2;lrp10;fhl1;tusc5;pde11a;klf11;e4f1;sec61a2;tec;necap1;plek;angptl5;il3;naprt1;pdcd2;plod2;adam20;vps26b;adra2b;cacng7;arih1;st3gal3;phkg2;rbm45;rbbp6;glis2;pstk;vsx1;cdx2;lalba;apobec2;aplp1;hgsnat;serpinf1;rasa1;gnpat;kpna2;nat8l;edn1;traf3;akt2;cdh13;myog;xpo5;mex3b;iqgap2;rspo1;cstf1;pld5;calr3;nrxn1;tff3;cdh16;cntrob;thoc5;rpl8;cno;wdr36;opcml;mrpl44;rbm23;abl2;atp11a;msx1;zbtb25;kctd18;vps33a;dyrk1b;usp15;fbxl8;kcne1;snx27;impa2;map4k2;eif4g2;kremen1;pkd2l1;klk7;snai1;pex12;alx1;tfrc;sirt1;fsd1l;gin1;hectd2;gabbr1;mrpl3;f7;c1qtnf3;leng1;h2afz;dcbld1;ccr5;rom1;fibcd1;elf5;spata9;lysmd3;ran;ykt6;tbcc;srd5a1;trib3;atrnl1;cdc14b;khk;slc29a3;nfkbib;asgr2;hsp90aa1;jmjd6;nxf1;fubp3;efemp1;pdxdc1;serpinc1;net1;zbtb32;l2hgdh;nras;tmub2;ints5;ppm1d;bcl6;senp2;gsc;apip;slc4a8;kiss1r;gnb2;usf1;dhrsx;rabep2;pcdhb6;cog7;camk4;tgfbi;wnt5b;gpr55;c4bpb;socs1;cbfa2t2;oxgr1;ddb1;gnat3;mmd;myom1;mlxipl;fbxo45;slc22a16;stac;kpna6;xrcc2;azu1;osbpl2;ezh2;mpped2;znf346;pnoc;tprkb;ctsw;ppic;tsfm;nbl1;yap1;ihh;ngfr;igf2bp3;tspan2;rhcg;mcam;nde1;slco1b3;proca1;igfbp6;islr;ppp2r2b;spns3;irx3;sdc2;sfpq;slbp;aldh16a1;znf330;znf581;cln5;optn;cdk6;skap2;fance;insig1;hcls1;creb3;gns;pds5a;inpp1;ndufv1;pts;hyal2;zbp1;senp8;dph1;pygl;ift88;sectm1;hunk;srgap3;daam2;utp6;mrpl30;prepl;kif9;ckm;eya3;susd5;dscaml1;kcnk2;cpa5;prkag2;asb2;pitpnc1;marveld2;rapgef4;ikzf3;camk2d;dnm2;tmod1;hamp;apeh;ca3;scamp2;depdc7;dnm3;zscan21;sergef;comp;mmab;gle1;adprh;hadha;tktl1;csnk2b;pvrl2;syncrip;esrrg;galns;hsph1;cdkn2aip;kcnip4;pim1;tp53bp2;samd4a;coro1a;omg;cited2;ap3d1;hibadh;galnt6;ulk3;adat1;zbtb20;qpctl;tff1;slc22a18;ebag9;duxa;mpp1;shroom2;atg7;zfx;med8;traf1;sp2;stbd1;atp2c1;nlk;galnt9;rab3c;ppp4c;syvn1;znf496;pygo1;kif3a;rd3;prkci;pak1;tp53rk;tgfb1;ebna1bp2;mapk1;als2cr12;dusp22;ace2;slmap;elk4;dync1i1;ghsr;rps29;gucy2c;cnih4;sgca;gprc5d;pkp3;cad;pcdhb11;drgx;tubg1;efnb3;slc23a2;ppp1cc;tceal8;aldh1a1;timp3;rnf41;egln2;mixl1;tox4;hus1;dhx15;slc22a4;slc16a14;tysnd1;matn1;slc16a3;polr2f;znf354a;capza2;znf451;grsf1;mrpl16;fa2h;snx25;arhgap1;sc5dl;hyou1;rtp4;lep;bpgm;scnn1b;tspyl1;plaur;tgds;usp2;best4;nrcam;pnpla3;kcnk1;pglyrp3;adamtsl4;bcl7c;scarf1;gpr19;trhr;ppargc1b;kcnq1;taok3;slc30a10;nts;oma1;mrpl37;neu1;pias1;set;ccr2;clul1;klf5;slc9a8;rab8a;slitrk4;tnip1;htra1;rhob;hspd1;lrrc29;snx14;agtr1;ltbr;mysm1;entpd1;tead1;nap1l5;kcnj12;sim2;lgals9;egr2;gfpt1;mrpl47;rad54l;mcl1;atp6v1g3;pgd;ampd1;pacs2;eln;p4ha1;rab3ip;fxyd1;eif3e;tpm4;sarm1;ing1;slamf1;onecut2;scap;ttc1;ncaph;ptges;alb;fbxo47;atg12;stat1;mln;epb41l4a;nat1;pycr1;gmppb;napg;mns1;gdpd3;ndrg2;runx1;ldhc;slc6a5;sycp3;gosr1;mrpl35;eda2r;ncapg;foxp4;btbd10;sap30bp;ears2;spcs2;znf558;qprt;dusp10;znf33b;nr4a2;tyr;tfip11;negr1;uts2;map3k7;popdc3;fkbp6;ndc80;cdk2ap1;plekhf1;pparg;cdc16;cadm3;znf414;sumo1;ctsa;atp5o;vps72;fbxl17;gabrb2;hapln3;afmid;ablim1;rgs17;tmem50b;cdc42bpb;b3gnt4;cacng6;ramp1;sqstm1;traf7;neurod6;pak1ip1;sparc;hook3;ddah1;cldn14;prmt2;pcdh1;iqcb1;cylc1;gna13;rasa4;cxcl14;csnk1g3;plp1;abcf1;cpa3;acsm5;strn4;kcns3;chaf1a;ikzf4;nhlrc2;acadl;ern1;cxadr;rasl11a;cd2;donson;c1qtnf1;med20;thbd;paics;kynu;akap8l;pltp;cpxm1;foxj3;mars2;ampd3;des;zfp42;ccnt2;pwp1;nupl2;atf3;hsd17b13;tshb;pou3f2;rbm14;tlr2;cdc37;arhgdia;cnih2;znf214;sox9;wars2;utf1;pou4f3;pag1;ppm1k;lrp6;ganab;ugdh;kcnmb4;vgll4;pcsk7;sncb;pou2f1;polr2d;mrps18b;znf605;pja2;prkaa2;gsk3b;ccdc11;diras3;gtf2e1;coro2a;hey2;nck1;trim54;neu3;sdha;soat1;shc1;aqp1;rasgrp2;psmd14;mrpl10;aldh5a1;lrrk1;syt9;uso1;tmem115;slc13a1;dnajc17;iqsec2;tagap;akap4;gpr142;pi15;pde6d;hoxa3;sema4d;pygo2;dera;mvd;rnf186;slc31a1;rgs11;tsga10;b3galt5;wars;slc25a10;defb123;prkca;fcgr3a;nhp2l1;plcz1;pram1;mthfd1l;arfgap1;npffr2;mettl1;adam10;gnai3;tubgcp3;sh2b1;znf175;sugt1;grip1;tmem48;taf4;ajap1;adm;cops2;sohlh2;wnt11;usp11;sntg1;sh2d1b;dlx5;slco6a1;araf;vegfa;ccbe1;slc7a7;papd5;eid2;fbxo9;cdkn3;dsc2;syt12;tbc1d14;hspa1l;cldn2;slc30a9;med18;vps53;lrpap1;crybb2;kif3b;errfi1;nxph4;sf3b2;ppie;ing3;pdgfc;cd93;whsc2;nr5a2;taf6;slc6a19;agxt2l1;lrrc14;qdpr;six6;cmas;fbxw8;malt1;nle1;ecsit;klhl2;rassf5;papss1;kctd12;picalm;hars;f3;sp5;hells;ctnna3;zbtb12;sec24b;sgcg;hoxc12;mrpl40;klf15;osbpl7;kcnf1;chst1;aurkb;shank1;plagl2;drp2;hexa;ccr9;nudt9;runx3;pax8;zc3h12d;lcorl;fbln5;upf3a;amtn;actn4;foxn1;neil1;map3k9;kctd9;sdc4;drd3;arfip1;supt16h;chst8;cachd1;klf1;hpse;nfkbil1;cd1b;sms;dcp1a;pdss2;pepd;lancl2;nr0b2;nfatc2ip;casp7;htr4;sh3pxd2a;dbnl;znf786;ldha;pdcd5;sars;b3galt2;ptpn21;senp1;herc6;eif4h;arfgef2;aipl1;crnkl1;pigu;cfi;prdm12;eef1d;il1rap;copg2;cdkn2d;piwil3;rbck1;slc5a3;chst12;sox6;birc3;pcna;aloxe3;setdb2;atp6v0b;sirt5;hnrnpl;arhgdib;pknox1;skiv2l2;ddx19b;nusap1;imp4;atp2a2;axl;rad51;ppp1r15a;kcnk6;gpr3;gpr139;bbs9;klhl15;aco1;mark1;atp5b;kcnj5;cdc27;tfap2c;fbxo38;trpv5;aldh7a1;slc38a4;mtnr1b;hif3a;ankrd2;snip1;clcf1;pgk2;pnn;rragc;aurkaip1;anapc7;lin7a;idh3a;prdx1;gga3;cgref1;twf1;krt5;elovl7;asphd1;lrrc41;grhl2;rcc1;dgka;scml2;ilf2;btk;rem1;ppp1r1b;ift81;psma8;mfap4;rps3;spint2;rpp30;pole3;ndor1;slc6a17;mid2;nt5c1a;npas1;farsa;trappc6a;abcg8;enosf1;ftsj1;pmch;tnp2;grb10;sgpl1;psmb5;ifi35;best1;rnpc3;cabp4;krba2;alg3;slc39a6;pip4k2a;nrm;usp33;rab42;st3gal4;rasal2;nit1;znf689;sema4a;mchr1;tns4;chrne;gpr88;dnali1;kiaa1598;cd74;znf131;cdc42ep4;pigb;rassf8;fstl3;elf3;wee2;parvb;crx;trub2;znf398;vav3;kitlg;gan;tnpo3;snd1;dmpk;bik;rpl9;fbp2;gtf2a1;acly;stxbp1;mapk8ip1;jak1;tubb6;ca2;rsl1d1;nab1;col8a2;hk3;gnal;epc2;nuak1;cd164;zbtb38;pdha1;gch1;phospho2;nxph2;nars;cdt1;dot1l;gmps;kcna5;scnm1;dbh;kiaa1967;pigq;snx15;exosc10;rgs4;cfb;pla2g12a;mcee;pcdh18;tshz3;nrbp1;nsf;il1r2;slc27a2;msrb3;gphn;arg1;sucla2;st13;arhgap9;grap2;ctdsp1;cdk9;tspan8;sf3a2;chst14;zadh2;hif1an;axin2;ccl28;tbcb;st6galnac4;rrm2b;plekho1;clec1a;pop5;slc30a4;ets2;rnf139;gpr82;eral1;znf41;pcdhb7;sirt3;eif3f;zbtb33;phka2;dpysl3;nrp1;pcdhga2;cd70;smad6;gfra3;sfrp2;stx4;runx1t1;xpo6;uchl5;cthrc1;icos;prdm14;snrpe;clic4;mki67ip;usp39;zxdc;elmo1;tpm1;usp32;aprt;akap8;rtp3;arpc1b;dcp2;adam12;rdbp;dffa;smap1;bace2;zcrb1;asah2;nr4a1;mrps35;tmem37;mobp;supt4h1;kcnq2;rgs21;tm9sf2;ldhb;arhgap21;rbl2;usp13;lrrc23;twist1;gnb1;cstb;adora2b;bhmt;gfra2;traf6;ogt;kcnc1;galr1;ms4a7;epha3;irf5;ncam2;pcbp3;sdf2;ucn2;tst;diras1;lats2;decr1;ldlrap1;sntb1;myd88;ythdf2;cables1;fbxl22;ca9;hdac10;cldn17;st6gal1;kctd8;fgd1;tcf23;phkg1;zbtb45;ccnd3;fuca2;kcna2;leprel2;flrt2;mmp9;dlst;ccin;slc6a2;sec61a1;angpt4;atf7;adam21;stard5;grpel2;sp1;lmnb2;stat3;gnpda1;pfkfb2;cpb1;maff;atp6v1d;brms1l;znf501;rae1;b3galt1;snrk;pdc;slc30a6;clk3;pth2r;junb;chrna4;gpr68;foxc1;ripk2;lyz;pgam2;htr1a;hoxd8;tgfb2;cbs;plekha8;spock1;clcn5;meis3;slc39a14;bclaf1;kpna3;mrps9;slc2a12;me2;cux1;zbtb16;hoxc9;znf768;pacsin1;polh;mtf1;mag;ccar1;dio3;mmp12;slc9a7;psd4;serinc1;prrx1;srpk1;trpc5;hcn4;bub1b;trmu;rufy3;rnf2;sphar;anxa11;slc25a43;arv1;arhgap15;klk13;cd14;ankrd27;ssrp1;mrps18c;atp13a5;rsad2;pogk;igj;slc20a1;slc2a2;brs3;svop;cdca7;scn1a;sema3a;capza3;kcnab3;cx3cl1;ripply1;tm6sf1;gpaa1;g6pd;nup35;hmgb4;sh3rf2;dctn1;mkl1;rpgr;kctd19;tox3;inppl1;vdac1;cyb561;procr;trpm8;taok2;znf446;rcor2;rfx5;gbp5;tlk1;pcdhb5;trim2;acsl3;rapgef1;tox;phc3;ndufs1;ddr2;acbd5;zdhhc7;trim38;slc16a4;gpr84;fen1;kcns1;fgf5;ccnb1ip1;zcchc2;ttll5;dek;krt13;man1a1;maoa;gper;rabggta;scrg1;ppp4r2;kcnj11;ap3s1;tbx10;mrpl22;rgs1;mtap;plod1;dnah12;kcnv2;slitrk3;chdh;nalcn;enpp2;pbrm1;tor2a;gpr128;znfx1;stau2;gnptg;ppp1cb;pam;eif1ax;trim8;clec3b;ogfod1;map3k2;ctnna1;ndrg1;fdps;rqcd1;trpv1;fgf22;trip13;exoc3;ppm1l;gpr174;cdyl2;hacl1;cdkl2;slc37a3;sfxn3;phtf1;klk9;cst11;pdgfd;mrcl3;slc27a4;ncoa2;adra1b;mia;ppp2r5b;cct6b;mfge8;slc39a7;ergic3;mgat4a;ogdh;kmo;bckdhb;eif5;amd1;aldh8a1;uap1;cyp2e1;arl6;stac3;duoxa2;eya4;cxcl16;ctnnb1;fxyd5;elavl2;bnipl;scrn1;uqcrh;mdga1;rax;efna2;rpp14;l3mbtl3;rdh11;tubgcp5;stx16;hdac9;flt1;snta1;krt20;cpa6;rundc3a;dcc;mapkapk5;rnps1;utp3;uroc1;hoxb3;tfpi2;adat3;cacna2d3;rps10;srpx2;il17a;nedd4l;csnk1a1;gna12;gpx4;eef1e1;fzd9;scfd1;ranbp9;agmat;cep57;cdk10;cercam;tsc22d4;ralgps1;hps6;krt9;spaca3;plekha1;pigs;elf1;ppp1r12b;ugt1a9;rbp7;rad23a;gabpb2;th;cnga3;trappc1;dhx38;hectd3;pcgf1;slc25a17;a4gnt;aoah;sgcb;cd34;slc16a13;krt1;cacna1f;cdh12;slc35f2;olr1;srms;arid5a;atg4a;adk;lilra6;aadacl3;upp1;cacnb3;slc30a1;creg1;aqp11;wisp1;c1qtnf2;psmc5;aldh1l1;arhgap28;dlx3;esm1;alkbh1;impa1;capn2;acadsb;tax1bp1;thbs4;fasn;ptk2;pde1a;ppil3;acaca;has3;cops3;izumo1;rbm3;dmc1;mark2;znf548;prkra;dkk2;map3k4;cxcr7;vps16;fdxr;iqsec3;c1qc;ctss;ccbl2;vps52;bcl2l11;lcor;glyctk;acox2;fez1;slc4a2;gab1;tomm22;nr5a1;tnfrsf25;rce1;acoxl;pyroxd1;cdc42ep5;myl1;fancf;gmeb2;cds1;slc17a7;ptpn18;ciapin1;chd3;pabpc4;trpc7;kifc3;capg;fubp1;tmem68;klk12;n6amt2;gp1ba;gpr124;enpp4;rrp9;znf664;keap1;crygd;nfatc2;zfp2;cldn1;fech;cnbp;eif1b;mtmr7;npr2;cd53;sesn1;arl16;hnrpll;dlx2;polr3c;hmgn2;dip2a;ptpla;sv2b;fbxl4;dnm1l;sord;flrt3;bcl11b;fscn1;mpped1;gdpd1;rab10;rab37;aurkc;schip1;colec11;tgm7;rab33a;dpp8;lonp1;pdss1;gfpt2;cck;clock;nrg3;cdc73;xpr1;ddx54;elk1;znf546;cnot6;alcam;srm;sucnr1;ero1lb;pdxp;alox12;ddhd1;med23;slc18a2;vax1;znf197;klf12;serpina5;uhrf2;fgf1;hyal3;ubqln4;srp72;ctbp2;abcd3;stx7;dgkb;cpa4;slc7a6;fgf6;ssu72;snph;senp3;chrm2;cyfip1;ap1s3;cept1;tlx3;b3gnt2;ftsj2;tinag;ap1g1;zfp1;nubp1;vps41;scn1b;chka;psap;doc2a;znf710;wnt2;ccrl1;ube2m;oxsr1;gaa;grid2;mrpl36;kcnj13;pank4;ndufb9;dus1l;mbp;nub1;tfe3;bub3;rpp38;fbxl13;il20;scd;st3gal1;ofd1;itk;lonrf1;rps8;chmp4a;madd;qsox2;hapln2;hspa4;tinf2;prdx5;slc8a2;rnf14;arhgef9;zscan29;prkaa1;kif18a;eif2ak2;abi2;map4k3;ostf1;snx22;cysltr2;hdx;znf771;slc10a5;cd1a;pum2;cldn20;stag1;scml1;arl4c;rbm17;ero1l;vsig4;fgf20;agk;rarb;rrm2;pja1;ovol2;trim13;ugt1a6;brf2;tpst1;acot9;habp4;meox2;crybb3;ict1;asz1;znf524;grm8;bcat2;sox15;icam5;znf787;sema3d;scara5;mdga2;dazap1;abcg2;evi5;dgkz;pde1c;gas2;pex16;liph;appl1;vrk1;cgn;pdk2;scmh1;dcbld2;nqo2;clca1;znf623;znf688;chrna5;arhgef6;mttp;vapb;ywhab;sh2d3c;ocrl;prps2;hdgf;psph;tgm5;tnp1;itgb1bp2;rnf26;tnks;gp6;cd274;rexo2;rs1;hmgn3;ldb2;gja1;syt10;trim24;gabpa;mrpl27;apobec1;elspbp1;uimc1;trim3;neu2;tacr3;rbm5;nlrp2;crim1;fut3;ltb4r;mad2l2;srp54;npr3;nanog;dhrs9;slc9a5;nxph3;adam32;gcnt4;stac2;spin1;mid1;pld2;mut;map2k7;lcmt1;acvr2b;cda;prelp;shbg;cacng3;dusp13;fip1l1;vps26a;slc25a38;pdcd4;ilf3;adrm1;prpf6;opn1sw;clp1;vgf;znf24;stk39;ccr8;cnot4;ubtd2;nek8;minpp1;slc17a4;spsb3;ephx1;pdia2;srd5a3;ccr7;tnfaip3;kcng1;bmf;kng1;pafah1b3;heph;abt1;synj2bp;cnnm2;rgs22;b3gnt5;ugcg;camk2g;ctcf;cckbr;tcea3;exosc7;spry1;isca1;ipmk;chordc1;nxn;hspb1;gfer;ncln;apoh;cxcl11;zmat5;asna1;pcgf2;cryl1;cndp2;csrp2bp;rplp1;dedd;nmbr;isg20;sh3bp1;tbpl2;areg;pak3;gmppa;cdc40;il13;hadh;crebzf;slc22a14;trub1;smc5;cog2;faim;crem;nlgn4x;terf2;casz1;cog5;ca13;ttll9;dck;sirt4;cd28;kif1c;npm2;atp11b;fzd5;ccng1;sox5;prdm4;sfn;mkln1;hnrnpr;snx5;dbx2;atf2;znf454;nr6a1;tspan18;epha1;prtn3;igsf6;cxxc4;med24;tubgcp4;cd248;pskh1;cnksr1;gzf1;ubqln2;lman1;tspyl2;nrap;csn2;igsf1;wif1;pxk;nsun2;rfx3;brsk1;cstf2t;denr;lmbr1l;eif3i;trove2;rab9b;arl9;bves;atp2b3;mecp2;sac3d1;sgol1;pnmt;st8sia5;dnaja4;rnaseh1;adad1;rad1;fbxl6 |
| positive regulation of cellular process; | 140#954 | 2.43914E-23 | sectm1;actn4;sap30bp;asz1;slc20a1;dnm2;nr4a2;prkra;sox15;cx3cl1;plekhf1;traf3ip2;pparg;bcl2l11;taok2;esrrg;tnfrsf25;cdkn2aip;il3;sqstm1;traf7;pdcd5;tp53bp2;samd4a;ddr2;glis2;cited2;trim38;lalba;nrp1;cd70;edn1;traf3;cdh13;gja1;myog;eef1d;ern1;cd2;nfatc2;atp2c1;dlx2;ctnna1;nusap1;tgfb1;pou3f2;rbm14;mapk1;tlr2;rad51;dyrk1b;acvr2b;sox9;utf1;ghsr;ncoa2;supt4h1;ilf3;adra1b;prpf6;clock;clcf1;gsk3b;nck1;elk1;shc1;aurkaip1;traf6;ctnnb1;alox12;ran;kng1;ldlrap1;myd88;lep;ilf2;flt1;btk;ctcf;cckbr;nrcam;dcc;adamtsl4;ccnd3;prkca;nras;mmp9;ppargc1b;bcl6;taok3;nedd4l;dedd;eef1e1;pias1;klf5;grip1;sp1;adm;wnt2;dlx5;pth2r;ltbr;cd28;vegfa;spaca3;foxc1;ripk2;elf1;lgals9;htr1a;tgfb2;azu1;sfn;il20;crx;bclaf1;prtn3;ihh;yap1;ngfr;bik;rnf14;slamf1;scap;malt1;prkaa1;c1qtnf2;psmc5;cdk6;klf15;zbtb38;hcls1;gch1;runx1;plagl2;eda2r;trim13;runx3;pax8;ift88 |
| cellular process#regulation of cellular process; | 537#5704 | 1.7517E-21 | rgs4;ikzf1;tigd3;hoxd1;tshz3;znf566;traf3ip2;klf11;cdk9;hif1an;il3;plekho1;glis2;pstk;ets2;vsx1;cdx2;lalba;aplp1;znf41;rasa1;sirt3;eif3f;zbtb33;nrp1;cd70;kpna2;smad6;edn1;traf3;cdh13;myog;runx1t1;prdm14;clic4;iqgap2;zxdc;msx1;rdbp;zbtb25;dyrk1b;dffa;smap1;bace2;eif4g2;nr4a1;supt4h1;alx1;rgs21;sirt1;rbl2;twist1;bhmt;traf6;elf5;irf5;ran;lats2;trib3;ldlrap1;myd88;cables1;hdac10;fgd1;tcf23;fubp3;zbtb45;ccnd3;net1;zbtb32;nras;mmp9;ppm1d;bcl6;senp2;gsc;kiss1r;usf1;atf7;stat3;sp1;tgfbi;maff;socs1;brms1l;znf501;cbfa2t2;ddb1;pth2r;junb;mlxipl;foxc1;ripk2;htr1a;tgfb2;hoxd8;azu1;ezh2;meis3;bclaf1;nbl1;yap1;ihh;ngfr;igf2bp3;cux1;zbtb16;hoxc9;znf768;igfbp6;polh;irx3;sfpq;mtf1;znf581;psd4;prrx1;srpk1;cdk6;skap2;hcls1;bub1b;creb3;rnf2;dph1;ift88;sectm1;ssrp1;eya3;pogk;slc20a1;rapgef4;ikzf3;dnm2;camk2d;cdca7;capza3;cx3cl1;ripply1;zscan21;hmgb4;mkl1;tox3;taok2;esrrg;znf446;rcor2;rfx5;cdkn2aip;tlk1;pim1;rapgef1;tox;tp53bp2;samd4a;ddr2;cited2;trim38;zbtb20;ebag9;dek;duxa;zfx;tbx10;traf1;med8;sp2;rgs1;atp2c1;nlk;pbrm1;znfx1;znf496;ctnna1;tgfb1;mapk1;elk4;ghsr;phtf1;ncoa2;drgx;adra1b;tceal8;eif5;egln2;mixl1;tox4;hus1;eya4;ctnnb1;fxyd5;elavl2;bnipl;polr2f;znf354a;capza2;rax;znf451;lep;l3mbtl3;scnn1b;hdac9;flt1;dcc;nrcam;adamtsl4;hoxb3;ppargc1b;taok3;nedd4l;eef1e1;pias1;set;klf5;rab8a;htra1;rhob;cdk10;hspd1;tsc22d4;ltbr;mysm1;tead1;spaca3;sim2;elf1;lgals9;egr2;gabpb2;mcl1;hectd3;pcgf1;eif3e;ing1;arid5a;slamf1;onecut2;scap;alb;stat1;creg1;wisp1;psmc5;c1qtnf2;dlx3;runx1;esm1;sycp3;tax1bp1;eda2r;foxp4;sap30bp;znf558;znf33b;prkra;nr4a2;znf548;dkk2;plekhf1;cdk2ap1;iqsec3;pparg;cdc16;bcl2l11;sumo1;znf414;lcor;vps72;gab1;nr5a1;tnfrsf25;rgs17;cdc42bpb;ramp1;sqstm1;traf7;neurod6;cdc42ep5;gmeb2;pak1ip1;chd3;ciapin1;capg;fubp1;iqcb1;gna13;rasa4;chaf1a;ikzf4;ern1;znf664;keap1;cd2;med20;nfatc2;zfp2;eif1b;cnbp;sesn1;polr3c;dlx2;hmgn2;foxj3;ccnt2;zfp42;atf3;pou3f2;cdc37;tlr2;rbm14;arhgdia;znf214;bcl11b;sox9;pou4f3;utf1;rab10;rab37;vgll4;pou2f1;clock;znf605;gtf2e1;diras3;nrg3;gsk3b;hey2;cdc73;trim54;nck1;ddx54;shc1;elk1;znf546;cnot6;rasgrp2;alox12;med23;klf12;znf197;vax1;uhrf2;serpina5;iqsec2;hoxa3;sema4d;ctbp2;rgs11;wars;prkca;pram1;arfgap1;adam10;tlx3;znf175;grip1;zfp1;taf4;adm;sohlh2;znf710;wnt2;dlx5;vegfa;eid2;cdkn3;bub3;tfe3;tbc1d14;med18;slc30a9;il20;ppie;ing3;madd;whsc2;nr5a2;taf6;rnf14;six6;arhgef9;malt1;eif2ak2;prkaa1;zscan29;rassf5;hdx;sp5;znf771;hells;zbtb12;hoxc12;klf15;pum2;scml1;vsig4;rarb;plagl2;trim13;ovol2;pax8;runx3;brf2;lcorl;habp4;meox2;actn4;asz1;foxn1;znf524;grm8;supt16h;sox15;klf1;znf787;nfkbil1;evi5;nr0b2;lancl2;gas2;nfatc2ip;znf786;pdcd5;scmh1;dcbld2;znf623;znf688;arhgef6;tnp1;cd274;arfgef2;eif4h;pigu;gja1;prdm12;eef1d;cdkn2d;trim24;gabpa;uimc1;sox6;pcna;birc3;rbm5;crim1;nlrp2;sirt5;arhgdib;mad2l2;pknox1;nusap1;nanog;rad51;pld2;ppp1r15a;cda;acvr2b;tfap2c;pdcd4;ilf3;prpf6;hif3a;znf24;snip1;clcf1;cnot4;pnn;aurkaip1;tnfaip3;cgref1;kng1;synj2bp;abt1;grhl2;rgs22;rcc1;ilf2;scml2;btk;ctcf;tcea3;cckbr;spry1;hspb1;pcgf2;npas1;dedd;tbpl2;krba2;crebzf;faim;rasal2;crem;znf689;casz1;terf2;cd28;sirt4;npm2;cd74;znf131;cdc42ep4;ccng1;sox5;prdm4;fstl3;sfn;elf3;znf398;crx;vav3;atf2;dbx2;znf454;nr6a1;prtn3;snd1;cxxc4;med24;dmpk;bik;gtf2a1;mapk8ip1;gzf1;tspyl2;igsf1;nab1;rfx3;brsk1;epc2;eif3i;cd164;zbtb38;gch1;mecp2;cdt1;rad1 |
| multicellular organismal development#system development; | 200#1605 | 1.7517E-21 | sectm1;amtn;ikzf1;dscaml1;asz1;pcdh18;foxn1;dmc1;myl4;ikzf3;nr4a2;prkra;tfip11;sox15;sema3a;chst8;klf1;comp;dctn1;sema3d;fhl1;mdga2;fez1;nr5a1;ablim1;pcdhb5;il3;traf7;neurod6;myl1;tbcb;sparc;rbm45;glis2;cited2;ets2;pcdh1;ap3d1;cdx2;iqcb1;tgm5;itgb1bp2;aplp1;gna13;serpinf1;fgf5;rasa1;plp1;gnpat;nrp1;dpysl3;krt13;shroom2;edn1;cdh13;gja1;gfra3;myog;scrg1;keap1;cd2;slc5a3;sox6;trim3;atp2c1;plod1;slitrk3;crim1;dlx2;npr3;clec3b;prkci;ctnna1;atp2a2;tgfb1;msx1;pou3f2;adam12;mapk1;vps33a;prelp;sox9;pou4f3;ghsr;map4k2;sgca;mobp;pcdhb11;adra1b;kcnq2;klk7;snai1;alx1;efnb3;sirt1;vgf;ankrd2;cck;clcf1;twist1;nrg3;adora2b;hey2;minpp1;ctnnb1;prdx1;matn1;aldh5a1;mdga1;pafah1b3;krt5;lep;fgf1;ugcg;b3gnt5;bpgm;hdac9;flt1;btk;sema4d;jmjd6;fgd1;spry1;dcc;ipmk;nrcam;utp3;hoxb3;nras;mmp9;fgf6;bcl6;npas1;cyfip1;chrm2;tlx3;b3gnt2;tinag;fzd9;klf5;pcdhb6;stat3;slitrk4;rhob;adm;maff;doc2a;snrk;wnt2;sema4a;dlx5;krt9;pth2r;gaa;chrna4;vegfa;foxc1;cd74;sim2;mbp;egr2;tgfb2;azu1;sfn;il20;elf3;mpped2;crx;spock1;kitlg;pdgfc;sgcb;eln;ihh;krt1;ngfr;cacna1f;cux1;nde1;zbtb16;six6;onecut2;gzf1;papss1;ostf1;cln5;cdk6;sgcg;trpc5;hcls1;ndrg2;runx1;dlx3;bves;rufy3;pts;drp2;hexa;eda2r;ift88 |
| cell differentiation#cell development; | 163#1242 | 3.29589E-20 | kiaa1967;cd14;actn4;sap30bp;dscaml1;asz1;dnm2;nr4a2;prkra;sox15;capza3;plekhf1;klf1;mdga2;bcl2l11;fez1;vdac1;taok2;tnfrsf25;gas2;casp7;il3;pdcd2;sqstm1;traf7;pdcd5;pim1;ndufs1;tp53bp2;arhgef6;ciapin1;lalba;tnp1;aplp1;serpinf1;rasa1;ebag9;nrp1;cd70;edn1;traf3;cdh13;gja1;myog;ern1;cdkn2d;cd2;traf1;birc3;slitrk3;nlrp2;elmo1;prkci;pak1;dnm1l;ctnna1;tgfb1;pou3f2;adam12;mapk1;tlr2;dffa;arhgdia;dusp22;ppp1r15a;kcne1;sox9;pou4f3;asah2;map4k2;eif4g2;pdcd4;efnb3;sirt1;cck;clcf1;gsk3b;rragc;traf6;pdia2;ctnnb1;bnipl;tnfaip3;alox12;bmf;kng1;mdga1;trib3;bpgm;btk;krt20;sema4d;jmjd6;dcc;nrcam;adamtsl4;bcl7c;hspb1;prkca;nras;mmp9;fgf6;bcl6;apip;cyfip1;il17a;b3gnt2;dedd;tlx3;eef1e1;sgpl1;fzd9;clul1;slitrk4;rhob;cep57;hspd1;faim;wnt2;tns4;dlx5;pth2r;mmd;ltbr;cd28;vegfa;foxc1;cd74;fzd5;ripk2;lyz;tgfb2;azu1;sfn;znf346;mcl1;bclaf1;madd;pacs2;ihh;ngfr;cacna1f;bik;zbtb16;ppp2r2b;malt1;eif2ak2;prkaa1;rassf5;alb;atg12;stat1;ccar1;cln5;optn;psmc5;cdk6;gch1;bub1b;sycp3;plagl2;tax1bp1;runx3 |
| anatomical structure morphogenesis; | 143#1047 | 9.88814E-20 | prickle2;amtn;eya3;dscaml1;asz1;foxn1;mark2;camk2d;prkra;sox15;klf1;comp;fhl1;fez1;taok2;ablim1;cdc42bpb;gas2;cdkn2aip;il3;cdc42ep5;scmh1;dcbld2;capg;cdx2;aplp1;gna13;serpinf1;rasa1;gp1ba;ebag9;gnpat;nrp1;shroom2;edn1;cdh13;gja1;keap1;cdkn2d;tbx10;slitrk3;crim1;prkci;ctnna1;dhrs9;tshb;tgfb1;msx1;pou3f2;mapk1;mid1;kcne1;cda;pou4f3;kcnmb4;adra1b;snai1;efnb3;cck;twist1;nrg3;adora2b;hey2;nck1;rragc;egln2;shc1;eya4;fxyd5;ctnnb1;alox12;kng1;fgf1;akap4;ca9;flt1;scml2;sema4d;fgd1;spry1;nrcam;ipmk;dcc;net1;fgf6;bcl6;gsc;cyfip1;nts;b3gnt2;tinag;klf5;slitrk4;htra1;rhob;maff;wnt11;brms1l;socs1;wnt2;dlx5;gaa;mlxipl;vegfa;foxc1;fzd5;cdc42ep4;tgfb2;il20;elf3;th;crx;ing3;kitlg;ihh;eln;krt1;ngfr;nr5a2;cacna1f;igf2bp3;mcam;rhcg;ing1;six6;igfbp6;onecut2;gzf1;nle1;tspyl2;ca2;creg1;wisp1;mrpl40;gch1;runx1;scml1;esm1;fgf20;thbs4;trim13;ift88;pax8 |
| transcription from RNA polymerase II promoter; | 99#640 | 1.52166E-18 | elk1;elf5;ctnnb1;foxn1;med23;polr2f;znf354a;ikzf3;nr4a2;sox15;supt16h;abt1;klf12;myd88;klf1;pparg;ctcf;lcor;klf11;cdk9;zbtb32;vps72;nr5a1;nr0b2;ppargc1b;rfx5;bcl6;npas1;sqstm1;gmeb2;usf1;tbpl2;klf5;stat3;sp1;chd3;cited2;vsx1;cdx2;fubp1;cops2;maff;dek;junb;foxc1;myog;sim2;elf1;trim24;tfe3;gabpa;sox5;prdm4;med20;gabpb2;tbx10;elf3;sp2;crx;nr6a1;yap1;ihh;med24;nr5a2;dlx2;pknox1;gtf2a1;cux1;rnf14;znf768;ccnt2;gzf1;msx1;pou3f2;rbm14;mtf1;stat1;creg1;trip13;sox9;utf1;pou4f3;elk4;tfap2c;psmc5;ncoa2;zbtb38;supt4h1;runx1;adrm1;prpf6;alx1;polr2d;mecp2;plagl2;clock;twist1;runx3;gtf2e1 |
| cell death; | 124#908 | 5.57856E-17 | kiaa1967;cd14;rragc;traf6;pdia2;actn4;sap30bp;alox12;tnfaip3;bnipl;bmf;kng1;dnm2;prkra;trib3;plekhf1;btk;krt20;sema4d;jmjd6;bcl2l11;dcc;adamtsl4;bcl7c;hspb1;vdac1;prkca;nras;taok2;mmp9;tnfrsf25;gas2;bcl6;casp7;apip;il3;pdcd2;sqstm1;traf7;il17a;pdcd5;dedd;pim1;eef1e1;sgpl1;ndufs1;clul1;tp53bp2;arhgef6;ciapin1;rhob;lalba;aplp1;hspd1;faim;rasa1;tns4;ebag9;mmd;ltbr;cd70;cd28;vegfa;foxc1;traf3;cdh13;cd74;gja1;ripk2;ern1;lyz;cdkn2d;cd2;tgfb2;azu1;sfn;traf1;znf346;birc3;mcl1;madd;nlrp2;bclaf1;pacs2;ihh;elmo1;ngfr;bik;zbtb16;pak1;dnm1l;ctnna1;ppp2r2b;malt1;tgfb1;mapk1;tlr2;prkaa1;eif2ak2;dffa;rassf5;alb;arhgdia;dusp22;ppp1r15a;stat1;atg12;ccar1;sox9;pou4f3;asah2;eif4g2;optn;psmc5;pdcd4;bub1b;gch1;sycp3;sirt1;plagl2;tax1bp1;clcf1;gsk3b;runx3 |
| death; | 124#908 | 5.57856E-17 | kiaa1967;cd14;rragc;traf6;pdia2;actn4;sap30bp;alox12;tnfaip3;bnipl;bmf;kng1;dnm2;prkra;trib3;plekhf1;btk;krt20;sema4d;jmjd6;bcl2l11;dcc;adamtsl4;bcl7c;hspb1;vdac1;prkca;nras;taok2;mmp9;tnfrsf25;gas2;bcl6;casp7;apip;il3;pdcd2;sqstm1;traf7;il17a;pdcd5;dedd;pim1;eef1e1;sgpl1;ndufs1;clul1;tp53bp2;arhgef6;ciapin1;rhob;lalba;aplp1;hspd1;faim;rasa1;tns4;ebag9;mmd;ltbr;cd70;cd28;vegfa;foxc1;traf3;cdh13;cd74;gja1;ripk2;ern1;lyz;cdkn2d;cd2;tgfb2;azu1;sfn;traf1;znf346;birc3;mcl1;madd;nlrp2;bclaf1;pacs2;ihh;elmo1;ngfr;bik;zbtb16;pak1;dnm1l;ctnna1;ppp2r2b;malt1;tgfb1;mapk1;tlr2;prkaa1;eif2ak2;dffa;rassf5;alb;arhgdia;dusp22;ppp1r15a;stat1;atg12;ccar1;sox9;pou4f3;asah2;eif4g2;optn;psmc5;pdcd4;bub1b;gch1;sycp3;sirt1;plagl2;tax1bp1;clcf1;gsk3b;runx3 |
| protein kinase cascade; | 66#376 | 2.98493E-16 | sectm1;adora2b;rgs4;shc1;traf6;slc20a1;map3k9;tnfaip3;mark2;dusp10;lats2;map3k7;map3k4;myd88;traf3ip2;gab1;prkca;taok2;tec;taok3;il3;sqstm1;traf7;dbnl;cdc42ep5;pias1;ccr2;arhgef6;stat3;trim38;sh2d3c;socs1;gna13;ltbr;pigu;oxsr1;gja1;edn1;ripk2;eef1d;lgals9;il20;atp2c1;nlk;madd;mapk8ip1;map3k2;malt1;jak1;prkaa1;mapk1;stat1;dusp22;map2k7;map4k3;mark1;map4k2;c1qtnf2;srpk1;hcls1;adra1b;snip1;trim13;clcf1;eda2r;nrg3 |
| programmed cell death; | 118#862 | 3.02837E-16 | kiaa1967;cd14;rragc;traf6;pdia2;actn4;sap30bp;alox12;tnfaip3;bnipl;bmf;kng1;dnm2;prkra;trib3;plekhf1;btk;krt20;sema4d;jmjd6;bcl2l11;dcc;adamtsl4;bcl7c;hspb1;vdac1;prkca;nras;taok2;mmp9;tnfrsf25;gas2;bcl6;casp7;apip;il3;pdcd2;sqstm1;traf7;il17a;pdcd5;dedd;pim1;eef1e1;sgpl1;ndufs1;tp53bp2;arhgef6;ciapin1;rhob;lalba;aplp1;hspd1;faim;rasa1;tns4;ebag9;ltbr;cd70;cd28;vegfa;foxc1;traf3;cdh13;cd74;gja1;ripk2;ern1;cdkn2d;cd2;tgfb2;azu1;sfn;traf1;znf346;birc3;mcl1;madd;nlrp2;bclaf1;pacs2;ihh;elmo1;ngfr;bik;zbtb16;pak1;dnm1l;ctnna1;malt1;tgfb1;mapk1;tlr2;prkaa1;eif2ak2;rassf5;dffa;alb;arhgdia;dusp22;ppp1r15a;stat1;atg12;ccar1;sox9;pou4f3;asah2;psmc5;pdcd4;bub1b;gch1;sycp3;sirt1;plagl2;tax1bp1;clcf1;gsk3b;runx3 |
| apoptosis; | 117#855 | 4.24391E-16 | kiaa1967;cd14;rragc;traf6;pdia2;actn4;sap30bp;alox12;tnfaip3;bnipl;bmf;kng1;dnm2;prkra;trib3;plekhf1;btk;krt20;sema4d;jmjd6;bcl2l11;dcc;adamtsl4;bcl7c;hspb1;vdac1;prkca;nras;taok2;mmp9;tnfrsf25;gas2;bcl6;casp7;apip;il3;pdcd2;sqstm1;traf7;il17a;pdcd5;dedd;pim1;eef1e1;sgpl1;ndufs1;tp53bp2;arhgef6;ciapin1;rhob;lalba;aplp1;hspd1;faim;rasa1;tns4;ebag9;ltbr;cd70;cd28;vegfa;foxc1;traf3;cdh13;cd74;gja1;ripk2;ern1;cdkn2d;cd2;tgfb2;azu1;sfn;traf1;znf346;birc3;mcl1;madd;nlrp2;bclaf1;pacs2;ihh;elmo1;ngfr;bik;zbtb16;pak1;dnm1l;ctnna1;malt1;tgfb1;mapk1;tlr2;prkaa1;eif2ak2;rassf5;dffa;alb;arhgdia;dusp22;ppp1r15a;stat1;atg12;ccar1;sox9;pou4f3;asah2;pdcd4;bub1b;gch1;sycp3;sirt1;plagl2;tax1bp1;clcf1;gsk3b;runx3 |
| cell proliferation; | 103#745 | 1.81051E-14 | nck1;shc1;elf5;ctnnb1;asz1;foxn1;prdx1;alox12;cgref1;bnipl;cdca7;prkra;myd88;uhrf2;fgf1;flt1;cckbr;cdc16;ctbp2;klf11;wars;cdk9;e4f1;gfer;gab1;evi5;nras;fgf6;ppm1d;bcl6;il3;appl1;kiss1r;pim1;eef1e1;isg20;areg;fzd9;klf5;ddr2;tgfbi;adm;hdgf;cdk10;serpinf1;fgf5;cd274;wnt2;nrp1;pth2r;cd70;cd28;vegfa;foxc1;edn1;cdh13;cd74;gja1;ripk2;eid2;cdkn2d;bub3;htr1a;cdkn3;prdm4;tgfb2;sfn;pcna;lrpap1;spock1;kitlg;sesn1;pdgfc;nr6a1;eln;ihh;prtn3;tspan2;ing1;igfbp6;slamf1;nanog;ctnna1;tgfb1;pou3f2;eif2ak2;dusp22;creg1;fscn1;sox9;hells;cdc27;cdk6;skap2;pdgfd;cd164;hcls1;insig1;adra1b;mia;bub1b;vsig4;runx3 |
| cellular metabolic process; | 994#12668 | 3.0911E-14 | pigq;ttbk1;exosc10;fkbp3;ikzf1;nox1;cfb;fbxo10;tigd3;usp30;pla2g12a;mcee;hoxd1;tshz3;myo3a;nrbp1;nsf;psmc2;znf566;slc27a2;msrb3;gphn;arg1;fut1;sucla2;st13;klf11;cdk9;tspan8;chst14;sf3a2;tec;hif1an;il3;naprt1;plod2;adam20;arih1;st3gal3;rrm2b;st6galnac4;phkg2;pop5;rbbp6;glis2;pstk;rnf139;ets2;vsx1;cdx2;apobec2;aplp1;hgsnat;znf41;rasa1;sirt3;eif3f;zbtb33;phka2;gnpat;dpysl3;kpna2;smad6;edn1;akt2;myog;xpo5;runx1t1;xpo6;uchl5;prdm14;snrpe;mex3b;cstf1;calr3;mki67ip;usp39;zxdc;rpl8;wdr36;mrpl44;usp32;aprt;rbm23;abl2;dcp2;msx1;adam12;rdbp;zbtb25;dyrk1b;dffa;usp15;fbxl8;bace2;zcrb1;impa2;asah2;map4k2;eif4g2;nr4a1;mrps35;supt4h1;klk7;alx1;sirt1;gin1;ldhb;hectd2;mrpl3;f7;rbl2;twist1;usp13;bhmt;h2afz;traf6;ogt;elf5;lysmd3;epha3;irf5;ran;sdf2;pcbp3;ucn2;tst;lats2;tbcc;srd5a1;trib3;ldlrap1;myd88;cdc14b;fbxl22;ca9;hdac10;nfkbib;st6gal1;hsp90aa1;tcf23;fubp3;phkg1;zbtb45;pdxdc1;ccnd3;zbtb32;flrt2;l2hgdh;mmp9;tmub2;ints5;dlst;ppm1d;bcl6;senp2;gsc;usf1;adam21;atf7;stard5;grpel2;stat3;sp1;camk4;gnpda1;pfkfb2;cpb1;c4bpb;maff;atp6v1d;socs1;brms1l;znf501;b3galt1;snrk;cbfa2t2;clk3;ddb1;junb;mlxipl;fbxo45;foxc1;ripk2;lyz;pgam2;tgfb2;hoxd8;xrcc2;azu1;cbs;osbpl2;ezh2;znf346;meis3;ctsw;bclaf1;ppic;tsfm;yap1;mrps9;ihh;me2;igf2bp3;cux1;zbtb16;hoxc9;znf768;proca1;polh;irx3;sfpq;mtf1;slbp;znf581;dio3;mmp12;serinc1;prrx1;srpk1;cdk6;fance;trpc5;insig1;hcls1;bub1b;creb3;gns;trmu;inpp1;ndufv1;pts;rnf2;sphar;hyal2;senp8;dph1;pygl;ift88;arv1;klk13;hunk;ssrp1;utp6;mrps18c;prepl;mrpl30;ckm;eya3;pogk;slc20a1;cpa5;prkag2;asb2;rapgef4;brs3;ikzf3;camk2d;dnm2;cdca7;hamp;apeh;ca3;capza3;ripply1;zscan21;gpaa1;g6pd;sh3rf2;hmgb4;mkl1;mmab;adprh;hadha;tktl1;tox3;inppl1;syncrip;taok2;esrrg;znf446;rcor2;galns;rfx5;cdkn2aip;tlk1;acsl3;pim1;tox;ndufs1;samd4a;ddr2;zdhhc7;cited2;ap3d1;galnt6;hibadh;ulk3;adat1;zbtb20;fen1;qpctl;ccnb1ip1;ttll5;dek;duxa;man1a1;maoa;atg7;rabggta;ppp4r2;zfx;tbx10;mrpl22;med8;sp2;mtap;plod1;nlk;chdh;galnt9;enpp2;pbrm1;tor2a;syvn1;znfx1;gnptg;pam;ppp1cb;eif1ax;znf496;prkci;pak1;tp53rk;map3k2;tgfb1;fdps;mapk1;dusp22;trip13;ace2;slmap;elk4;ppm1l;ghsr;cdyl2;rps29;hacl1;cdkl2;gucy2c;phtf1;klk9;cad;ncoa2;slc27a4;drgx;adra1b;tubg1;cct6b;slc23a2;ppp1cc;mgat4a;tceal8;aldh1a1;ogdh;bckdhb;eif5;amd1;uap1;rnf41;mixl1;tox4;eya4;hus1;dhx15;ctnnb1;elavl2;tysnd1;polr2f;scrn1;znf354a;uqcrh;capza2;rax;znf451;grsf1;mrpl16;fa2h;sc5dl;rpp14;lep;l3mbtl3;rdh11;bpgm;tspyl1;plaur;tgds;flt1;hdac9;usp2;cpa6;mapkapk5;pnpla3;rnps1;utp3;uroc1;hoxb3;scarf1;adat3;ppargc1b;taok3;rps10;il17a;nedd4l;csnk1a1;gpx4;mrpl37;oma1;eef1e1;pias1;set;klf5;rab8a;agmat;tnip1;htra1;cdk10;hspd1;cercam;tsc22d4;lrrc29;mysm1;tead1;spaca3;nap1l5;pigs;sim2;elf1;ugt1a9;egr2;rad23a;gfpt1;mrpl47;rad54l;gabpb2;th;dhx38;hectd3;atp6v1g3;pcgf1;pgd;ampd1;a4gnt;aoah;krt1;eif3e;olr1;srms;arid5a;atg4a;adk;onecut2;scap;ttc1;ptges;upp1;fbxo47;atg12;stat1;creg1;pycr1;psmc5;c1qtnf2;napg;gdpd3;aldh1l1;dlx3;runx1;ldhc;alkbh1;sycp3;impa1;capn2;acadsb;mrpl35;fasn;eda2r;foxp4;ptk2;ppil3;acaca;sap30bp;rbm3;ears2;spcs2;znf558;qprt;dmc1;mark2;dusp10;znf33b;znf548;prkra;nr4a2;tyr;tfip11;map3k7;map3k4;fkbp6;cdk2ap1;fdxr;pparg;ctss;c1qc;cdc16;sumo1;znf414;lcor;glyctk;ctsa;acox2;atp5o;vps72;nr5a1;fbxl17;afmid;rce1;cdc42bpb;acoxl;b3gnt4;sqstm1;traf7;cdc42ep5;neurod6;fancf;gmeb2;cds1;ddah1;ptpn18;chd3;pabpc4;prmt2;capg;fubp1;klk12;csnk1g3;n6amt2;abcf1;cpa3;acsm5;rrp9;chaf1a;ikzf4;acadl;znf664;ern1;keap1;med20;paics;kynu;nfatc2;zfp2;fech;cnbp;eif1b;mtmr7;npr2;cpxm1;polr3c;dlx2;hnrpll;hmgn2;ptpla;foxj3;mars2;ampd3;fbxl4;zfp42;ccnt2;pwp1;sord;atf3;pou3f2;rbm14;bcl11b;znf214;sox9;wars2;utf1;pou4f3;ppm1k;gdpd1;rab10;rab37;aurkc;ugdh;vgll4;tgm7;pcsk7;pou2f1;sncb;dpp8;lonp1;pdss1;polr2d;mrps18b;gfpt2;clock;znf605;prkaa2;pja2;gsk3b;gtf2e1;diras3;coro2a;cdc73;hey2;nck1;trim54;neu3;ddx54;sdha;elk1;soat1;znf546;srm;cnot6;psmd14;ero1lb;alox12;mrpl10;med23;aldh5a1;lrrk1;vax1;znf197;klf12;serpina5;uhrf2;dnajc17;hoxa3;ubqln4;dera;mvd;b3galt5;wars;slc25a10;prkca;nhp2l1;cpa4;ssu72;mthfd1l;senp3;mettl1;adam10;cept1;tlx3;b3gnt2;znf175;sugt1;ftsj2;tinag;grip1;zfp1;tmem48;nubp1;taf4;adm;cops2;sohlh2;psap;usp11;znf710;wnt2;ube2m;dlx5;oxsr1;gaa;mrpl36;araf;pank4;ndufb9;slc7a7;papd5;dus1l;eid2;tfe3;fbxo9;rpp38;cdkn3;il20;med18;fbxl13;slc30a9;scd;lrpap1;st3gal1;itk;lonrf1;sf3b2;rps8;ppie;ing3;whsc2;tinf2;nr5a2;taf6;agxt2l1;rnf14;qdpr;lrrc14;six6;cmas;fbxw8;malt1;zscan29;prkaa1;eif2ak2;abi2;papss1;map4k3;hdx;hars;znf771;sp5;hells;zbtb12;hoxc12;klf15;pum2;osbpl7;scml1;rbm17;ero1l;chst1;agk;aurkb;rarb;rrm2;pja1;plagl2;hexa;ovol2;ugt1a6;runx3;pax8;brf2;tpst1;acot9;habp4;lcorl;meox2;upf3a;ict1;asz1;foxn1;neil1;znf524;map3k9;grm8;bcat2;supt16h;sox15;chst8;klf1;znf787;hpse;sms;dcp1a;pdss2;pepd;lancl2;nr0b2;nfatc2ip;casp7;znf786;ldha;vrk1;pdk2;scmh1;znf623;sars;znf688;mttp;b3galt2;prps2;ptpn21;psph;tgm5;tnp1;tnks;senp1;herc6;eif4h;rexo2;aipl1;crnkl1;pigu;cfi;prdm12;eef1d;cdkn2d;trim24;gabpa;mrpl27;rbck1;apobec1;uimc1;chst12;sox6;pcna;rbm5;aloxe3;setdb2;atp6v0b;crim1;sirt5;hnrnpl;fut3;pknox1;skiv2l2;nanog;dhrs9;imp4;axl;adam32;rad51;gcnt4;mid1;pld2;map2k7;lcmt1;acvr2b;cda;klhl15;aco1;mark1;atp5b;dusp13;tfap2c;fip1l1;pdcd4;fbxo38;aldh7a1;slc38a4;ilf3;adrm1;prpf6;opn1sw;hif3a;clp1;znf24;snip1;stk39;clcf1;pgk2;cnot4;pnn;ubtd2;rragc;nek8;minpp1;slc17a4;aurkaip1;anapc7;ephx1;pdia2;idh3a;tnfaip3;twf1;elovl7;asphd1;abt1;lrrc41;grhl2;rcc1;b3gnt5;ugcg;camk2g;scml2;ilf2;btk;ctcf;tcea3;exosc7;isca1;psma8;ipmk;rps3;hspb1;rpp30;pole3;ncln;zmat5;nt5c1a;npas1;farsa;pcgf2;cryl1;cndp2;rplp1;ftsj1;dedd;tnp2;sgpl1;isg20;tbpl2;pak3;psmb5;cdc40;rnpc3;hadh;alg3;krba2;crebzf;trub1;pip4k2a;usp33;smc5;st3gal4;cog2;znf689;crem;terf2;casz1;ca13;ttll9;dck;sirt4;cd28;npm2;cd74;znf131;pigb;sox5;prdm4;elf3;wee2;crx;trub2;znf398;hnrnpr;dbx2;atf2;gan;nr6a1;znf454;snd1;epha1;prtn3;dmpk;med24;fbp2;rpl9;gtf2a1;acly;pskh1;gzf1;ubqln2;lman1;jak1;tspyl2;tubb6;ca2;igsf1;rsl1d1;nsun2;rfx3;nab1;brsk1;cstf2t;hk3;denr;nuak1;epc2;eif3i;trove2;zbtb38;pdha1;gch1;mecp2;nars;cdt1;dot1l;gmps;dbh;scnm1;pnmt;st8sia5;dnaja4;rnaseh1;adad1;rad1;fbxl6 |
| localization of cell#cell motility; | 64#383 | 3.76137E-14 | nck1;cxcl16;actn4;asz1;alox12;mdga1;capza2;cx3cl1;scnn1b;akap4;plaur;flt1;dcc;nrcam;spint2;hspb1;fez1;gab1;prkca;taok2;bcl6;kiss1r;tlx3;b3gnt2;tnp2;coro1a;stat3;il13;cabp4;tnp1;cercam;gna13;wnt2;nrp1;dnali1;vegfa;gja1;cdh13;foxc1;shroom2;tgfb2;azu1;mkln1;clic4;spock1;dnah12;enpp2;cd34;arhgdib;elmo1;ltb4r;tpm1;ngfr;tspan2;tpm4;tgfb1;arpc1b;abi2;arhgdia;pou4f3;mmp12;efnb3;cck;thbs4 |
| localization of cell; | 64#383 | 3.76137E-14 | nck1;cxcl16;actn4;asz1;alox12;mdga1;capza2;cx3cl1;scnn1b;akap4;plaur;flt1;dcc;nrcam;spint2;hspb1;fez1;gab1;prkca;taok2;bcl6;kiss1r;tlx3;b3gnt2;tnp2;coro1a;stat3;il13;cabp4;tnp1;cercam;gna13;wnt2;nrp1;dnali1;vegfa;gja1;cdh13;foxc1;shroom2;tgfb2;azu1;mkln1;clic4;spock1;dnah12;enpp2;cd34;arhgdib;elmo1;ltb4r;tpm1;ngfr;tspan2;tpm4;tgfb1;arpc1b;abi2;arhgdia;pou4f3;mmp12;efnb3;cck;thbs4 |
| multicellular organismal development#system development#organ development; | 140#1141 | 4.4246E-14 | sectm1;amtn;ikzf1;dscaml1;asz1;pcdh18;foxn1;dmc1;myl4;ikzf3;prkra;tfip11;sox15;klf1;comp;fhl1;nr5a1;ablim1;il3;myl1;sparc;cited2;ets2;ap3d1;cdx2;iqcb1;tgm5;itgb1bp2;aplp1;gna13;serpinf1;rasa1;gnpat;nrp1;krt13;shroom2;edn1;cdh13;gja1;myog;keap1;cd2;sox6;atp2c1;plod1;dlx2;npr3;clec3b;prkci;atp2a2;tgfb1;msx1;pou3f2;adam12;mapk1;vps33a;prelp;sox9;pou4f3;ghsr;map4k2;sgca;adra1b;klk7;snai1;alx1;sirt1;vgf;ankrd2;clcf1;twist1;nrg3;adora2b;hey2;minpp1;ctnnb1;prdx1;matn1;mdga1;krt5;lep;fgf1;ugcg;bpgm;hdac9;flt1;btk;jmjd6;spry1;fgd1;utp3;hoxb3;nras;mmp9;fgf6;bcl6;tinag;klf5;rhob;adm;maff;wnt2;snrk;dlx5;krt9;pth2r;gaa;vegfa;cd74;foxc1;sim2;egr2;tgfb2;sfn;il20;elf3;crx;kitlg;sgcb;ihh;eln;krt1;ngfr;cux1;zbtb16;six6;onecut2;gzf1;papss1;cln5;ostf1;cdk6;sgcg;hcls1;runx1;dlx3;bves;hexa;eda2r;ift88 |
| cell cycle process; | 90#625 | 4.4246E-14 | cdc73;pnn;aurkaip1;shc1;anapc7;hus1;dmc1;cgref1;ran;dnm2;lats2;uhrf2;ndc80;rcc1;cables1;cdk2ap1;dctn1;ctcf;cdc16;dcc;ccnd3;gas2;bcl6;sugt1;tp53bp2;coro1a;plekho1;cited2;rhob;cdk10;ddb1;cd28;kpna2;npm2;foxc1;papd5;ccng1;ern1;cdkn2d;bub3;cdkn3;tgfb2;xrcc2;rad54l;sfn;rbm5;hectd3;cntrob;sesn1;madd;nbl1;pbrm1;mad2l2;nde1;ing1;akap8;ccnt2;nusap1;ncaph;tgfb1;tspyl2;cdc37;rad51;rassf5;ppp1r15a;brsk1;eif4g2;hells;cdc27;dusp13;cdk6;mns1;pdcd4;ilf3;bub1b;stag1;tubg1;sycp3;aurkb;cdt1;pds5a;sac3d1;sgol1;dph1;rad1;ncapg;trim13;rbl2;diras3;runx3 |
| primary metabolic process; | 999#12764 | 4.49956E-14 | pigq;ttbk1;exosc10;fkbp3;ikzf1;nox1;cfb;fbxo10;tigd3;usp30;pla2g12a;hoxd1;tshz3;myo3a;nrbp1;nsf;psmc2;znf566;slc27a2;msrb3;arg1;fut1;sucla2;lrp10;st13;klf11;cdk9;tspan8;chst14;sf3a2;tec;hif1an;il3;naprt1;plod2;adam20;arih1;st3gal3;rrm2b;st6galnac4;phkg2;pop5;rbbp6;glis2;pstk;rnf139;ets2;vsx1;cdx2;lalba;apobec2;aplp1;hgsnat;znf41;rasa1;sirt3;eif3f;zbtb33;phka2;gnpat;dpysl3;kpna2;smad6;edn1;akt2;myog;xpo5;runx1t1;xpo6;uchl5;prdm14;snrpe;mex3b;cstf1;calr3;mki67ip;usp39;zxdc;rpl8;wdr36;mrpl44;usp32;aprt;rbm23;abl2;dcp2;msx1;adam12;rdbp;zbtb25;dyrk1b;dffa;usp15;fbxl8;bace2;zcrb1;asah2;map4k2;eif4g2;nr4a1;supt4h1;klk7;alx1;sirt1;gin1;ldhb;hectd2;mrpl3;f7;rbl2;twist1;usp13;bhmt;h2afz;traf6;ogt;elf5;epha3;irf5;ran;sdf2;pcbp3;ucn2;lats2;tbcc;srd5a1;trib3;ldlrap1;myd88;cdc14b;fbxl22;khk;hdac10;nfkbib;st6gal1;hsp90aa1;tcf23;fubp3;phkg1;zbtb45;ccnd3;fuca2;leprel2;zbtb32;flrt2;l2hgdh;mmp9;tmub2;ints5;dlst;ppm1d;bcl6;senp2;gsc;usf1;adam21;atf7;stard5;grpel2;stat3;sp1;camk4;gnpda1;pfkfb2;cpb1;c4bpb;maff;atp6v1d;socs1;brms1l;znf501;b3galt1;snrk;cbfa2t2;clk3;ddb1;junb;mlxipl;fbxo45;foxc1;ripk2;pgam2;tgfb2;hoxd8;xrcc2;azu1;cbs;osbpl2;ezh2;znf346;tprkb;meis3;ctsw;bclaf1;kpna3;ppic;tsfm;yap1;mrps9;ihh;me2;igf2bp3;cux1;zbtb16;hoxc9;znf768;proca1;polh;irx3;sfpq;mtf1;slbp;znf581;cln5;dio3;mmp12;serinc1;prrx1;srpk1;cdk6;skap2;fance;trpc5;insig1;hcls1;bub1b;creb3;gns;trmu;pts;rnf2;sphar;hyal2;senp8;dph1;pygl;ift88;arv1;klk13;hunk;ssrp1;utp6;mrps18c;prepl;mrpl30;ckm;eya3;pogk;cpa5;slc2a2;prkag2;asb2;rapgef4;brs3;ikzf3;camk2d;dnm2;cdca7;apeh;capza3;ripply1;zscan21;gpaa1;g6pd;sh3rf2;hmgb4;mkl1;adprh;hadha;tktl1;tox3;syncrip;taok2;esrrg;znf446;rcor2;galns;rfx5;cdkn2aip;tlk1;acsl3;pim1;tox;ndufs1;samd4a;ddr2;zdhhc7;cited2;ap3d1;galnt6;hibadh;ulk3;adat1;zbtb20;fen1;qpctl;ccnb1ip1;tff1;ttll5;dek;duxa;man1a1;maoa;atg7;rabggta;ppp4r2;zfx;ap3s1;tbx10;mrpl22;med8;traf1;sp2;stbd1;mtap;plod1;nlk;galnt9;enpp2;pbrm1;tor2a;syvn1;znfx1;gnptg;pam;ppp1cb;eif1ax;znf496;ogfod1;prkci;pak1;tp53rk;ctnna1;map3k2;tgfb1;fdps;mapk1;dusp22;trip13;ace2;slmap;elk4;ppm1l;ghsr;cdyl2;rps29;hacl1;cdkl2;gucy2c;phtf1;klk9;cad;ncoa2;slc27a4;drgx;adra1b;tubg1;cct6b;slc23a2;ppp1cc;mgat4a;tceal8;ogdh;bckdhb;eif5;amd1;uap1;rnf41;egln2;mixl1;tox4;eya4;hus1;dhx15;ctnnb1;elavl2;tysnd1;matn1;polr2f;scrn1;znf354a;capza2;rax;znf451;grsf1;mrpl16;fa2h;sc5dl;rpp14;lep;l3mbtl3;rdh11;bpgm;tspyl1;plaur;flt1;hdac9;usp2;cpa6;mapkapk5;pnpla3;rnps1;pglyrp3;utp3;uroc1;hoxb3;scarf1;adat3;ppargc1b;taok3;rps10;il17a;nedd4l;csnk1a1;gpx4;mrpl37;oma1;eef1e1;pias1;set;klf5;ranbp9;rab8a;agmat;tnip1;htra1;cdk10;hspd1;cercam;tsc22d4;lrrc29;mysm1;tead1;spaca3;nap1l5;pigs;sim2;elf1;egr2;rad23a;gfpt1;mrpl47;rad54l;gabpb2;th;dhx38;hectd3;atp6v1g3;pcgf1;pgd;ampd1;a4gnt;aoah;p4ha1;krt1;eif3e;olr1;srms;arid5a;atg4a;adk;onecut2;scap;ttc1;ptges;upp1;fbxo47;atg12;stat1;creg1;pycr1;psmc5;c1qtnf2;napg;dlx3;runx1;ldhc;alkbh1;sycp3;impa1;capn2;acadsb;mrpl35;fasn;eda2r;foxp4;ptk2;ppil3;acaca;has3;sap30bp;rbm3;ears2;spcs2;znf558;qprt;dmc1;mark2;dusp10;znf33b;znf548;prkra;nr4a2;tyr;tfip11;map3k7;map3k4;fkbp6;cdk2ap1;fdxr;pparg;ctss;c1qc;cdc16;sumo1;znf414;lcor;glyctk;ctsa;acox2;atp5o;vps72;nr5a1;fbxl17;afmid;rce1;cdc42bpb;acoxl;b3gnt4;sqstm1;traf7;cdc42ep5;neurod6;fancf;gmeb2;cds1;ddah1;cldn14;ptpn18;chd3;pabpc4;prmt2;capg;fubp1;klk12;csnk1g3;abcf1;cpa3;acsm5;rrp9;chaf1a;ikzf4;acadl;znf664;ern1;keap1;med20;paics;kynu;nfatc2;zfp2;cnbp;eif1b;mtmr7;pltp;npr2;cpxm1;polr3c;dlx2;hnrpll;hmgn2;ptpla;foxj3;mars2;ampd3;fbxl4;zfp42;ccnt2;pwp1;sord;atf3;pou3f2;rbm14;bcl11b;znf214;sox9;wars2;utf1;pou4f3;ppm1k;rab10;rab37;ganab;aurkc;ugdh;vgll4;tgm7;pcsk7;pou2f1;sncb;dpp8;lonp1;pdss1;polr2d;mrps18b;gfpt2;clock;znf605;prkaa2;pja2;gsk3b;gtf2e1;diras3;coro2a;cdc73;hey2;nck1;trim54;neu3;ddx54;sdha;elk1;soat1;znf546;srm;cnot6;psmd14;ero1lb;alox12;mrpl10;ddhd1;med23;aldh5a1;lrrk1;vax1;znf197;klf12;serpina5;uhrf2;dnajc17;hyal3;hoxa3;ubqln4;dera;mvd;b3galt5;wars;slc25a10;prkca;nhp2l1;plcz1;cpa4;ssu72;senp3;mettl1;adam10;cept1;ap1s3;tlx3;b3gnt2;znf175;sugt1;ftsj2;tinag;ap1g1;grip1;zfp1;tmem48;nubp1;taf4;chka;adm;cops2;sohlh2;psap;usp11;znf710;wnt2;ube2m;dlx5;oxsr1;gaa;mrpl36;araf;slc7a7;papd5;dus1l;eid2;tfe3;fbxo9;rpp38;il20;med18;fbxl13;slc30a9;scd;lrpap1;st3gal1;itk;lonrf1;sf3b2;rps8;ppie;ing3;whsc2;tinf2;nr5a2;taf6;agxt2l1;rnf14;qdpr;lrrc14;six6;cmas;fbxw8;malt1;zscan29;prkaa1;eif2ak2;abi2;papss1;map4k3;hdx;picalm;hars;znf771;sp5;hells;zbtb12;hoxc12;klf15;pum2;osbpl7;scml1;rbm17;ero1l;chst1;agk;aurkb;rarb;rrm2;pja1;plagl2;hexa;ovol2;runx3;pax8;brf2;tpst1;acot9;habp4;lcorl;meox2;upf3a;ict1;asz1;foxn1;neil1;znf524;map3k9;grm8;bcat2;supt16h;sox15;chst8;klf1;znf787;hpse;sms;dcp1a;pdss2;pepd;lancl2;nr0b2;nfatc2ip;casp7;liph;znf786;ldha;vrk1;pdk2;scmh1;znf623;sars;znf688;mttp;b3galt2;ocrl;prps2;ptpn21;psph;tgm5;tnp1;tnks;senp1;herc6;eif4h;rexo2;aipl1;crnkl1;pigu;cfi;gja1;prdm12;eef1d;il1rap;copg2;cdkn2d;trim24;gabpa;mrpl27;rbck1;apobec1;uimc1;chst12;sox6;pcna;rbm5;aloxe3;setdb2;atp6v0b;crim1;sirt5;hnrnpl;fut3;pknox1;skiv2l2;nanog;dhrs9;imp4;axl;adam32;rad51;gcnt4;mid1;pld2;map2k7;lcmt1;acvr2b;cda;klhl15;aco1;mark1;atp5b;dusp13;tfap2c;fip1l1;pdcd4;trpv5;fbxo38;slc38a4;ilf3;adrm1;prpf6;opn1sw;hif3a;clp1;znf24;snip1;stk39;clcf1;pgk2;cnot4;pnn;ubtd2;rragc;nek8;aurkaip1;anapc7;lin7a;pdia2;idh3a;srd5a3;gga3;tnfaip3;twf1;pafah1b3;elovl7;asphd1;abt1;lrrc41;grhl2;rcc1;b3gnt5;ugcg;camk2g;scml2;ilf2;btk;ctcf;tcea3;exosc7;psma8;ipmk;rps3;hspb1;rpp30;pole3;ncln;zmat5;nt5c1a;npas1;farsa;pcgf2;cryl1;cndp2;rplp1;ftsj1;dedd;tnp2;sgpl1;isg20;tbpl2;pak3;psmb5;cdc40;rnpc3;hadh;alg3;krba2;crebzf;trub1;pip4k2a;usp33;smc5;st3gal4;cog2;znf689;crem;terf2;casz1;ttll9;dck;sirt4;cd28;npm2;cd74;znf131;pigb;sox5;prdm4;elf3;wee2;crx;trub2;znf398;hnrnpr;dbx2;atf2;gan;nr6a1;znf454;snd1;epha1;prtn3;dmpk;med24;fbp2;rpl9;gtf2a1;tubgcp4;acly;pskh1;gzf1;ubqln2;lman1;jak1;tspyl2;tubb6;igsf1;rsl1d1;nsun2;rfx3;nab1;brsk1;cstf2t;hk3;denr;nuak1;epc2;eif3i;trove2;zbtb38;pdha1;gch1;mecp2;nars;cdt1;dot1l;gmps;dbh;scnm1;pnmt;st8sia5;dnaja4;rnaseh1;adad1;rad1;fbxl6 |
| localization; | 411#4481 | 1.11724E-13 | stx10;snx15;btbd10;chml;nox1;nrbp1;nsf;chmp7;vps16;fdxr;lrp10;c1qc;vps52;sumo1;ctsa;atp5o;fez1;slc4a2;gab1;gabrb2;tomm22;sec61a2;necap1;cacng6;ramp1;sqstm1;traf7;vps26b;hook3;cacng7;slc17a7;slc30a4;trpc7;kifc3;aplp1;gna13;nrp1;kpna2;kcns3;edn1;cdh13;xpo5;cd2;stx4;c1qtnf1;xpo6;cthrc1;clic4;pltp;tpm1;elmo1;sv2b;akap8;dnm1l;rtp3;nupl2;atp11a;arpc1b;tlr2;cdc37;kctd18;vps33a;arhgdia;kcne1;snx27;bace2;pou4f3;map4k2;lrp6;rab10;rab37;kcnmb4;tmem37;colec11;rab33a;pkd2l1;kcnq2;pex12;tfrc;tm9sf2;cck;gsk3b;adora2b;c1qtnf3;nck1;sdha;aqp1;kcnc1;galr1;ero1lb;alox12;ran;tst;syt9;ykt6;slc18a2;uso1;ldlrap1;slc13a1;akap4;ca9;slc29a3;asgr2;srp72;kctd8;hsp90aa1;nxf1;slc31a1;stx7;abcd3;slc25a10;kcna2;prkca;pram1;slc7a6;snph;senp2;bcl6;slc6a2;arfgap1;sec61a1;slc4a8;gnai3;kiss1r;ap1s3;tlx3;b3gnt2;stard5;ap1g1;rabep2;stat3;cog7;tmem48;camk4;vps41;scn1b;chka;adm;atp6v1d;psap;rae1;doc2a;wnt2;slc30a6;slco6a1;gaa;grid2;chrna4;kcnj13;vegfa;ccbe1;foxc1;slc22a16;slc7a7;kpna6;tgfb2;syt12;azu1;osbpl2;slc30a9;plekha8;vps53;lrpap1;kif3b;spock1;clcn5;chmp4a;slc39a14;kpna3;cd93;slc2a12;ngfr;slc6a19;tspan2;rhcg;slc8a2;slco1b3;spns3;pacsin1;malt1;kif18a;klhl2;abi2;mmp12;kctd12;snx22;picalm;optn;slc9a7;serinc1;slc10a5;sec24b;klf15;trpc5;osbpl7;hcn4;kcnf1;ero1l;ift88;nudt9;slc25a43;cd14;ankrd27;upf3a;kif9;atp13a5;actn4;asz1;kcnk2;slc20a1;slc2a2;kctd9;pitpnc1;rapgef4;svop;dnm2;arfip1;scn1a;scamp2;kcnab3;cachd1;dnm3;cx3cl1;gpaa1;nup35;sergef;nfkbil1;gle1;scara5;rpgr;kctd19;vdac1;cyb561;abcg2;trpm8;taok2;pex16;tlk1;kcnip4;dbnl;clca1;chrna5;coro1a;acbd5;mttp;ap3d1;ywhab;slc16a4;tnp1;tnks;kcns1;arfgef2;slc22a18;shroom2;gja1;atg7;syt10;copg2;kcnj11;ap3s1;slc5a3;pcna;atp2c1;dnah12;kcnv2;atp6v0b;nlrp2;nalcn;enpp2;arhgdib;rab3c;ltb4r;stau2;gnptg;srp54;kif3a;prkci;ddx19b;nusap1;atp2a2;tgfb1;slc9a5;trpv1;pld2;kcnk6;shbg;exoc3;dync1i1;ghsr;atp5b;cacng3;kcnj5;slc37a3;sfxn3;vps26a;slc25a38;trpv5;slc27a4;slc38a4;efnb3;slc23a2;slc39a7;ergic3;rragc;slc17a4;duoxa2;cxcl16;lin7a;pdia2;ctnnb1;fxyd5;slc22a4;slc16a14;gga3;scrn1;slc16a3;kcng1;kng1;uqcrh;mdga1;capza2;heph;synj2bp;fa2h;snx25;cnnm2;rtp4;scnn1b;camk2g;stx16;plaur;flt1;krt20;best4;dcc;nrcam;kcnk1;spint2;hspb1;scarf1;cacna2d3;slc6a17;kcnq1;asna1;trappc6a;abcg8;slc30a10;nedd4l;tnp2;set;scfd1;best1;il13;rab8a;slc9a8;cabp4;slc22a14;rhob;slc39a6;cep57;cercam;hspd1;cog2;agtr1;snx14;cog5;chrne;kif1c;dnali1;cd74;spaca3;atp11b;kcnj12;rbp7;mkln1;cnga3;trappc1;slc25a17;atp6v1g3;snx5;tnpo3;cd34;tspan18;slc16a13;rab3ip;cacna1f;slc35f2;fxyd1;tpm4;stxbp1;atg4a;mapk8ip1;lman1;tubb6;csn2;ca2;cacnb3;alb;slc30a1;stat1;aqp11;col8a2;c1qtnf2;napg;lmbr1l;rab9b;atp2b3;slc6a5;kcna5;gosr1;thbs4 |
| positive regulation of metabolic process; | 64#391 | 1.50751E-13 | nck1;egln2;cd28;elk1;aurkaip1;foxc1;gja1;myog;ctnnb1;elf1;ran;azu1;il20;nfatc2;nr4a2;sox15;crx;myd88;yap1;ihh;ilf2;dlx2;ctcf;hsp90aa1;rnf14;scap;ccnd3;tgfb1;prkca;rbm14;prkaa1;esrrg;rad51;dyrk1b;ppargc1b;sox9;il3;utf1;sqstm1;cdc42ep5;ghsr;psmc5;c1qtnf2;pias1;samd4a;klf5;klf15;grip1;sp1;ncoa2;ilf3;zbtb38;hcls1;adra1b;supt4h1;glis2;runx1;cited2;prpf6;plagl2;clock;clcf1;pax8;ift88 |
| biopolymer metabolic process; | 662#7940 | 2.57923E-13 | pigq;ttbk1;exosc10;ikzf1;fbxo10;usp30;hoxd1;tshz3;myo3a;nrbp1;psmc2;znf566;fut1;klf11;cdk9;tspan8;sf3a2;tec;hif1an;il3;plod2;arih1;st3gal3;rrm2b;st6galnac4;phkg2;pop5;rbbp6;glis2;pstk;rnf139;ets2;vsx1;cdx2;apobec2;aplp1;znf41;rasa1;sirt3;zbtb33;phka2;kpna2;smad6;akt2;myog;runx1t1;uchl5;prdm14;snrpe;mex3b;cstf1;mki67ip;usp39;zxdc;wdr36;mrpl44;usp32;rbm23;abl2;dcp2;msx1;rdbp;zbtb25;dyrk1b;dffa;usp15;fbxl8;bace2;zcrb1;map4k2;eif4g2;nr4a1;supt4h1;alx1;sirt1;gin1;hectd2;rbl2;twist1;usp13;bhmt;h2afz;traf6;ogt;elf5;epha3;irf5;ran;sdf2;pcbp3;lats2;trib3;myd88;cdc14b;fbxl22;hdac10;st6gal1;fubp3;phkg1;zbtb45;ccnd3;zbtb32;mmp9;tmub2;ints5;ppm1d;bcl6;senp2;gsc;usf1;atf7;stat3;sp1;camk4;maff;socs1;brms1l;znf501;b3galt1;snrk;cbfa2t2;clk3;ddb1;junb;mlxipl;fbxo45;foxc1;ripk2;hoxd8;xrcc2;ezh2;znf346;tprkb;meis3;bclaf1;tsfm;yap1;ihh;igf2bp3;cux1;zbtb16;hoxc9;znf768;polh;irx3;sfpq;mtf1;slbp;znf581;cln5;prrx1;srpk1;cdk6;fance;hcls1;bub1b;creb3;trmu;rnf2;sphar;senp8;pygl;hunk;ssrp1;utp6;eya3;pogk;prkag2;asb2;rapgef4;ikzf3;camk2d;cdca7;ripply1;zscan21;gpaa1;hmgb4;sh3rf2;mkl1;adprh;tox3;syncrip;taok2;esrrg;znf446;rcor2;rfx5;tlk1;pim1;tox;ddr2;zdhhc7;cited2;galnt6;ulk3;adat1;zbtb20;fen1;ccnb1ip1;ttll5;dek;duxa;man1a1;atg7;rabggta;ppp4r2;zfx;tbx10;med8;sp2;plod1;nlk;galnt9;pbrm1;syvn1;znfx1;gnptg;ppp1cb;pam;znf496;prkci;pak1;tp53rk;map3k2;tgfb1;mapk1;dusp22;trip13;elk4;ppm1l;cdyl2;cdkl2;gucy2c;phtf1;ncoa2;drgx;adra1b;ppp1cc;mgat4a;tceal8;rnf41;egln2;mixl1;tox4;eya4;hus1;dhx15;ctnnb1;elavl2;polr2f;znf354a;rax;znf451;grsf1;rpp14;tspyl1;flt1;hdac9;usp2;mapkapk5;rnps1;utp3;hoxb3;adat3;ppargc1b;taok3;il17a;nedd4l;csnk1a1;gpx4;pias1;set;klf5;rab8a;cdk10;cercam;tsc22d4;lrrc29;mysm1;tead1;nap1l5;pigs;sim2;elf1;egr2;rad23a;rad54l;gabpb2;dhx38;hectd3;pcgf1;a4gnt;aoah;srms;arid5a;atg4a;onecut2;scap;fbxo47;stat1;creg1;atg12;psmc5;c1qtnf2;dlx3;runx1;alkbh1;sycp3;foxp4;ptk2;ppil3;sap30bp;rbm3;ears2;znf558;spcs2;dmc1;mark2;dusp10;znf33b;prkra;nr4a2;znf548;tfip11;map3k7;map3k4;cdk2ap1;pparg;cdc16;sumo1;znf414;lcor;glyctk;vps72;nr5a1;fbxl17;cdc42bpb;b3gnt4;sqstm1;traf7;neurod6;fancf;gmeb2;ptpn18;chd3;pabpc4;prmt2;fubp1;csnk1g3;chaf1a;rrp9;ikzf4;ern1;znf664;keap1;med20;nfatc2;zfp2;cnbp;mtmr7;npr2;polr3c;dlx2;hnrpll;hmgn2;foxj3;mars2;fbxl4;zfp42;ccnt2;atf3;pou3f2;rbm14;znf214;bcl11b;wars2;sox9;utf1;pou4f3;ppm1k;rab10;rab37;aurkc;vgll4;pcsk7;tgm7;pou2f1;polr2d;clock;znf605;prkaa2;pja2;gsk3b;gtf2e1;hey2;ddx54;elk1;znf546;cnot6;psmd14;ero1lb;med23;lrrk1;vax1;klf12;znf197;uhrf2;hoxa3;ubqln4;b3galt5;wars;nhp2l1;prkca;cpa4;senp3;mettl1;adam10;b3gnt2;tlx3;sugt1;znf175;ftsj2;grip1;zfp1;taf4;cops2;usp11;znf710;wnt2;dlx5;ube2m;gaa;oxsr1;araf;papd5;dus1l;tfe3;fbxo9;rpp38;med18;fbxl13;slc30a9;il20;st3gal1;itk;sf3b2;ppie;ing3;whsc2;tinf2;nr5a2;taf6;rnf14;six6;cmas;fbxw8;malt1;eif2ak2;prkaa1;zscan29;abi2;map4k3;hdx;hars;znf771;sp5;hells;zbtb12;hoxc12;klf15;scml1;rbm17;ero1l;chst1;agk;aurkb;rarb;rrm2;pja1;plagl2;ovol2;pax8;runx3;brf2;tpst1;lcorl;habp4;meox2;upf3a;asz1;foxn1;map3k9;znf524;neil1;supt16h;sox15;klf1;znf787;dcp1a;pepd;nr0b2;nfatc2ip;znf786;pdk2;vrk1;scmh1;znf623;sars;znf688;mttp;b3galt2;ptpn21;tnp1;tgm5;tnks;senp1;herc6;aipl1;crnkl1;pigu;gja1;prdm12;eef1d;cdkn2d;trim24;gabpa;rbck1;apobec1;uimc1;sox6;pcna;rbm5;setdb2;sirt5;hnrnpl;fut3;pknox1;skiv2l2;nanog;imp4;axl;rad51;gcnt4;mid1;map2k7;lcmt1;acvr2b;klhl15;mark1;dusp13;fip1l1;tfap2c;fbxo38;ilf3;prpf6;adrm1;opn1sw;hif3a;clp1;znf24;snip1;stk39;clcf1;cnot4;pnn;rragc;nek8;ubtd2;anapc7;tnfaip3;twf1;asphd1;abt1;lrrc41;grhl2;rcc1;b3gnt5;camk2g;scml2;ilf2;btk;ctcf;tcea3;exosc7;psma8;rpp30;pole3;ncln;zmat5;farsa;pcgf2;npas1;rplp1;ftsj1;dedd;tnp2;tbpl2;isg20;pak3;psmb5;cdc40;rnpc3;alg3;krba2;crebzf;trub1;usp33;smc5;st3gal4;cog2;znf689;crem;casz1;terf2;ttll9;sirt4;npm2;znf131;pigb;sox5;prdm4;wee2;elf3;znf398;trub2;crx;hnrnpr;dbx2;atf2;gan;znf454;nr6a1;prtn3;epha1;snd1;med24;dmpk;gtf2a1;pskh1;gzf1;ubqln2;jak1;tspyl2;rfx3;nab1;nsun2;brsk1;cstf2t;nuak1;epc2;trove2;zbtb38;mecp2;nars;cdt1;dot1l;scnm1;st8sia5;rnaseh1;adad1;rad1;fbxl6 |
| negative regulation of cellular metabolic process; | 62#381 | 6.11181E-13 | sirt4;edn1;hus1;sim2;ctnnb1;eid2;cdkn2d;grm8;uimc1;sox15;klf12;serpina5;bclaf1;ripply1;sirt5;nr6a1;ctcf;cux1;zbtb16;arid5a;sumo1;lcor;scap;klf11;gzf1;tgfb1;msx1;tspyl2;zbtb32;vps72;prkca;prkaa1;lancl2;nr0b2;bcl6;npas1;cda;sox9;nab1;rfx3;ghsr;hells;dedd;scmh1;set;pdcd4;stat3;ncoa2;pou2f1;ilf3;adra1b;supt4h1;glis2;crebzf;mecp2;sirt1;cdt1;tnp1;socs1;znf24;sirt3;twist1 |
| negative regulation of nucleobase, nucleoside, nucleotide and nucleic acid metabolic process; | 54#319 | 2.96918E-12 | sirt4;edn1;hus1;sim2;ctnnb1;eid2;grm8;uimc1;sox15;klf12;bclaf1;ripply1;sirt5;nr6a1;ctcf;cux1;zbtb16;arid5a;sumo1;lcor;klf11;gzf1;tgfb1;msx1;tspyl2;vps72;zbtb32;lancl2;nr0b2;bcl6;npas1;cda;sox9;nab1;rfx3;hells;dedd;scmh1;set;pdcd4;stat3;ncoa2;pou2f1;ilf3;supt4h1;glis2;crebzf;mecp2;sirt1;tnp1;cdt1;znf24;sirt3;twist1 |
| positive regulation of cellular metabolic process; | 59#365 | 4.23761E-12 | cd28;elk1;aurkaip1;foxc1;myog;ctnnb1;elf1;ran;azu1;il20;nfatc2;nr4a2;sox15;crx;myd88;yap1;ihh;ilf2;dlx2;ctcf;rnf14;scap;ccnd3;tgfb1;prkca;rbm14;prkaa1;esrrg;rad51;dyrk1b;ppargc1b;il3;sox9;utf1;sqstm1;ghsr;c1qtnf2;psmc5;pias1;samd4a;klf5;klf15;grip1;sp1;ncoa2;ilf3;zbtb38;hcls1;adra1b;supt4h1;glis2;runx1;cited2;prpf6;plagl2;clock;clcf1;pax8;ift88 |
| regulation of cell cycle; | 58#359 | 7.08454E-12 | cdc73;pnn;cd28;aurkaip1;npm2;shc1;foxc1;hus1;ccng1;ern1;cdkn2d;bub3;cdkn3;tgfb2;cgref1;sfn;lats2;rbm5;hectd3;uhrf2;rcc1;madd;sesn1;cables1;nbl1;cdk2ap1;mad2l2;ctcf;cdc16;ing1;dcc;nusap1;ccnt2;ccnd3;tgfb1;tspyl2;cdc37;rassf5;gas2;ppp1r15a;bcl6;brsk1;eif4g2;pdcd4;tp53bp2;plekho1;bub1b;cited2;rhob;cdt1;cdk10;dph1;rad1;rbl2;trim13;ddb1;runx3;diras3 |
| RNA metabolic process; | 377#4155 | 1.14644E-11 | foxp4;exosc10;ikzf1;ppil3;sap30bp;rbm3;hoxd1;ears2;tshz3;znf558;znf566;znf33b;znf548;nr4a2;tfip11;pparg;znf414;lcor;klf11;cdk9;sf3a2;vps72;nr5a1;hif1an;sqstm1;traf7;neurod6;gmeb2;pop5;chd3;glis2;ets2;pabpc4;vsx1;cdx2;fubp1;apobec2;aplp1;znf41;rasa1;sirt3;zbtb33;rrp9;smad6;chaf1a;ikzf4;myog;znf664;ern1;keap1;runx1t1;med20;nfatc2;prdm14;zfp2;cnbp;snrpe;cstf1;mki67ip;usp39;zxdc;wdr36;hnrpll;polr3c;dlx2;mrpl44;hmgn2;foxj3;rbm23;mars2;zfp42;ccnt2;atf3;dcp2;msx1;pou3f2;rbm14;rdbp;zbtb25;dyrk1b;bcl11b;znf214;sox9;wars2;utf1;pou4f3;zcrb1;eif4g2;rab10;rab37;nr4a1;vgll4;supt4h1;pou2f1;alx1;polr2d;sirt1;clock;znf605;rbl2;twist1;gtf2e1;hey2;ddx54;elk1;znf546;cnot6;elf5;irf5;ran;med23;pcbp3;vax1;znf197;trib3;klf12;myd88;hdac10;hoxa3;fubp3;zbtb45;wars;zbtb32;nhp2l1;ints5;bcl6;gsc;mettl1;tlx3;znf175;usf1;ftsj2;atf7;stat3;sp1;grip1;zfp1;taf4;cops2;maff;brms1l;znf501;znf710;cbfa2t2;dlx5;junb;mlxipl;foxc1;dus1l;tfe3;rpp38;hoxd8;slc30a9;med18;ezh2;znf346;meis3;sf3b2;ppie;ing3;bclaf1;yap1;whsc2;ihh;nr5a2;taf6;igf2bp3;cux1;zbtb16;rnf14;hoxc9;znf768;six6;zscan29;irx3;sfpq;mtf1;eif2ak2;slbp;znf581;hdx;hars;znf771;sp5;hells;prrx1;zbtb12;srpk1;hoxc12;klf15;hcls1;scml1;rbm17;creb3;trmu;rarb;plagl2;rnf2;ovol2;runx3;pax8;brf2;habp4;lcorl;meox2;upf3a;ssrp1;utp6;eya3;pogk;foxn1;znf524;ikzf3;cdca7;supt16h;sox15;ripply1;zscan21;klf1;hmgb4;mkl1;znf787;tox3;dcp1a;syncrip;esrrg;nr0b2;znf446;rcor2;rfx5;nfatc2ip;znf786;scmh1;tox;znf623;sars;znf688;cited2;zbtb20;tnp1;adat1;crnkl1;dek;duxa;prdm12;ppp4r2;trim24;gabpa;zfx;apobec1;tbx10;med8;uimc1;sox6;sp2;rbm5;nlk;sirt5;pbrm1;hnrnpl;znfx1;pknox1;znf496;skiv2l2;nanog;imp4;tgfb1;trip13;elk4;phtf1;fip1l1;tfap2c;ncoa2;drgx;ilf3;prpf6;adrm1;hif3a;clp1;znf24;tceal8;snip1;cnot4;pnn;rragc;tox4;mixl1;eya4;dhx15;ctnnb1;elavl2;polr2f;znf354a;rax;abt1;znf451;grsf1;grhl2;rpp14;scml2;hdac9;ilf2;ctcf;tcea3;exosc7;rnps1;rpp30;utp3;hoxb3;adat3;ppargc1b;zmat5;farsa;pcgf2;npas1;ftsj1;dedd;pias1;isg20;tbpl2;klf5;cdc40;rab8a;rnpc3;krba2;crebzf;trub1;tsc22d4;znf689;crem;casz1;mysm1;sirt4;tead1;znf131;sim2;elf1;egr2;sox5;prdm4;gabpb2;elf3;znf398;trub2;dhx38;crx;pcgf1;hnrnpr;dbx2;atf2;znf454;nr6a1;snd1;med24;gtf2a1;arid5a;onecut2;scap;gzf1;tspyl2;creg1;stat1;nsun2;rfx3;nab1;cstf2t;psmc5;epc2;trove2;zbtb38;runx1;dlx3;mecp2;nars;scnm1;rnaseh1;adad1 |
| negative regulation of metabolic process; | 66#436 | 1.25481E-11 | trim54;hus1;ctnnb1;grm8;capza2;sox15;klf12;serpina5;capza3;ripply1;ctcf;sumo1;lcor;klf11;zbtb32;prkca;vps72;lancl2;nr0b2;bcl6;npas1;dedd;scmh1;set;stat3;glis2;crebzf;capg;tnp1;socs1;sirt3;sirt4;edn1;sim2;eid2;cdkn2d;uimc1;bclaf1;nr6a1;sirt5;cux1;zbtb16;arid5a;scap;gzf1;msx1;tgfb1;tspyl2;prkaa1;cda;sox9;rfx3;nab1;ghsr;hells;pdcd4;ncoa2;supt4h1;adra1b;ilf3;pou2f1;mecp2;cdt1;sirt1;znf24;twist1 |
| regulation of transcription from RNA polymerase II promoter; | 64#423 | 3.01799E-11 | dek;junb;elk1;foxc1;myog;sim2;ctnnb1;elf1;foxn1;gabpa;med20;med23;znf354a;gabpb2;tbx10;ikzf3;sp2;nr4a2;sox15;crx;klf12;myd88;nr6a1;yap1;ihh;pparg;dlx2;ctcf;pknox1;cux1;rnf14;lcor;klf11;gzf1;msx1;zbtb32;vps72;pou3f2;rbm14;mtf1;nr5a1;nr0b2;ppargc1b;creg1;bcl6;npas1;sox9;utf1;sqstm1;pou4f3;tfap2c;stat3;sp1;ncoa2;chd3;supt4h1;zbtb38;runx1;cited2;prpf6;mecp2;plagl2;clock;twist1 |
| response to stress; | 140#1222 | 3.06488E-11 | tpst1;cd14;ssrp1;cfb;actn4;neil1;dusp10;prkra;tmod1;sox15;supt16h;map3k4;cx3cl1;hpse;c1qc;sumo1;gab1;procr;taok2;hsph1;tlk1;sqstm1;plod2;fancf;dcbld2;rrm2b;cited2;pabpc4;vapb;tnp1;fen1;gp6;gna13;cxcl14;gp1ba;abcf1;cfi;chaf1a;edn1;gja1;il1rap;ern1;cdkn2d;thbd;pcna;plod1;sesn1;ltb4r;syvn1;sord;tgfb1;rbm14;mapk1;tlr2;rad51;ppp1r15a;map2k7;ghsr;map4k2;mrps35;tgm7;stk39;f7;gsk3b;egln2;hus1;pdia2;ccr7;cgref1;kng1;ucn2;trib3;hyou1;myd88;scnn1b;hdac9;plaur;krt20;hsp90aa1;mapkapk5;serpinc1;hspb1;prkca;tfpi2;apoh;cxcl11;bcl6;slc6a2;il17a;gpx4;gna12;eef1e1;ccr2;stat3;il13;adm;c4bpb;maff;hspd1;ddb1;oxsr1;chrna4;vegfa;entpd1;gpr68;ripk2;lyz;rad23a;tgfb2;xrcc2;azu1;rad54l;sfn;hspa1l;il20;elf3;errfi1;aoah;hspa4;mrps9;cd93;ngfr;krt1;prdx5;olr1;scap;lman1;polh;sfpq;eif2ak2;prkaa1;alb;map4k3;brsk1;f3;epc2;fance;alkbh1;chst1;rad1 |
| blood circulation; | 33#160 | 4.52033E-11 | gaa;soat1;meox2;foxc1;edn1;gja1;kcnj12;asz1;tgfb2;azu1;kng1;brs3;uts2;npr2;eln;dmpk;tpm1;npr3;olr1;des;atp2a2;prkca;kcne1;kcnq1;chrm2;nts;kcnmb4;adra1b;hcn4;gch1;adm;wnt2;agtr1 |
| circulatory system process; | 33#160 | 4.52033E-11 | gaa;soat1;meox2;foxc1;edn1;gja1;kcnj12;asz1;tgfb2;azu1;kng1;brs3;uts2;npr2;eln;dmpk;tpm1;npr3;olr1;des;atp2a2;prkca;kcne1;kcnq1;chrm2;nts;kcnmb4;adra1b;hcn4;gch1;adm;wnt2;agtr1 |
| sensory perception of light stimulus; | 41#223 | 4.65034E-11 | chml;rom1;eya4;crybb3;rabggta;eya3;myo3a;grm8;crygd;rax;tyr;cnga3;crybb2;crx;kcnv2;wdr36;cacna1f;pde6d;rd3;six6;rpgr;efemp1;sord;ablim1;cln5;bbs9;pou4f3;gprc5d;cds1;best1;cabp4;tgfbi;opn1sw;kifc3;iqcb1;vsx1;pdc;sema4a;rs1;timp3;aipl1 |
| visual perception; | 41#223 | 4.65034E-11 | chml;rom1;eya4;crybb3;rabggta;eya3;myo3a;grm8;crygd;rax;tyr;cnga3;crybb2;crx;kcnv2;wdr36;cacna1f;pde6d;rd3;six6;rpgr;efemp1;sord;ablim1;cln5;bbs9;pou4f3;gprc5d;cds1;best1;cabp4;tgfbi;opn1sw;kifc3;iqcb1;vsx1;pdc;sema4a;rs1;timp3;aipl1 |
| response to external stimulus; | 85#633 | 4.65034E-11 | tpst1;ccr8;cd14;cxcl16;ogt;cfb;ccr7;kng1;brs3;sox15;myd88;lep;cx3cl1;scnn1b;plaur;hdac9;pparg;hpse;c1qc;pglyrp3;serpinc1;procr;prkca;tfpi2;apoh;cxcl11;bcl6;il17a;gna12;gpx4;ccl28;dcbld2;ccr2;stat3;il13;pabpc4;adm;c4bpb;gp6;gna13;cxcl14;pdc;gp1ba;ccrl1;abcf1;cfi;vegfa;entpd1;gpr68;shroom2;gja1;cdh13;ripk2;il1rap;lyz;cdkn2d;egr2;tgfb2;azu1;thbd;il20;elf3;fech;aoah;enpp2;cd93;prdx5;krt1;ltb4r;ngfr;cacna1f;olr1;sord;lman1;tgfb1;tlr2;mapk1;alb;ghsr;f3;creb3;opn1sw;chst1;f7;ccr9 |
| cell morphogenesis; | 69#478 | 7.51881E-11 | nck1;egln2;rragc;prickle2;shc1;ctnnb1;dscaml1;fxyd5;alox12;mark2;camk2d;akap4;sema4d;fhl1;fgd1;dcc;nrcam;fez1;net1;taok2;cdc42bpb;gas2;bcl6;cdkn2aip;il3;cyfip1;cdc42ep5;b3gnt2;dcbld2;klf5;slitrk4;htra1;capg;socs1;brms1l;gna13;rasa1;gp1ba;dlx5;ebag9;nrp1;gja1;cdh13;cdc42ep4;cdkn2d;tgfb2;ing3;slitrk3;crim1;ngfr;cacna1f;ing1;igfbp6;prkci;ctnna1;tgfb1;pou3f2;tspyl2;creg1;cda;wisp1;pou4f3;adra1b;efnb3;esm1;fgf20;cck;thbs4;nrg3 |
| cellular structure morphogenesis; | 69#478 | 7.51881E-11 | nck1;egln2;rragc;prickle2;shc1;ctnnb1;dscaml1;fxyd5;alox12;mark2;camk2d;akap4;sema4d;fhl1;fgd1;dcc;nrcam;fez1;net1;taok2;cdc42bpb;gas2;bcl6;cdkn2aip;il3;cyfip1;cdc42ep5;b3gnt2;dcbld2;klf5;slitrk4;htra1;capg;socs1;brms1l;gna13;rasa1;gp1ba;dlx5;ebag9;nrp1;gja1;cdh13;cdc42ep4;cdkn2d;tgfb2;ing3;slitrk3;crim1;ngfr;cacna1f;ing1;igfbp6;prkci;ctnna1;tgfb1;pou3f2;tspyl2;creg1;cda;wisp1;pou4f3;adra1b;efnb3;esm1;fgf20;cck;thbs4;nrg3 |
| macromolecule metabolic process; | 866#11144 | 1.3369E-10 | pigq;ttbk1;exosc10;fkbp3;ikzf1;cfb;fbxo10;usp30;hoxd1;tshz3;myo3a;nrbp1;nsf;psmc2;znf566;msrb3;fut1;sucla2;st13;klf11;cdk9;tspan8;chst14;sf3a2;tec;hif1an;il3;plod2;adam20;arih1;st3gal3;rrm2b;st6galnac4;phkg2;pop5;rbbp6;glis2;pstk;rnf139;ets2;vsx1;cdx2;lalba;apobec2;aplp1;hgsnat;znf41;rasa1;sirt3;eif3f;zbtb33;phka2;kpna2;smad6;akt2;myog;runx1t1;xpo6;uchl5;prdm14;snrpe;mex3b;cstf1;calr3;mki67ip;usp39;zxdc;rpl8;wdr36;mrpl44;usp32;rbm23;abl2;dcp2;msx1;adam12;rdbp;zbtb25;dyrk1b;dffa;usp15;fbxl8;bace2;zcrb1;map4k2;eif4g2;nr4a1;supt4h1;klk7;alx1;sirt1;gin1;ldhb;hectd2;mrpl3;f7;rbl2;twist1;usp13;bhmt;h2afz;traf6;ogt;elf5;epha3;irf5;ran;sdf2;pcbp3;lats2;tbcc;trib3;myd88;cdc14b;fbxl22;khk;hdac10;st6gal1;hsp90aa1;fubp3;phkg1;zbtb45;ccnd3;fuca2;leprel2;zbtb32;l2hgdh;mmp9;tmub2;ints5;dlst;ppm1d;bcl6;senp2;gsc;usf1;adam21;atf7;grpel2;stat3;sp1;camk4;gnpda1;pfkfb2;cpb1;c4bpb;maff;socs1;brms1l;znf501;b3galt1;snrk;cbfa2t2;clk3;ddb1;junb;mlxipl;fbxo45;foxc1;ripk2;pgam2;hoxd8;xrcc2;azu1;ezh2;znf346;tprkb;meis3;ctsw;bclaf1;kpna3;ppic;tsfm;yap1;mrps9;ihh;me2;igf2bp3;cux1;zbtb16;hoxc9;znf768;polh;irx3;sfpq;mtf1;slbp;znf581;cln5;mmp12;prrx1;srpk1;cdk6;skap2;fance;trpc5;hcls1;bub1b;creb3;gns;trmu;rnf2;sphar;hyal2;senp8;dph1;pygl;ift88;klk13;hunk;ssrp1;utp6;mrps18c;prepl;mrpl30;eya3;pogk;slc2a2;cpa5;prkag2;asb2;rapgef4;brs3;ikzf3;camk2d;cdca7;apeh;capza3;ripply1;zscan21;gpaa1;g6pd;sh3rf2;hmgb4;mkl1;adprh;tktl1;tox3;syncrip;taok2;esrrg;znf446;rcor2;galns;rfx5;cdkn2aip;tlk1;pim1;tox;samd4a;ddr2;zdhhc7;cited2;ap3d1;galnt6;hibadh;ulk3;adat1;zbtb20;fen1;qpctl;ccnb1ip1;tff1;ttll5;dek;duxa;man1a1;atg7;rabggta;ppp4r2;zfx;ap3s1;tbx10;mrpl22;med8;traf1;sp2;stbd1;plod1;nlk;galnt9;pbrm1;tor2a;syvn1;znfx1;gnptg;pam;ppp1cb;eif1ax;znf496;ogfod1;prkci;pak1;tp53rk;ctnna1;map3k2;tgfb1;mapk1;dusp22;trip13;ace2;slmap;elk4;ppm1l;ghsr;cdyl2;rps29;cdkl2;gucy2c;phtf1;klk9;ncoa2;drgx;adra1b;tubg1;cct6b;ppp1cc;mgat4a;tceal8;ogdh;eif5;uap1;rnf41;egln2;mixl1;tox4;eya4;hus1;dhx15;ctnnb1;elavl2;tysnd1;matn1;polr2f;scrn1;znf354a;capza2;rax;znf451;grsf1;mrpl16;rpp14;lep;bpgm;tspyl1;plaur;flt1;hdac9;usp2;cpa6;mapkapk5;rnps1;pglyrp3;utp3;hoxb3;scarf1;adat3;ppargc1b;taok3;rps10;il17a;nedd4l;csnk1a1;gpx4;mrpl37;oma1;eef1e1;pias1;set;klf5;ranbp9;rab8a;tnip1;htra1;cdk10;hspd1;cercam;tsc22d4;lrrc29;mysm1;tead1;spaca3;nap1l5;pigs;sim2;elf1;egr2;gfpt1;rad23a;mrpl47;rad54l;gabpb2;dhx38;hectd3;pcgf1;pgd;a4gnt;aoah;p4ha1;krt1;eif3e;olr1;srms;arid5a;atg4a;onecut2;scap;ttc1;fbxo47;atg12;stat1;creg1;psmc5;c1qtnf2;napg;dlx3;runx1;ldhc;alkbh1;sycp3;impa1;capn2;mrpl35;foxp4;ptk2;ppil3;has3;sap30bp;rbm3;ears2;spcs2;znf558;dmc1;mark2;dusp10;znf33b;znf548;prkra;nr4a2;tfip11;map3k7;map3k4;fkbp6;cdk2ap1;pparg;ctss;c1qc;cdc16;sumo1;znf414;lcor;glyctk;ctsa;vps72;nr5a1;fbxl17;rce1;cdc42bpb;b3gnt4;sqstm1;traf7;cdc42ep5;neurod6;fancf;gmeb2;cldn14;ptpn18;chd3;pabpc4;prmt2;capg;fubp1;klk12;csnk1g3;abcf1;cpa3;rrp9;chaf1a;ikzf4;znf664;ern1;keap1;med20;nfatc2;zfp2;cnbp;eif1b;mtmr7;npr2;polr3c;dlx2;hnrpll;cpxm1;hmgn2;ptpla;foxj3;mars2;fbxl4;zfp42;ccnt2;sord;atf3;pou3f2;rbm14;bcl11b;znf214;wars2;sox9;utf1;pou4f3;ppm1k;rab10;rab37;ganab;aurkc;ugdh;vgll4;tgm7;pcsk7;pou2f1;dpp8;lonp1;polr2d;mrps18b;gfpt2;clock;znf605;prkaa2;pja2;gsk3b;gtf2e1;hey2;nck1;trim54;neu3;ddx54;sdha;elk1;znf546;cnot6;psmd14;ero1lb;mrpl10;med23;aldh5a1;lrrk1;vax1;znf197;klf12;serpina5;uhrf2;dnajc17;hyal3;hoxa3;ubqln4;b3galt5;wars;slc25a10;nhp2l1;prkca;cpa4;senp3;mettl1;adam10;ap1s3;tlx3;b3gnt2;znf175;sugt1;ftsj2;tinag;ap1g1;grip1;zfp1;tmem48;nubp1;taf4;cops2;usp11;znf710;wnt2;ube2m;dlx5;oxsr1;gaa;mrpl36;araf;slc7a7;papd5;dus1l;eid2;tfe3;fbxo9;rpp38;med18;fbxl13;slc30a9;il20;lrpap1;st3gal1;itk;lonrf1;sf3b2;rps8;ppie;ing3;whsc2;tinf2;nr5a2;taf6;rnf14;lrrc14;six6;cmas;fbxw8;malt1;zscan29;prkaa1;eif2ak2;abi2;map4k3;hdx;picalm;hars;znf771;sp5;hells;zbtb12;hoxc12;klf15;pum2;scml1;rbm17;ero1l;chst1;agk;aurkb;rarb;rrm2;pja1;plagl2;hexa;ovol2;runx3;pax8;brf2;tpst1;habp4;lcorl;meox2;upf3a;ict1;asz1;foxn1;neil1;znf524;map3k9;supt16h;sox15;chst8;klf1;znf787;hpse;dcp1a;pepd;nr0b2;nfatc2ip;casp7;znf786;ldha;vrk1;pdk2;scmh1;znf623;sars;znf688;mttp;b3galt2;ptpn21;tgm5;tnp1;tnks;senp1;herc6;eif4h;aipl1;crnkl1;pigu;cfi;gja1;prdm12;eef1d;copg2;il1rap;cdkn2d;trim24;gabpa;mrpl27;rbck1;apobec1;uimc1;chst12;sox6;pcna;rbm5;setdb2;crim1;sirt5;hnrnpl;fut3;pknox1;skiv2l2;nanog;imp4;axl;adam32;rad51;gcnt4;mid1;pld2;map2k7;lcmt1;acvr2b;cda;klhl15;aco1;mark1;dusp13;fip1l1;tfap2c;trpv5;fbxo38;slc38a4;ilf3;prpf6;adrm1;opn1sw;hif3a;clp1;znf24;snip1;stk39;clcf1;pgk2;cnot4;pnn;ubtd2;rragc;nek8;aurkaip1;anapc7;lin7a;pdia2;idh3a;tnfaip3;gga3;twf1;asphd1;abt1;lrrc41;grhl2;rcc1;b3gnt5;camk2g;scml2;ilf2;btk;ctcf;tcea3;exosc7;psma8;ipmk;rps3;hspb1;rpp30;pole3;ncln;zmat5;npas1;farsa;pcgf2;cndp2;rplp1;ftsj1;dedd;tnp2;isg20;tbpl2;pak3;psmb5;cdc40;rnpc3;alg3;krba2;crebzf;trub1;usp33;smc5;st3gal4;cog2;znf689;crem;terf2;casz1;ttll9;sirt4;cd28;npm2;cd74;znf131;pigb;sox5;prdm4;wee2;elf3;crx;znf398;trub2;hnrnpr;dbx2;atf2;gan;znf454;nr6a1;epha1;snd1;prtn3;dmpk;med24;fbp2;rpl9;gtf2a1;tubgcp4;acly;pskh1;gzf1;ubqln2;lman1;jak1;tspyl2;tubb6;rsl1d1;nsun2;rfx3;nab1;brsk1;cstf2t;hk3;denr;nuak1;epc2;eif3i;trove2;zbtb38;pdha1;mecp2;nars;cdt1;dot1l;scnm1;st8sia5;dnaja4;rnaseh1;adad1;rad1;fbxl6 |
| nervous system development; | 92#716 | 1.39787E-10 | hey2;ctnnb1;dscaml1;asz1;pcdh18;aldh5a1;mdga1;pafah1b3;nr4a2;sema3a;chst8;b3gnt5;dctn1;sema3d;sema4d;mdga2;dcc;ipmk;nrcam;fez1;utp3;npas1;pcdhb5;il3;cyfip1;chrm2;neurod6;tlx3;b3gnt2;tbcb;fzd9;stat3;pcdhb6;rbm45;slitrk4;glis2;pcdh1;aplp1;doc2a;serpinf1;fgf5;wnt2;sema4a;plp1;dlx5;dpysl3;nrp1;chrna4;vegfa;shroom2;foxc1;gfra3;gja1;sim2;scrg1;mbp;egr2;tgfb2;azu1;slc5a3;mpped2;trim3;spock1;slitrk3;crim1;pdgfc;ngfr;cacna1f;dlx2;nde1;zbtb16;ctnna1;msx1;tgfb1;pou3f2;cln5;pou4f3;ghsr;cdk6;mobp;pcdhb11;trpc5;ndrg2;kcnq2;alx1;snai1;efnb3;rufy3;pts;drp2;cck;hexa |
| regulation of progression through cell cycle; | 55#353 | 2.14816E-10 | cdc73;pnn;cd28;aurkaip1;npm2;shc1;foxc1;hus1;ccng1;ern1;cdkn2d;bub3;cdkn3;tgfb2;cgref1;sfn;lats2;rbm5;hectd3;rcc1;sesn1;nbl1;cdk2ap1;mad2l2;ctcf;cdc16;ing1;dcc;nusap1;ccnt2;ccnd3;tgfb1;tspyl2;cdc37;rassf5;gas2;ppp1r15a;bcl6;brsk1;eif4g2;pdcd4;tp53bp2;plekho1;bub1b;cited2;rhob;cdt1;cdk10;dph1;rad1;rbl2;trim13;ddb1;runx3;diras3 |
| cell cycle; | 103#839 | 2.39328E-10 | cdc73;pnn;aurkaip1;shc1;anapc7;hus1;dmc1;cgref1;ran;dnm2;lats2;uhrf2;ndc80;rcc1;cables1;cdk2ap1;dctn1;ctcf;cdc16;dcc;ccnd3;evi5;gas2;ppm1d;bcl6;tlk1;appl1;sugt1;tp53bp2;coro1a;plekho1;cited2;rhob;cdk10;terf2;ddb1;cd28;kpna2;npm2;chaf1a;foxc1;papd5;ccng1;ern1;cdkn2d;bub3;cdkn3;tgfb2;xrcc2;rad54l;sfn;rbm5;hectd3;cntrob;madd;sesn1;nbl1;pbrm1;mad2l2;ppp1cb;nde1;ing1;akap8;ccnt2;nusap1;ncaph;tgfb1;tspyl2;mapk1;cdc37;eif2ak2;rad51;rassf5;spin1;ppp1r15a;ccar1;brsk1;eif4g2;hells;cdc27;dusp13;aurkc;cdk6;mns1;pdcd4;ilf3;stag1;bub1b;tubg1;sycp3;aurkb;cdt1;ppp1cc;pds5a;sac3d1;sgol1;dph1;rad1;ncapg;trim13;rbl2;diras3;runx3 |
| positive regulation of transcription, DNA-dependent; | 40#223 | 2.83822E-10 | elk1;foxc1;myog;ctnnb1;elf1;ran;nr4a2;sox15;crx;myd88;yap1;ihh;ilf2;dlx2;ctcf;rnf14;scap;tgfb1;rbm14;esrrg;dyrk1b;ppargc1b;sox9;utf1;sqstm1;psmc5;pias1;klf15;grip1;sp1;ncoa2;ilf3;zbtb38;supt4h1;cited2;runx1;prpf6;plagl2;clock;pax8 |
| intracellular signaling cascade; | 198#1965 | 8.04702E-10 | sectm1;rgs4;slc20a1;map3k9;asb2;grm8;mark2;rapgef4;dusp10;map3k7;map3k4;ndc80;depdc7;traf3ip2;iqsec3;grap2;vdac1;gab1;evi5;taok2;dgkz;tec;cdc42bpb;casp7;tlk1;plek;htr4;il3;sqstm1;traf7;dbnl;cdc42ep5;dcbld2;rapgef1;ddah1;arhgef6;trim38;vapb;sh2d3c;fen1;gna13;rasa4;arfgef2;rasa1;zbtb33;pigu;edn1;cdh13;gja1;eef1d;ern1;rasl11a;pcna;rgs1;atp2c1;nlk;iqgap2;npr2;arl16;arhgdib;rab3c;ltb4r;elmo1;npr3;prkci;pak1;dnm1l;map3k2;atp2a2;rbm14;mapk1;dffa;stac2;arhgdia;pld2;dusp22;gpr3;cnih2;smap1;map2k7;gpr139;pag1;ghsr;map4k2;mark1;rab10;gucy2c;cnih4;rab37;ncoa2;rab33a;adra1b;mtnr1b;gabbr1;snip1;clcf1;gnb1;gsk3b;nrg3;coro2a;diras3;adora2b;arl6;ddx54;rragc;egln2;shc1;stac3;traf6;spsb3;pdia2;galr1;rasgrp2;ctnnb1;tnfaip3;ran;lrrk1;diras1;lats2;synj2bp;arhgap1;myd88;dgka;iqsec2;btk;rem1;cckbr;rundc3a;fgd1;rgs11;net1;dgkb;prkca;plcz1;nras;ppargc1b;taok3;bcl6;arfgap1;chrm2;gnai3;nmbr;sh2b1;pias1;ccr2;grip1;stat3;rab8a;rhob;socs1;rab42;ralgps1;rasal2;agtr1;mchr1;snx14;pth2r;oxsr1;ltbr;araf;ripk2;stac;lgals9;azu1;tbc1d14;sfn;il20;mcl1;vav3;itk;madd;med24;dmpk;bik;rnf14;cnksr1;mapk8ip1;arhgef9;scap;malt1;jak1;prkaa1;eif2ak2;rassf5;stat1;map4k3;gnal;psd4;c1qtnf2;srpk1;rab9b;hcls1;bub1b;arl4c;arl9;agk;eda2r;trim13 |
| cell communication; | 470#5560 | 1.10328E-09 | snx15;ptk2;rgs4;rsu1;pde1a;cops3;qprt;mark2;dusp10;nr4a2;prkra;dkk2;uts2;map3k7;map3k4;cxcr7;ndc80;traf3ip2;pparg;iqsec3;pde11a;grap2;arhgap9;gab1;gabrb2;nr5a1;tnfrsf25;rgs17;tec;cdc42bpb;plek;angptl5;il3;ramp1;sqstm1;traf7;cdc42ep5;axin2;pak1ip1;sparc;adra2b;cds1;ddah1;clec1a;prmt2;pcdh1;gpr82;lalba;gna13;rasa4;cxcl14;csnk1g3;rasa1;plp1;gp1ba;zbtb33;gpr124;nrp1;dpysl3;cd70;strn4;smad6;edn1;traf3;cdh13;gfra3;sfrp2;ern1;rasl11a;cd2;iqgap2;rspo1;cd53;npr2;arl16;elmo1;akap8;abl2;dnm1l;tshb;rbm14;tlr2;dffa;arhgdia;cnih2;smap1;snx27;pag1;impa2;asah2;map4k2;lrp6;rab10;rab37;nr4a1;kcnmb4;kremen1;rab33a;pkd2l1;sncb;kcnq2;rgs21;cck;arhgap21;clock;gabbr1;gnb1;prkaa2;gsk3b;nrg3;diras3;coro2a;adora2b;hey2;xpr1;nck1;trim54;ccr5;ddx54;shc1;gfra2;traf6;fibcd1;ogt;alcam;galr1;rasgrp2;sucnr1;ms4a7;epha3;ran;aldh5a1;lrrk1;diras1;lats2;srd5a1;myd88;tmem115;fgf1;iqsec2;tagap;akap4;gpr142;nfkbib;asgr2;hsp90aa1;jmjd6;pygo2;fgd1;rgs11;net1;prkca;dgkb;plcz1;nras;pram1;fgf6;snph;senp2;bcl6;slc6a2;arfgap1;npffr2;adam10;chrm2;angpt4;kiss1r;gnai3;gnb2;sh2b1;stat3;pcdhb6;grip1;camk4;wnt5b;scn1b;adm;gpr55;cops2;socs1;wnt11;doc2a;pdc;wnt2;oxgr1;sntg1;ccrl1;pth2r;gnat3;oxsr1;grid2;chrna4;araf;vegfa;gpr68;foxc1;ripk2;eid2;mbp;stac;htr1a;tgfb2;tbc1d14;azu1;il20;pnoc;nxph4;itk;madd;ppic;ihh;ngfr;slc8a2;rnf14;igfbp6;arhgef9;ppp2r2b;malt1;nle1;prkaa1;eif2ak2;rassf5;ostf1;map4k3;cysltr2;snx22;optn;psd4;srpk1;skap2;hcls1;bub1b;arl4c;fgf20;agk;rarb;inpp1;hexa;ccr9;trim13;ift88;sectm1;arhgap15;hunk;cd14;srgap3;kcnk2;asz1;slc20a1;map3k9;asb2;grm8;pitpnc1;rapgef4;drd3;brs3;dnm2;depdc7;cx3cl1;sergef;csnk2b;vdac1;evi5;taok2;dgkz;pde1c;cdkn2aip;casp7;tlk1;pcdhb5;htr4;sh3pxd2a;appl1;dbnl;dcbld2;rapgef1;chrna5;tp53bp2;arhgef6;ddr2;cited2;trim38;vapb;sh2d3c;ocrl;hdgf;gpr84;itgb1bp2;fen1;gp6;fgf5;cd274;arfgef2;zcchc2;dek;pigu;maoa;gper;mpp1;gja1;eef1d;il1rap;ap3s1;traf1;birc3;pcna;rgs1;tacr3;atp2c1;nlk;enpp2;arhgdib;rab3c;gpr128;ltb4r;npr3;pygo1;prkci;pak1;map3k2;ctnna1;atp2a2;axl;tgfb1;nxph3;mapk1;stac2;trpv1;pld2;dusp22;gpr3;map2k7;gpr139;acvr2b;cda;ghsr;gpr174;mark1;cdkl2;gucy2c;cnih4;tfap2c;gprc5d;pcdhb11;ncoa2;adra1b;ppp2r5b;mtnr1b;opn1sw;efnb3;hif3a;snip1;clcf1;timp3;ccr8;arl6;egln2;rragc;stac3;spsb3;lin7a;pdia2;ctnnb1;ccr7;tnfaip3;synj2bp;snx25;arhgap1;efna2;lep;rgs22;dgka;camk2g;plaur;flt1;btk;rem1;ppp1r1b;cckbr;rundc3a;spry1;mapkapk5;mfap4;gpr19;trhr;ppargc1b;cxcl11;taok3;npas1;il17a;csnk1a1;gna12;pmch;nts;nmbr;grb10;pias1;sh3bp1;ccr2;areg;fzd9;rab8a;il13;cabp4;htra1;rhob;cep57;rab42;ralgps1;rasal2;crem;mchr1;agtr1;snx14;ltbr;chrne;gpr88;cd28;entpd1;cd74;fzd5;sim2;ppp1r12b;elf1;rassf8;lgals9;egr2;prdm4;fstl3;sfn;th;mkln1;cnga3;mcl1;vav3;snx5;kitlg;epha1;igsf6;dmpk;cxxc4;med24;bik;sarm1;mapk8ip1;cnksr1;scap;ptges;jak1;tspyl2;cacnb3;igsf1;alb;wif1;stat1;mln;pxk;wisp1;gnal;c1qtnf2;arhgap28;cd164;rab9b;gch1;nxph2;arl9;slc6a5;impa1;dbh;eda2r |
| negative regulation of transcription; | 47#291 | 1.25165E-09 | sirt4;sim2;ctnnb1;eid2;uimc1;sox15;klf12;bclaf1;ripply1;sirt5;nr6a1;ctcf;cux1;zbtb16;arid5a;sumo1;lcor;klf11;gzf1;msx1;tgfb1;vps72;zbtb32;nr0b2;lancl2;bcl6;npas1;sox9;nab1;rfx3;hells;dedd;scmh1;pdcd4;stat3;ncoa2;ilf3;pou2f1;supt4h1;glis2;crebzf;mecp2;sirt1;tnp1;znf24;sirt3;twist1 |
| secretion; | 57#384 | 1.35895E-09 | adora2b;kif1c;aqp1;edn1;lin7a;slc22a4;copg2;nrbp1;scrn1;rapgef4;kng1;ykt6;arfip1;trappc1;uso1;clcn5;scamp2;nlrp2;scnn1b;camk2g;stx16;sergef;ca9;srp54;krt20;srp72;stxbp1;prkci;stx7;lman1;ca2;vps33a;pram1;sec61a2;snph;bace2;sec61a1;traf7;exoc3;ghsr;nedd4l;optn;tnp2;napg;sec24b;kcnmb4;scfd1;rab9b;adm;doc2a;cog2;gosr1;arfgef2;agtr1;slc22a18;cog5;ift88 |
| response to wounding; | 61#423 | 1.45909E-09 | tpst1;cd14;cfi;entpd1;gpr68;gja1;ripk2;cfb;il1rap;lyz;ccr7;tgfb2;thbd;azu1;kng1;elf3;il20;sox15;myd88;aoah;cx3cl1;scnn1b;cd93;prdx5;plaur;ngfr;ltb4r;krt1;hdac9;hpse;c1qc;olr1;serpinc1;lman1;tgfb1;procr;prkca;tlr2;tfpi2;apoh;cxcl11;bcl6;il17a;ghsr;f3;gna12;gpx4;dcbld2;ccr2;stat3;il13;pabpc4;chst1;adm;c4bpb;gp6;gna13;cxcl14;f7;gp1ba;abcf1 |
| regulation of cellular metabolic process; | 349#3933 | 1.80583E-09 | foxp4;ikzf1;sap30bp;tigd3;hoxd1;tshz3;znf558;znf566;znf33b;znf548;nr4a2;pparg;znf414;sumo1;lcor;klf11;cdk9;vps72;nr5a1;hif1an;il3;sqstm1;traf7;neurod6;gmeb2;chd3;glis2;pstk;ets2;vsx1;cdx2;fubp1;aplp1;znf41;rasa1;sirt3;eif3f;zbtb33;kpna2;smad6;chaf1a;edn1;ikzf4;myog;znf664;ern1;keap1;runx1t1;med20;nfatc2;prdm14;zfp2;cnbp;eif1b;zxdc;polr3c;dlx2;hmgn2;foxj3;zfp42;ccnt2;atf3;msx1;pou3f2;rbm14;rdbp;zbtb25;dyrk1b;bcl11b;znf214;sox9;bace2;utf1;pou4f3;eif4g2;rab10;rab37;nr4a1;vgll4;supt4h1;pou2f1;alx1;sirt1;clock;znf605;rbl2;twist1;gtf2e1;hey2;bhmt;ddx54;elk1;znf546;cnot6;elf5;irf5;ran;med23;vax1;znf197;trib3;klf12;myd88;serpina5;hdac10;hoxa3;tcf23;fubp3;zbtb45;ccnd3;zbtb32;prkca;bcl6;gsc;tlx3;znf175;usf1;atf7;stat3;sp1;grip1;zfp1;taf4;maff;sohlh2;socs1;brms1l;znf501;znf710;cbfa2t2;dlx5;junb;mlxipl;foxc1;ripk2;eid2;tfe3;hoxd8;azu1;il20;slc30a9;med18;ezh2;meis3;ppie;ing3;bclaf1;yap1;whsc2;ihh;nr5a2;taf6;igf2bp3;cux1;zbtb16;hoxc9;rnf14;znf768;six6;polh;zscan29;prkaa1;irx3;sfpq;mtf1;eif2ak2;znf581;hdx;znf771;sp5;hells;prrx1;zbtb12;srpk1;hoxc12;klf15;pum2;hcls1;scml1;creb3;rarb;plagl2;rnf2;ovol2;ift88;runx3;pax8;brf2;habp4;lcorl;meox2;ssrp1;eya3;pogk;foxn1;znf524;grm8;dnm2;ikzf3;cdca7;supt16h;sox15;ripply1;zscan21;klf1;hmgb4;mkl1;znf787;tox3;esrrg;lancl2;nr0b2;znf446;rcor2;rfx5;nfatc2ip;tlk1;znf786;scmh1;tox;znf623;znf688;samd4a;cited2;zbtb20;tnp1;eif4h;dek;duxa;prdm12;cdkn2d;trim24;gabpa;zfx;tbx10;med8;uimc1;sox6;sp2;pcna;nlk;sirt5;pbrm1;znfx1;pknox1;znf496;nanog;tgfb1;rad51;acvr2b;cda;elk4;ghsr;phtf1;tfap2c;pdcd4;ncoa2;drgx;ilf3;adra1b;prpf6;hif3a;znf24;tceal8;snip1;clcf1;eif5;cnot4;pnn;tox4;mixl1;aurkaip1;hus1;eya4;ctnnb1;elavl2;polr2f;znf354a;rax;abt1;znf451;grhl2;l3mbtl3;scml2;hdac9;ilf2;ctcf;tcea3;hspb1;hoxb3;ppargc1b;pcgf2;npas1;dedd;pias1;set;tbpl2;klf5;rab8a;krba2;crebzf;tsc22d4;znf689;crem;casz1;terf2;mysm1;cd28;sirt4;tead1;znf131;sim2;elf1;egr2;sox5;prdm4;gabpb2;elf3;znf398;crx;pcgf1;dbx2;atf2;znf454;nr6a1;snd1;med24;eif3e;gtf2a1;arid5a;onecut2;scap;gzf1;tspyl2;igsf1;creg1;stat1;rfx3;nab1;c1qtnf2;psmc5;epc2;eif3i;zbtb38;runx1;dlx3;mecp2;cdt1;eda2r |
| vesicle-mediated transport; | 79#606 | 1.80583E-09 | stx10;cd14;ankrd27;cxcl16;lin7a;actn4;ctnnb1;nrbp1;gga3;scrn1;rapgef4;dnm2;ykt6;synj2bp;uso1;ldlrap1;scamp2;dnm3;stx16;lrp10;asgr2;stx7;scarf1;pram1;necap1;snph;arfgap1;trappc6a;sqstm1;traf7;dbnl;nedd4l;ap1s3;tnp2;scfd1;ap1g1;rabep2;vps41;rhob;ap3d1;aplp1;doc2a;cog2;arfgef2;cog5;kif1c;spaca3;cdh13;copg2;stx4;ap3s1;azu1;lrpap1;trappc1;clcn5;cd93;elmo1;prkci;stxbp1;mapk8ip1;dnm1l;pacsin1;lman1;tgfb1;vps33a;pld2;exoc3;picalm;lrp6;optn;map4k2;napg;sec24b;lmbr1l;vps26a;rab9b;tfrc;ergic3;gosr1 |
| RNA processing; | 71#525 | 2.00247E-09 | exosc10;pnn;ddx54;rragc;utp6;ppil3;dhx15;rbm3;tfip11;grsf1;rpp14;exosc7;rnps1;rpp30;utp3;nhp2l1;sf3a2;syncrip;adat3;ints5;zmat5;mettl1;ftsj1;ftsj2;sars;cdc40;pop5;rnpc3;pabpc4;trub1;apobec2;adat1;aplp1;crnkl1;rrp9;dus1l;ern1;ppp4r2;rpp38;apobec1;znf346;trub2;snrpe;dhx38;rbm5;hnrnpr;cstf1;usp39;ppie;sf3b2;wdr36;hnrnpl;hnrpll;mrpl44;igf2bp3;rbm23;skiv2l2;imp4;sfpq;slbp;nsun2;zcrb1;cstf2t;fip1l1;srpk1;rbm17;prpf6;trmu;clp1;scnm1;adad1 |
| cell-cell signaling; | 82#640 | 2.32457E-09 | lin7a;galr1;ctnnb1;asz1;qprt;grm8;aldh5a1;drd3;nr4a2;srd5a1;uts2;efna2;fgf1;lep;camk2g;spry1;grap2;nras;gabrb2;nr5a1;cxcl11;fgf6;snph;slc6a2;pcdhb5;il3;adam10;il17a;pmch;adra2b;grb10;areg;chrna5;pcdhb6;il13;camk4;scn1b;pcdh1;adm;lalba;wnt11;doc2a;fgf5;cxcl14;wnt2;plp1;nrp1;cd70;chrne;maoa;chrna4;strn4;entpd1;edn1;gja1;mbp;egr2;tgfb2;th;pnoc;ihh;tshb;tgfb1;mapk1;wif1;mln;wisp1;pxk;ghsr;tfap2c;kcnmb4;pcdhb11;adra1b;sncb;gch1;kcnq2;mtnr1b;efnb3;slc6a5;fgf20;dbh;hexa |
| metabolic process; | 1084#14566 | 2.41722E-09 | ttbk1;fkbp3;ikzf1;nox1;fbxo10;tigd3;usp30;hoxd1;myo3a;psmc2;znf566;fut1;lrp10;klf11;tec;il3;naprt1;plod2;adam20;arih1;st3gal3;phkg2;rbbp6;glis2;pstk;vsx1;cdx2;lalba;apobec2;aplp1;hgsnat;rasa1;gnpat;kpna2;nat8l;edn1;akt2;myog;xpo5;mex3b;rspo1;cstf1;pld5;calr3;rpl8;wdr36;mrpl44;rbm23;abl2;atp11a;msx1;zbtb25;dyrk1b;usp15;fbxl8;impa2;map4k2;eif4g2;klk7;alx1;sirt1;gin1;hectd2;mrpl3;f7;h2afz;elf5;lysmd3;ran;tbcc;srd5a1;trib3;cdc14b;khk;nfkbib;hsp90aa1;fubp3;pdxdc1;zbtb32;l2hgdh;tmub2;ints5;ppm1d;bcl6;senp2;gsc;usf1;dhrsx;camk4;c4bpb;socs1;cbfa2t2;ddb1;mlxipl;fbxo45;xrcc2;azu1;osbpl2;ezh2;znf346;tprkb;ctsw;ppic;tsfm;yap1;ihh;igf2bp3;proca1;irx3;sfpq;slbp;aldh16a1;znf581;cln5;cdk6;skap2;fance;insig1;hcls1;creb3;gns;inpp1;ndufv1;pts;hyal2;senp8;dph1;pygl;ift88;hunk;utp6;prepl;mrpl30;ckm;eya3;cpa5;prkag2;asb2;rapgef4;ikzf3;camk2d;dnm2;tmod1;hamp;apeh;ca3;zscan21;mmab;adprh;hadha;tktl1;syncrip;esrrg;galns;cdkn2aip;pim1;samd4a;cited2;ap3d1;hibadh;galnt6;ulk3;adat1;zbtb20;qpctl;tff1;duxa;atg7;zfx;med8;traf1;sp2;stbd1;atp2c1;nlk;galnt9;syvn1;znf496;prkci;pak1;tp53rk;tgfb1;mapk1;dusp22;ace2;slmap;elk4;ghsr;rps29;gucy2c;cad;drgx;tubg1;slc23a2;ppp1cc;tceal8;aldh1a1;rnf41;egln2;mixl1;tox4;hus1;dhx15;tysnd1;matn1;polr2f;znf354a;capza2;znf451;grsf1;mrpl16;fa2h;sc5dl;lep;bpgm;tspyl1;plaur;tgds;usp2;pnpla3;pglyrp3;scarf1;ppargc1b;taok3;mrpl37;oma1;neu1;pias1;set;klf5;rab8a;tnip1;htra1;hspd1;lrrc29;mysm1;tead1;nap1l5;sim2;egr2;gfpt1;mrpl47;rad54l;atp6v1g3;pgd;ampd1;p4ha1;eif3e;onecut2;scap;ttc1;ptges;fbxo47;atg12;stat1;nat1;pycr1;gmppb;napg;gdpd3;runx1;ldhc;slc6a5;sycp3;mrpl35;eda2r;foxp4;sap30bp;ears2;spcs2;znf558;qprt;dusp10;znf33b;nr4a2;tyr;tfip11;map3k7;fkbp6;cdk2ap1;pparg;cdc16;sumo1;znf414;ctsa;atp5o;vps72;fbxl17;afmid;cdc42bpb;b3gnt4;sqstm1;traf7;neurod6;ddah1;cldn14;prmt2;csnk1g3;abcf1;cpa3;acsm5;chaf1a;ikzf4;nhlrc2;acadl;ern1;med20;paics;kynu;pltp;cpxm1;foxj3;mars2;ampd3;zfp42;ccnt2;pwp1;atf3;hsd17b13;pou3f2;rbm14;tlr2;znf214;sox9;wars2;utf1;pou4f3;ppm1k;ganab;ugdh;vgll4;pcsk7;pou2f1;sncb;polr2d;mrps18b;znf605;prkaa2;pja2;gsk3b;gtf2e1;diras3;coro2a;hey2;nck1;trim54;neu3;sdha;soat1;psmd14;mrpl10;aldh5a1;lrrk1;dnajc17;hoxa3;dera;mvd;rnf186;b3galt5;wars;slc25a10;prkca;nhp2l1;plcz1;mthfd1l;mettl1;adam10;znf175;sugt1;grip1;tmem48;taf4;ajap1;adm;cops2;sohlh2;usp11;dlx5;araf;slc7a7;papd5;eid2;fbxo9;cdkn3;med18;slc30a9;lrpap1;sf3b2;ppie;ing3;whsc2;nr5a2;taf6;agxt2l1;qdpr;lrrc14;six6;cmas;fbxw8;malt1;papss1;picalm;hars;sp5;hells;zbtb12;hoxc12;klf15;osbpl7;chst1;aurkb;plagl2;hexa;runx3;pax8;lcorl;upf3a;foxn1;neil1;map3k9;supt16h;chst8;klf1;hpse;sms;dcp1a;pdss2;pepd;lancl2;nr0b2;nfatc2ip;casp7;znf786;ldha;sars;b3galt2;ptpn21;senp1;herc6;eif4h;aipl1;crnkl1;pigu;cfi;prdm12;eef1d;il1rap;copg2;cdkn2d;rbck1;chst12;sox6;pcna;aloxe3;setdb2;atp6v0b;sirt5;hnrnpl;pknox1;skiv2l2;imp4;atp2a2;axl;rad51;klhl15;aco1;mark1;atp5b;tfap2c;trpv5;fbxo38;aldh7a1;slc38a4;hif3a;snip1;clcf1;pgk2;pnn;rragc;aurkaip1;anapc7;lin7a;idh3a;gga3;twf1;elovl7;asphd1;lrrc41;grhl2;rcc1;scml2;ilf2;btk;psma8;rps3;rpp30;pole3;ndor1;nt5c1a;npas1;farsa;enosf1;ftsj1;tnp2;sgpl1;psmb5;rnpc3;krba2;alg3;pip4k2a;usp33;st3gal4;nit1;znf689;mchr1;cd74;znf131;pigb;elf3;wee2;crx;trub2;znf398;gan;snd1;dmpk;fbp2;rpl9;gtf2a1;acly;jak1;tubb6;ca2;rsl1d1;nab1;hk3;nuak1;epc2;zbtb38;pdha1;phospho2;gch1;nars;cdt1;dot1l;gmps;dbh;scnm1;pigq;exosc10;cfb;pla2g12a;mcee;nrbp1;tshz3;nsf;slc27a2;msrb3;gphn;arg1;sucla2;st13;cdk9;tspan8;sf3a2;chst14;zadh2;hif1an;st6galnac4;rrm2b;pop5;ets2;rnf139;znf41;eif3f;sirt3;zbtb33;phka2;dpysl3;smad6;runx1t1;uchl5;xpo6;prdm14;snrpe;mki67ip;usp39;zxdc;usp32;aprt;dcp2;rdbp;adam12;dffa;bace2;zcrb1;asah2;nr4a1;mrps35;supt4h1;ldhb;usp13;twist1;rbl2;bhmt;traf6;ogt;epha3;irf5;pcbp3;sdf2;ucn2;tst;lats2;decr1;ldlrap1;myd88;fbxl22;ca9;hdac10;st6gal1;tcf23;zbtb45;phkg1;fuca2;ccnd3;leprel2;flrt2;mmp9;dlst;atf7;adam21;grpel2;stard5;sp1;stat3;gnpda1;pfkfb2;cpb1;maff;atp6v1d;b3galt1;znf501;brms1l;snrk;clk3;junb;foxc1;ripk2;lyz;pgam2;hoxd8;tgfb2;cbs;meis3;bclaf1;kpna3;mrps9;me2;cux1;hoxc9;zbtb16;znf768;polh;mtf1;mmp12;dio3;serinc1;prrx1;srpk1;trpc5;bub1b;trmu;rnf2;sphar;arv1;klk13;mrps18c;ssrp1;pogk;slc20a1;slc2a2;brs3;cdca7;capza3;ripply1;gpaa1;g6pd;hmgb4;sh3rf2;mkl1;tox3;inppl1;cyb561;taok2;znf446;rcor2;rfx5;tlk1;acsl3;tox;ndufs1;ddr2;zdhhc7;fen1;ccnb1ip1;ttll5;dek;man1a1;maoa;rabggta;ppp4r2;ap3s1;mrpl22;tbx10;mtap;plod1;chdh;enpp2;tor2a;pbrm1;znfx1;ppp1cb;pam;gnptg;eif1ax;ogfod1;map3k2;ctnna1;fdps;trip13;ppm1l;hacl1;cdyl2;cdkl2;phtf1;klk9;slc27a4;ncoa2;adra1b;cct6b;mgat4a;ogdh;kmo;bckdhb;amd1;eif5;aldh8a1;uap1;cyp2e1;eya4;elavl2;ctnnb1;scrn1;uqcrh;rax;rpp14;l3mbtl3;rdh11;hdac9;flt1;cpa6;mapkapk5;rnps1;utp3;hoxb3;uroc1;adat3;rps10;srpx2;il17a;csnk1a1;nedd4l;gpx4;eef1e1;ranbp9;agmat;cercam;cdk10;tsc22d4;spaca3;pigs;elf1;ugt1a9;rad23a;gabpb2;th;hectd3;dhx38;pcgf1;a4gnt;aoah;krt1;olr1;arid5a;srms;atg4a;adk;aadacl3;upp1;creg1;c1qtnf2;psmc5;aldh1l1;dlx3;alkbh1;impa1;capn2;acadsb;fasn;ptk2;ppil3;acaca;has3;rbm3;dmc1;mark2;prkra;znf548;map3k4;fdxr;c1qc;ctss;ccbl2;glyctk;lcor;acox2;nr5a1;rce1;acoxl;pyroxd1;cdc42ep5;fancf;gmeb2;cds1;ptpn18;chd3;pabpc4;capg;tmem68;fubp1;klk12;n6amt2;enpp4;rrp9;znf664;keap1;nfatc2;zfp2;eif1b;cnbp;fech;mtmr7;npr2;hnrpll;dlx2;polr3c;hmgn2;dip2a;ptpla;fbxl4;sord;bcl11b;gdpd1;rab10;rab37;aurkc;tgm7;dpp8;lonp1;pdss1;gfpt2;clock;cdc73;ddx54;elk1;znf546;cnot6;srm;pdxp;ero1lb;alox12;med23;ddhd1;klf12;znf197;vax1;uhrf2;serpina5;hyal3;ubqln4;ctbp2;cpa4;ssu72;senp3;ap1s3;cept1;b3gnt2;tlx3;tinag;ftsj2;ap1g1;zfp1;nubp1;chka;psap;znf710;wnt2;ube2m;gaa;oxsr1;mrpl36;pank4;ndufb9;dus1l;rpp38;tfe3;fbxl13;il20;scd;st3gal1;itk;rps8;lonrf1;tinf2;rnf14;eif2ak2;prkaa1;zscan29;abi2;map4k3;hdx;znf771;pum2;scml1;rbm17;ero1l;agk;rrm2;rarb;pja1;ovol2;ugt1a6;tpst1;brf2;acot9;habp4;meox2;ict1;asz1;znf524;grm8;sox15;bcat2;znf787;liph;pdk2;vrk1;scmh1;znf623;nqo2;znf688;mttp;prps2;ocrl;psph;tnp1;tgm5;tnks;rexo2;gja1;trim24;gabpa;mrpl27;apobec1;uimc1;neu2;rbm5;crim1;fut3;nanog;dhrs9;adam32;gcnt4;pld2;mid1;map2k7;mut;cda;acvr2b;lcmt1;fip1l1;dusp13;pdcd4;ilf3;prpf6;adrm1;opn1sw;clp1;znf24;vgf;stk39;cnot4;nek8;ubtd2;slc17a4;minpp1;ephx1;pdia2;srd5a3;tnfaip3;pafah1b3;abt1;ugcg;b3gnt5;camk2g;ctcf;tcea3;exosc7;isca1;ipmk;nxn;hspb1;ncln;zmat5;pcgf2;cryl1;cndp2;rplp1;csrp2bp;dedd;tbpl2;isg20;gmppa;pak3;cdc40;hadh;crebzf;trub1;smc5;cog2;crem;casz1;terf2;ca13;dck;ttll9;cd28;sirt4;npm2;atp11b;sox5;prdm4;hnrnpr;atf2;dbx2;znf454;nr6a1;prtn3;epha1;med24;tubgcp4;pskh1;gzf1;lman1;ubqln2;tspyl2;igsf1;rfx3;nsun2;brsk1;cstf2t;denr;eif3i;trove2;mecp2;atp2b3;pnmt;rnaseh1;dnaja4;st8sia5;adad1;rad1;fbxl6 |
| regulation of biological quality; | 110#953 | 4.53772E-09 | ccr8;nck1;egln2;rragc;shc1;pdia2;ctnnb1;slc22a4;asz1;ccr7;alox12;kng1;brs3;camk2d;capza2;uts2;hamp;ldlrap1;lep;capza3;plaur;cckbr;fhl1;nxn;serpinc1;net1;procr;prkca;nras;taok2;tfpi2;apoh;bcl6;cdkn2aip;il3;cdc42ep5;nedd4l;gna12;nts;ccl28;dcbld2;ndufs1;ccr2;slc9a8;pabpc4;htra1;capg;iqcb1;c4bpb;socs1;brms1l;gp6;gna13;wnt2;agtr1;mchr1;rasa1;plp1;gp1ba;ebag9;pth2r;chrne;entpd1;foxc1;edn1;mbp;cdkn2d;egr2;tgfb2;azu1;thbd;mcl1;atp2c1;ing3;npr2;crim1;qsox2;cd93;krt1;nr5a2;cacna1f;rhcg;npr3;ing1;igfbp6;lman1;atp2a2;tgfb1;slc9a5;tspyl2;slc30a1;creg1;cda;cln5;wisp1;ghsr;f3;slc9a7;napg;kcnmb4;adra1b;gch1;esm1;tfrc;fgf20;kcna5;hexa;ccr9;f7;nrg3 |
| protein transport; | 102#866 | 5.43743E-09 | stx10;snx15;chml;duoxa2;lin7a;gga3;nsf;ran;ykt6;arfip1;synj2bp;uso1;snx25;rtp4;scamp2;chmp7;stx16;vps16;nup35;nfkbil1;srp72;gle1;vps52;rpgr;ctsa;stx7;taok2;tomm22;sec61a2;necap1;bcl6;senp2;pex16;arfgap1;tlk1;ramp1;sec61a1;ap1s3;vps26b;scfd1;ap1g1;rabep2;cog7;tmem48;rab8a;vps41;rhob;ap3d1;ywhab;cep57;hspd1;tnks;cog2;snx14;cog5;kpna2;cd74;atg7;copg2;xpo5;kpna6;stx4;ap3s1;xpo6;plekha8;vps53;pcna;snx5;chmp4a;tnpo3;kpna3;rab3c;rab3ip;gnptg;srp54;stxbp1;prkci;atg4a;ddx19b;rtp3;nupl2;lman1;tgfb1;cdc37;kif18a;vps33a;klhl2;stat1;snx27;snx22;exoc3;rab10;optn;rab37;napg;sec24b;vps26a;rab9b;rab33a;pex12;gosr1;gsk3b |
| regulation of transferase activity; | 41#247 | 6.08481E-09 | adora2b;rgs4;shc1;edn1;traf6;ccng1;ern1;cdkn2d;map3k9;cdkn3;azu1;sfn;lats2;trib3;map3k7;map3k4;madd;dgka;spry1;pak1;ccnt2;map3k2;ccnd3;malt1;tspyl2;dgkb;prkca;gab1;taok2;cdc37;prkaa1;dgkz;dusp22;traf7;dbnl;serinc1;c1qtnf2;pdcd4;agk;rbl2;diras3 |
| metabolic process#regulation of metabolic process; | 362#4150 | 6.82064E-09 | foxp4;ikzf1;sap30bp;tigd3;hoxd1;tshz3;znf558;znf566;znf33b;znf548;nr4a2;pparg;znf414;sumo1;lcor;klf11;cdk9;vps72;nr5a1;hif1an;il3;sqstm1;traf7;cdc42ep5;neurod6;gmeb2;chd3;glis2;pstk;ets2;capg;vsx1;cdx2;fubp1;aplp1;znf41;rasa1;sirt3;eif3f;zbtb33;kpna2;smad6;chaf1a;edn1;ikzf4;myog;znf664;ern1;keap1;runx1t1;med20;nfatc2;prdm14;zfp2;cnbp;eif1b;zxdc;polr3c;dlx2;hmgn2;foxj3;zfp42;ccnt2;atf3;msx1;pou3f2;rbm14;rdbp;zbtb25;dyrk1b;bcl11b;znf214;sox9;bace2;utf1;pou4f3;eif4g2;rab10;rab37;nr4a1;vgll4;supt4h1;pou2f1;alx1;sirt1;clock;znf605;rbl2;twist1;gtf2e1;hey2;bhmt;nck1;trim54;ddx54;elk1;znf546;cnot6;elf5;irf5;ran;med23;vax1;znf197;trib3;klf12;myd88;serpina5;hdac10;hoxa3;hsp90aa1;tcf23;fubp3;zbtb45;ccnd3;zbtb32;prkca;bcl6;gsc;tlx3;znf175;usf1;atf7;stat3;sp1;grip1;zfp1;taf4;maff;sohlh2;socs1;brms1l;znf501;znf710;cbfa2t2;ube2m;dlx5;junb;mlxipl;foxc1;ripk2;eid2;tfe3;hoxd8;azu1;il20;slc30a9;med18;ezh2;meis3;ppie;ing3;bclaf1;yap1;whsc2;ihh;nr5a2;taf6;igf2bp3;cux1;zbtb16;hoxc9;rnf14;znf768;six6;polh;zscan29;prkaa1;irx3;sfpq;mtf1;eif2ak2;znf581;hdx;znf771;sp5;hells;prrx1;zbtb12;srpk1;hoxc12;klf15;pum2;hcls1;scml1;creb3;rarb;plagl2;rnf2;ovol2;ift88;runx3;pax8;brf2;habp4;lcorl;meox2;ssrp1;eya3;pogk;foxn1;znf524;grm8;dnm2;ikzf3;cdca7;supt16h;sox15;capza3;ripply1;zscan21;klf1;hmgb4;mkl1;znf787;tox3;esrrg;lancl2;nr0b2;znf446;rcor2;rfx5;nfatc2ip;tlk1;znf786;scmh1;tox;znf623;znf688;samd4a;cited2;zbtb20;tnp1;eif4h;dek;duxa;gja1;prdm12;cdkn2d;trim24;gabpa;zfx;tbx10;med8;uimc1;sox6;sp2;pcna;nlk;sirt5;pbrm1;znfx1;pknox1;znf496;nanog;tgfb1;rad51;acvr2b;cda;elk4;ghsr;phtf1;tfap2c;pdcd4;ncoa2;drgx;ilf3;adra1b;prpf6;hif3a;znf24;tceal8;snip1;clcf1;eif5;cnot4;pnn;egln2;tox4;mixl1;aurkaip1;hus1;eya4;ctnnb1;elavl2;polr2f;znf354a;capza2;rax;abt1;znf451;grhl2;lep;l3mbtl3;scml2;hdac9;ilf2;plaur;ctcf;tcea3;hspb1;hoxb3;ppargc1b;pcgf2;npas1;nedd4l;dedd;pias1;set;tbpl2;klf5;rab8a;krba2;crebzf;tsc22d4;znf689;crem;casz1;terf2;mysm1;sirt4;cd28;tead1;znf131;sim2;elf1;egr2;sox5;prdm4;gabpb2;elf3;znf398;crx;pcgf1;dbx2;atf2;znf454;nr6a1;snd1;med24;eif3e;gtf2a1;arid5a;onecut2;scap;gzf1;tspyl2;igsf1;creg1;stat1;rfx3;nab1;c1qtnf2;psmc5;epc2;eif3i;zbtb38;runx1;dlx3;mecp2;cdt1;eda2r |
| protein localization; | 110#961 | 7.96645E-09 | stx10;snx15;chml;duoxa2;lin7a;pdia2;gga3;nsf;ran;ykt6;arfip1;synj2bp;uso1;snx25;rtp4;scamp2;chmp7;gpaa1;stx16;vps16;nup35;sergef;nfkbil1;krt20;srp72;gle1;vps52;rpgr;ctsa;stx7;taok2;tomm22;sec61a2;necap1;bcl6;senp2;pex16;arfgap1;tlk1;ramp1;sec61a1;sqstm1;ap1s3;vps26b;scfd1;ap1g1;rabep2;cog7;tmem48;rab8a;vps41;ap3d1;rhob;ywhab;cep57;hspd1;tnks;cog2;snx14;cog5;kpna2;shroom2;cd74;atg7;copg2;xpo5;kpna6;stx4;ap3s1;xpo6;plekha8;vps53;pcna;snx5;chmp4a;nlrp2;tnpo3;kpna3;rab3c;rab3ip;gnptg;srp54;stxbp1;prkci;atg4a;ddx19b;rtp3;nupl2;lman1;tgfb1;kif18a;cdc37;vps33a;klhl2;stat1;snx27;bace2;snx22;exoc3;rab10;optn;rab37;napg;sec24b;vps26a;rab9b;rab33a;pex12;gosr1;gsk3b |
| regulation of kinase activity; | 40#242 | 1.20588E-08 | adora2b;rgs4;shc1;edn1;traf6;ccng1;ern1;cdkn2d;map3k9;cdkn3;azu1;sfn;lats2;trib3;map3k7;map3k4;madd;dgka;spry1;pak1;ccnt2;map3k2;ccnd3;malt1;tspyl2;dgkb;prkca;gab1;taok2;cdc37;prkaa1;dgkz;dusp22;traf7;dbnl;c1qtnf2;pdcd4;agk;rbl2;diras3 |
| biopolymer modification; | 260#2815 | 1.20588E-08 | pigq;ttbk1;ptk2;fbxo10;usp30;spcs2;myo3a;nrbp1;mark2;dusp10;prkra;map3k7;fut1;map3k4;cdk2ap1;cdc16;sumo1;glyctk;cdk9;tspan8;fbxl17;tec;cdc42bpb;b3gnt4;il3;traf7;plod2;arih1;st3gal3;st6galnac4;ptpn18;phkg2;rbbp6;rnf139;prmt2;apobec2;csnk1g3;sirt3;phka2;akt2;ern1;uchl5;mtmr7;mex3b;npr2;usp32;abl2;fbxl4;rbm14;dyrk1b;usp15;fbxl8;bace2;ppm1k;map4k2;aurkc;tgm7;pcsk7;sirt1;hectd2;pja2;usp13;prkaa2;gsk3b;bhmt;traf6;ogt;ero1lb;epha3;sdf2;lrrk1;lats2;trib3;uhrf2;cdc14b;fbxl22;hdac10;ubqln4;st6gal1;phkg1;b3galt5;ccnd3;prkca;tmub2;cpa4;senp2;ppm1d;senp3;mettl1;adam10;b3gnt2;sugt1;camk4;socs1;b3galt1;usp11;snrk;wnt2;clk3;ddb1;ube2m;oxsr1;fbxo45;araf;ripk2;fbxo9;il20;fbxl13;st3gal1;itk;ihh;zbtb16;rnf14;fbxw8;malt1;prkaa1;eif2ak2;abi2;map4k3;hells;srpk1;cdk6;hcls1;bub1b;ero1l;agk;aurkb;pja1;rnf2;senp8;tpst1;hunk;asz1;map3k9;asb2;rapgef4;camk2d;gpaa1;sh3rf2;adprh;taok2;tlk1;nfatc2ip;pdk2;vrk1;pim1;ddr2;mttp;zdhhc7;b3galt2;galnt6;ptpn21;ulk3;tgm5;tnks;senp1;herc6;ccnb1ip1;ttll5;aipl1;pigu;man1a1;rabggta;atg7;ppp4r2;rbck1;apobec1;med8;nlk;plod1;galnt9;sirt5;fut3;syvn1;ppp1cb;pam;gnptg;prkci;tp53rk;pak1;map3k2;tgfb1;axl;mapk1;gcnt4;mid1;dusp22;map2k7;acvr2b;lcmt1;ppm1l;klhl15;mark1;gucy2c;cdkl2;dusp13;fbxo38;opn1sw;ppp1cc;mgat4a;stk39;clcf1;cnot4;rnf41;nek8;ubtd2;hus1;anapc7;tnfaip3;twf1;lrrc41;asphd1;b3gnt5;camk2g;flt1;hdac9;btk;ctcf;usp2;mapkapk5;ncln;taok3;il17a;csnk1a1;nedd4l;pias1;set;pak3;alg3;trub1;usp33;cdk10;st3gal4;cog2;lrrc29;ttll9;sirt4;pigs;pigb;rad23a;wee2;hectd3;trub2;a4gnt;gan;epha1;dmpk;srms;pskh1;atg4a;ubqln2;jak1;fbxo47;atg12;stat1;nsun2;brsk1;nuak1;st8sia5;fbxl6 |
| protein localization#establishment of protein localization; | 106#922 | 1.23115E-08 | stx10;snx15;chml;duoxa2;lin7a;gga3;nsf;ran;ykt6;arfip1;synj2bp;uso1;snx25;rtp4;scamp2;chmp7;stx16;vps16;nup35;sergef;nfkbil1;krt20;srp72;gle1;vps52;rpgr;ctsa;stx7;taok2;tomm22;sec61a2;necap1;bcl6;senp2;pex16;arfgap1;tlk1;ramp1;sec61a1;ap1s3;vps26b;scfd1;ap1g1;rabep2;cog7;tmem48;rab8a;vps41;ap3d1;rhob;ywhab;cep57;hspd1;tnks;cog2;snx14;cog5;kpna2;cd74;atg7;copg2;xpo5;kpna6;stx4;ap3s1;xpo6;plekha8;vps53;pcna;snx5;chmp4a;tnpo3;nlrp2;kpna3;rab3c;rab3ip;gnptg;srp54;stxbp1;prkci;atg4a;ddx19b;rtp3;nupl2;lman1;tgfb1;kif18a;cdc37;vps33a;klhl2;stat1;snx27;bace2;snx22;exoc3;rab10;optn;rab37;napg;sec24b;vps26a;rab9b;rab33a;pex12;gosr1;gsk3b |
| protein modification process; | 251#2704 | 1.47474E-08 | tpst1;pigq;ttbk1;ptk2;hunk;fbxo10;usp30;asz1;spcs2;myo3a;map3k9;nrbp1;asb2;mark2;rapgef4;dusp10;camk2d;prkra;map3k7;fut1;map3k4;gpaa1;cdk2ap1;sh3rf2;adprh;cdc16;sumo1;glyctk;cdk9;tspan8;taok2;fbxl17;tec;cdc42bpb;nfatc2ip;b3gnt4;tlk1;il3;traf7;plod2;vrk1;pdk2;pim1;arih1;st3gal3;st6galnac4;ptpn18;phkg2;ddr2;zdhhc7;mttp;rbbp6;b3galt2;rnf139;prmt2;galnt6;ptpn21;tgm5;ulk3;tnks;csnk1g3;senp1;ccnb1ip1;herc6;sirt3;aipl1;ttll5;phka2;pigu;man1a1;akt2;atg7;rabggta;ern1;ppp4r2;rbck1;uchl5;med8;mtmr7;mex3b;plod1;nlk;npr2;galnt9;sirt5;fut3;syvn1;gnptg;pam;ppp1cb;usp32;prkci;pak1;tp53rk;abl2;fbxl4;map3k2;axl;tgfb1;rbm14;mapk1;gcnt4;dyrk1b;usp15;mid1;fbxl8;dusp22;map2k7;lcmt1;acvr2b;bace2;klhl15;ppm1k;ppm1l;map4k2;mark1;cdkl2;gucy2c;dusp13;aurkc;fbxo38;tgm7;pcsk7;opn1sw;sirt1;ppp1cc;mgat4a;hectd2;stk39;clcf1;pja2;usp13;prkaa2;gsk3b;cnot4;bhmt;rnf41;ubtd2;nek8;anapc7;traf6;hus1;ogt;ero1lb;epha3;tnfaip3;sdf2;lrrk1;twf1;lats2;asphd1;trib3;lrrc41;uhrf2;b3gnt5;cdc14b;fbxl22;camk2g;hdac9;flt1;btk;hdac10;usp2;ubqln4;st6gal1;mapkapk5;phkg1;b3galt5;ccnd3;prkca;tmub2;ncln;cpa4;taok3;senp2;ppm1d;senp3;adam10;il17a;nedd4l;csnk1a1;b3gnt2;sugt1;pias1;set;pak3;alg3;camk4;socs1;cdk10;usp33;b3galt1;usp11;st3gal4;cog2;snrk;wnt2;lrrc29;clk3;ddb1;ube2m;ttll9;oxsr1;sirt4;fbxo45;araf;ripk2;pigs;pigb;fbxo9;rad23a;wee2;fbxl13;il20;hectd3;st3gal1;itk;a4gnt;gan;ihh;epha1;dmpk;zbtb16;rnf14;srms;atg4a;pskh1;fbxw8;ubqln2;malt1;jak1;prkaa1;eif2ak2;abi2;stat1;atg12;fbxo47;map4k3;brsk1;srpk1;cdk6;nuak1;hcls1;bub1b;ero1l;aurkb;pja1;rnf2;senp8;st8sia5;fbxl6 |
| programmed cell death#regulation of programmed cell death; | 74#576 | 1.58317E-08 | traf6;actn4;sap30bp;alox12;tnfaip3;kng1;dnm2;prkra;plekhf1;btk;sema4d;bcl2l11;dcc;adamtsl4;hspb1;prkca;nras;mmp9;tnfrsf25;bcl6;il3;traf7;pdcd5;dedd;pim1;eef1e1;tp53bp2;ciapin1;lalba;hspd1;faim;rasa1;cd70;cd28;vegfa;cd74;cdh13;traf3;foxc1;ripk2;ern1;cdkn2d;cd2;tgfb2;azu1;sfn;traf1;mcl1;birc3;bclaf1;nlrp2;madd;ihh;ngfr;bik;ctnna1;tgfb1;malt1;prkaa1;tlr2;mapk1;dffa;alb;arhgdia;stat1;sox9;psmc5;gch1;sycp3;plagl2;tax1bp1;clcf1;gsk3b;runx3 |
| multicellular organismal development#embryonic development; | 39#235 | 1.60875E-08 | meox2;npm2;foxc1;edn1;gja1;hus1;sfrp2;cops3;sim2;ctnnb1;dscaml1;keap1;tgfb2;hoxd8;thbd;elf3;prkra;ripply1;ihh;nr5a2;flt1;ipmk;nanog;msx1;nle1;slc30a1;gsc;pou4f3;adam10;tinag;hcn4;cdx2;maff;gna13;rasa1;eda2r;nrg3;dlx5;ift88 |
| localization#establishment of localization; | 358#4135 | 2.15123E-08 | stx10;snx15;btbd10;chml;nox1;nrbp1;nsf;chmp7;vps16;fdxr;lrp10;c1qc;vps52;ctsa;atp5o;slc4a2;gabrb2;tomm22;sec61a2;necap1;cacng6;ramp1;sqstm1;traf7;vps26b;cacng7;slc17a7;slc30a4;trpc7;kifc3;aplp1;kpna2;kcns3;edn1;cdh13;xpo5;stx4;c1qtnf1;xpo6;cthrc1;clic4;pltp;elmo1;sv2b;akap8;dnm1l;rtp3;nupl2;atp11a;tlr2;cdc37;kctd18;vps33a;kcne1;bace2;snx27;map4k2;lrp6;rab10;rab37;kcnmb4;tmem37;colec11;rab33a;pkd2l1;kcnq2;pex12;tfrc;tm9sf2;gsk3b;adora2b;c1qtnf3;sdha;aqp1;kcnc1;galr1;ero1lb;ran;tst;syt9;ykt6;slc18a2;ldlrap1;uso1;slc13a1;ca9;slc29a3;asgr2;srp72;kctd8;hsp90aa1;nxf1;slc31a1;stx7;abcd3;slc25a10;kcna2;prkca;pram1;slc7a6;snph;senp2;bcl6;slc6a2;arfgap1;sec61a1;slc4a8;gnai3;ap1s3;stard5;ap1g1;rabep2;cog7;tmem48;camk4;vps41;scn1b;chka;adm;atp6v1d;psap;rae1;doc2a;wnt2;slc30a6;slco6a1;grid2;chrna4;kcnj13;ccbe1;slc22a16;slc7a7;kpna6;syt12;azu1;osbpl2;plekha8;slc30a9;vps53;lrpap1;kif3b;clcn5;chmp4a;slc39a14;kpna3;cd93;slc2a12;slc6a19;rhcg;slc8a2;slco1b3;spns3;pacsin1;malt1;kif18a;klhl2;kctd12;snx22;picalm;optn;slc9a7;serinc1;slc10a5;sec24b;klf15;trpc5;osbpl7;hcn4;kcnf1;ero1l;ift88;nudt9;slc25a43;cd14;upf3a;ankrd27;kif9;atp13a5;actn4;asz1;kcnk2;slc20a1;slc2a2;kctd9;pitpnc1;rapgef4;svop;dnm2;arfip1;scn1a;scamp2;kcnab3;cachd1;dnm3;nup35;sergef;nfkbil1;gle1;scara5;rpgr;kctd19;vdac1;cyb561;abcg2;trpm8;taok2;tlk1;pex16;kcnip4;dbnl;clca1;chrna5;coro1a;acbd5;mttp;ap3d1;ywhab;slc16a4;tnks;kcns1;arfgef2;slc22a18;shroom2;gja1;atg7;syt10;copg2;kcnj11;ap3s1;slc5a3;pcna;atp2c1;kcnv2;atp6v0b;nalcn;nlrp2;rab3c;stau2;gnptg;srp54;kif3a;prkci;ddx19b;nusap1;atp2a2;slc9a5;tgfb1;trpv1;pld2;kcnk6;shbg;exoc3;dync1i1;ghsr;atp5b;cacng3;kcnj5;slc37a3;sfxn3;vps26a;slc25a38;trpv5;slc27a4;slc38a4;slc23a2;slc39a7;ergic3;rragc;slc17a4;duoxa2;cxcl16;lin7a;ctnnb1;slc22a4;slc16a14;fxyd5;gga3;scrn1;slc16a3;kcng1;kng1;uqcrh;heph;synj2bp;fa2h;snx25;cnnm2;rtp4;scnn1b;camk2g;stx16;krt20;best4;kcnk1;scarf1;cacna2d3;slc6a17;kcnq1;asna1;trappc6a;abcg8;slc30a10;nedd4l;tnp2;set;scfd1;best1;rab8a;slc9a8;slc22a14;rhob;slc39a6;cep57;hspd1;cog2;agtr1;snx14;cog5;chrne;kif1c;cd74;spaca3;atp11b;kcnj12;rbp7;cnga3;trappc1;slc25a17;atp6v1g3;snx5;tnpo3;slc16a13;tspan18;rab3ip;cacna1f;fxyd1;slc35f2;stxbp1;atg4a;mapk8ip1;lman1;tubb6;csn2;ca2;cacnb3;alb;slc30a1;stat1;aqp11;col8a2;c1qtnf2;napg;lmbr1l;rab9b;atp2b3;slc6a5;kcna5;gosr1 |
| regulation of protein kinase activity; | 39#237 | 2.30185E-08 | adora2b;rgs4;shc1;edn1;traf6;ccng1;ern1;cdkn2d;map3k9;cdkn3;azu1;sfn;lats2;trib3;map3k7;map3k4;madd;dgka;spry1;pak1;ccnt2;map3k2;ccnd3;malt1;tspyl2;dgkb;prkca;gab1;cdc37;prkaa1;taok2;dgkz;dusp22;traf7;dbnl;c1qtnf2;pdcd4;agk;diras3 |
| regulation of apoptosis; | 73#570 | 2.39935E-08 | traf6;actn4;sap30bp;alox12;tnfaip3;kng1;dnm2;prkra;plekhf1;btk;sema4d;bcl2l11;dcc;adamtsl4;hspb1;prkca;nras;mmp9;tnfrsf25;bcl6;il3;traf7;pdcd5;dedd;pim1;eef1e1;tp53bp2;ciapin1;lalba;hspd1;faim;rasa1;cd70;cd28;vegfa;cd74;cdh13;traf3;foxc1;ripk2;ern1;cdkn2d;cd2;tgfb2;azu1;sfn;traf1;mcl1;birc3;bclaf1;nlrp2;madd;ihh;ngfr;bik;ctnna1;tgfb1;malt1;prkaa1;tlr2;mapk1;dffa;alb;arhgdia;stat1;sox9;gch1;sycp3;plagl2;tax1bp1;clcf1;gsk3b;runx3 |
| macromolecule localization; | 113#1012 | 2.42111E-08 | stx10;snx15;chml;upf3a;duoxa2;lin7a;pdia2;gga3;nsf;ran;ykt6;arfip1;synj2bp;uso1;snx25;rtp4;scamp2;chmp7;gpaa1;stx16;vps16;nup35;sergef;nfkbil1;krt20;srp72;gle1;vps52;nxf1;rpgr;ctsa;stx7;taok2;tomm22;sec61a2;necap1;bcl6;senp2;pex16;arfgap1;tlk1;ramp1;sec61a1;sqstm1;ap1s3;vps26b;scfd1;ap1g1;rabep2;cog7;tmem48;rab8a;vps41;ap3d1;rhob;ywhab;cep57;rae1;hspd1;tnks;cog2;snx14;cog5;kpna2;shroom2;cd74;atg7;copg2;xpo5;kpna6;stx4;ap3s1;xpo6;plekha8;vps53;pcna;snx5;chmp4a;nlrp2;tnpo3;kpna3;rab3c;rab3ip;gnptg;srp54;stxbp1;prkci;atg4a;ddx19b;rtp3;nupl2;lman1;tgfb1;kif18a;cdc37;vps33a;klhl2;stat1;snx27;bace2;snx22;exoc3;rab10;optn;rab37;napg;sec24b;vps26a;rab9b;rab33a;pex12;gosr1;gsk3b |
| positive regulation of signal transduction; | 29#153 | 2.46072E-08 | sectm1;malt1;ltbr;taok2;vegfa;traf6;cdh13;gja1;taok3;ripk2;cdkn2aip;eef1d;acvr2b;il3;lgals9;slc20a1;traf7;ghsr;il20;atp2c1;myd88;hcls1;cited2;trim38;traf3ip2;eda2r;clcf1;trim13;ctnna1 |
| cell cycle phase; | 53#369 | 2.8501E-08 | kpna2;cd28;aurkaip1;npm2;shc1;anapc7;hus1;papd5;ccng1;cdkn2d;bub3;cdkn3;dmc1;xrcc2;ran;rad54l;dnm2;lats2;hectd3;ndc80;rcc1;pbrm1;cdk2ap1;mad2l2;dctn1;nde1;cdc16;akap8;nusap1;ncaph;tgfb1;rad51;bcl6;brsk1;hells;cdc27;dusp13;sugt1;cdk6;mns1;coro1a;ilf3;stag1;bub1b;sycp3;cdt1;aurkb;cdk10;pds5a;sac3d1;sgol1;rad1;ncapg |
| multicellular organismal process#regulation of multicellular organismal process; | 43#277 | 4.29405E-08 | nck1;cfi;cd28;edn1;traf6;spaca3;gja1;cd74;cfb;kcnj12;ppp1r12b;elf1;cd2;tgfb2;slc5a3;map3k7;myd88;lep;dmpk;tpm1;krt1;c1qc;des;malt1;atp2a2;tgfb1;prkca;nras;cacnb3;tlr2;kcnq1;kcne1;bcl6;pxk;chrm2;pag1;kcnmb4;mrcl3;adra1b;hcn4;vsig4;c4bpb;cd274 |
| nucleobase, nucleoside, nucleotide and nucleic acid metabolic process; | 480#5848 | 4.64384E-08 | foxp4;exosc10;ikzf1;ppil3;nox1;sap30bp;rbm3;tigd3;hoxd1;ears2;tshz3;znf558;qprt;dmc1;znf566;znf33b;znf548;nr4a2;tfip11;cdk2ap1;pparg;znf414;sumo1;lcor;klf11;cdk9;atp5o;sf3a2;vps72;nr5a1;hif1an;naprt1;sqstm1;traf7;neurod6;fancf;gmeb2;rrm2b;pop5;chd3;glis2;ets2;pabpc4;vsx1;cdx2;fubp1;apobec2;aplp1;znf41;rasa1;sirt3;zbtb33;dpysl3;kpna2;rrp9;smad6;chaf1a;edn1;ikzf4;myog;znf664;ern1;keap1;xpo5;runx1t1;med20;paics;kynu;nfatc2;prdm14;zfp2;cnbp;snrpe;cstf1;mki67ip;usp39;npr2;zxdc;wdr36;hnrpll;mrpl44;polr3c;dlx2;hmgn2;foxj3;aprt;rbm23;mars2;ampd3;zfp42;pwp1;ccnt2;atf3;dcp2;msx1;pou3f2;rbm14;rdbp;zbtb25;dyrk1b;dffa;bcl11b;znf214;sox9;wars2;utf1;pou4f3;zcrb1;eif4g2;rab10;rab37;nr4a1;ugdh;vgll4;supt4h1;pou2f1;alx1;polr2d;sirt1;clock;gin1;znf605;rbl2;twist1;diras3;gtf2e1;cdc73;hey2;h2afz;ddx54;elk1;znf546;cnot6;elf5;irf5;ran;med23;pcbp3;ucn2;vax1;znf197;trib3;klf12;myd88;hoxa3;hdac10;nfkbib;dera;tcf23;fubp3;zbtb45;wars;zbtb32;nhp2l1;ints5;cpa4;bcl6;gsc;mettl1;tlx3;znf175;usf1;ftsj2;atf7;stat3;sp1;grip1;zfp1;taf4;adm;cops2;maff;sohlh2;atp6v1d;brms1l;znf501;znf710;cbfa2t2;ddb1;dlx5;junb;mlxipl;foxc1;ripk2;papd5;dus1l;eid2;tfe3;rpp38;hoxd8;xrcc2;slc30a9;ezh2;med18;znf346;meis3;sf3b2;ing3;ppie;bclaf1;yap1;tinf2;whsc2;ihh;nr5a2;taf6;igf2bp3;cux1;zbtb16;rnf14;hoxc9;znf768;six6;polh;zscan29;irx3;sfpq;mtf1;eif2ak2;slbp;papss1;znf581;hdx;hars;znf771;sp5;hells;prrx1;zbtb12;srpk1;hoxc12;fance;klf15;hcls1;scml1;rbm17;creb3;trmu;rarb;rrm2;plagl2;rnf2;sphar;ovol2;runx3;pax8;brf2;habp4;lcorl;meox2;upf3a;ssrp1;utp6;eya3;pogk;foxn1;neil1;znf524;grm8;dnm2;ikzf3;cdca7;supt16h;sox15;ripply1;zscan21;g6pd;klf1;hmgb4;znf787;mkl1;tox3;dcp1a;syncrip;esrrg;lancl2;nr0b2;znf446;rcor2;rfx5;nfatc2ip;tlk1;znf786;scmh1;tox;znf623;sars;ndufs1;znf688;cited2;hibadh;prps2;adat1;zbtb20;tnp1;tnks;fen1;rexo2;crnkl1;dek;duxa;prdm12;ppp4r2;cdkn2d;trim24;gabpa;zfx;apobec1;tbx10;med8;uimc1;sox6;sp2;pcna;rbm5;mtap;setdb2;nlk;atp6v0b;sirt5;pbrm1;hnrnpl;znfx1;pknox1;znf496;skiv2l2;nanog;imp4;tgfb1;mapk1;rad51;acvr2b;trip13;cda;elk4;cdyl2;atp5b;gucy2c;tfap2c;phtf1;fip1l1;pdcd4;cad;ncoa2;drgx;ilf3;adrm1;prpf6;slc23a2;hif3a;clp1;znf24;tceal8;snip1;uap1;cnot4;pnn;rragc;mixl1;tox4;hus1;eya4;dhx15;ctnnb1;elavl2;polr2f;znf354a;rax;abt1;znf451;grsf1;grhl2;rpp14;rcc1;l3mbtl3;tspyl1;scml2;ilf2;hdac9;ctcf;tcea3;exosc7;rnps1;rpp30;utp3;pole3;hoxb3;adat3;ppargc1b;zmat5;nt5c1a;npas1;farsa;pcgf2;ftsj1;dedd;gpx4;tnp2;pias1;isg20;set;tbpl2;klf5;cdc40;rnpc3;rab8a;krba2;crebzf;trub1;tsc22d4;smc5;znf689;crem;terf2;casz1;dck;mysm1;sirt4;npm2;tead1;znf131;nap1l5;sim2;elf1;egr2;sox5;rad23a;prdm4;rad54l;gabpb2;elf3;crx;dhx38;trub2;znf398;atp6v1g3;pcgf1;pgd;hnrnpr;ampd1;dbx2;atf2;nr6a1;znf454;snd1;med24;gtf2a1;acly;arid5a;adk;onecut2;scap;gzf1;tspyl2;upp1;igsf1;stat1;creg1;nsun2;rfx3;nab1;cstf2t;psmc5;epc2;trove2;zbtb38;runx1;dlx3;mecp2;nars;sycp3;alkbh1;cdt1;dot1l;gmps;scnm1;rnaseh1;adad1;eda2r;rad1 |
| positive regulation of transcription; | 43#279 | 5.82968E-08 | elk1;foxc1;myog;ctnnb1;elf1;ran;nfatc2;nr4a2;sox15;crx;myd88;yap1;ihh;ilf2;dlx2;ctcf;rnf14;scap;tgfb1;rbm14;esrrg;dyrk1b;ppargc1b;sox9;utf1;sqstm1;psmc5;pias1;klf5;klf15;grip1;sp1;ncoa2;ilf3;zbtb38;supt4h1;glis2;cited2;runx1;prpf6;plagl2;clock;pax8 |
| positive regulation of nucleobase, nucleoside, nucleotide and nucleic acid metabolic process; | 44#289 | 6.44697E-08 | elk1;foxc1;myog;ctnnb1;elf1;ran;nfatc2;nr4a2;sox15;crx;myd88;yap1;ihh;ilf2;dlx2;ctcf;rnf14;scap;tgfb1;rbm14;rad51;esrrg;dyrk1b;ppargc1b;sox9;utf1;sqstm1;psmc5;pias1;klf5;klf15;grip1;sp1;ncoa2;ilf3;zbtb38;supt4h1;glis2;runx1;cited2;prpf6;plagl2;clock;pax8 |
| MAPKKK cascade; | 28#150 | 8.35552E-08 | adora2b;gab1;prkca;taok2;mapk1;prkaa1;rgs4;shc1;dusp22;taok3;map2k7;map4k3;il3;traf7;dbnl;cdc42ep5;map3k9;map4k2;dusp10;c1qtnf2;arhgef6;map3k4;myd88;madd;sh2d3c;eda2r;mapk8ip1;map3k2 |
| signal transduction; | 427#5142 | 1.08447E-07 | ptk2;rgs4;rsu1;pde1a;cops3;mark2;dusp10;nr4a2;prkra;dkk2;map3k7;map3k4;cxcr7;ndc80;traf3ip2;pparg;iqsec3;pde11a;grap2;arhgap9;gab1;gabrb2;nr5a1;tnfrsf25;rgs17;tec;cdc42bpb;plek;angptl5;il3;ramp1;sqstm1;traf7;cdc42ep5;axin2;pak1ip1;sparc;adra2b;cds1;ddah1;clec1a;prmt2;gpr82;lalba;gna13;rasa4;cxcl14;csnk1g3;rasa1;gp1ba;zbtb33;gpr124;nrp1;dpysl3;cd70;strn4;smad6;edn1;traf3;cdh13;gfra3;sfrp2;ern1;rasl11a;cd2;iqgap2;rspo1;cd53;npr2;arl16;elmo1;akap8;abl2;dnm1l;tshb;rbm14;tlr2;dffa;arhgdia;cnih2;smap1;snx27;pag1;impa2;asah2;map4k2;lrp6;rab10;rab37;nr4a1;kremen1;rab33a;pkd2l1;rgs21;cck;arhgap21;clock;gabbr1;gnb1;prkaa2;gsk3b;nrg3;diras3;coro2a;adora2b;hey2;xpr1;nck1;trim54;ccr5;ddx54;shc1;gfra2;traf6;fibcd1;ogt;alcam;galr1;rasgrp2;sucnr1;ms4a7;epha3;ran;lrrk1;diras1;lats2;myd88;tmem115;fgf1;iqsec2;tagap;akap4;gpr142;nfkbib;asgr2;hsp90aa1;jmjd6;pygo2;fgd1;rgs11;net1;prkca;dgkb;plcz1;nras;pram1;fgf6;senp2;bcl6;arfgap1;npffr2;adam10;chrm2;angpt4;kiss1r;gnai3;gnb2;sh2b1;stat3;grip1;camk4;wnt5b;adm;gpr55;cops2;socs1;wnt11;pdc;wnt2;oxgr1;ccrl1;pth2r;gnat3;oxsr1;grid2;chrna4;araf;vegfa;gpr68;foxc1;ripk2;eid2;stac;htr1a;tbc1d14;azu1;il20;pnoc;nxph4;itk;madd;ppic;ihh;ngfr;rnf14;igfbp6;arhgef9;ppp2r2b;malt1;nle1;prkaa1;eif2ak2;rassf5;ostf1;map4k3;cysltr2;optn;psd4;srpk1;skap2;hcls1;bub1b;arl4c;fgf20;agk;rarb;inpp1;ccr9;trim13;ift88;sectm1;arhgap15;hunk;cd14;srgap3;kcnk2;slc20a1;map3k9;asb2;grm8;pitpnc1;rapgef4;drd3;brs3;dnm2;depdc7;cx3cl1;sergef;csnk2b;vdac1;evi5;taok2;dgkz;pde1c;cdkn2aip;casp7;tlk1;htr4;appl1;dbnl;dcbld2;rapgef1;chrna5;tp53bp2;arhgef6;ddr2;cited2;trim38;vapb;sh2d3c;ocrl;hdgf;gpr84;itgb1bp2;fen1;gp6;fgf5;cd274;arfgef2;dek;pigu;gper;mpp1;gja1;eef1d;il1rap;ap3s1;traf1;birc3;pcna;tacr3;rgs1;atp2c1;nlk;enpp2;arhgdib;rab3c;gpr128;ltb4r;npr3;pygo1;prkci;pak1;map3k2;ctnna1;atp2a2;axl;tgfb1;nxph3;mapk1;stac2;trpv1;pld2;dusp22;gpr3;map2k7;gpr139;acvr2b;cda;ghsr;gpr174;mark1;cdkl2;gucy2c;cnih4;gprc5d;ncoa2;adra1b;ppp2r5b;mtnr1b;opn1sw;efnb3;hif3a;snip1;clcf1;timp3;ccr8;arl6;egln2;rragc;stac3;spsb3;pdia2;ctnnb1;ccr7;tnfaip3;synj2bp;arhgap1;lep;rgs22;dgka;plaur;flt1;btk;rem1;ppp1r1b;cckbr;rundc3a;spry1;mapkapk5;mfap4;gpr19;trhr;ppargc1b;cxcl11;taok3;npas1;csnk1a1;gna12;pmch;nts;nmbr;grb10;pias1;sh3bp1;ccr2;fzd9;rab8a;il13;cabp4;htra1;rhob;cep57;rab42;ralgps1;rasal2;crem;mchr1;agtr1;snx14;ltbr;chrne;gpr88;cd28;cd74;fzd5;sim2;ppp1r12b;elf1;rassf8;lgals9;prdm4;fstl3;sfn;mkln1;cnga3;mcl1;vav3;kitlg;epha1;igsf6;dmpk;cxxc4;med24;bik;sarm1;mapk8ip1;cnksr1;scap;ptges;jak1;tspyl2;cacnb3;igsf1;wif1;stat1;mln;wisp1;gnal;c1qtnf2;arhgap28;cd164;rab9b;nxph2;arl9;impa1;eda2r |
| transport; | 346#4035 | 1.30793E-07 | stx10;snx15;btbd10;chml;nox1;nrbp1;nsf;chmp7;vps16;fdxr;lrp10;c1qc;vps52;ctsa;atp5o;slc4a2;gabrb2;tomm22;sec61a2;necap1;cacng6;ramp1;sqstm1;traf7;vps26b;cacng7;slc17a7;slc30a4;trpc7;kifc3;aplp1;kpna2;kcns3;edn1;cdh13;xpo5;stx4;c1qtnf1;xpo6;cthrc1;clic4;pltp;elmo1;sv2b;akap8;dnm1l;rtp3;nupl2;atp11a;tlr2;cdc37;kctd18;vps33a;kcne1;snx27;map4k2;lrp6;rab10;rab37;kcnmb4;tmem37;colec11;rab33a;pkd2l1;kcnq2;pex12;tfrc;tm9sf2;gsk3b;c1qtnf3;sdha;aqp1;kcnc1;galr1;ero1lb;ran;tst;syt9;ykt6;slc18a2;ldlrap1;uso1;slc13a1;slc29a3;asgr2;srp72;kctd8;hsp90aa1;nxf1;slc31a1;stx7;abcd3;slc25a10;kcna2;prkca;pram1;slc7a6;snph;senp2;bcl6;slc6a2;arfgap1;sec61a1;slc4a8;gnai3;ap1s3;stard5;ap1g1;rabep2;cog7;tmem48;camk4;vps41;scn1b;chka;atp6v1d;psap;rae1;doc2a;wnt2;slc30a6;slco6a1;grid2;chrna4;kcnj13;ccbe1;slc22a16;slc7a7;kpna6;syt12;azu1;osbpl2;plekha8;slc30a9;vps53;lrpap1;kif3b;clcn5;chmp4a;slc39a14;kpna3;cd93;slc2a12;slc6a19;rhcg;slc8a2;slco1b3;spns3;pacsin1;malt1;kif18a;klhl2;kctd12;snx22;picalm;optn;slc9a7;serinc1;slc10a5;sec24b;klf15;trpc5;osbpl7;hcn4;kcnf1;ero1l;nudt9;slc25a43;cd14;upf3a;ankrd27;kif9;atp13a5;actn4;asz1;kcnk2;slc20a1;slc2a2;kctd9;pitpnc1;rapgef4;dnm2;svop;arfip1;scn1a;scamp2;kcnab3;cachd1;dnm3;nup35;nfkbil1;gle1;scara5;rpgr;kctd19;vdac1;cyb561;abcg2;trpm8;taok2;tlk1;pex16;kcnip4;dbnl;clca1;chrna5;coro1a;acbd5;mttp;ap3d1;ywhab;slc16a4;tnks;kcns1;arfgef2;slc22a18;gja1;atg7;syt10;copg2;kcnj11;ap3s1;slc5a3;pcna;atp2c1;kcnv2;atp6v0b;nalcn;rab3c;stau2;gnptg;srp54;kif3a;prkci;ddx19b;slc9a5;tgfb1;atp2a2;trpv1;pld2;kcnk6;shbg;dync1i1;exoc3;ghsr;atp5b;cacng3;kcnj5;slc37a3;sfxn3;vps26a;slc25a38;trpv5;slc27a4;slc38a4;slc23a2;slc39a7;ergic3;rragc;slc17a4;duoxa2;cxcl16;lin7a;ctnnb1;slc22a4;slc16a14;fxyd5;gga3;scrn1;slc16a3;kcng1;uqcrh;heph;synj2bp;fa2h;snx25;cnnm2;rtp4;scnn1b;camk2g;stx16;best4;kcnk1;scarf1;cacna2d3;slc6a17;kcnq1;asna1;trappc6a;abcg8;slc30a10;nedd4l;tnp2;set;scfd1;best1;rab8a;slc9a8;slc22a14;rhob;slc39a6;cep57;hspd1;cog2;snx14;cog5;chrne;kif1c;cd74;spaca3;atp11b;kcnj12;rbp7;cnga3;trappc1;slc25a17;atp6v1g3;snx5;tnpo3;slc16a13;tspan18;rab3ip;cacna1f;fxyd1;slc35f2;stxbp1;atg4a;mapk8ip1;lman1;tubb6;csn2;ca2;cacnb3;alb;slc30a1;stat1;aqp11;col8a2;c1qtnf2;napg;lmbr1l;rab9b;atp2b3;slc6a5;kcna5;gosr1 |
| response to chemical stimulus; | 73#589 | 1.3913E-07 | ccr8;cxcl16;ogt;ephx1;cfb;asz1;ccr7;brs3;lats2;tmod1;hamp;rtp4;cx3cl1;plaur;pparg;hsp90aa1;hadha;hspb1;abcg2;gab1;prkca;cxcl11;hsph1;asna1;il3;nedd4l;gpx4;ccl28;ccr2;vapb;hspd1;cxcl14;wnt2;slc22a18;ccrl1;oxsr1;chrna4;vegfa;cdh13;ern1;ugt1a9;cdkn2d;egr2;kcnj11;tgfb2;rad54l;azu1;thbd;hspa1l;enpp2;hspa4;krt1;prdx5;ndrg1;rtp3;sord;tshb;tgfb1;eif2ak2;mtf1;tlr2;mapk1;rbm14;trpv1;snx27;ghsr;kcnmb4;tgm7;adra1b;hcls1;creb3;ccr9;ugt1a6 |
| multicellular organismal development#system development#organ development#organ morphogenesis; | 51#362 | 1.40041E-07 | gnpat;nrp1;adora2b;gaa;hey2;vegfa;shroom2;foxc1;edn1;cdh13;gja1;amtn;ctnnb1;dscaml1;foxn1;tgfb2;prkra;crx;fgf1;kitlg;eln;ihh;klf1;ngfr;flt1;krt1;comp;fhl1;fgd1;spry1;prkci;six6;onecut2;tgfb1;msx1;mapk1;ablim1;fgf6;pou4f3;tinag;klf5;adra1b;runx1;rhob;cdx2;aplp1;gna13;serpinf1;rasa1;dlx5;ift88 |
| biological adhesion; | 106#960 | 1.57202E-07 | ccr8;pnn;dcbld1;fbln5;rom1;amtn;amica1;alcam;susd5;izumo1;ctnnb1;dscaml1;fxyd5;pcdh18;mybpc2;matn1;alox12;ncam2;kng1;negr1;scarb2;cx3cl1;icam5;comp;cldn17;sema4d;cadm3;mfap4;nrcam;inppl1;fez1;pvrl2;flrt2;taok2;scarf1;hapln3;bcl6;pcdhb5;adam10;dcbld2;tinag;pcdhb6;cldn14;ddr2;omg;ajap1;tgfbi;pcdh1;rhob;aplp1;cercam;pcdhb7;nlgn4x;rasa1;rs1;gp1ba;nrp1;pcdhga2;entpd1;cdh13;cxadr;cd2;dsc2;azu1;cldn2;parvb;mkln1;cldn1;atp2c1;spock1;nrxn1;cdh16;kitlg;cd34;hapln2;arhgdib;cd93;opcml;cdh12;tspan2;cpxm1;mcam;olr1;abl2;islr;ctnna1;flrt3;atp2a2;adam12;igsf1;mag;arhgdia;sox9;col8a2;wisp1;ctnna3;cdk6;pkp3;pcdhb11;cd164;cldn20;mia;alx1;snai1;mfge8;thbs4 |
| cell adhesion; | 106#960 | 1.57202E-07 | ccr8;pnn;dcbld1;fbln5;rom1;amtn;amica1;alcam;susd5;izumo1;ctnnb1;dscaml1;fxyd5;pcdh18;mybpc2;matn1;alox12;ncam2;kng1;negr1;scarb2;cx3cl1;icam5;comp;cldn17;sema4d;cadm3;mfap4;nrcam;inppl1;fez1;pvrl2;flrt2;taok2;scarf1;hapln3;bcl6;pcdhb5;adam10;dcbld2;tinag;pcdhb6;cldn14;ddr2;omg;ajap1;tgfbi;pcdh1;rhob;aplp1;cercam;pcdhb7;nlgn4x;rasa1;rs1;gp1ba;nrp1;pcdhga2;entpd1;cdh13;cxadr;cd2;dsc2;azu1;cldn2;parvb;mkln1;cldn1;atp2c1;spock1;nrxn1;cdh16;kitlg;cd34;hapln2;arhgdib;cd93;opcml;cdh12;tspan2;cpxm1;mcam;olr1;abl2;islr;ctnna1;flrt3;atp2a2;adam12;igsf1;mag;arhgdia;sox9;col8a2;wisp1;ctnna3;cdk6;pkp3;pcdhb11;cd164;cldn20;mia;alx1;snai1;mfge8;thbs4 |
| multicellular organismal development#system development#organ development#tissue development; | 45#305 | 1.60679E-07 | sectm1;pth2r;krt13;minpp1;foxc1;edn1;gja1;amtn;ikzf1;ctnnb1;foxn1;keap1;matn1;tgfb2;sfn;elf3;il20;ikzf3;krt5;tfip11;sox15;atp2c1;plod1;ugcg;ihh;ngfr;krt1;btk;atp2a2;msx1;tgfb1;pou3f2;ostf1;sox9;ghsr;sparc;cdk6;alx1;snai1;klk7;maff;tgm5;eda2r;dlx5;krt9 |
| M phase; | 45#306 | 1.84107E-07 | kpna2;cd28;aurkaip1;npm2;shc1;anapc7;hus1;papd5;ccng1;bub3;dmc1;xrcc2;ran;rad54l;lats2;hectd3;ndc80;rcc1;pbrm1;mad2l2;dctn1;nde1;cdc16;akap8;nusap1;ncaph;tgfb1;rad51;brsk1;hells;cdc27;dusp13;sugt1;mns1;coro1a;ilf3;stag1;bub1b;sycp3;aurkb;pds5a;sac3d1;sgol1;ncapg;rad1 |
| wound healing; | 25#131 | 2.82251E-07 | serpinc1;lman1;tgfb1;procr;tfpi2;apoh;entpd1;gja1;tgfb2;f3;gna12;thbd;dcbld2;kng1;sox15;pabpc4;scnn1b;cd93;c4bpb;krt1;plaur;gp6;gna13;f7;gp1ba |
| neurological system process#transmission of nerve impulse#synaptic transmission; | 43#290 | 2.84289E-07 | chrne;maoa;strn4;chrna4;edn1;lin7a;galr1;ctnnb1;asz1;mbp;egr2;qprt;tgfb2;grm8;aldh5a1;drd3;th;pnoc;nr4a2;uts2;nras;gabrb2;mapk1;snph;pcdhb5;slc6a2;pxk;pmch;kcnmb4;chrna5;pcdhb11;pcdhb6;camk4;sncb;gch1;kcnq2;scn1b;mtnr1b;slc6a5;doc2a;dbh;wnt2;plp1 |
| cellular localization; | 119#1126 | 3.56667E-07 | stx10;snx15;chml;ankrd27;kif9;lin7a;pdia2;nrbp1;gga3;ran;scrn1;rapgef4;ykt6;arfip1;synj2bp;uso1;rtp4;scamp2;gpaa1;stx16;vps16;nup35;sergef;nfkbil1;krt20;srp72;gle1;hsp90aa1;nxf1;rpgr;ctsa;stx7;slc25a10;taok2;tomm22;sec61a2;pram1;snph;bcl6;senp2;pex16;tlk1;ramp1;sec61a1;sqstm1;traf7;ap1s3;vps26b;tnp2;hook3;set;scfd1;ap1g1;tmem48;camk4;vps41;ap3d1;rhob;kifc3;ywhab;cep57;rae1;hspd1;tnks;doc2a;cog2;arfgef2;cog5;kif1c;kpna2;shroom2;cdh13;cd74;copg2;cd2;kpna6;stx4;ap3s1;xpo6;trappc1;pcna;kif3b;atp2c1;slc25a17;nlrp2;kpna3;gnptg;srp54;kif3a;prkci;stxbp1;atg4a;ddx19b;rtp3;nupl2;nusap1;lman1;malt1;tgfb1;tubb6;kif18a;cdc37;vps33a;klhl2;alb;stat1;bace2;exoc3;dync1i1;map4k2;optn;napg;sec24b;vps26a;kcnmb4;rab9b;pex12;gosr1;gsk3b |
| regulation of nucleobase, nucleoside, nucleotide and nucleic acid metabolic process; | 318#3713 | 6.88208E-07 | foxp4;ikzf1;sap30bp;tigd3;hoxd1;tshz3;znf558;znf566;znf33b;znf548;nr4a2;pparg;znf414;sumo1;lcor;klf11;cdk9;vps72;nr5a1;hif1an;sqstm1;traf7;neurod6;gmeb2;chd3;glis2;ets2;vsx1;cdx2;fubp1;znf41;rasa1;sirt3;zbtb33;kpna2;smad6;chaf1a;edn1;ikzf4;myog;znf664;ern1;keap1;runx1t1;med20;nfatc2;prdm14;zfp2;cnbp;zxdc;polr3c;dlx2;hmgn2;foxj3;zfp42;ccnt2;atf3;msx1;pou3f2;rbm14;rdbp;zbtb25;dyrk1b;bcl11b;znf214;sox9;utf1;pou4f3;rab10;rab37;nr4a1;vgll4;supt4h1;pou2f1;alx1;sirt1;clock;znf605;rbl2;twist1;gtf2e1;hey2;ddx54;elk1;znf546;cnot6;elf5;irf5;ran;med23;vax1;znf197;trib3;klf12;myd88;hdac10;hoxa3;tcf23;fubp3;zbtb45;zbtb32;bcl6;gsc;tlx3;znf175;usf1;atf7;stat3;sp1;grip1;zfp1;taf4;maff;sohlh2;brms1l;znf501;znf710;cbfa2t2;dlx5;junb;mlxipl;foxc1;ripk2;eid2;tfe3;hoxd8;med18;ezh2;slc30a9;meis3;ppie;ing3;bclaf1;yap1;ihh;whsc2;nr5a2;taf6;cux1;zbtb16;hoxc9;rnf14;znf768;six6;polh;zscan29;irx3;sfpq;mtf1;eif2ak2;znf581;hdx;znf771;sp5;hells;prrx1;zbtb12;srpk1;hoxc12;klf15;hcls1;scml1;creb3;rarb;plagl2;rnf2;ovol2;runx3;pax8;brf2;habp4;lcorl;meox2;ssrp1;eya3;pogk;foxn1;znf524;grm8;dnm2;ikzf3;cdca7;supt16h;sox15;ripply1;zscan21;klf1;hmgb4;mkl1;znf787;tox3;esrrg;nr0b2;lancl2;znf446;rcor2;rfx5;nfatc2ip;tlk1;znf786;scmh1;tox;znf623;znf688;cited2;zbtb20;tnp1;dek;duxa;prdm12;trim24;gabpa;zfx;tbx10;med8;uimc1;sox6;sp2;pcna;nlk;sirt5;pbrm1;znfx1;pknox1;znf496;nanog;tgfb1;rad51;cda;acvr2b;elk4;phtf1;tfap2c;pdcd4;ncoa2;drgx;ilf3;prpf6;hif3a;znf24;tceal8;snip1;cnot4;pnn;tox4;mixl1;hus1;eya4;elavl2;ctnnb1;polr2f;znf354a;rax;znf451;abt1;grhl2;l3mbtl3;scml2;hdac9;ilf2;ctcf;tcea3;hoxb3;ppargc1b;pcgf2;npas1;dedd;pias1;set;tbpl2;klf5;rab8a;krba2;crebzf;tsc22d4;znf689;crem;casz1;terf2;mysm1;sirt4;tead1;znf131;sim2;elf1;egr2;sox5;prdm4;gabpb2;elf3;znf398;crx;pcgf1;dbx2;atf2;znf454;nr6a1;snd1;med24;gtf2a1;arid5a;onecut2;scap;gzf1;tspyl2;igsf1;creg1;stat1;rfx3;nab1;psmc5;epc2;zbtb38;runx1;dlx3;mecp2;cdt1;eda2r |
| mitotic cell cycle; | 46#326 | 7.31037E-07 | kpna2;cd28;aurkaip1;npm2;shc1;foxc1;anapc7;hus1;papd5;ccng1;cdkn2d;bub3;cdkn3;ran;dnm2;lats2;hectd3;ndc80;rcc1;pbrm1;cdk2ap1;mad2l2;dctn1;nde1;cdc16;akap8;nusap1;ncaph;tgfb1;bcl6;brsk1;hells;cdc27;sugt1;cdk6;coro1a;stag1;bub1b;sycp3;aurkb;cdt1;pds5a;cdk10;sac3d1;sgol1;ncapg |
| sensory perception of smell; | 3#540 | -9.20284E-07 | grm8;gnal;b3gnt2 |
| stress-activated protein kinase signaling pathway; | 19#68 | 9.20284E-07 | adora2b;gab1;taok2;taok3;map2k7;map4k3;il3;dbnl;cdc42ep5;map3k9;map4k2;dusp10;arhgef6;map3k4;myd88;sh2d3c;eda2r;mapk8ip1;map3k2 |
| transcription, DNA-dependent; | 296#3439 | 1.37748E-06 | foxp4;ikzf1;sap30bp;hoxd1;tshz3;znf558;znf566;znf33b;znf548;nr4a2;pparg;znf414;lcor;klf11;cdk9;vps72;nr5a1;hif1an;sqstm1;traf7;neurod6;gmeb2;chd3;glis2;ets2;vsx1;cdx2;fubp1;znf41;sirt3;zbtb33;smad6;chaf1a;ikzf4;myog;znf664;ern1;keap1;runx1t1;med20;nfatc2;prdm14;zfp2;cnbp;mki67ip;zxdc;polr3c;dlx2;hmgn2;foxj3;zfp42;ccnt2;atf3;msx1;pou3f2;rbm14;rdbp;zbtb25;dyrk1b;bcl11b;znf214;sox9;utf1;pou4f3;rab10;rab37;nr4a1;vgll4;supt4h1;pou2f1;alx1;polr2d;sirt1;clock;znf605;rbl2;twist1;gtf2e1;hey2;ddx54;elk1;znf546;cnot6;elf5;irf5;ran;med23;vax1;znf197;trib3;klf12;myd88;hdac10;hoxa3;fubp3;zbtb45;zbtb32;bcl6;gsc;tlx3;znf175;usf1;atf7;stat3;sp1;grip1;zfp1;taf4;cops2;maff;brms1l;znf501;znf710;cbfa2t2;dlx5;junb;mlxipl;foxc1;tfe3;hoxd8;med18;ezh2;slc30a9;meis3;ing3;bclaf1;yap1;ihh;whsc2;nr5a2;taf6;cux1;zbtb16;hoxc9;rnf14;znf768;six6;zscan29;irx3;eif2ak2;sfpq;mtf1;znf581;hdx;znf771;sp5;hells;prrx1;zbtb12;hoxc12;klf15;hcls1;scml1;creb3;rarb;plagl2;rnf2;ovol2;pax8;runx3;brf2;habp4;lcorl;meox2;ssrp1;eya3;pogk;foxn1;znf524;ikzf3;cdca7;supt16h;sox15;ripply1;zscan21;klf1;hmgb4;mkl1;znf787;tox3;esrrg;nr0b2;znf446;rcor2;rfx5;nfatc2ip;znf786;scmh1;tox;znf623;znf688;cited2;zbtb20;tnp1;duxa;dek;prdm12;trim24;gabpa;zfx;med8;uimc1;tbx10;sox6;sp2;nlk;sirt5;pbrm1;znfx1;pknox1;znf496;nanog;tgfb1;trip13;elk4;phtf1;tfap2c;ncoa2;drgx;ilf3;prpf6;adrm1;hif3a;znf24;tceal8;snip1;cnot4;pnn;tox4;mixl1;eya4;elavl2;ctnnb1;polr2f;znf354a;rax;znf451;abt1;grhl2;scml2;hdac9;ilf2;ctcf;tcea3;hoxb3;ppargc1b;pcgf2;npas1;dedd;pias1;tbpl2;klf5;rab8a;krba2;crebzf;tsc22d4;znf689;crem;casz1;mysm1;sirt4;tead1;znf131;sim2;elf1;egr2;sox5;prdm4;gabpb2;elf3;znf398;crx;pcgf1;dbx2;atf2;znf454;nr6a1;snd1;med24;gtf2a1;arid5a;scap;onecut2;gzf1;tspyl2;creg1;stat1;rfx3;nab1;psmc5;epc2;trove2;zbtb38;runx1;dlx3;mecp2 |
| organic acid metabolic process; | 89#798 | 1.52772E-06 | bhmt;acot9;acaca;ears2;prkag2;alox12;aldh5a1;slc27a2;tst;elovl7;tyr;bcat2;arg1;fa2h;lep;hadha;pdxdc1;ccnd3;acox2;wars;slc25a10;sms;uroc1;prkca;pepd;afmid;ssu72;acoxl;mthfd1l;farsa;cryl1;il3;acsl3;sgpl1;sars;ddah1;hadh;agmat;hibadh;psph;socs1;gnpat;acsm5;cd74;slc7a7;acadl;tfe3;gfpt1;cbs;kynu;il20;scd;th;aloxe3;plod1;me2;znfx1;fbp2;agxt2l1;acly;qdpr;mars2;ptges;prkaa1;wars2;pycr1;hars;aco1;ghsr;hacl1;c1qtnf2;aldh1l1;ugdh;cad;slc27a4;hcls1;gch1;nars;slc23a2;gfpt2;gmps;pts;acadsb;dbh;bckdhb;fasn;clcf1;prkaa2;coro2a |
| RNA biosynthetic process; | 296#3444 | 1.57282E-06 | foxp4;ikzf1;sap30bp;hoxd1;tshz3;znf558;znf566;znf33b;znf548;nr4a2;pparg;znf414;lcor;klf11;cdk9;vps72;nr5a1;hif1an;sqstm1;traf7;neurod6;gmeb2;chd3;glis2;ets2;vsx1;cdx2;fubp1;znf41;sirt3;zbtb33;smad6;chaf1a;ikzf4;myog;znf664;ern1;keap1;runx1t1;med20;nfatc2;prdm14;zfp2;cnbp;mki67ip;zxdc;polr3c;dlx2;hmgn2;foxj3;zfp42;ccnt2;atf3;msx1;pou3f2;rbm14;rdbp;zbtb25;dyrk1b;bcl11b;znf214;sox9;utf1;pou4f3;rab10;rab37;nr4a1;vgll4;supt4h1;pou2f1;alx1;polr2d;sirt1;clock;znf605;rbl2;twist1;gtf2e1;hey2;ddx54;elk1;znf546;cnot6;elf5;irf5;ran;med23;vax1;znf197;trib3;klf12;myd88;hdac10;hoxa3;fubp3;zbtb45;zbtb32;bcl6;gsc;tlx3;znf175;usf1;atf7;stat3;sp1;grip1;zfp1;taf4;cops2;maff;brms1l;znf501;znf710;cbfa2t2;dlx5;junb;mlxipl;foxc1;tfe3;hoxd8;med18;ezh2;slc30a9;meis3;ing3;bclaf1;yap1;ihh;whsc2;nr5a2;taf6;cux1;zbtb16;hoxc9;rnf14;znf768;six6;zscan29;irx3;eif2ak2;sfpq;mtf1;znf581;hdx;znf771;sp5;hells;prrx1;zbtb12;hoxc12;klf15;hcls1;scml1;creb3;rarb;plagl2;rnf2;ovol2;pax8;runx3;brf2;habp4;lcorl;meox2;ssrp1;eya3;pogk;foxn1;znf524;ikzf3;cdca7;supt16h;sox15;ripply1;zscan21;klf1;hmgb4;mkl1;znf787;tox3;esrrg;nr0b2;znf446;rcor2;rfx5;nfatc2ip;znf786;scmh1;tox;znf623;znf688;cited2;zbtb20;tnp1;duxa;dek;prdm12;trim24;gabpa;zfx;med8;uimc1;tbx10;sox6;sp2;nlk;sirt5;pbrm1;znfx1;pknox1;znf496;nanog;tgfb1;trip13;elk4;phtf1;tfap2c;ncoa2;drgx;ilf3;prpf6;adrm1;hif3a;znf24;tceal8;snip1;cnot4;pnn;tox4;mixl1;eya4;elavl2;ctnnb1;polr2f;znf354a;rax;znf451;abt1;grhl2;scml2;hdac9;ilf2;ctcf;tcea3;hoxb3;ppargc1b;pcgf2;npas1;dedd;pias1;tbpl2;klf5;rab8a;krba2;crebzf;tsc22d4;znf689;crem;casz1;mysm1;sirt4;tead1;znf131;sim2;elf1;egr2;sox5;prdm4;gabpb2;elf3;znf398;crx;pcgf1;dbx2;atf2;znf454;nr6a1;snd1;med24;gtf2a1;arid5a;scap;onecut2;gzf1;tspyl2;creg1;stat1;rfx3;nab1;psmc5;epc2;trove2;zbtb38;runx1;dlx3;mecp2 |
| membrane organization and biogenesis; | 41#284 | 1.75062E-06 | cd14;cdh13;cxcl16;spaca3;atg7;actn4;izumo1;cd2;azu1;dnm2;synj2bp;ldlrap1;uso1;clcn5;serpina5;dnm3;cd93;lrp10;elmo1;asgr2;prkci;nrcam;dnm1l;pacsin1;tgfb1;adam12;scarf1;necap1;pld2;pex16;dbnl;picalm;nedd4l;lrp6;ap1s3;napg;lmbr1l;ap1g1;rabep2;tfrc;aplp1 |
| glycoprotein biosynthetic process; | 30#182 | 1.81463E-06 | tspan8;man1a1;gcnt4;ogt;b3gnt4;pigb;bace2;il17a;sdf2;b3gnt2;st3gal3;st6galnac4;st3gal1;fut1;alg3;a4gnt;b3galt2;tnip1;b3gnt5;galnt6;galnt9;fut3;b3galt1;gnptg;mgat4a;st3gal4;st6gal1;cog2;st8sia5;b3galt5 |
| GO:0045944,positive regulation of transcription from RNA polymerase II promoter; | 25#139 | 1.81463E-06 | rbm14;elk1;foxc1;ppargc1b;myog;ctnnb1;sox9;elf1;utf1;sqstm1;nr4a2;sox15;crx;sp1;ncoa2;myd88;zbtb38;supt4h1;runx1;prpf6;yap1;ihh;dlx2;plagl2;clock |
| gene expression#regulation of gene expression; | 324#3833 | 1.90492E-06 | foxp4;ikzf1;sap30bp;tigd3;hoxd1;tshz3;znf558;znf566;znf33b;znf548;nr4a2;pparg;znf414;sumo1;lcor;klf11;cdk9;vps72;nr5a1;hif1an;sqstm1;traf7;neurod6;gmeb2;chd3;glis2;pstk;ets2;vsx1;cdx2;fubp1;aplp1;znf41;sirt3;eif3f;zbtb33;smad6;chaf1a;ikzf4;myog;znf664;ern1;keap1;runx1t1;med20;nfatc2;prdm14;zfp2;cnbp;eif1b;zxdc;polr3c;dlx2;hmgn2;foxj3;zfp42;ccnt2;atf3;msx1;pou3f2;rbm14;rdbp;zbtb25;dyrk1b;bcl11b;znf214;sox9;bace2;utf1;pou4f3;eif4g2;rab10;rab37;nr4a1;vgll4;supt4h1;pou2f1;alx1;sirt1;clock;znf605;rbl2;twist1;gtf2e1;diras3;hey2;ddx54;elk1;znf546;cnot6;elf5;irf5;ran;med23;vax1;znf197;trib3;klf12;myd88;hdac10;hoxa3;tcf23;fubp3;zbtb45;zbtb32;bcl6;gsc;tlx3;znf175;usf1;atf7;stat3;sp1;grip1;zfp1;taf4;maff;sohlh2;brms1l;znf501;znf710;cbfa2t2;dlx5;junb;mlxipl;foxc1;ripk2;eid2;tfe3;hoxd8;azu1;med18;ezh2;slc30a9;meis3;ppie;ing3;bclaf1;yap1;ihh;whsc2;nr5a2;taf6;igf2bp3;cux1;zbtb16;hoxc9;rnf14;znf768;six6;zscan29;irx3;sfpq;mtf1;eif2ak2;znf581;hdx;znf771;sp5;hells;prrx1;zbtb12;cdk6;hoxc12;klf15;pum2;hcls1;scml1;creb3;rarb;plagl2;rnf2;ovol2;runx3;pax8;brf2;habp4;lcorl;meox2;ssrp1;eya3;pogk;foxn1;znf524;dnm2;ikzf3;cdca7;supt16h;sox15;ripply1;zscan21;klf1;hmgb4;mkl1;znf787;tox3;esrrg;nr0b2;lancl2;znf446;rcor2;rfx5;nfatc2ip;znf786;scmh1;tox;znf623;znf688;samd4a;cited2;zbtb20;tnp1;eif4h;dek;duxa;prdm12;trim24;gabpa;zfx;tbx10;med8;uimc1;sox6;sp2;nlk;sirt5;pbrm1;znfx1;pknox1;znf496;nanog;tgfb1;acvr2b;elk4;ghsr;phtf1;tfap2c;pdcd4;ncoa2;drgx;ilf3;prpf6;hif3a;znf24;tceal8;snip1;eif5;cnot4;pnn;tox4;mixl1;eya4;elavl2;ctnnb1;polr2f;znf354a;rax;abt1;znf451;grhl2;l3mbtl3;scml2;hdac9;ilf2;ctcf;tcea3;hspb1;hoxb3;ppargc1b;pcgf2;npas1;dedd;pias1;tbpl2;klf5;rab8a;krba2;crebzf;tsc22d4;znf689;crem;casz1;terf2;mysm1;cd28;sirt4;tead1;znf131;sim2;elf1;egr2;sox5;prdm4;gabpb2;elf3;znf398;crx;pcgf1;dbx2;atf2;znf454;nr6a1;snd1;med24;eif3e;gtf2a1;arid5a;onecut2;scap;gzf1;tspyl2;igsf1;creg1;stat1;rfx3;nab1;psmc5;epc2;eif3i;zbtb38;runx1;dlx3;mecp2;eda2r |
| regulation of transcription, DNA-dependent; | 289#3358 | 1.95694E-06 | foxp4;ikzf1;sap30bp;hoxd1;tshz3;znf558;znf566;znf33b;znf548;nr4a2;pparg;znf414;lcor;klf11;cdk9;vps72;nr5a1;hif1an;sqstm1;traf7;neurod6;gmeb2;chd3;glis2;ets2;vsx1;cdx2;fubp1;znf41;sirt3;zbtb33;smad6;chaf1a;ikzf4;myog;znf664;ern1;keap1;runx1t1;med20;nfatc2;prdm14;zfp2;cnbp;zxdc;polr3c;dlx2;hmgn2;foxj3;zfp42;ccnt2;atf3;msx1;pou3f2;rbm14;rdbp;zbtb25;dyrk1b;bcl11b;znf214;sox9;utf1;pou4f3;rab10;rab37;nr4a1;vgll4;supt4h1;pou2f1;alx1;sirt1;clock;znf605;rbl2;twist1;gtf2e1;hey2;ddx54;elk1;znf546;cnot6;elf5;irf5;ran;med23;vax1;znf197;trib3;klf12;myd88;hdac10;hoxa3;fubp3;zbtb45;zbtb32;bcl6;gsc;tlx3;znf175;usf1;atf7;stat3;sp1;grip1;zfp1;taf4;maff;brms1l;znf501;znf710;cbfa2t2;dlx5;junb;mlxipl;foxc1;tfe3;hoxd8;med18;ezh2;slc30a9;meis3;ing3;bclaf1;yap1;ihh;whsc2;nr5a2;taf6;cux1;zbtb16;hoxc9;rnf14;znf768;six6;zscan29;irx3;eif2ak2;sfpq;mtf1;znf581;hdx;znf771;sp5;hells;prrx1;zbtb12;hoxc12;klf15;hcls1;scml1;creb3;rarb;plagl2;rnf2;ovol2;pax8;runx3;brf2;habp4;lcorl;meox2;ssrp1;eya3;pogk;foxn1;znf524;ikzf3;cdca7;supt16h;sox15;ripply1;zscan21;klf1;hmgb4;mkl1;znf787;tox3;esrrg;nr0b2;znf446;rcor2;rfx5;nfatc2ip;znf786;scmh1;tox;znf623;znf688;cited2;zbtb20;tnp1;duxa;dek;prdm12;trim24;gabpa;zfx;med8;uimc1;tbx10;sox6;sp2;nlk;sirt5;pbrm1;znfx1;pknox1;znf496;nanog;tgfb1;elk4;phtf1;tfap2c;ncoa2;drgx;ilf3;prpf6;hif3a;znf24;tceal8;snip1;cnot4;pnn;tox4;mixl1;eya4;elavl2;ctnnb1;znf354a;rax;znf451;abt1;grhl2;scml2;hdac9;ilf2;ctcf;tcea3;hoxb3;ppargc1b;pcgf2;npas1;dedd;pias1;tbpl2;klf5;rab8a;krba2;crebzf;tsc22d4;crem;znf689;casz1;mysm1;sirt4;tead1;znf131;sim2;elf1;egr2;sox5;prdm4;gabpb2;elf3;znf398;crx;pcgf1;dbx2;atf2;znf454;nr6a1;snd1;med24;gtf2a1;arid5a;scap;onecut2;gzf1;tspyl2;creg1;stat1;rfx3;nab1;psmc5;epc2;zbtb38;runx1;dlx3;mecp2 |
| growth#regulation of growth; | 32#201 | 2.14497E-06 | tgfb1;net1;tspyl2;pou3f2;taok2;egln2;shc1;foxc1;bcl6;creg1;cdkn2aip;il3;cda;dio3;wisp1;cdkn2d;ghsr;tgfb2;alox12;bnipl;dcbld2;camk2d;ing3;htra1;crim1;esm1;socs1;brms1l;ing1;igfbp6;nrg3;ebag9 |
| JNK cascade; | 18#65 | 2.22812E-06 | adora2b;gab1;taok2;taok3;map4k3;il3;dbnl;map3k9;cdc42ep5;map4k2;dusp10;arhgef6;map3k4;myd88;sh2d3c;eda2r;mapk8ip1;map3k2 |
| post-translational protein modification; | 204#2235 | 2.42593E-06 | tpst1;ttbk1;ptk2;hunk;fbxo10;usp30;asz1;spcs2;myo3a;map3k9;nrbp1;asb2;mark2;rapgef4;dusp10;camk2d;prkra;map3k7;map3k4;gpaa1;cdk2ap1;sh3rf2;adprh;cdc16;sumo1;glyctk;cdk9;taok2;fbxl17;tec;cdc42bpb;tlk1;il3;traf7;vrk1;pdk2;pim1;arih1;ptpn18;phkg2;ddr2;rbbp6;rnf139;prmt2;ptpn21;tgm5;ulk3;tnks;csnk1g3;senp1;ccnb1ip1;herc6;sirt3;pigu;akt2;atg7;ern1;rbck1;uchl5;med8;mtmr7;mex3b;nlk;npr2;sirt5;syvn1;ppp1cb;usp32;prkci;pak1;tp53rk;abl2;fbxl4;map3k2;axl;tgfb1;rbm14;mapk1;dyrk1b;usp15;mid1;fbxl8;dusp22;map2k7;lcmt1;acvr2b;bace2;klhl15;ppm1k;ppm1l;map4k2;mark1;cdkl2;gucy2c;dusp13;aurkc;fbxo38;tgm7;pcsk7;opn1sw;sirt1;ppp1cc;hectd2;stk39;clcf1;prkaa2;usp13;pja2;gsk3b;cnot4;bhmt;rnf41;nek8;anapc7;traf6;hus1;ero1lb;epha3;tnfaip3;lrrk1;twf1;lats2;lrrc41;trib3;uhrf2;cdc14b;fbxl22;camk2g;hdac9;flt1;btk;hdac10;usp2;mapkapk5;phkg1;ccnd3;prkca;ncln;cpa4;taok3;senp2;ppm1d;senp3;adam10;nedd4l;csnk1a1;sugt1;pias1;set;pak3;camk4;socs1;usp33;cdk10;usp11;snrk;wnt2;lrrc29;clk3;ddb1;ube2m;oxsr1;sirt4;fbxo45;araf;ripk2;pigs;fbxo9;wee2;fbxl13;il20;hectd3;itk;gan;epha1;ihh;dmpk;rnf14;zbtb16;srms;atg4a;pskh1;fbxw8;malt1;jak1;prkaa1;eif2ak2;abi2;stat1;atg12;fbxo47;map4k3;brsk1;srpk1;cdk6;nuak1;hcls1;bub1b;ero1l;aurkb;pja1;rnf2;senp8;fbxl6 |
| mitosis; | 36#239 | 2.46057E-06 | cd28;aurkaip1;npm2;shc1;anapc7;hus1;papd5;ccng1;bub3;ran;lats2;hectd3;ndc80;rcc1;pbrm1;mad2l2;dctn1;nde1;cdc16;akap8;nusap1;ncaph;tgfb1;brsk1;cdc27;hells;sugt1;coro1a;bub1b;stag1;sycp3;aurkb;pds5a;sgol1;sac3d1;ncapg |
| carboxylic acid metabolic process; | 88#795 | 2.51686E-06 | bhmt;acot9;acaca;ears2;prkag2;alox12;aldh5a1;slc27a2;elovl7;tyr;bcat2;arg1;fa2h;lep;hadha;pdxdc1;ccnd3;acox2;wars;slc25a10;sms;uroc1;prkca;pepd;afmid;ssu72;acoxl;mthfd1l;farsa;cryl1;il3;acsl3;sgpl1;sars;ddah1;hadh;agmat;hibadh;psph;socs1;gnpat;acsm5;cd74;slc7a7;acadl;tfe3;gfpt1;cbs;kynu;il20;scd;th;aloxe3;plod1;me2;znfx1;fbp2;agxt2l1;acly;qdpr;mars2;ptges;prkaa1;wars2;pycr1;hars;aco1;ghsr;hacl1;c1qtnf2;ugdh;aldh1l1;cad;slc27a4;hcls1;gch1;nars;slc23a2;gfpt2;gmps;pts;acadsb;dbh;bckdhb;fasn;clcf1;prkaa2;coro2a |
| negative regulation of transcription, DNA-dependent; | 32#203 | 2.90585E-06 | sirt4;sim2;ctnnb1;sox15;klf12;ripply1;sirt5;nr6a1;ctcf;cux1;zbtb16;arid5a;lcor;klf11;gzf1;msx1;vps72;zbtb32;nr0b2;bcl6;npas1;sox9;hells;stat3;ncoa2;supt4h1;ilf3;mecp2;tnp1;sirt1;twist1;sirt3 |
| sensory perception of chemical stimulus; | 6#584 | -3.00149E-06 | grm8;rtp4;gnal;b3gnt2;scnn1b;rtp3 |
| regulation of body fluid levels; | 23#125 | 3.02774E-06 | serpinc1;lman1;procr;tfpi2;apoh;entpd1;edn1;slc22a4;nedd4l;f3;gna12;thbd;kng1;pabpc4;cd93;c4bpb;krt1;plaur;gp6;gna13;f7;agtr1;gp1ba |
| neurological system process#transmission of nerve impulse; | 45#330 | 3.60376E-06 | chrne;maoa;strn4;chrna4;edn1;lin7a;galr1;ctnnb1;asz1;mbp;egr2;qprt;tgfb2;grm8;aldh5a1;drd3;th;pnoc;nr4a2;uts2;tgfb1;nras;gabrb2;mapk1;snph;pcdhb5;slc6a2;pxk;pmch;kcnmb4;chrna5;pcdhb11;pcdhb6;camk4;sncb;gch1;kcnq2;scn1b;mtnr1b;slc6a5;doc2a;dbh;wnt2;hexa;plp1 |
| establishment of cellular localization; | 113#1098 | 3.79654E-06 | stx10;snx15;chml;ankrd27;kif9;lin7a;nrbp1;gga3;ran;scrn1;rapgef4;ykt6;arfip1;synj2bp;uso1;rtp4;scamp2;stx16;vps16;nup35;sergef;nfkbil1;krt20;srp72;gle1;hsp90aa1;nxf1;rpgr;ctsa;stx7;slc25a10;taok2;tomm22;sec61a2;pram1;snph;bcl6;senp2;pex16;tlk1;ramp1;sec61a1;sqstm1;traf7;ap1s3;vps26b;tnp2;set;scfd1;ap1g1;tmem48;camk4;vps41;ap3d1;rhob;kifc3;ywhab;cep57;rae1;hspd1;tnks;doc2a;cog2;arfgef2;cog5;kif1c;kpna2;shroom2;cd74;copg2;kpna6;stx4;ap3s1;xpo6;trappc1;pcna;atp2c1;kif3b;slc25a17;nlrp2;kpna3;gnptg;srp54;kif3a;prkci;stxbp1;atg4a;ddx19b;rtp3;nupl2;nusap1;lman1;malt1;tgfb1;tubb6;kif18a;cdc37;vps33a;klhl2;stat1;bace2;exoc3;dync1i1;map4k2;optn;napg;sec24b;vps26a;kcnmb4;rab9b;pex12;gosr1;gsk3b |
| cell migration; | 35#233 | 3.93309E-06 | nrp1;nck1;vegfa;shroom2;foxc1;cdh13;cxcl16;gja1;asz1;tgfb2;azu1;mdga1;clic4;cx3cl1;scnn1b;cd34;flt1;ngfr;dcc;nrcam;tgfb1;fez1;prkca;gab1;taok2;abi2;pou4f3;kiss1r;b3gnt2;tlx3;efnb3;gna13;cck;wnt2;thbs4 |
| membrane invagination; | 31#197 | 4.69643E-06 | pacsin1;tgfb1;cd14;scarf1;cdh13;cxcl16;pld2;spaca3;necap1;actn4;picalm;dbnl;nedd4l;ap1s3;lrp6;azu1;lmbr1l;dnm2;ap1g1;rabep2;synj2bp;ldlrap1;clcn5;dnm3;cd93;tfrc;lrp10;elmo1;aplp1;asgr2;dnm1l |
| endocytosis; | 31#197 | 4.69643E-06 | pacsin1;tgfb1;cd14;scarf1;cdh13;cxcl16;pld2;spaca3;necap1;actn4;picalm;dbnl;nedd4l;ap1s3;lrp6;azu1;lmbr1l;dnm2;ap1g1;rabep2;synj2bp;ldlrap1;clcn5;dnm3;cd93;tfrc;lrp10;elmo1;aplp1;asgr2;dnm1l |
| mRNA processing; | 39#273 | 4.80835E-06 | pnn;ppil3;dhx15;ern1;ppp4r2;apobec1;tfip11;dhx38;snrpe;grsf1;cstf1;hnrnpr;sf3b2;ppie;usp39;hnrnpl;hnrpll;rbm23;skiv2l2;rnps1;nhp2l1;sf3a2;syncrip;sfpq;slbp;zmat5;zcrb1;cstf2t;fip1l1;srpk1;cdc40;rnpc3;rbm17;prpf6;apobec2;aplp1;clp1;scnm1;crnkl1 |
| regulation of cell growth; | 27#162 | 5.45146E-06 | tgfb1;net1;tspyl2;taok2;egln2;shc1;bcl6;creg1;cdkn2aip;il3;cda;wisp1;cdkn2d;tgfb2;alox12;dcbld2;camk2d;ing3;htra1;crim1;esm1;socs1;brms1l;ing1;igfbp6;nrg3;ebag9 |
| M phase of mitotic cell cycle; | 36#245 | 5.47642E-06 | cd28;aurkaip1;npm2;shc1;anapc7;hus1;papd5;ccng1;bub3;ran;lats2;hectd3;ndc80;rcc1;pbrm1;mad2l2;dctn1;nde1;cdc16;akap8;nusap1;ncaph;tgfb1;brsk1;cdc27;hells;sugt1;coro1a;bub1b;stag1;sycp3;aurkb;pds5a;sgol1;sac3d1;ncapg |
| developmental process#regulation of developmental process; | 35#236 | 5.89751E-06 | cdc42ep4;ctnnb1;keap1;tgfb2;il20;crx;lep;ihh;ngfr;pparg;sema4d;zbtb16;fgd1;nrcam;nanog;ctnna1;tgfb1;pou3f2;mapk1;taok2;vps33a;bcl6;gas2;cyfip1;cdc42ep5;ghsr;tlx3;cdk6;runx1;iqcb1;maff;gna13;serpinf1;rasa1;dlx5 |
| ubiquitin cycle; | 65#549 | 6.67982E-06 | cnot4;rnf41;anapc7;traf6;fbxo10;usp30;asb2;tnfaip3;lrrc41;uhrf2;fbxl22;sh3rf2;usp2;cdc16;sumo1;fbxl17;senp2;senp3;traf7;nedd4l;sugt1;pias1;arih1;rbbp6;rnf139;socs1;usp33;usp11;senp1;herc6;ccnb1ip1;lrrc29;ddb1;ube2m;fbxo45;atg7;fbxo9;rbck1;uchl5;med8;fbxl13;hectd3;gan;syvn1;usp32;rnf14;zbtb16;fbxl4;atg4a;fbxw8;malt1;usp15;fbxl8;mid1;atg12;fbxo47;klhl15;fbxo38;rnf2;pja1;senp8;hectd2;pja2;usp13;fbxl6 |
| skeletal development; | 32#209 | 7.07776E-06 | pth2r;tgfb1;msx1;mmp9;minpp1;foxc1;edn1;amtn;papss1;ctnnb1;dscaml1;prelp;sox9;ostf1;prdx1;matn1;tgfb2;sparc;cdk6;prkra;tfip11;ets2;dlx3;snai1;alx1;ihh;comp;npr3;clec3b;hexa;twist1;dlx5 |
| Golgi vesicle transport; | 18#89 | 7.50314E-06 | stx10;lman1;kif1c;copg2;nrbp1;optn;napg;sec24b;ykt6;trappc1;rab9b;scamp2;stx16;gosr1;cog2;prkci;stx7;cog5 |
| cellular carbohydrate metabolic process; | 60#496 | 7.6916E-06 | uap1;gaa;sdha;foxc1;idh3a;pgam2;gfpt1;prkag2;brs3;chst12;fut1;sucla2;pgd;chst8;aoah;lep;bpgm;g6pd;me2;hpse;ppp1cb;fbp2;acly;tktl1;ipmk;phkg1;cmas;sord;slc25a10;chst14;dlst;galns;aco1;ldha;hk3;pdk2;c1qtnf2;ugdh;phkg2;gnpda1;adra1b;pdha1;hibadh;pfkfb2;ldhc;gns;chst1;lalba;ppp1cc;gfpt2;cercam;hgsnat;hyal2;ldhb;ogdh;hexa;pygl;gsk3b;phka2;pgk2 |
| intracellular protein transport; | 63#529 | 7.71819E-06 | stx10;snx15;chml;kpna2;cd74;copg2;kpna6;stx4;gga3;ap3s1;ran;xpo6;arfip1;pcna;synj2bp;uso1;rtp4;kpna3;stx16;vps16;nup35;gnptg;nfkbil1;srp54;srp72;gle1;prkci;atg4a;rpgr;ddx19b;rtp3;ctsa;stx7;nupl2;tgfb1;taok2;tomm22;cdc37;klhl2;sec61a2;bcl6;senp2;stat1;pex16;tlk1;ramp1;sec61a1;optn;ap1s3;napg;sec24b;ap1g1;tmem48;vps41;ap3d1;pex12;ywhab;cep57;hspd1;tnks;cog2;gosr1;gsk3b |
| positive regulation of transferase activity; | 25#147 | 8.38756E-06 | ccnd3;adora2b;malt1;gab1;dgkb;taok2;prkaa1;dgkz;shc1;edn1;traf6;ern1;traf7;dbnl;map3k9;serinc1;azu1;c1qtnf2;map3k7;map3k4;dgka;madd;agk;pak1;map3k2 |
| cell growth; | 31#201 | 8.41139E-06 | tgfb1;net1;tspyl2;taok2;egln2;rragc;shc1;bcl6;creg1;cdkn2aip;il3;cda;wisp1;cdkn2d;tgfb2;alox12;dcbld2;camk2d;adra1b;ing3;htra1;crim1;esm1;fgf20;socs1;brms1l;fhl1;ing1;igfbp6;nrg3;ebag9 |
| protein amino acid glycosylation; | 28#174 | 9.01455E-06 | tspan8;man1a1;gcnt4;ogt;b3gnt4;pigb;il17a;sdf2;b3gnt2;st3gal3;st6galnac4;st3gal1;fut1;alg3;a4gnt;b3galt2;b3gnt5;galnt6;galnt9;fut3;b3galt1;gnptg;mgat4a;st3gal4;st6gal1;cog2;st8sia5;b3galt5 |
| gene expression; | 435#5454 | 9.41293E-06 | foxp4;exosc10;ikzf1;ppil3;sap30bp;rbm3;tigd3;hoxd1;ears2;tshz3;znf558;znf566;znf33b;znf548;nr4a2;tfip11;pparg;znf414;sumo1;lcor;klf11;cdk9;sf3a2;vps72;nr5a1;hif1an;sqstm1;traf7;neurod6;gmeb2;pop5;chd3;glis2;pstk;ets2;pabpc4;vsx1;cdx2;fubp1;apobec2;aplp1;znf41;sirt3;eif3f;zbtb33;abcf1;rrp9;smad6;chaf1a;ikzf4;myog;znf664;ern1;keap1;runx1t1;med20;nfatc2;prdm14;zfp2;cnbp;eif1b;snrpe;cstf1;mki67ip;usp39;zxdc;rpl8;wdr36;hnrpll;mrpl44;polr3c;dlx2;hmgn2;foxj3;rbm23;mars2;zfp42;pwp1;ccnt2;atf3;msx1;pou3f2;rbm14;rdbp;zbtb25;dyrk1b;bcl11b;znf214;sox9;wars2;bace2;utf1;pou4f3;zcrb1;eif4g2;rab10;rab37;nr4a1;vgll4;supt4h1;pou2f1;alx1;polr2d;sirt1;mrps18b;clock;mrpl3;znf605;rbl2;twist1;gtf2e1;diras3;cdc73;hey2;ddx54;elk1;znf546;cnot6;elf5;mrpl10;irf5;ran;med23;vax1;znf197;trib3;klf12;myd88;hdac10;hoxa3;nfkbib;tcf23;fubp3;zbtb45;wars;zbtb32;nhp2l1;ints5;bcl6;gsc;mettl1;tlx3;znf175;usf1;ftsj2;atf7;stat3;sp1;grip1;zfp1;taf4;cops2;maff;sohlh2;brms1l;znf501;znf710;cbfa2t2;dlx5;junb;mlxipl;mrpl36;foxc1;ripk2;dus1l;eid2;tfe3;rpp38;hoxd8;azu1;slc30a9;ezh2;med18;znf346;meis3;sf3b2;ppie;rps8;ing3;bclaf1;tsfm;yap1;whsc2;ihh;mrps9;nr5a2;taf6;igf2bp3;cux1;zbtb16;rnf14;hoxc9;znf768;six6;zscan29;irx3;sfpq;mtf1;eif2ak2;slbp;znf581;hdx;hars;znf771;sp5;hells;prrx1;zbtb12;srpk1;cdk6;hoxc12;klf15;trpc5;pum2;hcls1;scml1;rbm17;creb3;trmu;rarb;plagl2;rnf2;dph1;ovol2;runx3;pax8;brf2;habp4;lcorl;meox2;ssrp1;mrpl30;mrps18c;utp6;eya3;ict1;pogk;foxn1;znf524;dnm2;ikzf3;cdca7;supt16h;sox15;ripply1;zscan21;klf1;hmgb4;znf787;mkl1;tox3;syncrip;esrrg;lancl2;nr0b2;znf446;rcor2;rfx5;nfatc2ip;znf786;scmh1;tox;znf623;sars;znf688;samd4a;cited2;adat1;zbtb20;tnp1;eif4h;crnkl1;dek;duxa;prdm12;eef1d;ppp4r2;trim24;gabpa;mrpl27;zfx;apobec1;tbx10;med8;uimc1;mrpl22;sox6;sp2;rbm5;nlk;sirt5;pbrm1;hnrnpl;znfx1;pknox1;eif1ax;znf496;skiv2l2;nanog;imp4;tgfb1;acvr2b;trip13;elk4;ghsr;rps29;phtf1;fip1l1;tfap2c;pdcd4;slc38a4;ncoa2;drgx;ilf3;prpf6;adrm1;hif3a;clp1;znf24;tceal8;snip1;eif5;cnot4;pnn;rragc;tox4;mixl1;eya4;dhx15;ctnnb1;elavl2;polr2f;znf354a;rax;abt1;znf451;grsf1;mrpl16;grhl2;rpp14;l3mbtl3;scml2;hdac9;ilf2;ctcf;tcea3;exosc7;rnps1;rps3;hspb1;rpp30;utp3;hoxb3;adat3;ppargc1b;zmat5;rps10;npas1;farsa;pcgf2;rplp1;ftsj1;mrpl37;dedd;eef1e1;pias1;tbpl2;klf5;cdc40;rnpc3;rab8a;krba2;crebzf;tnip1;trub1;tsc22d4;znf689;crem;terf2;casz1;mysm1;sirt4;cd28;tead1;znf131;sim2;elf1;egr2;sox5;prdm4;mrpl47;gabpb2;elf3;crx;dhx38;trub2;znf398;pcgf1;hnrnpr;dbx2;atf2;nr6a1;znf454;snd1;med24;rpl9;eif3e;gtf2a1;arid5a;onecut2;scap;gzf1;tspyl2;igsf1;rsl1d1;creg1;stat1;nsun2;rfx3;nab1;cstf2t;denr;psmc5;epc2;eif3i;trove2;zbtb38;runx1;dlx3;mecp2;nars;mrpl35;scnm1;adad1;eda2r |
| catabolic process; | 101#973 | 1.01206E-05 | exosc10;neu3;sdha;egln2;upf3a;ephx1;idh3a;usp30;pla2g12a;lysmd3;psmd14;psmc2;ddhd1;aldh5a1;pafah1b3;tst;bcat2;arg1;fut1;sucla2;uhrf2;bpgm;g6pd;usp2;dera;tktl1;exosc7;psma8;pnpla3;rnps1;pglyrp3;uroc1;dcp1a;flrt2;pepd;mmp9;afmid;dlst;liph;sqstm1;nedd4l;ldha;gpx4;sgpl1;isg20;arih1;psmb5;ddah1;gnpda1;agmat;pabpc4;hibadh;usp33;usp11;gaa;maoa;gja1;spaca3;lyz;pgam2;kynu;uchl5;tprkb;pgd;usp39;enpp2;prtn3;syvn1;usp32;acly;qdpr;proca1;ampd3;fbxl4;dcp2;upp1;usp15;dffa;pld2;cda;cln5;dio3;aco1;hk3;psmc5;aldh1l1;adra1b;pdha1;gch1;ldhc;gns;pts;hyal2;ldhb;ogdh;dbh;hexa;rnaseh1;bckdhb;usp13;pgk2 |
| mRNA metabolic process; | 45#342 | 1.25221E-05 | exosc10;pnn;upf3a;ppil3;dhx15;ern1;ppp4r2;pcbp3;apobec1;tfip11;dhx38;snrpe;grsf1;cstf1;hnrnpr;sf3b2;ppie;usp39;hnrnpl;hnrpll;rbm23;skiv2l2;rnps1;dcp2;dcp1a;nhp2l1;sf3a2;syncrip;sfpq;slbp;zmat5;zcrb1;cstf2t;fip1l1;srpk1;cdc40;rnpc3;pabpc4;rbm17;prpf6;apobec2;aplp1;clp1;scnm1;crnkl1 |
| transcription; | 316#3803 | 1.32446E-05 | foxp4;ikzf1;sap30bp;tigd3;hoxd1;tshz3;znf558;znf566;znf33b;znf548;nr4a2;pparg;znf414;sumo1;lcor;klf11;cdk9;vps72;nr5a1;hif1an;sqstm1;traf7;neurod6;gmeb2;chd3;glis2;ets2;vsx1;cdx2;fubp1;znf41;sirt3;zbtb33;smad6;chaf1a;ikzf4;myog;znf664;ern1;keap1;runx1t1;med20;nfatc2;prdm14;zfp2;cnbp;mki67ip;zxdc;polr3c;dlx2;hmgn2;foxj3;zfp42;pwp1;ccnt2;atf3;msx1;pou3f2;rbm14;rdbp;zbtb25;dyrk1b;bcl11b;znf214;sox9;utf1;pou4f3;rab10;rab37;nr4a1;vgll4;supt4h1;pou2f1;alx1;polr2d;sirt1;clock;znf605;rbl2;twist1;gtf2e1;cdc73;hey2;ddx54;elk1;znf546;cnot6;elf5;irf5;ran;med23;vax1;znf197;trib3;klf12;myd88;hdac10;hoxa3;nfkbib;tcf23;fubp3;zbtb45;zbtb32;bcl6;gsc;tlx3;znf175;usf1;atf7;stat3;sp1;grip1;zfp1;taf4;cops2;maff;sohlh2;brms1l;znf501;znf710;cbfa2t2;dlx5;junb;mlxipl;foxc1;ripk2;eid2;tfe3;hoxd8;med18;ezh2;slc30a9;meis3;ppie;ing3;bclaf1;yap1;ihh;whsc2;nr5a2;taf6;cux1;zbtb16;hoxc9;rnf14;znf768;six6;zscan29;irx3;sfpq;mtf1;eif2ak2;znf581;hdx;znf771;sp5;hells;prrx1;zbtb12;hoxc12;klf15;hcls1;scml1;creb3;rarb;plagl2;rnf2;ovol2;runx3;pax8;brf2;habp4;lcorl;meox2;ssrp1;eya3;pogk;foxn1;znf524;dnm2;ikzf3;cdca7;supt16h;sox15;ripply1;zscan21;klf1;hmgb4;mkl1;znf787;tox3;esrrg;nr0b2;lancl2;znf446;rcor2;rfx5;nfatc2ip;znf786;scmh1;tox;znf623;znf688;cited2;zbtb20;tnp1;duxa;dek;prdm12;trim24;gabpa;zfx;tbx10;med8;uimc1;sox6;sp2;nlk;sirt5;pbrm1;znfx1;pknox1;znf496;nanog;tgfb1;trip13;acvr2b;elk4;phtf1;tfap2c;pdcd4;ncoa2;drgx;ilf3;prpf6;adrm1;hif3a;znf24;tceal8;snip1;cnot4;pnn;rragc;tox4;mixl1;eya4;elavl2;ctnnb1;polr2f;znf354a;rax;znf451;abt1;grhl2;l3mbtl3;scml2;hdac9;ilf2;ctcf;tcea3;rnps1;hoxb3;ppargc1b;pcgf2;npas1;dedd;pias1;tbpl2;klf5;rab8a;krba2;crebzf;tsc22d4;znf689;crem;casz1;terf2;mysm1;sirt4;tead1;znf131;sim2;elf1;egr2;sox5;prdm4;gabpb2;elf3;znf398;crx;pcgf1;dbx2;atf2;znf454;nr6a1;snd1;med24;gtf2a1;arid5a;onecut2;scap;gzf1;tspyl2;igsf1;creg1;stat1;rfx3;nab1;psmc5;epc2;trove2;zbtb38;runx1;dlx3;mecp2;eda2r |
| positive regulation of protein kinase activity; | 24#141 | 1.3758E-05 | ccnd3;adora2b;malt1;gab1;dgkb;taok2;prkaa1;dgkz;shc1;edn1;traf6;ern1;traf7;dbnl;map3k9;azu1;c1qtnf2;map3k7;map3k4;dgka;madd;agk;pak1;map3k2 |
| regulation of transcription; | 305#3654 | 1.39778E-05 | foxp4;ikzf1;sap30bp;tigd3;hoxd1;tshz3;znf558;znf566;znf33b;znf548;nr4a2;pparg;znf414;sumo1;lcor;klf11;cdk9;vps72;nr5a1;hif1an;sqstm1;traf7;neurod6;gmeb2;chd3;glis2;ets2;vsx1;cdx2;fubp1;znf41;sirt3;zbtb33;smad6;chaf1a;ikzf4;myog;znf664;ern1;keap1;runx1t1;med20;nfatc2;prdm14;zfp2;cnbp;zxdc;polr3c;dlx2;hmgn2;foxj3;zfp42;ccnt2;atf3;msx1;pou3f2;rbm14;rdbp;zbtb25;dyrk1b;bcl11b;znf214;sox9;utf1;pou4f3;rab10;rab37;nr4a1;vgll4;supt4h1;pou2f1;alx1;sirt1;clock;znf605;rbl2;twist1;gtf2e1;hey2;ddx54;elk1;znf546;cnot6;elf5;irf5;ran;med23;vax1;znf197;trib3;klf12;myd88;hdac10;hoxa3;tcf23;fubp3;zbtb45;zbtb32;bcl6;gsc;tlx3;znf175;usf1;atf7;stat3;sp1;grip1;zfp1;taf4;maff;sohlh2;brms1l;znf501;znf710;cbfa2t2;dlx5;junb;mlxipl;foxc1;ripk2;eid2;tfe3;hoxd8;med18;ezh2;slc30a9;meis3;ppie;ing3;bclaf1;yap1;ihh;whsc2;nr5a2;taf6;cux1;zbtb16;hoxc9;rnf14;znf768;six6;zscan29;irx3;eif2ak2;sfpq;mtf1;znf581;hdx;znf771;sp5;hells;prrx1;zbtb12;hoxc12;klf15;hcls1;scml1;creb3;rarb;plagl2;rnf2;ovol2;pax8;runx3;brf2;habp4;lcorl;meox2;ssrp1;eya3;pogk;foxn1;znf524;dnm2;ikzf3;cdca7;supt16h;sox15;ripply1;zscan21;klf1;hmgb4;mkl1;znf787;tox3;esrrg;nr0b2;lancl2;znf446;rcor2;rfx5;nfatc2ip;znf786;scmh1;tox;znf623;znf688;cited2;zbtb20;tnp1;duxa;dek;prdm12;trim24;gabpa;zfx;med8;uimc1;tbx10;sox6;sp2;nlk;sirt5;pbrm1;znfx1;pknox1;znf496;nanog;tgfb1;acvr2b;elk4;phtf1;tfap2c;pdcd4;ncoa2;drgx;ilf3;prpf6;hif3a;znf24;tceal8;snip1;cnot4;pnn;tox4;mixl1;eya4;elavl2;ctnnb1;polr2f;znf354a;rax;znf451;abt1;grhl2;l3mbtl3;scml2;hdac9;ilf2;ctcf;tcea3;hoxb3;ppargc1b;pcgf2;npas1;dedd;pias1;tbpl2;klf5;rab8a;krba2;crebzf;tsc22d4;znf689;crem;casz1;terf2;mysm1;sirt4;tead1;znf131;sim2;elf1;egr2;sox5;prdm4;gabpb2;elf3;znf398;crx;pcgf1;dbx2;atf2;znf454;nr6a1;snd1;med24;gtf2a1;arid5a;onecut2;scap;gzf1;tspyl2;igsf1;creg1;stat1;rfx3;nab1;psmc5;epc2;zbtb38;runx1;dlx3;mecp2;eda2r |
| biopolymer glycosylation; | 28#177 | 1.45527E-05 | tspan8;man1a1;gcnt4;ogt;b3gnt4;pigb;il17a;sdf2;b3gnt2;st3gal3;st6galnac4;st3gal1;fut1;alg3;a4gnt;b3galt2;b3gnt5;galnt6;galnt9;fut3;b3galt1;gnptg;mgat4a;st3gal4;st6gal1;cog2;st8sia5;b3galt5 |
| wound healing#blood coagulation; | 19#100 | 1.63457E-05 | serpinc1;lman1;procr;tfpi2;apoh;entpd1;f3;gna12;thbd;kng1;pabpc4;cd93;c4bpb;krt1;plaur;gna13;gp6;f7;gp1ba |
| cellular lipid metabolic process; | 83#768 | 1.63457E-05 | pigq;arv1;acot9;neu3;soat1;acaca;pla2g12a;prkag2;alox12;aldh5a1;slc27a2;elovl7;srd5a1;fa2h;ldlrap1;sc5dl;lep;rdh11;b3gnt5;ugcg;gpaa1;fdxr;hadha;mvd;pnpla3;acox2;pdss2;flrt2;nr5a1;nr0b2;acoxl;cryl1;cept1;acsl3;gpx4;cds1;sgpl1;stard5;st6galnac4;hadh;mttp;adm;pip4k2a;psap;cercam;b3galt1;gnpat;pigu;acsm5;cd74;acadl;pigs;pigb;osbpl2;scd;cnbp;aloxe3;mtmr7;aoah;acly;proca1;cmas;scap;ptges;dhrs9;fdps;prkaa1;ghsr;asah2;hacl1;serinc1;c1qtnf2;slc27a4;osbpl7;insig1;pdss1;agk;impa1;acadsb;hexa;st8sia5;fasn;prkaa2 |
| behavior; | 40#294 | 1.69856E-05 | ccr8;cstb;maoa;vegfa;cdh13;cxcl16;egr2;htr1a;ccr7;tgfb2;azu1;drd3;brs3;lep;cx3cl1;enpp2;plaur;cckbr;aprt;prkca;nras;mapk1;cxcl11;trpv1;npas1;kiss1r;ghsr;pmch;ccl28;ccr2;fzd9;adra1b;creb3;efnb3;cxcl14;cck;hexa;mchr1;ccr9;ccrl1 |
| cell morphogenesis#regulation of cell size; | 31#206 | 1.70472E-05 | tgfb1;net1;tspyl2;taok2;egln2;rragc;shc1;bcl6;creg1;cdkn2aip;il3;cda;wisp1;cdkn2d;tgfb2;alox12;dcbld2;camk2d;adra1b;ing3;htra1;crim1;esm1;fgf20;socs1;brms1l;fhl1;ing1;igfbp6;nrg3;ebag9 |
| heart contraction; | 14#47 | 1.70472E-05 | atp2a2;gaa;prkca;adra1b;hcn4;gja1;kcnq1;kcne1;kcnj12;dmpk;tpm1;chrm2;tgfb2;des |
| heart process; | 14#47 | 1.70472E-05 | atp2a2;gaa;prkca;adra1b;hcn4;gja1;kcnq1;kcne1;kcnj12;dmpk;tpm1;chrm2;tgfb2;des |
| energy derivation by oxidation of organic compounds; | 21#117 | 1.81511E-05 | gaa;sdha;dlst;idh3a;aco1;gfpt1;prkag2;uqcrh;c1qtnf2;ndufs1;sucla2;phkg2;adra1b;lep;gfpt2;ppp1cc;ppp1cb;pygl;gsk3b;phka2;phkg1 |
| positive regulation of kinase activity; | 24#144 | 2.33234E-05 | ccnd3;adora2b;malt1;gab1;dgkb;taok2;prkaa1;dgkz;shc1;edn1;traf6;ern1;traf7;dbnl;map3k9;azu1;c1qtnf2;map3k7;map3k4;dgka;madd;agk;pak1;map3k2 |
| secretion by cell; | 40#297 | 2.33234E-05 | kif1c;lin7a;copg2;nrbp1;rapgef4;scrn1;ykt6;arfip1;trappc1;uso1;scamp2;nlrp2;stx16;sergef;srp54;krt20;srp72;stxbp1;stx7;lman1;pram1;sec61a2;vps33a;snph;bace2;sec61a1;traf7;exoc3;optn;tnp2;sec24b;napg;kcnmb4;scfd1;rab9b;doc2a;cog2;gosr1;arfgef2;cog5 |
| heart contraction#regulation of heart contraction; | 13#42 | 2.49791E-05 | atp2a2;prkca;adra1b;hcn4;gja1;kcnq1;kcne1;kcnj12;dmpk;tpm1;chrm2;tgfb2;des |
| coagulation; | 19#102 | 2.54111E-05 | serpinc1;lman1;procr;tfpi2;apoh;entpd1;f3;gna12;thbd;kng1;pabpc4;cd93;c4bpb;krt1;plaur;gna13;gp6;f7;gp1ba |
| Wnt receptor signaling pathway; | 24#145 | 2.76048E-05 | wif1;fzd5;senp2;sfrp2;ctnnb1;wisp1;axin2;csnk1a1;lrp6;fzd9;dkk2;kremen1;rspo1;nlk;wnt5b;cxxc4;wnt11;csnk1g3;wnt2;pygo2;pygo1;csnk2b;zbtb33;gsk3b |
| regulation of cell proliferation; | 56#469 | 2.83237E-05 | nrp1;pth2r;nck1;cd28;vegfa;shc1;edn1;cdh13;gja1;ctnnb1;eid2;asz1;cdkn2d;htr1a;cdkn3;tgfb2;alox12;cgref1;bnipl;cdca7;prkra;myd88;sesn1;prtn3;ihh;flt1;cckbr;ing1;igfbp6;ctbp2;slamf1;ctnna1;klf11;wars;tgfb1;pou3f2;nras;eif2ak2;ppm1d;bcl6;sox9;il3;kiss1r;eef1e1;cdk6;skap2;klf5;ddr2;cd164;hcls1;vsig4;adm;cdk10;cd274;wnt2;runx3 |
| fatty acid metabolic process; | 29#191 | 2.89614E-05 | gnpat;acox2;ptges;acot9;acsm5;prkaa1;cd74;acoxl;acaca;acadl;cryl1;prkag2;ghsr;alox12;hacl1;acsl3;aldh5a1;slc27a2;c1qtnf2;scd;elovl7;aloxe3;fa2h;slc27a4;hadh;acadsb;hadha;fasn;prkaa2 |
| inflammatory response; | 39#291 | 3.66011E-05 | tpst1;cd14;cfi;gpr68;ripk2;cfb;il1rap;lyz;ccr7;azu1;kng1;elf3;il20;myd88;aoah;cx3cl1;prdx5;ltb4r;hdac9;krt1;c1qc;hpse;olr1;tgfb1;prkca;procr;tlr2;cxcl11;bcl6;il17a;ghsr;gpx4;ccr2;stat3;il13;chst1;c4bpb;cxcl14;abcf1 |
| muscle contraction; | 26#165 | 3.83207E-05 | myom1;gaa;prkca;chrne;gja1;kcnq1;kcne1;kcnj12;ppp1r12b;slmap;mybpc2;chrm2;pgam2;sgca;kng1;brs3;mrcl3;stbd1;uts2;sntb1;hcn4;tpm1;ltb4r;fxyd1;snta1;des |
| muscle system process; | 26#165 | 3.83207E-05 | myom1;gaa;prkca;chrne;gja1;kcnq1;kcne1;kcnj12;ppp1r12b;slmap;mybpc2;chrm2;pgam2;sgca;kng1;brs3;mrcl3;stbd1;uts2;sntb1;hcn4;tpm1;ltb4r;fxyd1;snta1;des |
| negative regulation of signal transduction; | 19#104 | 3.95506E-05 | prkca;rgs4;pld2;rgs17;taok3;eid2;tnfaip3;fstl3;pak1ip1;dkk2;rgs1;nlk;rgs22;htra1;rgs21;ihh;cxxc4;socs1;rgs11 |
| hemostasis; | 19#105 | 4.976E-05 | serpinc1;lman1;procr;tfpi2;apoh;entpd1;f3;gna12;thbd;kng1;pabpc4;cd93;c4bpb;krt1;plaur;gna13;gp6;f7;gp1ba |
| lipid metabolic process; | 96#946 | 5.50022E-05 | pigq;arv1;acot9;neu3;soat1;acaca;pla2g12a;srd5a3;prkag2;alox12;ddhd1;aldh5a1;slc27a2;pafah1b3;elovl7;srd5a1;fa2h;ldlrap1;sc5dl;lep;rdh11;b3gnt5;ugcg;gpaa1;fdxr;lrp10;pparg;hadha;mvd;pnpla3;acox2;pdss2;flrt2;plcz1;nr5a1;nr0b2;acoxl;liph;cryl1;cept1;acsl3;gpx4;cds1;sgpl1;stard5;st6galnac4;hadh;mttp;chka;ocrl;adm;pip4k2a;psap;cercam;b3galt1;gnpat;pigu;acsm5;cd74;acadl;pigs;pigb;apobec1;osbpl2;scd;cnbp;aloxe3;mtmr7;aoah;pltp;enpp2;acly;proca1;scap;cmas;ptges;dhrs9;fdps;prkaa1;pld2;ghsr;asah2;hacl1;serinc1;c1qtnf2;slc27a4;osbpl7;insig1;pdss1;agk;impa1;acadsb;hexa;st8sia5;fasn;prkaa2 |
| negative regulation of progression through cell cycle; | 32#225 | 5.88793E-05 | cdc73;pnn;aurkaip1;foxc1;ern1;cdkn2d;cdkn3;cgref1;lats2;rbm5;sesn1;nbl1;cdk2ap1;ctcf;ing1;dcc;tgfb1;tspyl2;rassf5;bcl6;ppp1r15a;gas2;eif4g2;tp53bp2;pdcd4;plekho1;bub1b;rhob;dph1;trim13;rbl2;runx3 |
| glycoprotein metabolic process; | 30#208 | 8.28161E-05 | tspan8;man1a1;gcnt4;ogt;b3gnt4;pigb;bace2;il17a;sdf2;b3gnt2;st3gal3;st6galnac4;st3gal1;fut1;alg3;a4gnt;b3galt2;tnip1;b3gnt5;galnt6;galnt9;fut3;b3galt1;gnptg;mgat4a;st3gal4;st6gal1;cog2;st8sia5;b3galt5 |
| organelle organization and biogenesis; | 141#1526 | 0.000103722 | exosc10;daam2;utp6;kif9;actn4;asz1;tmod1;sox15;supt16h;ndc80;capza3;klf1;vps72;taok2;tomm22;ablim1;cdc42bpb;pex16;tlk1;cdc42ep5;hook3;chd3;kifc3;capg;tnp1;tnks;rasa1;sirt3;kpna2;rrp9;chaf1a;shroom2;sox6;atp2c1;setdb2;cntrob;sirt5;pbrm1;arhgdib;wdr36;cno;elmo1;ppp4c;hmgn2;kif3a;prkci;akap8;pak1;des;dnm1l;nusap1;imp4;ebna1bp2;rbm14;dffa;mid1;pld2;fscn1;dync1i1;ghsr;map4k2;cdyl2;mark1;supt4h1;pex12;tubg1;sirt1;rbl2;nck1;trim54;h2afz;ran;capza2;ykt6;arhgap1;rcc1;tubgcp5;tspyl1;hdac9;hdac10;krt20;hsp90aa1;exosc7;fgd1;abcd3;slc25a10;utp3;nhp2l1;nras;cpa4;bcl6;tubgcp3;ftsj1;gpx4;ftsj2;set;ranbp9;tmem48;nubp1;hspd1;psap;wnt2;cog2;terf2;krt9;hps6;gaa;kif1c;sirt4;npm2;nap1l5;bub3;sfn;ezh2;kif3b;slc25a17;ing3;sgcb;tinf2;tubgcp4;nde1;atg4a;ncaph;tspyl2;tubb6;kif18a;abi2;atg12;cln5;picalm;optn;hells;epc2;sgcg;bub1b;hcn4;sycp3;dot1l;shank1;hexa;ncapg |
| intracellular transport; | 92#910 | 0.000105831 | stx10;snx15;chml;ankrd27;kif9;nrbp1;gga3;ran;ykt6;arfip1;synj2bp;uso1;rtp4;scamp2;stx16;vps16;nup35;nfkbil1;srp72;gle1;hsp90aa1;nxf1;rpgr;ctsa;stx7;slc25a10;taok2;tomm22;sec61a2;bcl6;senp2;pex16;tlk1;ramp1;sec61a1;sqstm1;ap1s3;vps26b;set;ap1g1;tmem48;camk4;vps41;rhob;ap3d1;kifc3;ywhab;cep57;rae1;hspd1;tnks;cog2;cog5;kif1c;kpna2;cd74;copg2;kpna6;stx4;ap3s1;xpo6;trappc1;pcna;atp2c1;kif3b;slc25a17;kpna3;gnptg;srp54;kif3a;prkci;atg4a;ddx19b;rtp3;nupl2;lman1;malt1;tgfb1;tubb6;cdc37;kif18a;klhl2;stat1;dync1i1;optn;napg;sec24b;vps26a;rab9b;pex12;gosr1;gsk3b |
| metal ion transport; | 73#682 | 0.000105831 | btbd10;slc17a4;kcnc1;kcnk2;slc22a4;kctd9;kcng1;heph;scn1a;kcnab3;slc13a1;cachd1;scnn1b;kctd8;slc31a1;kcnk1;kctd19;kcna2;cacna2d3;kcnq1;cacng6;kcnip4;slc30a10;nedd4l;cacng7;slc9a8;slc30a4;scn1b;trpc7;slc39a6;kcns1;slc30a6;kcnj13;kcns3;gja1;kcnj12;kcnj11;slc5a3;slc30a9;cnga3;atp2c1;kcnv2;slc39a14;cacna1f;slc8a2;slc9a5;atp2a2;csn2;cacnb3;kctd18;trpv1;slc30a1;kcne1;kcnk6;kctd12;slc9a7;kcnj5;cacng3;sfxn3;slc10a5;kcnmb4;tmem37;trpv5;trpc5;slc38a4;hcn4;kcnq2;kcnf1;atp2b3;tfrc;slc23a2;slc39a7;kcna5 |
| cell differentiation#cell development#cellular morphogenesis during differentiation; | 21#126 | 0.000107873 | nrp1;fez1;pou3f2;gja1;ctnnb1;dscaml1;cyfip1;pou4f3;tgfb2;b3gnt2;slitrk4;slitrk3;efnb3;ngfr;cacna1f;cck;sema4d;prkci;dcc;nrcam;dlx5 |
| secretory pathway; | 33#240 | 0.000108149 | kif1c;lin7a;copg2;nrbp1;rapgef4;scrn1;ykt6;trappc1;uso1;scamp2;stx16;srp54;srp72;stxbp1;stx7;lman1;pram1;vps33a;snph;exoc3;traf7;optn;tnp2;sec24b;napg;kcnmb4;scfd1;rab9b;doc2a;gosr1;cog2;arfgef2;cog5 |
| negative regulation of programmed cell death; | 32#230 | 0.000108149 | cd28;vegfa;foxc1;cdh13;cd74;cdkn2d;alox12;tnfaip3;azu1;birc3;mcl1;ihh;sema4d;ctnna1;malt1;hspb1;prkaa1;nras;dffa;alb;arhgdia;bcl6;il3;psmc5;pim1;ciapin1;sycp3;faim;tax1bp1;clcf1;rasa1;gsk3b |
| epidermis development; | 22#135 | 0.000108149 | atp2a2;krt13;pou3f2;sox9;keap1;foxn1;tgfb2;sfn;elf3;il20;krt5;atp2c1;plod1;ugcg;snai1;klk7;maff;tgm5;ngfr;krt1;eda2r;krt9 |
| multicellular organismal development#tube development#tube morphogenesis; | 16#84 | 0.000112257 | gzf1;foxc1;edn1;ctnnb1;asz1;nts;kng1;tinag;kcnmb4;adra1b;gch1;ihh;flt1;gna13;wnt2;ipmk |
| energy reserve metabolic process; | 14#55 | 0.000114224 | gaa;phkg2;adra1b;lep;ppp1cc;gfpt2;ppp1cb;gfpt1;prkag2;c1qtnf2;pygl;gsk3b;phka2;phkg1 |
| response to hypoxia; | 13#48 | 0.000114711 | tgfb1;plod1;prkaa1;hyou1;egln2;chrna4;vegfa;edn1;scnn1b;pdia2;actn4;plod2;tgfb2 |
| GO:0000122,negative regulation of transcription from RNA polymerase II promoter; | 21#127 | 0.000125555 | gzf1;msx1;zbtb32;vps72;nr0b2;bcl6;npas1;sim2;ctnnb1;sox15;klf12;stat3;ncoa2;supt4h1;nr6a1;mecp2;ctcf;cux1;twist1;lcor;klf11 |
| positive regulation of protein metabolic process; | 16#85 | 0.000141827 | tgfb1;nck1;egln2;cd28;aurkaip1;gja1;il3;cdc42ep5;azu1;il20;samd4a;hcls1;myd88;clcf1;ift88;scap |
| ectoderm development; | 23#146 | 0.000147291 | atp2a2;krt13;pou3f2;ctnnb1;sox9;keap1;foxn1;tgfb2;sfn;elf3;il20;krt5;atp2c1;plod1;ugcg;snai1;klk7;maff;tgm5;ngfr;krt1;eda2r;krt9 |
| cellular catabolic process; | 83#809 | 0.00015544 | exosc10;neu3;sdha;upf3a;ephx1;idh3a;usp30;lysmd3;psmd14;aldh5a1;tst;bcat2;arg1;fut1;sucla2;uhrf2;bpgm;g6pd;usp2;dera;tktl1;exosc7;psma8;pnpla3;rnps1;uroc1;dcp1a;afmid;dlst;sqstm1;ldha;gpx4;sgpl1;isg20;arih1;psmb5;ddah1;gnpda1;agmat;pabpc4;hibadh;usp33;usp11;gaa;maoa;spaca3;lyz;pgam2;kynu;uchl5;pgd;usp39;syvn1;usp32;acly;qdpr;fbxl4;ampd3;dcp2;upp1;usp15;dffa;cda;dio3;aco1;hk3;psmc5;aldh1l1;adra1b;pdha1;gch1;ldhc;gns;pts;hyal2;ldhb;hexa;ogdh;dbh;rnaseh1;bckdhb;usp13;pgk2 |
| system process; | 141#1539 | 0.000157769 | chml;meox2;crybb3;eya3;asz1;mybpc2;myo3a;qprt;grm8;marveld2;drd3;brs3;nr4a2;tyr;uts2;rpgr;gabrb2;ablim1;pcdhb5;cds1;chrna5;cldn14;kifc3;vsx1;iqcb1;plp1;rs1;slc22a18;aipl1;maoa;strn4;edn1;gja1;rabggta;crygd;stbd1;kcnv2;npr2;wdr36;ltb4r;tpm1;npr3;rd3;des;rtp3;sord;atp2a2;tgfb1;mapk1;kcne1;slmap;bbs9;pou4f3;sgca;gprc5d;kcnmb4;mrcl3;aldh7a1;pcdhb11;sncb;adra1b;kcnq2;mtnr1b;opn1sw;timp3;adora2b;aqp1;soat1;eya4;rom1;lin7a;galr1;ctnnb1;kng1;znf354a;aldh5a1;rax;sntb1;rtp4;lep;scnn1b;pde6d;snta1;cckbr;efemp1;prkca;nras;snph;kcnq1;slc6a2;chrm2;nedd4l;pmch;nts;b3gnt2;pcdhb6;best1;camk4;cabp4;tgfbi;scn1b;adm;doc2a;wnt2;pdc;agtr1;sema4a;gaa;myom1;chrne;chrna4;ndufb9;foxc1;kcnj12;ppp1r12b;mbp;egr2;pgam2;tgfb2;azu1;pnoc;th;cnga3;crx;crybb2;clcn5;eln;dmpk;cacna1f;fxyd1;olr1;six6;cln5;pxk;gnal;gch1;hcn4;slc6a5;hexa;dbh;ift88 |
| cell part morphogenesis; | 29#204 | 0.000164356 | nrp1;fez1;pou3f2;nck1;cdh13;gja1;cdc42ep4;dscaml1;fxyd5;pou4f3;cyfip1;cdc42ep5;tgfb2;b3gnt2;klf5;slitrk4;slitrk3;capg;efnb3;akap4;ngfr;cacna1f;cck;sema4d;fgd1;thbs4;dcc;nrcam;dlx5 |
| cell projection morphogenesis#cell projection organization and biogenesis; | 29#204 | 0.000164356 | nrp1;fez1;pou3f2;nck1;cdh13;gja1;cdc42ep4;dscaml1;fxyd5;pou4f3;cyfip1;cdc42ep5;tgfb2;b3gnt2;klf5;slitrk4;slitrk3;capg;efnb3;akap4;ngfr;cacna1f;cck;sema4d;fgd1;thbs4;dcc;nrcam;dlx5 |
| cell projection morphogenesis; | 29#204 | 0.000164356 | nrp1;fez1;pou3f2;nck1;cdh13;gja1;cdc42ep4;dscaml1;fxyd5;pou4f3;cyfip1;cdc42ep5;tgfb2;b3gnt2;klf5;slitrk4;slitrk3;capg;efnb3;akap4;ngfr;cacna1f;cck;sema4d;fgd1;thbs4;dcc;nrcam;dlx5 |
| carbohydrate metabolic process; | 82#800 | 0.0001795 | pigq;uap1;neu3;sdha;has3;idh3a;slc2a2;prkag2;brs3;fut1;sucla2;chst8;lep;bpgm;khk;g6pd;hyal3;hpse;st6gal1;tktl1;ipmk;phkg1;pglyrp3;slc25a10;fuca2;chst14;dlst;galns;ldha;pdk2;phkg2;gnpda1;b3galt2;pfkfb2;hibadh;lalba;b3galt1;cercam;hgsnat;cog2;tff1;phka2;gaa;spaca3;foxc1;gfpt1;pgam2;chst12;stbd1;pgd;a4gnt;aoah;fut3;me2;ppp1cb;fbp2;acly;cmas;sord;gcnt4;aco1;hk3;c1qtnf2;ganab;ugdh;adra1b;pdha1;gns;ldhc;chst1;impa1;gfpt2;ppp1cc;mgat4a;ldhb;hyal2;hexa;ogdh;pygl;st8sia5;gsk3b;pgk2 |
| negative regulation of cell proliferation; | 31#225 | 0.000188434 | wars;pth2r;tgfb1;eif2ak2;cdh13;gja1;ppm1d;bcl6;asz1;cdkn2d;cdkn3;kiss1r;tgfb2;bnipl;cgref1;eef1e1;cdk6;skap2;prkra;cd164;sesn1;vsig4;cdk10;cd274;wnt2;ing1;igfbp6;ctbp2;klf11;runx3;ctnna1 |
| positive regulation of apoptosis; | 34#256 | 0.000199764 | cd70;traf3;ripk2;sap30bp;ern1;cd2;tgfb2;kng1;sfn;dnm2;prkra;bclaf1;plekhf1;ngfr;btk;bik;bcl2l11;dcc;adamtsl4;tgfb1;prkca;mmp9;tlr2;mapk1;tnfrsf25;bcl6;pdcd5;dedd;eef1e1;tp53bp2;gch1;lalba;plagl2;runx3 |
| embryonic development ending in birth or egg hatching; | 17#95 | 0.00020222 | nle1;meox2;foxc1;edn1;slc30a1;gja1;sfrp2;cops3;dscaml1;keap1;adam10;hcn4;ripply1;cdx2;maff;gna13;ipmk |
| chordate embryonic development; | 17#95 | 0.00020222 | nle1;meox2;foxc1;edn1;slc30a1;gja1;sfrp2;cops3;dscaml1;keap1;adam10;hcn4;ripply1;cdx2;maff;gna13;ipmk |
| protein metabolic process; | 451#5858 | 0.000234684 | pigq;ttbk1;ptk2;fkbp3;ppil3;cfb;fbxo10;usp30;ears2;spcs2;myo3a;nrbp1;nsf;psmc2;mark2;dusp10;msrb3;prkra;map3k7;fut1;map3k4;fkbp6;cdk2ap1;c1qc;ctss;st13;cdc16;sumo1;glyctk;ctsa;cdk9;tspan8;chst14;fbxl17;rce1;tec;cdc42bpb;b3gnt4;il3;sqstm1;traf7;cdc42ep5;plod2;adam20;arih1;st3gal3;st6galnac4;ptpn18;cldn14;phkg2;rbbp6;pstk;rnf139;pabpc4;prmt2;capg;klk12;aplp1;csnk1g3;rasa1;sirt3;eif3f;phka2;abcf1;cpa3;chaf1a;akt2;ern1;xpo6;uchl5;eif1b;mtmr7;mex3b;calr3;mki67ip;usp39;npr2;rpl8;cpxm1;ptpla;usp32;mars2;abl2;fbxl4;adam12;rbm14;dyrk1b;usp15;fbxl8;wars2;bace2;ppm1k;map4k2;eif4g2;aurkc;tgm7;pcsk7;dpp8;klk7;lonp1;sirt1;mrps18b;hectd2;mrpl3;f7;pja2;usp13;prkaa2;gsk3b;bhmt;nck1;trim54;traf6;ogt;psmd14;ero1lb;epha3;mrpl10;sdf2;aldh5a1;lrrk1;lats2;tbcc;trib3;myd88;serpina5;uhrf2;dnajc17;cdc14b;fbxl22;hdac10;ubqln4;st6gal1;hsp90aa1;phkg1;b3galt5;ccnd3;wars;leprel2;prkca;l2hgdh;mmp9;tmub2;cpa4;ppm1d;senp2;senp3;adam10;ap1s3;b3gnt2;sugt1;tinag;adam21;grpel2;ap1g1;tmem48;camk4;nubp1;cpb1;c4bpb;socs1;b3galt1;usp11;snrk;wnt2;clk3;ddb1;ube2m;oxsr1;mrpl36;fbxo45;araf;ripk2;slc7a7;eid2;tfe3;fbxo9;azu1;il20;fbxl13;lrpap1;tprkb;st3gal1;itk;lonrf1;ctsw;ppie;rps8;kpna3;ppic;tsfm;ihh;mrps9;igf2bp3;zbtb16;lrrc14;rnf14;fbxw8;malt1;prkaa1;eif2ak2;abi2;cln5;map4k3;mmp12;picalm;hars;srpk1;cdk6;skap2;trpc5;pum2;hcls1;bub1b;ero1l;aurkb;pja1;rnf2;senp8;dph1;ift88;tpst1;klk13;hunk;prepl;mrpl30;mrps18c;ict1;asz1;cpa5;map3k9;asb2;rapgef4;camk2d;supt16h;apeh;chst8;capza3;gpaa1;sh3rf2;hpse;adprh;taok2;pepd;cdkn2aip;nfatc2ip;casp7;tlk1;vrk1;pdk2;pim1;sars;samd4a;ddr2;zdhhc7;mttp;b3galt2;galnt6;ap3d1;ptpn21;tgm5;ulk3;tnp1;tnks;qpctl;senp1;herc6;ccnb1ip1;eif4h;ttll5;aipl1;pigu;man1a1;cfi;gja1;atg7;rabggta;eef1d;il1rap;copg2;ppp4r2;mrpl27;ap3s1;rbck1;apobec1;traf1;med8;mrpl22;chst12;nlk;plod1;crim1;galnt9;sirt5;tor2a;fut3;syvn1;znfx1;gnptg;ppp1cb;pam;eif1ax;ogfod1;prkci;pak1;tp53rk;map3k2;ctnna1;axl;tgfb1;adam32;mapk1;rad51;gcnt4;mid1;pld2;dusp22;map2k7;lcmt1;acvr2b;cda;ace2;slmap;klhl15;ppm1l;ghsr;rps29;mark1;cdkl2;gucy2c;dusp13;klk9;trpv5;fbxo38;slc38a4;adrm1;opn1sw;cct6b;tubg1;ppp1cc;mgat4a;stk39;clcf1;eif5;cnot4;rnf41;ubtd2;egln2;nek8;aurkaip1;anapc7;hus1;lin7a;pdia2;tysnd1;matn1;tnfaip3;gga3;scrn1;twf1;capza2;asphd1;lrrc41;mrpl16;b3gnt5;camk2g;plaur;hdac9;flt1;btk;usp2;cpa6;psma8;mapkapk5;rps3;hspb1;scarf1;ncln;taok3;rps10;farsa;cndp2;il17a;nedd4l;csnk1a1;rplp1;mrpl37;oma1;eef1e1;pias1;set;pak3;psmb5;ranbp9;alg3;tnip1;htra1;cdk10;hspd1;usp33;st3gal4;cog2;lrrc29;ttll9;sirt4;cd28;cd74;pigs;pigb;rad23a;mrpl47;wee2;hectd3;a4gnt;gan;epha1;prtn3;p4ha1;dmpk;krt1;rpl9;eif3e;tubgcp4;olr1;srms;atg4a;pskh1;scap;ttc1;ubqln2;lman1;jak1;tubb6;rsl1d1;stat1;atg12;fbxo47;brsk1;hk3;denr;c1qtnf2;psmc5;napg;nuak1;eif3i;nars;capn2;mrpl35;st8sia5;dnaja4;fbxl6 |
| negative regulation of apoptosis; | 31#227 | 0.000234684 | hspb1;malt1;nras;prkaa1;cd28;vegfa;dffa;foxc1;cdh13;arhgdia;alb;cd74;bcl6;il3;cdkn2d;alox12;tnfaip3;azu1;pim1;birc3;mcl1;ciapin1;ihh;sycp3;sema4d;tax1bp1;faim;rasa1;clcf1;gsk3b;ctnna1 |
| positive regulation of programmed cell death; | 34#258 | 0.000243618 | cd70;traf3;ripk2;sap30bp;ern1;cd2;tgfb2;kng1;sfn;dnm2;prkra;bclaf1;plekhf1;ngfr;btk;bik;bcl2l11;dcc;adamtsl4;tgfb1;prkca;mmp9;tlr2;mapk1;tnfrsf25;bcl6;pdcd5;dedd;eef1e1;tp53bp2;gch1;lalba;plagl2;runx3 |
| growth; | 38#300 | 0.000246044 | egln2;rragc;shc1;foxc1;gja1;cdkn2d;tgfb2;alox12;bnipl;camk2d;sox15;ing3;crim1;fhl1;ing1;igfbp6;net1;tgfb1;pou3f2;tspyl2;taok2;creg1;bcl6;cdkn2aip;cda;il3;wisp1;dio3;ghsr;dcbld2;adra1b;htra1;esm1;fgf20;brms1l;socs1;ebag9;nrg3 |
| antigen processing and presentation; | 5#415 | -0.000288897 | cd1a;procr;cd1b;cd74;ap3d1 |
| negative regulation of cell adhesion; | 9#26 | 0.000307349 | tgfbi;cdh13;arhgdia;bcl6;arhgdib;fxyd5;adam10;kng1;rasa1 |
| reproduction; | 59#537 | 0.000309781 | dek;cd28;npm2;dnali1;foxc1;izumo1;spata9;asz1;dmc1;thbd;elspbp1;srd5a1;bcat2;sox15;serpina5;capza3;nr6a1;akap4;flt1;ift81;ctbp2;dazap1;tsga10;tgfb1;gfer;rqcd1;nhp2l1;nr5a1;spin1;ccin;bcl6;sox9;ace2;ghsr;adam20;cdkl2;dedd;pmch;gpx4;dusp13;tnp2;adam21;gnpda1;tnip1;vapb;cct6b;adm;sycp3;maff;mfge8;cep57;tnp1;cylc1;vgf;wnt2;hexa;crem;adad1;krt9 |
| sexual reproduction; | 39#315 | 0.000367931 | npm2;dnali1;foxc1;izumo1;spata9;dmc1;elspbp1;serpina5;capza3;nr6a1;akap4;ift81;tsga10;dazap1;tgfb1;nhp2l1;gfer;spin1;ccin;bcl6;adam20;dedd;pmch;gpx4;tnp2;dusp13;adam21;gnpda1;cct6b;sycp3;tnp1;cep57;mfge8;cylc1;vgf;hexa;crem;adad1;krt9 |
| regulation of a molecular function; | 70#671 | 0.000403071 | adora2b;chml;rgs4;shc1;traf6;galr1;actn4;map3k9;grm8;lats2;trib3;map3k7;map3k4;ldlrap1;arhgap1;myd88;dgka;cckbr;fgd1;spry1;sumo1;ccnd3;gab1;evi5;dgkb;prkca;taok2;dgkz;bcl6;arfgap1;traf7;dbnl;gnai3;ccr2;crebzf;edn1;ripk2;ccng1;ern1;cdkn2d;cdkn3;azu1;tbc1d14;sfn;nlrp2;madd;taf6;abl2;pak1;map3k2;ccnt2;tgfb1;malt1;tspyl2;ecsit;cdc37;prkaa1;als2cr12;stat1;dusp22;smap1;serinc1;gnal;c1qtnf2;pdcd4;agk;gabbr1;eda2r;rbl2;diras3 |
| amino acid derivative metabolic process; | 16#90 | 0.000416549 | sms;maoa;afmid;ckm;srm;dio3;tgfb2;aldh5a1;kynu;th;nr4a2;sncb;agmat;pnmt;dbh;amd1 |
| amine metabolic process; | 67#636 | 0.000418394 | uap1;bhmt;srm;ears2;aldh5a1;nr4a2;tyr;bcat2;arg1;ccnd3;wars;sms;uroc1;prkca;pepd;afmid;ssu72;galns;farsa;il3;sars;ddah1;gnpda1;agmat;hibadh;psph;socs1;hgsnat;maoa;foxc1;slc7a7;gfpt1;tfe3;tgfb2;cbs;kynu;il20;chst12;th;plod1;znfx1;agxt2l1;qdpr;mars2;wars2;pycr1;dio3;hars;ugdh;cad;sncb;hcls1;gch1;gns;chst1;nars;gfpt2;gmps;pts;hyal2;pnmt;hexa;dbh;bckdhb;amd1;clcf1;coro2a |
| multicellular organismal development#tube development; | 19#117 | 0.000481263 | gzf1;foxc1;edn1;gja1;sim2;ctnnb1;asz1;nts;kng1;tinag;kcnmb4;adra1b;gch1;ihh;flt1;gna13;cux1;wnt2;ipmk |
| embryonic pattern specification; | 9#28 | 0.000582993 | ctnnb1;flt1;gna13;hoxd8;meox2;edn1;ripply1;ihh;sim2 |
| nitrogen compound metabolic process; | 70#678 | 0.00058722 | uap1;bhmt;srm;ears2;aldh5a1;tst;nr4a2;tyr;bcat2;arg1;hsp90aa1;ccnd3;wars;sms;uroc1;prkca;pepd;afmid;ssu72;galns;farsa;il3;sars;ddah1;gnpda1;agmat;hibadh;psph;socs1;hgsnat;nit1;maoa;foxc1;slc7a7;tfe3;gfpt1;tgfb2;cbs;kynu;il20;chst12;th;plod1;znfx1;agxt2l1;qdpr;mars2;wars2;pycr1;dio3;hars;ugdh;cad;sncb;hcls1;gch1;gns;chst1;nars;gfpt2;gmps;pts;hyal2;hexa;dbh;pnmt;bckdhb;amd1;clcf1;coro2a |
| cation transport; | 84#849 | 0.00058722 | btbd10;slc17a4;kcnc1;atp13a5;nox1;kcnk2;slc22a4;kctd9;kcng1;heph;scn1a;kcnab3;slc13a1;cachd1;scnn1b;kctd8;slc31a1;kcnk1;kctd19;atp5o;kcna2;cacna2d3;kcnq1;cacng6;kcnip4;slc30a10;nedd4l;cacng7;slc9a8;slc30a4;scn1b;trpc7;slc39a6;atp6v1d;kcns1;slc30a6;kcnj13;kcns3;gja1;slc22a16;kcnj12;kcnj11;slc5a3;slc30a9;cnga3;atp2c1;atp6v1g3;kcnv2;atp6v0b;slc39a14;cacna1f;rhcg;slc8a2;slc9a5;atp2a2;csn2;cacnb3;kctd18;trpv1;slc30a1;kcne1;kcnk6;kctd12;atp5b;slc9a7;cacng3;kcnj5;sfxn3;slc10a5;kcnmb4;tmem37;trpv5;trpc5;slc38a4;pkd2l1;hcn4;kcnq2;kcnf1;atp2b3;tfrc;slc23a2;slc39a7;kcna5;nudt9 |
| regulation of signal transduction; | 80#800 | 0.000591256 | sectm1;rgs4;shc1;traf6;rasgrp2;slc20a1;tnfaip3;rapgef4;dkk2;myd88;rgs22;iqsec2;traf3ip2;iqsec3;fgd1;spry1;rgs11;net1;prkca;evi5;nras;taok2;rgs17;cdc42bpb;taok3;bcl6;senp2;cdkn2aip;arfgap1;ramp1;il3;sqstm1;traf7;pak1ip1;rapgef1;arhgef6;cited2;htra1;trim38;socs1;rasa4;rasal2;arfgef2;rasa1;ltbr;pigu;vegfa;gja1;cdh13;ripk2;eef1d;eid2;lgals9;fstl3;tbc1d14;il20;rgs1;atp2c1;vav3;iqgap2;nlk;ihh;cxxc4;dmpk;mapk8ip1;ctnna1;arhgef9;tgfb1;malt1;tspyl2;pld2;smap1;acvr2b;ghsr;psd4;hcls1;rgs21;eda2r;trim13;clcf1 |
| single fertilization; | 13#56 | 0.000591256 | nhp2l1;serpina5;gnpda1;npm2;dnali1;akap4;izumo1;mfge8;tnp1;adam20;tnp2;adam21;elspbp1 |
| alcohol metabolic process; | 50#445 | 0.000665593 | gaa;maoa;soat1;pgam2;gfpt1;prkag2;tgfb2;brs3;th;nr4a2;cnbp;fut1;ldlrap1;sc5dl;pgd;lep;chdh;bpgm;g6pd;fdxr;fbp2;tktl1;mvd;ipmk;scap;sord;slc25a10;fdps;prkaa1;nr0b2;gdpd1;ldha;hk3;pdk2;gdpd3;ugdh;sncb;insig1;pdha1;hibadh;pfkfb2;ldhc;chst1;gfpt2;ldhb;ogdh;dbh;pnmt;prkaa2;pgk2 |
| intra-Golgi vesicle-mediated transport; | 7#17 | 0.000670048 | copg2;optn;cog2;gosr1;napg;stx16;cog5 |
| regulation of MAP kinase activity; | 16#93 | 0.000756522 | adora2b;gab1;prkca;prkaa1;rgs4;shc1;dusp22;dbnl;map3k9;c1qtnf2;pdcd4;trib3;madd;spry1;pak1;map3k2 |
| glycolipid metabolic process; | 9#29 | 0.000766532 | st6galnac4;neu3;prkaa1;ugcg;b3gnt5;psap;hexa;aldh5a1;st8sia5 |
| generation of neurons#neuron differentiation#neuron development#neuron morphogenesis during differentiation; | 19#120 | 0.000777125 | nrp1;fez1;pou3f2;gja1;dscaml1;cyfip1;pou4f3;tgfb2;b3gnt2;slitrk4;slitrk3;efnb3;ngfr;cacna1f;sema4d;cck;nrcam;dcc;dlx5 |
| generation of neurons#neuron differentiation#neuron development#neuron morphogenesis during differentiation#neurite morphogenesis; | 19#120 | 0.000777125 | nrp1;fez1;pou3f2;gja1;dscaml1;cyfip1;pou4f3;tgfb2;b3gnt2;slitrk4;slitrk3;efnb3;ngfr;cacna1f;sema4d;cck;nrcam;dcc;dlx5 |
| zinc ion transport; | 8#23 | 0.000783332 | slc30a4;slc30a1;slc39a14;slc39a6;slc39a7;slc30a10;slc30a6;slc30a9 |
| in utero embryonic development; | 12#50 | 0.000784444 | nle1;hcn4;foxc1;edn1;slc30a1;gja1;cops3;cdx2;maff;keap1;adam10;gna13 |
| fertilization; | 13#58 | 0.000838162 | nhp2l1;serpina5;gnpda1;npm2;dnali1;akap4;izumo1;mfge8;tnp1;adam20;tnp2;adam21;elspbp1 |
| response to stimulus; | 284#3553 | 0.000925473 | cfb;cops3;myo3a;dusp10;il1r2;prkra;map3k7;map3k4;pparg;c1qc;ctss;tusc5;sumo1;gab1;il3;sqstm1;plod2;fancf;ccl28;rrm2b;clec1a;pabpc4;vsx1;lalba;gna13;cxcl14;gp1ba;abcf1;cd70;chaf1a;edn1;cdh13;ern1;crygd;thbd;icos;fech;rspo1;tff3;sesn1;wdr36;aprt;rtp3;sord;tshb;rbm14;tlr2;snx27;pag1;map4k2;mrps35;kcnmb4;tgm7;dpp8;cck;clock;f7;gsk3b;adora2b;cstb;rom1;traf6;ogt;ucn2;lats2;trib3;myd88;ythdf2;pde6d;st6gal1;sema4d;hsp90aa1;serpinc1;defb123;prkca;fcgr3a;nras;pram1;ppm1d;bcl6;slc6a2;npffr2;kiss1r;znf175;stat3;tgfbi;adm;c4bpb;maff;pdc;wnt2;ccrl1;ddb1;oxsr1;chrna4;vegfa;gpr68;ripk2;mbp;lyz;htr1a;tgfb2;xrcc2;azu1;hspa1l;il20;crybb2;errfi1;itk;ctsw;cd93;mrps9;hspa4;prdx5;ngfr;malt1;polh;prkaa1;eif2ak2;sfpq;mtf1;ecsit;map4k3;cysltr2;f3;fance;cd1a;hcls1;creb3;ero1l;chst1;hexa;ccr9;anxa11;ugt1a6;sectm1;tpst1;cd14;ssrp1;rsad2;actn4;asz1;igj;foxn1;neil1;grm8;drd3;brs3;tmod1;supt16h;sox15;hamp;cx3cl1;hpse;cd1b;hadha;rpgr;inppl1;abcg2;trpm8;procr;taok2;gbp5;hsph1;tlk1;dbnl;dcbld2;cited2;vapb;tnp1;gp6;fen1;tff1;cd274;rs1;aipl1;slc22a18;maoa;cfi;shroom2;gja1;il1rap;cdkn2d;kcnj11;sp2;rgs1;pcna;plod1;kcnv2;nlrp2;enpp2;arhgdib;ltb4r;syvn1;rd3;ndrg1;tgfb1;rad51;mapk1;trpv1;ppp1r15a;map2k7;bbs9;ghsr;cst11;adra1b;opn1sw;efnb3;stk39;timp3;ccr8;egln2;hus1;cxcl16;ephx1;pdia2;ccr7;cgref1;kng1;rtp4;hyou1;lep;scnn1b;hdac9;ilf2;plaur;krt20;cckbr;mapkapk5;pglyrp3;hspb1;tfpi2;apoh;cxcl11;asna1;npas1;il17a;nedd4l;gpx4;pmch;gna12;eef1e1;isg20;ccr2;fzd9;ifi35;best1;il13;tnip1;crebzf;hspd1;mchr1;sema4a;ltbr;cd28;entpd1;cd74;spaca3;elf1;ugt1a9;egr2;rad23a;rad54l;sfn;elf3;cnga3;crx;aoah;krt1;igsf6;cacna1f;olr1;sarm1;lilra6;scap;lman1;cacnb3;alb;stat1;brsk1;epc2;cd164;alkbh1;rad1 |
| reproductive process; | 37#305 | 0.000980454 | dek;cd28;npm2;dnali1;foxc1;izumo1;asz1;dmc1;thbd;elspbp1;srd5a1;bcat2;sox15;serpina5;akap4;flt1;ctbp2;tgfb1;rqcd1;nhp2l1;nr5a1;sox9;ace2;ghsr;adam20;cdkl2;tnp2;adam21;gnpda1;tnip1;vapb;adm;maff;tnp1;mfge8;vgf;wnt2 |
| regulation of mitosis; | 14#67 | 0.000993637 | tgfb1;hectd3;cd28;aurkaip1;bub1b;rcc1;npm2;shc1;hus1;mad2l2;bub3;brsk1;cdc16;nusap1 |
| polysaccharide metabolic process; | 15#86 | 0.00101801 | gaa;phkg2;adra1b;aoah;chst1;ppp1cc;cercam;ppp1cb;prkag2;c1qtnf2;pygl;gsk3b;phka2;phkg1;cmas |
| peptidyl-tyrosine modification; | 11#44 | 0.001032316 | tpst1;jak1;prkca;hcls1;abi2;stat1;il3;socs1;il20;abl2;clcf1 |
| protein complex assembly; | 40#340 | 0.001141562 | ptk2;nck1;chaf1a;gja1;cd74;slc7a7;lin7a;eid2;copg2;il1rap;matn1;gga3;ap3s1;xpo6;aldh5a1;traf1;capza2;mki67ip;kpna3;gpaa1;tubgcp4;ctnna1;malt1;rad51;cda;picalm;ap1s3;c1qtnf2;napg;skap2;trpv5;ap1g1;ranbp9;cldn14;tmem48;nubp1;ap3d1;adrm1;capg;cct6b |
| axonogenesis; | 18#114 | 0.001281813 | nrp1;fez1;pou3f2;dscaml1;cyfip1;pou4f3;tgfb2;b3gnt2;slitrk4;slitrk3;efnb3;ngfr;cacna1f;sema4d;cck;nrcam;dcc;dlx5 |
| monocarboxylic acid metabolic process; | 35#287 | 0.001342225 | gnpat;acot9;acsm5;cd74;acaca;acadl;prkag2;alox12;aldh5a1;slc27a2;scd;elovl7;aloxe3;fa2h;lep;fbp2;hadha;ptges;slc25a10;acox2;prkaa1;mthfd1l;acoxl;cryl1;hacl1;ghsr;acsl3;c1qtnf2;ugdh;aldh1l1;hadh;slc27a4;acadsb;fasn;prkaa2 |
| cell division; | 31#245 | 0.00146785 | ccnd3;ncaph;evi5;anapc7;papd5;ccng1;tgfb2;hells;aurkc;cdk6;lats2;ndc80;stag1;rcc1;bub1b;cntrob;cables1;sycp3;mad2l2;aurkb;ppp1cc;pds5a;ppp1cb;nde1;sac3d1;sgol1;cdc16;rasa1;ncapg;ccnt2;nusap1 |
| second-messenger-mediated signaling; | 33#268 | 0.001702952 | adora2b;pth2r;edn1;cdh13;galr1;grm8;azu1;pcna;rgs1;ndc80;dgka;ltb4r;btk;npr3;cckbr;dgkb;dgkz;gpr3;htr4;gpr139;chrm2;gnai3;nmbr;gnal;ccr2;adra1b;bub1b;mtnr1b;agk;fen1;gabbr1;agtr1;mchr1 |
| blood vessel development; | 23#164 | 0.001708822 | nrp1;adora2b;vegfa;foxc1;edn1;cdh13;gja1;fgf6;ctnnb1;tgfb2;klf5;adra1b;runx1;fgf1;rhob;ihh;cdx2;krt1;flt1;gna13;serpinf1;jmjd6;rasa1 |
| ion transport; | 122#1364 | 0.001764816 | btbd10;c1qtnf3;slc17a4;kcnc1;atp13a5;nox1;galr1;fxyd5;kcnk2;slc22a4;asz1;slc20a1;kctd9;kcng1;slc16a3;svop;tst;heph;scn1a;cnnm2;kcnab3;slc13a1;cachd1;scnn1b;c1qc;kctd8;scara5;best4;slc31a1;kcnk1;kctd19;atp5o;vdac1;slc4a2;kcna2;trpm8;gabrb2;cacna2d3;kcnq1;cacng6;asna1;kcnip4;slc4a8;slc30a10;nedd4l;cacng7;chrna5;slc17a7;best1;slc9a8;slc30a4;scn1b;trpc7;slc39a6;atp6v1d;kcns1;wnt2;slc30a6;slc22a18;grid2;chrne;chrna4;kcnj13;kcns3;ccbe1;gja1;slc22a16;atp11b;kcnj12;kcnj11;c1qtnf1;slc5a3;slc30a9;cthrc1;cnga3;atp2c1;clic4;atp6v1g3;clcn5;atp6v0b;kcnv2;slc39a14;nalcn;cacna1f;rhcg;slc8a2;fxyd1;slco1b3;atp2a2;slc9a5;csn2;cacnb3;kctd18;slc30a1;trpv1;kcnk6;kcne1;col8a2;kctd12;atp5b;slc9a7;cacng3;kcnj5;sfxn3;c1qtnf2;slc10a5;kcnmb4;tmem37;colec11;trpv5;trpc5;slc38a4;pkd2l1;kcnq2;hcn4;kcnf1;atp2b3;slc23a2;tfrc;slc39a7;kcna5;nudt9 |
| glycogen metabolic process; | 11#47 | 0.001891913 | gaa;phkg2;adra1b;ppp1cc;ppp1cb;prkag2;c1qtnf2;pygl;gsk3b;phkg1;phka2 |
| regulation of catalytic activity; | 62#605 | 0.00195289 | adora2b;chml;rgs4;shc1;edn1;traf6;galr1;actn4;ccng1;ern1;cdkn2d;map3k9;cdkn3;grm8;tbc1d14;azu1;sfn;lats2;trib3;map3k7;map3k4;arhgap1;madd;nlrp2;dgka;cckbr;fgd1;spry1;pak1;abl2;ccnt2;map3k2;ccnd3;malt1;tspyl2;gab1;evi5;dgkb;prkca;taok2;prkaa1;cdc37;ecsit;dgkz;als2cr12;dusp22;bcl6;stat1;smap1;arfgap1;traf7;dbnl;gnai3;gnal;serinc1;c1qtnf2;ccr2;pdcd4;agk;gabbr1;rbl2;diras3 |
| intracellular receptor-mediated signaling pathway; | 12#55 | 0.001954247 | grip1;ncoa2;rbm14;ddx54;egln2;ppargc1b;ctnnb1;med24;ran;rnf14;dcbld2;pias1 |
| vasculature development; | 23#166 | 0.002159316 | nrp1;adora2b;vegfa;foxc1;edn1;cdh13;gja1;fgf6;ctnnb1;tgfb2;klf5;adra1b;runx1;fgf1;rhob;ihh;cdx2;krt1;flt1;gna13;serpinf1;jmjd6;rasa1 |
| glucan metabolic process; | 11#48 | 0.002272611 | gaa;phkg2;adra1b;ppp1cc;ppp1cb;prkag2;c1qtnf2;pygl;gsk3b;phkg1;phka2 |
| protein targeting; | 28#218 | 0.002286902 | tgfb1;taok2;kpna2;cdc37;tomm22;stat1;bcl6;pex16;kpna6;optn;ran;xpo6;synj2bp;rtp4;pex12;kpna3;ywhab;cep57;hspd1;gnptg;nfkbil1;srp54;srp72;prkci;atg4a;gsk3b;rtp3;nupl2 |
| amino acid and derivative metabolic process; | 57#548 | 0.002295709 | bhmt;maoa;ckm;srm;slc7a7;tfe3;ears2;gfpt1;tgfb2;aldh5a1;kynu;cbs;il20;th;nr4a2;tyr;bcat2;arg1;plod1;znfx1;agxt2l1;qdpr;mars2;ccnd3;wars;sms;uroc1;prkca;pepd;afmid;ssu72;farsa;wars2;il3;dio3;pycr1;hars;sars;cad;ddah1;hcls1;sncb;agmat;gch1;hibadh;nars;psph;socs1;gfpt2;pts;gmps;dbh;pnmt;bckdhb;clcf1;amd1;coro2a |
| blood vessel development#blood vessel morphogenesis; | 21#147 | 0.002374602 | nrp1;adora2b;vegfa;foxc1;edn1;cdh13;gja1;fgf6;ctnnb1;tgfb2;klf5;adra1b;runx1;fgf1;rhob;ihh;krt1;flt1;gna13;serpinf1;rasa1 |
| cytokine production#regulation of cytokine production; | 10#41 | 0.00251672 | malt1;map3k7;myd88;tlr2;traf6;bcl6;vsig4;elf1;ghsr;tgfb2 |
| nervous system development#neurogenesis; | 32#262 | 0.002529975 | nrp1;fez1;tgfb1;pou3f2;gja1;dscaml1;cln5;asz1;pou4f3;cyfip1;tgfb2;tlx3;azu1;b3gnt2;mdga1;cdk6;nr4a2;fzd9;slitrk4;slitrk3;efnb3;ngfr;cacna1f;cck;serpinf1;sema4d;wnt2;mdga2;dcc;nrcam;dlx5;ctnna1 |
| cell activation; | 27#209 | 0.00262283 | malt1;tgfb1;nck1;cd28;pram1;traf6;spaca3;cd74;ripk2;bcl6;pag1;cd2;hells;azu1;skap2;map3k7;cx3cl1;ap3d1;vsig4;cd93;hdac9;gp6;gna13;cd274;clcf1;gp1ba;slamf1 |
| regulation of interleukin-2 production; | 4#6 | 0.002717562 | malt1;map3k7;traf6;vsig4 |
| biogenic amine metabolic process; | 14#74 | 0.002736724 | sms;maoa;sncb;agmat;afmid;srm;dio3;tgfb2;dbh;pnmt;kynu;amd1;th;nr4a2 |
| aromatic compound catabolic process; | 7#21 | 0.002736724 | pts;afmid;gch1;qdpr;aldh5a1;kynu;ephx1 |
| ER-nuclear signaling pathway; | 7#21 | 0.002736724 | ern1;atp2a2;eif2ak2;vapb;pak1;gsk3b;scap |
| generation of neurons; | 30#242 | 0.002865419 | nrp1;fez1;tgfb1;pou3f2;gja1;dscaml1;cln5;asz1;pou4f3;cyfip1;tgfb2;tlx3;b3gnt2;mdga1;nr4a2;fzd9;slitrk4;slitrk3;efnb3;ngfr;cacna1f;cck;sema4d;serpinf1;wnt2;mdga2;dcc;nrcam;dlx5;ctnna1 |
| cellular macromolecule metabolic process; | 426#5656 | 0.002865419 | pigq;ttbk1;ptk2;fkbp3;ppil3;cfb;fbxo10;usp30;ears2;spcs2;myo3a;nrbp1;nsf;psmc2;mark2;dusp10;msrb3;prkra;map3k7;fut1;map3k4;fkbp6;cdk2ap1;c1qc;ctss;st13;cdc16;sumo1;glyctk;ctsa;cdk9;tspan8;chst14;fbxl17;rce1;tec;cdc42bpb;b3gnt4;il3;sqstm1;traf7;cdc42ep5;plod2;adam20;arih1;st3gal3;st6galnac4;ptpn18;phkg2;rbbp6;pstk;rnf139;pabpc4;prmt2;capg;klk12;aplp1;csnk1g3;rasa1;sirt3;eif3f;phka2;abcf1;cpa3;akt2;ern1;xpo6;uchl5;eif1b;mtmr7;mex3b;calr3;usp39;npr2;rpl8;cpxm1;ptpla;usp32;mars2;abl2;fbxl4;adam12;rbm14;dyrk1b;usp15;fbxl8;wars2;bace2;ppm1k;map4k2;eif4g2;aurkc;tgm7;pcsk7;dpp8;klk7;lonp1;sirt1;mrps18b;hectd2;mrpl3;f7;pja2;usp13;prkaa2;gsk3b;bhmt;nck1;trim54;traf6;ogt;psmd14;ero1lb;epha3;mrpl10;sdf2;lrrk1;lats2;tbcc;trib3;myd88;serpina5;uhrf2;dnajc17;cdc14b;fbxl22;hdac10;ubqln4;st6gal1;hsp90aa1;phkg1;b3galt5;ccnd3;wars;prkca;l2hgdh;mmp9;tmub2;cpa4;ppm1d;senp2;senp3;adam10;b3gnt2;sugt1;tinag;adam21;grpel2;tmem48;camk4;nubp1;cpb1;c4bpb;socs1;b3galt1;usp11;snrk;wnt2;clk3;ddb1;ube2m;oxsr1;gaa;mrpl36;fbxo45;araf;ripk2;eid2;tfe3;fbxo9;azu1;il20;fbxl13;lrpap1;st3gal1;itk;lonrf1;ctsw;ppie;rps8;tsfm;ppic;ihh;mrps9;igf2bp3;zbtb16;lrrc14;rnf14;cmas;fbxw8;malt1;prkaa1;eif2ak2;abi2;map4k3;mmp12;hars;srpk1;cdk6;trpc5;pum2;hcls1;bub1b;ero1l;aurkb;pja1;rnf2;senp8;dph1;pygl;ift88;tpst1;klk13;hunk;prepl;mrpl30;mrps18c;ict1;asz1;cpa5;map3k9;prkag2;asb2;rapgef4;camk2d;supt16h;apeh;capza3;chst8;gpaa1;sh3rf2;hpse;adprh;taok2;pepd;cdkn2aip;nfatc2ip;casp7;tlk1;vrk1;pdk2;pim1;sars;samd4a;ddr2;mttp;zdhhc7;b3galt2;galnt6;ptpn21;tgm5;ulk3;tnp1;tnks;qpctl;senp1;herc6;ccnb1ip1;eif4h;ttll5;aipl1;pigu;man1a1;cfi;atg7;rabggta;eef1d;ppp4r2;mrpl27;rbck1;apobec1;med8;mrpl22;chst12;nlk;plod1;crim1;galnt9;sirt5;tor2a;fut3;syvn1;znfx1;gnptg;ppp1cb;pam;eif1ax;prkci;pak1;tp53rk;map3k2;axl;tgfb1;adam32;mapk1;gcnt4;mid1;pld2;dusp22;map2k7;lcmt1;acvr2b;ace2;slmap;klhl15;ppm1l;ghsr;rps29;mark1;cdkl2;gucy2c;dusp13;klk9;fbxo38;slc38a4;adra1b;adrm1;opn1sw;cct6b;tubg1;ppp1cc;mgat4a;stk39;clcf1;eif5;cnot4;rnf41;ubtd2;nek8;aurkaip1;anapc7;hus1;pdia2;tysnd1;tnfaip3;scrn1;twf1;capza2;asphd1;lrrc41;mrpl16;b3gnt5;camk2g;plaur;hdac9;flt1;btk;usp2;cpa6;psma8;mapkapk5;rps3;hspb1;scarf1;ncln;taok3;rps10;farsa;cndp2;il17a;nedd4l;csnk1a1;rplp1;mrpl37;oma1;eef1e1;pias1;set;pak3;psmb5;alg3;tnip1;htra1;cercam;hspd1;usp33;cdk10;st3gal4;cog2;lrrc29;ttll9;sirt4;cd28;pigs;pigb;rad23a;mrpl47;wee2;hectd3;a4gnt;aoah;gan;prtn3;epha1;dmpk;krt1;rpl9;eif3e;olr1;srms;atg4a;pskh1;scap;ttc1;ubqln2;lman1;jak1;tubb6;rsl1d1;stat1;atg12;fbxo47;brsk1;hk3;denr;c1qtnf2;psmc5;napg;nuak1;eif3i;nars;capn2;mrpl35;st8sia5;dnaja4;fbxl6 |
| regulation of cell adhesion; | 13#66 | 0.002943212 | tgfbi;cx3cl1;cdh13;arhgdia;bcl6;arhgdib;fxyd5;adam10;alox12;azu1;kng1;rasa1;cdk6 |
| peptidyl-tyrosine phosphorylation; | 10#42 | 0.002974412 | jak1;prkca;hcls1;abi2;stat1;il3;socs1;il20;abl2;clcf1 |
| excretion; | 10#42 | 0.002974412 | adora2b;clcn5;aqp1;edn1;scnn1b;adm;nedd4l;kng1;slc22a18;ift88 |
| leukocyte activation; | 24#180 | 0.003193128 | malt1;tgfb1;nck1;cd28;pram1;traf6;spaca3;cd74;ripk2;bcl6;pag1;cd2;hells;azu1;skap2;map3k7;cx3cl1;ap3d1;vsig4;cd93;hdac9;cd274;clcf1;slamf1 |
| response to stimulus#regulation of response to stimulus; | 9#35 | 0.003203988 | prkca;lep;aoah;cx3cl1;bcl6;ghsr;gpx4;il20;eef1e1 |
| microtubule cytoskeleton organization and biogenesis; | 16#102 | 0.003403154 | ranbp9;trim54;ndc80;bub1b;rcc1;mid1;tubg1;tubgcp5;asz1;ppp4c;tubgcp4;tubgcp3;ran;wnt2;hook3;nusap1 |
| lipid biosynthetic process; | 38#333 | 0.003434148 | pigq;pigu;cd74;acaca;pigs;pigb;prkag2;alox12;scd;elovl7;srd5a1;cnbp;aloxe3;fa2h;sc5dl;b3gnt5;ugcg;gpaa1;fdxr;acly;mvd;pnpla3;scap;cmas;fdps;pdss2;nr5a1;prkaa1;cept1;cds1;stard5;pdss1;adm;impa1;cercam;st8sia5;fasn;prkaa2 |
| G-protein signaling, coupled to cAMP nucleotide second messenger; | 14#84 | 0.003450641 | ccr2;rgs1;adora2b;pth2r;adra1b;gpr3;galr1;chrm2;npr3;gnai3;grm8;gnal;gabbr1;mchr1 |
| cellular polysaccharide metabolic process; | 14#84 | 0.003450641 | gaa;phkg2;adra1b;aoah;ppp1cc;cercam;ppp1cb;prkag2;c1qtnf2;pygl;gsk3b;phka2;phkg1;cmas |
| extracellular structure organization and biogenesis; | 14#84 | 0.003450641 | pcdhb11;pcdhb6;mmp9;mia;foxc1;pcdhb5;ctnnb1;aplp1;col8a2;ghsr;drp2;tgfb2;nrcam;elf3 |
| vascular process in circulatory system; | 10#43 | 0.003538634 | adra1b;gch1;foxc1;edn1;asz1;nts;wnt2;azu1;kng1;kcnmb4 |
| enzyme linked receptor protein signaling pathway; | 35#300 | 0.003579243 | ptk2;nck1;vegfa;smad6;gfra2;shc1;foxc1;cdh13;eid2;epha3;ap3s1;fstl3;map3k7;epha1;flt1;cnksr1;tgfb1;prkca;gab1;jak1;acvr2b;ghsr;gucy2c;rapgef1;sparc;grb10;ddr2;cited2;htra1;efnb3;cep57;socs1;gp6;fgf5;timp3 |
| steroid hormone receptor signaling pathway; | 11#51 | 0.003616132 | grip1;ncoa2;rbm14;ddx54;egln2;ppargc1b;ctnnb1;med24;ran;rnf14;pias1 |
| hemopoiesis; | 22#161 | 0.003625335 | tgfb1;mmp9;vps33a;cd74;bcl6;ctnnb1;il3;cd2;map4k2;tgfb2;cdk6;hcls1;runx1;lep;kitlg;bpgm;ap3d1;klf1;hdac9;snrk;zbtb16;clcf1 |
| nervous system development#central nervous system development; | 29#235 | 0.003857386 | msx1;utp3;pou3f2;shroom2;foxc1;npas1;ctnnb1;dscaml1;cln5;asz1;mbp;pcdh18;egr2;tlx3;aldh5a1;mdga1;chst8;b3gnt5;alx1;pdgfc;ngfr;dlx2;aplp1;pts;drp2;wnt2;mdga2;zbtb16;nrcam |
| cartilage development; | 9#36 | 0.003858112 | pth2r;msx1;edn1;snai1;alx1;ihh;sox9;ctnnb1;matn1 |
| patterning of blood vessels; | 5#11 | 0.003926798 | ctnnb1;flt1;gna13;edn1;ihh |
| muscle development; | 23#172 | 0.003926798 | tgfb1;gaa;msx1;nras;adam12;foxc1;gja1;fgf6;myog;myl1;sgca;myl4;sox6;sgcg;sox15;sgcb;bves;sirt1;hdac9;ngfr;itgb1bp2;ankrd2;fhl1 |
| defense response; | 59#584 | 0.003931339 | tpst1;adora2b;cd14;cfi;gpr68;spaca3;ripk2;cfb;rsad2;il1rap;foxn1;lyz;ccr7;azu1;kng1;elf3;il20;hamp;itk;myd88;tff3;aoah;nlrp2;cx3cl1;prdx5;ltb4r;krt1;hdac9;hpse;c1qc;olr1;sarm1;pglyrp3;malt1;tgfb1;procr;defb123;prkca;tlr2;ecsit;apoh;cxcl11;bcl6;il17a;ghsr;gpx4;cst11;ccr2;clec1a;stat3;il13;tnip1;chst1;lalba;c4bpb;cxcl14;tff1;ccr9;abcf1 |
| aromatic compound metabolic process; | 24#183 | 0.00420204 | maoa;mapk1;afmid;ephx1;mthfd1l;cda;tgfb2;paics;aldh5a1;kynu;th;aldh1l1;nr4a2;tyr;cad;gphn;sncb;gch1;pts;gmps;dbh;qdpr;pnmt;aprt |
| developmental maturation; | 11#53 | 0.004984501 | pth2r;adam12;gja1;fzd5;kcne1;klf1;ihh;ctnnb1;cln5;tgfb2;nrcam |
| cell maturation; | 10#45 | 0.005016402 | pth2r;adam12;gja1;fzd5;kcne1;klf1;ihh;ctnnb1;cln5;nrcam |
| multicellular organismal development#pattern specification process; | 18#124 | 0.005037247 | meox2;mid1;edn1;sfrp2;sim2;ctnnb1;acvr2b;dscaml1;hoxd8;kif3b;cited2;ripply1;ihh;cdx2;flt1;gna13;spry1;ift88 |
| regulation of cell differentiation; | 20#144 | 0.005165724 | pou3f2;mapk1;bcl6;ctnnb1;keap1;tgfb2;tlx3;il20;cdk6;crx;runx1;lep;iqcb1;ihh;maff;pparg;zbtb16;nrcam;dlx5;nanog |
| cellular protein metabolic process; | 418#5584 | 0.00520921 | pigq;ttbk1;ptk2;fkbp3;ppil3;cfb;fbxo10;usp30;ears2;spcs2;myo3a;nrbp1;nsf;psmc2;mark2;dusp10;msrb3;prkra;map3k7;fut1;map3k4;fkbp6;cdk2ap1;c1qc;ctss;st13;cdc16;sumo1;glyctk;ctsa;cdk9;tspan8;chst14;fbxl17;rce1;tec;cdc42bpb;b3gnt4;il3;sqstm1;traf7;cdc42ep5;plod2;adam20;arih1;st3gal3;st6galnac4;ptpn18;phkg2;rbbp6;pstk;rnf139;pabpc4;prmt2;capg;klk12;aplp1;csnk1g3;rasa1;sirt3;eif3f;phka2;abcf1;cpa3;akt2;ern1;xpo6;uchl5;eif1b;mtmr7;mex3b;calr3;usp39;npr2;rpl8;cpxm1;ptpla;usp32;mars2;abl2;fbxl4;adam12;rbm14;dyrk1b;usp15;fbxl8;wars2;bace2;ppm1k;map4k2;eif4g2;aurkc;tgm7;pcsk7;dpp8;klk7;lonp1;sirt1;mrps18b;hectd2;mrpl3;f7;pja2;usp13;prkaa2;gsk3b;bhmt;nck1;trim54;traf6;ogt;psmd14;ero1lb;epha3;mrpl10;sdf2;lrrk1;lats2;tbcc;trib3;myd88;serpina5;uhrf2;dnajc17;cdc14b;fbxl22;hdac10;ubqln4;st6gal1;hsp90aa1;phkg1;b3galt5;ccnd3;wars;prkca;l2hgdh;mmp9;tmub2;cpa4;ppm1d;senp2;senp3;adam10;b3gnt2;sugt1;tinag;adam21;grpel2;tmem48;camk4;nubp1;cpb1;c4bpb;socs1;b3galt1;usp11;snrk;wnt2;clk3;ddb1;ube2m;oxsr1;mrpl36;fbxo45;araf;ripk2;eid2;tfe3;fbxo9;azu1;il20;fbxl13;lrpap1;st3gal1;itk;lonrf1;ctsw;ppie;rps8;tsfm;ppic;ihh;mrps9;igf2bp3;zbtb16;lrrc14;rnf14;fbxw8;malt1;prkaa1;eif2ak2;abi2;map4k3;mmp12;hars;srpk1;cdk6;trpc5;pum2;hcls1;bub1b;ero1l;aurkb;pja1;rnf2;senp8;dph1;ift88;tpst1;klk13;hunk;prepl;mrpl30;mrps18c;ict1;asz1;cpa5;map3k9;asb2;rapgef4;camk2d;supt16h;apeh;capza3;chst8;gpaa1;sh3rf2;hpse;adprh;taok2;pepd;cdkn2aip;nfatc2ip;casp7;tlk1;vrk1;pdk2;pim1;sars;samd4a;ddr2;mttp;zdhhc7;b3galt2;galnt6;ptpn21;tgm5;ulk3;tnp1;tnks;qpctl;senp1;herc6;ccnb1ip1;eif4h;ttll5;aipl1;pigu;man1a1;cfi;atg7;rabggta;eef1d;ppp4r2;mrpl27;rbck1;apobec1;med8;mrpl22;chst12;nlk;plod1;crim1;galnt9;sirt5;tor2a;fut3;syvn1;znfx1;gnptg;ppp1cb;pam;eif1ax;prkci;pak1;tp53rk;map3k2;tgfb1;axl;adam32;mapk1;gcnt4;mid1;pld2;dusp22;map2k7;lcmt1;acvr2b;ace2;slmap;klhl15;ppm1l;ghsr;rps29;mark1;cdkl2;gucy2c;dusp13;klk9;fbxo38;slc38a4;adrm1;opn1sw;cct6b;tubg1;ppp1cc;mgat4a;stk39;clcf1;eif5;cnot4;rnf41;ubtd2;nek8;aurkaip1;anapc7;hus1;pdia2;tysnd1;tnfaip3;scrn1;twf1;capza2;asphd1;lrrc41;mrpl16;b3gnt5;camk2g;hdac9;flt1;plaur;btk;usp2;cpa6;psma8;mapkapk5;rps3;hspb1;scarf1;ncln;taok3;rps10;farsa;cndp2;il17a;nedd4l;csnk1a1;rplp1;mrpl37;oma1;eef1e1;pias1;set;pak3;psmb5;alg3;tnip1;htra1;hspd1;usp33;cdk10;st3gal4;cog2;lrrc29;ttll9;sirt4;cd28;pigs;pigb;rad23a;mrpl47;wee2;hectd3;a4gnt;gan;prtn3;epha1;krt1;dmpk;rpl9;eif3e;olr1;srms;atg4a;pskh1;scap;ttc1;ubqln2;lman1;jak1;tubb6;rsl1d1;stat1;atg12;fbxo47;brsk1;hk3;denr;psmc5;napg;nuak1;eif3i;nars;capn2;mrpl35;st8sia5;dnaja4;fbxl6 |
| microtubule nucleation; | 4#7 | 0.005320764 | ranbp9;tubgcp4;tubgcp3;tubgcp5 |
| nitrogen compound catabolic process; | 15#96 | 0.005435058 | bcat2;ddah1;arg1;uroc1;maoa;agmat;afmid;gch1;dio3;pts;dbh;qdpr;kynu;bckdhb;tst |
| cAMP-mediated signaling; | 14#87 | 0.005615014 | ccr2;rgs1;adora2b;pth2r;adra1b;gpr3;galr1;chrm2;npr3;gnai3;grm8;gnal;gabbr1;mchr1 |
| cholesterol metabolic process; | 13#78 | 0.005615014 | cnbp;fdps;ldlrap1;prkaa1;insig1;soat1;lep;nr0b2;fdxr;prkag2;mvd;prkaa2;scap |
| generation of neurons#neuron differentiation#neuron development#neurite development; | 19#135 | 0.005737913 | nrp1;fez1;pou3f2;gja1;dscaml1;cyfip1;pou4f3;tgfb2;b3gnt2;slitrk4;slitrk3;efnb3;ngfr;cacna1f;sema4d;cck;nrcam;dcc;dlx5 |
| G-protein signaling, coupled to IP3 second messenger (phospholipase C activating); | 14#88 | 0.00662102 | pth2r;dgkb;dgkz;edn1;dgka;gpr139;agk;ltb4r;chrm2;npr3;cckbr;nmbr;azu1;agtr1 |
| JAK-STAT cascade; | 9#39 | 0.006866603 | ccr2;pigu;stat3;hcls1;stat1;socs1;pias1;il20;clcf1 |
| positive regulation of cell differentiation; | 10#47 | 0.006950379 | crx;lep;runx1;ihh;ctnnb1;pparg;tgfb2;nrcam;il20;dlx5 |
| biosynthetic process; | 198#2454 | 0.007115041 | pigq;mrps18c;mrpl30;ckm;ict1;acaca;ears2;prkag2;qprt;grm8;tyr;bcat2;gphn;fut1;sucla2;chst8;gpaa1;fdxr;ccbl2;mmab;atp5o;tspan8;pdss2;nr5a1;b3gnt4;naprt1;cds1;sars;st3gal3;samd4a;st6galnac4;phkg2;zdhhc7;mttp;pstk;b3galt2;pabpc4;ap3d1;galnt6;prps2;lalba;psph;aplp1;eif4h;eif3f;aipl1;abcf1;pigu;man1a1;edn1;atg7;rabggta;eef1d;mrpl27;paics;kynu;mrpl22;chst12;fech;cnbp;eif1b;aloxe3;plod1;atp6v0b;npr2;galnt9;rpl8;fut3;znfx1;gnptg;eif1ax;mars2;ampd3;dhrs9;fdps;gcnt4;bace2;wars2;ghsr;eif4g2;rps29;atp5b;gucy2c;mrps35;ugdh;cad;slc38a4;pdss1;mrps18b;gfpt2;mgat4a;mrpl3;eif5;amd1;prkaa2;coro2a;uap1;bhmt;ogt;srm;alox12;mrpl10;sdf2;ucn2;elovl7;srd5a1;mrpl16;fa2h;myd88;sc5dl;b3gnt5;ugcg;st6gal1;hsp90aa1;mvd;pnpla3;phkg1;rps3;b3galt5;wars;slc25a10;hspb1;mthfd1l;rps10;farsa;il17a;cept1;rplp1;mrpl37;b3gnt2;eef1e1;stard5;gmppa;alg3;tnip1;agmat;adm;atp6v1d;b3galt1;cercam;st3gal4;cog2;mrpl36;cd28;pank4;cd74;pigs;pigb;tfe3;gfpt1;mrpl47;tgfb2;azu1;cbs;scd;th;st3gal1;atp6v1g3;ampd1;pgd;a4gnt;rps8;tsfm;mrps9;igf2bp3;fbp2;rpl9;eif3e;acly;qdpr;adk;scap;cmas;prkaa1;eif2ak2;papss1;rsl1d1;dio3;pycr1;gmppb;hars;denr;c1qtnf2;aldh1l1;eif3i;trpc5;pum2;gch1;nars;impa1;pts;gmps;mrpl35;dbh;pnmt;st8sia5;dph1;fasn |
| immune response#regulation of immune response; | 15#98 | 0.007310661 | malt1;tgfb1;map3k7;myd88;tlr2;cfi;cacnb3;traf6;bcl6;cfb;c4bpb;krt1;elf1;c1qc;tgfb2 |
| cholesterol biosynthetic process; | 7#25 | 0.007698004 | cnbp;fdps;prkag2;prkaa1;mvd;prkaa2;scap |
| regulation of defense response; | 7#25 | 0.007698004 | prkca;ghsr;gpx4;aoah;cx3cl1;il20;bcl6 |
| inflammatory response#regulation of inflammatory response; | 7#25 | 0.007698004 | prkca;ghsr;gpx4;aoah;cx3cl1;il20;bcl6 |
| calcium-dependent cell-cell adhesion; | 7#25 | 0.007698004 | fxyd5;pcdhb11;pcdhb6;atp2c1;pcdhb7;cdh13;pcdhb5 |
| regulation of I-kappaB kinase/NF-kappaB cascade; | 16#108 | 0.007711213 | sectm1;malt1;ltbr;traf6;gja1;ripk2;eef1d;lgals9;slc20a1;sqstm1;tnfaip3;atp2c1;myd88;trim38;traf3ip2;trim13 |
| cellular component assembly; | 76#813 | 0.007737595 | brf2;ptk2;h2afz;nck1;lin7a;matn1;gga3;med23;aldh5a1;capza2;gpaa1;tspyl1;cdk9;hspb1;sf3a2;ap1s3;set;tbpl2;ap1g1;ranbp9;cldn14;tmem48;nubp1;taf4;ap3d1;capg;tnp1;eif4h;sirt3;eif3f;crnkl1;sirt4;chaf1a;cd74;gja1;nap1l5;slc7a7;il1rap;copg2;eid2;ap3s1;xpo6;traf1;snrpe;eif1b;mki67ip;usp39;kpna3;sirt5;med24;taf6;eif3e;gtf2a1;eif1ax;tubgcp4;ctnna1;malt1;tspyl2;rad51;cda;picalm;eif4g2;hells;c1qtnf2;denr;napg;skap2;eif3i;trpv5;prpf6;adrm1;cct6b;sycp3;sirt1;eif5;gtf2e1 |
| regulation of cell activation; | 13#80 | 0.007788239 | malt1;tgfb1;map3k7;nck1;cd28;traf6;spaca3;cd74;bcl6;vsig4;cd2;pag1;cd274 |
| response to biotic stimulus; | 33#289 | 0.007919105 | cd14;spaca3;cfb;rsad2;ern1;lyz;thbd;azu1;hspa1l;prkra;hamp;myd88;hspa4;hsp90aa1;tusc5;pglyrp3;tgfb1;hspb1;defb123;eif2ak2;tlr2;mapk1;stat1;hsph1;znf175;cst11;isg20;ifi35;crebzf;vapb;lalba;hspd1;gsk3b |
| cell-cell adhesion; | 38#347 | 0.008207597 | pcdhga2;cdh13;ctnnb1;fxyd5;pcdh18;matn1;cd2;dsc2;ncam2;cldn2;cldn1;atp2c1;cdh16;cx3cl1;icam5;cd34;cd93;cdh12;cldn17;cadm3;nrcam;pvrl2;bcl6;pcdhb5;sox9;col8a2;ctnna3;pcdhb6;cldn14;pcdhb11;cd164;cldn20;alx1;pcdh1;snai1;cercam;pcdhb7;rasa1 |
| potassium ion transport; | 30#256 | 0.008324574 | btbd10;kcna2;kcns3;kctd18;kcnj13;kcnq1;kcnc1;kcnk6;kcne1;kcnj12;kcnk2;kctd12;kcnip4;kcnj11;kctd9;slc9a7;kcnj5;kcng1;kcnmb4;cnga3;kcnq2;kcnv2;hcn4;kcnab3;kcnf1;kcna5;kcns1;kctd8;kcnk1;kctd19 |
| positive regulation of catalytic activity; | 30#256 | 0.008324574 | ccnd3;adora2b;malt1;gab1;dgkb;taok2;prkaa1;dgkz;shc1;edn1;traf6;stat1;ern1;traf7;dbnl;map3k9;gnal;serinc1;azu1;c1qtnf2;map3k7;map3k4;madd;dgka;nlrp2;agk;cckbr;pak1;abl2;map3k2 |
| induction of apoptosis; | 26#212 | 0.008336211 | tgfb1;prkca;cd70;mapk1;tlr2;tnfrsf25;traf3;sap30bp;ern1;cd2;pdcd5;dedd;sfn;prkra;tp53bp2;gch1;bclaf1;plekhf1;lalba;ngfr;btk;bik;plagl2;bcl2l11;dcc;runx3 |
| positive regulation of JNK cascade; | 5#13 | 0.008780037 | il3;taok2;myd88;eda2r;taok3 |
| regulation of JNK cascade; | 6#19 | 0.008836541 | il3;taok2;myd88;eda2r;taok3;mapk8ip1 |
| I-kappaB kinase/NF-kappaB cascade; | 19#139 | 0.008863238 | sectm1;malt1;ltbr;traf6;gja1;ripk2;stat1;eef1d;lgals9;slc20a1;sqstm1;tnfaip3;atp2c1;map3k7;myd88;trim38;traf3ip2;snip1;trim13 |
| phosphoinositide-mediated signaling; | 18#129 | 0.008922957 | pth2r;dgkb;dgkz;edn1;gpr139;chrm2;nmbr;azu1;pcna;ndc80;bub1b;dgka;ltb4r;agk;npr3;fen1;cckbr;agtr1 |
| sterol biosynthetic process; | 8#33 | 0.008938435 | cnbp;fdps;prkaa1;sc5dl;prkag2;mvd;prkaa2;scap |
| induction of programmed cell death; | 26#213 | 0.00895728 | tgfb1;prkca;cd70;mapk1;tlr2;tnfrsf25;traf3;sap30bp;ern1;cd2;pdcd5;dedd;sfn;prkra;tp53bp2;gch1;bclaf1;plekhf1;lalba;ngfr;btk;bik;plagl2;bcl2l11;dcc;runx3 |
| locomotory behavior; | 23#181 | 0.009167338 | ccr8;cstb;prkca;mapk1;vegfa;cdh13;cxcl16;cxcl11;ccr7;tgfb2;azu1;ccl28;ccr2;adra1b;creb3;cx3cl1;enpp2;efnb3;plaur;cxcl14;hexa;ccr9;ccrl1 |
| regulation of immune system process; | 15#100 | 0.009303894 | malt1;tgfb1;map3k7;myd88;tlr2;cfi;cacnb3;traf6;bcl6;cfb;c4bpb;krt1;elf1;c1qc;tgfb2 |
| catecholamine metabolic process; | 7#26 | 0.009332296 | maoa;tgfb2;sncb;pnmt;dbh;th;nr4a2 |
| negative regulation of gene expression, epigenetic; | 7#26 | 0.009332296 | sirt1;tnp1;sirt4;hells;crebzf;sirt3;sirt5 |
| GO:0002726,positive regulation of T cell cytokine production; | 3#4 | 0.009332296 | malt1;map3k7;traf6 |
| glucosylceramide metabolic process; | 3#4 | 0.009332296 | prkaa1;aldh5a1;ugcg |
| positive regulation of T cell mediated immunity; | 3#4 | 0.009332296 | malt1;map3k7;traf6 |
| positive regulation of interleukin-2 production; | 3#4 | 0.009332296 | malt1;map3k7;traf6 |
| transport#regulation of transport; | 16#110 | 0.009522797 | tgfb1;prkca;pram1;cdh13;spaca3;gja1;actn4;traf7;nedd4l;azu1;c1qtnf2;synj2bp;ldlrap1;scnn1b;nfkbil1;gsk3b |
| RNA splicing; | 27#225 | 0.009522797 | pnn;syncrip;sf3a2;nhp2l1;rragc;sfpq;zmat5;ppil3;dhx15;ppp4r2;zcrb1;srpk1;tfip11;dhx38;snrpe;cdc40;rnpc3;hnrnpr;sf3b2;ppie;usp39;rbm17;prpf6;scnm1;skiv2l2;rnps1;crnkl1 |
| response to radiation; | 15#101 | 0.010492466 | fech;nras;adra1b;hus1;ppm1d;opn1sw;cops3;cacna1f;cdkn2d;fen1;brsk1;clock;thbd;rad54l;pdc |
| endocytosis#regulation of endocytosis; | 8#34 | 0.01056913 | tgfb1;synj2bp;ldlrap1;nedd4l;azu1;cdh13;spaca3;actn4 |
| multicellular organismal development#tube development#tube morphogenesis#regulation of tube size; | 9#42 | 0.01076251 | adra1b;gch1;foxc1;edn1;asz1;nts;wnt2;kng1;kcnmb4 |
| regulation of blood vessel size; | 9#42 | 0.01076251 | adra1b;gch1;foxc1;edn1;asz1;nts;wnt2;kng1;kcnmb4 |
| positive regulation of developmental process; | 12#68 | 0.010998194 | crx;lep;runx1;ihh;ctnnb1;pparg;tgfb2;sema4d;serpinf1;nrcam;il20;dlx5 |
| hemopoietic or lymphoid organ development; | 22#173 | 0.011399836 | tgfb1;mmp9;vps33a;cd74;bcl6;ctnnb1;il3;cd2;map4k2;tgfb2;cdk6;hcls1;runx1;lep;kitlg;bpgm;ap3d1;klf1;hdac9;snrk;zbtb16;clcf1 |
| phenol metabolic process; | 7#27 | 0.011399836 | maoa;tgfb2;sncb;pnmt;dbh;th;nr4a2 |
| adult behavior; | 7#27 | 0.011399836 | cstb;ghsr;adra1b;hexa;lep;brs3;efnb3 |
| amino acid derivative catabolic process; | 5#14 | 0.011948218 | dio3;maoa;afmid;aldh5a1;kynu |
| cellular metal ion homeostasis; | 17#123 | 0.013830458 | ccr8;pth2r;atp2a2;tgfb1;prkca;edn1;ccr7;nedd4l;ccl28;kng1;ccr2;atp2c1;cacna1f;cckbr;mchr1;agtr1;ccr9 |
| metal ion homeostasis; | 17#123 | 0.013830458 | ccr8;pth2r;atp2a2;tgfb1;prkca;edn1;ccr7;nedd4l;ccl28;kng1;ccr2;atp2c1;cacna1f;cckbr;mchr1;agtr1;ccr9 |
| activation of protein kinase activity; | 12#70 | 0.013915958 | malt1;map3k7;map3k4;dgkb;taok2;dgkz;edn1;traf6;dgka;agk;traf7;azu1 |
| generation of neurons#neuron differentiation#neuron development; | 20#154 | 0.013915958 | nrp1;fez1;pou3f2;gja1;dscaml1;cln5;cyfip1;pou4f3;tgfb2;b3gnt2;slitrk4;slitrk3;efnb3;ngfr;cacna1f;sema4d;cck;dcc;nrcam;dlx5 |
| chromatin modification; | 26#219 | 0.014094773 | utp3;tspyl2;vps72;rbm14;sirt4;npm2;cpa4;tlk1;hells;epc2;set;setdb2;supt4h1;chd3;ing3;sirt5;pbrm1;sycp3;klf1;sirt1;hdac9;tnp1;hdac10;dot1l;sirt3;rbl2 |
| retrograde transport, endosome to Golgi; | 4#9 | 0.014159458 | gosr1;vps26a;stx16;ykt6 |
| regulation of production of molecular mediator of immune response; | 4#9 | 0.014159458 | malt1;map3k7;traf6;bcl6 |
| Rac protein signal transduction; | 4#9 | 0.014159458 | elmo1;dbnl;nras;cdh13 |
| cytokine production during immune response; | 4#9 | 0.014159458 | malt1;map3k7;traf6;bcl6 |
| regulation of cytokine production during immune response; | 4#9 | 0.014159458 | malt1;map3k7;traf6;bcl6 |
| macrophage activation; | 4#9 | 0.014159458 | azu1;spaca3;cd74;cd93 |
| response to abiotic stimulus; | 21#165 | 0.014159458 | sord;nras;gja1;hus1;ppm1d;cops3;npffr2;cdkn2d;egr2;brsk1;thbd;rad54l;fech;adra1b;opn1sw;ero1l;ngfr;cacna1f;fen1;clock;pdc |
| chemotaxis; | 19#144 | 0.014159458 | ccr8;prkca;mapk1;vegfa;cdh13;cxcl16;cxcl11;ccr7;tgfb2;azu1;ccl28;ccr2;creb3;cx3cl1;enpp2;plaur;cxcl14;ccr9;ccrl1 |
| taxis; | 19#144 | 0.014159458 | ccr8;prkca;mapk1;vegfa;cdh13;cxcl16;cxcl11;ccr7;tgfb2;azu1;ccl28;ccr2;creb3;cx3cl1;enpp2;plaur;cxcl14;ccr9;ccrl1 |
| nuclear export; | 9#44 | 0.014268555 | malt1;rae1;gle1;ran;nxf1;xpo6;ddx19b;gsk3b;nupl2 |
| protein amino acid autophosphorylation; | 9#44 | 0.014268555 | mex3b;nlk;eif2ak2;taok3;asz1;map3k9;myo3a;wnt2;pak1 |
| sterol metabolic process; | 14#94 | 0.014301911 | cnbp;fdps;ldlrap1;prkaa1;sc5dl;insig1;soat1;lep;nr0b2;fdxr;prkag2;mvd;prkaa2;scap |
| amine catabolic process; | 14#94 | 0.014301911 | bcat2;ddah1;arg1;uroc1;maoa;agmat;afmid;gch1;dio3;pts;dbh;qdpr;kynu;bckdhb |
| activation of immune response; | 12#71 | 0.015067374 | malt1;map3k7;myd88;tlr2;cfi;cacnb3;traf6;cfb;c4bpb;krt1;elf1;c1qc |
| protein amino acid lipidation; | 10#53 | 0.01516581 | pigq;pigu;zdhhc7;mttp;atg7;gpaa1;rabggta;pigs;pigb;aipl1 |
| chromatin remodeling; | 11#62 | 0.015339209 | sirt4;supt4h1;npm2;sirt5;pbrm1;klf1;sycp3;sirt1;tnp1;hells;sirt3 |
| positive regulation of immune response; | 13#85 | 0.015380572 | malt1;map3k7;myd88;tlr2;cfi;cacnb3;traf6;cfb;c4bpb;krt1;elf1;c1qc;tgfb2 |
| negative regulation of adenylate cyclase activity; | 5#15 | 0.015633824 | ccr2;gnai3;grm8;gabbr1;galr1 |
| negative regulation of cyclase activity; | 5#15 | 0.015633824 | ccr2;gnai3;grm8;gabbr1;galr1 |
| regulation of JAK-STAT cascade; | 5#15 | 0.015633824 | socs1;pigu;hcls1;clcf1;il20 |
| positive regulation of cell adhesion; | 5#15 | 0.015633824 | alox12;azu1;cx3cl1;cdh13;cdk6 |
| negative regulation of lyase activity; | 5#15 | 0.015633824 | ccr2;gnai3;grm8;gabbr1;galr1 |
| protein amino acid phosphorylation; | 101#1167 | 0.016394637 | ttbk1;ptk2;hunk;nek8;hus1;asz1;myo3a;map3k9;nrbp1;epha3;mark2;rapgef4;lrrk1;twf1;camk2d;lats2;prkra;trib3;map3k7;map3k4;camk2g;cdk2ap1;flt1;btk;mapkapk5;phkg1;glyctk;ccnd3;cdk9;prkca;taok2;tec;cdc42bpb;taok3;tlk1;il3;adam10;vrk1;pdk2;csnk1a1;pim1;pak3;phkg2;ddr2;camk4;ulk3;socs1;cdk10;tnks;csnk1g3;snrk;wnt2;clk3;oxsr1;araf;akt2;ripk2;ern1;wee2;il20;mex3b;nlk;itk;npr2;epha1;dmpk;prkci;srms;pak1;abl2;tp53rk;pskh1;map3k2;tgfb1;axl;jak1;mapk1;prkaa1;eif2ak2;abi2;dyrk1b;stat1;map2k7;acvr2b;map4k3;brsk1;map4k2;mark1;cdkl2;gucy2c;srpk1;cdk6;nuak1;aurkc;hcls1;bub1b;aurkb;stk39;clcf1;prkaa2;gsk3b |
| tissue remodeling; | 15#105 | 0.016505145 | tfip11;pth2r;tgfb1;adra1b;minpp1;foxc1;amtn;ihh;ctnnb1;ostf1;tgfb2;sparc;elf3;cdk6;dlx5 |
| positive regulation of cell proliferation; | 27#233 | 0.016515913 | nrp1;pth2r;tgfb1;pou3f2;nck1;nras;cd28;vegfa;shc1;edn1;cdh13;bcl6;il3;htr1a;tgfb2;alox12;cdk6;klf5;ddr2;myd88;hcls1;adm;ihh;prtn3;flt1;cckbr;slamf1 |
| response to drug; | 11#63 | 0.017014252 | sord;tgfb1;abcg2;tlr2;snx27;asz1;kcnj11;rad54l;hadha;wnt2;slc22a18 |
| calcium-independent cell-cell adhesion; | 6#22 | 0.017302035 | cldn1;cldn14;cldn20;cldn17;cx3cl1;cldn2 |
| positive regulation of immune system process; | 13#86 | 0.01741238 | malt1;map3k7;myd88;tlr2;cfi;cacnb3;traf6;cfb;c4bpb;krt1;elf1;c1qc;tgfb2 |
| cellular calcium ion homeostasis; | 16#116 | 0.01845822 | ccr8;pth2r;atp2a2;tgfb1;prkca;edn1;ccr7;ccl28;kng1;ccr2;atp2c1;cacna1f;cckbr;mchr1;agtr1;ccr9 |
| calcium ion homeostasis; | 16#116 | 0.01845822 | ccr8;pth2r;atp2a2;tgfb1;prkca;edn1;ccr7;ccl28;kng1;ccr2;atp2c1;cacna1f;cckbr;mchr1;agtr1;ccr9 |
| protein autoprocessing; | 9#46 | 0.018568963 | mex3b;nlk;eif2ak2;taok3;asz1;map3k9;myo3a;wnt2;pak1 |
| RNA catabolic process; | 9#46 | 0.018568963 | exosc10;dcp2;dcp1a;upf3a;pabpc4;rnaseh1;exosc7;rnps1;isg20 |
| regulation of steroid biosynthetic process; | 3#5 | 0.018798288 | prkaa1;nr5a1;scap |
| nucleosome disassembly; | 3#5 | 0.018798288 | supt16h;tnp1;set |
| GPI anchor biosynthetic process#attachment of GPI anchor to protein; | 3#5 | 0.018798288 | pigu;gpaa1;pigs |
| chromatin disassembly; | 3#5 | 0.018798288 | supt16h;tnp1;set |
| response to lipopolysaccharide; | 3#5 | 0.018798288 | myd88;mapk1;thbd |
| T cell cytokine production; | 3#5 | 0.018798288 | malt1;map3k7;traf6 |
| regulation of T cell cytokine production; | 3#5 | 0.018798288 | malt1;map3k7;traf6 |
| lipoprotein biosynthetic process; | 10#55 | 0.018832269 | pigq;pigu;zdhhc7;mttp;atg7;gpaa1;rabggta;pigs;pigb;aipl1 |
| nucleotide metabolic process; | 36#340 | 0.019060665 | dck;edn1;nox1;qprt;grm8;paics;kynu;ucn2;atp6v1g3;pgd;ampd1;atp6v0b;npr2;g6pd;acly;dera;ampd3;adk;atp5o;upp1;papss1;nt5c1a;cda;naprt1;atp5b;gucy2c;ndufs1;cad;rrm2b;hibadh;adm;prps2;atp6v1d;rrm2;gmps;rexo2 |
| gene silencing; | 8#38 | 0.019252443 | sirt4;sirt5;tnp1;sirt1;xpo5;scmh1;hells;sirt3 |
| negative regulation of catalytic activity; | 13#87 | 0.019434213 | ccr2;pdcd4;trib3;prkca;rgs4;dusp22;galr1;gnai3;grm8;gabbr1;sfn;spry1;lats2 |
| amino acid metabolic process; | 47#474 | 0.019503295 | bhmt;slc7a7;tfe3;ears2;gfpt1;cbs;kynu;il20;th;tyr;bcat2;arg1;plod1;znfx1;agxt2l1;qdpr;mars2;ccnd3;wars;sms;uroc1;prkca;pepd;afmid;ssu72;farsa;wars2;il3;pycr1;hars;sars;cad;ddah1;hcls1;gch1;agmat;hibadh;nars;psph;socs1;gfpt2;pts;gmps;dbh;bckdhb;clcf1;coro2a |
| G-protein signaling, coupled to cyclic nucleotide second messenger; | 16#117 | 0.019823076 | adora2b;pth2r;gpr3;galr1;htr4;chrm2;gnai3;grm8;gnal;rgs1;ccr2;adra1b;mtnr1b;npr3;gabbr1;mchr1 |
| positive regulation of positive chemotaxis; | 4#10 | 0.019823076 | prkca;vegfa;azu1;cdh13 |
| positive chemotaxis; | 4#10 | 0.019823076 | prkca;vegfa;azu1;cdh13 |
| regulation of positive chemotaxis; | 4#10 | 0.019823076 | prkca;vegfa;azu1;cdh13 |
| response to bacterium#response to molecule of bacterial origin; | 4#10 | 0.019823076 | cd14;myd88;mapk1;thbd |
| catecholamine biosynthetic process; | 4#10 | 0.019823076 | tgfb2;pnmt;dbh;th |
| carbohydrate biosynthetic process; | 18#138 | 0.020482174 | slc25a10;uap1;gfpt1;c1qtnf2;chst12;ugdh;phkg2;pgd;b3galt2;chst8;lalba;gfpt2;cercam;b3galt1;fbp2;cog2;cmas;phkg1 |
| nucleobase metabolic process; | 6#23 | 0.020544795 | cad;cda;gmps;mapk1;paics;aprt |
| chromatin silencing; | 6#23 | 0.020544795 | sirt1;tnp1;sirt4;hells;sirt3;sirt5 |
| heterochromatin formation; | 6#23 | 0.020544795 | sirt1;tnp1;sirt4;hells;sirt3;sirt5 |
| macromolecular complex assembly; | 69#756 | 0.021538748 | brf2;ptk2;h2afz;nck1;lin7a;matn1;gga3;med23;aldh5a1;capza2;gpaa1;tspyl1;cdk9;hspb1;sf3a2;ap1s3;set;tbpl2;ap1g1;ranbp9;cldn14;tmem48;nubp1;taf4;ap3d1;capg;eif4h;eif3f;crnkl1;chaf1a;cd74;gja1;nap1l5;slc7a7;il1rap;copg2;eid2;ap3s1;xpo6;traf1;snrpe;eif1b;mki67ip;usp39;kpna3;med24;taf6;eif3e;gtf2a1;tubgcp4;eif1ax;ctnna1;malt1;tspyl2;rad51;cda;picalm;eif4g2;c1qtnf2;denr;napg;skap2;eif3i;trpv5;prpf6;adrm1;cct6b;eif5;gtf2e1 |
| nucleobase, nucleoside and nucleotide metabolic process; | 38#367 | 0.021993561 | dck;edn1;nox1;qprt;grm8;paics;kynu;ucn2;atp6v1g3;pgd;ampd1;atp6v0b;npr2;g6pd;acly;dera;aprt;ampd3;adk;atp5o;upp1;mapk1;papss1;nt5c1a;cda;naprt1;atp5b;gucy2c;ndufs1;cad;rrm2b;hibadh;adm;prps2;atp6v1d;rrm2;gmps;rexo2 |
| T cell activation; | 14#98 | 0.022269042 | malt1;tgfb1;map3k7;nck1;cd28;traf6;cd74;ap3d1;ripk2;bcl6;vsig4;cd2;pag1;cd274 |
| immune system development; | 22#182 | 0.022269042 | tgfb1;mmp9;vps33a;cd74;bcl6;ctnnb1;il3;cd2;map4k2;tgfb2;cdk6;hcls1;runx1;lep;kitlg;bpgm;ap3d1;klf1;hdac9;snrk;zbtb16;clcf1 |
| monovalent inorganic cation transport; | 51#528 | 0.022946385 | btbd10;kcns3;kcnj13;slc17a4;kcnc1;nox1;kcnj12;kcnk2;slc22a4;kcnj11;kctd9;kcng1;slc5a3;cnga3;atp6v1g3;scn1a;kcnab3;slc13a1;atp6v0b;kcnv2;scnn1b;slc8a2;kctd8;kcnk1;kctd19;atp5o;atp2a2;slc9a5;kcna2;kctd18;kcnq1;kcnk6;kcne1;kcnip4;kctd12;nedd4l;atp5b;slc9a7;kcnj5;slc10a5;kcnmb4;slc9a8;slc38a4;hcn4;kcnq2;scn1b;kcnf1;slc23a2;atp6v1d;kcna5;kcns1 |
| blood vessel development#blood vessel morphogenesis#angiogenesis; | 17#129 | 0.023458664 | nrp1;adora2b;vegfa;edn1;cdh13;fgf6;ctnnb1;tgfb2;klf5;runx1;fgf1;rhob;ihh;flt1;krt1;gna13;serpinf1 |
| steroid biosynthetic process; | 13#89 | 0.024805394 | srd5a1;cnbp;fdps;prkaa1;sc5dl;nr5a1;adm;fdxr;prkag2;mvd;prkaa2;scap;stard5 |
| regulation of biosynthetic process; | 25#217 | 0.025025485 | hspb1;prkaa1;nr5a1;cd28;edn1;bace2;ghsr;eif4g2;grm8;azu1;c1qtnf2;eif3i;samd4a;eif1b;pum2;myd88;pstk;igf2bp3;aplp1;eif3e;hsp90aa1;eif4h;eif5;eif3f;scap |
| positive regulation of I-kappaB kinase/NF-kappaB cascade; | 14#99 | 0.025025485 | sectm1;malt1;ltbr;atp2c1;myd88;trim38;traf6;gja1;ripk2;eef1d;traf3ip2;lgals9;slc20a1;trim13 |
| protein processing; | 14#99 | 0.025025485 | pcsk7;mex3b;nlk;eif2ak2;ncln;taok3;ihh;bace2;asz1;spcs2;myo3a;map3k9;wnt2;pak1 |
| positive regulation of progression through cell cycle; | 6#24 | 0.025047025 | ccnd3;tgfb2;cd28;cited2;shc1;nusap1 |
| mesenchymal cell differentiation#mesenchymal cell development; | 6#24 | 0.025047025 | ctnnb1;sox9;tgfb1;tgfb2;foxc1;edn1 |
| mesenchymal cell differentiation; | 6#24 | 0.025047025 | ctnnb1;sox9;tgfb1;tgfb2;foxc1;edn1 |
| endosome transport; | 8#40 | 0.025194019 | sqstm1;rab9b;ankrd27;gosr1;rhob;vps26a;stx16;ykt6 |
| T cell receptor signaling pathway; | 5#17 | 0.025350468 | elf1;malt1;map3k7;cacnb3;traf6 |
| aromatic amino acid family catabolic process; | 5#17 | 0.025350468 | pts;afmid;gch1;qdpr;kynu |
| hemopoiesis#myeloid cell differentiation; | 12#77 | 0.025680746 | mmp9;hcls1;vps33a;lep;runx1;bpgm;bcl6;klf1;ctnnb1;snrk;zbtb16;cdk6 |
| immune response-activating signal transduction; | 7#32 | 0.025680746 | elf1;malt1;map3k7;myd88;tlr2;cacnb3;traf6 |
| tricarboxylic acid cycle intermediate metabolic process; | 7#32 | 0.025680746 | me2;sucla2;aco1;ldha;acly;ldhb;ldhc |
| ceramide metabolic process; | 7#32 | 0.025680746 | neu3;asah2;prkaa1;hexa;aldh5a1;ugcg;sgpl1 |
| neurological system process#transmission of nerve impulse#synaptic transmission#regulation of neurotransmitter levels#neurotransmitter metabolic process; | 7#32 | 0.025680746 | maoa;tgfb2;sncb;gch1;aldh5a1;th;nr4a2 |
| morphogenesis of a branching structure; | 7#32 | 0.025680746 | ctnnb1;gzf1;flt1;tgfb1;gna13;edn1;ihh |
| regulation of cyclin-dependent protein kinase activity; | 9#49 | 0.025680746 | ccnd3;cdc37;ccng1;cdkn2d;cdkn3;sfn;diras3;ccnt2;lats2 |
| glycosaminoglycan metabolic process; | 9#49 | 0.025680746 | foxc1;galns;gns;chst1;hyal2;hgsnat;hexa;chst12;ugdh |
| regulation of DNA metabolic process; | 9#49 | 0.025680746 | pcna;tspyl2;polh;kpna2;rad51;hus1;tlk1;cdt1;set |
| sphingolipid metabolic process; | 11#68 | 0.026943855 | arv1;neu3;prkaa1;ugcg;psap;asah2;hexa;serinc1;aldh5a1;st8sia5;sgpl1 |
| biogenic amine catabolic process; | 4#11 | 0.026943855 | dio3;maoa;afmid;kynu |
| estrogen receptor signaling pathway; | 4#11 | 0.026943855 | rbm14;ddx54;egln2;ppargc1b |
| dopamine metabolic process; | 4#11 | 0.026943855 | maoa;tgfb2;sncb;nr4a2 |
| positive regulation of chemotaxis; | 4#11 | 0.026943855 | prkca;vegfa;azu1;cdh13 |
| cartilage development#cartilage condensation; | 4#11 | 0.026943855 | sox9;matn1;snai1;alx1 |
| epithelial to mesenchymal transition; | 4#11 | 0.026943855 | ctnnb1;sox9;tgfb1;tgfb2 |
| response to protein stimulus; | 10#59 | 0.027384516 | hspb1;eif2ak2;vapb;hsph1;cfb;hspa4;ern1;hspd1;hsp90aa1;hspa1l |
| response to unfolded protein; | 10#59 | 0.027384516 | hspb1;eif2ak2;vapb;hsph1;cfb;hspa4;ern1;hspd1;hsp90aa1;hspa1l |
| regulation of T cell activation; | 10#59 | 0.027384516 | malt1;map3k7;nck1;cd28;traf6;bcl6;vsig4;cd2;pag1;cd274 |
| chromosome segregation; | 10#59 | 0.027384516 | ncaph;ndc80;stag1;sycp3;bub3;sgol1;akap8;ncapg;srpk1;nusap1 |
| cytosine metabolic process; | 2#2 | 0.027384516 | cda;mapk1 |
| glucosylceramide biosynthetic process; | 2#2 | 0.027384516 | prkaa1;ugcg |
| hormone transport; | 2#2 | 0.027384516 | shbg;slc6a2 |
| GO:0030818,negative regulation of cAMP biosynthetic process; | 2#2 | 0.027384516 | grm8;edn1 |
| response to fungus#response to molecule of fungal origin; | 2#2 | 0.027384516 | myd88;tlr2 |
| cellular pigmentation#pigment granule organization and biogenesis; | 2#2 | 0.027384516 | shroom2;cno |
| melanosome organization and biogenesis; | 2#2 | 0.027384516 | shroom2;cno |
| monocyte activation; | 2#2 | 0.027384516 | azu1;spaca3 |
| primary sex determination; | 2#2 | 0.027384516 | sox9;nr5a1 |
| meiotic chromosome segregation; | 2#2 | 0.027384516 | sgol1;sycp3 |
| maintenance of DNA methylation; | 2#2 | 0.027384516 | ctcf;hells |
| MyD88-dependent toll-like receptor signaling pathway; | 2#2 | 0.027384516 | myd88;tlr2 |
| negative regulation of cAMP metabolic process; | 2#2 | 0.027384516 | grm8;edn1 |
| regulation of protein metabolic process; | 42#422 | 0.027384516 | trim54;nck1;egln2;cd28;aurkaip1;gja1;azu1;il20;capza2;eif1b;myd88;serpina5;capza3;plaur;igf2bp3;eif3e;scap;hspb1;tgfb1;prkca;bace2;il3;cdc42ep5;ghsr;nedd4l;eif4g2;set;eif3i;samd4a;pum2;hcls1;pstk;capg;socs1;aplp1;eif4h;rasa1;clcf1;eif3f;eif5;ift88;ube2m |
| oxidoreduction coenzyme metabolic process; | 9#50 | 0.027685748 | pdss2;pgd;hibadh;pdss1;nox1;g6pd;naprt1;qprt;kynu |
| aminoglycan metabolic process; | 9#50 | 0.027685748 | foxc1;galns;gns;chst1;hyal2;hgsnat;hexa;chst12;ugdh |
| anatomical structure morphogenesis#anatomical structure formation; | 19#153 | 0.027779846 | nrp1;adora2b;nle1;vegfa;foxc1;edn1;cdh13;fgf6;ctnnb1;tgfb2;klf5;runx1;fgf1;rhob;ihh;flt1;krt1;gna13;serpinf1 |
| regulation of JNK activity; | 6#25 | 0.028126532 | pdcd4;dbnl;gab1;map3k9;pak1;map3k2 |
| pigmentation; | 6#25 | 0.028126532 | tyr;vps33a;shroom2;ap3d1;hps6;cno |
| exocytosis; | 15#111 | 0.028200751 | scfd1;uso1;vps33a;pram1;snph;lin7a;traf7;doc2a;exoc3;rapgef4;scrn1;tnp2;arfgef2;stxbp1;ykt6 |
| response to light stimulus; | 12#81 | 0.028200751 | fech;nras;adra1b;hus1;opn1sw;cops3;cacna1f;cdkn2d;brsk1;fen1;clock;pdc |
| immune response-regulating signal transduction; | 7#33 | 0.028398219 | elf1;malt1;map3k7;myd88;tlr2;cacnb3;traf6 |
| macromolecule catabolic process; | 58#625 | 0.028519832 | exosc10;gaa;egln2;upf3a;spaca3;gja1;usp30;psmd14;pgam2;psmc2;uchl5;tprkb;fut1;pgd;uhrf2;usp39;bpgm;g6pd;prtn3;syvn1;usp2;usp32;tktl1;exosc7;psma8;fbxl4;rnps1;pglyrp3;dcp2;dcp1a;pepd;mmp9;dffa;usp15;cln5;sqstm1;nedd4l;ldha;hk3;psmc5;isg20;arih1;psmb5;gnpda1;adra1b;pdha1;pabpc4;hibadh;ldhc;gns;usp33;usp11;hyal2;ldhb;ogdh;rnaseh1;usp13;pgk2 |
| ion homeostasis; | 29#267 | 0.029126165 | ccr8;pth2r;atp2a2;tgfb1;slc9a5;prkca;chrne;edn1;slc30a1;cln5;ccr7;nedd4l;slc9a7;ccl28;kng1;ndufs1;kcnmb4;ccr2;atp2c1;hamp;slc9a8;tfrc;cacna1f;rhcg;kcna5;cckbr;ccr9;mchr1;agtr1 |
| regulation of T cell mediated immunity; | 3#6 | 0.029126165 | malt1;map3k7;traf6 |
| adult feeding behavior; | 3#6 | 0.029126165 | ghsr;lep;brs3 |
| tetrahydrobiopterin biosynthetic process; | 3#6 | 0.029126165 | pts;gch1;qdpr |
| negative regulation of nucleotide metabolic process; | 3#6 | 0.029126165 | cda;grm8;edn1 |
| positive regulation of cytokine production during immune response; | 3#6 | 0.029126165 | malt1;map3k7;traf6 |
| positive regulation of production of molecular mediator of immune response; | 3#6 | 0.029126165 | malt1;map3k7;traf6 |
| skin development; | 3#6 | 0.029126165 | ngfr;sfn;krt9 |
| tyrosine phosphorylation of STAT protein; | 5#18 | 0.029273108 | socs1;hcls1;clcf1;il20;stat1 |
| positive regulation of endocytosis; | 5#18 | 0.029273108 | ldlrap1;nedd4l;azu1;spaca3;actn4 |
| nucleocytoplasmic transport; | 19#154 | 0.029343354 | malt1;tgfb1;kpna2;stat1;bcl6;kpna6;ran;xpo6;set;camk4;kpna3;cep57;rae1;nfkbil1;gle1;nxf1;ddx19b;gsk3b;nupl2 |
| cyclic-nucleotide-mediated signaling; | 16#122 | 0.029343354 | adora2b;pth2r;gpr3;galr1;htr4;chrm2;gnai3;grm8;gnal;rgs1;ccr2;adra1b;mtnr1b;npr3;gabbr1;mchr1 |
| regulation of cell morphogenesis; | 8#42 | 0.029804186 | taok2;gas2;cdc42ep4;cyfip1;gna13;cdc42ep5;fgd1;rasa1 |
| regulation of anatomical structure morphogenesis; | 8#42 | 0.029804186 | taok2;gas2;cdc42ep4;cyfip1;gna13;cdc42ep5;fgd1;rasa1 |
| regulation of cell shape; | 8#42 | 0.029804186 | taok2;gas2;cdc42ep4;cyfip1;gna13;cdc42ep5;fgd1;rasa1 |
| amino acid derivative biosynthetic process; | 8#42 | 0.029804186 | tgfb2;agmat;pnmt;dbh;ckm;srm;amd1;th |
| generation of neurons#neuron differentiation; | 24#210 | 0.029848083 | nrp1;fez1;pou3f2;gja1;dscaml1;cln5;cyfip1;pou4f3;tgfb2;tlx3;b3gnt2;mdga1;nr4a2;slitrk4;slitrk3;efnb3;ngfr;cacna1f;cck;sema4d;mdga2;dcc;nrcam;dlx5 |
| transmembrane receptor protein tyrosine kinase signaling pathway; | 24#210 | 0.029848083 | ptk2;gab1;prkca;nck1;vegfa;gfra2;shc1;foxc1;cdh13;epha3;ghsr;ap3s1;sparc;rapgef1;grb10;ddr2;efnb3;epha1;cep57;flt1;socs1;fgf5;timp3;cnksr1 |
| elevation of cytosolic calcium ion concentration; | 11#70 | 0.030262961 | ccr2;ccr8;tgfb1;edn1;ccr7;cckbr;kng1;agtr1;mchr1;ccl28;ccr9 |
| cytosolic calcium ion homeostasis; | 11#70 | 0.030262961 | ccr2;ccr8;tgfb1;edn1;ccr7;cckbr;kng1;agtr1;mchr1;ccl28;ccr9 |
| tRNA processing; | 13#92 | 0.031394099 | rpp30;trub2;pop5;adat3;rpp14;trub1;dus1l;trmu;mettl1;adat1;nsun2;rpp38;sars |
| lymphocyte activation; | 19#155 | 0.031506316 | malt1;tgfb1;nck1;cd28;traf6;cd74;ripk2;bcl6;pag1;cd2;hells;skap2;map3k7;ap3d1;vsig4;hdac9;cd274;slamf1;clcf1 |
| biogenic amine biosynthetic process; | 7#34 | 0.032025595 | tgfb2;agmat;pnmt;dbh;srm;amd1;th |
| meiotic cell cycle; | 11#71 | 0.033479794 | rad51;npm2;tubg1;sycp3;dmc1;xrcc2;rad54l;sgol1;dusp13;rad1;mns1 |
| cytokine and chemokine mediated signaling pathway; | 8#43 | 0.033858848 | ccr2;stat3;jak1;myd88;cx3cl1;il3;socs1;clcf1 |
| negative regulation of epithelial cell proliferation; | 4#12 | 0.033858848 | tgfb1;tgfb2;cdk6;runx3 |
| chemotaxis#regulation of chemotaxis; | 4#12 | 0.033858848 | prkca;vegfa;azu1;cdh13 |
| male gamete generation; | 24#212 | 0.033886684 | gfer;ccin;bcl6;spata9;dmc1;gpx4;dedd;pmch;dusp13;tnp2;serpina5;capza3;nr6a1;cct6b;sycp3;tnp1;cep57;cylc1;ift81;crem;adad1;tsga10;dazap1;krt9 |
| spermatogenesis; | 24#212 | 0.033886684 | gfer;ccin;bcl6;spata9;dmc1;gpx4;dedd;pmch;dusp13;tnp2;serpina5;capza3;nr6a1;cct6b;sycp3;tnp1;cep57;cylc1;ift81;crem;adad1;tsga10;dazap1;krt9 |
| phosphorylation; | 115#1394 | 0.033886684 | ttbk1;ptk2;hunk;nek8;hus1;asz1;myo3a;map3k9;nrbp1;epha3;mark2;rapgef4;lrrk1;twf1;uqcrh;camk2d;lats2;prkra;trib3;map3k7;map3k4;camk2g;cdk2ap1;flt1;btk;mvd;ipmk;mapkapk5;phkg1;glyctk;ccnd3;atp5o;cdk9;prkca;taok2;tec;cdc42bpb;taok3;tlk1;il3;adam10;vrk1;pdk2;csnk1a1;pim1;ndufs1;pak3;phkg2;ddr2;camk4;pip4k2a;ulk3;atp6v1d;socs1;cdk10;tnks;csnk1g3;snrk;wnt2;clk3;oxsr1;araf;akt2;ndufb9;ripk2;ern1;cdkn2d;wee2;il20;mex3b;nlk;itk;atp6v0b;npr2;epha1;dmpk;prkci;srms;pak1;pskh1;tp53rk;abl2;map3k2;axl;tgfb1;jak1;mapk1;prkaa1;eif2ak2;dyrk1b;abi2;stat1;map2k7;acvr2b;map4k3;brsk1;map4k2;atp5b;mark1;cdkl2;gucy2c;srpk1;aurkc;cdk6;nuak1;hcls1;bub1b;agk;aurkb;ndufv1;stk39;clcf1;prkaa2;gsk3b;pgk2 |
| nuclear transport; | 19#156 | 0.033886684 | malt1;tgfb1;kpna2;stat1;bcl6;kpna6;ran;xpo6;set;camk4;kpna3;cep57;rae1;nfkbil1;gle1;nxf1;ddx19b;gsk3b;nupl2 |
| carboxylic acid biosynthetic process; | 12#83 | 0.034709519 | aloxe3;fa2h;prkaa1;cd74;acaca;prkag2;alox12;fasn;prkaa2;scd;ugdh;elovl7 |
| organic acid biosynthetic process; | 12#83 | 0.034709519 | aloxe3;fa2h;prkaa1;cd74;acaca;prkag2;alox12;fasn;prkaa2;scd;ugdh;elovl7 |
| glycosphingolipid metabolic process; | 5#19 | 0.035558364 | psap;neu3;hexa;st8sia5;ugcg |
| odontogenesis of dentine-containing teeth; | 5#19 | 0.035558364 | ctnnb1;ngfr;foxc1;amtn;ift88 |
| spindle organization and biogenesis; | 5#19 | 0.035558364 | ran;ndc80;bub1b;rcc1;tubg1 |
| sphingoid metabolic process; | 7#35 | 0.036719931 | neu3;asah2;prkaa1;hexa;aldh5a1;ugcg;sgpl1 |
| cytoskeleton organization and biogenesis; | 62#686 | 0.037393458 | trim54;nck1;kpna2;kif1c;daam2;kif9;actn4;asz1;ran;capza2;ykt6;tmod1;kif3b;atp2c1;arhgap1;ndc80;cntrob;rcc1;capza3;sgcb;tubgcp5;arhgdib;elmo1;ppp4c;krt20;tubgcp4;kif3a;fgd1;prkci;pak1;des;nusap1;tubb6;nras;taok2;kif18a;abi2;ablim1;mid1;pld2;cdc42bpb;bcl6;fscn1;cdc42ep5;dync1i1;ghsr;map4k2;mark1;tubgcp3;hook3;sgcg;ranbp9;nubp1;hcn4;bub1b;kifc3;capg;tubg1;shank1;wnt2;rasa1;krt9 |
| nucleobase, nucleoside, nucleotide and nucleic acid transport; | 14#104 | 0.037704191 | tmem48;upf3a;gja1;senp2;slc23a2;nup35;slc29a3;rae1;tnks;gle1;ran;nxf1;ddx19b;nupl2 |
| cellular chemical homeostasis; | 26#237 | 0.037704191 | ccr8;pth2r;atp2a2;tgfb1;prkca;chrne;edn1;slc30a1;cln5;ccr7;nedd4l;ccl28;kng1;ndufs1;kcnmb4;ccr2;atp2c1;hamp;tfrc;cacna1f;rhcg;kcna5;cckbr;ccr9;mchr1;agtr1 |
| cellular ion homeostasis; | 26#237 | 0.037704191 | ccr8;pth2r;atp2a2;tgfb1;prkca;chrne;edn1;slc30a1;cln5;ccr7;nedd4l;ccl28;kng1;ndufs1;kcnmb4;ccr2;atp2c1;hamp;tfrc;cacna1f;rhcg;kcna5;cckbr;ccr9;mchr1;agtr1 |
| positive regulation of amine metabolic process; | 6#27 | 0.037704191 | ccnd3;il3;prkca;hcls1;clcf1;il20 |
| positive regulation of amino acid metabolic process; | 6#27 | 0.037704191 | ccnd3;il3;prkca;hcls1;clcf1;il20 |
| myeloid leukocyte activation; | 6#27 | 0.037704191 | cd2;azu1;pram1;spaca3;cd74;cd93 |
| mitotic cell cycle checkpoint; | 6#27 | 0.037704191 | mad2l2;tgfb1;bub3;brsk1;bub1b;hus1 |
| positive regulation of protein amino acid phosphorylation; | 6#27 | 0.037704191 | ccnd3;il3;prkca;hcls1;clcf1;il20 |
| lipoprotein metabolic process; | 12#84 | 0.038645103 | pigq;pigu;zdhhc7;scarf1;mttp;atg7;gpaa1;rabggta;pigs;pigb;apobec1;aipl1 |
| fatty acid biosynthetic process; | 11#73 | 0.039325821 | aloxe3;fa2h;prkaa1;cd74;acaca;prkag2;alox12;fasn;prkaa2;scd;elovl7 |
| positive regulation of multicellular organismal process; | 14#105 | 0.04176372 | malt1;map3k7;myd88;tlr2;cfi;adra1b;cacnb3;traf6;cfb;c4bpb;krt1;elf1;c1qc;tgfb2 |
| generation of neurons#neuron migration; | 7#36 | 0.041852314 | asz1;cck;tlx3;wnt2;gja1;mdga1;nrcam |
| protein targeting to membrane; | 7#36 | 0.041852314 | srp54;srp72;taok2;rtp4;prkci;atg4a;rtp3 |
| water-soluble vitamin metabolic process; | 10#64 | 0.04220884 | pgd;hibadh;nox1;g6pd;slc23a2;naprt1;mmab;qprt;tktl1;kynu |
| phosphorus metabolic process; | 136#1697 | 0.04220884 | ttbk1;ptk2;hunk;asz1;slc20a1;myo3a;map3k9;nrbp1;mark2;rapgef4;dusp10;camk2d;prkra;map3k7;map3k4;cdk2ap1;glyctk;atp5o;cdk9;inppl1;taok2;tec;cdc42bpb;tlk1;il3;vrk1;pdk2;pim1;ndufs1;ptpn18;phkg2;ddr2;ptpn21;ulk3;tnks;csnk1g3;akt2;ern1;cdkn2d;mtmr7;mex3b;nlk;atp6v0b;npr2;enpp2;ppp1cb;prkci;pak1;tp53rk;abl2;map3k2;axl;tgfb1;mapk1;dyrk1b;dusp22;map2k7;acvr2b;ppm1l;ppm1k;impa2;map4k2;mark1;atp5b;cdkl2;gucy2c;dusp13;aurkc;ppp1cc;stk39;clcf1;prkaa2;gsk3b;pgk2;nek8;slc17a4;minpp1;hus1;epha3;uqcrh;twf1;lrrk1;lats2;trib3;cdc14b;camk2g;flt1;btk;mvd;mapkapk5;ipmk;phkg1;ccnd3;prkca;ppm1d;taok3;adam10;csnk1a1;pak3;camk4;pip4k2a;atp6v1d;cdk10;socs1;wnt2;snrk;clk3;oxsr1;araf;ndufb9;ripk2;cdkn3;wee2;il20;itk;epha1;dmpk;srms;pskh1;jak1;eif2ak2;prkaa1;abi2;stat1;map4k3;brsk1;nuak1;cdk6;srpk1;hcls1;bub1b;impa1;agk;aurkb;inpp1;ndufv1 |
| phosphate metabolic process; | 136#1697 | 0.04220884 | ttbk1;ptk2;hunk;asz1;slc20a1;myo3a;map3k9;nrbp1;mark2;rapgef4;dusp10;camk2d;prkra;map3k7;map3k4;cdk2ap1;glyctk;atp5o;cdk9;inppl1;taok2;tec;cdc42bpb;tlk1;il3;vrk1;pdk2;pim1;ndufs1;ptpn18;phkg2;ddr2;ptpn21;ulk3;tnks;csnk1g3;akt2;ern1;cdkn2d;mtmr7;mex3b;nlk;atp6v0b;npr2;enpp2;ppp1cb;prkci;pak1;tp53rk;abl2;map3k2;axl;tgfb1;mapk1;dyrk1b;dusp22;map2k7;acvr2b;ppm1l;ppm1k;impa2;map4k2;mark1;atp5b;cdkl2;gucy2c;dusp13;aurkc;ppp1cc;stk39;clcf1;prkaa2;gsk3b;pgk2;nek8;slc17a4;minpp1;hus1;epha3;uqcrh;twf1;lrrk1;lats2;trib3;cdc14b;camk2g;flt1;btk;mvd;mapkapk5;ipmk;phkg1;ccnd3;prkca;ppm1d;taok3;adam10;csnk1a1;pak3;camk4;pip4k2a;atp6v1d;cdk10;socs1;wnt2;snrk;clk3;oxsr1;araf;ndufb9;ripk2;cdkn3;wee2;il20;itk;epha1;dmpk;srms;pskh1;jak1;eif2ak2;prkaa1;abi2;stat1;map4k3;brsk1;nuak1;cdk6;srpk1;hcls1;bub1b;impa1;agk;aurkb;inpp1;ndufv1 |
| sperm motility; | 5#20 | 0.04220884 | asz1;tnp1;wnt2;tnp2;akap4 |
| regulation of cell-cell adhesion; | 4#13 | 0.04220884 | fxyd5;cx3cl1;rasa1;bcl6 |
| regulation of cell-matrix adhesion; | 4#13 | 0.04220884 | cdh13;rasa1;cdk6;bcl6 |
| wound healing#blood coagulation#regulation of blood coagulation; | 4#13 | 0.04220884 | krt1;apoh;kng1;gp1ba |
| regulation of adaptive immune response based on somatic recombination of immune receptors built from immunoglobulin superfamily domains.; | 4#13 | 0.04220884 | malt1;map3k7;traf6;bcl6 |
| regulation of tyrosine phosphorylation of STAT protein; | 4#13 | 0.04220884 | socs1;hcls1;clcf1;il20 |
| regulation of striated muscle development; | 4#13 | 0.04220884 | sox15;hdac9;tgfb1;gja1 |
| regulation of adaptive immune response; | 4#13 | 0.04220884 | malt1;map3k7;traf6;bcl6 |
| tumor necrosis factor production; | 4#13 | 0.04220884 | ghsr;myd88;tlr2;azu1 |
| blood vessel remodeling; | 3#7 | 0.04220884 | tgfb2;adra1b;foxc1 |
| GO:0042531,positive regulation of tyrosine phosphorylation of STAT protein; | 3#7 | 0.04220884 | hcls1;clcf1;il20 |
| establishment and/or maintenance of epithelial cell polarity; | 3#7 | 0.04220884 | mark2;prickle2;prkci |
| positive regulation of JAK-STAT cascade; | 3#7 | 0.04220884 | hcls1;clcf1;il20 |
| erythrocyte differentiation#erythrocyte development; | 3#7 | 0.04220884 | bpgm;bcl6;klf1 |
| positive regulation of catabolic process; | 3#7 | 0.04220884 | egln2;adra1b;gja1 |
| epidermis morphogenesis#epidermal cell differentiation#regulation of epidermal cell differentiation; | 3#7 | 0.04220884 | maff;keap1;il20 |
| tetrahydrobiopterin metabolic process; | 3#7 | 0.04220884 | pts;gch1;qdpr |
| arginine catabolic process; | 3#7 | 0.04220884 | ddah1;arg1;agmat |
| regulation of the force of heart contraction; | 3#7 | 0.04220884 | atp2a2;prkca;adra1b |
| water homeostasis; | 3#7 | 0.04220884 | nedd4l;edn1;kng1 |
| regulation of peptidyl-tyrosine phosphorylation; | 6#28 | 0.042655549 | il3;socs1;prkca;hcls1;clcf1;il20 |
| branching morphogenesis of a tube; | 6#28 | 0.042655549 | ctnnb1;gzf1;flt1;gna13;edn1;ihh |
| mitotic sister chromatid segregation; | 6#28 | 0.042655549 | ncaph;bub3;ndc80;akap8;ncapg;nusap1 |
| gamete generation; | 28#264 | 0.044161959 | tgfb1;gfer;npm2;spin1;foxc1;ccin;bcl6;spata9;dmc1;gpx4;dedd;pmch;dusp13;tnp2;serpina5;capza3;nr6a1;cct6b;sycp3;tnp1;cep57;cylc1;ift81;crem;adad1;tsga10;dazap1;krt9 |
| regulation of amine metabolic process; | 8#46 | 0.045936179 | ccnd3;bhmt;prkca;hcls1;il3;socs1;il20;clcf1 |
| regulation of amino acid metabolic process; | 8#46 | 0.045936179 | ccnd3;bhmt;prkca;hcls1;il3;socs1;il20;clcf1 |
| feeding behavior; | 7#37 | 0.045956691 | ghsr;cck;cckbr;lep;pmch;mchr1;brs3 |
| vitamin metabolic process; | 12#86 | 0.046849747 | dhrs9;pgd;rdh11;hibadh;nox1;g6pd;slc23a2;naprt1;mmab;qprt;tktl1;kynu |
| cell cycle checkpoint; | 9#56 | 0.048943484 | tgfb1;bub1b;hus1;mad2l2;cdt1;brsk1;bub3;rad1;ddb1 |
| chemical homeostasis; | 31#302 | 0.049085895 | ccr8;pth2r;atp2a2;tgfb1;slc9a5;prkca;chrne;edn1;slc30a1;cln5;ccr7;nedd4l;slc9a7;ccl28;kng1;ndufs1;kcnmb4;ccr2;atp2c1;hamp;ldlrap1;slc9a8;adra1b;tfrc;cacna1f;rhcg;kcna5;cckbr;ccr9;agtr1;mchr1 |
| regulation of lymphocyte activation; | 11#76 | 0.049328826 | malt1;tgfb1;map3k7;nck1;cd28;traf6;bcl6;vsig4;cd2;pag1;cd274 |
| positive regulation of MAP kinase activity; | 10#66 | 0.04970096 | adora2b;gab1;prkaa1;shc1;madd;dbnl;map3k9;c1qtnf2;pak1;map3k2 |
| chromosome segregation#sister chromatid segregation; | 6#29 | 0.049931444 | ncaph;bub3;ndc80;akap8;ncapg;nusap1 |
